# Using multiple linear regression and BP neural network to predict critical meteorological conditions of expressway bridge pavement icing

**DOI:** 10.1371/journal.pone.0263539

**Published:** 2022-02-04

**Authors:** Shuo Han, Jinliang Xu, Menghua Yan, Zhaoxin Liu

**Affiliations:** 1 College of Transportation Engineering, Chang’an University, Xi’an, China; 2 School of Highway, Chang’an University, Xi’an, China; 3 Shandong Hi-speed Infrastructure Construction Co., Ltd., Jinan, China; Tongji University, CHINA

## Abstract

Icy bridge deck in winter has tremendous consequences for expressway traffic safety, which is closely related to the bridge pavement temperature. In this paper, the critical meteorological conditions of icy bridge deck were predicted by multiple linear regression and BP neural network respectively. Firstly, the main parameters affecting the bridge pavement temperature were determined by Pearson partial correlation analysis based on the three-year winter meteorological data of the traffic meteorological monitoring station on the bridge in Shandong province. Secondly, the bridge pavement temperature is selected as the dependent variable, while air temperature, wind speed, relative humidity, dew point temperature, wet bulb temperature and wind cold temperature were selected as independent variables, and the bridge pavement temperature prediction models of linear regression and 5-layer hidden layer classical BP neural network regression were established respectively based on whether the variables are linear or not. Finally, the prediction accuracy of the above models was compared by using the measured data. The results show that the linear regression model could be established only with air temperature, relative humidity and wind speed, owing to collinearity problem. Compared with multiple linear regression model, the predicted value of the BP neural network has a higher degree of fitting with the measured data, and the coefficient of determination reaches 0.7929. Using multiple linear regression and BP neural network, the critical meteorological conditions of bridge deck icing in winter can be effectively predicted even when the sample size is insufficient.

## 1. Introduction

Icy bridge deck seriously affects traffic safety in winter. According to the statistics of the federal highway administration, there were 156164 collision accidents caused by icy roads from 2007 to 2016 [1]. The state and local agencies in the United States spend more than $2.3 billion on ice and snow control operations annually [2]. Bridges, key transportation network elements, are the first sections of the road network to experience freezing temperatures [3]. Thus, it is essential to predict bridge pavement temperature accurately for expressway icing early warning.

Since the 1960s, Countries such as Europe and the United States have carried out extensive research on the temperature distribution and temperature effects of bridges, and formulated corresponding specifications, which are widely concentrated on bridge temperature loads [4]. Seasonal climatic factors, weather changes and other external environments could lead to complex temperature changes in bridge structures, causing temperature stress and affecting bridge performance.

Temperature is the key factor to determine road icing. Expressway road icing should meet two conditions: pavement temperature is less than 0°C and precipitation occurs [5, 6]. To accurately predict road icing, researchers generally start with pavement temperature prediction [7, 8], and have established various temperature prediction models by using finite element simulation, theoretical analysis and statistical analysis, including numerical and empirical models [9–12].

Numerical models are mostly based on the principle of energy balance to predict the pavement temperature. The heat exchange between the road and the atmosphere, as well as the influence of the bridge on the radiation were considered by Barker et al., and the road temperature prediction formula based on surface energy balance was established [13]. According to ground heat conduction and energy balance equations, prediction models for road temperature and icing thickness were created by Sass [14]. Based on the existing theoretical models of Sass and Baker, A numerical prediction model for automatic icing of roads in northern Europe and North America was developed and the temperature distribution could be predicted with meteorological factors as input variables [15]. Considering heat conservation, Xiao et al. used the finite element method and theoretical analysis method to study the temperature distribution model of the bridge deck [16]. Due to complex parameters and complicated calculation process, the numerical model needs to be simplified in varying degrees.

Statistical analysis methods generally were used in Empirical models to predict pavement temperatures based on meteorological data. To predict road icing, in the strategic highway research program of the United States in 1987, meteorological statistics were applied to predict road surface temperature on a large scale for the first time [17]. Without complex numerical computations, empirical models are widely used in practice [6, 18], such as neural networks and linear regression models. The pavement temperature prediction model developed by Shao based on back propagation neural network was used for road icing prediction in winter [19]. Later, the artificial neural network was used to predict the road surface temperature within 3–12 hours, so as to achieve the purpose of short-term road icing prediction [20]. Considered the pavement temperature and meteorological factors affecting pavement icing, Xu et al. built the winter pavement temperature prediction model through improved BP neural network [21]. The neural network has large-scale data processing ability and nonlinear algorithm, while it can not explain the reasoning basis and the calculation is complicated.

To simplify data processing, some researchers have focused on the linear prediction model of pavement temperature based on the linear correlation between variables [22, 23]. Based on field test, Wang et al. established a linear regression model of the minimum bridge pavement temperature, minimum air temperature, and radiation. There are multiple factors affecting the bridge pavement temperature, and the model cannot accurately reflect the impact of the meteorological environment on the bridge pavement temperature [24]. With the minimum air temperature, solar radiation, wind speed, humidity and pavement depth as independent variables, the difference between the bridge deck and the pavement temperature variation characteristics was analyzed, and a linear regression model for predicting the minimum temperature of the bridge deck/pavement layer was established by Hou et al. [25]. Using data from nearby weather stations, the model cannot accurately measure the weather changes of bridges.

Clearly, the existing bridge deck/pavement temperature prediction models are generally computationally complex and require huge data support. Compared with the pavement connected to the subgrade, the bridge-span structure can allow airflow to pass through, and convection heat transfer occurs, resulting in differences in temperature change characteristics [26]. In addition, the long-span rigid-frame bridges commonly used in expressway have a thermal conductivity much larger than that of concrete, and a specific heat capacity smaller than that of concrete. Different structural forms and materials lead to diverse temperature variation characteristics of bridge deck and pavement. Under normal circumstances, the temperature of the bridge deck is about 2–3°C lower than that of the pavement near the ordinary road section, while in special weather (strong wind, snowfall or cold wave), the difference is more obvious. In some areas, the temperature difference can even reach about 7–8°C [27]. Therefore, the prediction of bridge pavement temperature is of great significance to the prediction and maintenance of road icing in winter.

With the acceleration of expressway construction in the mountainous or remote areas of western China, it is inconvenient to obtain such a large quantity of data due to terrain and economic cost issues. When a few critical data is achieved, accurate and rapid prediction of bridge pavement temperature could meet certain accuracy requirements. Therefore, it is essential to carry out small-sample, fast and accurate prediction to ensure the winter expressway driving safety in mountainous or remote areas.

Hence, minute-by-minute observed data from a small traffic meteorological monitoring station of the Tuhai River Bridge on the Beijing-Shanghai Expressway were used, including the bridge pavement temperature, dew point temperature, wind chill temperature, wet bulb temperature, relative humidity, air temperature and wind speed. The Pearson partial correlation analysis was adopted to analyze the correlation of variables, and the classification indexes and standards of meteorological data were proposed to determine the effective samples for bridge pavement temperature prediction. The multiple linear regression model of bridge pavement temperature and BP neural network prediction model based on nonlinear algorithm were established respectively, and the fitting degree of the two models was analyzed by using the observation value of bridge pavement temperature. The two main advantages of this study are as follows. First, the linear regression model and BP neural network regression model with a nonlinear algorithm are established respectively, and the issue of whether the variables are linearly correlated is fully considered. Second, with a handful of key data, accurate and fast prediction of bridge pavement temperature could meet certain road icing prediction accuracy requirements for expressways in mountainous or remote areas.

The rest of this paper is organized as follows. The section 2 is the method, including data collection and analysis, construction of the bridge pavement temperature prediction model based on multiple linear regression, and construction of the bridge pavement temperature prediction model based on BP neural network. The analysis of prediction results based on BP neural network, and the comparison between linear regression and BP neural network prediction model are presented in section 3. The conclusion and discussion is drawn at section 4.

## 2. Method

To predict the critical meteorological conditions of road icing, the minute by minute measured data of traffic meteorological stations installed on expressway bridges was collected and analyzed. The meteorological data classification index and standard were proposed, and the effective samples for bridge pavement temperature prediction were determined. Moreover, to improve the prediction accuracy, multiple linear regression model and BP neural network regression model based on nonlinear function were established respectively.

### 2.1. Data collection and analysis

#### (1) Data collection

The measured data of a small traffic weather monitoring station installed on the Tuhai River Bridge on the Beijing-Shanghai Expressway was adopted. The Beijing-Shanghai Expressway is an important part of the national expressway network and a significant passageway connecting capital Beijing and metropolis Shanghai. The Tuhai River Bridge is a continuous rigid-frame bridge, which is often used in the construction of large-span bridges. The meteorological station is located in Shandong Province (N37.05°, E117.05°), with a temperate continental climate and severe winter. Therefore, this meteorological station could be a typical observation station for icing on bridges.

According to the definition of road icing [5], the bridge pavement temperature less than 0°C with precipitation is considered as the criterion for judging the icing of highway bridge deck in winter. The site meteorological data includes bridge pavement temperature, dew point temperature, wind chill temperature, wet bulb temperature, relative humidity, air temperature and wind speed. Select the meteorological observation data in winter (December, January, and February) in the past three years (2020–2022) since the establishment of the meteorological observatory, some of which are shown in Table 1.

**Table 1:** Meteorological data of Tuhai River Bridge.

Time	Temperature(°C)					Relative humidity (%)	Wind speed (m/s)
	Dew point	Wind chill	Wet bulb	Air	Bridge deck		
09:32:00	-5.1	-5.94	-4.74	-4.57	0.32	96.13	0.96
09:31:00	-5.09	-6.23	-4.74	-4.57	0.3	96.13	1.06
09:30:00	-5.16	-6.32	-4.81	-4.64	0.25	96.16	1.08
09:29:00	-5.16	-6.42	-4.81	-4.63	0.2	96.08	1.12
09:28:00	-5.24	-6.56	-4.88	-4.71	0.14	96.03	1.17
09:27:00	-5.23	-6.58	-4.88	-4.71	0.08	96.09	1.17
09:26:00	-5.32	-6.62	-4.96	-4.78	0.03	96.06	1.18
09:24:00	-5.31	-6.7	-4.96	-4.78	-0.02	96.09	1.23
09:23:00	-5.41	-6.65	-5.04	-4.86	-0.06	95.98	1.21
09:22:00	-5.4	-6.46	-5.04	-4.86	-0.09	96.04	1.13
09:21:00	-5.4	-6.45	-5.04	-4.87	-0.1	96.03	1.13
09:20:00	-5.33	-6.45	-4.96	-4.79	-0.14	95.98	1.13
09:18:00	-5.33	-6.2	-4.96	-4.79	-0.18	96.0	1.01
09:17:00	-5.33	-6.19	-4.96	-4.79	-0.2	95.98	1.0
09:16:00	-5.29	-6.17	-4.92	-4.74	-0.24	95.99	0.98

**Fig 1:**
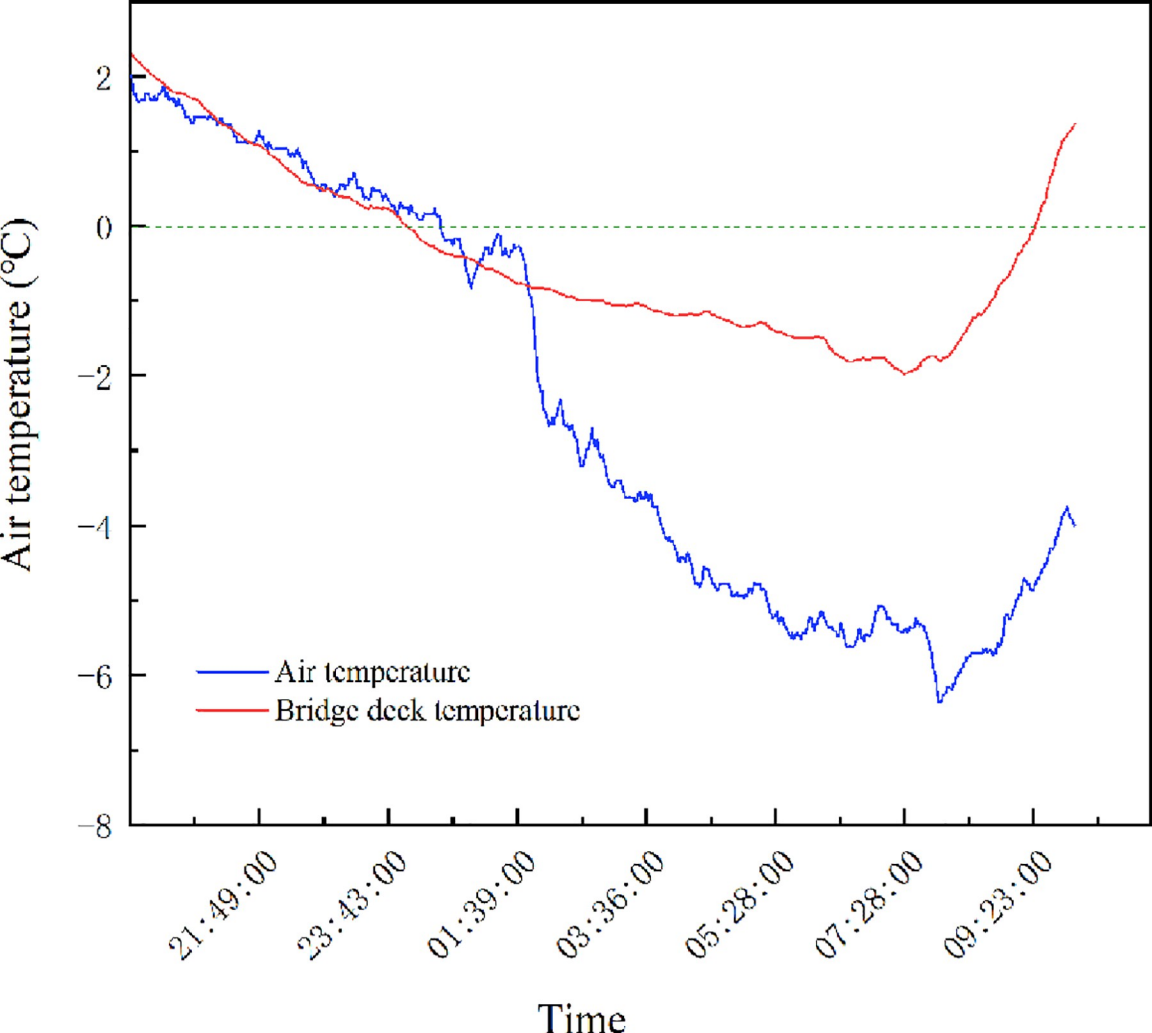
Variation law of bridge deck and air temperatures.

Fig 1 shows the variation law of bridge deck and air temperatures. At night, the temperature first drops and then rises. The reason is that the combined action of solar radiation and heat transfer, the temperature difference causes the bridge deck energy transfer, resulting in an increase in the temperature of the bridge deck during the daytime and a decrease at night. The approximate time period when the bridge pavement temperature is below 0°C is from 12:00 pm to 9:00 am. The lowest temperature on the bridge deck occurs in the morning, and the deck temperature decreases more slowly than the air temperature.

The minute-by-minute meteorological data are relatively consistent in a short period of time, as shown in Table 1. The road adhesion coefficient decreases rapidly as the bridge deck begins to freeze, while the adjacent road sections have not yet formed ice. When the vehicle enters the bridge road section, the sudden change of the adhesion coefficient seriously affects the driving safety. Therefore, the change point of the bridge deck icing state is extremely important, whereas stable meteorological factors cannot change the icing state. For example, after the bridge deck freezes and other meteorological factors remain unchanged, as the bridge pavement temperature drops, the bridge section is still in a frozen state. The significant change of meteorological factors may change the icing state of the bridge deck, which is meaningful for the bridge pavement temperature and icing prediction. To select an effective sample for bridge pavement temperature prediction, it is necessary to classify meteorological data levels to meet the requirements of significant changes.

#### (2) Selection of meteorological data grading indicators

To select effective samples, accurately predict the temperature of the bridge deck, and judge whether the bridge section is icing or not, it is necessary to find out the factors that affect the temperature of the bridge deck in the first step. Literature analysis shows that the meteorological factors that affect the bridge pavement temperature include multiple continuous variables such as temperature, humidity, and wind speed, and the dependent variable has only one continuous variable, which is the bridge pavement temperature. Multiple linear regression analysis can be used to screen significant influencing factors, which can not only analyze the interaction effect between independent variables, but also predict the dependent variable.

Due to the bridge pavement temperature affected by various factors, the Pearson partial correlation analysis method is used to analyze the correlation between the variables. The meteorological factors are stable in a short period of time, and there are abnormal data of the bridge pavement temperature caused by factors such as external environment and vehicles, the data at 10-minute intervals in three winters were selected. There are 11837 groups of valid data after removing the missing data recorded by the equipment. The detailed data are described in S1 Text.

Table 2 shows the Pearson partial correlation coefficient among variables. The closer the correlation coefficient is to 1, the higher the correlation between the two variables. The correlation between bridge pavement temperature and air temperature is the highest; air temperature is highly correlated with wind chill temperature and wet bulb temperature. The dew point temperature is determined by the air temperature and relative humidity with constant air pressure. Therefore, to screen effective samples comprehensively and quickly, air temperature, relative humidity and wind speed were selected as the indicators for the classification of sample grades when predicting the bridge pavement temperature.

**Table 2:** Pearson partial correlation analysis results.

Parameter	Temperature						
	Bridge deck	Dew point	Wind chill	Wet bulb	Air	Relative humidity	Wind speed
bridge pavement temperature	1.000	0.481	0.831	0.824	0.839	-0.178	0.300
Dew point temperature	0.481	1.000	0.639	0.825	0.573	0.683	-0.103
Wind chill temperature	0.831	0.639	1.000	0.936	0.923	-0.082	-0.058
Wet bulb temperature	0.824	0.825	0.936	1.000	0.895	0.176	0.073
Relative humidity	-0.178	0.683	-0.082	0.176	-0.135	1.000	-0.230
Air temperature	0.839	0.573	0.923	0.895	1.000	-0.135	0.148
Wind speed	0.300	-0.103	-0.058	0.073	0.148	-0.230	1.000

#### (3) Criteria for valid sample classification

Since significant changes in meteorological factors are meaningful for bridge pavement temperature and icing prediction, it is necessary to classify meteorological data according to the above-mentioned sample grading index. The above 11837 sets of data are preprocessed, and the results show that the bridge deck begins to freeze when the air temperature is below 5°C, and the bridge deck freezes stable below -10°C; According to the wind scale specification (GB/T 28591–2012) [28], the wind range is 0 to 6, and the relative humidity is 0 to 100%. A sample level division is proposed, as shown in Table 3. There are 3906 sample classification standards, and 1378 groups of valid samples are screened according to this classification standard.

**Table 3:** Classification criteria for valid sample grades.

Independent variable	Classification	Number of levels
Air temperature	-10°C–5°C, increasing by 0.5°C	31
Relative humidity	15% and below; 15%–100%, increasing by 5%	18
Wind scale	Level 0–6, increasing step by step	7

### 2.2. Construction of bridge pavement temperature prediction model based on multiple linear regression

When using multiple linear regression analysis methods, it is necessary to test for collinearity problems. The closer the partial correlation coefficient between the independent variables is to 1, it is considered that there is a collinearity problem between the two independent variables, which affects the accuracy of the regression model. To avoid the influence of collinearity problem on the fitting results of regression analysis, these factors should be excluded.

Pearson partial correlation analysis method was used to analyze 1378 groups of valid samples. It can be seen from Table 4 that the partial correlation coefficients of air temperature, wind chill temperature and wet bulb temperature in the independent variables are close to 1, as are the relative humidity and dew point temperature, indicating that the collinearity trend is obvious. Therefore, dew point temperature, wind chill temperature and wet bulb temperature are excluded, and air temperature, relative humidity and wind speed are introduced into the highway bridge pavement temperature prediction model.

**Table 4:** Pearson partial correlation analysis results.

Parameter	Temperature						
	Bridge deck	Dew point	Wind chill	Wet bulb	Air	Relative humidity	Wind speed
bridge pavement temperature	1.000	0.381	0.649	0.730	0.753	0.019	0.300
Dew point temperature	0.381	1.000	0.611	0.828	0.548	0.856	-0.152
Wind chill temperature	0.649	0.611	1.000	0.884	0.852	0.190	-0.326
Wet bulb temperature	0.730	0.828	0.884	1.000	0.867	0.456	-0.020
Relative humidity	0.019	0.856	0.190	0.456	0.112	1.000	-0.195
Air temperature	0.753	0.548	0.852	0.867	1.000	0.112	0.048
Wind speed	0.300	-0.152	-0.326	-0.020	0.048	-0.195	1.000

At the 95% confidence interval level, with air temperature, relative humidity and wind speed as independent variables, a multiple linear regression-based prediction model for highway bridge pavement temperature is established, as shown below:

$$T=0.983X_{1}‐0.003X_{2}+0.564X_{3}-0.299$$ (1)

Where, *T*, *X*_*1*,_ *X*_*2*_, and *X*_*3*_ represent the bridge pavement temperature (°C), air temperature (°C), relative humidity (%), wind speed (m/s) respectively.

The model fitting test results show that the coefficient of determination R^**2**^ is 0.635, indicating that 63.5% of the bridge pavement temperature can be explained by air temperature and wind speed. The F-test value is 801.053, and the significance probability (P) value is less than 0.05, which reveals the model parameters could be used to estimate the whole sample, and the significance test of the model is passed.

The above is the bridge pavement temperature prediction model based on the linear relationship between air temperature, wind speed and bridge pavement temperature. In fact, the relationship between meteorological data is complex. To improve the prediction accuracy, the widely used BP neural network is selected to predict the bridge pavement temperature based on a nonlinear algorithm.

### 2.3. Construction of bridge pavement temperature prediction model based on BP neural network

**Fig 2:**
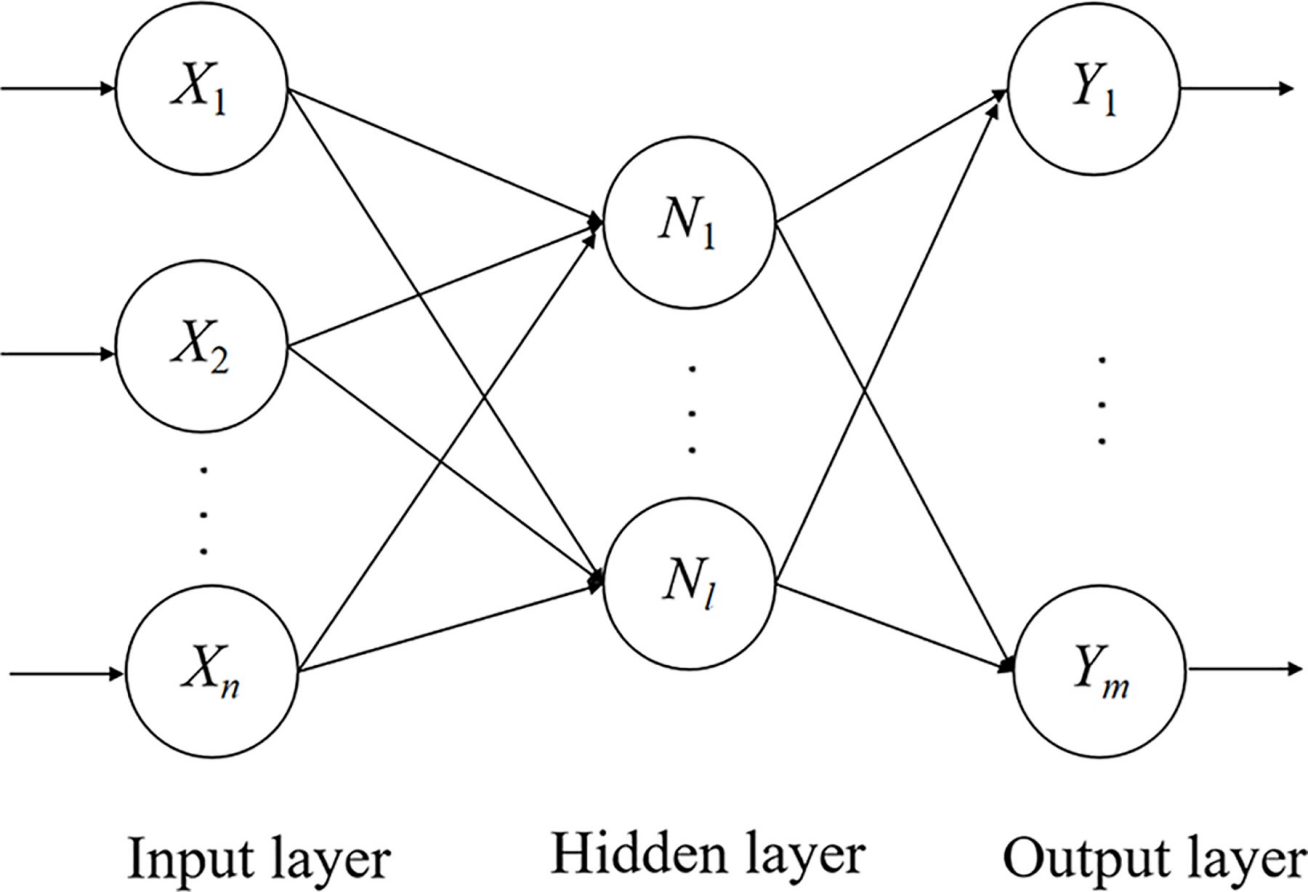
Schematic diagram of BP neural network topology.

One of the most important and valuable features of BP neural networks with nonlinear transfer functions is the ability to simulate complex nonlinear problems. A properly trained BP neural network could identify the prediction error of bridge pavement temperature in winter without knowing the internal interaction mechanism of the influencing factors, which can improve the accuracy of the prediction of bridge deck icing. The BP neural network is a multi-layer feed-forward neural network using the error back propagation algorithm, and the structure is shown in Fig 2.

BP neural network could accomplish prediction by training the network. The training process is as follows:

Step1: Network initialization. According to the original input and output data sequence (*X*, *Y*), the number of neurons in the input layer *n*, the hidden layer *l*, and the output layer *m* should be set. Then the connection weights of the input layer and the hidden layer *w*_*ij*_, the ones of the hidden layer and the output layer *w*_*jk*_ ought to be initialized, so do the hidden layer threshold *A* and the output layer threshold *B*. Moreover, the learning rate *v*, accuracy and training times need to be assigned.
Step2: Calculate the output *H* of the hidden layer. There are a great deal of choices for the hidden layer activation function of BP neural network, among which S-type function is often used, and the expression is as follows:

$$f(x)=\frac{1}{1+e^{-x}}$$ (2)

After determining the activation function, the output *H* of the hidden layer is calculated according to the output layer value, the weight of the input layer and the hidden layer *w*_*ij*_, and the hidden layer threshold *A*:

$$H_{j}=f(\overset{n}{\underset{i=1}{\sum }}w_{ij}x_{i}-A_{j}),j=1,2,\cdots ,l$$ (3)

Where, *f*, *x* represent the excitation function and the input data respectively.

Step3: Calculate the output value *O*_*k*_ of the output layer. Similar to the hidden layer output calculation principle, it is calculated based on the hidden layer output value *H*, the hidden layer and output layer weight *w*_*jk*_, and the output layer threshold *B*, as the following described:

$$O_{k}=\overset{l}{\underset{j=1}{\sum }}H_{j}w_{jk}-B_{k},k=1,2,\cdots ,m$$ (4)

Step 4: Calculation of prediction error of BP neural network. The error value is the difference between the expected value *Y* and the predicted value *O*_*k*_ of the output layer.

$$e_{k}=Y_{k}-O_{k},k=1,2,\cdots m$$ (5)

Step5: Adjust the weight according to the error, where $w_{ij}{}^{'}$ is the adjusted weight.

$$w_{ij}{}^{'}=w_{ij}+vH_{j}(1-H_{j})x(i)\overset{m}{\underset{k=1}{\sum }}w_{jk}e_{k},i=1,2,\cdots ,n;j=1,2,\cdots ,l$$ (6)

$$w_{jk}{}^{'}=w_{jk}+vH_{j}e_{k},j=1,2,\cdots ,l;k=1,2,\cdots ,m$$ (7)

**Fig 3:**
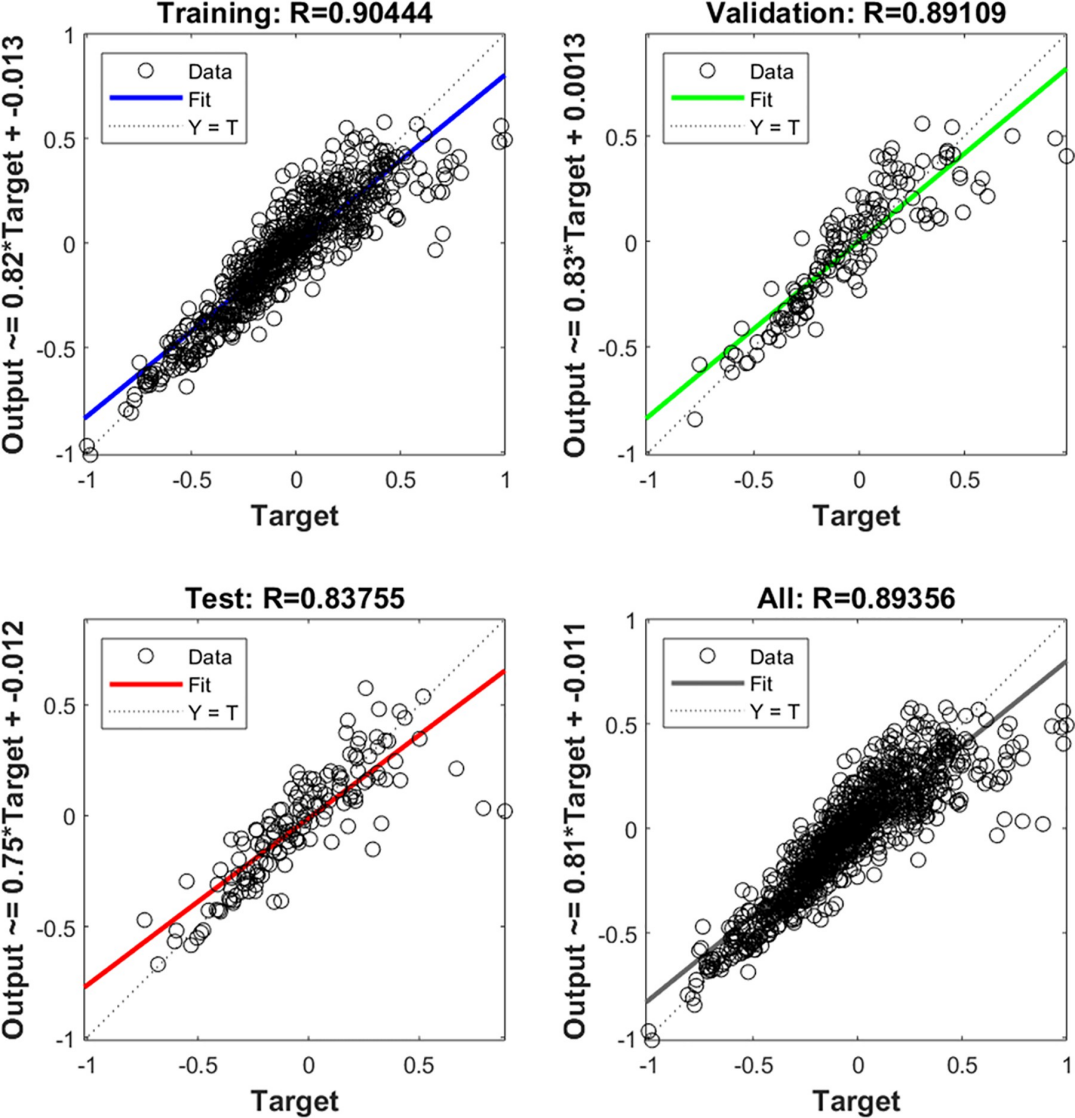
Correlation between output value and target value.

The 1378 groups of valid samples were divided into two groups, the first 1000 groups of data were randomly selected for neural network training, and the remaining 378 groups were used for neural network testing. Firstly, the training data was normalized to eliminate the large magnitude difference of input variables based on the grouped data, which could be used to train the BP neural network. To reduce the discreteness of the neural network and improve the prediction accuracy, the number of hidden layer nodes is determined to be 5, and the learning rate is adopted as 0.3 through data preprocessing. In the training process of BP neural network, the correlation of training data, verification data, test data and overall data is shown in Fig 3.

It can be seen that the regression coefficient R of the predicted value and the target value is close to 1, indicating that the BP neural network could quantify the relationship between the input and output data through training process. The trained neural network could be used to predict the temperature of expressway bridge deck in winter.

## 3. Results

### 3.1. Analysis of prediction results based on BP neural network

**Fig 4:**
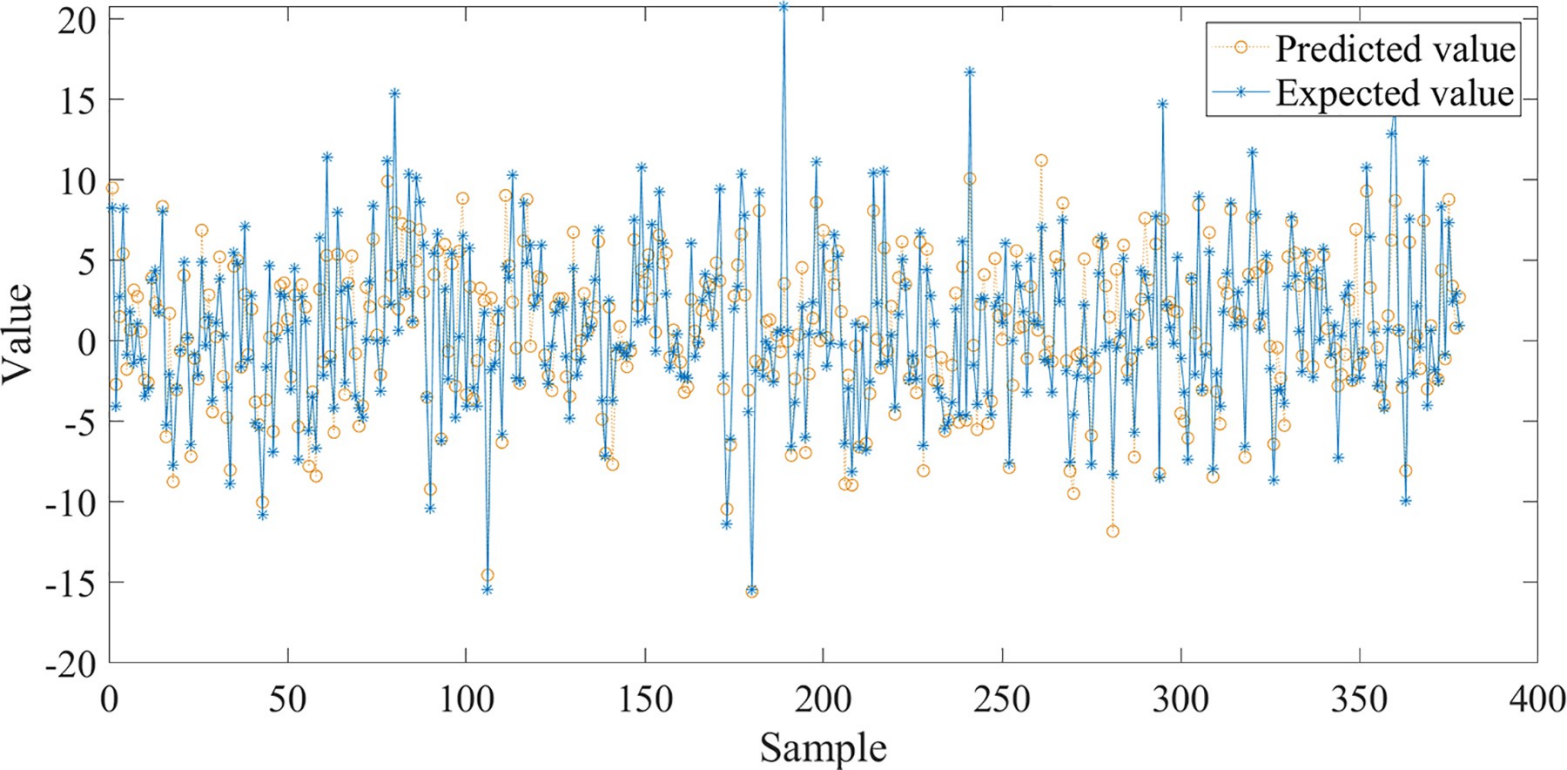
Comparison of predicted and expected value of BP neural network.

The trained BP neural network was applied to predict the test group data, and the results were shown in Fig 4. The trend of predicted and expected value is consistent, with determination coefficient R^**2**^ of 0.7929, which represents high reliability and good fitting degree, indicating that the trained BP neural network could be used to predict the bridge pavement temperature.

**Fig 5:**
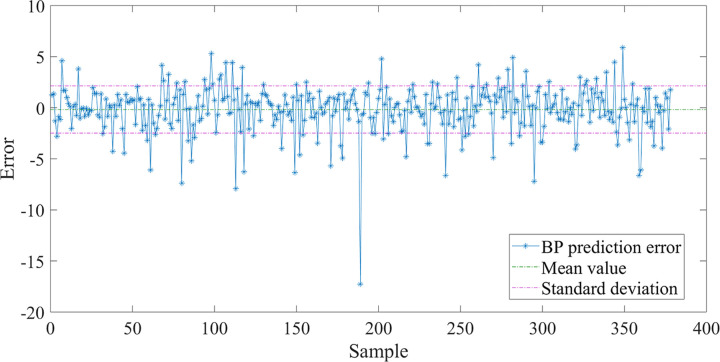
Prediction error analysis of BP neural network.

**Fig 6:**
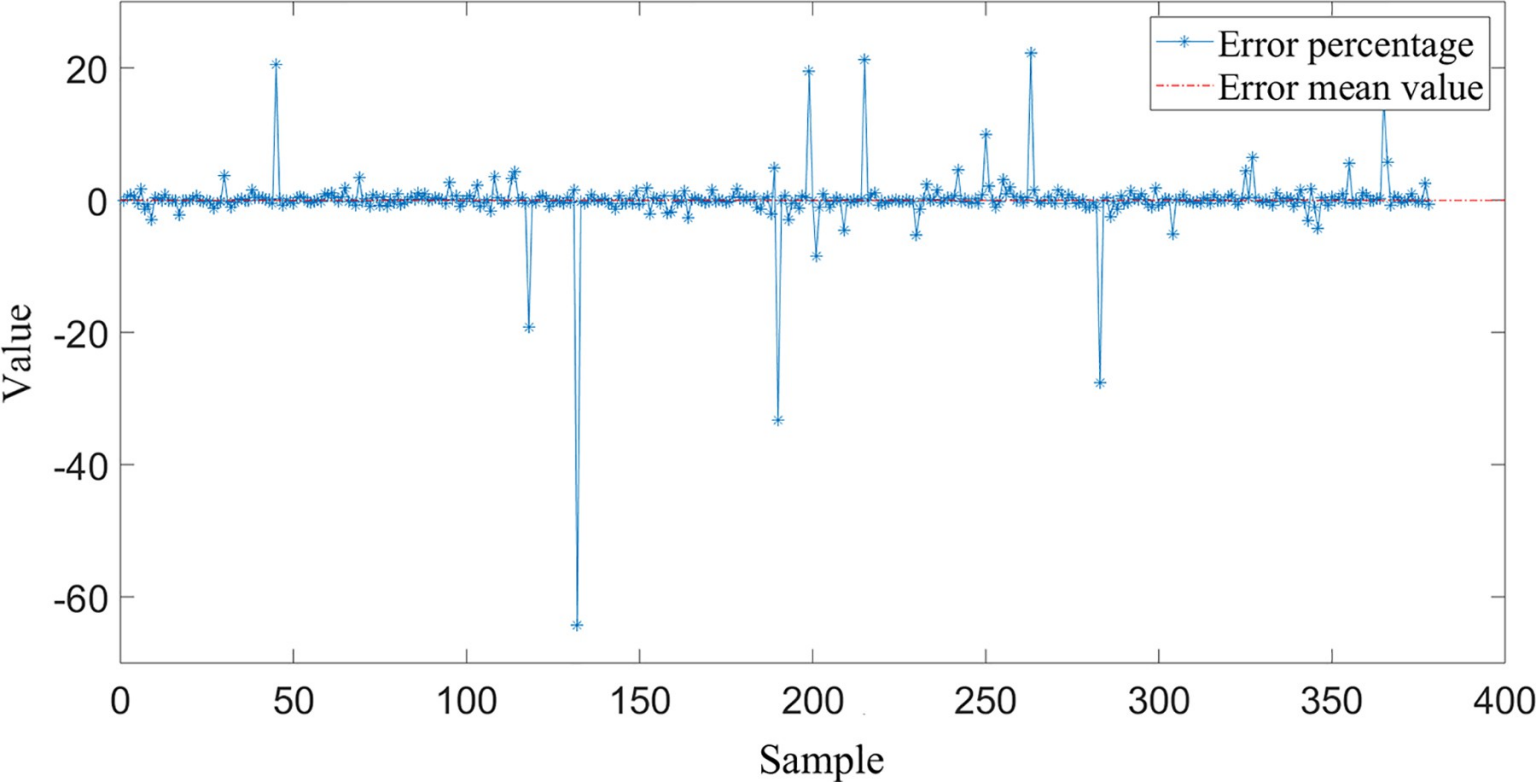
Prediction error percentage of BP neural network.

Subsequently, the prediction error of the BP neural network was analyzed. Fig 5 describes the value and the prediction error dispersion of BP neural network. The average prediction error of bridge pavement temperature is 0.1749°C, and the standard deviation is 2.313. Fig 6 illustrates the neural network prediction error percentage, the average error percentage is 0.0342, indicating that the predicted and expected values fit well. The reason for the large deviation of some samples may be that the road surface temperature sensor is interfered by factors such as vehicle operating conditions.

### 3.2. Comparison of linear regression and BP neural network prediction models

**Fig 7:**
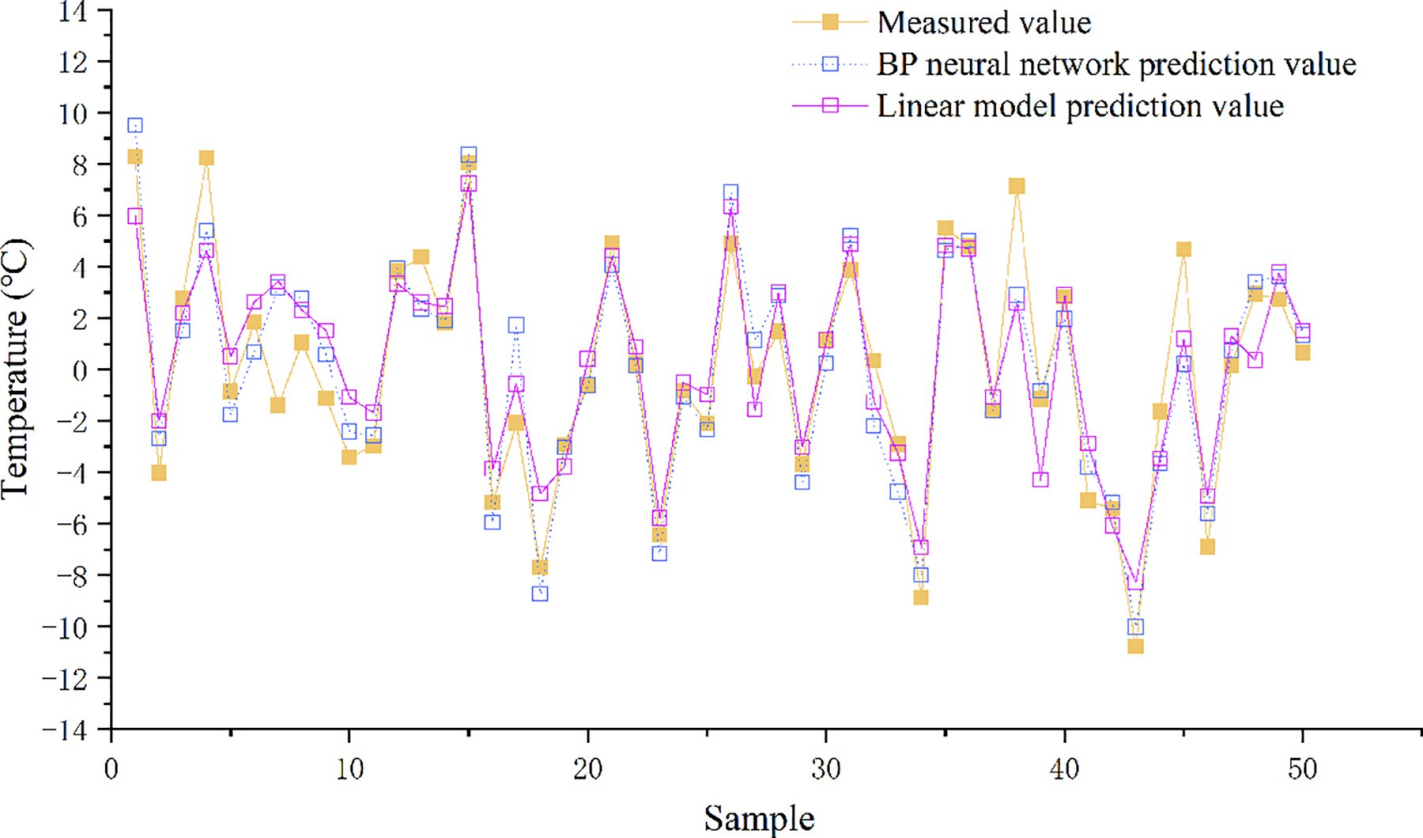
Comparison of predicted and measured values of different models.

Due to the unclear interaction of the factors that affect the bridge pavement temperature, a linear regression and a BP neural network with nonlinear function prediction model were established respectively. The predicted values were compared with the measured values, as shown in Fig 7, the BP neural network are better fitted to the true values than the linear regression model.

To further analyze the prediction accuracy of the linear and BP neural network models, deviation between predicted and true value was quantified, and the root mean square error of the two models was calculated. The root mean square error of the linear model is 3.065, and that of the BP neural network model is 2.317, indicating that the former has a large deviation from the true value. In addition, the coefficient of determination of linear regression is 0.635, and that of BP neural network is 0.7929, which also shows that the predicted value of BP neural network fits better with the true data. Therefore, the BP neural network method could be selected to predict the temperature of the expressway bridge deck in winter.

## 4. Conclusion and discussion

To predict critical meteorological conditions of expressway bridge deck Icing, the winter bridge pavement temperature and other meteorological data of small traffic meteorological monitoring stations in expressway bridge sections in recent three years were adopted, and the expressway bridge pavement temperature prediction models based on multiple linear regression and BP neural network were established respectively. Different from the existing bridge pavement temperature prediction models, which require large amounts of data and complex calculation, the models proposed could quickly predict the bridge pavement temperature through small sample observation data, and the predicted value can meet certain accuracy requirements, which makes up for the lack of measured data of expressways in remote areas of China.

Determining effective samples is essential to ensuring the rationality and accuracy of the bridge pavement temperature prediction model. When the bridge deck begins to freeze, the adhesion coefficient decreases sharply, which seriously affects the driving safety. Only significant changes in meteorological factors could change the icing state of the bridge deck. Therefore, meteorological data grading indicators and valid sample determination criteria were proposed to screen out valid samples for bridge pavement temperature prediction.

Since the influencing factors are all continuous variables, the multiple linear regression method was selected to carry out Pearson partial correlation analysis, which avoids the influence of the collinearity among variables on the accuracy of the regression prediction model. A linear regression prediction model of bridge pavement temperature based on air temperature, relative humidity and wind speed was established, under the premise that the variables are linear. In fact, the correlation between meteorological factors is not yet clear. The widely used BP neural network was used to establish a nonlinear bridge pavement temperature prediction model.

An significant part of BP neural network is training. The number of hidden layer nodes and the learning rate were proposed through data preprocessing, which ensures the optimal discreteness and accuracy. The fit of training data, verification data, test data and overall data was also analyzed, and the results show that the correlation coefficient between the predicted measured values is close to 1, which guarantees the feasibility of training network.

According to the measured values, the prediction results of linear regression model and BP neural network model were compared and analyzed. The root mean square errors of linear regression and BP neural network are 3.065 and 2.317 respectively, indicating that the deviation between the predicted value of linear regression model and the real is greater. In addition, the coefficients of determination of linear regression and BP neural network are 0.635 and 0.7929, respectively, which also means the BP neural network has a higher degree of fitting, and it is confirmed that a few key observation data (bridge pavement temperature, dew point temperature, wind chill temperature, wet bulb temperature, relative humidity, air temperature and wind speed) could meet certain accuracy requirements.

At present, numerous expressway bridge sections in China do not have meteorological monitoring stations, or the meteorological observation station data is not open access, so a meteorological station in the bridge section of Beijing-shanghai expressway were adopted. Data from various meteorological stations under different climatic and topographic conditions should be added to expand the scope of application of the temperature prediction model for expressway bridge decks in winter, which will be included in the next step.

## Supporting information

S1 Text Detailed data description. (PDF)
