# Supplementary material for: Using multiple linear regression and BP neural network to predict critical meteorological conditions of expressway bridge pavement icing
Source: PLoS One. 2022 Feb 4;17(2):e0263539. doi: 10.1371/journal.pone.0263539 (PMC8815869; doi:10.1371/journal.pone.0263539)
Supplement: S1 Text — (PDF) [file pone.0263539.s001.pdf]

All the raw data used in the manuscript are described as the follows:

temperature (°C)						
Bridge deck	Dew point	Wind chill	Wet bulb	Air	Relative humidity (%)	Wind speed （m/s）
-16.88	-30.08	-23.29	-19.56	-18.35	35.02	1.76
-16.78	-31.3	-23.65	-19.63	-18.34	31.16	1.95
-16.85	-30.49	-22.97	-19.56	-18.32	33.6	1.66
-16.7	-31.33	-23.77	-19.54	-18.23	30.78	2.05
-16.89	-29.33	-23.76	-19.38	-18.19	37.06	1.98
-16.83	-30.23	-23.6	-19.43	-18.18	34.04	1.98
-16.82	-29.66	-22.73	-19.39	-18.18	35.91	1.54
-16.7	-30.89	-23.67	-19.46	-18.18	31.95	2.01
-16.88	-29.76	-23.86	-19.39	-18.17	35.55	2.1
-16.67	-30.75	-23.95	-19.45	-18.17	32.37	2.15
-16.64	-30.4	-23.15	-19.38	-18.11	33.29	1.81
-16.6	-29.99	-22.61	-19.36	-18.11	34.61	1.6
-16.57	-29.77	-23.62	-19.26	-18.02	35.07	2.03
-15.99	-34.66	-23.69	-19.34	-17.81	21.51	2.3
14.78	-5.29	11.82	4.91	-17.75	29.86	1.35
-15.92	-34.7	-21.88	-19.26	-17.72	21.25	1.51
10.23	-10.1	12.23	4.02	-17.68	20.32	1.29
-15.74	-34.82	-21.96	-19.19	-17.63	20.86	1.61
11.29	-9.15	13.41	4.68	-17.61	20.55	1.5
-15.78	-34.94	-21.76	-19.18	-17.36	20.57	1.46
17.94	-11.86	9.33	2.25	-17.02	21.15	0.98
-15.07	-34.11	-18.68	-18.6	-16.97	21.15	0.73
15.41	-4.84	12.5	5.31	-16.96	29.79	1.34
-14.41	-34.81	-25.03	-18.62	-16.96	19.73	3.93
8.4	-14.51	5.95	-0.07	-16.95	21.21	0.92
-14.97	-34.19	-19.51	-18.74	-16.93	21.28	0.97
-14.23	-34.8	-24.12	-18.56	-16.89	19.62	3.33
12.13	-5.95	11.39	4.38	-16.88	29.78	0.75
-15	-31.79	-24.39	-18.41	-16.88	26.3	3.29
8.68	-14.63	5.4	-0.24	-16.85	21.37	1.18
-14.11	-34.89	-25.25	-18.44	-16.74	19.22	4.39
-13.73	-34.44	-24.45	-18.21	-16.5	19.68	3.96
-13.86	-33.84	-21.98	-18.14	-16.44	20.79	2.2
-13.68	-30.56	-24.53	-17.93	-16.39	28.37	3.78
-13.08	-31.65	-24.66	-17.94	-16.33	25.46	4.14
-13.63	-34.67	-23.8	-18.06	-16.3	18.94	3.76
-12.12	-31.63	-25.07	-17.75	-16.1	25.02	4.8

-13.5	-34.72	-24.96	-17.86	-16.07	18.46	5.04
-13.23	-34.79	-24.85	-17.82	-16.01	18.27	4.96
-13.32	-34.57	-23.85	-17.75	-15.94	18.56	3.9
-13.42	-34.73	-25.21	-17.75	-15.93	18.24	5.44
-13.39	-34.75	-24.85	-17.73	-15.91	18.19	4.93
-11.12	-31.11	-24.31	-17.47	-15.81	25.66	4.63
-13.06	-35.33	-26.09	-17.64	-15.77	16.98	6.88
-10.79	-31.11	-24.74	-17.4	-15.72	25.47	5.12
-12.93	-35.31	-26.1	-17.59	-15.71	16.92	7.35
-12.77	-34.67	-24.38	-17.31	-15.41	17.59	5.58
-9.59	-31.89	-24.82	-17.16	-15.38	23	5.67
-9.27	-31.67	-24.53	-16.97	-15.16	23.05	5.51
-12.39	-34.16	-21.76	-16.73	-14.73	17.49	3.8
-7.33	-31.65	-23.03	-16.41	-14.48	21.86	4.96
-12.27	-34.47	-24.15	-16.53	-14.47	16.61	6.5
-12.18	-34.74	-24.87	-16.48	-14.39	16.06	7.85
-12.08	-34.5	-24.53	-16.41	-14.32	16.36	7.55
-11.99	-32.77	-22.52	-16.22	-14.18	19.14	5.02
-6.54	-31.42	-22.19	-16.09	-14.11	21.66	4.72
-6.96	-30.44	-23.03	-16.03	-14.11	23.77	5.16
-11.55	-32.67	-21.99	-15.84	-13.73	18.62	4.88
-11.37	-33.09	-22.75	-15.73	-13.56	17.64	6.26
-11.27	-33.33	-22.92	-15.62	-13.42	17.03	6.78
-11.16	-33.27	-23.05	-15.51	-13.29	16.95	7.2
-11.06	-33.06	-23.06	-15.41	-13.19	17.17	7.5
-4.56	-30.64	-22.53	-15.28	-13.17	21.63	6.07
-13.55	-21.81	-18.87	-14.46	-13.04	47.83	2.84
-10.93	-32.89	-22.73	-15.29	-13.04	17.24	7.24
-12.06	-23.79	-17.93	-14.61	-12.98	39.97	2.23
-12.02	-23.67	-17.3	-14.53	-12.9	40.13	1.86
-12.16	-25.75	-16.54	-14.72	-12.9	33.27	1.55
-13.67	-22.04	-19.02	-14.36	-12.89	46.29	3.03
-12.19	-25.77	-16.76	-14.72	-12.89	33.21	1.63
-13.76	-22.1	-18.99	-14.35	-12.87	45.99	3.08
-12.16	-25.28	-17.18	-14.66	-12.87	34.65	1.83
-12.17	-25.11	-17.74	-14.63	-12.85	35.14	2.15
2.21	-12.88	2.21	-1.7	-12.84	30.08	1.16
4.67	-13.32	2.01	-2.07	-12.84	30.09	1.03
-13.84	-22.12	-19.52	-14.32	-12.82	45.72	3.52
-12.12	-24.38	-18.14	-14.52	-12.8	37.34	2.43
-13.91	-22.22	-19.42	-14.29	-12.78	45.17	3.53
-13.44	-21.36	-17.92	-14.19	-12.77	48.65	2.25
-10.66	-32.36	-21.28	-15.03	-12.75	17.73	5.59
-14.02	-22.27	-18.38	-14.24	-12.7	44.69	2.71

-8.62	-24.88	-13.35	-12.87	-12.69	30.12	1.27
-10.54	-31.9	-20.57	-14.95	-12.68	18.44	4.96
-13.98	-23.07	-18.33	-14.27	-12.64	41.43	2.8
20.3	3.09	22.66	11.84	-12.63	30.12	1.26
-13.93	-22.18	-18.93	-14.17	-12.63	44.78	3.2
-11.99	-24.8	-15.76	-14.42	-12.62	35.48	1.34
-14	-21.95	-17.7	-14.1	-12.57	45.49	2.32
21.15	3.25	23.04	11.97	-12.55	30.14	1.2
-11.41	-24.78	-15.76	-14.34	-12.53	35.27	1.37
2.71	-13.18	2.48	-1.97	-12.52	30.16	0.79
-1.33	-16.06	-1.83	-4.59	-12.51	30.19	1.16
-11.8	-22.91	-16.74	-14.14	-12.51	41.59	1.76
-11.72	-23.07	-16.49	-14.15	-12.5	40.97	1.74
-11.15	-24.62	-16.96	-14.31	-12.5	35.7	1.94
3.97	-10.94	4.42	0	-12.49	30.2	1.36
3.23	-13.16	2.14	-1.97	-12.46	30.22	0.93
-11.25	-24.3	-15.42	-14.24	-12.46	36.59	1.31
2.98	-13.14	1.96	-1.97	-12.45	30.28	1.05
8.78	-6.06	10.54	4.16	-12.45	30.36	1.3
-10.43	-32.2	-21.22	-14.78	-12.45	17.57	6.12
-11.06	-24.3	-16.03	-14.23	-12.45	36.56	1.58
4.91	-13.2	2.01	-2.05	-12.41	30.36	1.01
-13.87	-23.1	-18.12	-14.09	-12.41	40.57	2.8
-13.92	-23.09	-18.31	-14.09	-12.4	40.57	2.9
-3.34	-29.92	-21.29	-14.61	-12.39	21.72	5.83
-11.6	-25.5	-15.93	-14.28	-12.37	32.64	1.53
-11.19	-24.4	-15.85	-14.17	-12.36	35.98	1.43
-11.01	-24.38	-16.12	-14.15	-12.34	35.98	1.67
-13.85	-23.18	-17.41	-14.04	-12.33	40.04	2.42
3.85	-10.93	4.31	-0.05	-12.3	30.37	1.31
-3.03	-30.03	-21.15	-14.52	-12.27	21.29	5.89
-13.53	-23.5	-15.39	-13.98	-12.22	38.56	1.35
-13.71	-23.42	-15.41	-13.98	-12.22	38.84	1.39
-13.23	-20.89	-17.44	-13.69	-12.22	48.5	2.25
-10.28	-31.79	-20.88	-14.58	-12.22	17.94	6.04
-13.58	-23.68	-15.61	-13.99	-12.21	37.94	1.48
-13.83	-23.13	-16.27	-13.94	-12.21	39.81	1.77
-10.89	-24.18	-17.03	-14.03	-12.21	36.28	2.32
-13.83	-23.17	-17.04	-13.93	-12.2	39.64	2.17
-10.96	-24.46	-16.78	-14.04	-12.2	35.34	2.02
-13.85	-23.19	-17.34	-13.93	-12.19	39.55	2.41
-13.81	-23.3	-16.25	-13.94	-12.19	39.16	1.8
-13.46	-23.43	-17.48	-13.94	-12.18	38.68	2.61
-13.84	-23.11	-16.66	-13.9	-12.16	39.73	1.99

-13.76	-23.38	-15.82	-13.89	-12.12	38.66	1.57
-10.54	-23.95	-17.17	-13.94	-12.12	36.75	2.52
-10.58	-23.97	-16.86	-13.93	-12.11	36.64	2.18
-13.69	-23.53	-15.87	-13.87	-12.07	37.98	1.66
-10.62	-24.16	-16.62	-13.89	-12.04	35.85	2.01
-13.7	-23.43	-13.84	-13.82	-12.02	38.19	0.9
-13.66	-23.52	-15.19	-13.8	-11.99	37.8	1.34
-10.11	-31.5	-20.37	-14.38	-11.99	18.11	5.66
-10.66	-24.27	-16.28	-13.85	-11.97	35.29	1.85
-13.32	-23.39	-19.24	-13.74	-11.93	38.03	4.3
-10.74	-24.5	-15.78	-13.82	-11.91	34.4	1.69
-13.31	-23.84	-18.93	-13.75	-11.88	36.41	4.1
-13.04	-24.15	-18.15	-13.77	-11.88	35.41	3.39
-13.19	-23.78	-18.68	-13.73	-11.87	36.56	3.85
-13.1	-23.81	-18.25	-13.73	-11.87	36.47	3.4
-10.29	-24.45	-16.59	-13.79	-11.87	34.47	2.18
-13.3	-23.55	-19.77	-13.69	-11.85	37.25	4.93
-9.93	-31.86	-21.21	-14.26	-11.83	17.27	7.33
-10.24	-24.48	-16.57	-13.75	-11.82	34.24	2.28
-13.28	-23.87	-18.68	-13.67	-11.79	36.03	3.88
-13.12	-23.92	-18.63	-13.68	-11.79	35.88	3.82
-13.22	-23.87	-18.86	-13.67	-11.78	36.03	4.08
-13.18	-23.51	-18.97	-13.62	-11.76	37.14	4.1
-10.07	-23.86	-15.77	-13.64	-11.75	35.97	1.78
-10.02	-23.64	-16.42	-13.61	-11.75	36.66	2.11
-10.12	-24.09	-16.66	-13.65	-11.74	35.23	2.3
-12.95	-24.21	-18.01	-13.66	-11.73	34.8	3.4
-9.58	-23.72	-16.49	-13.55	-11.67	36.19	2.3
-12.83	-24.26	-17.78	-13.61	-11.66	34.44	3.31
-9.75	-31.85	-20.78	-14.1	-11.62	16.99	6.94
-9.83	-23.7	-15.52	-13.51	-11.62	36.11	1.74
-10.18	-24.28	-15.61	-13.56	-11.61	34.27	1.78
-9.8	-23.79	-15.73	-13.52	-11.61	35.79	1.86
-9.51	-23.5	-16	-13.48	-11.61	36.71	2.06
-10.15	-24.13	-16.4	-13.53	-11.59	34.67	2.17
-9.44	-23.52	-16.02	-13.43	-11.53	36.43	2.09
-9.6	-23.92	-16.49	-13.46	-11.52	35.12	2.3
-9.4	-23.57	-16.08	-13.42	-11.51	36.21	2.15
-12.76	-24.56	-18.23	-13.51	-11.5	33.13	3.89
-9.55	-32.04	-21.23	-14	-11.49	16.53	8.16
-9.69	-23.95	-15.51	-13.41	-11.45	34.84	1.77
-9.31	-23.76	-16.01	-13.38	-11.45	35.41	2.19
-12.69	-24.68	-18.62	-13.43	-11.39	32.48	4.53
-9.06	-23.11	-14.77	-13.25	-11.36	37.26	1.42

-9.27	-23.91	-15.97	-13.28	-11.3	34.54	2.2
-12.57	-25.34	-17.37	-13.41	-11.28	30.34	3.53
-9.33	-31.84	-20.76	-13.8	-11.24	16.51	7.93
-12.48	-20.4	-16.46	-12.83	-11.21	46.63	2.16
-12.62	-25.11	-17.89	-13.32	-11.2	30.76	3.74
-12.67	-24.82	-18.14	-13.25	-11.14	31.43	4.2
-2.1	-29.19	-20.05	-13.5	-11.05	20.9	6.06
-9.1	-31.03	-19.67	-13.59	-11.03	17.53	6.5
-12.46	-25.17	-17.4	-13.17	-11	30.11	3.57
-9.04	-24.51	-15.85	-13.1	-11	31.97	2.39
-1.86	-29.14	-19.53	-13.45	-10.99	20.9	5.81
-12.3	-25.52	-17.86	-13.17	-10.97	29.12	4.26
-12.4	-25.43	-17.85	-13.16	-10.96	29.33	4.09
-12.32	-25.2	-18.5	-13.14	-10.96	29.94	4.85
-8.99	-24.78	-15.17	-13.05	-10.91	31	2.03
-12.45	-25.34	-17.88	-13.1	-10.89	29.39	4.15
-8.9	-31.13	-19.7	-13.47	-10.88	17.16	6.92
-8.85	-25.1	-14.33	-13.02	-10.84	29.93	1.64
-12.22	-25.2	-17.6	-12.97	-10.75	29.44	4.15
-8.72	-25.34	-14.07	-12.96	-10.73	29.03	1.56
-12.12	-26.25	-16.77	-13	-10.68	26.63	3.42
2.77	-12.84	2.32	-1.77	-10.67	30.41	1.4
-12.19	-25.17	-17.15	-12.91	-10.67	29.36	3.74
-11.99	-25.64	-17.37	-12.95	-10.67	28.13	4.01
-8.68	-30.8	-19.01	-13.29	-10.66	17.41	6.27
-8.56	-24.8	-13.98	-12.86	-10.66	30.3	1.59
-12.17	-26.02	-16.93	-12.92	-10.6	27.02	3.54
-7.11	-22.62	-14.93	-12.61	-10.6	36.65	1.85
-12.06	-24.94	-17.03	-12.83	-10.59	29.78	3.62
-1.21	-28.97	-18.47	-13.1	-10.56	20.52	5.48
-11.23	-21.07	-17.06	-12.39	-10.53	41.7	3.41
-11.9	-26.31	-17.5	-12.89	-10.52	26.14	4.37
-6.62	-21.83	-14.62	-12.41	-10.46	38.84	1.88
12.76	-6.18	11.51	4.19	-10.44	29.72	0.99
-11.86	-26.53	-17.41	-12.83	-10.43	25.45	4.48
-6.02	-23.56	-15.82	-12.57	-10.43	33.26	2.75
-8.47	-30.38	-18.41	-13.05	-10.39	17.73	5.82
-1.13	-29.12	-18.69	-12.92	-10.32	19.86	5.77
-11.79	-26.21	-16.45	-12.71	-10.31	25.96	3.55
-11.82	-26.3	-16.87	-12.71	-10.3	25.73	3.91
-3.96	-19.47	-6.6	-7.58	-10.29	29.72	1.46
15.24	-4.69	13.05	5.46	-10.29	29.72	1.13
-11.84	-26.43	-17.45	-12.71	-10.29	25.4	4.53
-11.77	-24.31	-15.35	-12.51	-10.25	30.67	2.68

13.69	-5.76	11.4	4.55	-10.21	29.71	1.29
-8.25	-30.11	-18.34	-12.9	-10.21	17.95	6.01
-11.31	-26.48	-15.99	-12.65	-10.2	25.11	3.33
-11.53	-23.44	-15.85	-12.36	-10.16	32.92	3.13
-0.92	-29.17	-18.11	-12.79	-10.14	19.49	5.78
-8.03	-29.89	-17.97	-12.83	-10.14	18.22	5.7
-7.85	-25.61	-13.49	-12.52	-10.14	27.04	1.62
-11.67	-23.67	-14.33	-12.34	-10.11	32.11	2.03
-11.62	-22.13	-14.18	-12.17	-10.1	36.77	1.95
-4.04	-19.63	-6.19	-7.7	-10.09	29.64	1.16
-11.48	-22.75	-15.39	-12.23	-10.09	34.78	2.84
-7	-26.1	-15.52	-12.46	-10.02	25.61	2.98
-6.9	-26.24	-15.47	-12.48	-10.02	25.31	3.11
-7.19	-26.2	-15.49	-12.47	-10.01	25.37	2.94
-7.1	-26.04	-15.47	-12.46	-10.01	25.75	2.93
15.29	-4.87	12.2	5.32	-10	29.63	1.2
-11.36	-23.59	-15.51	-12.25	-10	32.07	2.8
-11.42	-21.98	-14.95	-12.04	-9.95	36.79	2.59
-7.34	-26.82	-15.01	-12.47	-9.95	23.87	2.59
-11.2	-26.88	-16.1	-12.46	-9.92	23.69	3.5
-7.4	-26.56	-13.28	-12.4	-9.89	24.32	1.58
-6.81	-26.29	-15.31	-12.37	-9.87	24.88	3.06
-4.57	-23.18	-16.18	-12.09	-9.86	32.9	3.59
-11.04	-25.69	-16.21	-12.32	-9.85	26.24	3.86
13.24	-5.83	11.06	4.53	-9.84	29.55	1.23
-0.74	-29.13	-17.83	-12.53	-9.82	19.07	6.08
-7.78	-30.41	-18.38	-12.61	-9.82	16.91	6.86
-0.97	-28.35	-17.98	-12.46	-9.8	20.47	5.25
2.78	-12.02	3.77	-0.73	-9.78	29.39	1.15
-10.95	-26.5	-16.45	-12.29	-9.73	24.15	4.37
-8.29	-24.99	-13.29	-12.7	-9.72	29.3	1.38
-10.77	-23.44	-15.12	-12.01	-9.72	31.78	2.97
-4.07	-23.67	-16.05	-12.03	-9.72	31.14	3.67
-6.75	-26.34	-15.23	-12.25	-9.72	24.48	3.01
-6.66	-26.28	-15.56	-12.25	-9.72	24.6	3.41
-10.92	-25.86	-15.73	-12.22	-9.71	25.55	3.48
-7.52	-30.42	-18.32	-12.5	-9.69	16.71	7.13
-10.2	-21.64	-16.88	-11.79	-9.67	37.09	4.23
-10.64	-24.74	-15.3	-12.07	-9.65	28.12	3.31
-11.11	-26.83	-16.15	-12.21	-9.6	23.19	3.89
-10.26	-24.92	-14.56	-12.04	-9.58	27.54	2.7
-6.58	-26.36	-15.79	-12.15	-9.58	24.17	3.69
-6.48	-26.21	-15.49	-12.13	-9.57	24.5	3.45
-10.55	-17.16	-16.31	-11.11	-9.56	53.98	4.27

-3.68	-23.84	-16.53	-11.92	-9.56	30.28	4.41
-10.91	-26.51	-16.44	-12.15	-9.55	23.79	4.4
-10.4	-17.39	-16.45	-11.11	-9.51	52.73	4.48
-6.37	-26.23	-14.93	-12.08	-9.51	24.32	3.08
-10.47	-25.87	-15.05	-12.05	-9.5	25.12	3.19
-10.37	-25.77	-14.99	-12.05	-9.5	25.33	2.93
-10.64	-17.06	-15.71	-11.05	-9.5	54.16	3.86
-10.54	-17.4	-15.28	-11.11	-9.5	52.62	3.4
-10.6	-17.42	-16.11	-11.1	-9.49	52.54	4.19
-10.54	-17.61	-15.45	-11.13	-9.49	51.69	3.48
-10.52	-26.41	-16.01	-12.08	-9.48	23.88	4.34
-7.24	-30.25	-18.22	-12.33	-9.47	16.7	7.4
-10.61	-16.77	-14.81	-10.97	-9.46	55.31	2.96
-10.4	-18.47	-16.16	-11.22	-9.45	47.88	4.38
-10.63	-16.48	-15.18	-10.9	-9.42	56.52	3.39
-10.57	-16.44	-15.15	-10.89	-9.42	56.68	3.28
-10.57	-16.75	-15.81	-10.94	-9.41	55.18	3.77
-10.55	-17.24	-15.95	-11.01	-9.41	52.94	3.99
-10.38	-18.75	-15.77	-11.23	-9.41	46.66	4.02
-10.16	-19.8	-16.46	-11.37	-9.41	42.61	4.81
-9.7	-20.09	-16.15	-11.41	-9.41	41.56	4.33
-9.67	-20.07	-16.05	-11.41	-9.41	41.64	4.34
-10.23	-19.43	-16.7	-11.32	-9.4	43.97	5.05
-10.18	-19.6	-16.48	-11.34	-9.4	43.34	4.75
-10.38	-19.04	-17.04	-11.26	-9.39	45.43	5.35
-10.32	-17.28	-16.49	-10.98	-9.37	52.62	4.62
-10.63	-16.47	-15.3	-10.84	-9.34	56.18	3.48
-10.34	-19.16	-16.06	-11.23	-9.33	44.74	4.3
-10.34	-18.92	-15.89	-11.19	-9.33	45.66	4.07
-10.12	-19.97	-15.95	-11.33	-9.33	41.74	4.51
-10.24	-18.81	-16.66	-11.17	-9.32	46.06	4.95
-0.77	-28.73	-16.69	-12.11	-9.31	19.02	4.74
-10.6	-16.71	-15.68	-10.85	-9.3	54.88	3.92
-10.43	-17.22	-14.77	-10.9	-9.26	52.45	3.12
-9.64	-20.1	-15.49	-11.29	-9.26	41.04	3.82
-9.54	-20.12	-15.48	-11.29	-9.26	40.98	3.83
-9.49	-20.09	-16.02	-11.29	-9.26	41.1	4.52
-10.51	-27.05	-16.24	-11.95	-9.25	22.12	4.45
-10.38	-17.99	-16.09	-11	-9.25	49.11	4.48
-10.07	-27.57	-13.87	-11.98	-9.24	21.08	2.52
-6.9	-30.36	-17.88	-12.15	-9.23	16.21	7.3
-6.15	-26	-14.23	-11.82	-9.2	24.24	2.7
-9.5	-19.91	-15.85	-11.2	-9.18	41.48	4.22
-10.37	-18.43	-16.75	-11.01	-9.17	47.03	5.11

-0.73	-28.32	-16.53	-11.96	-9.15	19.51	4.93
-9.91	-19.08	-14.22	-11.06	-9.13	44.35	2.81
-9.85	-19.89	-14.92	-11.16	-9.12	41.32	3.46
-5.92	-26.01	-14.98	-11.76	-9.12	24.06	3.48
-9.43	-20.14	-15.56	-11.18	-9.11	40.44	4.1
-6.55	-30.27	-17.01	-12.04	-9.1	16.19	6.23
-9.82	-19.67	-15.38	-11.12	-9.1	42.06	3.89
-5.79	-26.29	-15.66	-11.77	-9.1	23.44	4.28
-1.28	-28.68	-16.97	-11.92	-9.06	18.75	5.92
-9.39	-20.29	-14.88	-11.15	-9.04	39.71	3.49
-10.08	-20.09	-15.59	-11.12	-9.03	40.36	4.4
-2.14	-24.37	-16.31	-11.55	-9.02	27.69	5.14
-10.12	-16.89	-16.08	-10.64	-8.99	52.78	4.62
-9.32	-20.27	-14.88	-11.09	-8.96	39.54	3.67
-9.92	-27.72	-13.99	-11.74	-8.91	20.25	2.92
-10.02	-20.23	-15.23	-11.02	-8.89	39.41	4.16
-6.13	-30.4	-17.25	-11.87	-8.87	15.71	7.04
-9.25	-20.34	-15.3	-11.02	-8.86	38.99	4.19
-1.45	-28.39	-15.91	-11.74	-8.85	18.94	5
-9.96	-19.89	-15.38	-10.96	-8.85	40.5	4.27
-9.24	-21.42	-14.6	-11.12	-8.82	35.39	2.98
-9.96	-20.08	-15.36	-10.95	-8.81	39.68	4.23
-9.9	-19.97	-15.75	-10.93	-8.81	40.08	4.78
-9.13	-20.26	-15.89	-10.96	-8.8	39.05	4.86
-9.04	-20.22	-15.56	-10.96	-8.8	39.2	4.53
-9.01	-20.18	-15.18	-10.95	-8.8	39.34	4.17
-1.82	-23.96	-16.07	-11.33	-8.78	28.19	5.13
-10.05	-20.31	-15.2	-10.92	-8.74	38.69	4.16
-5.14	-26.32	-15.5	-11.49	-8.74	22.7	4.61
-9.19	-20.23	-15.51	-10.9	-8.73	38.95	4.48
-8.93	-20.12	-15.51	-10.89	-8.73	39.32	4.81
-9.72	-26.75	-12.33	-11.51	-8.71	21.78	1.86
-9.36	-26.42	-12.19	-11.49	-8.71	22.47	1.76
-1.1	-23.64	-16.5	-11.25	-8.71	28.84	6.09
-9.23	-27.58	-12.91	-11.56	-8.69	20.15	2.15
-9.01	-27.74	-13.49	-11.56	-8.68	19.85	2.64
-4.35	-9.89	-11.03	-9	-8.68	90.99	1.24
-9.78	-27.71	-13.12	-11.53	-8.64	19.85	2.33
-9.55	-26.14	-11.81	-11.41	-8.64	22.92	1.6
-1.64	-23.42	-15.85	-11.17	-8.64	29.27	4.9
-9.47	-25.36	-12.23	-11.34	-8.63	24.56	1.78
-8.72	-27.89	-13.1	-11.51	-8.59	19.44	2.5
-1.3	-23.3	-16.09	-11.11	-8.57	29.39	5.48
-4.3	-9.75	-10.31	-8.88	-8.57	91.17	1.06

-8.89	-20.5	-15.51	-10.8	-8.56	37.53	4.96
-5.6	-30.55	-16.06	-11.61	-8.53	15.08	6.23
-4.29	-9.72	-10.87	-8.84	-8.52	91.02	1.17
-8.55	-28.11	-13.5	-11.42	-8.46	18.85	2.98
-9.8	-16.84	-15.63	-10.23	-8.46	50.86	4.88
-8.84	-20.3	-14.55	-10.71	-8.46	37.92	3.94
-8.74	-20.27	-14.32	-10.7	-8.46	37.98	3.7
-4.68	-26.43	-15.11	-11.29	-8.46	22.01	4.63
-8.78	-20.01	-14.58	-10.67	-8.45	38.84	3.87
-8.28	-21.66	-15.46	-10.85	-8.44	33.63	4.67
-1.78	-28.78	-14.97	-11.44	-8.43	17.67	4.57
-1.57	-27.99	-15.31	-11.37	-8.41	19.01	4.32
-8.7	-20.27	-14.12	-10.65	-8.38	37.79	3.59
-3.96	-9.51	-10.42	-8.64	-8.32	91.08	1.16
-4.13	-9.5	-9.93	-8.64	-8.32	91.16	0.99
-4.57	-25.82	-14.98	-11.12	-8.3	22.98	4.7
-8.66	-18.5	-13.27	-10.33	-8.27	43.58	2.87
-8.29	-27.94	-13.26	-11.23	-8.23	18.81	2.91
-8.44	-19.89	-14.08	-10.48	-8.23	38.56	3.7
-4.66	-30.18	-16.58	-11.36	-8.22	15.25	7.41
-8.54	-19.31	-14.1	-10.4	-8.22	40.51	3.71
-8.46	-20.23	-14.42	-10.52	-8.22	37.44	4.02
-4.49	-26.25	-15.08	-11.09	-8.21	21.94	4.99
-4.03	-9.49	-11.64	-8.54	-8.19	90.38	1.89
-9.63	-16.87	-14.71	-10.04	-8.19	49.69	4.06
-4.22	-9.33	-9.41	-8.49	-8.18	91.37	0.85
-0.46	-23.79	-15.42	-10.85	-8.17	27.31	5.16
-8.08	-27.91	-13.13	-11.17	-8.15	18.76	2.88
-8.49	-19.27	-14.4	-10.34	-8.14	40.38	4
-5.14	-29.8	-16.09	-11.27	-8.13	15.7	6.24
-8.4	-18.7	-13.64	-10.24	-8.11	42.3	3.35
-1.69	-28.09	-15.27	-11.14	-8.1	18.37	4.71
-3.35	-9.25	-11.24	-8.4	-8.08	91.32	1.6
-7.85	-27.95	-13.33	-11.12	-8.08	18.58	3.18
-0.18	-24.47	-16.08	-10.85	-8.08	25.5	6.38
-4.41	-25.3	-15.01	-10.9	-8.07	23.66	4.99
-4.19	-25.71	-15.15	-10.94	-8.07	22.79	5.21
-4.13	-9.23	-9.93	-8.38	-8.07	91.32	1.03
-4.37	-25.57	-15.49	-10.92	-8.06	23.06	5.66
-8.66	-13.62	-12.94	-9.35	-8.02	64.11	3
-8.14	-14.02	-14.97	-9.42	-8.01	61.99	5.11
-3.96	-24.92	-14.18	-10.83	-8.01	24.35	4.1
-1.94	-28.67	-14.76	-11.07	-7.96	17.22	4.39
-3.83	-24.99	-14.69	-10.8	-7.96	24.12	4.79

-3.18	-29.07	-15.25	-11.09	-7.95	16.57	5.64
-7.94	-20.76	-12.71	-10.38	-7.95	35.01	2.75
-2.87	-29.04	-15.21	-11.07	-7.93	16.6	5.75
-3.61	-29.61	-15.87	-11.11	-7.93	15.74	6.92
-4.14	-29.53	-15.95	-11.09	-7.92	15.83	6.56
-3.68	-25.51	-15.64	-10.81	-7.92	22.93	6.19
-3.4	-25.72	-15.5	-10.83	-7.92	22.51	6.07
-2.14	-28.95	-14.65	-11.04	-7.89	16.69	4.6
-7.33	-27.84	-12.57	-10.96	-7.88	18.48	2.71
0.17	-23.94	-15.61	-10.64	-7.87	26.33	5.76
-3.25	-25.93	-14.81	-10.8	-7.86	21.98	5.26
-2.58	-29.16	-13.97	-11.01	-7.83	16.28	4.3
-3.88	-28.94	-14.66	-10.98	-7.82	16.61	5.09
-8.15	-13.46	-10.9	-9.17	-7.82	63.92	1.67
-7.04	-27.87	-12.45	-10.9	-7.81	18.34	2.7
-8.09	-20.95	-11.9	-10.29	-7.8	34.06	2.23
-8.49	-13.3	-13.57	-9.11	-7.79	64.6	3.74
-8.5	-13.44	-11.76	-9.13	-7.78	63.85	2.22
-8.72	-13.38	-12.8	-9.12	-7.77	64.15	2.87
-6.81	-16.09	-10.09	-9.59	-7.76	51.27	1.27
-6.59	-15.41	-10.25	-9.48	-7.76	54.25	1.43
-6.61	-15.26	-10.47	-9.45	-7.76	54.94	1.45
-4.54	-28.82	-13.67	-10.92	-7.75	16.7	4.07
-7.97	-13.3	-11.45	-9.08	-7.75	64.45	1.9
-6.68	-16.81	-11.18	-9.7	-7.75	48.26	1.8
-6.76	-28.09	-12.9	-10.86	-7.74	17.87	3.11
-6.44	-28.51	-12.91	-10.89	-7.74	17.18	3.15
-8.58	-13.21	-12.25	-9.06	-7.74	64.83	2.8
-7.83	-13.62	-12.32	-9.14	-7.74	62.7	2.64
-7.56	-15.22	-11.88	-9.43	-7.74	55.02	2.22
-7.88	-13.43	-12.22	-9.1	-7.73	63.67	2.51
-7.51	-15.34	-11.52	-9.44	-7.73	54.45	2.06
-8.63	-15.37	-9.35	-9.46	-7.73	54.31	1.02
-3.16	-8.89	-9.03	-8.05	-7.73	91.43	0.81
-7.7	-14.43	-15.14	-9.28	-7.72	58.61	5.76
-8.67	-13.55	-13.62	-9.11	-7.71	62.95	3.9
-7.84	-13.49	-12.06	-9.08	-7.7	63.22	2.39
-7.39	-17.45	-14.72	-9.75	-7.7	45.57	5.26
-3.07	-25.72	-14.84	-10.66	-7.7	22.14	5.45
-3.85	-19.14	-5.44	-7.49	-7.68	30.41	0.94
-7.37	-21.65	-15.05	-10.27	-7.67	31.7	5.03
-6	-28.48	-13.42	-10.83	-7.66	17.13	3.66
-5.35	-28.43	-14.74	-10.82	-7.65	17.19	5.33
-8.67	-15.34	-9.89	-9.39	-7.65	54.1	1.2

-8.68	-15.37	-11.16	-9.39	-7.63	53.89	1.83
-7.95	-13.94	-15.2	-9.12	-7.63	60.58	5.63
-7.59	-17.64	-14.45	-9.73	-7.63	44.59	5.03
-2.89	-25.8	-14.86	-10.61	-7.63	21.85	5.59
-8.04	-13.29	-10.8	-8.99	-7.62	63.88	1.6
-7.42	-15.61	-11.59	-9.41	-7.62	52.79	2.32
-3.55	-28.78	-14.03	-10.81	-7.61	16.59	4.36
-2.25	-28.85	-13.68	-10.82	-7.61	16.47	3.8
-8.29	-13.16	-10.99	-8.95	-7.61	64.45	1.78
-7.8	-13.87	-11.48	-9.08	-7.59	60.81	2.15
-8.56	-15.44	-9.53	-9.37	-7.59	53.43	1.2
0.56	-24.27	-15.13	-10.45	-7.58	24.99	5.58
-2.59	-26.31	-15.49	-10.6	-7.56	20.75	7.07
-8.58	-13.25	-13.18	-8.94	-7.55	63.71	3.39
-8.67	-13.66	-13.84	-9.01	-7.55	61.65	4.17
-6.88	-16.58	-9.38	-9.51	-7.54	48.41	1.12
-6.85	-15.24	-9.83	-9.28	-7.54	54.07	1.18
-8.4	-13.24	-11.3	-8.91	-7.53	63.63	1.99
-8.37	-13.26	-11.33	-8.91	-7.53	63.55	2.05
-7.01	-14.94	-9.88	-9.22	-7.53	55.42	1.24
-4.16	-28.5	-14.51	-10.73	-7.52	16.9	4.9
-8.25	-13.14	-11.35	-8.89	-7.52	64.12	1.95
-7.55	-21.85	-11.64	-10.17	-7.52	30.81	2.16
1.08	-24.86	-15.02	-10.45	-7.52	23.59	5.69
-8.54	-12.96	-12.04	-8.84	-7.51	65.01	2.43
-7.6	-14.55	-12.38	-9.14	-7.51	57.12	2.89
-1.51	-29.32	-15.26	-10.77	-7.5	15.65	7.13
-1.87	-29	-16.03	-10.74	-7.49	16.1	7.79
-8.67	-13.6	-13.43	-8.96	-7.49	61.62	4.11
-7.6	-17.68	-14.06	-9.63	-7.49	43.96	4.87
1.28	-25.43	-15.01	-10.48	-7.49	22.34	6.02
-7.16	-16.88	-14.19	-9.51	-7.48	46.98	4.58
-7.45	-16.9	-11.7	-9.49	-7.46	46.85	2.44
-7.68	-22.12	-10.78	-10.16	-7.46	29.93	1.8
-7.74	-22.15	-11.29	-10.15	-7.45	29.84	2.22
-7.69	-13.94	-12.2	-8.98	-7.44	59.7	2.87
0.05	-28.7	-15.81	-10.68	-7.43	16.49	8.19
-7.59	-17.36	-12.97	-9.54	-7.43	44.93	3.51
-3.03	-29.12	-14.59	-10.69	-7.42	15.83	5.54
-7.57	-16.53	-13.95	-9.41	-7.42	48.15	4.25
-2.21	-28.8	-15.07	-10.67	-7.41	16.31	6.14
-6.29	-14.65	-12.77	-9.09	-7.41	56.21	3.34
-8.34	-13.29	-14.75	-8.82	-7.39	62.75	5.33
-6.86	-15.13	-9.38	-9.16	-7.39	53.99	1.06

-6.93	-14.37	-12.56	-9.02	-7.38	57.39	2.92
-7	-15.67	-9.95	-9.24	-7.38	51.56	1.32
-6.49	-16.92	-10.72	-9.44	-7.38	46.49	1.85
-7.36	-16.88	-11.83	-9.43	-7.37	46.61	2.55
-7.51	-13.98	-11.71	-8.93	-7.36	59.17	2.38
-7.57	-14.68	-11.89	-9.06	-7.36	55.87	2.44
-7.25	-16.91	-12.86	-9.42	-7.36	46.41	3.64
-8.51	-13.13	-12.49	-8.76	-7.35	63.32	3.03
0.45	-28.28	-15.47	-10.57	-7.33	17	7.89
-6.99	-16.2	-8.98	-9.28	-7.32	49.14	1
-7	-16.08	-9.6	-9.25	-7.31	49.57	1.23
-8.66	-15.32	-11.64	-9.14	-7.3	52.79	2.31
-8.43	-15.23	-10.73	-9.12	-7.3	53.15	1.89
-7.62	-13.99	-11.67	-8.89	-7.3	58.86	2.42
-8.56	-17.12	-9.75	-9.42	-7.3	45.44	1.46
-6.48	-17.41	-10.97	-9.45	-7.3	44.33	2.08
-7.71	-22.19	-11.16	-10.04	-7.29	29.36	2.05
-7.56	-17.11	-12.51	-9.39	-7.28	45.38	3.28
-7.46	-20.07	-9.31	-9.77	-7.25	35.21	1.21
-0.88	-28.97	-14.38	-10.55	-7.24	15.85	6.18
-7.47	-16.91	-12.19	-9.31	-7.21	45.9	2.92
-7.61	-22.54	-10.24	-10.01	-7.21	28.3	1.54
-1.21	-28.77	-15.72	-10.49	-7.18	16.06	7.68
-0.46	-28.51	-16.21	-10.48	-7.18	16.45	8.66
-6.88	-15.82	-12.39	-9.11	-7.18	50.15	3.28
-1.88	-25.59	-14.31	-10.26	-7.18	21.53	5.68
-8.74	-17.11	-9.9	-9.31	-7.16	44.95	1.53
-2.58	-28.68	-14.28	-10.47	-7.16	16.18	5.42
-8.39	-15.17	-10.97	-8.99	-7.15	52.76	2.19
-6.48	-21.37	-14.09	-9.86	-7.15	31.24	4.68
-6.83	-16.3	-12.87	-9.15	-7.13	48	3.8
-6.77	-16.17	-12.33	-9.14	-7.13	48.56	3.2
-6.89	-15.98	-12.31	-9.1	-7.13	49.29	3.18
-7.37	-21.09	-10.48	-9.8	-7.13	31.95	1.86
-0.12	-28.57	-14.73	-10.44	-7.12	16.3	6.29
0.73	-28.04	-15.58	-10.4	-7.12	17.11	8.32
-8.88	-17.09	-8.38	-9.27	-7.11	44.87	0.87
-1.6	-25.88	-14.28	-10.23	-7.11	20.84	5.79
-6.95	-16.05	-9.03	-9.09	-7.1	48.88	1.05
-2	-25.53	-14.24	-10.2	-7.1	21.5	5.46
-5.56	-15.15	-13.62	-8.94	-7.08	52.61	4.58
-7.33	-15.73	-10.28	-9.03	-7.08	50.11	1.74
-6.84	-14.92	-8.6	-8.89	-7.07	53.58	1.02
-6.87	-15.03	-9.01	-8.9	-7.06	53.03	1.15

-8.19	-16.99	-12.77	-9.21	-7.05	45.02	4.32
-7.15	-16.92	-12.75	-9.19	-7.05	45.31	3.91
-6.95	-16.47	-12.56	-9.13	-7.05	47.06	3.55
-0.58	-28.59	-14.91	-10.37	-7.03	16.16	6.59
-6.73	-15.94	-13.21	-9.03	-7.03	49.11	4.42
-6.97	-16.75	-14.06	-9.15	-7.02	45.85	4.94
-8.39	-15.21	-10.16	-8.9	-7.01	52.07	1.84
-2.46	-8.11	-8.25	-7.33	-7.01	91.85	0.78
-7.28	-15.91	-10.85	-9	-7	49.12	2.19
-8.7	-17.14	-9.64	-9.2	-7	44.32	1.5
-8.78	-16.46	-8.15	-9.09	-6.99	46.87	0.87
-7.29	-21.93	-10.52	-9.79	-6.99	29.36	1.88
-1.1	-25.95	-15.19	-10.15	-6.99	20.52	7.5
1.46	-27.08	-15.9	-10.24	-6.98	18.49	9.91
-8.66	-16.74	-9.43	-9.13	-6.98	45.74	1.37
1.02	-27.34	-15.51	-10.25	-6.97	18.04	8.67
-8.84	-17.15	-8.37	-9.17	-6.96	44.13	1.01
-1.43	-25.32	-14.03	-10.07	-6.96	21.67	5.65
-7.2	-16.1	-10.53	-9	-6.95	48.14	1.97
-1.1	-25.88	-15.02	-10.11	-6.95	20.59	7.17
-0.98	-25.66	-15.09	-10.1	-6.95	21.01	7.64
-8.58	-17.07	-10.32	-9.14	-6.94	44.36	1.64
-6.79	-14.84	-8.06	-8.77	-6.93	53.36	0.89
-7	-16.07	-10.16	-8.97	-6.91	48.1	1.81
-8.4	-17.06	-11.07	-9.12	-6.9	44.25	2.39
-7.04	-16.72	-12.55	-9.05	-6.9	45.53	3.72
-6.93	-15.17	-9.48	-8.8	-6.89	51.76	1.38
1.99	-25.17	-14.52	-10.01	-6.89	21.85	6.23
-6.65	-15.84	-13.48	-8.9	-6.88	48.91	5.05
-0.74	-25.96	-15.63	-10.06	-6.88	20.32	8.91
-6.23	-15.5	-9.02	-8.83	-6.87	50.29	1.33
-1.03	-25.16	-14.63	-10	-6.87	21.85	6.65
-7.11	-16.15	-10.52	-8.94	-6.86	47.6	2.09
-8.78	-17.15	-9.23	-9.1	-6.86	43.78	1.28
-6.74	-21.3	-10.14	-9.62	-6.85	30.71	1.94
-7.11	-12.88	-8.04	-8.33	-6.84	62.16	1
-7.01	-14.8	-8.34	-8.69	-6.83	53.1	0.94
1.79	-24.99	-14.47	-9.95	-6.83	22.1	5.61
-6.96	-15.28	-9.06	-8.76	-6.81	50.99	1.24
-6.91	-15.76	-9.83	-8.84	-6.8	48.96	1.68
-0.76	-25.1	-15.35	-9.94	-6.8	21.83	8.09
-8.38	-15.16	-10.16	-8.73	-6.79	51.39	1.98
-4.89	-15.74	-14.84	-8.81	-6.78	48.96	7.04
-8.45	-16.97	-9.34	-9.01	-6.78	44.19	1.38

-6.74	-14.39	-7.48	-8.58	-6.78	54.71	0.77
-0.62	-25.68	-15.75	-9.97	-6.78	20.69	9.58
-3.82	-8.09	-8.96	-7.16	-6.78	90.36	1.32
-1.79	-7.84	-8.94	-7.09	-6.78	92.22	1.17
-1.97	-7.79	-9.47	-7.06	-6.76	92.4	1.44
-8.27	-17.07	-12.19	-9	-6.74	43.68	3.58
1.25	-26.8	-15.6	-10.04	-6.74	18.63	8.65
-6.91	-15.78	-8.09	-8.79	-6.74	48.63	0.84
-1.08	-25.03	-13.94	-9.88	-6.73	21.86	5.66
-1.04	-24.27	-14.49	-9.81	-6.73	23.39	6.42
-3.84	-7.96	-7.13	-7.07	-6.7	90.74	0.64
-8.49	-17.21	-10	-8.98	-6.69	43.03	1.78
1.83	-26.46	-15.53	-9.97	-6.69	19.14	9.79
-6.84	-16.06	-9.36	-8.8	-6.69	47.35	1.53
-7.03	-14.93	-8.72	-8.6	-6.68	51.91	1.1
-8.79	-17.16	-8.97	-8.96	-6.67	43.12	1.34
-6.55	-15.56	-12.56	-8.7	-6.67	49.29	4.11
-6.54	-16.64	-12.06	-8.88	-6.67	45.03	3.15
-6.69	-16.86	-13.65	-8.89	-6.65	44.15	5.23
-8.3	-15.19	-10.66	-8.63	-6.64	50.66	2.5
-6.71	-22.89	-9.56	-9.62	-6.64	26.27	1.67
-8.64	-15.19	-10.88	-8.61	-6.61	50.56	2.31
-6.82	-23.25	-10.59	-9.64	-6.61	25.38	2.19
-6.35	-14.87	-11.95	-8.53	-6.6	51.87	3.54
-6.63	-14.48	-8.07	-8.47	-6.6	53.59	0.99
-0.36	-25.84	-15	-9.84	-6.6	20.11	8.63
-6.54	-15.59	-12.29	-8.65	-6.59	48.85	3.84
-3.75	-7.96	-8.79	-6.99	-6.59	89.98	1.27
-8.72	-17.09	-8.53	-8.87	-6.57	43.02	1.1
-6.37	-15.08	-11.51	-8.55	-6.57	50.9	3.07
-5.6	-21.73	-13.86	-9.45	-6.56	28.9	5.49
-4.63	-9.11	-10.69	-7.26	-6.55	82.06	2.45
-6.72	-16.16	-7.91	-8.71	-6.54	46.41	0.97
-7.07	-14.94	-8.32	-8.5	-6.54	51.32	1.07
-6.63	-22.33	-9.22	-9.5	-6.54	27.39	1.53
-4.26	-16.88	-14.93	-8.82	-6.53	43.69	7.79
-6.55	-15.14	-11.8	-8.52	-6.52	50.43	3.59
-6.22	-17.28	-13.99	-8.85	-6.51	42.17	6.31
4.2	-11.04	4.65	0.18	-6.5	29.25	1.44
-6.54	-21.91	-9.25	-9.42	-6.49	28.32	1.61
-5.86	-17.57	-13.75	-8.87	-6.48	41.06	6.14
-2.2	-7.47	-7.62	-6.78	-6.48	92.68	0.82
-4.5	-9.03	-10.47	-7.19	-6.47	82.03	2.26
-7.05	-15.04	-8	-8.47	-6.47	50.67	1.03

-3.71	-7.9	-8.16	-6.89	-6.47	89.55	1.08
-0.27	-25.99	-14.92	-9.75	-6.45	19.61	9.08
-5.69	-18.15	-13.76	-8.92	-6.44	38.99	6.17
-6	-17.55	-13.72	-8.83	-6.44	40.98	6.01
-5.98	-16.44	-8.09	-8.67	-6.44	44.98	1.06
2.57	-12.03	4.27	-0.69	-6.43	29.22	0.87
3.46	-13.14	1.53	-2.01	-6.43	30.43	1.18
8.8	-9.82	11.89	3.83	-6.42	21.4	1.2
-4.84	-9.08	-11.01	-7.17	-6.42	81.42	2.87
-3.68	-18.21	-15.06	-8.92	-6.42	38.73	8.67
-8.38	-17.12	-9.27	-8.76	-6.41	42.39	1.5
-5.47	-18.44	-13.74	-8.93	-6.4	37.92	6.13
-6.3	-14.91	-7.29	-8.39	-6.4	50.93	0.79
-6.44	-23.67	-9.12	-9.52	-6.4	24.08	1.55
-4.11	-8.9	-10.95	-7.1	-6.39	82.38	2.73
-6.76	-11.91	-7.07	-7.81	-6.39	64.92	0.75
-3.66	-7.9	-8.11	-6.83	-6.39	89.03	1.11
-5.68	-18.29	-8.91	-8.9	-6.38	38.33	1.43
-6.08	-17.9	-7.37	-8.85	-6.38	39.63	0.83
-5.71	-16.11	-8.56	-8.58	-6.38	46.04	1.29
-5.91	-16.14	-8.1	-8.57	-6.37	45.87	1.09
-5.88	-15.98	-7.77	-8.55	-6.37	46.52	0.95
-8.57	-14.63	-7.83	-8.33	-6.37	51.99	0.99
-4.29	-15.02	-9.33	-8.4	-6.36	50.32	1.7
-6.06	-17.77	-7.65	-8.81	-6.36	40.01	0.89
-6.51	-15	-9.07	-8.38	-6.36	50.38	1.49
-8.6	-14.53	-8.43	-8.31	-6.36	52.38	1.26
-1.79	-6.99	-8.81	-6.54	-6.35	95.22	1.55
-5.62	-15.47	-8.41	-8.45	-6.35	48.47	1.25
-6.3	-14.76	-6.39	-8.33	-6.35	51.36	0.55
-7.01	-14.57	-7.39	-8.29	-6.34	52.11	0.86
7.83	-14.84	5.55	-0.43	-6.33	21.41	1.18
-8.22	-14.87	-9.7	-8.35	-6.33	50.8	1.97
-6.96	-22.64	-9.28	-9.37	-6.33	26.22	1.8
2.67	-24.47	-14.17	-9.54	-6.33	22.29	6.66
-5.71	-18.23	-9.25	-8.86	-6.33	38.39	1.61
-7.8	-14.95	-10.48	-8.36	-6.32	50.47	2.67
-5.51	-16.53	-11.49	-8.6	-6.32	44.23	3.15
-4.83	-9.05	-10.2	-7.08	-6.31	80.94	2.27
-4.8	-9.02	-10.82	-7.07	-6.31	81.13	2.74
-4.57	-8.82	-11.05	-7.02	-6.31	82.4	3.02
-4.48	-8.58	-11.2	-6.96	-6.31	83.94	3.15
-4.4	-9.06	-12.02	-7.08	-6.31	80.86	3.98
-6.65	-16.29	-8.82	-8.56	-6.31	45.12	1.45

-4.32	-9.05	-11.82	-7.08	-6.3	80.89	3.78
-5.51	-16.12	-6.65	-8.52	-6.3	45.71	0.65
-5.47	-15.85	-7.07	-8.48	-6.3	46.76	0.82
-4.78	-9.08	-10.38	-7.08	-6.29	80.6	2.44
-3.56	-7.93	-8.9	-6.77	-6.29	88.18	1.56
-6.12	-18.35	-8.05	-8.84	-6.29	37.88	1.08
-6.05	-16.92	-7.22	-8.64	-6.29	42.75	0.77
-6.43	-15.78	-12.31	-8.46	-6.29	46.99	4.41
-4.33	-14.88	-9.57	-8.32	-6.28	50.59	1.81
-4.25	-15.08	-9.33	-8.35	-6.28	49.75	1.72
-4.25	-15.18	-9.8	-8.36	-6.28	49.33	2.01
-8.25	-17.06	-8.42	-8.66	-6.28	42.21	1.21
-5.77	-15.53	-6.06	-8.41	-6.28	47.95	0.61
-3.77	-8.88	-10.52	-7.01	-6.27	81.79	2.46
-5.91	-13.03	-8.93	-7.96	-6.27	58.8	1.55
-4.21	-15.24	-10.04	-8.37	-6.27	49.08	2.17
-6.6	-16.42	-9.66	-8.55	-6.27	44.5	2.02
-5.81	-15.21	-8.24	-8.35	-6.27	49.19	1.19
-4.75	-9.05	-9.83	-7.04	-6.26	80.61	2.07
-4.61	-8.94	-11.61	-7.01	-6.25	81.26	3.49
-4.54	-8.82	-10.64	-6.97	-6.25	81.99	2.7
-6.07	-17.04	-7.49	-8.63	-6.25	42.19	1.01
2.82	-24.41	-13.24	-9.47	-6.25	22.27	5.58
-4.67	-8.98	-10.19	-7.01	-6.24	80.91	2.38
-4.42	-8.71	-10.7	-6.94	-6.24	82.66	2.8
-7.07	-13.96	-7.61	-8.11	-6.24	54.38	0.87
-4.48	-8.69	-11.42	-6.93	-6.23	82.69	3.31
0	-25.73	-14.25	-9.56	-6.23	19.75	8.17
-6.35	-15.54	-12.05	-8.37	-6.22	47.68	4.12
-7.93	-15	-10.57	-8.29	-6.21	49.84	2.69
-8.04	-16.97	-10.51	-8.6	-6.21	42.28	2.89
-3.6	-7.76	-7.77	-6.67	-6.21	88.75	1.02
8.13	-14.45	5.91	-0.1	-6.2	21.42	0.99
-4.18	-15.3	-9.33	-8.33	-6.2	48.56	1.79
-5.88	-16.55	-6.88	-8.51	-6.19	43.74	0.73
-5.86	-12.98	-9.95	-7.88	-6.18	58.6	2.23
-4.74	-21.87	-13	-9.19	-6.18	27.75	5.23
2.39	-25.64	-14.96	-9.52	-6.17	19.81	9.8
-4.73	-8.92	-10.35	-6.95	-6.16	80.82	2.44
-4.65	-8.81	-11.26	-6.92	-6.16	81.52	3.26
-4.2	-8.99	-11.69	-6.96	-6.16	80.39	3.88
-6.44	-11.66	-7.4	-7.59	-6.16	65.08	1.02
-6.14	-11.63	-7	-7.58	-6.16	65.24	0.79
-4.41	-8.8	-11.87	-6.9	-6.15	81.47	3.84

-4.57	-18.9	-13.58	-8.81	-6.15	35.77	6.2
-8.2	-14.7	-7.69	-8.19	-6.14	50.81	1.03
-6.85	-23.88	-10.9	-9.34	-6.14	23.15	2.68
-8.09	-14.63	-8.14	-8.16	-6.13	51.06	1.25
-8.15	-14.59	-8.13	-8.16	-6.13	51.2	1.22
-4.32	-14.74	-9.57	-8.18	-6.12	50.56	1.9
-4.14	-15.38	-9.76	-8.29	-6.12	47.95	2.13
-6.42	-12.01	-6.66	-7.63	-6.12	63.08	0.78
-5.88	-10.04	-9.01	-7.19	-6.12	73.81	1.68
-6.32	-15.56	-12.45	-8.3	-6.11	47.19	4.91
-8.77	-21	-9.36	-9.05	-6.1	29.76	1.86
-8.62	-20.94	-9.97	-9.04	-6.1	29.91	2.4
-6.64	-16.06	-8.06	-8.37	-6.1	45.26	1.07
-5.73	-9.86	-9.65	-7.13	-6.1	74.73	2.05
-1.65	-6.76	-7.73	-6.3	-6.1	95.1	1.02
-6.16	-11.62	-7.76	-7.53	-6.09	64.96	1.03
-6.05	-11.53	-9.02	-7.51	-6.09	65.39	1.66
-7.94	-14.99	-10.46	-8.19	-6.08	49.36	2.62
-7.94	-14.92	-10.2	-8.18	-6.08	49.64	2.32
-7.88	-14.93	-10.5	-8.18	-6.08	49.62	2.54
-8.5	-20.29	-10.42	-8.95	-6.08	31.57	2.65
-6	-11.17	-9.48	-7.42	-6.08	67.23	1.84
-3.5	-7.9	-8.04	-6.62	-6.08	86.94	1.22
-5.44	-18.04	-8.01	-8.64	-6.07	38.26	1.2
-3.09	-18.24	-14.58	-8.68	-6.07	37.62	8.23
-6.48	-16.49	-8.64	-8.41	-6.07	43.56	1.54
-8.45	-14.62	-7.83	-8.12	-6.07	50.88	1.05
-6.9	-23.9	-10.34	-9.29	-6.07	23	2.52
-5.02	-17.09	-10.76	-8.5	-6.06	41.41	2.75
-5.8	-12.8	-10.17	-7.75	-6.05	58.93	2.5
-5.81	-10.26	-9.37	-7.19	-6.05	72.14	2.17
-15.17	-33.87	-18.2	-18.49	-6.04	21.45	0.72
-4.11	-15.45	-9.57	-8.24	-6.04	47.4	2.04
-8.47	-20.76	-10.1	-8.97	-6.04	30.24	2.52
-4.09	-15.51	-9.77	-8.24	-6.03	47.09	2.19
-8.6	-14.83	-8.72	-8.13	-6.03	49.85	1.44
2.94	-24.17	-12.54	-9.29	-6.03	22.38	5.3
-7.71	-14.82	-10	-8.12	-6.02	49.82	2.42
-5.72	-12.07	-10.57	-7.58	-6.02	62.26	2.91
-4.07	-15.52	-10.35	-8.24	-6.02	47.04	2.65
-8.85	-20.73	-9.42	-8.96	-6.02	30.27	1.94
-5.97	-16.73	-7.3	-8.42	-6.02	42.54	0.93
-6.09	-13.99	-7.72	-7.95	-6.02	53.31	1.08
-5.23	-9.62	-7.44	-7.01	-6.02	75.67	1.04

-7.74	-14.99	-10.3	-8.14	-6.01	49.09	2.61
-4.03	-15.59	-10.31	-8.24	-6.01	46.73	2.66
-6.27	-15.17	-11.18	-8.16	-6.01	48.36	3.48
-6.6	-14.26	-7.44	-8	-6.01	52.14	0.91
-5.41	-9.77	-6.18	-7.04	-6.01	74.78	0.64
-0.22	-23.56	-14.22	-9.2	-5.99	23.55	8.48
-8.07	-14.31	-8.19	-7.99	-5.98	51.79	1.3
-0.18	-24.26	-14.56	-9.26	-5.98	22.11	8.93
-5.83	-9.34	-10.23	-6.92	-5.98	77.1	2.57
-5.8	-9.96	-6.42	-7.07	-5.97	73.42	0.71
-3.68	-7.63	-6.47	-6.46	-5.97	88.06	0.67
22.12	-6.64	15.64	6.41	-5.96	21.5	0.9
-6.22	-14.39	-10.11	-7.99	-5.96	51.43	2.49
-3.96	-15.66	-10.13	-8.21	-5.95	46.25	2.53
-6.22	-14.82	-10.54	-8.05	-5.95	49.56	2.88
-3.99	-15.59	-10.47	-8.19	-5.94	46.48	2.81
-3.45	-7.87	-7.15	-6.51	-5.93	86.19	0.93
-6.41	-16.45	-7.48	-8.3	-5.92	43.21	1.08
-6.09	-13.48	-7.21	-7.79	-5.92	55.18	0.95
-6.17	-13.95	-9.57	-7.87	-5.91	53.05	2.05
-0.6	-22.1	-13.88	-9.01	-5.91	26.65	7.93
-0.24	-24.24	-14.72	-9.2	-5.9	22.03	9.48
-5.86	-15.3	-6.3	-8.09	-5.89	47.42	0.71
-7.67	-14.7	-9.55	-7.99	-5.88	49.79	2.23
-4.17	-10.14	-11.01	-7.05	-5.88	71.83	3.5
-5.69	-12.39	-9.78	-7.55	-5.88	60.08	2.49
-5.65	-9.96	-9.56	-7	-5.88	72.91	2.18
-5.61	-10.06	-8.48	-7.03	-5.88	72.37	1.67
20.4	-6.93	16.38	6.17	-5.87	21.51	0.67
-3.89	-15.77	-10.6	-8.17	-5.87	45.59	3.06
2.5	-25.38	-13.51	-9.28	-5.87	19.84	7.78
2.92	-24.19	-12.48	-9.17	-5.87	22.06	5.29
-8	-14.87	-9.77	-8.01	-5.86	49.01	2.22
-5.73	-12.92	-10.09	-7.64	-5.86	57.48	2.52
-6.33	-16.75	-7.47	-8.3	-5.86	41.96	1.04
-8.07	-16.32	-7.52	-8.24	-5.86	43.48	0.99
-5.92	-14.65	-7.93	-7.96	-5.86	49.93	1.26
-6.16	-11.53	-7.08	-7.35	-5.86	64.28	0.84
-6.57	-15.72	-7.08	-8.12	-5.84	45.62	0.85
-8.36	-14.84	-8.17	-8	-5.84	49.1	1.24
-5.97	-13.74	-7.88	-7.78	-5.84	53.73	1.25
-0.2	-24.55	-14.04	-9.18	-5.84	21.31	8.03
-5.3	-9.59	-7.01	-6.88	-5.84	74.83	0.85
-6.18	-16.86	-7.22	-8.3	-5.83	41.47	0.81

-3.97	-22.1	-13.1	-8.96	-5.83	26.48	5.97
-3.68	-7.33	-5.41	-6.28	-5.83	89.2	0.49
-5.76	-10.44	-6.3	-7.07	-5.82	69.86	0.71
-5.21	-9.83	-5.74	-6.93	-5.82	73.27	0.56
-4.2	-10.15	-10.7	-7	-5.81	71.42	3.37
-4.31	-14.05	-9.26	-7.83	-5.81	52.26	1.92
-6.21	-16.99	-6.45	-8.3	-5.81	40.96	0.75
-5.9	-15.34	-6.7	-8.04	-5.81	46.96	0.78
-4.32	-14.02	-8.22	-7.82	-5.81	52.38	1.42
-6.41	-11.72	-7.29	-7.35	-5.81	63.07	1.13
-3.74	-20.63	-14.07	-8.77	-5.8	30.03	7.96
-6.53	-14.33	-6.85	-7.86	-5.8	51.01	0.79
2.82	-25.31	-13.1	-9.21	-5.8	19.84	6.64
-3.76	-7.35	-7.64	-6.26	-5.8	88.82	1.17
-3.85	-15.91	-10.61	-8.13	-5.79	44.75	3.13
-5.09	-9.78	-6.61	-6.89	-5.79	73.44	0.78
-4.07	-9.98	-11.71	-6.95	-5.78	72.26	4.24
-5.16	-23.9	-8.58	-9.08	-5.78	22.49	1.67
2.92	-23.74	-12.94	-9.07	-5.78	22.82	5.6
-5.33	-9.68	-6.77	-6.86	-5.78	73.93	0.83
-3.38	-7.87	-7.13	-6.4	-5.78	85.23	1.03
-8.14	-21.6	-10.14	-8.86	-5.76	27.5	2.78
-8.06	-14.64	-7.08	-7.9	-5.76	49.59	0.99
-1.39	-6.41	-6.35	-5.96	-5.76	95.21	0.67
-6.33	-13.96	-6.31	-7.75	-5.75	52.39	0.71
-5.67	-10.02	-6.06	-6.92	-5.75	71.81	0.78
-7.6	-14.54	-9.27	-7.87	-5.74	49.96	2.09
-2.65	-8.62	-11	-6.57	-5.74	80.13	3.39
-6.43	-14.18	-6.63	-7.79	-5.74	51.41	0.78
-6.31	-11.6	-6.55	-7.28	-5.74	63.36	0.76
-5.17	-9.84	-5.41	-6.86	-5.73	72.73	0.49
-4.08	-8.62	-10.11	-6.56	-5.72	80.05	2.93
-3.16	-21.94	-13.16	-8.86	-5.72	26.64	6.68
-3.74	-16.45	-11.02	-8.16	-5.72	42.55	3.74
2.94	-23.9	-12.39	-9.04	-5.72	22.4	5.13
-7.76	-17.13	-10.05	-8.26	-5.71	40.19	2.84
-8.24	-21.39	-10.27	-8.81	-5.71	27.92	2.98
-7.88	-16.92	-10.96	-8.22	-5.7	40.86	3.73
-7.84	-16.91	-10.18	-8.22	-5.7	40.92	2.88
-2.45	-18.55	-14.1	-8.44	-5.7	35.62	7.95
-5.98	-14.02	-8.1	-7.72	-5.69	51.87	1.48
2.88	-24.76	-12.44	-9.09	-5.69	20.69	5.37
-1.09	-20.04	-13.51	-8.62	-5.69	31.32	7.76
-3.55	-7.12	-5.83	-6.12	-5.69	89.6	0.6

-1.88	-20.37	-14.01	-8.66	-5.68	30.43	8.61
-7.98	-14.81	-7.61	-7.87	-5.68	48.59	1.17
-0.88	-20.31	-13.94	-8.64	-5.68	30.58	8.46
-1.07	-6.3	-8.03	-5.87	-5.68	95.35	1.39
-7.53	-14.37	-8.58	-7.79	-5.67	50.36	1.68
-7.48	-14.28	-8.28	-7.77	-5.67	50.73	1.59
-2.42	-20.36	-14.06	-8.64	-5.67	30.43	8.72
-4.52	-16.78	-9.34	-8.17	-5.67	41.24	1.98
-5.17	-18.53	-8.25	-8.42	-5.67	35.6	1.52
-8.14	-14.89	-6.98	-7.87	-5.66	48.23	1.04
-3.66	-7.22	-6.97	-6.13	-5.66	88.79	0.95
-3.78	-16.12	-9.77	-8.07	-5.65	43.51	2.53
-3.75	-16.19	-9.38	-8.07	-5.65	43.25	2.25
-3.64	-7.37	-8.53	-6.17	-5.65	87.67	1.62
-5.36	-8.25	-8.69	-6.4	-5.65	81.91	1.82
-3.71	-7.13	-5.59	-6.09	-5.65	89.28	0.53
-3.88	-8.61	-10.74	-6.5	-5.64	79.57	3.52
-3.62	-16.45	-10.17	-8.11	-5.64	42.31	2.92
1.08	-21.81	-13.94	-8.79	-5.64	26.76	9.08
-5.84	-15.39	-5.86	-7.93	-5.64	46.22	0.7
-5.1	-18.67	-7.36	-8.4	-5.63	35.05	1.18
-8.33	-20.96	-10.36	-8.7	-5.63	28.8	3.07
-3.66	-16.36	-11.15	-8.08	-5.63	42.6	3.85
-4.89	-23.83	-9.47	-8.96	-5.63	22.38	2.27
-5.57	-9.79	-7.66	-6.79	-5.63	72.51	1.38
-0.81	-6.25	-8.15	-5.82	-5.62	95.34	1.45
-5.11	-12.49	-10.47	-7.38	-5.61	58.41	3.33
-5.58	-12.73	-9.19	-7.42	-5.61	57.26	2.05
-5.17	-18.04	-7.34	-8.3	-5.61	36.92	1.09
-1.8	-6.19	-8.45	-5.79	-5.61	95.71	1.65
-6.52	-16	-7.45	-8	-5.61	43.83	1.06
-4.32	-14.47	-8.11	-7.76	-5.61	49.73	1.44
-0.37	-20.79	-13.17	-8.65	-5.61	29.19	6.63
-5.69	-8.4	-8.46	-6.41	-5.61	80.71	1.68
-5.62	-11.69	-8.88	-7.19	-5.59	62.14	2.08
-5.61	-16.96	-5.95	-8.14	-5.59	40.38	0.76
-4.94	-10.04	-5.02	-6.81	-5.59	70.85	0.46
-4.98	-10.03	-5.56	-6.81	-5.59	70.91	0.57
-5.03	-7.09	-6.91	-6.05	-5.59	89.22	0.96
-3.7	-16.37	-9.48	-8.06	-5.58	42.4	2.36
-8.1	-21.42	-9.58	-8.71	-5.58	27.58	2.56
-1.74	-6.16	-5.25	-5.76	-5.58	95.64	0.53
-3.75	-9.75	-11.33	-6.74	-5.57	72.42	4.23
-5.33	-11.43	-9.77	-7.12	-5.57	63.39	2.6

-5.29	-12.22	-9.46	-7.29	-5.57	59.52	2.39
-3.66	-16.44	-9.56	-8.06	-5.57	42.12	2.41
-5.5	-8.23	-7.85	-6.35	-5.57	81.55	1.39
-4.89	-7.15	-6.65	-6.05	-5.57	88.65	0.9
-4.95	-7.11	-7.45	-6.03	-5.57	88.89	1.18
-5.01	-7.07	-7.72	-6.02	-5.57	89.14	1.3
-1.01	-6.47	-6.54	-5.85	-5.57	93.33	0.84
-3.79	-8.54	-11.08	-6.42	-5.56	79.53	4.06
-3.92	-9.72	-11.58	-6.73	-5.56	72.48	4.41
-3.59	-16.56	-10.57	-8.07	-5.56	41.66	3.38
-3.51	-16.73	-10.44	-8.09	-5.56	41.05	3.35
-8.2	-21.47	-10	-8.7	-5.56	27.4	2.69
-1.76	-20.13	-13.41	-8.53	-5.56	30.77	7.66
-3.78	-16.53	-10.82	-8.06	-5.56	41.75	3.64
2.71	-25.12	-13.03	-9.02	-5.56	19.83	6.94
-5.77	-10.06	-10	-6.8	-5.56	70.56	2.41
-8.31	-15.04	-8.38	-7.82	-5.55	47.23	1.55
-7.78	-16.92	-10.36	-8.12	-5.55	40.39	3.3
-7.74	-16.94	-9.97	-8.12	-5.55	40.33	2.78
-6.22	-12.99	-6.33	-7.43	-5.55	55.81	0.73
-3.75	-16.6	-11.18	-8.07	-5.55	41.52	4.12
-4.35	-23.85	-10.12	-8.91	-5.55	22.21	2.96
-7.87	-16.95	-10.8	-8.11	-5.54	40.25	3.52
1.29	-21.83	-14.58	-8.72	-5.54	26.53	9.96
-3.8	-16.46	-10.27	-8.03	-5.54	41.92	3.07
-0.79	-19.41	-12.94	-8.43	-5.54	32.7	6.62
-5.86	-10.18	-7.19	-6.82	-5.54	69.84	1.09
-5.76	-8.49	-9.07	-6.39	-5.54	79.73	1.9
-4.85	-7.16	-6.93	-6.03	-5.54	88.41	1.01
-7.36	-14.13	-8.51	-7.64	-5.53	50.81	1.76
-7.67	-17.08	-10.12	-8.12	-5.53	39.79	2.86
-5.89	-14.16	-8.68	-7.63	-5.53	50.7	1.83
-3.29	-7.81	-8.05	-6.2	-5.52	83.93	1.52
-7.9	-14.85	-7.91	-7.77	-5.52	47.88	1.34
-4.83	-7.12	-6.89	-6	-5.52	88.45	1.03
-4.17	-10.05	-10.11	-6.77	-5.51	70.41	3.09
-5.5	-12.05	-8.66	-7.21	-5.51	60.02	1.89
2.47	-24.53	-12.84	-8.95	-5.51	20.83	7.11
-3.66	-16.59	-10.93	-8.04	-5.51	41.41	3.83
-7.5	-17.02	-9.83	-8.09	-5.5	39.91	2.96
-5	-18.43	-5.93	-8.28	-5.5	35.44	0.74
0.5	-21.33	-13.71	-8.64	-5.5	27.62	9.39
-4.9	-10.08	-5.86	-6.76	-5.5	70.15	0.67
-3.98	-8.5	-10.18	-6.36	-5.49	79.36	2.96

-3.19	-8.52	-10.8	-6.37	-5.49	79.26	3.79
2.63	-25.04	-13.06	-8.98	-5.49	19.87	7.02
-3.44	-7.3	-5.95	-6.03	-5.49	87.09	0.69
-4.83	-7.12	-7.01	-5.98	-5.49	88.29	1.05
-4.86	-7.07	-6.27	-5.97	-5.49	88.62	0.78
-5.4	-12.53	-10.13	-7.29	-5.48	57.62	2.95
-3.05	-20.2	-13.42	-8.48	-5.48	30.42	7.33
-3.7	-16.72	-10.93	-8.04	-5.48	40.86	3.86
-3.61	-16.45	-11.08	-7.99	-5.48	41.79	4.11
-4.14	-24.16	-10.21	-8.88	-5.48	21.49	3.18
-5.18	-7.93	-9.62	-6.2	-5.48	82.83	2.47
-5.57	-8.19	-8.09	-6.27	-5.48	81.23	1.52
-5.26	-12.52	-10.22	-7.28	-5.47	57.59	3.04
-1.77	-6.04	-8.13	-5.65	-5.47	95.75	1.6
0.79	-21.53	-14.58	-8.64	-5.47	27.08	10.34
-5.95	-9.88	-6.97	-6.69	-5.47	71.1	1.01
-3.29	-7.58	-5.24	-6.11	-5.47	85.1	0.56
-3.28	-7.55	-4.45	-6.09	-5.47	85.27	0.37
-1.78	-6.02	-8.77	-5.64	-5.46	95.81	1.94
-3.33	-7.03	-8.53	-5.93	-5.46	88.68	1.87
-3.29	-7	-8.71	-5.93	-5.46	88.9	1.96
-7.33	-14.04	-7.9	-7.57	-5.46	50.9	1.47
-3.3	-7.56	-4.83	-6.09	-5.46	85.19	0.45
-4.55	-7.02	-7.23	-5.93	-5.46	88.79	1.18
-7.74	-17.01	-10.61	-8.06	-5.45	39.8	3.47
-5.88	-13.76	-8.37	-7.51	-5.45	52.03	1.74
-3.38	-7.14	-8.44	-5.97	-5.45	87.92	1.72
-4.85	-10.08	-5.09	-6.73	-5.45	69.92	0.53
-3.56	-9.63	-10.68	-6.62	-5.44	72.4	3.54
-5.8	-13.56	-7.95	-7.46	-5.44	52.86	1.5
-7.91	-21.4	-8.93	-8.6	-5.43	27.3	2.05
-1.49	-6.02	-6.69	-5.62	-5.43	95.68	0.93
-3.2	-8.42	-10.63	-6.29	-5.42	79.41	3.53
-3.12	-9.46	-9.71	-6.56	-5.42	73.19	2.67
-3.46	-16.74	-10.09	-7.99	-5.42	40.61	3.1
-7.77	-21.32	-9.69	-8.59	-5.42	27.48	2.65
2.8	-25.01	-12.54	-8.91	-5.42	19.81	6.03
-4.95	-6.98	-6.59	-5.89	-5.42	88.78	0.94
-5.19	-12.87	-10.25	-7.31	-5.41	55.78	3.39
-1.34	-20.3	-13.78	-8.45	-5.41	29.99	8.52
-3.53	-16.43	-11.08	-7.94	-5.41	41.65	4.3
2.29	-24.03	-14.28	-8.82	-5.4	21.59	9.88
-3.42	-7.11	-7.21	-5.92	-5.4	87.74	1.12
-2.21	-8.41	-10.21	-6.27	-5.39	79.34	3.03

-3.35	-9.58	-9.98	-6.57	-5.39	72.41	2.96
2.32	-25.45	-12.69	-8.92	-5.39	19	6.56
-5.75	-10.19	-7.39	-6.71	-5.39	68.96	1.2
-3.12	-8.45	-10.27	-6.28	-5.38	79.04	3.32
-7.33	-14.12	-7.66	-7.53	-5.38	50.27	1.39
-1.17	-20.65	-13.85	-8.46	-5.38	29.03	9.03
-1.45	-5.94	-6.71	-5.56	-5.38	95.78	1.01
-3.41	-8.32	-10.87	-6.23	-5.37	79.72	3.98
-4.31	-14.65	-8.08	-7.63	-5.37	48.11	1.58
-4.3	-14.73	-8.58	-7.64	-5.37	47.8	1.78
-0.63	-6.26	-5.96	-5.64	-5.37	93.48	0.71
-0.31	-20.51	-13.65	-8.44	-5.36	29.34	8.87
-5.56	-9.79	-5.96	-6.6	-5.36	71.04	0.87
-5.52	-9.62	-5.27	-6.55	-5.36	71.96	0.54
-4.27	-6.97	-6.3	-5.85	-5.36	88.38	0.84
-4.91	-6.95	-5.92	-5.85	-5.36	88.58	0.73
-4.13	-9.86	-10.29	-6.61	-5.35	70.56	3.39
-8.03	-21.46	-8.63	-8.55	-5.35	27	1.85
-3.58	-8.24	-10.43	-6.19	-5.34	80.05	3.59
-3.19	-8.37	-10.61	-6.22	-5.34	79.27	3.68
-3.37	-8.38	-10.56	-6.23	-5.34	79.19	3.69
-3.48	-8.32	-10.45	-6.21	-5.34	79.57	3.47
-3.34	-8.31	-10.7	-6.21	-5.34	79.65	3.89
2.01	-23.14	-13.05	-8.7	-5.34	23.28	8.06
-5.73	-13.53	-7.85	-7.38	-5.34	52.58	1.54
-4.67	-9.91	-5.9	-6.61	-5.34	70.24	0.78
-3.41	-16.66	-10.26	-7.92	-5.33	40.6	3.4
2.39	-23.59	-13.42	-8.74	-5.33	22.35	8.32
-8.19	-15.04	-7.83	-7.66	-5.33	46.41	1.41
-7.74	-14.44	-7.84	-7.56	-5.33	48.76	1.42
2.51	-25.1	-13.15	-8.85	-5.33	19.52	7.34
-4.86	-7.85	-9.65	-6.07	-5.33	82.44	2.71
-3.32	-7.55	-6.16	-6	-5.33	84.41	0.77
-8.05	-21.59	-8.69	-8.54	-5.32	26.65	1.99
2.53	-24.53	-14.01	-8.81	-5.32	20.54	8.91
-4.97	-7.8	-9.53	-6.06	-5.32	82.68	2.51
-3.23	-7.06	-7.65	-5.85	-5.32	87.55	1.49
-1.9	-5.86	-7.62	-5.49	-5.32	96	1.42
-1.33	-20.8	-14.04	-8.43	-5.31	28.5	10.05
-1.87	-5.87	-4.99	-5.49	-5.31	95.86	0.5
-4.9	-18.31	-5.91	-8.12	-5.3	35.26	0.73
-3.96	-17.41	-9.54	-8	-5.3	38.05	2.4
-6.09	-10.89	-7.05	-6.81	-5.3	64.84	1.01
-4.78	-9.82	-4.58	-6.56	-5.3	70.55	0.45

-7.66	-21.68	-8.41	-8.53	-5.29	26.35	1.85
-7.58	-20.97	-7.98	-8.45	-5.29	28.06	1.58
-5.6	-15.98	-7.34	-7.77	-5.29	42.82	1.36
-7.21	-15.53	-6.18	-7.71	-5.29	44.48	0.72
-3.39	-7.49	-5.42	-5.95	-5.29	84.53	0.59
-3.4	-7.45	-5.03	-5.94	-5.29	84.78	0.51
-4.1	-6.94	-6.28	-5.8	-5.29	88.16	0.85
-3.59	-8.37	-9.65	-6.18	-5.28	78.88	2.82
-3.42	-8.26	-9.83	-6.15	-5.27	79.53	2.9
-3.44	-8.23	-10.11	-6.14	-5.27	79.67	3.31
-3.24	-8.28	-10.24	-6.15	-5.27	79.4	3.28
-3.35	-8.22	-10.47	-6.13	-5.27	79.72	3.75
-4.08	-9.93	-10.3	-6.57	-5.27	69.8	3.66
-4.93	-17.87	-5.38	-8.03	-5.27	36.5	0.6
-7.45	-21.42	-8.18	-8.49	-5.27	26.92	1.91
-4.93	-6.8	-6.26	-5.74	-5.27	88.96	0.81
-4.63	-6.77	-6.36	-5.73	-5.27	89.15	0.88
-5.54	-12.32	-9.51	-7.09	-5.26	57.66	2.5
-3.46	-16.46	-10.96	-7.83	-5.26	41.07	4.45
-7.45	-17.09	-9.04	-7.93	-5.25	38.93	2.43
-0.81	-20.25	-13.57	-8.34	-5.25	29.76	8.38
-5.57	-9.68	-6.32	-6.49	-5.25	71.07	0.89
-4.45	-7.78	-9.56	-6	-5.25	82.4	2.69
-3.18	-7.83	-6.5	-6.02	-5.25	82.05	0.97
-1.19	-20.25	-14.09	-8.31	-5.24	29.74	10.09
5.11	-13.09	1.77	-1.97	-5.23	30.43	1.03
0.03	-20.68	-14.39	-8.37	-5.23	28.63	10.36
-3.45	-16.29	-10.45	-7.78	-5.23	41.54	3.49
-1.42	-20.44	-14.05	-8.33	-5.23	29.23	10.41
-1.49	-5.82	-5	-5.42	-5.23	95.66	0.48
-5.63	-13.2	-7.91	-7.24	-5.22	53.52	1.64
-7.63	-14.2	-7.72	-7.44	-5.22	49.35	1.47
-3.92	-6.92	-5.91	-5.74	-5.22	87.85	0.75
-2.35	-22.97	-11.94	-8.59	-5.21	23.38	5.67
-6.16	-11.09	-4.69	-6.79	-5.21	63.34	0.49
-1.4	-5.75	-5.99	-5.38	-5.21	95.95	0.82
-7.24	-14.2	-8.79	-7.42	-5.2	49.26	2.29
-7.07	-14.59	-8.77	-7.49	-5.2	47.71	2.18
-5.14	-12.92	-9.34	-7.17	-5.2	54.66	2.74
-4.67	-9.68	-5.92	-6.46	-5.2	70.76	0.74
-4.45	-9.54	-5.25	-6.42	-5.2	71.52	0.58
-4.67	-6.92	-5.82	-5.73	-5.2	87.75	0.77
-7.19	-13.57	-7.27	-7.3	-5.19	51.82	1.26
2.07	-25.2	-11.97	-8.76	-5.19	19.14	5.98

-4.36	-9.42	-5.93	-6.38	-5.19	72.14	0.76
-3.47	-7.16	-7.41	-5.79	-5.19	86.06	1.24
-3.32	-16.63	-10.91	-7.81	-5.18	40.24	4.4
-5.42	-16.14	-8.6	-7.71	-5.17	41.89	2.13
-4.36	-9.53	-6.65	-6.4	-5.17	71.5	1.03
-3.2	-6.92	-6.98	-5.71	-5.17	87.51	1.18
-1.79	-5.7	-7.41	-5.34	-5.17	96.07	1.41
-5.66	-15.41	-7.31	-7.59	-5.16	44.45	1.34
-1.91	-19.26	-13.5	-8.14	-5.16	32.18	9.57
-5.5	-8.79	-7.27	-6.21	-5.16	75.65	1.25
-7.32	-21.56	-8.15	-8.41	-5.15	26.36	2.04
-5.37	-13.59	-7.82	-7.26	-5.15	51.58	1.61
-3.19	-6.95	-7.85	-5.7	-5.15	87.22	1.65
-1.44	-19.98	-13.91	-8.22	-5.15	30.22	10.25
-4.72	-9.64	-3.92	-6.41	-5.15	70.77	0.35
-1.5	-5.73	-6.29	-5.34	-5.15	95.72	0.87
-1.76	-5.69	-8.28	-5.32	-5.14	95.92	1.73
-1.1	-22.81	-11.62	-8.52	-5.14	23.6	5.68
-5.52	-13.58	-8.06	-7.25	-5.14	51.58	1.75
-5.46	-13.6	-7.87	-7.25	-5.14	51.5	1.64
-3.2	-6.99	-7.85	-5.71	-5.14	86.86	1.59
-3.71	-6.9	-5.67	-5.68	-5.14	87.49	0.71
-4.29	-14.66	-8.93	-7.45	-5.13	47.19	2.26
-5.51	-17.17	-5.59	-7.84	-5.13	38.33	0.69
-7.07	-14.56	-8.84	-7.42	-5.12	47.53	2.25
-6.99	-14.52	-8.63	-7.42	-5.12	47.7	2.17
-5.38	-17.18	-6.01	-7.84	-5.12	38.27	0.83
1	-25.13	-12.2	-8.7	-5.12	19.17	6.45
2.34	-25.2	-11.94	-8.71	-5.12	19.05	5.63
-4.61	-6.83	-5.59	-5.64	-5.12	87.77	0.72
-5.22	-13.35	-6.8	-7.18	-5.11	52.42	1.15
-3.41	-24.56	-10.85	-8.64	-5.1	20.14	4.35
-1.73	-18.33	-13.54	-7.97	-5.1	34.67	9.26
-3.25	-16.76	-10.65	-7.76	-5.09	39.56	4.16
2.25	-22.89	-13.57	-8.5	-5.09	23.36	8.72
-0.98	-19.52	-12.97	-8.12	-5.09	31.3	7.28
-5.29	-13.47	-7.53	-7.19	-5.09	51.84	1.46
-1.52	-18.62	-12.67	-8	-5.08	33.79	7.37
-5.35	-16.12	-7.77	-7.64	-5.08	41.67	1.77
-3.5	-6.9	-5.66	-5.64	-5.08	87.07	0.72
-5.56	-15.91	-7.37	-7.61	-5.07	42.37	1.39
-6.91	-14.46	-8.52	-7.37	-5.06	47.71	2.12
-7.14	-17.34	-8.25	-7.82	-5.06	37.58	1.93
-0.27	-20.34	-13.13	-8.19	-5.06	29.1	8.27

-4.74	-9.9	-4.48	-6.41	-5.06	68.85	0.45
-5.52	-15.88	-7.68	-7.59	-5.06	42.46	1.51
-5.4	-16.15	-8.01	-7.64	-5.06	41.5	1.78
-4.69	-9.6	-4.69	-6.34	-5.06	70.47	0.48
-4.42	-19.46	-8.42	-8.09	-5.06	31.39	2.09
-7.18	-14.23	-8.68	-7.32	-5.05	48.59	2.41
-6.9	-14.52	-8.58	-7.37	-5.05	47.43	2.19
-5.29	-17.29	-6.27	-7.8	-5.05	37.7	1.01
-6.96	-14.51	-8.78	-7.36	-5.04	47.46	2.3
-3.27	-8.13	-9.92	-5.95	-5.04	78.95	3.48
-6.99	-17.3	-8.74	-7.81	-5.04	37.67	2.29
-2.34	-9.14	-9.45	-6.22	-5.04	72.96	2.69
-4.01	-9.8	-10.11	-6.39	-5.04	69.26	3.68
-4.83	-11.96	-8.81	-6.86	-5.04	58.37	2.37
-4.45	-19.72	-8.95	-8.11	-5.04	30.65	2.44
-7.25	-17.09	-9.66	-7.77	-5.03	38.29	3.09
-3.24	-6.9	-7.98	-5.61	-5.03	86.76	1.8
-5.97	-13.19	-7.12	-7.11	-5.03	52.83	1.27
-3.39	-16.13	-10.28	-7.62	-5.03	41.48	3.84
-3.04	-8.04	-6.47	-5.93	-5.03	79.46	1.03
0.28	-21.44	-13.11	-8.29	-5.02	26.38	8.57
-5.15	-13.09	-7.27	-7.07	-5.02	53.19	1.39
-3.97	-15.03	-5.97	-7.42	-5.01	45.38	0.9
-5.08	-13.04	-7.32	-7.05	-5.01	53.36	1.44
-6.83	-14.52	-8.86	-7.33	-5	47.27	2.45
-2.37	-17.66	-13.05	-7.81	-5	36.42	8.48
-2.58	-18.24	-13.48	-7.89	-5	34.66	9.55
-3.2	-6.95	-8.48	-5.6	-4.99	86.12	2.19
-2.51	-18.55	-13.59	-7.92	-4.98	33.74	9.79
-7.18	-17.27	-8.8	-7.75	-4.97	37.54	2.38
0.42	-25.08	-11.98	-8.59	-4.97	19.03	6.43
-2.99	-8.13	-6.03	-5.91	-4.97	78.51	0.95
-7.37	-17.09	-8.95	-7.72	-4.96	38.09	2.53
-7.29	-17.07	-9.15	-7.71	-4.96	38.15	2.72
-7.18	-17.24	-9.42	-7.74	-4.96	37.63	2.93
-6.84	-17.22	-9.18	-7.74	-4.96	37.68	2.8
1.37	-21.45	-12.84	-8.25	-4.96	26.23	8.35
-3.32	-15.81	-9.91	-7.52	-4.96	42.38	3.5
-5.49	-9.83	-5.98	-6.33	-4.96	68.74	0.91
-3.09	-17.23	-10.08	-7.73	-4.95	37.63	3.72
-2.08	-24.78	-11.2	-8.55	-4.95	19.52	5.19
-1.35	-5.47	-5.83	-5.12	-4.95	96.17	0.83
-4.68	-12.87	-8.45	-6.97	-4.94	53.79	2.29
1.76	-22.53	-13.1	-8.35	-4.94	23.83	7.65

-2.99	-6.73	-5.86	-5.49	-4.93	87.17	0.8
-3.72	-11.29	-9.79	-6.64	-4.92	61.03	3.06
-5	-13.24	-7.32	-7.03	-4.92	52.18	1.48
-0.51	-25.09	-10.2	-8.55	-4.92	18.95	4.08
-2.68	-18.07	-13.58	-7.81	-4.92	34.96	10.2
-0.35	-5.49	-6.28	-5.1	-4.92	95.79	0.94
1.47	-24.92	-11.56	-8.53	-4.91	19.22	5.86
-4.99	-11.3	-8.32	-6.62	-4.9	60.87	2.06
-4.92	-12.02	-8.6	-6.77	-4.9	57.46	2.28
-4.94	-13.11	-7.42	-6.99	-4.9	52.63	1.53
-1.57	-24.93	-10.78	-8.52	-4.9	19.19	4.71
-1.33	-5.42	-5.23	-5.07	-4.9	96.16	0.66
-3.95	-9.43	-9.4	-6.19	-4.89	70.51	3.21
-4.76	-12.77	-9.25	-6.92	-4.89	54.05	2.87
-6.7	-14.6	-9.11	-7.27	-4.88	46.57	2.8
-6.44	-14.84	-9.75	-7.3	-4.88	45.64	3.43
1.81	-21.47	-12.53	-8.19	-4.88	26.03	7.53
-3.07	-7.3	-7.04	-5.61	-4.88	83.16	1.42
-6.67	-14.67	-9.3	-7.27	-4.87	46.26	3.01
-4.26	-14.64	-8.57	-7.27	-4.87	46.35	2.26
-5.11	-14.72	-4.89	-7.26	-4.87	46.04	0.62
-5.56	-9.59	-6.11	-6.21	-4.87	69.54	0.86
-0.1	-5.4	-6.45	-5.04	-4.87	96.03	1.13
-2.77	-6.69	-5.9	-5.43	-4.86	87.06	0.85
-1.28	-5.36	-5.49	-5.02	-4.86	96.29	0.77
-1.22	-5.35	-5.94	-5.02	-4.86	96.37	0.94
-3.28	-14.99	-9.75	-7.3	-4.85	44.95	3.21
-1.3	-5.37	-4.94	-5.02	-4.85	96.15	0.62
-2.99	-17.39	-9.96	-7.68	-4.84	36.82	3.72
-4.85	-12.96	-7.44	-6.92	-4.84	53.03	1.64
-3.39	-17.36	-9.2	-7.66	-4.84	36.88	2.41
-3.92	-9.7	-8.72	-6.21	-4.83	68.72	2.44
-3.86	-9.74	-8.47	-6.23	-4.83	68.53	2.32
0.38	-5.7	-6.27	-5.11	-4.83	93.65	0.91
-3.17	-8.03	-10.48	-5.77	-4.82	78.25	4.43
-7	-21.65	-8.28	-8.18	-4.82	25.53	2.18
-3.24	-15.26	-9.34	-7.33	-4.82	43.9	3.13
-2.91	-17.7	-9.6	-7.7	-4.81	35.77	3.33
-3.5	-6.26	-8.63	-5.26	-4.81	89.57	2.57
-6.26	-14.89	-8.94	-7.26	-4.81	45.19	2.7
-0.07	-24.68	-11.5	-8.44	-4.81	19.5	5.64
-4.32	-19.66	-9.2	-7.94	-4.81	30.29	2.99
-6.61	-14.61	-9.18	-7.21	-4.8	46.25	2.92
-5.14	-17.24	-6.08	-7.62	-4.8	37.18	0.99

-6.42	-14.83	-9.65	-7.24	-4.8	45.39	3.35
-5.38	-10.66	-5.94	-6.41	-4.8	63.59	0.93
-4.48	-14.23	-5.5	-7.12	-4.79	47.67	0.78
-6.37	-14.87	-9.33	-7.25	-4.79	45.22	3.09
-2.51	-6.65	-5.93	-5.37	-4.79	86.78	0.9
-6.73	-17.1	-9.49	-7.59	-4.78	37.53	3.28
-2.96	-17.68	-12.63	-7.66	-4.78	35.74	8.51
-5.35	-10.78	-5.97	-6.42	-4.78	62.86	1.05
-1.18	-5.25	-5.9	-4.93	-4.78	96.51	1
-7.21	-14.26	-8.02	-7.12	-4.76	47.42	1.98
-5.01	-17.11	-5.78	-7.57	-4.76	37.45	0.89
-4.27	-11.23	-8.53	-6.5	-4.75	60.51	2.45
-6.19	-14.87	-9	-7.22	-4.75	45.08	2.79
-1.25	-24.86	-10.85	-8.41	-4.75	19.08	4.98
-6.69	-17.38	-8.91	-7.6	-4.74	36.57	2.71
-3.81	-9.64	-9.03	-6.14	-4.74	68.6	2.89
-4.55	-11.84	-8.44	-6.63	-4.74	57.63	2.3
-4.48	-12.02	-9.4	-6.66	-4.74	56.75	3.27
2.32	-21.54	-12.06	-8.09	-4.74	25.59	7.14
-2.91	-7.94	-5.48	-5.7	-4.74	78.3	0.8
-2.81	-17.93	-9.42	-7.68	-4.73	34.9	3.28
-6.29	-14.87	-9.05	-7.21	-4.73	45	2.8
-4.17	-9.83	-6.1	-6.16	-4.73	67.49	1.08
-6.55	-14.59	-9.59	-7.15	-4.72	46	3.36
-3.14	-15.47	-9.14	-7.29	-4.72	42.79	2.98
-0.35	-24.66	-10.4	-8.36	-4.71	19.39	4.29
-3.26	-7.86	-6.71	-5.66	-4.71	78.66	1.16
-4.88	-15.68	-7.51	-7.3	-4.69	41.99	1.82
-6.57	-17.29	-8.4	-7.54	-4.68	36.67	2.4
-4.33	-11.13	-8.17	-6.43	-4.68	60.67	2.11
-4.63	-12.95	-8.12	-6.81	-4.68	52.45	2.18
-6.6	-17.34	-8.74	-7.54	-4.67	36.49	2.64
-3.93	-9.44	-8.92	-6.04	-4.67	69.3	2.71
-4.2	-11.32	-8.63	-6.47	-4.67	59.74	2.66
-7.05	-21.52	-8.17	-8.06	-4.67	25.51	2.13
-6.7	-20.81	-7.54	-7.99	-4.67	27.13	2.04
-0.52	-22.77	-11.7	-8.18	-4.67	22.86	6.11
-4.55	-12.94	-9.02	-6.79	-4.67	52.45	2.95
-1	-24.42	-10.83	-8.32	-4.67	19.75	4.83
-5.3	-11.15	-5.8	-6.43	-4.67	60.53	1.06
-2.8	-7.84	-10.31	-5.61	-4.66	78.4	4.4
-3.78	-9.58	-9.18	-6.07	-4.66	68.51	3.08
-6.09	-14.94	-8.88	-7.16	-4.66	44.5	2.81
-5.05	-9.34	-5.8	-6	-4.66	69.79	0.86

-4.22	-19.8	-8.28	-7.85	-4.66	29.59	2.36
-6.12	-11.67	-7.9	-6.53	-4.65	58.01	2.2
-3	-7.84	-10.48	-5.6	-4.64	78.32	4.58
-2.7	-18.08	-9.59	-7.63	-4.64	34.2	3.62
-4.82	-16.5	-6.69	-7.39	-4.64	39.04	1.28
0.85	-20.16	-12.7	-7.87	-4.64	28.64	7.91
-2.99	-7.01	-7.56	-5.37	-4.64	83.55	1.77
-3.41	-16.85	-12.3	-7.43	-4.63	37.91	7.93
-3.55	-16.81	-12.65	-7.43	-4.63	38.02	8.96
1.09	-5.49	-4.5	-4.9	-4.63	93.66	0.54
-5.11	-15.75	-7.44	-7.26	-4.62	41.48	1.76
-0.94	-7.85	-7.78	-5.58	-4.61	78.06	1.87
-6.9	-21.42	-7.72	-8	-4.61	25.61	1.92
-4.27	-6.36	-4.77	-5.16	-4.61	87.57	0.62
-4.21	-6.31	-4.77	-5.15	-4.61	87.9	0.63
-3.15	-15.16	-9.44	-7.15	-4.6	43.5	3.62
-5.89	-11.47	-6.51	-6.46	-4.6	58.73	1.21
-2.84	-7	-6.32	-5.34	-4.6	83.35	1.22
-4.09	-6.21	-5.3	-5.11	-4.6	88.5	0.79
-3.73	-9.52	-9.21	-6	-4.59	68.42	3.19
3.11	-21.01	-11.58	-7.93	-4.59	26.51	6.48
2.75	-21.21	-11.7	-7.95	-4.59	26.06	6.65
-6.03	-15.02	-8.49	-7.13	-4.59	43.99	2.55
-2.66	-7.8	-9.27	-5.55	-4.58	78.21	3.44
-4.43	-12.65	-9.53	-6.68	-4.58	53.33	3.54
-2.79	-7.88	-6.21	-5.58	-4.58	77.72	1.18
-2.84	-7.85	-5.69	-5.57	-4.58	77.96	0.92
-3.97	-6.16	-5.82	-5.08	-4.58	88.73	1.01
-2.66	-7.07	-6.83	-5.34	-4.57	82.7	1.47
0.3	-5.09	-6.23	-4.74	-4.57	96.13	1.06
-1.14	-5.05	-4.7	-4.73	-4.57	96.47	0.59
-3.68	-9.25	-9.45	-5.92	-4.56	69.76	3.51
-4.18	-19.87	-7.4	-7.77	-4.55	29.16	1.83
-6.19	-11.77	-8.4	-6.48	-4.54	57.06	2.75
-3.71	-9.43	-9.33	-5.94	-4.53	68.6	3.35
-4.14	-11.37	-8.73	-6.37	-4.53	58.86	3.04
-6.8	-21.46	-7.72	-7.95	-4.53	25.37	1.93
0.64	-23.04	-11.45	-8.11	-4.53	22.09	6.64
-2.67	-6.9	-5.58	-5.26	-4.52	83.45	0.88
-3.61	-9.14	-9.12	-5.85	-4.51	70.12	3.31
-2.6	-18.23	-9.37	-7.56	-4.51	33.45	3.57
-2.94	-13.86	-5.37	-6.85	-4.51	48.06	0.75
6.34	-13.51	0.95	-2.36	-4.5	30.5	1.24
-4.47	-17.32	-5.6	-7.42	-4.5	36.1	0.95

-4.1	-9.95	-5.42	-6.04	-4.5	65.75	0.9
3.57	-12.99	2.56	-1.53	-4.49	29.19	1.15
-4.57	-15.45	-6.65	-7.12	-4.49	42.14	1.36
-1.19	-4.95	-5.32	-4.64	-4.49	96.61	0.86
-6.1	-11.47	-7.22	-6.37	-4.48	58.18	1.68
-1.69	-4.97	-7.73	-4.64	-4.47	96.3	2.05
-1.63	-8.84	-8.72	-5.74	-4.46	71.54	2.56
-4.7	-15.26	-6.97	-7.07	-4.46	42.72	1.61
-4.58	-15.92	-7.7	-7.18	-4.46	40.46	2.02
-2.89	-17.7	-8.58	-7.44	-4.46	34.84	2.46
-5.78	-11.09	-6	-6.28	-4.46	59.86	1.1
-4.02	-7.85	-8.17	-5.48	-4.46	77.26	2.42
-1.67	-4.96	-7.16	-4.62	-4.45	96.23	1.66
-5.12	-10.74	-5.29	-6.19	-4.45	61.51	0.84
-5.87	-15.12	-8.64	-7.03	-4.44	43.13	2.82
-5.75	-11.1	-5.7	-6.27	-4.44	59.76	0.99
-2.47	-14.99	-5.7	-6.99	-4.43	43.56	0.96
-5.82	-15.16	-8.59	-7.04	-4.43	42.95	2.88
-1.82	-4.92	-6.3	-4.59	-4.43	96.4	1.23
-1.41	-4.92	-7.8	-4.59	-4.42	96.29	2.17
-4.1	-19.89	-7	-7.69	-4.42	28.84	1.58
-1.63	-4.92	-7.18	-4.58	-4.41	96.21	1.71
-1.89	-4.87	-6.77	-4.56	-4.41	96.55	1.51
-1.31	-4.9	-7.47	-4.57	-4.4	96.3	1.91
-1.68	-4.9	-6.99	-4.57	-4.4	96.3	1.58
-5.06	-16.2	-6.64	-7.17	-4.4	39.34	1.47
1.67	-5.26	-5.53	-4.68	-4.4	93.73	0.9
-1.65	-4.89	-7.73	-4.56	-4.39	96.33	2.15
2.45	-13	2.13	-1.94	-4.39	30.59	0.96
-5.62	-11.07	-4.95	-6.22	-4.39	59.63	0.76
-6.28	-17.43	-7.13	-7.36	-4.38	35.44	1.75
-2.98	-14.51	-9.58	-6.88	-4.38	45.15	3.71
-2.55	-7.6	-9.1	-5.35	-4.37	78.16	3.38
-1.6	-4.87	-6.89	-4.54	-4.37	96.25	1.55
-7.16	-21.67	-7.58	-7.86	-4.37	24.61	1.94
1.06	-23.04	-11.44	-7.99	-4.37	21.83	7.01
-5.11	-10.79	-4.89	-6.14	-4.37	60.9	0.74
-4.04	-9.89	-4.3	-5.93	-4.37	65.43	0.59
-2.74	-6.64	-5.78	-5.08	-4.37	84.15	1
1.65	-22.59	-11.87	-7.94	-4.36	22.68	7.69
1.42	-22.9	-11.84	-7.97	-4.36	22.07	7.71
3.43	-20.48	-11.29	-7.71	-4.36	27.29	6.08
-4.35	-12.68	-8.4	-6.53	-4.36	52.34	2.7
-3.13	-5.74	-8.56	-4.8	-4.36	90.14	2.81

-3.95	-9.53	-5.36	-5.83	-4.36	67.23	0.88
-2.72	-7.84	-5.94	-5.42	-4.36	76.72	1.18
-3.58	-9.03	-9.03	-5.72	-4.35	69.89	3.27
-3.54	-8.95	-9.35	-5.69	-4.35	70.27	3.67
-2.86	-10.99	-9.63	-6.18	-4.35	59.86	3.57
-6.17	-11.62	-7.03	-6.3	-4.34	56.88	1.67
-4.85	-13.89	-8.7	-6.75	-4.34	47.35	3.02
2.92	-11.89	3.9	-0.55	-4.34	29.18	1.2
-2.32	-4.82	-7.71	-4.5	-4.34	96.4	2.43
-4.38	-16.29	-6.14	-7.14	-4.34	38.85	1.21
-1.52	-4.84	-6.75	-4.51	-4.34	96.29	1.5
-1.17	-4.82	-7.25	-4.49	-4.33	96.35	1.79
-5.89	-15.3	-5.01	-6.99	-4.33	42.13	0.65
3.87	-21.13	-11.43	-7.75	-4.33	25.73	7.1
-4.34	-16.48	-6.96	-7.16	-4.33	38.21	1.66
-6.27	-11.7	-7.49	-6.31	-4.32	56.44	2.06
-6.08	-11.52	-7	-6.27	-4.32	57.24	1.59
-4.43	-16.54	-6.42	-7.16	-4.32	38.01	1.35
-4.69	-17.03	-6.82	-7.24	-4.32	36.48	1.66
-4.27	-16.33	-6.91	-7.14	-4.32	38.65	1.7
-6.3	-11.53	-7.37	-6.26	-4.31	57.14	1.87
-1.97	-4.77	-7.74	-4.46	-4.31	96.64	2.19
-3.29	-5.74	-7.53	-4.77	-4.31	89.77	2.01
-3.76	-14.24	-3.93	-6.78	-4.31	45.93	0.5
-3.35	-16.32	-12.27	-7.13	-4.31	38.68	8.8
-3.34	-15.7	-12.09	-7.03	-4.31	40.72	8.53
0.72	-4.8	-4.51	-4.47	-4.31	96.36	0.61
-6.25	-11.63	-7.84	-6.28	-4.3	56.67	2.19
-6.31	-17.46	-7.05	-7.3	-4.3	35.13	1.67
-4.94	-10.06	-5.27	-5.92	-4.3	64.22	0.99
-6.26	-11.71	-8.13	-6.29	-4.29	56.25	2.42
-2.44	-7.32	-8.88	-5.22	-4.29	79.45	3.26
-0.9	-4.77	-9.13	-4.45	-4.29	96.45	3.44
0.49	-12.85	1.55	-1.82	-4.29	30.64	1.35
-3.07	-15.73	-9.15	-7.03	-4.29	40.55	3.56
-5.2	-11.24	-4.4	-6.18	-4.29	58.44	0.58
-5.75	-15.19	-8.39	-6.94	-4.29	42.43	2.86
-5.53	-11.09	-5.64	-6.16	-4.29	59.1	1.06
-5.09	-10.86	-4.73	-6.1	-4.29	60.23	0.66
-4	-9.81	-4.87	-5.86	-4.29	65.42	0.73
-3.94	-9.68	-5.08	-5.82	-4.29	66.08	0.81
-2.57	-7.51	-9.41	-5.26	-4.28	78.15	4.03
-5.9	-20.95	-8.38	-9.27	-4.28	30.64	1.17
-4.25	-12.39	-8.19	-6.41	-4.28	53.22	2.64

-3.42	-8.5	-9.8	-5.52	-4.27	72.37	4.34
-1.11	-4.75	-7.48	-4.43	-4.26	96.4	2.01
-1.71	-4.75	-7.35	-4.42	-4.26	96.35	1.86
-2.03	-14.13	-5.87	-6.73	-4.26	46.16	1.07
-1.19	-4.7	-5.04	-4.41	-4.26	96.74	0.87
-5	-13.47	-8.35	-6.62	-4.25	48.67	2.78
-4.45	-16.78	-6.22	-7.15	-4.25	37.05	1.27
-3.39	-15.73	-12.79	-6.99	-4.25	40.44	10.66
-1.6	-6	-5.35	-4.8	-4.25	87.6	0.87
-6.11	-11.62	-7.39	-6.24	-4.24	56.43	2.13
-2.6	-6.86	-7.64	-5.05	-4.24	81.93	2.2
-5.21	-13.16	-8.14	-6.54	-4.23	49.83	2.73
-1.99	-4.67	-7.64	-4.37	-4.23	96.75	2.18
-2.09	-4.66	-7.53	-4.37	-4.23	96.8	2.15
-3.5	-8.78	-9.67	-5.57	-4.23	70.62	4.28
-3.38	-8.38	-9.69	-5.47	-4.23	72.81	4.26
-4.82	-16.6	-7.17	-7.11	-4.23	37.55	2.01
-2.66	-7.81	-5.06	-5.32	-4.23	76.11	0.9
-3.45	-8.65	-9.73	-5.53	-4.22	71.25	4.31
-5.23	-11.31	-5.15	-6.15	-4.22	57.79	0.91
-2.45	-18.24	-8.57	-7.34	-4.22	32.71	2.71
-4.91	-16.43	-7.56	-7.08	-4.22	38.08	2.15
-3.5	-8.35	-6.12	-5.45	-4.22	72.92	1.7
-2.45	-18.39	-8.7	-7.36	-4.21	32.27	3.21
-5.24	-11.52	-4.98	-6.19	-4.21	56.79	0.82
-5.47	-11.1	-5.59	-6.1	-4.21	58.7	1.06
1.9	-22.61	-11.72	-7.83	-4.2	22.39	7.68
-4.02	-11.84	-9.29	-6.24	-4.2	55.3	3.79
-5.52	-11.09	-6.17	-6.1	-4.2	58.73	1.31
-5.97	-11.3	-6.97	-6.14	-4.19	57.71	1.78
-2.53	-7.37	-9.37	-5.17	-4.19	78.53	3.83
-3.34	-8.25	-9.5	-5.41	-4.19	73.34	4.17
-4.17	-14.59	-9.63	-6.78	-4.19	44.2	3.6
-4.48	-16.85	-6.53	-7.11	-4.18	36.62	1.46
-3.17	-12.09	-6.71	-6.28	-4.18	54.11	1.41
-6.29	-11.81	-6	-6.24	-4.18	55.36	1.28
-3.34	-16.03	-12.7	-6.99	-4.17	39.23	10.58
-1.36	-5.97	-5.51	-4.75	-4.17	87.32	0.99
3.67	-20.6	-10.67	-7.58	-4.16	26.59	5.68
-4.65	-17.18	-7.16	-7.15	-4.16	35.59	1.95
-5.71	-11.16	-4.5	-6.08	-4.15	58.16	0.62
-4.75	-17.16	-7.67	-7.14	-4.15	35.62	2.11
-6.22	-11.65	-7.88	-6.18	-4.14	55.89	2.43
-0.42	-4.6	-9.29	-4.29	-4.14	96.58	3.78

-4.01	-11	-8.03	-6.03	-4.14	58.91	2.65
-3.02	-15.57	-8.65	-6.9	-4.14	40.63	3.17
-4.2	-16.26	-7.29	-7	-4.14	38.38	2.03
-4.11	-16.28	-7.66	-7	-4.14	38.32	2.31
-2.41	-7.16	-9.78	-5.06	-4.13	79.47	4.54
-5.26	-13.22	-8.61	-6.48	-4.13	49.2	3.04
-2.14	-4.55	-8.48	-4.28	-4.13	96.89	3.11
-5.67	-15.22	-8.19	-6.84	-4.13	41.82	2.83
-4.08	-16.46	-7.67	-7.02	-4.13	37.74	2.37
-3.98	-7.59	-8.58	-5.18	-4.13	76.84	2.82
3.46	-12.45	2.05	-1.48	-4.12	30.66	1.47
2.25	-22.69	-11.49	-7.78	-4.12	22.09	7.35
-5.42	-11.08	-5.93	-6.04	-4.12	58.42	1.26
-6.33	-11.51	-7.22	-6.12	-4.11	56.4	1.83
-5.89	-11.42	-6.51	-6.11	-4.11	56.82	1.57
-2.48	-7.18	-10.26	-5.07	-4.11	79.2	5.14
-4.07	-12.45	-9.04	-6.31	-4.11	52.3	3.56
-5.6	-11.11	-4.29	-6.03	-4.09	58.15	0.63
-2.03	-18.76	-9.01	-7.31	-4.09	30.97	3.38
-6.29	-11.71	-6.8	-6.15	-4.08	55.41	1.7
-6.02	-17.76	-6.74	-7.19	-4.08	33.68	1.76
-5.18	-11.42	-4.79	-6.08	-4.08	56.71	0.76
-2.84	-12.95	-7.58	-6.39	-4.08	50.11	2.33
-4.62	-17.21	-7.27	-7.1	-4.08	35.28	2.1
-4.56	-17.18	-7.17	-7.09	-4.08	35.37	2.04
-4.48	-17.1	-6.85	-7.08	-4.08	35.63	1.78
-2.96	-14.42	-8.06	-6.66	-4.07	44.42	2.67
-2.89	-13	-7.47	-6.4	-4.07	49.89	2.18
-3.73	-9.94	-4.23	-5.73	-4.07	63.7	0.65
-3.83	-10.02	-5.43	-5.74	-4.06	63.24	1.04
-4.85	-16.82	-7.05	-7.03	-4.06	36.4	1.87
-4.84	-16.84	-7.72	-7.03	-4.06	36.34	2.26
-2.56	-7.2	-9.14	-5.03	-4.05	78.76	3.79
-2.36	-18.58	-8.87	-7.28	-4.05	31.36	3.67
-3.91	-5.26	-9.09	-4.45	-4.05	91.23	3.65
-1.77	-13.78	-9.99	-6.53	-4.05	46.77	4.6
-5.59	-15.14	-8.25	-6.77	-4.05	41.86	2.8
-3.28	-8.07	-9.42	-5.25	-4.04	73.54	4.25
-5.65	-15.24	-8.29	-6.78	-4.04	41.48	2.91
-5.28	-11.19	-6.53	-6.01	-4.03	57.52	1.68
-3.84	-13.2	-7.8	-6.41	-4.03	48.96	2.22
-5.01	-15.18	-4.14	-6.75	-4.03	41.6	0.56
-3.8	-11.38	-9.86	-6.03	-4.03	56.64	4.99
-3.21	-15.94	-12.38	-6.87	-4.03	39.1	10.11

2.22	-13.19	1.95	-2.13	-4.02	30.68	1.01
-2.5	-6.38	-7.53	-4.77	-4.02	83.65	2.32
-4.48	-17.09	-6.99	-7.04	-4.02	35.49	1.77
-3.82	-5.27	-7.4	-4.42	-4.01	90.97	2.29
-2.69	-11.56	-7.04	-6.07	-4.01	55.75	1.98
-4.48	-9.42	-3.66	-5.57	-4.01	66.06	0.41
-2.93	-7.83	-6.02	-5.17	-4.01	74.76	1.22
-4.69	-11	-4.41	-5.93	-4	58.28	0.77
-2.54	-7.16	-8.99	-4.97	-3.99	78.61	3.75
-4.9	-10.9	-5.47	-5.9	-3.99	58.69	1.08
-3.57	-9.93	-5.05	-5.68	-3.99	63.39	0.95
-4.76	-17.23	-7.42	-7.04	-3.99	34.99	2.31
-4.61	-17.37	-7.45	-7.06	-3.99	34.61	2.25
-3.85	-7.54	-8.14	-5.07	-3.99	76.34	2.73
-2.59	-7.62	-4.24	-5.11	-3.99	75.88	0.66
-2.58	-7.21	-8.9	-4.98	-3.98	78.29	3.85
-0.19	-4.42	-9.21	-4.13	-3.98	96.75	3.95
2.82	-22.83	-10.88	-7.7	-3.98	21.59	7.08
4.19	-20.53	-10.36	-7.44	-3.98	26.39	5.89
-5.49	-15.14	-7.8	-6.72	-3.98	41.61	2.64
-3.68	-9.87	-4.41	-5.66	-3.98	63.61	0.71
-4.48	-17.27	-7.35	-7.04	-3.98	34.85	2.19
-2.55	-7.65	-4.96	-5.1	-3.98	75.61	0.9
-2.46	-4.92	-8.74	-4.28	-3.97	93.06	3.58
-3.23	-7.96	-9.24	-5.18	-3.96	73.75	4.21
-2.11	-10.64	-9.43	-5.83	-3.96	59.8	3.95
-3.51	-16.87	-5.22	-6.96	-3.96	35.97	1.02
-4.89	-10.7	-5.95	-5.84	-3.96	59.47	1.32
-1.14	-4.38	-2.46	-4.1	-3.96	96.92	0.34
-4.11	-9.67	-4.11	-5.59	-3.95	64.46	0.6
1.43	-4.43	-5.2	-4.11	-3.95	96.48	1.02
-3.95	-18.07	-6.39	-7.12	-3.94	32.48	1.59
-3.5	-9.88	-7.07	-5.63	-3.94	63.37	1.97
-2.75	-12.36	-6.87	-6.18	-3.93	51.97	1.84
-6.06	-11.55	-7.06	-6	-3.92	55.44	1.74
-5.25	-13.63	-8.02	-6.41	-3.92	46.85	2.83
-5.93	-17.77	-7.04	-7.07	-3.92	33.25	2.09
-0.97	-8.55	-7.83	-5.3	-3.92	70.24	2.33
-4.58	-17.35	-7.45	-7.01	-3.92	34.47	2.21
-3.59	-15.6	-4.11	-6.73	-3.91	39.85	0.66
4.1	-20.46	-10.81	-7.39	-3.91	26.43	6.33
-2.4	-6.39	-7.66	-4.7	-3.91	82.89	2.48
-5.85	-11.22	-5.97	-5.93	-3.91	56.86	1.24
-3.57	-8.84	-6.12	-5.36	-3.91	68.58	1.34

-2.5	-7.69	-5.38	-5.07	-3.91	75	1.12
1.79	-4.36	-6.03	-4.06	-3.91	96.71	1.39
-2.66	-4.92	-8.87	-4.24	-3.9	92.56	3.76
-2.14	-4.33	-8.9	-4.05	-3.9	96.83	3.89
1.98	-12.95	2.05	-1.95	-3.9	30.71	1.01
-2.69	-4.84	-9.44	-4.21	-3.89	93.11	4.48
-2.72	-4.85	-9.62	-4.21	-3.89	93.06	4.83
-2.6	-4.98	-9.39	-4.25	-3.89	92.14	4.35
-2.51	-4.98	-9.22	-4.26	-3.89	92.14	4.31
-3.7	-5.19	-6.57	-4.31	-3.88	90.6	1.78
-0.42	-7.44	-5.9	-4.97	-3.88	76.31	1.15
21.6	-6.68	15.98	6.38	-3.87	21.53	1.15
-3.48	-5.24	-6.75	-4.32	-3.87	90.16	1.88
-4.51	-10.88	-4.39	-5.81	-3.87	58.25	0.79
-3.82	-18.18	-6.47	-7.08	-3.86	32.01	1.73
-3.77	-5.09	-6.93	-4.26	-3.86	91.18	1.96
-3.91	-7.73	-7.09	-5.05	-3.86	74.5	2.31
-3.51	-9.89	-6.61	-5.57	-3.86	62.93	1.75
-4.35	-13.6	-7.08	-6.36	-3.85	46.75	2.12
-3.67	-5.21	-6.28	-4.3	-3.85	90.3	1.65
1.14	-4.32	-3.49	-4.01	-3.85	96.54	0.46
-5.32	-13.59	-7.39	-6.35	-3.84	46.75	2.28
-4.4	-13.39	-7.1	-6.31	-3.84	47.52	2.1
-4.45	-13.41	-7.76	-6.32	-3.84	47.43	2.66
-3.85	-11.18	-7.78	-5.86	-3.84	56.79	2.78
-2.31	-6.36	-7.6	-4.64	-3.84	82.67	2.53
-0.04	-4.26	-8.81	-3.98	-3.83	96.82	3.62
-3.13	-7.79	-9.13	-5.05	-3.83	73.99	4.16
-4.16	-14.83	-5.08	-6.56	-3.83	42.21	0.95
-2.61	-12.99	-6.67	-6.23	-3.83	49.05	1.9
-4.72	-17.55	-7.91	-6.97	-3.83	33.65	2.57
-2.57	-4.76	-8.76	-4.14	-3.82	93.16	3.69
-2.39	-4.96	-9.07	-4.2	-3.82	91.8	4.09
-3.31	-15	-5.07	-6.57	-3.82	41.57	1.03
3.06	-12.74	2.71	-1.29	-3.81	29.14	1.23
-2.71	-4.81	-9.43	-4.15	-3.81	92.78	4.49
-2.26	-4.46	-7.17	-4.03	-3.81	95.23	2.27
-3.08	-7.72	-9.35	-5	-3.81	74.27	4.51
-4.91	-13.49	-5.88	-6.31	-3.81	47.01	1.37
1.66	-13.15	1.98	-1.62	-3.8	29.09	1.45
2.25	-4.23	-6.66	-3.94	-3.8	96.86	1.8
-3.69	-11.36	-10.06	-5.87	-3.8	55.79	5.72
-4.03	-5.49	-6.72	-4.36	-3.8	88.06	2.06
-3.55	-5.09	-6.05	-4.23	-3.8	90.78	1.43

-5.16	-11.2	-6.97	-5.84	-3.78	56.41	2.13
-4.41	-13.34	-6.85	-6.27	-3.78	47.5	1.98
-3.45	-5.2	-7.13	-4.25	-3.78	89.85	2.23
-3.27	-9.7	-5.85	-5.47	-3.78	63.49	1.4
-2.97	-18.28	-4.21	-6.79	-3.77	30.75	0.86
-5.53	-21.96	-6.6	-7.46	-3.77	22.94	1.93
-2.21	-6.49	-7.03	-4.64	-3.77	81.41	2.17
-3.86	-17.45	-7.24	-6.91	-3.77	33.78	2.5
-5.78	-17.86	-7.35	-6.98	-3.76	32.62	2.5
-3.9	-11.9	-7.98	-5.95	-3.76	53.26	2.91
2.54	-22.73	-10.58	-7.53	-3.76	21.43	6.45
7.51	-14.78	4.85	-0.46	-3.75	21.59	1.41
-2.2	-18.94	-8.48	-7.11	-3.75	29.75	3.64
-3.7	-18.13	-6.22	-6.99	-3.75	31.85	1.63
-4.57	-11.69	-4.29	-5.91	-3.75	54.13	0.74
-1.56	-18.76	-8.63	-7.08	-3.75	30.2	3.4
-2.33	-4.88	-9.48	-4.12	-3.74	91.8	4.79
-3.17	-9.69	-5.8	-5.45	-3.74	63.39	1.42
-3.33	-9.65	-5.11	-5.43	-3.74	63.55	1.06
-3.19	-9.64	-5.6	-5.43	-3.74	63.61	1.28
21.03	-6.69	15.72	6.34	-3.73	21.59	1.14
-2.44	-4.86	-9.39	-4.11	-3.73	91.89	4.55
-3.04	-7.61	-9.39	-4.92	-3.73	74.48	4.7
-2.95	-7.51	-9.5	-4.9	-3.73	75.08	4.98
-3.27	-9.64	-4.93	-5.43	-3.73	63.53	0.99
-3.77	-9.45	-4.02	-5.39	-3.73	64.51	0.63
-3.56	-5	-5.8	-4.16	-3.73	90.89	1.33
24.67	-7.04	14.75	6.01	-3.72	21.71	1.34
-4.68	-13.49	-6.52	-6.25	-3.72	46.71	1.76
-3.6	-18.17	-6.32	-6.98	-3.72	31.67	1.76
-2.81	-13.75	-11.6	-6.29	-3.72	45.71	9.27
-2.51	-10.39	-11.18	-5.6	-3.72	59.88	7.93
2.31	-15.24	1.16	-3.5	-3.71	29.07	0.56
-2.31	-4.24	-7.67	-3.89	-3.71	96.1	2.65
-2.67	-8.32	-5.11	-5.09	-3.71	70.34	1.02
-5.81	-21.58	-7.94	-9.34	-3.7	29.01	0.99
4.57	-20.47	-9.59	-7.24	-3.7	25.97	5.33
-3.41	-5.17	-7.32	-4.18	-3.7	89.55	2.42
-2.47	-6.78	-7.59	-4.66	-3.69	79.1	2.74
-2.26	-4.67	-8.37	-4.02	-3.69	92.9	3.26
4.36	-20.3	-9.92	-7.21	-3.69	26.33	5.16
-2.12	-6.41	-7.32	-4.56	-3.69	81.46	2.45
-3.71	-7.42	-7.79	-4.84	-3.69	75.33	2.66
2.79	-22.44	-10.91	-7.45	-3.68	21.86	7.41

-1.03	-4.05	-2.42	-3.8	-3.67	97.19	0.35
-4.32	-13.9	-6.17	-6.28	-3.66	44.97	1.63
-2.98	-7.52	-9.52	-4.85	-3.66	74.63	5
-2.9	-7.43	-9.71	-4.83	-3.66	75.07	5.51
-3.58	-5.01	-6.18	-4.11	-3.66	90.31	1.66
-4.89	-11.31	-5.43	-5.77	-3.66	55.4	1.27
-5.02	-11.26	-5.73	-5.77	-3.66	55.63	1.4
-2.4	-7.7	-5.48	-4.9	-3.66	73.6	1.26
-3.64	-5.04	-5.79	-4.11	-3.66	90.17	1.42
11.39	-14.43	6.77	-0.2	-3.65	21.72	0.51
-1.09	-4.05	-2.07	-3.79	-3.65	97.11	0
-3.54	-10.2	-9.43	-5.51	-3.65	60.47	5.08
-3.1	-9.64	-5.5	-5.38	-3.65	63.19	1.3
11.32	-14.78	5.66	-0.52	-3.64	21.74	0.93
3.47	-12.82	3.46	-1.31	-3.64	28.95	1.02
4.56	-20.6	-9.17	-7.21	-3.64	25.58	4.78
-3.31	-5.38	-6.39	-4.21	-3.64	87.78	1.75
-2.64	-11.5	-11.16	-5.79	-3.64	54.52	7.98
-2.79	-19.68	-6.68	-7.1	-3.64	27.69	2.05
-2.09	-4.62	-7.58	-3.96	-3.63	92.83	2.76
-2.31	-4.22	-7.84	-3.83	-3.63	95.74	2.84
-0.31	-8.49	-7.76	-5.09	-3.63	69.06	2.66
2.71	-22.47	-10.5	-7.41	-3.63	21.71	6.54
-4.44	-11.61	-3.25	-5.8	-3.63	53.99	0.54
-2.76	-11.37	-5.14	-5.75	-3.63	55.02	0.93
0.58	-4.02	-8.7	-3.76	-3.62	97.09	3.94
0.35	-4.03	-7.92	-3.77	-3.62	96.98	2.91
-3.6	-4.99	-6.23	-4.07	-3.62	90.18	1.69
-4.96	-11.26	-5.78	-5.73	-3.62	55.5	1.44
-4.53	-11.18	-3.71	-5.71	-3.62	55.83	0.62
-2.36	-4.08	-7.95	-3.78	-3.61	96.61	2.85
-5.71	-17.93	-7.5	-6.87	-3.6	32.04	2.7
-2.33	-4.03	-8.21	-3.75	-3.6	96.84	3.46
-2.38	-4.04	-7.94	-3.76	-3.6	96.76	3.12
-2.09	-19.08	-8.12	-7.02	-3.6	29.08	3.39
-3.21	-5.56	-6.77	-4.24	-3.6	86.26	2.1
-5.07	-11.25	-5.7	-5.72	-3.6	55.44	1.38
-2.55	-11.18	-5.36	-5.7	-3.6	55.72	1.28
-1.2	-19.47	-8.7	-7.05	-3.6	28.09	3.79
-2.34	-7.85	-4.33	-4.9	-3.59	72.35	0.89
-4.01	-5.2	-4.78	-4.12	-3.59	88.59	0.98
-3.56	-4.87	-5.84	-4.02	-3.59	90.82	1.42
-4.15	-13.92	-5.9	-6.23	-3.58	44.61	1.6
-4.77	-11.28	-6.17	-5.7	-3.57	55.14	1.75

0.05	-7.64	-6.51	-4.82	-3.57	73.41	1.82
-3.33	-9.56	-4.81	-5.3	-3.57	63.21	0.98
-4.03	-5.22	-5.27	-4.12	-3.57	88.32	1.19
-1.06	-3.94	-2.65	-3.7	-3.57	97.22	0.42
-5.1	-11.35	-6.95	-5.71	-3.56	54.84	2.26
-2.31	-4.06	-7.42	-3.73	-3.56	96.38	2.63
-3.33	-5.2	-7.14	-4.1	-3.56	88.38	2.4
-3.31	-5.26	-6.9	-4.12	-3.56	88.05	2.14
-2.71	-19.62	-7.13	-7.04	-3.56	27.67	2.44
-1.93	-4.57	-8.01	-3.89	-3.55	92.61	3.2
-3.76	-11.81	-6.91	-5.79	-3.55	52.8	2.35
-5.27	-21.99	-6.12	-7.31	-3.55	22.51	1.68
-5.08	-22.01	-6.06	-7.31	-3.55	22.48	1.63
-3.34	-5.15	-7.24	-4.09	-3.55	88.68	2.48
-2.02	-6.31	-7.11	-4.43	-3.55	81.2	2.41
-4.2	-11.8	-3.45	-5.79	-3.55	52.83	0.62
-4.02	-11.57	-3.82	-5.74	-3.55	53.81	0.73
0.66	-15.38	-10.14	-6.45	-3.55	39.49	6.05
-2.39	-7.01	-7.6	-4.63	-3.54	76.89	3.09
-2.19	-4.7	-8.36	-3.93	-3.54	91.61	3.84
5.42	-13.08	1.34	-2.07	-3.54	30.81	1.29
-4.35	-11.69	-3.59	-5.76	-3.54	53.3	0.66
-4.88	-15.13	-7.16	-6.41	-3.54	40.28	2.6
-3.23	-9.8	-8.55	-5.34	-3.53	61.85	3.96
6.14	-13.29	1.67	-2.29	-3.53	30.93	1.1
2.9	-22.54	-10.26	-7.35	-3.53	21.42	6.46
-2.15	-4.25	-6.86	-3.77	-3.52	94.74	2.52
-3.56	-9.56	-5.5	-5.27	-3.52	62.97	1.18
4.14	-12.76	3.84	-1.24	-3.51	28.93	0.74
5.27	-13.1	1.35	-2.13	-3.51	30.96	1.27
-0.74	-19.67	-9.22	-7.01	-3.51	27.44	4.64
-2.4	-7.88	-5.25	-4.85	-3.51	71.72	1.22
-3.48	-18.26	-5.9	-6.83	-3.5	30.92	1.59
-3.37	-18.51	-5.73	-6.87	-3.5	30.29	1.57
-3.46	-10.34	-9.54	-5.44	-3.5	59.15	5.44
-5.1	-11.35	-6.29	-5.67	-3.49	54.57	1.78
-4.71	-11.29	-6.42	-5.65	-3.49	54.77	1.95
-1.41	-6.91	-4.45	-4.56	-3.49	77.2	0.85
-1.05	-3.87	-3	-3.62	-3.49	97.22	0.53
-15.45	-33.84	-20.81	-18.63	-3.48	21.81	1.32
-1.92	-6.29	-7.2	-4.38	-3.48	80.87	2.59
-2.63	-19.34	-6.77	-6.95	-3.48	28.19	2.2
4.56	-19.97	-9.68	-7.02	-3.47	26.66	5.32
-2.9	-12.03	-5.56	-5.8	-3.47	51.57	1.31

-2.91	-9.49	-5.41	-5.22	-3.47	63.09	1.3
-3.17	-9.81	-8.4	-5.29	-3.46	61.48	3.94
-3.82	-18.17	-7.21	-6.79	-3.46	31.07	2.74
-3.48	-14.53	-5.7	-6.24	-3.45	42.04	1.55
6.04	-13.06	1.8	-2.1	-3.45	30.96	0.92
-2.32	-7.77	-3.82	-4.78	-3.45	72.03	0.72
-3.54	-14.58	-6.47	-6.24	-3.43	41.81	2.03
-3.63	-11.87	-6.69	-5.72	-3.43	52.12	2.28
-2.4	-18.72	-6.03	-6.84	-3.43	29.61	1.7
-4.42	-22	-6.2	-7.22	-3.42	22.27	1.82
-1.9	-18.38	-6.29	-6.78	-3.42	30.42	1.94
4.72	-20.36	-9.21	-7.02	-3.41	25.67	4.96
-3.11	-5.59	-5.93	-4.13	-3.41	84.86	1.7
-2.48	-11.78	-5.52	-5.7	-3.41	52.37	1.4
-2.13	-4.71	-7.65	-3.84	-3.4	90.71	3.11
2.97	-22.73	-9.9	-7.27	-3.4	20.85	6.4
-2.79	-5.81	-7.33	-4.18	-3.4	83.44	2.85
-2.47	-9.27	-7.34	-5.12	-3.39	63.82	2.39
-1.71	-4.41	-8.15	-3.73	-3.39	92.63	3.52
-3.69	-11.97	-7.33	-5.72	-3.39	51.51	2.81
-0.02	-19.7	-9.27	-6.94	-3.39	27.15	5.11
-1.63	-5.93	-7.01	-4.21	-3.39	82.58	2.32
-3.09	-9.83	-8.22	-5.24	-3.38	61.02	3.81
-2.16	-4.64	-7.77	-3.8	-3.38	91	3.17
-2.41	-12.89	-4.5	-5.9	-3.38	47.79	0.98
-1.94	-19.29	-8.08	-6.89	-3.37	28.05	3.59
2.92	-12.89	2.97	-1.34	-3.36	28.85	1.04
-2.92	-18.94	-4.04	-6.81	-3.36	28.9	0.76
-1.34	-10.65	-8.55	-5.42	-3.36	57.1	3.66
-1.91	-13.67	-5.18	-6.04	-3.36	44.82	1.33
-1.8	-6.2	-6.78	-4.27	-3.36	80.71	2.41
-4.06	-11.39	-3.5	-5.58	-3.36	53.81	0.62
-3.75	-7.5	-5.45	-4.64	-3.36	73	1.43
-3.03	-5.62	-5.36	-4.09	-3.36	84.36	1.38
-3.08	-5.58	-5.52	-4.09	-3.36	84.63	1.48
-2.19	-6.93	-7.35	-4.48	-3.35	76.29	2.86
-2.78	-7.27	-9.24	-4.58	-3.35	74.33	5.25
-3.38	-10.08	-9.11	-5.28	-3.35	59.71	5.08
-4.82	-15.16	-7.25	-6.28	-3.35	39.61	2.8
-3.36	-17.74	-6.2	-6.66	-3.35	31.98	1.88
-1.83	-18.34	-6.25	-6.73	-3.35	30.37	1.96
-3.73	-14.46	-6.59	-6.16	-3.34	41.97	2.23
-2.96	-5.65	-5.82	-4.09	-3.34	84.01	1.68
-2.36	-8.43	-5.33	-4.88	-3.34	67.9	1.42

-1.57	-9.41	-7.1	-5.11	-3.34	62.9	2.57
-3.69	-7.56	-5.54	-4.65	-3.34	72.65	1.52
-3.96	-14.06	-6.54	-6.09	-3.33	43.3	2.18
-3.77	-14.31	-6.8	-6.12	-3.33	42.43	2.26
-0.33	-19.5	-9.47	-6.87	-3.33	27.48	5.18
-1.22	-10.03	-7.57	-5.25	-3.33	59.82	3.07
-1.69	-18.92	-7.04	-6.79	-3.33	28.87	2.55
-2.09	-4.65	-7.76	-3.77	-3.32	90.52	3.31
-0.22	-5.19	-5.94	-3.94	-3.32	86.94	1.56
-2.84	-5.65	-7	-4.08	-3.32	83.9	2.48
-4.28	-11.67	-3.14	-5.61	-3.32	52.5	0.56
1.55	-16.4	-9.74	-6.45	-3.32	35.67	5.89
-1.67	-18.32	-6.42	-6.71	-3.32	30.37	2.06
-4	-22.09	-6.82	-7.15	-3.31	21.92	2.52
-4.42	-14.97	-7.14	-6.22	-3.31	40.11	2.82
-1.69	-19.12	-6.8	-6.81	-3.31	28.36	2.35
-1.68	-18.84	-6.79	-6.77	-3.31	29.02	2.41
-3.05	-9.83	-8.27	-5.19	-3.3	60.63	3.99
-4.12	-12.76	-5.24	-5.82	-3.3	48.04	1.34
-3.41	-14.54	-5.78	-6.13	-3.29	41.51	1.69
-2.24	-15.89	-5.01	-6.36	-3.29	37.15	1.25
-2.3	-15.87	-5.18	-6.36	-3.29	37.21	1.31
-2.94	-5.64	-6.52	-4.06	-3.29	83.81	2.17
-2.97	-9.18	-4.64	-5.02	-3.29	63.76	1.09
-2.73	-7.29	-9.26	-4.53	-3.28	73.81	5.44
-3.32	-14.94	-4.96	-6.19	-3.28	40.14	1.06
-2.9	-5.62	-7.01	-4.05	-3.28	83.92	2.55
-1.8	-18.88	-5.96	-6.75	-3.28	28.85	1.8
2.83	-3.67	-5.32	-3.42	-3.28	97.15	1.27
-3.69	-14.18	-6.05	-6.06	-3.27	42.67	1.86
-3.27	-18.69	-5.55	-6.73	-3.27	29.33	1.55
-3.29	-10.1	-9.36	-5.23	-3.27	59.23	5.59
-1.69	-6.97	-10.95	-4.43	-3.27	75.6	8.85
-1.67	-18.81	-6.37	-6.74	-3.27	29.03	2.07
-3.56	-7.49	-5.57	-4.58	-3.27	72.65	1.66
-3.47	-11.89	-5.8	-5.61	-3.26	51.33	1.66
-2.72	-5.76	-7.35	-4.07	-3.26	82.86	3.03
-1.49	-5.54	-10.98	-4.01	-3.26	84.28	8.98
-3.67	-7.48	-5.7	-4.57	-3.26	72.62	1.66
-2.04	-4.62	-7.19	-3.71	-3.25	90.26	2.81
-3.23	-17.25	-6	-6.52	-3.25	33.07	1.96
-3.58	-7.43	-6.06	-4.55	-3.25	72.84	1.86
-4.52	-11.45	-6.68	-5.52	-3.24	53.11	2.45
-4.49	-11.64	-6.8	-5.56	-3.24	52.32	2.47

4	-13.05	3.03	-1.47	-3.24	28.84	1.03
-1.63	-18.97	-7.02	-6.74	-3.24	28.55	2.63
-1.63	-18.85	-7.3	-6.72	-3.24	28.85	2.95
-2.08	-6.74	-7.39	-4.35	-3.23	76.73	3.07
-2.95	-5.55	-6.54	-3.99	-3.23	84.04	2.19
-4.58	-15.37	-6.73	-6.23	-3.23	38.6	2.38
-3.55	-17.84	-6.7	-6.59	-3.23	31.4	2.28
-2.28	-6.86	-7.58	-4.37	-3.22	75.94	3.15
1.09	-3.58	-8.18	-3.35	-3.22	97.36	3.86
-2.19	-15.97	-4.86	-6.32	-3.22	36.69	1.18
-1.7	-19.32	-7.65	-6.78	-3.22	27.68	3.4
-3.46	-14.61	-5.1	-6.09	-3.22	41.05	1.38
-3.42	-14.52	-5.19	-6.08	-3.22	41.37	1.36
-4.53	-15.29	-7.03	-6.21	-3.22	38.83	2.63
-3.38	-18.06	-6.53	-6.61	-3.22	30.8	2.16
-2.97	-9.79	-8.67	-5.12	-3.21	60.43	4.56
-3.79	-11.49	-4.29	-5.5	-3.21	52.83	1.02
-3.31	-11.44	-4.65	-5.48	-3.21	53.03	1.2
-0.8	-9.96	-7.8	-5.15	-3.21	59.65	3.37
0.72	-7.66	-5.67	-4.57	-3.2	71.31	1.51
-3.64	-14.59	-5.82	-6.09	-3.2	41.08	1.66
-2.63	-7.26	-9.2	-4.47	-3.2	73.53	5.65
-2.15	-15.28	-4.61	-6.2	-3.2	38.81	1.17
-3.4	-12.08	-4.87	-5.61	-3.2	50.35	1.2
-2.97	-9.12	-5.14	-4.95	-3.2	63.65	1.35
-3.59	-18.02	-6.56	-6.59	-3.2	30.86	2.26
-3.66	-14.51	-6.25	-6.06	-3.19	41.32	2.04
-3.55	-12.23	-5.77	-5.63	-3.19	49.7	1.74
-3.17	-18.78	-5.69	-6.68	-3.19	28.92	1.76
-3.2	-10.02	-9.32	-5.16	-3.19	59.24	5.76
-4.68	-15.18	-7.21	-6.17	-3.19	39.07	2.86
-3.29	-6.97	-6.62	-4.38	-3.19	75.14	2.21
-3.76	-7.48	-5.68	-4.52	-3.19	72.23	1.71
-2.88	-10.01	-8.44	-5.15	-3.18	59.25	4.51
4.69	-19.89	-8.3	-6.81	-3.18	26.29	4.09
-2.6	-12.64	-6.01	-5.71	-3.18	48.08	2.02
-4.68	-15.27	-6.8	-6.18	-3.18	38.78	2.45
-3.74	-7.39	-5.91	-4.49	-3.18	72.7	1.71
-2.93	-9.15	-6.14	-4.93	-3.18	63.43	1.97
-3.72	-13.97	-6.13	-5.96	-3.17	43.12	1.97
4.52	-20.05	-8.46	-6.82	-3.17	25.9	4.45
-2.11	-6.5	-6.49	-4.23	-3.17	77.77	2.24
1.31	-19.11	-8.91	-6.72	-3.17	28.07	5.18
-2.52	-9.23	-4.66	-4.96	-3.17	62.97	1.01

-2.89	-9.07	-6.37	-4.91	-3.17	63.73	2.14
-3.64	-14.07	-6.97	-5.97	-3.16	42.72	2.52
-1.27	-4.2	-8.24	-3.51	-3.16	92.51	3.92
-2.16	-6.5	-7.16	-4.22	-3.16	77.72	2.91
-3.68	-18.11	-6.66	-6.57	-3.16	30.54	2.39
-1.6	-19.05	-7.13	-6.69	-3.16	28.2	2.9
-3.98	-17.14	-6.79	-6.47	-3.15	33.14	2.52
4.3	-11.22	4.79	0.16	-3.15	28.78	1.32
-8.05	-25.05	-12.9	-12.59	-3.15	28.79	1.27
-1.8	-15.9	-5.49	-6.26	-3.15	36.73	1.63
-4.33	-15.4	-7.13	-6.18	-3.15	38.26	2.91
-3.59	-7.4	-5.8	-4.48	-3.15	72.49	1.72
-2.78	-8.91	-7.25	-4.86	-3.15	64.48	3.02
-4.28	-17.05	-7.85	-6.45	-3.14	33.35	3.68
-2.91	-13.14	-6.55	-5.78	-3.14	46.01	1.99
-3.11	-15.52	-5.17	-6.19	-3.14	37.88	1.59
-2.01	-6.62	-7.5	-4.25	-3.14	76.89	3.15
-3.63	-7.43	-5.8	-4.48	-3.14	72.27	1.79
-3.17	-15.26	-5.88	-6.15	-3.13	38.67	1.8
-4.26	-15.51	-6.92	-6.18	-3.13	37.88	2.75
-2.78	-8.86	-6.83	-4.83	-3.13	64.65	2.58
-2.63	-5.74	-6.65	-3.98	-3.12	82.13	2.49
-1.17	-4.56	-10.59	-3.61	-3.12	89.75	8.77
2.95	-3.91	-3.48	-3.39	-3.12	94.28	0.6
-0.99	-3.46	-1.73	-3.24	-3.12	97.48	0.37
-4.11	-17.1	-7.44	-6.43	-3.11	33.12	3.09
-3.51	-12.16	-4.65	-5.56	-3.11	49.66	1.13
-2.68	-12.45	-5.45	-5.63	-3.11	48.58	1.64
-3.77	-7.45	-5.2	-4.46	-3.11	71.98	1.45
-3.2	-6.95	-6.65	-4.33	-3.11	74.86	2.34
-1.91	-4.5	-6.74	-3.58	-3.1	90.06	2.53
2.97	-17.64	-9.19	-6.48	-3.1	31.65	5.41
-3.89	-17.07	-6.36	-6.41	-3.09	33.15	2.33
-1.85	-4.52	-7.5	-3.58	-3.09	89.87	3.31
-0.49	-7.46	-5.7	-4.45	-3.09	71.85	1.59
-3.74	-11.92	-3.43	-5.5	-3.09	50.6	0.76
0.51	-19.25	-9.12	-6.67	-3.09	27.57	5.19
-4.19	-12.84	-6.09	-5.69	-3.08	46.96	1.9
-1	-3.45	-1.37	-3.21	-3.08	97.3	0
-1.76	-6.39	-7.48	-4.14	-3.08	77.91	3.33
-4.42	-16.69	-7.54	-6.35	-3.07	34.19	3.3
-4.25	-17.13	-7.44	-6.41	-3.07	32.94	3.35
-4.03	-17.15	-6.78	-6.41	-3.07	32.91	2.51
-2.1	-6.42	-7.37	-4.15	-3.07	77.66	3.24

-3.52	-9.71	-3.99	-5	-3.07	60.18	0.78
-2.08	-11.66	-5.95	-5.44	-3.06	51.51	1.77
-2.01	-6.5	-6.6	-4.17	-3.06	77.18	2.5
-4.37	-16.77	-7.77	-6.35	-3.06	33.95	3.8
-1.56	-19.49	-7.61	-6.69	-3.06	26.97	3.44
-3.83	-11.74	-4.79	-5.44	-3.06	51.23	1.2
-3.53	-7.42	-5.45	-4.42	-3.06	71.86	1.66
-2.76	-8.63	-7.46	-4.73	-3.06	65.46	3.31
-3.65	-10.78	-7.71	-5.24	-3.06	55.27	3.55
-3.38	-14.59	-5.34	-5.98	-3.05	40.64	1.67
-1.57	-6.08	-5.32	-4.03	-3.05	79.57	1.55
3.13	-3.4	-5.21	-3.18	-3.05	97.42	1.45
-1.58	-4.53	-7.35	-3.55	-3.04	89.5	3.21
-2.57	-7.26	-9.11	-4.37	-3.04	72.68	5.71
-4.07	-11.29	-5.22	-5.33	-3.04	53	1.36
-0.07	-9.17	-8.15	-4.85	-3.04	62.67	4.07
-2.99	-11.43	-5.44	-5.36	-3.03	52.36	1.64
-2.2	-8.19	-5.72	-4.61	-3.03	67.59	1.96
-1.47	-6.15	-5.44	-4.04	-3.03	79.06	1.67
3.68	-18.68	-9.11	-6.56	-3.03	28.81	5.62
-1.64	-16.1	-5.76	-6.21	-3.03	35.8	1.87
-4.22	-12.42	-5.96	-5.56	-3.02	48.37	1.98
-4.22	-12.58	-6.27	-5.59	-3.02	47.71	2.15
-1.51	-4.54	-7.02	-3.54	-3.02	89.26	2.94
-1.66	-6.48	-6.88	-4.13	-3.02	77	2.98
-1.44	-16.04	-5.69	-6.19	-3.02	35.95	1.88
-4.42	-11.76	-6.66	-5.43	-3.01	50.95	2.67
-1.61	-4.46	-7.79	-3.5	-3.01	89.68	3.73
1.36	-3.35	-8.2	-3.13	-3.01	97.49	4.06
-3.88	-14.36	-8.44	-5.92	-3.01	41.26	4.25
-3.37	-6.32	-5.54	-4.08	-3.01	77.95	1.71
22.12	-5.31	12.23	5.24	-3	28.77	1.45
-3.55	-22.07	-6.73	-6.93	-3	21.46	2.79
-3.97	-14.68	-6.49	-5.96	-3	40.15	2.46
1.25	-7.61	-5.05	-4.43	-3	70.51	1.35
-1.29	-18.99	-7.35	-6.57	-3	28	3.38
-2.17	-6.39	-6.9	-4.08	-2.99	77.41	2.7
-2.05	-6.38	-7.38	-4.08	-2.99	77.47	3.2
-4.15	-15.48	-6.77	-6.09	-2.99	37.57	2.67
-2.54	-5.73	-4.54	-3.88	-2.99	81.38	1.27
-1.92	-6.29	-7.18	-4.05	-2.98	77.96	3.12
-4.43	-16.7	-7.79	-6.29	-2.98	33.93	3.7
-4.31	-16.91	-8.01	-6.32	-2.98	33.34	3.95
-4.16	-17.17	-7.65	-6.35	-2.98	32.62	3.47

1.95	-13.22	1.91	-2.24	-2.98	30.97	0.95
4.53	-19.88	-8.66	-6.67	-2.98	25.92	4.66
-3.81	-12.31	-4.26	-5.51	-2.98	48.6	1.17
-2.54	-12.96	-5.05	-5.64	-2.98	46.12	1.7
-2.16	-3.81	-2.16	-3.26	-2.98	94.02	0.45
-3.14	-15.56	-6.01	-6.08	-2.97	37.28	2.06
-7.53	-24.56	-10.09	-12.11	-2.97	28.76	0.57
-1.73	-4.42	-7.41	-3.46	-2.97	89.74	3.38
4.34	-19.88	-7.27	-6.66	-2.97	25.89	3.31
-2.94	-6.87	-6.76	-4.21	-2.97	74.49	2.62
-3.07	-18.79	-5.18	-6.53	-2.96	28.4	1.55
-3.04	-9.88	-9.16	-4.97	-2.96	58.91	5.85
-2.04	-6.46	-6.17	-4.08	-2.96	76.77	2.1
0.22	-8.83	-7.64	-4.72	-2.96	64	3.53
-3.41	-7.53	-4.57	-4.38	-2.96	70.79	1.22
-1.46	-4.5	-6.92	-3.48	-2.95	89.03	2.97
-0.18	-14.35	-0.29	-3.29	-2.95	31	1.28
-2.65	-12.35	-5.61	-5.5	-2.95	48.35	1.71
-1.54	-16.17	-5.84	-6.16	-2.95	35.36	1.89
-3.42	-7.48	-5.21	-4.36	-2.95	71.01	1.54
-3.33	-7.52	-5.85	-4.37	-2.95	70.74	1.99
-1.69	-4.4	-7.21	-3.44	-2.94	89.61	3.18
-1.08	-6.42	-6.14	-4.06	-2.94	76.91	2.23
-1.7	-15.5	-5.38	-6.06	-2.94	37.37	1.62
3.14	-22.18	-9.09	-6.89	-2.93	21.13	5.06
-1.94	-6.47	-7.14	-4.06	-2.93	76.59	2.92
-1.4	-6.17	-6.22	-3.98	-2.93	78.38	2.26
4.44	-18.45	-9.87	-6.47	-2.93	29.18	7.16
-2.87	-12.22	-6.25	-5.47	-2.93	48.78	2.29
-2.82	-12.27	-6.5	-5.48	-2.93	48.58	2.56
-2.8	-11.98	-6.47	-5.42	-2.93	49.75	2.64
-1.82	-6.17	-7.46	-3.97	-2.92	78.3	3.5
-3.79	-17.02	-6.57	-6.29	-2.92	32.9	2.66
-0.85	-3.98	-8.29	-3.29	-2.92	92.34	4.36
-1.26	-19.69	-7.15	-6.61	-2.92	26.23	3.2
-3.82	-15.05	-6.56	-5.97	-2.92	38.73	2.66
-1.21	-19.17	-7.44	-6.54	-2.92	27.41	3.58
-3.61	-10.77	-7.72	-5.14	-2.92	54.71	3.63
-3.63	-10.11	-7.27	-4.99	-2.91	57.64	3.36
-2.71	-12.31	-4.56	-5.46	-2.9	48.36	1.29
-2.8	-12.12	-6.78	-5.43	-2.9	49.07	2.81
-3.25	-14.89	-5.25	-5.92	-2.89	39.17	1.63
-8.16	-25.06	-13.36	-12.59	-2.89	28.76	1.42
-2.46	-7.47	-8.95	-4.32	-2.89	70.76	5.75

-3.21	-11.78	-4.8	-5.33	-2.89	50.38	1.55
-3.2	-14.95	-5.1	-5.93	-2.89	38.97	1.5
21.66	2.94	23.49	12.04	-2.88	28.75	1.41
-2.95	-9.69	-8.82	-4.87	-2.88	59.45	5.54
-3.55	-11.89	-3.7	-5.35	-2.88	49.9	0.91
-3.41	-7.35	-5.14	-4.28	-2.88	71.33	1.53
-3.25	-7.56	-5.73	-4.34	-2.88	70.14	1.98
-2.75	-10.06	-6.49	-4.95	-2.87	57.7	2.72
2.79	-22.76	-8.64	-6.91	-2.87	20.02	5.48
-0.95	-6.43	-6.03	-4.01	-2.87	76.47	2.26
-2.56	-12.87	-6.07	-5.55	-2.87	46.11	2.14
-3.29	-6.18	-5.6	-3.94	-2.87	77.96	1.79
-3.12	-18.08	-6.76	-6.37	-2.87	29.97	2.82
-3.39	-7.43	-5.42	-4.29	-2.87	70.84	1.69
-3.63	-17.19	-6.21	-6.27	-2.86	32.25	2.4
-3.5	-17.07	-6.15	-6.25	-2.86	32.58	2.28
3.12	-22.61	-9.01	-6.88	-2.86	20.26	6.07
-2.82	-19	-5.38	-6.48	-2.86	27.68	1.8
-1.27	-6.2	-6.27	-3.94	-2.86	77.77	2.38
-3.36	-6.36	-6.13	-3.99	-2.86	76.85	2.32
3.21	-18.96	-5.61	-6.46	-2.86	27.78	1.91
-3.31	-7.48	-6.21	-4.3	-2.86	70.52	2.28
-2.87	-19.03	-4.46	-6.82	-2.85	28.66	0.94
-1.19	-6.28	-6.44	-3.96	-2.85	77.26	2.46
-3.07	-18.3	-6.58	-6.38	-2.85	29.37	2.77
-1.09	-19.29	-7.13	-6.5	-2.85	26.99	3.27
-1.73	-6.01	-7.14	-3.87	-2.84	78.81	3.25
-2.6	-8.73	-7.07	-4.6	-2.84	63.86	3.25
-3.58	-9.86	-7.14	-4.88	-2.84	58.49	3.32
-4.44	-16.69	-7.88	-6.18	-2.83	33.57	3.98
-0.99	-3.18	-1.52	-2.96	-2.83	97.46	0
-3.45	-14.7	-4.85	-5.84	-2.83	39.59	1.44
4.01	-19.71	-6.96	-6.54	-2.83	26	3.06
-3.07	-11.72	-6.29	-5.27	-2.82	50.37	2.64
-3.11	-15.63	-5.18	-5.99	-2.82	36.65	1.55
-2.4	-7.5	-8.82	-4.28	-2.82	70.15	5.69
-0.49	-6.04	-5	-3.88	-2.82	78.49	1.58
-3.39	-14.67	-4.9	-5.83	-2.82	39.68	1.34
-2.63	-12.14	-5.11	-5.37	-2.82	48.72	1.55
-1.58	-5.91	-7.24	-3.82	-2.81	79.2	3.51
-3.34	-14.85	-4.45	-5.85	-2.81	39.04	1.21
-3.48	-11.85	-4.26	-5.3	-2.81	49.83	1.22
-2.71	-12.44	-5.12	-5.42	-2.81	47.49	1.6
-1.05	-4.24	-10.2	-3.3	-2.81	89.85	8.49

-2.84	-11.02	-5.32	-5.11	-2.8	53.19	1.87
3.66	-13.23	2.12	-1.57	-2.8	28.64	1.46
-3.13	-15.61	-4.85	-5.98	-2.8	36.66	1.42
4.48	-19.95	-8.08	-6.55	-2.8	25.42	4.03
-3.21	-6.08	-4.96	-3.87	-2.8	78.14	1.51
-3.45	-17.05	-5.68	-6.2	-2.79	32.5	2.02
-1.41	-4.37	-7.08	-3.33	-2.79	88.91	3.25
-1.57	-6.68	-6.55	-4.03	-2.79	74.56	2.78
-0.97	-3.94	-10.03	-3.19	-2.79	91.79	8.25
-3.37	-8.85	-3.25	-4.61	-2.79	63.05	1.06
-3.03	-13.93	-5.91	-5.7	-2.79	42.05	2.17
-2.78	-11.92	-6.14	-5.31	-2.79	49.46	2.49
-4.27	-12.12	-6.26	-5.34	-2.78	48.64	2.39
-3.57	-17.21	-6.17	-6.21	-2.78	32.02	2.37
-3.56	-17.17	-5.67	-6.21	-2.78	32.14	1.94
-2.93	-18.39	-5.6	-6.35	-2.78	28.99	1.99
-0.73	-5.41	-6.13	-3.65	-2.78	82.07	2.19
-1.36	-4.39	-6.67	-3.33	-2.77	88.67	2.89
-2.17	-3.53	-6.26	-3.04	-2.77	94.45	2.36
-1.1	-19.65	-6.74	-6.51	-2.77	26.03	2.95
-3.12	-22.19	-7.43	-6.78	-2.77	20.87	3.8
3	-22.51	-9.54	-6.81	-2.77	20.3	6.76
-1.57	-6.49	-7.79	-3.96	-2.77	75.54	4.06
-3.26	-6.48	-5.87	-3.96	-2.77	75.65	2.11
-1	-19.36	-6.99	-6.46	-2.77	26.67	3.11
-2.54	-8.62	-6.66	-4.53	-2.77	64.1	2.89
-3.5	-9.65	-6.94	-4.79	-2.77	59.14	3.18
-4.49	-16.67	-7.44	-6.13	-2.76	33.46	3.67
-2.55	-12.03	-4.78	-5.31	-2.76	48.93	1.41
-3.41	-9.55	-4.24	-4.76	-2.76	59.61	0.94
-0.93	-19.07	-7.49	-6.42	-2.76	27.33	3.73
-0.89	-19.32	-7.9	-6.45	-2.76	26.73	4.33
-3.08	-15.68	-4.53	-5.95	-2.75	36.31	1.35
-3.83	-14.24	-7.7	-5.72	-2.75	40.86	3.81
-3.42	-14.82	-4.81	-5.81	-2.75	38.99	1.49
-2.69	-12.54	-5.44	-5.4	-2.75	46.93	1.69
5.07	-17.72	-9.06	-6.24	-2.75	30.62	5.69
-3.12	-5.92	-5.53	-3.78	-2.75	78.81	1.88
-0.83	-19.22	-8.33	-6.43	-2.75	26.97	5
-4.52	-16.65	-7.21	-6.11	-2.74	33.46	3.45
-2.98	-11.6	-6.24	-5.19	-2.73	50.55	2.39
-0.95	-3.08	-1.06	-2.86	-2.73	97.44	0
-2.87	-9.57	-9.07	-4.74	-2.73	59.37	6.24
-2.9	-10.87	-4.77	-5.03	-2.73	53.54	1.43

-1.93	-6.34	-6.6	-3.9	-2.73	76.21	2.67
-1.97	-15.3	-5.31	-5.88	-2.73	37.43	1.75
2	-18.85	-7.98	-6.37	-2.73	27.79	4.32
-2.41	-8.58	-2.47	-4.51	-2.73	64.11	0.56
-3.41	-14.77	-4.14	-5.78	-2.72	39.05	1.14
-2.8	-9.05	-8.42	-4.61	-2.72	61.77	5.44
-1.33	-16.24	-5.74	-6.02	-2.72	34.56	2.09
-3.3	-6.47	-5.49	-3.92	-2.72	75.39	1.93
-3.2	-6.59	-5.25	-3.96	-2.72	74.71	1.74
-3.11	-6.29	-5.36	-3.87	-2.72	76.42	1.84
-3.13	-6.43	-5.57	-3.91	-2.72	75.64	1.98
-3.13	-6.35	-5.52	-3.89	-2.72	76.07	1.99
1.74	-7.58	-4.25	-4.23	-2.72	69.25	1.14
-3.12	-11.98	-5.64	-5.25	-2.71	48.94	2.03
-3.35	-17.1	-5.89	-6.15	-2.71	32.15	2.37
3.04	-11.99	4.5	-0.47	-2.71	28.61	1.04
-1.31	-4.37	-7	-3.28	-2.71	88.36	3.36
2.18	-3.01	-7.98	-2.82	-2.71	97.81	4.34
0.65	-4.6	-5.36	-3.35	-2.71	86.79	1.58
-3.12	-6.27	-5.68	-3.86	-2.71	76.51	2.08
-3.16	-8.5	-5.32	-4.47	-2.71	64.44	1.8
-3.01	-13.9	-5.66	-5.64	-2.71	41.91	2.04
-3	-13.96	-5.81	-5.65	-2.71	41.71	2.16
-2.99	-13.93	-5.97	-5.65	-2.71	41.8	2.39
-0.48	-5.84	-3.48	-3.75	-2.71	79.04	0.82
-2.1	-3.55	-3.23	-3.01	-2.71	93.93	0.79
0.27	-7.75	-5.34	-4.27	-2.7	68.24	1.63
-0.86	-3.63	-9.16	-3.02	-2.7	93.3	6.63
-3.33	-6.41	-5.67	-3.9	-2.7	75.67	2.03
-3.16	-6.36	-5.62	-3.88	-2.7	75.94	2
-4.51	-16.52	-7.13	-6.06	-2.69	33.7	3.41
-2.87	-12.5	-6.61	-5.36	-2.69	46.88	2.6
-4.52	-16.47	-7.24	-6.05	-2.68	33.82	3.61
-3.71	-17.23	-6.75	-6.15	-2.68	31.73	3.07
-3.72	-17.31	-6.63	-6.16	-2.68	31.52	2.74
-0.8	-6.37	-6.3	-3.87	-2.68	75.78	2.7
-3.04	-5.85	-5.28	-3.72	-2.68	78.83	1.76
-2.5	-8.53	-6.57	-4.45	-2.68	64.14	2.8
-2.5	-8.32	-6.73	-4.4	-2.68	65.19	2.99
-2.49	-8.2	-6.95	-4.37	-2.68	65.82	3.28
-4.65	-16.43	-7.5	-6.04	-2.67	33.91	4.07
-3.05	-15.62	-4.61	-5.88	-2.67	36.29	1.43
-0.86	-3.62	-8.98	-3	-2.67	93.16	6.35
-0.89	-3.68	-9.15	-3.02	-2.67	92.75	6.59

0.41	-8.06	-4.43	-4.34	-2.67	66.47	1.07
-2.08	-7.98	-4.29	-4.32	-2.67	66.91	1.34
-0.9	-3.76	-9.68	-3.05	-2.66	92.11	7.74
-3.05	-6.44	-5.55	-3.88	-2.66	75.27	1.92
-3.14	-7.61	-4.76	-4.21	-2.66	68.8	1.53
-1.26	-6.28	-2.72	-3.83	-2.66	76.2	0.67
-0.84	-3.1	-1.93	-2.81	-2.66	96.73	0.52
-3.01	-15.6	-5.52	-5.86	-2.65	36.29	2.01
-3.45	-11.59	-5.01	-5.13	-2.65	50.25	1.76
-3.44	-7.24	-4.74	-4.1	-2.65	70.73	1.48
-2.64	-11.52	-5.32	-5.14	-2.65	50.56	2.04
-0.05	-5.72	-4.79	-3.66	-2.64	79.38	1.56
-2.46	-12.16	-4.59	-5.25	-2.64	47.98	1.33
-1.26	-16.42	-5.26	-5.99	-2.64	33.86	1.78
-0.9	-3.8	-9.84	-3.05	-2.64	91.76	8.2
-3.08	-14.17	-6.07	-5.64	-2.64	40.79	2.35
-2.88	-13.23	-5.63	-5.47	-2.64	44.01	2.19
-2.72	-11.45	-5.32	-5.11	-2.64	50.79	1.92
-2.51	-11.76	-5.69	-5.18	-2.64	49.54	2.14
-2.72	-9.98	-6.46	-4.77	-2.63	57.03	2.83
-3.26	-6.41	-5.44	-3.85	-2.63	75.28	1.85
-3.11	-6.43	-5.91	-3.85	-2.63	75.09	2.24
-3	-18.52	-5.76	-6.26	-2.63	28.37	2.04
-2.78	-18.54	-5.68	-6.26	-2.63	28.31	2.22
-1.39	-6.25	-4.12	-3.8	-2.63	76.18	1.17
-3.41	-10.86	-7.25	-4.96	-2.63	53.19	3.48
-3.39	-9.4	-6.19	-4.63	-2.63	59.69	2.57
-3.07	-11.82	-6.46	-5.15	-2.62	49.24	2.68
-3.03	-11.58	-6.09	-5.11	-2.62	50.2	2.28
-1.33	-5.77	-6.97	-3.66	-2.62	78.95	3.42
-0.54	-10.45	-7.03	-4.88	-2.62	54.93	2.94
-2.82	-18.37	-6.04	-6.24	-2.62	28.7	2.44
-2.69	-11.53	-6.47	-5.12	-2.62	50.4	2.68
-4.7	-16.29	-7.5	-5.98	-2.61	34.16	3.96
-4.68	-16.33	-7.35	-5.99	-2.61	34.04	3.87
-4.51	-16.35	-6.9	-5.99	-2.61	33.98	3.38
-2.99	-15.55	-4.93	-5.83	-2.61	36.33	1.61
-2.75	-15.82	-4.43	-5.87	-2.61	35.5	1.34
3.4	-19.23	-6.44	-6.33	-2.61	26.65	2.78
-3.71	-12.42	-4.06	-5.28	-2.61	46.9	1.14
-3.62	-15.23	-6.73	-5.78	-2.61	37.3	3.12
-3.34	-14.28	-6.17	-5.62	-2.61	40.33	2.64
-2.28	-8.52	-4.95	-4.42	-2.61	63.86	1.29
-2.88	-18.29	-6.45	-6.22	-2.61	28.89	2.77

-2.4	-7.95	-6.69	-4.25	-2.61	66.74	3.1
-4.52	-16.48	-7.5	-6	-2.6	33.59	3.96
-0.88	-3.59	-9.19	-2.95	-2.6	92.91	6.68
-3.1	-6.31	-5.52	-3.8	-2.6	75.67	2.06
-0.7	-18.88	-8.21	-6.28	-2.6	27.44	5.11
4.79	-11.2	5.08	0.23	-2.59	28.61	1.39
3.74	-10.78	4.71	-0.1	-2.59	31.04	1.01
3.68	-19.73	-7.26	-6.38	-2.59	25.5	3.79
-2.4	-12.09	-5.11	-5.21	-2.59	48.07	2.22
-1.61	-11.53	-6.03	-5.09	-2.58	50.26	2.13
-3.03	-15.15	-5.67	-5.75	-2.58	37.45	2.12
-2.05	-13.28	-2.46	-5.43	-2.58	43.65	0.61
-1.46	-6.59	-6.25	-3.87	-2.58	73.93	2.67
2.37	-18.8	-8.06	-6.26	-2.58	27.59	4.59
-3.12	-6.16	-5.49	-3.74	-2.58	76.41	1.94
-2.99	-8.67	-5.42	-4.41	-2.57	62.88	2.01
-0.39	-5.68	-3.21	-3.61	-2.57	79.16	0.78
-2.99	-15.46	-5.76	-5.78	-2.56	36.48	2.23
2.56	-18.64	-4.89	-6.44	-2.56	28.59	1.32
-1.26	-4.27	-7.14	-3.15	-2.56	88.05	3.72
-2.29	-7.87	-8.44	-4.21	-2.56	66.88	5.67
-2.08	-22.3	-7.42	-6.64	-2.56	20.35	4.11
1.49	-22.65	-8.27	-6.67	-2.56	19.74	5.42
2.36	-22.47	-8.48	-6.66	-2.56	20.04	5.39
2.94	-22.29	-8.1	-6.64	-2.56	20.38	4.68
-3.11	-6.27	-5.67	-3.76	-2.56	75.65	2.12
-3.31	-9.19	-5.94	-4.54	-2.56	60.34	2.42
-2.91	-13.31	-5.99	-5.43	-2.56	43.45	2.38
-2.45	-11.86	-6.35	-5.15	-2.56	48.85	2.82
-1.87	-13.96	-5.94	-5.53	-2.55	41.21	2.27
-2.84	-15.66	-5.51	-5.8	-2.55	35.83	2.04
-0.29	-3.69	-7.84	-2.95	-2.55	91.87	4.22
2.55	-22.49	-8.23	-6.66	-2.55	20.01	4.8
-3.1	-14.23	-5.96	-5.59	-2.55	40.31	2.34
-2.97	-13.72	-6.07	-5.5	-2.55	42.03	2.71
-1.28	-13.87	-6.46	-5.51	-2.54	41.5	2.79
0.1	-5.45	-5.5	-3.51	-2.54	80.39	1.92
-3.43	-14.63	-6.09	-5.64	-2.54	38.99	2.48
-2.89	-5.75	-5.52	-3.59	-2.54	78.62	2.01
-2.58	-10.05	-7.46	-4.72	-2.53	56.29	4.05
-2.6	-10.05	-7.47	-4.72	-2.53	56.29	4.07
-2.6	-10.1	-7.54	-4.74	-2.53	56.07	4.29
-4.59	-16.09	-7.76	-5.89	-2.53	34.53	4.52
-4.6	-16.16	-7.61	-5.91	-2.53	34.32	4.28

-4.68	-16.26	-7.46	-5.92	-2.53	34.05	3.97
-4.63	-16.33	-7.39	-5.93	-2.53	33.84	3.9
-4.54	-16.48	-7.25	-5.95	-2.53	33.43	3.67
-1.33	-6.19	-7.96	-3.72	-2.53	75.98	4.49
-2.5	-8.67	-3.24	-4.4	-2.53	62.72	0.9
-2.38	-7.64	-3.78	-4.12	-2.53	67.96	1.08
-0.74	-19.4	-8.68	-6.29	-2.53	26.12	5.86
-0.64	-19	-7.48	-6.25	-2.53	27.02	4.16
-3.39	-11.99	-3.97	-5.12	-2.52	48.23	1.17
-1.78	-6.2	-6.36	-3.71	-2.52	75.82	2.73
-2.19	-11.02	-4.81	-4.93	-2.52	52.11	1.85
-3.24	-14.53	-6.37	-5.61	-2.52	39.26	3.01
-3.06	-6.35	-5.17	-3.76	-2.52	74.98	1.87
3.76	-2.85	-4.63	-2.65	-2.52	97.62	1.3
-2.49	-18.87	-4.9	-6.22	-2.51	27.3	1.7
-2.58	-11.27	-4.06	-4.97	-2.51	51.04	1.26
-2.59	-9.99	-7.21	-4.69	-2.5	56.44	3.74
-3.06	-15.52	-5.36	-5.75	-2.5	36.1	1.89
-2.92	-15.38	-4.98	-5.72	-2.5	36.52	1.67
1.85	-22.48	-7.75	-6.62	-2.5	19.95	4.76
7.55	-18.8	-8.58	-6.21	-2.5	27.42	5.51
-4.53	-16.28	-6.88	-5.89	-2.49	33.88	3.29
-2.33	-11.95	-4.73	-5.1	-2.49	48.23	1.46
-2.64	-18.96	-5.3	-6.22	-2.49	27.03	2.21
-0.54	-19.1	-7.6	-6.23	-2.49	26.7	4.38
-3.25	-8.97	-5.62	-4.44	-2.49	61.1	2.29
-2.94	-8.64	-5.34	-4.36	-2.49	62.7	2.02
-2.84	-13.16	-5.37	-5.35	-2.49	43.78	2.19
2.87	-2.75	-7.81	-2.58	-2.48	97.98	4.48
-2.71	-8.98	-8.34	-4.43	-2.48	61.02	5.55
-3.32	-10.78	-6.97	-4.85	-2.48	52.97	3.33
-2.71	-11.44	-5.31	-5	-2.48	50.25	1.87
-2.6	-9.92	-7.09	-4.65	-2.47	56.61	3.63
-1.01	-5.71	-6.87	-3.54	-2.47	78.4	3.55
-1.23	-4.17	-7.28	-3.06	-2.47	88.1	3.99
-1.21	-4.17	-7.62	-3.06	-2.47	88.05	4.47
-1.62	-22.45	-7.86	-6.6	-2.47	19.96	4.84
4.66	-19.58	-5.12	-6.27	-2.47	25.61	1.98
-0.17	-5.71	-2.93	-3.55	-2.47	78.45	0.8
-2.54	-10.12	-7.51	-4.69	-2.46	55.69	4.45
-4.63	-16.5	-7.21	-5.9	-2.46	33.2	3.83
-4.61	-16.5	-6.92	-5.91	-2.46	33.19	3.5
-4.52	-16.4	-6.94	-5.89	-2.46	33.46	3.53
-2.26	-11.68	-3.98	-5.02	-2.46	49.21	1.21

-0.67	-6.04	-6.48	-3.63	-2.46	76.44	3.09
-0.53	-6.23	-7.32	-3.69	-2.46	75.35	4.23
-2.35	-7.25	-3.09	-3.97	-2.46	69.64	0.87
-0.39	-5.85	-3.68	-3.58	-2.46	77.5	1.09
-4.5	-16.45	-7.67	-5.89	-2.45	33.32	4.53
-4.53	-16.53	-7.12	-5.9	-2.45	33.08	3.78
-4.46	-16.4	-7.26	-5.88	-2.45	33.46	3.85
3.93	-18.79	-6.34	-6.17	-2.45	27.36	2.68
-0.4	-19	-7.76	-6.2	-2.45	26.88	4.8
-2.37	-6.61	-5.91	-3.79	-2.45	73.12	2.25
-4.53	-16.5	-7.6	-5.89	-2.44	33.14	4.44
-2.45	-15.84	-4.87	-5.75	-2.44	35.01	1.74
-2.23	-8.09	-8.48	-4.19	-2.44	65.19	5.97
-3.35	-12.04	-4.4	-5.08	-2.44	47.73	1.45
-2.2	-12.26	-4.52	-5.13	-2.44	46.9	1.54
-1.08	-16.68	-4.62	-5.89	-2.44	32.63	1.59
6.81	-19.12	-8.24	-6.2	-2.43	26.55	5.22
-3.05	-6.48	-4.63	-3.74	-2.43	73.7	1.62
1.66	-19.47	-4.94	-6.22	-2.42	25.73	1.94
-2.89	-10.07	-5.11	-4.65	-2.42	55.76	1.94
-2.51	-5.36	-5.77	-3.39	-2.41	80.18	2.46
-3.18	-8.39	-4.65	-4.24	-2.41	63.55	1.44
-1.82	-8.73	-4.78	-4.33	-2.41	61.92	2.16
-1.14	-8.63	-4.83	-4.3	-2.4	62.31	1.53
-3.26	-17.14	-5.71	-5.94	-2.4	31.35	2.52
-1.03	-22.37	-8	-6.54	-2.4	19.99	5.04
1.13	-22.49	-8.12	-6.55	-2.4	19.78	5.15
2.84	-19.67	-6.41	-6.24	-2.4	25.28	3.25
-2.81	-12.68	-5.78	-5.2	-2.4	45.21	2.51
-0.89	-2.79	-1.36	-2.55	-2.4	97.18	0.35
-0.82	-5.63	-6.92	-3.46	-2.39	78.44	3.62
-3.03	-17.47	-6.4	-5.98	-2.39	30.45	3.03
-0.74	-20.03	-6.39	-6.29	-2.39	24.49	3.06
2.49	-19.55	-5.82	-6.22	-2.39	25.52	2.43
-0.83	-4.05	-9.28	-2.97	-2.39	88.4	7.95
-2.82	-5.68	-5.22	-3.48	-2.39	78.14	1.89
-2.85	-12.95	-6.08	-5.25	-2.39	44.2	2.57
-2.17	-11.76	-3.75	-4.99	-2.38	48.62	1.28
-3.26	-9.59	-4.19	-4.51	-2.38	57.72	1.16
-2.23	-8.75	-3.39	-4.3	-2.38	61.63	1.06
-2.8	-11.52	-5.23	-4.92	-2.37	49.48	1.99
-4.56	-15.83	-7.96	-5.74	-2.37	34.85	4.87
-4.48	-16.33	-7.23	-5.82	-2.37	33.44	4.01
-4.48	-16.37	-7.61	-5.82	-2.37	33.33	4.45

-1.11	-6.05	-8.16	-3.58	-2.37	75.86	5.11
-2.26	-12.08	-4.98	-5.05	-2.37	47.34	1.7
-2.39	-8.04	-4.09	-4.12	-2.37	65.09	1.22
-2.06	-3.23	-3.96	-2.68	-2.37	93.85	1.26
-2.33	-15.92	-4.88	-5.71	-2.36	34.58	1.77
-2.7	-11.44	-4.22	-4.9	-2.36	49.77	1.42
-3.07	-7.55	-4.2	-3.99	-2.36	67.59	1.37
1.22	-9.62	-6.95	-4.5	-2.36	57.53	3.5
3.56	-3.32	-3.21	-2.71	-2.36	93.15	0.79
-2.65	-11.31	-3.91	-4.87	-2.35	50.28	1.18
6	-18.17	-7.96	-6.03	-2.35	28.61	4.53
-0.25	-16.21	-4.87	-5.76	-2.35	33.75	1.75
-0.32	-5.04	-5.64	-3.25	-2.35	81.75	2.11
-0.28	-5.58	-2.94	-3.43	-2.35	78.48	0.78
-3.15	-11.91	-4.83	-4.98	-2.34	47.86	1.71
-0.55	-22.54	-7.26	-6.51	-2.34	19.6	4.23
2.18	-22.28	-7.09	-6.49	-2.34	20.06	3.68
-2.5	-11.43	-4.15	-4.89	-2.34	49.74	1.34
-1.55	-11.28	-2.43	-4.85	-2.34	50.34	0.54
-0.12	-15.91	-5.22	-5.71	-2.34	34.55	2.03
5.84	-20.68	-5.83	-6.31	-2.34	23.07	2.39
-2.93	-10.01	-4.97	-4.58	-2.34	55.72	1.78
-2.25	-12.37	-5.89	-5.1	-2.34	46.17	2.85
-3.14	-11.97	-4.93	-4.99	-2.33	47.63	1.68
-2.23	-16.17	-4.99	-5.73	-2.33	33.81	1.89
4.41	-11.34	5	0.13	-2.33	28.56	1.22
-1.95	-7.74	-4.98	-4.03	-2.33	66.42	1.84
-1.88	-8.1	-5.95	-4.12	-2.33	64.61	2.7
0.01	-16.81	-5.15	-5.83	-2.33	32.04	2.04
-3.18	-8.53	-4.1	-4.22	-2.33	62.46	1.26
-0.13	-22.49	-8.34	-6.49	-2.32	19.67	5.69
-1.69	-19.1	-4.35	-6.11	-2.32	26.38	1.53
-2.58	-8.61	-3.33	-4.25	-2.32	62.08	1.01
-2.91	-8.67	-5.56	-4.25	-2.32	61.77	2.34
-3.12	-14.5	-5.82	-5.47	-2.32	38.76	2.32
-2.51	-10.09	-7.16	-4.58	-2.31	55.22	4.19
-0.75	-5.56	-6.75	-3.39	-2.31	78.41	3.58
1.92	-18.75	-4.89	-6.52	-2.31	28.52	1.37
11.51	-6.17	10.53	3.84	-2.31	31.09	0.9
-1.79	-10.32	-3.44	-4.65	-2.31	54.24	1.25
8.47	-18.78	-7.69	-6.07	-2.31	27.11	4.58
-0.7	-16.81	-4.83	-5.82	-2.31	31.99	1.8
-2.66	-5.65	-5.42	-3.41	-2.31	77.84	2.11
-2.48	-8.26	-2.46	-4.15	-2.31	63.7	0.77

-3.13	-10.52	-6.58	-4.67	-2.31	53.37	3.22
-2.9	-13.62	-6.14	-5.32	-2.31	41.63	2.81
-2.92	-13.51	-6.12	-5.3	-2.31	42	2.64
-2.72	-11.46	-4.91	-4.87	-2.3	49.52	1.74
-2.85	-19.38	-5.89	-7.06	-2.3	28.42	1.48
-1.17	-4.01	-7.62	-2.9	-2.3	88.07	4.77
-2.29	-7.69	-2.93	-3.98	-2.3	66.53	0.82
-3.7	-14.02	-7.36	-5.37	-2.29	40.23	3.9
-3.1	-12.24	-3.76	-5.02	-2.29	46.43	1.21
-3.2	-12.24	-3.56	-5.02	-2.29	46.46	1.14
9.22	-18.59	-7.54	-6.04	-2.29	27.5	4.47
-1.46	-8.67	-2.7	-4.24	-2.29	61.64	0.75
-2.18	-7.73	-7.14	-3.99	-2.29	66.29	4.08
0.3	-22.49	-7.6	-6.47	-2.28	19.61	4.56
-0.39	-6.01	-6.74	-3.51	-2.28	75.64	3.69
-2.48	-11.4	-4.02	-4.84	-2.28	49.66	1.34
-2.7	-11.34	-3.46	-4.83	-2.28	49.89	1
-3.01	-7.53	-4.04	-3.93	-2.28	67.28	1.33
-2.58	-18.8	-4.48	-6.05	-2.28	26.99	1.93
0.35	-5.1	-4.68	-3.23	-2.27	80.9	1.59
-2.56	-8.34	-7.15	-4.13	-2.27	63.11	4.19
-3.3	-12.11	-4.37	-4.98	-2.27	46.89	1.47
-1.63	-6.31	-6.66	-3.59	-2.27	73.82	3.17
-0.85	-16.31	-4.59	-5.71	-2.27	33.25	1.57
-0.42	-16.51	-4.79	-5.75	-2.27	32.71	1.79
-0.35	-16.39	-4.9	-5.73	-2.27	33.04	1.83
-0.04	-16.34	-4.51	-5.72	-2.27	33.19	1.59
-0.82	-4.16	-9.1	-2.92	-2.27	86.87	7.99
-3.16	-8.74	-4.11	-4.23	-2.27	61.18	1.34
-2.92	-13.73	-6.01	-5.3	-2.27	41.14	2.75
-2.16	-16.2	-5.38	-5.69	-2.26	33.55	2.22
-0.25	-5.72	-4.47	-3.41	-2.26	77.17	1.59
-2.31	-5.56	-5.36	-3.36	-2.26	78.07	2.36
-2.29	-10.63	-4.86	-4.67	-2.26	52.7	1.82
-3	-6.48	-4.63	-3.63	-2.26	72.84	1.66
-2.88	-10.06	-5.18	-4.54	-2.26	55.17	2.01
-2.61	-11.38	-4.74	-4.85	-2.26	49.67	1.84
0.97	-4.39	-4.19	-2.99	-2.26	85.33	1.26
-1.07	-11.8	-6.48	-4.92	-2.25	48.01	2.99
-2.95	-17.75	-5.33	-5.92	-2.25	29.41	2.29
-2.22	-11.63	-5.35	-4.88	-2.25	48.64	2.23
-0.61	-16.95	-4.88	-5.8	-2.25	31.48	1.83
-0.13	-18.97	-7.84	-6.05	-2.25	26.54	5.27
-2.9	-8.7	-5.44	-4.21	-2.25	61.29	2.3

-3.1	-14.62	-5.58	-5.45	-2.25	38.18	2.31
-0.71	-5.44	-6.35	-3.31	-2.24	78.7	3.2
-3.2	-17.05	-5.72	-5.82	-2.24	31.19	2.48
-2.97	-17.62	-5.97	-5.89	-2.24	29.71	2.75
-0.57	-20.08	-6.12	-6.18	-2.24	24.11	2.92
-2.37	-5.36	-5.68	-3.29	-2.24	79.19	2.52
-2.83	-13.11	-5.87	-5.17	-2.24	43.15	2.55
-1.12	-3.85	-7.19	-2.79	-2.23	88.62	4.24
-2.29	-12.66	-4.47	-5.08	-2.23	44.71	1.94
-2.25	-11.25	-5.48	-4.79	-2.23	50.07	2.31
-2.81	-12.49	-6.05	-5.05	-2.23	45.32	2.69
-1.99	-3.23	-4.18	-2.58	-2.23	92.83	1.48
-1.19	-3.85	-7.63	-2.79	-2.22	88.62	4.97
-2.5	-12.76	-3.44	-5.08	-2.22	44.34	1.11
-2.44	-11.31	-3.1	-4.78	-2.22	49.79	0.99
-3.11	-14.76	-6.3	-5.44	-2.22	37.68	3.17
0.09	-19.13	-7.96	-6.05	-2.22	26.12	5.78
-2.25	-8.89	-7	-4.24	-2.22	60.25	3.94
-2.27	-8.68	-6.78	-4.19	-2.22	61.24	3.59
3.63	-13.28	2.56	-1.54	-2.21	28.4	1.16
-3.13	-12.25	-3.61	-4.97	-2.21	46.13	1.12
-2.09	-8.56	-8.39	-4.15	-2.2	61.75	6.39
1	-8.02	-5.38	-4.01	-2.2	64.39	2.03
-2.91	-7.6	-4.04	-3.9	-2.2	66.55	1.43
-3.02	-10.57	-6.72	-4.61	-2.2	52.71	3.5
-2.19	-12.46	-4.97	-5.02	-2.2	45.35	2.46
-2.03	-3.08	-4.15	-2.51	-2.2	93.65	1.42
-2.05	-16.3	-5.12	-5.66	-2.19	33.11	2.09
-2.23	-11.5	-4.76	-4.81	-2.19	48.91	1.73
9.99	-18.22	-7.93	-5.92	-2.19	28.15	5.12
2.68	-18.79	-7.35	-5.99	-2.19	26.82	3.97
-2.18	-9.25	-7.24	-4.31	-2.19	58.48	4.3
-0.12	-5.77	-4.64	-3.38	-2.18	76.46	1.91
-1.16	-19.16	-4.6	-6.02	-2.18	25.97	1.76
1.38	-4.46	-4.74	-2.96	-2.18	84.37	1.77
-2.33	-10.46	-5.12	-4.58	-2.18	53.11	2.06
6.67	-8.93	7.71	2.25	-2.17	28.37	1.47
-2.56	-8.7	-2.63	-4.17	-2.17	60.95	0.79
6.23	-21.53	-5.37	-6.28	-2.17	21.17	2.65
-3.14	-17.16	-5.62	-5.78	-2.16	30.71	2.51
0.12	-17.34	-5.22	-5.78	-2.16	30.26	2.24
-0.65	-3.14	-7.17	-2.51	-2.16	93.02	4.87
-2.52	-5.55	-5.12	-3.29	-2.16	77.63	1.98
-3.09	-17.26	-6.04	-5.78	-2.15	30.44	2.97

-1.1	-3.7	-6.81	-2.7	-2.15	89.11	3.86
-2.99	-14.62	-6.44	-5.37	-2.15	37.9	3.58
-2.02	-3.08	-4.52	-2.47	-2.14	93.22	1.68
-2.47	-15.15	-3.15	-5.44	-2.14	36.27	0.76
-2.37	-11.28	-5.28	-4.73	-2.14	49.63	2.21
-2.22	-8.91	-7.05	-4.2	-2.14	59.82	4.06
-3.11	-9.3	-4.67	-4.28	-2.14	58.02	1.71
-2.52	-4.51	-3.93	-2.95	-2.14	83.83	1.43
-2.06	-3.05	-4.39	-2.46	-2.13	93.45	1.58
-2.44	-11.41	-3.57	-4.75	-2.13	49.09	1.16
-2.43	-10.31	-3.97	-4.51	-2.13	53.54	1.37
-1.96	-6.51	-5.43	-3.55	-2.13	72	2.12
-0.67	-5.26	-5.97	-3.18	-2.12	79.09	2.98
-0.83	-4.31	-9.45	-2.87	-2.12	84.96	9.29
-2.77	-8.66	-4.25	-4.11	-2.12	60.88	1.58
0.32	-13.84	-6.46	-5.21	-2.11	40.28	3.33
-2	-16.3	-5.42	-5.6	-2.11	32.88	2.46
7.37	-10.94	3.74	-0.28	-2.11	31.13	1.34
-1.72	-8.55	-4.96	-4.09	-2.11	61.41	2.19
2.15	-19.31	-4.6	-5.99	-2.11	25.52	1.72
5.94	-20.17	-5.4	-6.09	-2.11	23.7	2.24
-1.29	-3.39	-3.09	-2.57	-2.11	90.97	1.07
-2.91	-6.68	-4.36	-3.58	-2.1	70.89	1.64
-2.9	-6.72	-4.68	-3.59	-2.1	70.65	1.91
-3.33	-13.59	-4.78	-5.15	-2.1	41.07	2.03
-1.14	-6.25	-2.82	-3.46	-2.1	73.24	0.88
-1.09	-6.13	-2.68	-3.43	-2.1	73.92	0.82
0.47	-17.12	-5.63	-5.71	-2.09	30.66	2.64
-2.55	-8.55	-2.5	-4.08	-2.09	61.33	0.78
0.95	-10.56	-6.58	-4.53	-2.08	52.28	3.7
-0.26	-5.93	-6.07	-3.35	-2.08	74.93	3.03
0.22	-17.14	-5.56	-5.71	-2.08	30.6	2.55
-3.08	-13.11	-5.81	-5.04	-2.08	42.65	2.78
-3.2	-17.13	-6.02	-5.71	-2.07	30.6	2.93
-3.05	-17.38	-6.39	-5.74	-2.07	29.94	3.49
6.02	-4.57	-6.5	-2.93	-2.07	83	3.88
-2.19	-12.34	-4.73	-4.9	-2.07	45.35	1.93
-2.99	-8.88	-4.31	-4.13	-2.07	59.64	1.58
-4.48	-15.64	-7.7	-5.5	-2.06	34.62	4.9
-7.98	-25.17	-12.78	-12.54	-2.06	28.3	1.23
-1.12	-3.62	-6.72	-2.62	-2.06	89.11	3.72
-2.46	-12.61	-3.73	-4.94	-2.06	44.34	1.27
-2.68	-6.22	-4.92	-3.42	-2.06	73.17	2
0.29	-19.09	-8.02	-5.93	-2.06	25.89	6.07

-1.97	-7.33	-6.96	-3.72	-2.06	67.22	4.22
6.57	-8.71	8.24	2.46	-2.05	28.3	1.28
-2.45	-8.42	-6.83	-4.01	-2.05	61.75	3.97
-2.96	-10.82	-3.94	-4.56	-2.05	51.13	1.4
-2.57	-6.22	-4.29	-3.41	-2.05	73.14	1.68
-2.95	-11.03	-4.07	-4.61	-2.05	50.27	1.58
-2.09	-8.51	-3.91	-4.04	-2.05	61.34	1.59
-2.03	-8.27	-4.27	-3.98	-2.05	62.47	1.79
-2.44	-4.43	-4.06	-2.88	-2.05	83.77	1.44
-2.87	-17.91	-5.65	-5.79	-2.04	28.59	2.65
2.94	-18.71	-4.75	-6.39	-2.04	28.26	1.33
-2.06	-8.83	-8.52	-4.11	-2.04	59.76	6.93
-2.07	-12.36	-5.02	-4.88	-2.04	45.15	1.85
-2.87	-7.56	-3.98	-3.78	-2.04	65.96	1.49
-1.63	-8.24	-5.57	-3.97	-2.04	62.58	3.07
-1.45	-10.15	-5.19	-4.42	-2.03	53.84	2.34
-2.27	-5.37	-4.98	-3.15	-2.03	77.91	2.14
4.9	-18.58	-4.7	-5.84	-2.03	26.98	1.64
-2.95	-12.23	-3.63	-4.84	-2.02	45.58	1.27
-2.27	-5.53	-4.69	-3.19	-2.02	76.91	1.79
1.57	-9.96	-6.22	-4.35	-2.02	54.63	3.04
-0.65	-5.11	-5.77	-3.06	-2.01	79.34	2.91
0.46	-3.23	-7.32	-2.45	-2.01	91.37	4.24
-2.16	-11.84	-2.74	-4.75	-2.01	47	0.98
0.65	-17.15	-5.5	-5.66	-2.01	30.43	2.77
-3.18	-13.44	-5.11	-5.06	-2.01	41.32	2.23
-2.94	-14.46	-5.53	-5.26	-2.01	38.03	2.58
12.21	-19.04	-6.88	-5.89	-2	25.9	4.67
-2.96	-9.93	-4.75	-4.34	-2	54.66	1.79
-2.37	-10.12	-6.95	-4.37	-1.99	53.79	4.43
-1.16	-3.44	-7.24	-2.5	-1.99	89.79	4.9
-2.93	-12.28	-3.29	-4.82	-1.99	45.27	1.13
-1.43	-6.07	-5.67	-3.33	-1.99	73.62	2.46
-0.21	-8.52	-2.68	-4	-1.99	60.99	1.01
-2.1	-9.33	-7.28	-4.2	-1.99	57.27	4.5
-2.01	-8.13	-6.86	-3.9	-1.99	62.84	4.31
-4.35	-15.51	-7.59	-5.43	-1.98	34.78	5.2
-3.02	-17.5	-6.36	-5.7	-1.98	29.47	3.25
-1.2	-3.5	-7.55	-2.52	-1.98	89.3	5.22
-1.92	-2.95	-4.23	-2.33	-1.98	93.05	1.67
0.04	-5.98	-6.79	-3.31	-1.98	74.11	4.17
-2.52	-4.26	-4.09	-2.77	-1.98	84.43	1.58
-2.88	-6.63	-4.91	-3.48	-1.98	70.52	2.1
-2.55	-4.39	-3.75	-2.81	-1.98	83.55	1.32

22.69	-5.17	12.82	5.53	-1.97	28.16	1.12
-1.84	-10.03	-4.02	-4.35	-1.97	54.11	1.41
-3.03	-13.12	-4.11	-4.97	-1.97	42.26	1.61
-2.59	-8.75	-3.97	-4.04	-1.97	59.83	1.56
-2.04	-9.6	-8.31	-4.24	-1.96	55.96	6.69
-2.08	-9.85	-8.31	-4.31	-1.96	54.84	6.68
-2.08	-10.1	-8.25	-4.37	-1.96	53.8	6.61
-1.49	-12.94	-5.33	-4.96	-1.96	42.84	2.34
-2.17	-12.32	-4.29	-4.82	-1.96	45.05	1.63
-0.82	-4.49	-9.58	-2.83	-1.96	82.84	10.27
-2.55	-6.33	-4.19	-3.39	-1.96	72.02	1.76
-2	-10.49	-8.18	-4.44	-1.95	52.08	6.69
-2.08	-10.22	-6.7	-4.36	-1.95	53.24	3.84
-0.88	-5.46	-2.55	-3.12	-1.95	76.95	0.89
-1.5	-2.93	-4.74	-2.3	-1.94	92.98	1.91
-2.76	-6.49	-4.84	-3.42	-1.94	71.04	2.1
-2.76	-6.45	-5.01	-3.4	-1.94	71.29	2.22
-3.31	-13.64	-4.79	-5.05	-1.94	40.42	2.07
-3.25	-13.55	-4.65	-5.03	-1.94	40.75	2.06
-1.87	-10.29	-7.44	-4.39	-1.94	52.9	5.17
-1.99	-9.8	-7.25	-4.28	-1.94	54.98	4.75
-0.6	-4.92	-5.72	-2.95	-1.93	79.98	2.93
-2.2	-5.46	-4.36	-3.11	-1.93	76.82	1.82
-2.86	-6.57	-4.97	-3.43	-1.93	70.58	2.18
-2.7	-6.22	-5.24	-3.34	-1.93	72.52	2.33
1.69	-7.92	-3.72	-3.81	-1.93	63.65	1.36
-1.78	-10.34	-8.39	-4.39	-1.93	52.65	6.94
-0.6	-12.03	-6.89	-4.74	-1.92	45.98	3.83
-2.19	-8.65	-2.75	-3.99	-1.92	60.04	0.89
-2.56	-8.55	-3.09	-3.96	-1.92	60.53	0.89
1.05	-7.7	-3.8	-3.74	-1.92	64.67	1.17
-1.92	-9.98	-7.2	-4.3	-1.92	54.14	4.7
-1.8	-8.25	-5.66	-3.89	-1.92	61.97	2.85
-2.9	-19.56	-5.25	-7.14	-1.91	28.14	1.16
-1.1	-3.35	-7.03	-2.42	-1.91	89.87	4.63
-1.63	-2.76	-4.17	-2.22	-1.91	93.94	1.69
-2.25	-8.57	-6.88	-3.95	-1.91	60.4	4.46
1.18	-2.38	-6.12	-2.08	-1.91	96.55	3.34
-1	-9.28	-3.33	-4.13	-1.91	57.14	1.01
-1.99	-9.1	-3.2	-4.08	-1.91	57.95	1.14
-1.86	-7.05	-6.7	-3.55	-1.91	67.91	4.25
-0.83	-2.36	-0.76	-2.08	-1.91	96.69	0.55
-1.62	-2.73	-4.6	-2.21	-1.9	94.11	1.99
10.8	-18.4	-6.88	-5.74	-1.9	27.15	4.03

-2.37	-8.78	-3.35	-4.01	-1.9	59.37	1.32
-1.96	-8.12	-7.15	-3.84	-1.9	62.5	5.04
-1.89	-2.83	-4.75	-2.24	-1.89	93.3	2
-2.73	-7.49	-3.69	-3.66	-1.89	65.56	1.37
-2.27	-8.83	-4.19	-4.01	-1.89	59.07	1.66
-2.28	-8.87	-4.08	-4.02	-1.89	58.9	1.68
-1.89	-16.54	-5.12	-5.47	-1.88	31.68	2.42
-2.84	-7.5	-4	-3.66	-1.88	65.5	1.62
-2.94	-11.03	-4.03	-4.5	-1.88	49.65	1.64
-1.6	-9.86	-5.07	-4.24	-1.87	54.41	2.46
-1.66	-5.47	-4.58	-3.07	-1.87	76.4	1.86
3.26	-7.44	-3.67	-3.63	-1.87	65.73	1.29
-3	-13.2	-4.26	-4.92	-1.87	41.69	1.8
-1.66	-8.08	-5.37	-3.82	-1.87	62.53	2.52
1.72	-4.18	-4.37	-2.66	-1.86	84.13	1.63
0.86	-2.39	-6.36	-2.06	-1.86	96.17	3.91
-2.68	-7.48	-3.87	-3.65	-1.86	65.48	1.51
-0.44	-4.79	-5.58	-2.86	-1.85	80.36	2.95
-2.77	-17.79	-4.67	-5.65	-1.85	28.49	2.08
-0.18	-20.5	-5.67	-5.96	-1.85	22.6	3.01
-2.3	-8.73	-2.84	-3.97	-1.85	59.38	0.96
-0.67	-8.69	-3.81	-3.95	-1.85	59.6	1.23
-1.19	-10.34	-8.16	-4.34	-1.85	52.32	6.46
-1.63	-6.48	-4.32	-3.36	-1.85	70.66	1.63
-2.62	-13.83	-2.43	-5.04	-1.84	39.5	1.05
-2.28	-4.41	-4.18	-2.73	-1.84	82.58	1.56
-2.94	-9.76	-5.32	-4.19	-1.84	54.75	2.54
-1.06	-3.26	-7.01	-2.35	-1.83	89.92	4.66
-1.03	-3.33	-7.34	-2.36	-1.83	89.48	5.2
3.94	-2.05	-6.8	-1.91	-1.83	98.36	4.17
-2.08	-13.3	-3.28	-4.92	-1.83	41.23	1.29
-2.3	-8.77	-3.23	-3.95	-1.83	59.11	1.18
-1.97	-8.3	-3.84	-3.84	-1.83	61.3	1.8
-1.79	-2.71	-4.16	-2.15	-1.82	93.66	1.69
-1.73	-2.64	-4.78	-2.13	-1.82	94.11	2.15
-1.91	-10.8	-7.84	-4.42	-1.82	50.34	6.31
-0.75	-11.86	-1.61	-4.62	-1.82	46.25	0.51
-0.29	-11.78	-2.14	-4.6	-1.82	46.54	0.73
0.2	-6.29	-7.07	-3.29	-1.82	71.56	5
-2.18	-8.79	-3.66	-3.95	-1.82	58.94	1.38
-2.42	-3.8	-4.34	-2.51	-1.82	86.37	1.85
-2.66	-7.58	-3.73	-3.64	-1.82	64.78	1.5
6.08	-20.26	-4.39	-5.9	-1.82	23.02	1.85
0.47	-4.78	-4.96	-2.83	-1.82	80.21	2.18

-1.64	-2.63	-4.71	-2.11	-1.81	94.16	2.01
-2.27	-11.58	-4.09	-4.56	-1.81	47.26	1.45
13.3	-18	-6.91	-5.63	-1.81	27.89	4.87
-2.75	-12.86	-3.84	-4.81	-1.81	42.63	1.51
-2.63	-11.82	-4.05	-4.61	-1.81	46.37	1.6
-2.3	-4.14	-3.46	-2.63	-1.81	84.09	1.37
-1.63	-2.63	-4.11	-2.1	-1.8	94.03	1.64
1.41	-13.12	1.22	-2.21	-1.8	31.16	1.2
6.63	-4.31	-5.05	-2.67	-1.8	82.97	2.43
-3.35	-12.03	-2.44	-4.66	-1.8	45.56	1.01
-1.16	-5.49	-4.36	-3.03	-1.8	75.93	1.79
-2.72	-12.68	-3.96	-4.78	-1.8	43.27	1.61
-0.8	-12.74	-5.69	-4.8	-1.79	43.02	2.82
-2.23	-8.79	-3.43	-3.93	-1.79	58.87	1.27
-1.73	-7.88	-5.37	-3.71	-1.79	63.16	2.57
1.42	-2.19	-6.41	-1.93	-1.78	97.02	3.74
-2.81	-7.44	-3.86	-3.58	-1.78	65.28	1.48
-3.24	-13.63	-4.7	-4.93	-1.78	40.01	2.23
-0.99	-6.13	-2.38	-3.22	-1.78	72.23	0.73
0.81	-16.52	-5.58	-5.41	-1.77	31.48	3.01
-2.88	-8.35	-3.77	-3.8	-1.77	60.79	1.38
-2.44	-3.87	-4.44	-2.51	-1.77	85.58	1.9
-2.84	-14.41	-5.77	-5.09	-1.77	37.5	2.9
-2.31	-12.15	-5.45	-4.67	-1.77	45.02	2.29
-2.3	-10.15	-6.8	-4.23	-1.76	52.76	4.52
-1	-3.31	-6.9	-2.31	-1.76	89.12	4.72
-0.96	-3.28	-6.79	-2.31	-1.76	89.34	4.58
-1.4	-2.82	-4.65	-2.14	-1.76	92.45	1.96
-3.2	-13.58	-5.6	-4.92	-1.76	40.1	2.75
-2.68	-12.74	-4.05	-4.76	-1.76	42.9	1.68
-1.8	-7.02	-6.5	-3.45	-1.76	67.34	4.23
-2.27	-10.2	-7.05	-4.23	-1.75	52.54	4.92
0.85	-13.79	-6.29	-4.96	-1.75	39.39	3.62
-2.16	-8.43	-7.2	-3.81	-1.75	60.35	5.36
-2.78	-12.17	-2.93	-4.64	-1.75	44.88	1.07
-1.23	-6.47	-5.16	-3.29	-1.75	70.19	2.39
11.59	-18.65	-6.57	-5.67	-1.75	26.28	3.99
-2.11	-4.4	-4.33	-2.66	-1.75	82.11	1.79
-1.61	-2.53	-4.95	-2.03	-1.74	94.3	2.32
-1.14	-3.1	-4.54	-2.23	-1.74	90.48	2.08
-2.74	-15.36	-3.46	-5.22	-1.74	34.6	1.66
1.06	-4.53	-4.57	-2.71	-1.74	81.29	1.69
-2.31	-11.51	-3.15	-4.5	-1.74	47.33	1.35
-2.45	-8.7	-3.65	-3.88	-1.74	59.04	1.46

-2.4	-3.77	-4.14	-2.46	-1.74	86.01	1.78
2.79	-7.67	-2.79	-3.61	-1.74	63.97	0.96
-2.33	-4.15	-3.65	-2.58	-1.74	83.64	1.41
-1.86	-16.5	-4.94	-5.37	-1.73	31.45	2.45
-1.53	-2.61	-4.92	-2.06	-1.73	93.71	2.21
-1.58	-8.39	-3.85	-3.8	-1.73	60.41	1.63
0.05	-5.6	-4.33	-3.03	-1.73	74.86	2.06
-2.78	-15.05	-6.01	-5.15	-1.73	35.46	3.54
4.34	-20.13	-7	-5.83	-1.73	23.12	4.88
-2.47	-3.94	-4.42	-2.51	-1.73	84.83	1.93
-2.39	-3.65	-3.92	-2.41	-1.73	86.78	1.69
-2.29	-4.16	-3.45	-2.58	-1.73	83.51	1.44
-0.69	-3.42	-5.67	-2.32	-1.72	88.16	3.12
-1.12	-3.08	-5	-2.21	-1.72	90.48	2.49
-1.84	-9.03	-3.89	-3.95	-1.72	57.46	1.58
-1.83	-9.33	-4.41	-4.02	-1.72	56.15	1.87
-1.34	-11.71	-4.69	-4.53	-1.72	46.5	2.2
-2.51	-4.06	-4.28	-2.54	-1.72	84.06	1.87
-2.35	-3.66	-4.43	-2.4	-1.72	86.62	2.05
-2.58	-6.27	-4.09	-3.21	-1.72	71.12	1.75
6.41	-20.71	-4.86	-5.88	-1.72	21.97	2.01
6.09	-20.09	-4.38	-5.81	-1.72	23.19	1.63
-2.8	-17.72	-4.66	-5.54	-1.71	28.35	2.15
-0.73	-3.33	-6.34	-2.29	-1.71	88.66	4.04
-2.43	-13.76	-2.33	-4.93	-1.71	39.37	1.02
-1.41	-12.91	-4.66	-4.76	-1.71	42.15	2.14
-1.2	-6.56	-4.3	-3.3	-1.71	69.52	1.81
-2.75	-18.04	-5.19	-5.58	-1.7	27.59	2.76
-1.64	-15.78	-3.58	-5.24	-1.7	33.32	1.93
0.02	-20.71	-5.52	-5.88	-1.7	21.94	3.11
0.02	-2.23	-7.71	-1.89	-1.7	96.22	5.96
-1.57	-10.99	-6.68	-4.36	-1.7	49.14	4.55
-0.22	-10.58	-8.3	-4.29	-1.7	50.79	7.27
-0.31	-4.58	-5.9	-2.68	-1.69	80.68	3.42
-0.82	-3.25	-6.57	-2.25	-1.69	89.08	4.37
-0.38	-3.06	-4.72	-2.19	-1.69	90.33	2.28
-2.69	-15.31	-5.6	-5.17	-1.69	34.6	3.19
-0.6	-2.71	-6.27	-2.07	-1.69	92.8	4.07
0.35	-11.23	-8.73	-4.42	-1.69	48.2	8.57
-0.88	-3.25	-6.54	-2.25	-1.68	89.05	4.22
-0.33	-3.04	-4.71	-2.17	-1.68	90.39	2.22
-1.09	-3.03	-4.31	-2.17	-1.68	90.5	1.91
0.97	-16.5	-6.14	-5.34	-1.68	31.34	3.88
1.16	-18.93	-7.25	-5.66	-1.68	25.53	5.51

0.66	-4.51	-2.23	-2.66	-1.68	81.04	0.85
-0.39	-4.3	-4.2	-2.59	-1.67	82.3	1.9
-4.19	-15.2	-7.31	-5.17	-1.67	34.88	5.02
-0.97	-3.24	-6.78	-2.24	-1.67	89	4.51
-0.78	-3.25	-6.44	-2.24	-1.67	89	4.09
-2.29	-8.23	-3.31	-3.71	-1.67	60.92	1.32
-2.4	-3.68	-3.88	-2.38	-1.67	86.19	1.63
-1.54	-8.38	-5.01	-3.76	-1.67	60.2	3.03
0.03	-4.8	-3.99	-2.74	-1.66	79.16	1.77
-1.82	-11.06	-7.88	-4.37	-1.66	48.75	6.63
-3.53	-13.67	-6.96	-4.88	-1.66	39.52	4.26
-1.53	-14.15	-3.83	-4.95	-1.66	37.99	1.6
-1.25	-12.01	-4.01	-4.56	-1.66	45.18	1.81
-0.64	-4.83	-9.08	-2.75	-1.66	79.02	10.02
-2.39	-3.58	-4	-2.34	-1.66	86.72	1.68
-2.64	-7.57	-3.4	-3.54	-1.66	64.07	1.38
-2.6	-13	-3.79	-4.74	-1.66	41.72	1.6
0.07	-11.08	-8.62	-4.37	-1.66	48.66	8.17
8.5	-9.66	12.03	3.8	-1.65	21.83	0.89
0.09	-16.95	-5.09	-5.37	-1.65	30.11	2.85
-0.47	-3.06	-4.4	-2.16	-1.65	90.12	2.03
2.31	-4.08	-4.39	-2.5	-1.65	83.45	1.98
-2.31	-8.65	-2.94	-3.81	-1.65	58.89	1.07
-2.03	-3.89	-4.49	-2.44	-1.65	84.69	2.19
-2.49	-6.16	-3.69	-3.13	-1.65	71.36	1.53
-2.45	-6.2	-3.76	-3.15	-1.65	71.09	1.65
6.66	-21.03	-4.67	-5.87	-1.65	21.27	2.47
-2.27	-8.81	-4.24	-3.84	-1.65	58.14	1.93
2.05	-4.08	-3.64	-2.5	-1.65	83.51	1.42
-0.29	-4.44	-5.62	-2.61	-1.64	81.24	3.15
0.84	-17.38	-6.09	-5.43	-1.64	29.02	4.03
-0.71	-3.29	-6.36	-2.23	-1.64	88.52	4.03
-2.37	-3.51	-4.51	-2.3	-1.64	87.03	2.04
-2.31	-3.71	-4.66	-2.38	-1.64	85.79	2.24
-2.74	-12.93	-4.09	-4.72	-1.64	41.9	1.71
-2.26	-8.71	-4.44	-3.82	-1.64	58.56	1.96
-0.66	-3.39	-5.64	-2.26	-1.63	87.8	3.26
-3.24	-15.37	-4.47	-5.16	-1.63	34.31	2.07
-3.22	-15.51	-5.19	-5.17	-1.63	33.9	2.86
-2.13	-11.84	-2.92	-4.49	-1.63	45.68	1.18
-1.46	-11.17	-6.5	-4.36	-1.63	48.21	4.28
-0.32	-6.19	-3.55	-3.13	-1.63	71.07	1.48
-1.68	-7.96	-5.46	-3.63	-1.63	62.03	2.73
-1.15	-3.12	-2.02	-2.17	-1.63	89.57	0.87

2.62	-3.98	-5.35	-2.45	-1.62	83.93	2.79
1.58	-1.99	-6.41	-1.76	-1.62	97.35	4
-1.51	-10.98	-6.48	-4.31	-1.62	48.92	4.2
-2.84	-12.93	-4.77	-4.7	-1.62	41.85	2.3
0.89	-11.62	-8.96	-4.46	-1.62	46.46	9.3
-0.23	-4.24	-3.23	-2.54	-1.62	82.28	1.3
1.46	-17	-6.51	-5.36	-1.61	29.9	4.7
0.32	-12.78	1.5	-1.92	-1.61	31.2	1.31
-1.46	-13.34	-4.41	-4.77	-1.61	40.44	2.04
-1.64	-11.02	-6.91	-4.31	-1.61	48.7	4.95
-1.86	-4.27	-4.62	-2.54	-1.61	82.09	2.1
-2.33	-3.63	-4.78	-2.33	-1.61	86.15	2.36
-3.2	-13.64	-5.3	-4.82	-1.61	39.44	2.65
2.19	-10.13	-5.35	-4.12	-1.61	52.28	2.7
-0.91	-2.98	-3.62	-2.11	-1.61	90.35	1.49
3.99	-1.8	-6.34	-1.68	-1.6	98.55	3.96
-2.96	-15.01	-1.48	-5.08	-1.6	35.24	0.65
-2.55	-13.13	-3.14	-4.72	-1.6	41.09	1.31
-2.05	-8.36	-7.25	-3.7	-1.6	59.96	5.77
2.96	-10.02	-6.37	-4.1	-1.6	52.68	4.03
-1.89	-9.23	-3.11	-3.92	-1.6	56.06	1.24
-1.37	-11.18	-6.6	-4.34	-1.6	48.04	4.42
-1.68	-11.16	-7.14	-4.34	-1.6	48.15	5.34
-1.75	-11.14	-7.26	-4.33	-1.6	48.21	5.64
-1.16	-10.32	-7.34	-4.15	-1.6	51.46	5.24
0.48	-4.39	-2.51	-2.58	-1.6	81.28	0.98
-0.11	-4.38	-3.12	-2.57	-1.6	81.36	1.24
-0.65	-3.02	-3.37	-2.12	-1.6	90.08	1.39
-0.36	-4.3	-4.01	-2.54	-1.59	81.77	1.84
-0.6	-4.41	-3.69	-2.58	-1.59	81.11	1.57
-0.34	-2.89	-4.74	-2.06	-1.59	90.86	2.56
-1.7	-11.59	-7.6	-4.43	-1.59	46.49	6.38
-1.68	-9.6	-5.08	-4	-1.59	54.46	2.71
0.35	-6.36	-6.98	-3.16	-1.59	69.98	5.23
-2.66	-8.18	-2.56	-3.64	-1.59	60.8	0.97
-0.47	-4.38	-2.77	-2.57	-1.59	81.31	1.08
0.7	-3.31	-3.26	-2.21	-1.59	88.06	1.42
-0.15	-4.79	-3.82	-2.7	-1.58	78.77	1.67
-2.32	-4.06	-4.21	-2.45	-1.58	83.17	1.8
-0.67	-5.32	-4.23	-2.84	-1.58	75.61	1.81
-1.97	-12.86	-3.77	-4.68	-1.58	41.97	1.92
-0.15	-4.71	-4.57	-2.66	-1.57	79.18	2.15
-2.37	-3.58	-4.13	-2.28	-1.57	86.14	1.82
-0.16	-6.26	-2.72	-3.11	-1.57	70.4	1.09

-0.25	-6.21	-2.92	-3.1	-1.57	70.62	1.17
-2.1	-12.33	-3.52	-4.57	-1.57	43.75	1.78
-1.49	-8.23	-4.97	-3.65	-1.56	60.44	2.59
-1.22	-11.17	-5.19	-4.33	-1.56	47.99	2.46
12.8	-18.24	-7.21	-5.49	-1.56	26.82	5.27
-0.55	-6.1	-2.25	-3.06	-1.56	71.19	0.84
1.73	-4.71	-2.08	-2.65	-1.56	79.08	0.82
0.31	-4.38	-2.64	-2.55	-1.56	81.1	1.08
-1.05	-3.05	-3.02	-2.1	-1.56	89.54	1.21
-2.49	-18.21	-5.02	-5.5	-1.55	26.88	2.58
1.18	-2.95	-6.83	-2.05	-1.55	90.2	4.53
-1.77	-9.21	-5.13	-3.88	-1.55	55.96	2.91
3.99	-19.11	-6.97	-5.59	-1.55	24.91	4.64
-1.27	-11.41	-6.74	-4.36	-1.55	47.01	4.68
-0.39	-6.17	-2.96	-3.07	-1.55	70.78	1.2
-2.06	-8.6	-4.4	-3.73	-1.55	58.69	1.84
0.87	-4.28	-2.92	-2.51	-1.55	81.67	1.13
-0.56	-2.61	-6.15	-1.94	-1.54	92.43	4.07
-0.11	-11.93	-6.37	-4.47	-1.53	45.06	3.79
0.15	-4.3	-3.67	-2.5	-1.53	81.38	1.58
0	-4.3	-3.79	-2.5	-1.53	81.43	1.68
-1.79	-14.83	-2.97	-4.98	-1.53	35.58	1.21
2.9	-3.97	-5.64	-2.39	-1.53	83.43	3.15
-1.92	-11	-3.24	-4.26	-1.53	48.54	1.18
-1.75	-11.09	-7	-4.28	-1.53	48.17	5.36
-0.35	-4.29	-3.35	-2.5	-1.53	81.46	1.39
1.69	-10.42	-6.97	-4.13	-1.52	50.77	4.61
1.12	-16.79	-6.4	-5.28	-1.52	30.24	4.44
-2.53	-7.74	-3.06	-3.49	-1.52	62.61	1.34
-1.75	-6.71	-5.16	-3.21	-1.52	67.78	2.97
-1.61	-14.85	-3.39	-4.97	-1.51	35.49	1.43
0.11	-1.95	-7.65	-1.67	-1.51	96.77	6.24
-2.22	-4.1	-3.73	-2.42	-1.51	82.51	1.74
-2.29	-6.19	-3.49	-3.05	-1.51	70.47	1.6
-2.4	-6.02	-3.14	-3	-1.51	71.37	1.35
-2	-7.71	-4.08	-3.47	-1.51	62.67	1.89
-0.54	-4.47	-2.84	-2.55	-1.51	80.24	1.18
-0.07	-4.34	-5.44	-2.49	-1.5	80.98	3.24
-0.6	-3.33	-5.18	-2.16	-1.5	87.34	2.93
-2.26	-4.04	-4.07	-2.39	-1.5	82.81	1.89
-2.02	-3.82	-4.34	-2.32	-1.5	84.18	2.2
3.56	-12.77	-9.07	-4.6	-1.5	42.01	10.55
-2.6	-9.07	-4	-3.81	-1.5	56.36	1.88
0.21	-4.71	-4.62	-2.61	-1.49	78.74	2.35

0.27	-4.77	-5	-2.63	-1.49	78.38	2.84
-2.56	-12.02	-3.21	-4.44	-1.49	44.6	1.49
0.57	-2.04	-5.47	-1.7	-1.49	96.07	3.25
-1.14	-10.97	-6.37	-4.22	-1.49	48.46	4.25
-0.85	-6.52	-3.85	-3.14	-1.49	68.64	1.63
5.33	-1.72	-3.21	-1.58	-1.49	98.36	1.16
3.49	-17.48	2.02	-2.91	-1.48	21.84	0.76
-1.39	-8.18	-3.78	-3.58	-1.48	60.35	1.84
-2	-5.1	-4.57	-2.72	-1.48	76.37	2.35
1.8	-1.77	-6.04	-1.58	-1.48	97.85	3.77
-2.83	-13.05	-4.4	-4.63	-1.48	40.99	2.26
2.79	-12.84	-9	-4.59	-1.48	41.69	10.37
-2.64	-18.1	-4.47	-5.43	-1.47	26.98	2.24
2.3	-7.64	-3.87	-3.43	-1.47	62.87	1.56
-1.83	-10.05	-5.07	-4.02	-1.47	52.08	2.52
-2.06	-3.68	-4.26	-2.25	-1.47	84.93	1.94
3.26	-12.9	-8.68	-4.6	-1.47	41.49	9.46
-2.96	-9.55	-4.64	-3.89	-1.47	54.14	2.15
-2.4	-18.43	-4.28	-5.46	-1.46	26.22	2.29
0.22	-20.86	-5.63	-5.73	-1.46	21.29	3.48
1.19	-11.96	-8.76	-4.41	-1.46	44.69	9.08
-0.4	-4.29	-2.15	-2.45	-1.46	81.07	0.84
-3.95	-14.9	-6.61	-4.97	-1.45	35.17	4.52
1.17	-13.09	1.51	-2.2	-1.45	31.22	1.07
-1.96	-7.77	-6.5	-3.45	-1.45	62.13	4.91
0.11	-12.15	-2.52	-4.43	-1.45	44	0.89
0.5	-6.79	-6.73	-3.19	-1.45	66.98	5.3
-0.49	-6.04	-3.63	-2.98	-1.45	70.94	1.57
14.42	-18.71	-6	-5.48	-1.45	25.58	3.97
0.3	-3.38	-3.58	-2.14	-1.45	86.7	1.62
-1.75	-11.04	-7.07	-4.21	-1.45	48.07	5.72
-1.71	-4.23	-4.05	-2.42	-1.45	81.37	1.8
-2.06	-3.37	-3.92	-2.14	-1.45	86.72	1.83
-2.08	-3.06	-4.1	-2.03	-1.45	88.79	1.74
-1.6	-11.97	-7.55	-4.41	-1.44	44.58	6.65
-1.65	-14.84	-3.05	-4.92	-1.44	35.35	1.3
-3.43	-13.47	-6.26	-4.7	-1.44	39.53	4.06
-2.43	-13.42	-2.86	-4.67	-1.44	39.67	1.27
-2.5	-13.3	-3	-4.64	-1.44	40.06	1.28
-2.49	-13.22	-2.89	-4.63	-1.44	40.35	1.21
-1.18	-12.8	-3.24	-4.56	-1.44	41.73	1.46
-2.66	-12.22	-2.35	-4.44	-1.44	43.71	1
-0.44	-5.2	-7.84	-2.72	-1.44	75.59	7.07
-1.34	-10.27	-4.07	-4.06	-1.44	51.04	2.05

-2.62	-8.86	-2.93	-3.72	-1.44	57.07	1.28
-1.97	-3.96	-3.68	-2.32	-1.44	82.96	1.95
-1.71	-8.08	-6.93	-3.53	-1.44	60.61	5.45
-0.39	-4.07	-4.16	-2.37	-1.43	82.25	1.95
-1.28	-16.84	-2.1	-4.65	-1.43	28.14	1.28
-2.76	-11.08	-3.26	-4.21	-1.43	47.84	1.43
1.04	-3.11	-3.64	-2.04	-1.43	88.38	1.65
-3.16	-15.67	-4.22	-5.05	-1.42	32.93	2.08
-3.26	-15.28	-3.71	-5	-1.42	34.04	1.56
7.21	-3.93	-5.12	-2.31	-1.42	83.08	2.71
-1.93	-14.53	-2.17	-4.85	-1.42	36.19	0.96
0.48	-1.99	-4.94	-1.64	-1.42	95.89	2.79
-0.9	-11.3	-6.08	-4.24	-1.42	46.95	3.97
-2.04	-3.76	-3.94	-2.24	-1.42	84.07	1.78
-2.71	-11.23	-3.57	-4.23	-1.42	47.27	1.67
-2.69	-11.51	-3.88	-4.29	-1.42	46.23	1.83
0.9	-4.6	-4.66	-2.51	-1.42	79	2.18
-0.67	-10.3	-6.74	-4.03	-1.42	50.87	4.75
-2.57	-8.9	-3.92	-3.72	-1.42	56.79	1.79
-2.56	-9.1	-3.82	-3.77	-1.42	55.92	1.95
0.86	-3.1	-3.07	-2.02	-1.42	88.36	1.28
-2.27	-11.52	-3.85	-4.28	-1.41	46.12	1.67
-1.66	-11.47	-1.85	-4.29	-1.41	46.29	0.95
-1.49	-10.23	-4.38	-4.03	-1.41	51.13	2.16
-1.42	-10.21	-4.51	-4.03	-1.41	51.21	2.25
-0.08	-6.18	-2.86	-2.99	-1.41	69.97	1.26
0.29	-8.77	-3.83	-3.67	-1.4	57.25	1.62
-2.53	-18.14	-4.53	-5.39	-1.4	26.75	2.34
-3.13	-15.21	-4.89	-4.98	-1.4	34.16	2.8
0.2	-5.43	-4.75	-2.77	-1.4	74.07	2.59
-3.1	-14.63	-2.63	-4.88	-1.4	35.83	1.24
-1.96	-5.05	-4.49	-2.65	-1.4	76.22	2.32
-1.92	-5.09	-4.53	-2.66	-1.4	75.98	2.42
0.63	-3.49	-5.41	-2.15	-1.4	85.72	3.23
0.72	-3.52	-5.86	-2.16	-1.4	85.5	3.8
-0.15	-13.13	-5.28	-4.6	-1.39	40.47	2.83
-3.19	-15.39	-5.2	-5	-1.39	33.65	3.14
0.18	-1.79	-7.58	-1.54	-1.39	97.06	6.38
3.85	-12.7	-9.16	-4.51	-1.39	41.88	10.69
-2.02	-12.5	-4.84	-4.49	-1.39	42.58	2.34
-0.27	-2.77	-3.03	-1.9	-1.39	90.36	1.23
5.85	-13.07	0.89	-2.18	-1.38	31.25	1.4
-0.51	-2.56	-6.14	-1.82	-1.38	91.65	4.39
-0.45	-4.46	-2.54	-2.46	-1.38	79.53	1.1

4.92	-1.52	-6.69	-1.43	-1.37	98.86	4.96
-1.76	-7.7	-2.37	-3.37	-1.37	62.09	0.93
-1.63	-7.73	-6.8	-3.39	-1.37	61.95	5.48
0.31	-12.98	-6.83	-4.55	-1.36	40.86	5.02
5.56	-4.93	-4.14	-2.59	-1.36	76.7	3.16
-1.67	-14.86	-2.59	-4.87	-1.36	35.06	1.13
0.44	-3.4	-3.16	-2.09	-1.36	86.01	1.39
18.43	-6.78	15.47	6.17	-1.35	21.85	1.27
16.22	-11.66	8.9	2.14	-1.35	21.86	1.35
-0.56	-3.23	-4.78	-2.03	-1.35	87.03	2.71
0.37	-5.59	-4.15	-2.79	-1.35	72.91	2.49
-2.33	-14.46	-3.13	-4.81	-1.35	36.23	1.45
-2.33	-11.74	-3.15	-4.29	-1.35	45.12	1.49
-2.5	-11.74	-3.14	-4.29	-1.35	45.14	1.37
-0.53	-11.97	-3.44	-4.34	-1.35	44.3	1.59
0.33	-3.53	-4.16	-2.13	-1.35	85.13	2.2
-2.13	-3.85	-3.51	-2.24	-1.35	83.07	1.62
-2.23	-6.08	-3.37	-2.91	-1.35	70.2	1.68
0.04	-6.14	-2.37	-2.94	-1.35	69.9	1.05
-0.81	-5.33	-0.68	-2.7	-1.35	74.35	0.51
0.29	-3.16	-2.83	-2.01	-1.35	87.5	1.19
-0.17	-3.02	-2.71	-1.95	-1.35	88.41	1.21
6.75	-14.69	5.05	-0.49	-1.34	21.87	1.15
6.06	-14.71	5.07	-0.54	-1.34	21.96	1.13
-0.12	-2.54	-3.96	-1.78	-1.34	91.55	1.79
-3.24	-15.57	-3.71	-4.99	-1.34	33	1.75
0.47	-1.94	-4.79	-1.57	-1.34	95.66	2.73
-0.97	-18.58	-3.37	-5.38	-1.34	25.66	1.6
-2.56	-11.64	-3.89	-4.26	-1.34	45.44	1.82
-2.47	-11.66	-4	-4.26	-1.34	45.38	1.95
4.21	-3.57	-1.1	-2.14	-1.34	84.8	0.44
23.64	-6.53	15.95	6.34	-1.33	21.97	1.1
-0.23	-2.81	-4.72	-1.87	-1.33	89.71	2.57
-0.18	-2.89	-4.76	-1.9	-1.33	89.19	2.69
-0.39	-11.67	-3.88	-4.27	-1.33	45.32	1.93
1.46	-15.33	-6.49	-4.93	-1.33	33.66	4.95
0.85	-3.48	-5.19	-2.1	-1.33	85.27	3.09
-2.47	-7.69	-2.71	-3.34	-1.33	61.93	1.24
-2.12	-3.84	-3.27	-2.21	-1.33	83.02	1.51
-3.09	-15.58	-4.81	-4.98	-1.32	32.94	2.64
0.46	-21.1	-4.91	-5.66	-1.32	20.63	2.97
-0.92	-11.94	-4.67	-4.32	-1.32	44.34	2.52
-1.88	-5.05	-4.76	-2.59	-1.32	75.73	2.7
0.94	-3.53	-5.18	-2.11	-1.32	84.95	3.23

-1.68	-6.56	-5.06	-3.03	-1.32	67.5	3.01
0.14	-4.22	-5.7	-2.33	-1.31	80.54	3.83
-3.56	-14.57	-6.22	-4.82	-1.31	35.79	4.18
1.9	-2.95	-7	-1.91	-1.31	88.64	5.42
1.23	-12.79	-2.08	-4.46	-1.31	41.37	0.97
-2.86	-12.35	-1.37	-4.39	-1.31	42.86	0.68
-2.06	-10.63	-6.04	-4.03	-1.3	49.12	4.37
-2.44	-17.01	-5.45	-5.15	-1.3	29.21	3.68
-2.41	-7.66	-1.84	-3.32	-1.3	61.97	0.89
1.88	-18.54	-6.98	-5.35	-1.3	25.66	5.81
-3.32	-13.25	-6.05	-4.56	-1.29	39.78	3.98
-1.78	-11.7	-3	-4.24	-1.29	45.07	1.45
13.92	-18.54	-6.12	-5.34	-1.29	25.66	4.08
-1.71	-11.17	-7.08	-4.13	-1.29	47	6.15
-2.44	-7.63	-2.28	-3.31	-1.29	62.08	1.01
-2.39	-13.9	-3.35	-4.67	-1.29	37.75	1.64
-0.14	-4.46	-3.36	-2.4	-1.28	78.93	1.61
0.4	-4.7	-3.73	-2.47	-1.28	77.59	1.91
-1.5	-12.36	-6.96	-4.38	-1.28	42.74	5.88
3.09	-3.74	-5.28	-2.16	-1.28	83.34	2.95
-0.33	-4.96	-7.74	-2.54	-1.28	76.09	7.03
-2.44	-7.61	-2.53	-3.3	-1.28	62.14	1.1
6.6	-21.17	-3.84	-5.63	-1.28	20.45	2.18
-0.02	-4.48	-2.77	-2.4	-1.28	78.88	1.15
-2.21	-4.01	-3.06	-2.24	-1.28	81.69	1.39
-2.16	-3.84	-3.03	-2.18	-1.28	82.74	1.38
0.59	-3.18	-1.91	-1.97	-1.28	86.97	0.91
-3.09	-15.27	-4.81	-4.9	-1.27	33.7	2.84
-0.16	-11.52	-4.75	-4.2	-1.27	45.68	2.74
-2.39	-11.7	-3.16	-4.23	-1.27	45.01	1.44
0.5	-1.91	-4.87	-1.51	-1.27	95.35	2.91
-2.53	-7.64	-3.15	-3.3	-1.27	61.92	1.47
-2.09	-3.82	-3.36	-2.18	-1.27	82.79	1.65
-0.16	-5.14	-3.52	-2.59	-1.27	75	1.57
-0.72	-5.81	-1.19	-2.79	-1.27	71.26	0.65
0.03	-4.45	-3.15	-2.39	-1.27	78.99	1.45
15.2	-11.85	8.34	1.93	-1.26	21.99	1.47
3.8	-12.68	5.18	-0.93	-1.26	28.12	0.54
-1.19	-8.73	-3.75	-3.58	-1.26	56.85	1.99
-3.14	-15.18	-3.62	-4.88	-1.26	33.91	1.74
-2.61	-9.12	-2.89	-3.66	-1.26	55.17	1.3
4.56	-20.03	-6.26	-5.5	-1.26	22.53	4.04
-0.48	-11.01	-5.99	-4.08	-1.26	47.5	3.97
-2.55	-7.58	-3.23	-3.28	-1.26	62.2	1.42

-0.76	-19.21	-3.85	-5.4	-1.26	24.17	1.99
-1.8	-7.7	-3.96	-3.31	-1.26	61.61	2.1
-0.18	-3	-2.26	-1.89	-1.26	87.94	1.06
-0.92	-10.82	-4.99	-4.05	-1.25	48.22	3.08
6.65	-14.85	-8.05	-4.79	-1.25	34.81	9.19
-2.21	-14.23	-3.89	-4.71	-1.25	36.65	1.9
0.39	-3.28	-2.07	-1.98	-1.25	86.04	0.95
-3.13	-15.35	-4.54	-4.89	-1.24	33.37	2.41
-3	-12.13	-1.28	-4.31	-1.24	43.39	0.65
-3	-12.08	-1.35	-4.29	-1.24	43.57	0.69
2.52	-9.64	-5.37	-3.76	-1.24	52.89	3.05
-0.62	-5.97	-2.46	-2.82	-1.24	70.26	1.04
2.55	-12.35	-8.75	-4.34	-1.24	42.63	9.52
-1.58	-6.58	-5.16	-2.98	-1.24	67.02	3.21
-0.22	-2.71	-1.95	-1.78	-1.24	89.72	0.85
-3.09	-15.42	-4.89	-4.89	-1.23	33.17	3.01
7.71	-3.81	-5.36	-2.15	-1.23	82.64	3.25
-3.22	-11.06	-0.88	-4.08	-1.23	47.24	0.57
-1.61	-7.93	-5.25	-3.36	-1.23	60.37	3.07
-3.17	-10.89	-1.48	-4.04	-1.22	47.82	0.78
-1.54	-4.2	-3.56	-2.26	-1.22	80.18	1.6
-2.52	-11.76	-3.26	-4.21	-1.22	44.61	1.55
0.06	-2.55	-4.16	-1.7	-1.21	90.61	2.3
-1.77	-7.8	-6.75	-3.3	-1.21	60.88	5.76
1.63	-16.34	-6.36	-5	-1.21	30.67	4.98
-0.19	-11.58	-6.19	-4.16	-1.21	45.25	4.39
-1.39	-7.52	-2.76	-3.22	-1.21	62.21	1.19
-0.51	-3.06	-3.74	-1.87	-1.2	87.13	1.95
0.87	-13.06	1.94	-2.19	-1.2	31.28	0.87
1.28	-2.86	-3.88	-1.81	-1.2	88.48	1.9
-0.42	-5.24	-8.41	-2.57	-1.2	74.01	9.78
0.34	-1.53	-6.84	-1.33	-1.2	97.6	5.55
-0.7	-6.2	-0.67	-2.86	-1.2	68.82	0.57
-3.03	-14.9	-3.71	-4.78	-1.19	34.52	1.88
2.07	-1.46	-5.61	-1.29	-1.19	98.03	3.62
-0.58	-12.98	-3.31	-4.42	-1.19	40.36	1.55
0.48	-3.44	-4.85	-2	-1.19	84.71	2.93
4.79	-20.5	-6.3	-5.5	-1.19	21.52	4.69
-0.48	-2.5	-6.42	-1.67	-1.19	90.81	5.16
-2.4	-7.64	-2.98	-3.25	-1.19	61.57	1.39
-2.36	-13.52	-3.25	-4.52	-1.19	38.65	1.67
-0.5	-17.12	-3.54	-5.09	-1.19	28.71	1.86
-0.24	-6.4	-3.63	-2.91	-1.19	67.72	1.71
-3.26	-14.19	-6.12	-4.67	-1.18	36.55	4.3

2.82	-1.37	-6.13	-1.25	-1.18	98.58	4.6
0.69	-8.35	-6.05	-3.43	-1.18	58.24	4.87
-0.78	-12.79	-3.67	-4.39	-1.18	40.97	1.88
0.11	-11.46	-6.1	-4.12	-1.18	45.58	4.39
0.06	-4.97	-3.45	-2.48	-1.18	75.44	1.65
6.71	-20.53	-4.18	-5.49	-1.18	21.46	1.99
-2.03	-7.47	-4.21	-3.19	-1.18	62.32	2.02
-2.64	-11.45	-3.56	-4.12	-1.18	45.61	1.73
-2.58	-11.52	-3.6	-4.13	-1.18	45.35	1.85
-2.22	-11.66	-3.95	-4.16	-1.18	44.88	2.25
-1.79	-16.27	-4.65	-4.96	-1.17	30.77	2.48
2.47	-1.41	-6.01	-1.26	-1.17	98.27	4.28
1.04	-3.47	-5.24	-2	-1.17	84.36	3.41
-2.58	-12.1	-4.05	-4.24	-1.17	43.28	2.17
-2.09	-8.84	-4.39	-3.54	-1.17	56	1.67
-1.46	-7.61	-4.89	-3.22	-1.17	61.61	2.97
-0.9	-2.9	-0.84	-1.8	-1.17	88.07	0.64
1.25	-3.88	-3.44	-2.13	-1.16	81.75	1.59
-1.01	-10.59	-4.62	-3.94	-1.16	48.8	2.55
0.51	-3.36	-5.58	-1.95	-1.16	84.96	3.86
-1.28	-6.63	-5.38	-2.95	-1.16	66.38	3.8
0.63	-3.2	-1.47	-1.9	-1.16	86.01	0.71
0.6	-3.12	-1.8	-1.88	-1.16	86.53	0.82
-1.44	-7.75	-5.13	-3.25	-1.16	60.94	3.22
0.48	-4.06	-5.53	-2.18	-1.15	80.63	3.81
2.71	-17.81	-7.1	-5.15	-1.15	27.01	6.34
2.92	-18.42	-7.28	-5.23	-1.15	25.65	6.81
-1.67	-7.93	-5.03	-3.31	-1.15	60	3.04
-2.01	-10.57	-5.8	-3.91	-1.14	48.75	4.16
-0.64	-2.44	-3.35	-1.62	-1.14	90.86	1.71
1.83	-12.91	1.56	-2.08	-1.14	31.3	1.32
-1.03	-2.88	-3.94	-1.78	-1.14	87.97	2.25
-0.96	-14.08	-2.6	-4.59	-1.14	36.76	1.33
-2.58	-11.98	-3.83	-4.2	-1.14	43.61	2.06
0.49	-4.6	-2.83	-2.35	-1.14	77.33	1.34
0.53	-4.66	-3.13	-2.36	-1.13	76.92	1.57
2.03	-16.4	-6.04	-4.94	-1.13	30.35	4.22
-0.6	-2.48	-3.57	-1.63	-1.13	90.53	1.85
-1.38	-12.73	-6.83	-4.35	-1.13	41.01	6.06
0.52	-4.68	-7.63	-2.36	-1.13	76.84	7.28
-2.56	-9.16	-2.53	-3.59	-1.13	54.46	1.26
0.19	-6	-1.34	-2.75	-1.13	69.49	0.75
-1.42	-16.46	-3.52	-4.95	-1.12	30.17	1.79
3.6	-10.77	3.81	-0.17	-1.12	31.33	1.43

-0.27	-2.6	-3.49	-1.66	-1.12	89.72	1.73
-0.64	-12.27	-4.51	-4.25	-1.12	42.53	2.55
-1.66	-11.59	-3.1	-4.11	-1.12	44.92	1.49
0.56	-1.8	-3.93	-1.38	-1.12	95.14	2.12
-1.24	-10.34	-3.66	-3.86	-1.12	49.58	2.32
1.79	-16.12	-6.36	-4.91	-1.12	31.04	5.15
0.3	-3.43	-4.15	-1.95	-1.12	84.34	2.35
1.79	-14.93	-6.39	-4.72	-1.11	34.23	4.99
-1.41	-6.74	-4.19	-2.96	-1.11	65.57	2.47
-0.45	-3.02	-3.8	-1.8	-1.11	86.85	2.03
0.6	-1.82	-4.11	-1.38	-1.11	94.95	2.37
-0.27	-11.34	-3.34	-4.06	-1.11	45.79	1.74
0.41	-3.38	-4.27	-1.92	-1.11	84.59	2.34
4.73	-20.07	-6.12	-5.4	-1.11	22.2	4.25
0.14	-6.18	-3.88	-2.8	-1.11	68.43	2.01
1.28	-4.85	-4.13	-2.4	-1.11	75.75	2.03
-1.99	-3.08	-0.83	-1.81	-1.11	86.49	0.62
0.4	-2.96	-0.49	-1.79	-1.11	87.24	0.46
-0.13	-2.59	-4.35	-1.65	-1.1	89.65	2.52
-1.41	-10.19	-1.13	-3.8	-1.1	50.13	0.69
-0.71	-13.66	-3.58	-4.49	-1.1	37.95	1.84
1.14	-3.45	-4.37	-1.94	-1.1	84.07	2.53
1.36	-3.55	-5.25	-1.98	-1.1	83.42	3.66
0.41	-11.68	-5.97	-4.11	-1.1	44.55	4.4
-2.17	-6.08	-3.6	-2.75	-1.1	68.95	1.88
6.13	-14.18	-7.39	-4.58	-1.1	36.38	8.01
-1.74	-7.67	-3.8	-3.2	-1.1	61.07	1.89
0.51	-4.64	-2.46	-2.35	-1.1	76.9	1.18
-2.58	-9.1	-3.22	-3.56	-1.1	54.61	1.55
-0.36	-3.06	-4.71	-1.81	-1.09	86.47	2.92
-1.05	-8.76	-4.46	-3.48	-1.09	56.02	2.75
3.51	-4.01	-4.37	-2.12	-1.09	80.57	2.86
-1.75	-5.07	-4.28	-2.46	-1.09	74.37	2.57
1.24	-3.44	-5.09	-1.94	-1.09	84.02	3.31
6.63	-20.38	-4.12	-5.42	-1.09	21.59	1.81
6.33	-14.61	-8.15	-4.64	-1.09	35.07	9.36
-3.04	-9.49	-3.61	-3.63	-1.09	52.94	1.93
-1.39	-7.36	-4.64	-3.1	-1.09	62.48	2.88
-3.13	-15.54	-4.45	-4.81	-1.08	32.49	2.64
-3.15	-15.55	-4.51	-4.81	-1.08	32.46	2.6
-3.06	-15.29	-4.78	-4.77	-1.08	33.15	2.7
0.67	-21.69	-4.78	-5.56	-1.08	19.24	3.03
-3.03	-11.28	-0.7	-4.03	-1.08	45.88	0.54
-1.46	-10.28	-1.64	-3.8	-1.08	49.68	0.88

-0.64	-6.07	-2.2	-2.74	-1.08	68.87	1.02
6.93	-14.56	-8.05	-4.63	-1.08	35.22	9.17
-2.03	-12.55	-4.34	-4.29	-1.08	41.46	2.26
0.73	-4.01	-5.52	-2.11	-1.07	80.41	4.02
5.38	-1.19	-6.63	-1.12	-1.07	99.07	5.38
0.44	-12.35	-1.35	-4.22	-1.07	42.08	0.62
3.29	-17.84	-6.84	-5.1	-1.07	26.78	5.87
3.49	-18.37	-7.28	-5.17	-1.07	25.62	6.87
-1.94	-2.97	-0.84	-1.76	-1.07	86.89	0.64
-0.62	-2.39	-2.76	-1.56	-1.07	90.77	1.36
6.09	-1.26	-3	-1.14	-1.07	98.64	1.37
0.2	-2.38	-3.71	-1.55	-1.06	90.74	1.92
3.66	-10.78	4.44	-0.19	-1.06	31.33	1.09
-2.36	-9.04	-1.59	-3.51	-1.06	54.73	0.89
-1.22	-8.97	-2.86	-3.52	-1.06	55.01	1.44
-0.45	-2.57	-6.06	-1.62	-1.06	89.55	4.86
0.54	-4.62	-2.75	-2.3	-1.06	76.78	1.39
-0.67	-2.36	-3.19	-1.54	-1.05	90.86	1.59
-0.14	-2.66	-4.34	-1.64	-1.05	88.83	2.64
-3	-11.17	-1.27	-3.99	-1.05	46.23	0.68
-3.06	-10.83	-1.61	-3.92	-1.05	47.5	0.84
-3.04	-10.79	-1.71	-3.9	-1.05	47.62	0.87
-2.39	-9.04	-1.56	-3.5	-1.05	54.67	0.82
1.99	-17.15	-6.51	-5.01	-1.05	28.33	5.7
-1.28	-6.82	-6.71	-2.94	-1.05	64.89	5.98
-1.32	-8.87	-3.57	-3.48	-1.04	55.35	1.92
-1.29	-8.92	-3.53	-3.5	-1.04	55.13	1.87
-0.64	-2.34	-4.03	-1.52	-1.04	90.92	2.27
-0.12	-2.52	-4.29	-1.59	-1.04	89.69	2.45
-1.82	-14.47	-2.36	-4.59	-1.04	35.37	1.04
4.93	-10.36	-6.31	-3.8	-1.04	49.23	4.9
0.15	-4.62	-3.71	-2.29	-1.04	76.65	2
0.36	-3.38	-3.92	-1.89	-1.04	84.15	2.14
-1.96	-3.83	-2.91	-2.04	-1.04	81.38	1.59
0.34	-4.97	-3.66	-2.39	-1.04	74.68	1.88
-2.31	-13.74	-3.21	-4.46	-1.04	37.54	1.78
-0.22	-17.23	-3.7	-5	-1.04	28.12	2.08
0.04	-2.31	-3.11	-1.52	-1.04	91.14	1.49
-1.34	-8.8	-3.77	-3.46	-1.03	55.64	2.07
-2.68	-14.89	-2.99	-4.68	-1.03	34.15	1.62
-1.57	-10.01	-3.78	-3.73	-1.03	50.57	2.12
0.1	-4.45	-3.53	-2.23	-1.03	77.63	2.04
-2.49	-9.17	-2.7	-3.53	-1.03	54.03	1.42
5.08	-20.19	-4.15	-5.35	-1.03	21.84	2.88

1.02	-12.98	-6.91	-4.33	-1.02	39.88	5.95
-0.11	-2.49	-4.55	-1.56	-1.02	89.75	2.72
3.21	-1.23	-6.03	-1.1	-1.02	98.52	4.53
0.63	-1.77	-4.8	-1.3	-1.02	94.67	3.08
-2.4	-9.16	-2.49	-3.51	-1.02	54.03	1.29
-0.19	-10.68	-3.3	-3.86	-1.02	47.94	1.84
0.67	-11.42	-6	-4	-1.02	45.2	4.58
-3.06	-9.53	-3.31	-3.59	-1.02	52.5	1.71
-0.18	-2.76	-0.43	-1.66	-1.02	88.01	0.49
-0.4	-2.95	-4.4	-1.72	-1.01	86.63	2.58
-0.3	-2.86	-4.56	-1.69	-1.01	87.24	2.85
-0.08	-2.42	-4.73	-1.53	-1.01	90.12	2.96
0.91	-22.23	-4.35	-5.56	-1.01	18.27	2.77
-1.49	-10.03	-4.37	-3.72	-1.01	50.43	2.38
-1.35	-8.42	-2.32	-3.32	-1.01	57.21	1.22
7.52	-17.92	-7.55	-5.08	-1.01	26.48	8.19
-3.01	-9.46	-3.65	-3.57	-1.01	52.7	1.87
-1.4	-7.2	-4.36	-3.01	-1.01	62.89	2.67
-0.83	-3.97	-3.1	-2.05	-1	80.27	1.52
-0.63	-6.2	-1.49	-2.73	-1	67.85	0.77
7.38	-17.69	-7.74	-5.04	-1	27	8.32
-0.92	-13.54	-1.71	-4.39	-0.99	38.02	0.95
0.67	-1.78	-4.77	-1.29	-0.99	94.37	3.13
0.33	-5.66	0.18	-2.57	-0.99	70.62	0.4
-1.72	-7.93	-4.69	-3.2	-0.99	59.31	2.82
-2.02	-3.35	-1.98	-1.83	-0.99	84	1.04
-0.21	-2.54	-2.77	-1.56	-0.99	89.21	1.4
-1.76	-5.07	-4.49	-2.38	-0.98	73.77	2.9
-1.07	-10.47	-4.8	-3.8	-0.98	48.58	3.01
-1.76	-9.04	-2.02	-3.48	-0.98	54.38	1.05
-1.43	-8.41	-2.47	-3.3	-0.98	57.1	1.28
-0.4	-2.64	-5.36	-1.59	-0.98	88.49	4.02
-2.2	-7.64	-2.78	-3.1	-0.98	60.62	1.48
2.81	-10.43	-4.56	-3.77	-0.98	48.75	2.6
4.22	-12.17	-8.62	-4.13	-0.98	42.44	9.58
-0.95	-2.74	-2.72	-1.63	-0.98	87.83	1.36
-0.49	-2.54	-1.91	-1.56	-0.98	89.16	1.02
0.63	-4.72	-3.05	-2.29	-0.97	75.71	1.66
0.36	-12.7	1.46	-1.9	-0.97	31.41	1.34
0.26	-4.66	-3.63	-2.26	-0.97	76.01	1.96
-2.38	-9.11	-2.14	-3.47	-0.97	54.01	1.1
-1.56	-9.02	-2.61	-3.44	-0.97	54.44	1.26
-1.35	-8.6	-2.6	-3.34	-0.97	56.24	1.39
-1.21	-6.68	-6.81	-2.85	-0.97	65.21	6.26

-1.08	-6.36	-6.47	-2.76	-0.97	66.81	5.84
-1.7	-13.11	-3.92	-4.32	-0.97	39.33	2.56
-1.25	-6.69	-3.99	-2.85	-0.96	65.13	2.5
0.4	-12.4	-4.96	-4.17	-0.96	41.59	3.09
-0.8	-11.1	-3.78	-3.92	-0.96	46.13	2.45
3.9	-7.73	-2.09	-3.11	-0.96	60.1	0.99
0.05	-17.96	-4.18	-5.04	-0.96	26.3	2.56
-0.13	-3.04	-3.26	-1.71	-0.96	85.79	1.83
-2.09	-2.82	-2.73	-1.64	-0.96	87.23	1.17
-1.42	-8.81	-3.75	-3.41	-0.95	55.26	2.09
-0.83	-13.86	-2.34	-4.43	-0.95	36.93	1.12
2.15	-2.63	-4.18	-1.58	-0.95	88.42	2.34
0.68	-4.86	-4.19	-2.3	-0.95	74.79	2.45
-2.45	-12.12	-3.23	-4.1	-0.95	42.53	1.75
0.33	-18.33	-4.34	-5.08	-0.95	25.48	2.73
0.53	-10.3	-6.66	-3.71	-0.95	49.15	5.59
-0.3	-2.57	-1.75	-1.55	-0.95	88.83	1.03
-0.07	-2.32	-4.68	-1.45	-0.94	90.31	3.01
-2.74	-15.33	-4.52	-4.68	-0.94	32.72	3.05
0.72	-1.8	-4.76	-1.26	-0.94	93.94	3.21
-1.52	-10	-4.17	-3.67	-0.94	50.3	2.6
-0.26	-3.34	-4.08	-1.81	-0.94	83.74	2.46
-2.02	-3.04	-0.41	-1.7	-0.94	85.66	0.5
-1.96	-3.03	-0.35	-1.7	-0.94	85.77	0.53
-2.19	-18.58	-4.01	-5.12	-0.93	24.9	2.47
-0.27	-2.8	-4.7	-1.62	-0.93	87.1	3.07
-0.34	-2.45	-3.1	-1.49	-0.93	89.42	1.66
2.41	-10.93	-6.51	-3.85	-0.93	46.69	5.07
8.41	-3.58	-4.51	-1.89	-0.93	82.26	2.65
-1.7	-10.86	-2.35	-3.84	-0.93	46.95	1.08
0.72	-8.56	-2.83	-3.31	-0.93	56.27	1.29
0.53	-1.17	-6.07	-1.03	-0.93	98.29	4.85
3.47	-13.05	-4.85	-4.26	-0.93	39.39	3.19
-2.1	-3.32	-1.76	-1.79	-0.93	83.82	0.95
-0.18	-2.53	-1.95	-1.52	-0.93	88.87	1.16
-0.4	-2.44	-3.36	-1.48	-0.92	89.4	1.88
0.03	-2.92	-4.03	-1.65	-0.92	86.27	2.56
-2.02	-7.46	-4.25	-3.02	-0.92	61.22	2.36
-0.25	-2.87	-4.47	-1.63	-0.91	86.55	2.89
3.69	-3.81	-4.69	-1.94	-0.91	80.73	3.09
-1.26	-11.15	-1.98	-3.88	-0.91	45.79	1.14
2.99	-13.95	-3.78	-4.4	-0.91	36.55	2.42
-2.05	-3.32	-2.15	-1.78	-0.91	83.74	1.12
0.84	-3.8	-5.56	-1.94	-0.9	80.76	4.29

5.82	-3.65	-7.78	-1.89	-0.9	81.63	9.01
0.57	-1.13	-5.86	-0.99	-0.9	98.38	4.62
1.98	-5.23	-3.72	-2.38	-0.9	72.47	2.16
0.81	-4.62	-2.86	-2.2	-0.89	75.81	1.67
-1.65	-9.11	-3.26	-3.43	-0.89	53.69	1.94
15.82	-18.1	-5.52	-5.02	-0.89	25.85	3.98
0.77	-19.54	-4.42	-5.18	-0.89	22.86	2.92
-2.24	-11.71	-2.94	-3.98	-0.89	43.74	1.73
1.66	-5.06	-3.69	-2.32	-0.89	73.38	1.99
-2.07	-13.98	-3.97	-4.42	-0.89	36.43	2.06
-1.33	-9.29	-2.66	-3.45	-0.88	52.95	1.41
-2.26	-8.96	-1.52	-3.37	-0.88	54.31	0.98
-2.16	-7.63	-2.49	-3.04	-0.88	60.25	1.37
2.42	-4.2	-1.71	-2.06	-0.88	78.19	0.79
0.22	-12.75	2.22	-1.97	-0.87	31.49	0.9
-2.23	-18.98	-4.97	-5.12	-0.87	23.96	3.58
-1	-6.44	-1.21	-2.73	-0.87	65.98	0.77
-1.09	-6.43	-1.74	-2.73	-0.87	66.01	1.15
-2.27	-13.87	-3.24	-4.37	-0.87	36.7	1.96
-2.15	-6.22	-2.53	-2.65	-0.87	67.11	1.42
-0.08	-2.9	-3.36	-1.62	-0.87	86.09	1.91
-1.37	-11.48	-2.65	-3.91	-0.86	44.44	1.57
2.77	-2.62	-6.54	-1.51	-0.86	87.86	5.33
-0.92	-8.8	-3.12	-3.33	-0.86	54.91	1.85
-1.17	-13.51	-6.8	-4.31	-0.86	37.75	6.59
-2.71	-14.97	-3.75	-4.57	-0.86	33.48	2.49
-0.16	-3.72	-3.09	-1.88	-0.86	80.91	1.62
7.29	-17.63	-6.85	-4.94	-0.86	26.86	6.74
-0.22	-3.16	-3.98	-1.7	-0.86	84.42	2.39
-0.61	-4.84	-1.88	-2.25	-0.86	74.43	1.21
-0.66	-2.85	-1.12	-1.59	-0.86	86.35	0.84
-2.28	-18.62	-4	-5.07	-0.85	24.67	2.42
-0.21	-2.89	-4.71	-1.6	-0.85	86.05	3.19
-0.18	-2.93	-4.5	-1.61	-0.85	85.8	2.98
-0.12	-2.96	-4.56	-1.62	-0.85	85.6	3.03
-0.07	-2.96	-4.86	-1.62	-0.85	85.6	3.5
6.75	-0.96	-6.12	-0.9	-0.85	99.2	5.02
0.53	-13.27	-4.93	-4.27	-0.85	38.46	3.01
15.57	-18.08	-4.71	-4.99	-0.85	25.83	3.68
0.99	-6.99	-2.87	-2.86	-0.85	63.12	1.31
7.35	-16.97	-7.23	-4.84	-0.85	28.35	7.23
7.86	-16.17	-7.26	-4.73	-0.85	30.32	7.55
-1.06	-6.59	-4.86	-2.74	-0.85	65.13	3.43
0.32	-2.2	-2.04	-1.35	-0.85	90.56	1.06

-2.89	-13.91	-6.15	-4.39	-0.84	36.5	4.79
-2.63	-12.67	-0.68	-4.14	-0.84	40.35	0.66
-1.79	-5.04	-4.37	-2.29	-0.84	73.23	2.87
3.32	-2.64	-4.02	-1.51	-0.84	87.62	2.42
-0.3	-3.69	-4.99	-1.87	-0.84	81.02	3.42
0.12	-2.85	-5.38	-1.58	-0.84	86.27	4.18
-2.43	-9.14	-2.92	-3.39	-0.84	53.42	1.57
-0.33	-2.42	-1.86	-1.43	-0.84	89.06	0.99
0.13	-4.36	-4.15	-2.08	-0.83	77.03	2.73
-1.09	-9.07	-2.29	-3.39	-0.83	53.67	1.36
-1.64	-8.16	-0.89	-3.16	-0.83	57.58	0.72
0.63	-1.01	-5.96	-0.9	-0.83	98.69	4.98
0.61	-1.02	-5.66	-0.9	-0.83	98.61	4.45
0.59	-1.03	-5.37	-0.91	-0.83	98.52	3.96
-0.16	-2.53	-1.72	-1.47	-0.83	88.29	0.95
-0.41	-2.42	-1.39	-1.43	-0.83	88.95	0.87
-1.65	-7.33	-6.29	-2.92	-0.82	61.38	5.75
2.11	-12.43	-5.64	-4.06	-0.82	41.06	4.81
-0.09	-6.62	-2.91	-2.73	-0.82	64.78	1.66
2.64	-16.49	-5.91	-4.74	-0.81	29.45	4.4
-1.09	-13.9	-6.7	-4.35	-0.81	36.45	6.57
2.24	-16.01	-6.3	-4.68	-0.81	30.63	5.84
-1.01	-7.95	-2.6	-3.07	-0.81	58.48	1.27
-2.12	-7.68	-2.5	-3.01	-0.81	59.68	1.45
-1.61	-3.05	-1.55	-1.62	-0.81	84.74	0.81
-0.96	-7.07	-2.82	-2.85	-0.81	62.6	1.61
-0.6	-9.48	-3.87	-3.44	-0.81	51.89	2.38
3.74	-17.37	-6.74	-4.86	-0.8	27.32	6.3
0.34	-2.33	-3.92	-1.37	-0.8	89.4	2.42
1.69	-13	1.51	-2.2	-0.8	31.52	1.07
-0.07	-12.24	-2.96	-4.03	-0.8	41.65	1.74
-0.25	-5.21	-7.37	-2.31	-0.8	72.09	8.04
6.13	-20.45	-3.5	-5.23	-0.8	21.01	1.96
4.69	-4.02	-1.48	-1.94	-0.8	78.84	0.76
-0.11	-2.18	-3.82	-1.31	-0.79	90.3	2.3
3.28	-3.57	-3.62	-1.79	-0.79	81.47	1.91
3.41	-0.99	-5.34	-0.87	-0.79	98.54	3.85
0.77	-1.69	-4.55	-1.13	-0.79	93.65	3.27
-1.63	-9.11	-3.86	-3.36	-0.79	53.31	2.39
0.06	-11.32	-2.96	-3.84	-0.79	44.8	1.72
-2.07	-6.2	-2.69	-2.59	-0.79	66.8	1.56
-1.06	-6.35	-1.66	-2.65	-0.79	66.03	0.97
-1.94	-6.14	-2.55	-2.57	-0.79	67.1	1.47
-2.02	-6.11	-2.59	-2.56	-0.79	67.24	1.45

5.97	-20.15	-3.8	-5.19	-0.79	21.56	2.32
-0.36	-2.38	-2.11	-1.38	-0.79	88.94	1.18
2.33	-15.11	-5.84	-4.52	-0.78	32.92	4.58
6.88	-0.89	-5.89	-0.82	-0.78	99.22	5
-1.61	-9.26	-4.13	-3.4	-0.78	52.69	2.72
-1.57	-9.91	-3.73	-3.54	-0.78	50.04	2.26
-1.48	-10.02	-4.16	-3.57	-0.78	49.61	2.93
-0.53	-11.57	-4.07	-3.9	-0.78	43.9	2.6
5.51	-10.56	-5.27	-3.67	-0.78	47.58	3.67
4.3	-8.06	-2.3	-3.08	-0.78	57.84	1.29
-0.2	-5.95	-2.32	-2.52	-0.78	68.04	1.33
5.82	-20.35	-3.79	-5.2	-0.78	21.16	2.32
-0.95	-5.99	-6.02	-2.53	-0.78	67.79	5.32
-0.65	-2.67	-3.89	-1.48	-0.77	86.98	2.51
0.98	-4.93	-4.62	-2.21	-0.77	73.46	3.03
-0.15	-6.59	-2.52	-2.69	-0.77	64.73	1.42
8.16	-16	-7.33	-4.65	-0.77	30.57	8.04
-1.99	-7.52	-3.84	-2.94	-0.77	60.27	2.32
-0.93	-6.53	-4.61	-2.67	-0.77	65.04	3.28
-1.91	-2.86	-1.45	-1.53	-0.77	85.76	0.86
-0.42	-9.6	-3.66	-3.44	-0.77	51.24	2.23
-1.27	-6.15	-3.99	-2.56	-0.77	66.97	2.6
-1.18	-5.8	-3.85	-2.47	-0.77	68.78	2.37
-0.78	-2.6	-1.15	-1.45	-0.77	87.45	0.79
0.94	-4.86	-3.31	-2.19	-0.76	73.77	1.92
-1.88	-8.65	-2	-3.23	-0.76	55.15	1.15
-0.22	-4.9	-6.82	-2.19	-0.76	73.57	6.87
6.36	-20.73	-4.23	-5.23	-0.76	20.46	2.42
6.87	-14.03	-8.02	-4.33	-0.76	35.92	9.14
0.07	-2.86	-3.92	-1.53	-0.76	85.68	2.52
0.16	-2.74	-4.47	-1.5	-0.76	86.46	3.13
1.47	-3.56	-2.72	-1.77	-0.76	81.32	1.39
-1.35	-8.68	-4.94	-3.22	-0.76	55.07	3.31
-1.38	-16.78	-2.34	-4.75	-0.75	28.6	1.56
5.95	-3.56	-7.72	-1.77	-0.75	81.24	9.34
-2.12	-19.05	-4.8	-5.05	-0.75	23.61	3.51
0.67	-0.91	-5.82	-0.82	-0.75	98.81	4.83
3.15	-11.14	-4.69	-3.76	-0.75	45.33	2.99
-0.54	-7.37	-2.71	-2.89	-0.75	60.86	1.59
0.48	-12.26	-2.37	-3.99	-0.74	41.39	1.43
-1.78	-8.73	-2.93	-3.24	-0.74	54.76	1.75
-1.32	-9.84	-2.16	-3.48	-0.74	50.19	1.24
-0.19	-4.81	-6.92	-2.15	-0.74	73.91	7.15
-1.3	-6.25	-2.47	-2.58	-0.74	66.29	1.44

0.09	-2.79	-5.47	-1.5	-0.74	86.02	4.24
-1.93	-3.81	-2.25	-1.84	-0.74	79.73	1.32
-1.29	-6.27	-2.76	-2.58	-0.74	66.18	1.63
0.07	-6.56	-3.26	-2.67	-0.74	64.72	1.9
-0.67	-7.08	-2.71	-2.81	-0.74	62.22	1.56
-0.8	-2.6	-2.49	-1.43	-0.74	87.26	1.42
2.21	-13.43	-6.2	-4.21	-0.73	37.62	5.96
3.19	-16.95	-6.1	-4.75	-0.73	28.15	5.09
-1.1	-6.37	-3.12	-2.61	-0.73	65.63	1.87
-3.19	-13.01	-5.86	-4.14	-0.73	38.95	4.53
-0.41	-11.89	-3.53	-3.93	-0.73	42.59	2.44
0.14	-11.68	-2.66	-3.87	-0.73	43.32	1.56
1.56	-10.76	-5.86	-3.67	-0.73	46.63	4.57
-1.53	-10.96	-6.54	-3.72	-0.73	45.91	6.23
-1.01	-6.96	-2.62	-2.77	-0.73	62.75	1.47
2.63	-5.95	-3.71	-2.48	-0.73	67.75	2.38
0.1	-6.41	-3.53	-2.62	-0.73	65.43	2.15
-0.8	-7.17	-2.83	-2.83	-0.73	61.75	1.64
-1.28	-8.55	-3.56	-3.2	-0.72	55.44	2.13
-0.75	-2.59	-3.26	-1.41	-0.72	87.16	1.9
8.93	-3.43	-3.88	-1.71	-0.72	81.85	2.32
2.74	-13.49	-2.45	-4.19	-0.72	37.44	1.41
-1.53	-13	-2.32	-4.13	-0.72	38.92	1.31
3.71	-0.97	-5.19	-0.81	-0.72	98.16	3.98
-1.45	-10.42	-5.84	-3.6	-0.72	47.85	5.08
-1.92	-6.09	-2.89	-2.51	-0.72	67.01	1.74
-0.37	-6.1	-1.46	-2.52	-0.72	66.95	0.95
-0.28	-5.95	-2.19	-2.47	-0.72	67.72	1.28
2.16	-19.8	-4.03	-5.1	-0.72	22.08	2.79
1.26	-10.22	-6.37	-3.55	-0.72	48.62	5.58
-1.86	-3.74	-1.41	-1.8	-0.72	79.99	0.92
-0.9	-9.28	-4.04	-3.33	-0.72	52.39	2.64
-0.32	-2.32	-1.79	-1.32	-0.72	88.9	1.07
1.44	-12.87	-6.5	-4.1	-0.71	39.34	5.56
-2.62	-14.76	-3.03	-4.44	-0.71	33.71	1.62
1.19	-22.55	-3.74	-5.38	-0.71	17.38	2.45
-1.6	-9.45	-3.97	-3.39	-0.71	51.65	2.64
-1.59	-9.57	-3.83	-3.43	-0.71	51.14	2.45
-1.56	-9.86	-3.89	-3.49	-0.71	50	2.49
-0.48	-6.01	-1.34	-2.49	-0.71	67.37	0.91
-2.27	-12.08	-3.53	-3.93	-0.71	41.89	2.22
5.37	-17.69	-6.55	-4.84	-0.71	26.42	6.69
1.02	-5.24	-4.08	-2.28	-0.7	71.37	2.79
-1.29	-8.65	-3.52	-3.21	-0.7	54.94	2.25

0.11	-2.99	-4.48	-1.54	-0.7	84.46	3.21
-0.57	-2.64	-3.84	-1.42	-0.7	86.75	2.49
-0.57	-2.64	-4.25	-1.42	-0.7	86.67	2.91
-2.68	-15.06	-3.69	-4.48	-0.7	32.87	2.29
-1.81	-5.13	-3.92	-2.23	-0.7	71.95	2.55
-1.8	-5.32	-3.93	-2.28	-0.7	70.96	2.6
-1.57	-10.01	-3.8	-3.52	-0.7	49.4	2.34
-1.1	-10.28	-4.55	-3.57	-0.7	48.31	3.01
-0.62	-11.33	-4.17	-3.8	-0.7	44.46	2.73
-2.22	-8.91	-1.89	-3.25	-0.7	53.84	1.21
1.18	-8.55	-2.65	-3.16	-0.7	55.33	1.44
1.7	-3.41	-4.82	-1.68	-0.7	81.86	3.55
8.26	-15.63	-7.16	-4.54	-0.7	31.36	7.71
0.21	-2.69	-4.07	-1.44	-0.7	86.39	2.76
-1.8	-10.85	-5.86	-3.67	-0.69	46.18	5.28
-2.09	-18.45	-3.99	-4.94	-0.69	24.74	2.64
-0.01	-2.91	-4.68	-1.5	-0.69	84.93	3.43
5.79	-3.46	-7.53	-1.69	-0.69	81.53	8.55
-0.14	-2.15	-2.75	-1.24	-0.69	89.91	1.61
1.88	-3.38	-4.84	-1.67	-0.69	82.02	3.59
2.11	-3.45	-5.46	-1.69	-0.69	81.61	4.72
-0.17	-7.62	-2.22	-2.91	-0.69	59.43	1.32
-0.25	-2.59	-4.82	-1.39	-0.69	86.95	3.62
5.97	-12.9	-7.33	-4.08	-0.69	39.19	7.37
-1.9	-13.44	-3.19	-4.19	-0.69	37.47	1.81
0.3	-2.96	-5.3	-1.52	-0.68	84.53	4.24
0.39	-3	-5.15	-1.53	-0.68	84.3	4.02
-0.55	-2.67	-4.15	-1.42	-0.68	86.4	2.81
-2.56	-12.5	-1.67	-4.01	-0.68	40.43	1.07
-1.67	-7.07	-5.42	-2.76	-0.68	61.99	4.72
-1.57	-9.75	-3.49	-3.45	-0.68	50.32	2.22
0.7	-4.99	-3.32	-2.18	-0.68	72.64	2.08
-1.76	-8.86	-1.7	-3.24	-0.68	53.98	0.96
-2.06	-18.96	-4.59	-4.99	-0.68	23.68	3.51
-0.35	-7.46	-2.52	-2.87	-0.68	60.15	1.48
-0.97	-6.14	-1.43	-2.51	-0.68	66.55	0.93
7.8	-16.59	-7.21	-4.67	-0.68	28.92	7.38
-0.75	-5.45	-5	-2.32	-0.68	70.12	3.93
-0.6	-4.99	-4.91	-2.18	-0.68	72.61	3.67
0.34	-2.98	-5.5	-1.52	-0.67	84.36	4.56
3.2	-13.64	-0.36	-2.83	-0.67	31.58	1.46
-2.25	-13.98	-2.18	-4.25	-0.67	35.84	1.33
2.51	-4.33	-7.28	-1.96	-0.67	76.25	7.57
0.87	-13.16	-5.4	-4.12	-0.67	38.31	4.61

-1.82	-8.19	-1.86	-3.06	-0.67	56.8	1.13
2.06	-3.47	-5.64	-1.68	-0.67	81.37	4.87
0.74	-0.82	-5.63	-0.73	-0.67	98.95	4.77
0.72	-0.83	-5.77	-0.73	-0.67	98.86	4.89
-1.92	-3.75	-1.77	-1.77	-0.67	79.64	1.07
-1.07	-6.18	-2.6	-2.51	-0.67	66.27	1.6
-0.9	-9.22	-2.96	-3.32	-0.66	52.37	1.87
-0.48	-2.71	-3.31	-1.42	-0.66	86.02	2.13
-1.76	-8.8	-2.78	-3.21	-0.66	54.1	1.82
-1.76	-8.15	-2.45	-3.04	-0.66	56.92	1.45
-1.78	-8.14	-2.09	-3.05	-0.66	57	1.24
5.25	-20.43	-5.06	-5.14	-0.66	20.84	3.94
-0.24	-2.59	-4.49	-1.37	-0.66	86.76	3.3
-0.84	-6.21	-1.13	-2.52	-0.66	66.11	0.81
1.48	-19.12	-3.83	-4.98	-0.66	23.31	2.52
0.25	-6.58	-3.17	-2.62	-0.66	64.29	1.96
-1.12	-9.22	-4.16	-3.28	-0.66	52.37	2.85
6.75	-0.79	-2.74	-0.7	-0.65	98.95	1.51
-0.93	-10.72	-0.75	-3.61	-0.65	46.5	0.71
-1.71	-7.98	-1.88	-3	-0.65	57.65	1.21
16.17	-18.28	-5.33	-4.88	-0.65	25.02	4.1
1.89	-10.8	-5.01	-3.63	-0.65	46.24	3.77
2.32	-5.54	-3.71	-2.31	-0.65	69.52	2.26
-1.73	-8.16	-2.85	-3.04	-0.65	56.86	1.72
0.16	-7.89	-2.78	-2.96	-0.65	58.04	1.7
-0.97	-6.4	-3.2	-2.56	-0.64	65.07	1.99
-0.93	-6.65	-3.37	-2.63	-0.64	63.8	2.17
-0.63	-2.61	-3.67	-1.37	-0.64	86.57	2.4
0.27	-11.23	-2.71	-3.72	-0.64	44.62	1.66
-0.03	-7.65	-2.25	-2.89	-0.64	59.1	1.34
-0.87	-6.27	-1.6	-2.54	-0.64	65.7	0.93
-2.2	-12.16	-3.53	-3.9	-0.64	41.44	2.32
5.64	-18.29	-6.15	-4.87	-0.64	24.99	6.31
-1.72	-8.15	-2.8	-3.03	-0.64	56.86	1.73
-1.76	-10.9	-5.62	-3.65	-0.63	45.75	5.04
-0.77	-8.79	-3.19	-3.19	-0.63	54.08	2.11
-2.9	-15.09	-3.11	-4.44	-0.63	32.61	1.79
-0.68	-11.16	-3.73	-3.72	-0.63	44.85	2.44
-1.83	-6.13	-2.86	-2.46	-0.63	66.37	1.74
-1.77	-6.13	-2.82	-2.46	-0.63	66.37	1.75
-2.24	-12.11	-3.47	-3.89	-0.63	41.58	2.21
-1.64	-13.27	-3.38	-4.12	-0.63	37.87	2.45
0.23	-6.43	-2.97	-2.55	-0.63	64.88	1.82
0.07	-2.91	-4.15	-1.47	-0.62	84.51	2.84

-2.74	-15.07	-3.49	-4.43	-0.62	32.64	2.14
-0.7	-13.04	-1.65	-4.07	-0.62	38.52	1
-1.74	-5.51	-3.65	-2.29	-0.62	69.56	2.51
-1.07	-11.08	-1.28	-3.67	-0.62	45.09	0.91
-0.6	-10.6	-0.88	-3.57	-0.62	46.82	0.77
-0.99	-8.98	-2.26	-3.23	-0.62	53.2	1.37
2.89	-7.29	-1.88	-2.79	-0.62	60.67	0.98
-1.29	-9.25	-5.05	-3.27	-0.62	52.12	4.07
-0.62	-5.6	-1.32	-2.32	-0.62	69.06	0.93
-1.81	-13.13	-3.75	-4.08	-0.62	38.25	2.42
-1	-6.15	-2.69	-2.48	-0.62	66.2	1.66
-2.02	-18.6	-4.18	-4.91	-0.61	24.29	2.92
-0.67	-10.69	-1.62	-3.59	-0.61	46.48	1.08
-0.54	-10.64	-0.62	-3.57	-0.61	46.65	0.71
-0.22	-2.58	-4.57	-1.35	-0.61	86.53	3.45
1.24	-4.74	-4.44	-2.04	-0.61	73.64	3.02
0.25	-2.8	-4.01	-1.42	-0.61	85.12	2.97
0.26	-2.89	-4.86	-1.45	-0.6	84.55	3.66
-0.41	-13.4	-2.09	-4.13	-0.6	37.4	1.23
-2.27	-11.79	-1.78	-3.8	-0.6	42.55	1.16
-1.86	-8.25	-1.63	-3.03	-0.6	56.25	1.03
0.09	-7.77	-2.41	-2.89	-0.6	58.38	1.46
2.3	-3.37	-5.79	-1.6	-0.6	81.55	5.18
2.39	-3.37	-6.08	-1.61	-0.6	81.5	5.57
-0.82	-5.71	-1.78	-2.34	-0.6	68.35	1.15
-0.68	-5.48	-5.49	-2.27	-0.6	69.56	4.58
1.16	-1.2	-5.5	-0.84	-0.6	95.7	4.61
0.9	-4.56	-1.56	-1.99	-0.6	74.6	1.11
0.27	-6.54	-3.45	-2.57	-0.6	64.19	2.19
2.28	-18.94	-4.98	-6.56	-0.59	28.12	1.36
-0.85	-14.33	-6.29	-4.27	-0.59	34.62	6.41
-1.34	-10.81	-1.08	-3.61	-0.59	45.99	0.87
0.22	-4.65	-4.54	-2.02	-0.59	74.03	3.5
-1.9	-6.19	-3.22	-2.45	-0.59	65.88	2.01
0.15	-7.86	-3	-2.91	-0.59	57.94	1.89
1.18	-3.49	-6.25	-1.63	-0.58	80.68	6.38
-1.05	-10.61	-0.8	-3.54	-0.58	46.66	0.73
-1.78	-8.08	-2.53	-2.98	-0.58	56.9	1.63
-1.76	-8.38	-3	-3.05	-0.58	55.58	2.02
1.47	-4.8	-4.24	-2.05	-0.58	73.18	2.98
-0.91	-6.14	-2.77	-2.45	-0.58	66.1	1.77
2.79	-13.37	-6.37	-4.1	-0.57	37.4	6.19
1.07	-5.4	-3.09	-2.24	-0.57	69.84	2.02
-0.87	-9.25	-2.78	-3.27	-0.57	51.93	1.78

-0.88	-6.64	-3.77	-2.58	-0.57	63.56	2.64
1.47	-21.99	-3.33	-5.24	-0.57	18.06	2.32
2.03	-13.37	-1.86	-4.08	-0.57	37.37	1.02
-1.77	-8.24	-3.2	-3.01	-0.57	56.17	2.13
-1.77	-3.79	-2.34	-1.72	-0.57	78.82	1.51
0.63	-5.41	-0.11	-2.23	-0.57	69.79	0.56
0.3	-6.51	-3.5	-2.54	-0.57	64.2	2.28
-0.6	-2.5	-2.4	-1.29	-0.57	86.84	1.42
-1.5	-10.71	0.16	-3.57	-0.56	46.23	0.48
-0.86	-10.02	-0.49	-3.4	-0.56	48.82	0.66
0.4	-13.48	-2.9	-4.1	-0.56	37.04	1.78
-2.19	-12.22	-3.55	-3.86	-0.56	40.98	2.46
-2.52	-12.41	-1.38	-3.9	-0.55	40.33	0.99
0.83	-1.46	-4.58	-0.9	-0.55	93.53	3.54
-0.81	-10.59	-0.71	-3.52	-0.55	46.66	0.69
-1.68	-6.12	-3.07	-2.41	-0.55	66.03	1.96
-1.66	-6.09	-3.12	-2.41	-0.55	66.19	1.97
2.46	-19.9	-2.91	-5	-0.55	21.64	1.96
-0.66	-4.44	-0.73	-1.92	-0.55	74.97	1.09
-1.2	-4.31	-1.14	-1.89	-0.55	75.71	0.92
-0.07	-4.9	-2.9	-2.06	-0.55	72.45	1.86
0.66	-9.37	-3.4	-3.24	-0.55	51.34	2.24
0.35	-9.43	-3.57	-3.26	-0.55	51.11	2.3
-0.42	-0.97	0.38	-0.71	-0.55	96.97	0.49
-0.78	-0.97	-0.27	-0.71	-0.55	97.05	0.64
0.98	-4.96	-4.23	-2.08	-0.54	72.04	2.93
-2.33	-13.85	-5.93	-4.19	-0.54	35.89	5.22
-2.84	-15.45	-5.1	-4.44	-0.54	31.47	3.68
-1.43	-10.73	-0.21	-3.56	-0.54	46.09	0.56
-0.61	-10.46	-1.18	-3.48	-0.54	47.1	0.87
-1.16	-10.22	-4.01	-3.46	-0.54	47.99	2.58
-1	-5.37	-1.38	-2.2	-0.54	69.85	0.93
-0.16	-5.06	-1.83	-2.1	-0.54	71.5	1.18
6.04	-17.31	-6.75	-4.68	-0.54	26.95	6.92
8.16	-14.77	-7.03	-4.3	-0.54	33.25	7.13
-1.25	-4.23	-1.47	-1.85	-0.54	76.15	1.01
-0.63	-6.45	-4.42	-2.5	-0.54	64.37	3.44
-0.04	-4.98	-2.83	-2.08	-0.54	71.93	1.79
-0.37	-5.06	-2.74	-2.1	-0.54	71.49	1.76
1.2	-2.28	-1.67	-1.19	-0.54	88.01	1.04
0.68	-3.01	-4.53	-1.44	-0.53	83.36	3.44
0.69	-3.06	-4.52	-1.46	-0.53	83.05	3.42
0.96	-2.99	-5.01	-1.43	-0.53	83.44	4.13
-2.64	-15	-3.74	-4.36	-0.53	32.62	2.06

-2.48	-12.53	-0.83	-3.92	-0.53	39.89	0.79
-1.21	-11.71	-1.95	-3.74	-0.53	42.62	1.39
-1.46	-10.02	-4.45	-3.4	-0.53	48.74	2.91
0.85	-0.65	-5.27	-0.58	-0.53	99.14	4.39
-0.18	-2.51	-4.58	-1.27	-0.53	86.47	3.51
-0.14	-2.53	-4.59	-1.28	-0.53	86.36	3.51
-0.07	-2.55	-4.61	-1.28	-0.53	86.22	3.54
-0.02	-2.58	-4.57	-1.3	-0.53	86.03	3.51
0.16	-2.34	-4.97	-1.21	-0.53	87.57	4.21
-0.11	-5.16	-2.4	-2.13	-0.53	70.95	1.52
-2.93	-19.39	-4.78	-6.97	-0.52	28.1	0.94
-2.22	-13.88	-1.34	-4.13	-0.52	35.74	0.98
-2.17	-13.86	-1.47	-4.13	-0.52	35.8	1.05
-2.21	-11.92	-1.6	-3.77	-0.52	41.86	1.1
-2.29	-11.9	-1.29	-3.77	-0.52	41.92	0.94
4.29	-2.4	-3.5	-1.22	-0.52	87.13	2.24
0.86	-0.64	-5.31	-0.56	-0.52	99.17	4.54
8.15	-14.95	-6.52	-4.32	-0.52	32.74	6.36
-0.03	-8.14	-2.39	-2.94	-0.52	56.38	1.52
-0.48	-0.93	0.42	-0.68	-0.52	97.06	0.39
-0.36	-2.61	-3.13	-1.29	-0.51	85.7	2.12
-0.46	-4.4	-2.89	-1.89	-0.51	74.98	1.64
-3	-12.67	-5.27	-3.93	-0.51	39.39	4.31
1.63	-12.23	-4.99	-3.83	-0.51	40.81	4.03
-1.85	-8.37	-2.4	-3.01	-0.51	55.37	1.45
-0.11	-8.1	-2.77	-2.92	-0.51	56.55	1.8
1.92	-13.54	-5.33	-4.08	-0.5	36.69	4.46
-0.79	-9.31	-3.05	-3.23	-0.5	51.43	2.07
-0.74	-14.03	-6.29	-4.16	-0.5	35.23	6.55
-1.32	-11.47	-1.85	-3.69	-0.5	43.33	1.31
-2.19	-9.09	-2.04	-3.16	-0.5	52.32	1.34
2.5	-14.99	-5.91	-4.32	-0.5	32.59	5.62
1.54	-10.04	-5.74	-3.37	-0.5	48.54	4.95
-0.81	-6.13	-2.82	-2.39	-0.5	65.73	1.86
5.18	-3.23	-7.11	-1.5	-0.49	81.75	8.56
-2.08	-13.74	-1.89	-4.09	-0.49	36.07	1.28
9.6	-19.76	-6.61	-4.95	-0.49	21.8	7.1
-2.03	-7.58	-2.3	-2.77	-0.49	58.8	1.49
-2.16	-12.33	-3.3	-3.83	-0.49	40.43	2.42
-1.03	-8.99	-2.55	-3.15	-0.48	52.6	1.58
3.4	-2.64	-6.13	-1.28	-0.48	85.33	5.8
15.09	-17.52	-5.08	-4.66	-0.48	26.35	3.18
4.98	-3.28	-5.24	-1.5	-0.48	81.34	4.3
2.78	-12.11	-6	-3.78	-0.48	41.14	5.45

-1.37	-6.01	-2.53	-2.34	-0.48	66.23	1.7
-1.61	-13.25	-3.24	-4.02	-0.48	37.5	2.16
-0.58	-6.24	-3.6	-2.41	-0.48	65.13	2.5
-0.45	-6.22	-3.82	-2.4	-0.48	65.19	2.71
-0.31	-6.19	-3.87	-2.4	-0.48	65.32	2.76
-0.09	-6.29	-3.88	-2.42	-0.48	64.88	2.82
-1.72	-5.44	-3.76	-2.17	-0.47	69.1	2.71
0.87	-1.4	-4.66	-0.82	-0.47	93.44	3.78
-0.04	-10.11	-2.25	-3.37	-0.47	48.18	1.27
-0.48	-5.28	-1.55	-2.13	-0.47	70	1.04
0.21	-2.53	-4.02	-1.23	-0.47	85.96	2.97
1.75	-3.37	-2.34	-1.53	-0.47	80.74	1.38
-0.86	-6.57	-3.97	-2.5	-0.46	63.42	2.96
-0.84	-4.95	-0.82	-2.01	-0.46	71.73	0.81
-1.59	-3.04	-0.89	-1.4	-0.46	82.69	0.81
-0.67	-6.24	-3.33	-2.4	-0.46	65.02	2.21
-1.7	-11.33	-5.14	-3.62	-0.45	43.66	4.81
1.15	-2.92	-4.98	-1.35	-0.45	83.41	4.19
1.37	-2.88	-5.07	-1.34	-0.45	83.66	4.36
1.93	-2.96	-4.96	-1.38	-0.45	83.13	4.19
1.02	-14.26	-5.14	-4.16	-0.45	34.46	4.86
-1.77	-19.71	-4.42	-4.92	-0.45	21.83	3.64
2.76	-19.91	-3.2	-4.93	-0.45	21.46	1.99
-1.56	-2.94	-1.62	-1.36	-0.45	83.27	1.14
0.93	-4.41	-2.48	-1.85	-0.44	74.53	1.73
0.96	-4.68	-3.81	-1.94	-0.44	73.05	2.69
-0.94	-8.46	-2.97	-3	-0.44	54.71	2.03
-1.99	-13.68	-1.45	-4.05	-0.44	36.11	1.12
-2.19	-11.98	-1.32	-3.74	-0.44	41.44	1.03
0.65	-9.86	-0.87	-3.29	-0.44	49.01	0.87
-1.95	-19.86	-4.55	-4.93	-0.44	21.53	3.52
1.16	-1.12	-5.84	-0.7	-0.44	95.19	5.43
-0.83	-13.31	-1.79	-4	-0.44	37.21	1.52
0.11	-5.02	-2.36	-2.02	-0.44	71.16	1.59
2.73	-2.84	-6.29	-1.32	-0.43	83.8	6.56
3.12	-2.92	-6.36	-1.35	-0.43	83.3	6.84
3.47	-2.91	-6.53	-1.35	-0.43	83.36	7.13
5.7	-3.23	-7.09	-1.46	-0.43	81.38	7.63
-0.28	-2.58	-3.18	-1.23	-0.43	85.37	2.25
-0.53	-13.92	-6.26	-4.1	-0.43	35.39	6.81
3.08	-10.9	-5.43	-3.52	-0.43	45.12	4.48
-2.1	-13.77	-1.78	-4.05	-0.43	35.81	1.2
-2.02	-13.66	-1.83	-4.04	-0.43	36.14	1.29
-0.46	-9.66	-1.75	-3.23	-0.43	49.76	1.19

-0.27	-8.74	-1.41	-3.02	-0.43	53.49	1.09
0.18	-2.56	-3.96	-1.22	-0.43	85.56	2.99
1.13	-1.3	-6.11	-0.77	-0.43	93.84	6.1
-0.74	-9.33	-2.69	-3.19	-0.42	51.05	1.86
1.63	-2.94	-5.22	-1.35	-0.42	83.14	4.55
4.42	-4.08	-3.23	-1.73	-0.42	76.32	2.19
-1.82	-13.4	-2.55	-4	-0.42	36.89	1.82
-2.04	-8.89	-1.84	-3.06	-0.42	52.81	1.28
0.07	-4.36	-7.77	-1.81	-0.42	74.7	10.52
0.07	-4.14	-7.7	-1.74	-0.42	75.93	10.05
-1.75	-3.65	-2.58	-1.58	-0.42	78.81	1.77
-1.46	-3.72	-1.74	-1.6	-0.42	78.4	1.19
-1.78	-3.66	-2.2	-1.58	-0.42	78.75	1.48
-2.06	-2.86	-0.46	-1.32	-0.42	83.61	0.63
0.2	-6.36	-3.38	-2.4	-0.42	64.21	2.33
1.12	-5.54	-2.85	-2.18	-0.41	68.34	2.02
-0.77	-8.23	-3.91	-2.92	-0.41	55.59	2.67
6.94	-18.87	-6.02	-4.79	-0.41	23.39	6.35
-1.51	-12.28	-3.21	-3.78	-0.41	40.35	2.34
-1.34	-8.73	-1.4	-3.03	-0.41	53.44	1.19
-1.51	-7.88	-1.01	-2.82	-0.41	57.08	0.77
2.2	-3.4	-5	-1.5	-0.41	80.29	4.39
3.57	-12.58	-5.75	-3.83	-0.41	39.41	5.52
-1.37	-6.03	-2.47	-2.3	-0.41	65.81	1.67
-1.32	-5.98	-2.34	-2.28	-0.41	66.06	1.61
5.32	-20.01	-3	-4.91	-0.41	21.22	1.76
-1.39	-3.8	-1.71	-1.62	-0.41	77.91	1.21
-0.71	-6.19	-3.36	-2.35	-0.41	64.98	2.32
-0.35	-7.61	-2.49	-2.74	-0.41	58.31	1.68
-0.65	-8.69	-3.21	-3.01	-0.4	53.55	2.32
4	-0.73	-5.04	-0.53	-0.4	97.61	3.89
-1.7	-8.03	-3.07	-2.85	-0.4	56.41	2.23
1.55	-8.26	-1.77	-2.9	-0.4	55.39	1.11
-1.31	-3.07	-3.27	-1.37	-0.4	82.13	2.51
5.9	-17.88	-5.71	-4.66	-0.4	25.41	5.3
0.31	-2.64	-3.85	-1.23	-0.4	84.85	2.84
-1.95	-7.47	-2.72	-2.69	-0.4	58.9	1.91
3.28	-6.96	-3.57	-2.55	-0.4	61.27	2.49
-1.88	-18.77	-3.35	-4.78	-0.39	23.55	2.48
-1.85	-18.72	-3.42	-4.78	-0.39	23.64	2.37
2.32	-2.84	-5.82	-1.3	-0.39	83.51	5.66
-1.52	-11.97	0	-3.72	-0.39	41.3	0.54
0.93	-1.32	-4.72	-0.74	-0.39	93.44	4.03
-1.06	-8.85	-4.36	-3.03	-0.39	52.85	3.69

0.31	-2.63	-3.88	-1.22	-0.39	84.86	2.87
3.61	-7.34	-3.8	-2.64	-0.39	59.43	2.76
-0.52	-6.44	-4.08	-2.4	-0.39	63.69	3.15
-2.1	-2.98	-0.96	-1.34	-0.39	82.68	0.84
-0.83	-6.71	-4.13	-2.49	-0.38	62.36	3.23
-2.69	-12.18	-4.65	-3.75	-0.38	40.59	3.91
-1.57	-5.47	-3.69	-2.12	-0.38	68.49	2.87
-1.66	-12.39	-3.92	-3.79	-0.38	39.91	2.94
-1.45	-10.11	-4.17	-3.33	-0.38	47.87	3.21
-0.66	-8.4	-1.5	-2.92	-0.38	54.69	1.14
0.16	-4.93	-1.69	-1.96	-0.38	71.34	1.18
3.79	-4.12	-2.81	-1.71	-0.37	75.81	2.22
-2.86	-12.63	-4.73	-3.83	-0.37	39.09	3.86
-0.08	-13.08	-1.89	-3.91	-0.37	37.69	1.21
-1.62	-6.71	-5.09	-2.47	-0.37	62.33	4.64
0.94	-0.46	-5.78	-0.41	-0.37	99.33	5.54
0.31	-2.57	-3.11	-1.19	-0.37	85.14	2.22
-0.79	-4.75	-0.24	-1.9	-0.37	72.3	0.65
0.94	-3.51	-2.19	-1.5	-0.37	79.31	1.37
4.32	-2.99	-6.92	-1.33	-0.36	82.39	8.61
-2.02	-13.69	-1.56	-3.99	-0.36	35.85	1.15
-1.99	-13.68	-1.61	-3.99	-0.36	35.91	1.24
-2.01	-11.69	-1.06	-3.62	-0.36	42.15	0.97
0.49	-4.49	-2.11	-1.83	-0.36	73.65	1.56
-2.09	-8.93	-1.95	-3.03	-0.36	52.43	1.33
2.71	-15.35	-5.28	-4.29	-0.36	31.31	5.16
2.73	-3.5	-5.42	-1.5	-0.36	79.37	5.19
-1.54	-6.37	-1.52	-2.38	-0.36	63.89	1.1
0.28	-2.19	-5.6	-1.05	-0.36	87.46	5.43
-2.01	-2.91	-0.82	-1.29	-0.36	82.94	0.8
1.53	-3.2	-6.17	-1.4	-0.35	81.07	6.78
3.81	-2.89	-6.95	-1.29	-0.35	83	8.37
-0.18	-2.54	-2.93	-1.17	-0.35	85.17	2.06
-1.74	-8.74	-2.36	-3	-0.35	53.19	1.52
3.52	-7.45	-1.11	-2.66	-0.35	58.8	1.02
-1.71	-3.63	-1.9	-1.53	-0.35	78.5	1.38
-0.48	-2.29	-1.51	-1.08	-0.35	86.74	1.11
-1.19	-11.39	-1.91	-3.57	-0.34	43.09	1.34
-2.06	-8.98	-1.76	-3.03	-0.34	52.12	1.27
-1.96	-8.84	-1.81	-2.99	-0.34	52.74	1.29
0.23	-2.53	-3.72	-1.16	-0.34	85.14	2.9
0.08	-4.66	-2.5	-1.85	-0.34	72.67	1.67
0.92	-4.52	-2.19	-1.82	-0.33	73.35	1.51
-2.33	-15.2	-2.32	-4.26	-0.33	31.65	1.8

0.96	-1.26	-4.63	-0.69	-0.33	93.4	4.01
-1.39	-12.05	-3.6	-3.69	-0.33	40.86	2.57
6.32	-10.22	-4.93	-3.31	-0.33	47.3	3.78
-1.3	-5.89	-2.3	-2.2	-0.33	66.13	1.61
-1.19	-5.88	-2.18	-2.21	-0.33	66.18	1.57
-0.56	-5.73	-2.78	-2.16	-0.33	66.93	1.98
1.43	-6.03	-0.85	-2.25	-0.33	65.41	0.92
-1.31	-3.83	-1.99	-1.58	-0.33	77.24	1.4
-1.7	-8.06	-3.08	-2.81	-0.33	55.94	2.25
-1.54	-2.91	-0.55	-1.28	-0.33	82.7	0.73
2.27	-14.71	-5.03	-4.15	-0.32	32.92	4.42
-0.75	-4.71	0.3	-1.85	-0.32	72.26	0.54
-0.78	-2.52	-1.94	-1.14	-0.32	85.15	1.45
-0.52	-9.36	-4.01	-3.12	-0.31	50.5	3.14
-0.38	-9.57	-3.82	-3.17	-0.31	49.69	2.92
-2.44	-15.15	-3.13	-4.24	-0.31	31.71	2.17
6.92	-13.88	-1.08	-3.99	-0.31	35.2	1.13
3.69	-15.95	-4.84	-4.34	-0.31	29.68	4.34
-0.97	-10.68	-1.1	-3.4	-0.31	45.51	1.1
2.87	-3.3	-4.67	-1.4	-0.31	80.26	3.99
-1.42	-3.04	-3.23	-1.31	-0.31	81.85	2.39
5.27	-4.02	-0.97	-1.64	-0.31	76.07	0.79
-0.38	-6.37	-3.99	-2.33	-0.31	63.65	3.1
-1.21	-3.8	-2	-1.55	-0.31	77.28	1.43
-1.72	-7.96	-3.51	-2.77	-0.31	56.31	2.45
2.02	-3.32	-2.41	-1.41	-0.3	80.1	1.65
-0.47	-9.39	-4.19	-3.13	-0.3	50.35	3.35
-1.88	-18.65	-3.77	-4.71	-0.3	23.65	2.87
1.04	-1.36	-4.86	-0.71	-0.3	92.57	4.33
1.08	-1.41	-4.55	-0.73	-0.3	92.23	3.81
1.13	-1.45	-4.82	-0.74	-0.3	91.95	4.27
1.18	-1.5	-4.89	-0.76	-0.3	91.64	4.44
-1.67	-19.61	-4.28	-4.81	-0.3	21.78	3.64
3.19	-12.06	-5.47	-3.65	-0.3	40.72	4.98
1.32	-5.82	-0.29	-2.18	-0.3	66.33	0.66
0.76	-5.43	0.88	-2.06	-0.3	68.34	0.4
3.97	-12.67	-4.03	-3.77	-0.3	38.77	2.91
-1.09	-6.04	-1.84	-2.25	-0.3	65.25	1.61
-1.6	-2.95	-0.53	-1.27	-0.3	82.29	0.74
16.84	-17.48	-4.09	-4.53	-0.29	26.07	3.12
5.84	-4.14	-5.24	-1.67	-0.29	75.28	4.64
2.51	-3.38	-5.15	-1.42	-0.29	79.66	4.84
2.46	-3.51	-5.41	-1.46	-0.29	78.87	5.53
-1.29	-5.86	-2.24	-2.17	-0.29	66.11	1.58

-1.56	-6.36	-1.54	-2.33	-0.29	63.6	1.22
4.45	-13.28	-3.9	-3.88	-0.29	36.9	3.09
-1.08	-3.87	-1.57	-1.57	-0.29	76.79	1.32
2.28	-3.26	-3.25	-1.37	-0.28	80.33	2.39
-0.62	-9.31	-3.7	-3.09	-0.28	50.59	2.83
9.32	-19.55	-5.25	-4.78	-0.28	21.85	5.53
-2.09	-12.11	-1.02	-3.65	-0.28	40.55	0.93
-2.04	-11.96	-1.11	-3.62	-0.28	40.99	0.96
-2.14	-11.95	-1.13	-3.62	-0.28	41.05	0.99
-0.06	-10.17	-1.26	-3.25	-0.28	47.29	1.12
-0.18	-10.1	-1.18	-3.24	-0.28	47.54	1.04
-1.79	-12.72	-3.16	-3.78	-0.28	38.57	2.24
7.08	-11.17	-4.97	-3.47	-0.28	43.69	4.34
6.58	-5.84	-5.32	-2.18	-0.28	66.14	4.96
-1.62	-6.46	-2.03	-2.35	-0.28	63.07	1.44
-0.82	-2.53	-1.3	-1.11	-0.28	84.75	1.05
-0.57	-0.67	0.07	-0.43	-0.28	97.22	0.53
-0.38	-13.92	-6.04	-3.99	-0.27	34.96	6.62
-1.88	-13.86	-1.74	-3.98	-0.27	35.14	1.32
0.18	-3.08	-6.16	-1.3	-0.27	81.34	6.87
0.97	-0.36	-5.79	-0.31	-0.27	99.39	5.66
0.9	-4.35	-1.44	-1.72	-0.27	73.97	1.2
-1.26	-4	-1.93	-1.61	-0.27	75.94	1.33
-1.51	-6.44	-2.22	-2.34	-0.27	63.16	1.56
-1.72	-3.57	-1.67	-1.46	-0.27	78.44	1.26
7.13	-0.36	-3.62	-0.3	-0.26	99.25	2.52
-1.73	-8.1	-2.81	-2.78	-0.26	55.5	2.15
-1.88	-8.75	-1.42	-2.92	-0.26	52.79	1.17
-1.84	-8.68	-1.39	-2.91	-0.26	53.07	1.13
-1.05	-5.92	-2.21	-2.17	-0.26	65.62	1.65
-1.52	-6.43	-1.18	-2.33	-0.26	63.13	1.15
-1.03	-4.34	0.9	-1.71	-0.26	73.95	0.41
1.17	-5.56	-3.11	-2.09	-0.25	67.42	2.45
-1.76	-8.09	-2.34	-2.77	-0.25	55.53	1.59
-1.78	-8.24	-3.15	-2.81	-0.25	54.88	2.21
-1.1	-5.86	-2.16	-2.15	-0.25	65.93	1.59
-1.12	-4.17	-1.05	-1.65	-0.25	74.85	0.98
5.04	-3.88	-3.5	-1.55	-0.24	76.45	2.52
-0.74	-0.63	1.25	-0.39	-0.24	97.21	0
0.79	-6.26	-3.4	-2.25	-0.24	63.86	2.21
-1.43	-2.95	-3.12	-1.24	-0.24	82.01	2.22
-0.47	-5.77	-2.82	-2.12	-0.24	66.34	2.06
1.17	-5.67	-0.46	-2.09	-0.24	66.84	0.71
-2.14	-12.34	-3.13	-3.67	-0.24	39.67	2.37

0.3	-2.47	-2.99	-1.07	-0.24	84.93	2.13
-1.58	-2.98	-0.68	-1.24	-0.24	81.74	0.81
-0.31	-9.61	-3.65	-3.13	-0.23	49.25	2.83
-11.56	-25.78	-15.3	-14.24	-0.23	31.61	1.27
0.71	-2.45	-5.28	-1.06	-0.23	84.96	5.53
0.3	-2.52	-4.03	-1.09	-0.23	84.54	3.51
3.92	-12.22	-6.22	-3.64	-0.23	40.02	6.46
-1.45	-3.03	-3.21	-1.26	-0.23	81.44	2.37
-0.02	-6.5	-3.9	-2.32	-0.23	62.67	3.37
-1.98	-2.85	-1.75	-1.2	-0.23	82.51	1.24
-0.68	-2.68	-1.09	-1.13	-0.23	83.52	1.38
-0.36	-7.78	-3.37	-2.66	-0.23	56.8	2.58
1.97	-8.13	-3.51	-2.74	-0.23	55.26	2.57
-1.7	-13.86	-6	-3.98	-0.22	35.03	6.03
-1.56	-6.65	-4.55	-2.36	-0.22	61.92	4.35
4.56	-3.56	-4.66	-1.43	-0.22	78.18	4.25
4.77	-8.96	-1.41	-2.94	-0.22	51.77	1.11
-1.77	-8.66	-1.86	-2.88	-0.22	52.99	1.44
-0.53	-8.46	-1.56	-2.84	-0.22	53.84	1.32
4.5	-12.36	-6.19	-3.66	-0.22	39.55	6.59
-0.97	-8.55	-4.32	-2.85	-0.22	53.47	3.69
4.51	-12.71	-3.67	-3.72	-0.22	38.46	2.8
-1.53	-7.63	-1.65	-2.61	-0.21	57.37	1.71
4.9	-2.98	-6.14	-1.23	-0.2	81.5	6.52
-0.09	-2.42	-2.45	-1.03	-0.2	84.94	1.78
5.14	-17.68	-5.57	-4.5	-0.2	25.46	5.98
2.94	-15.86	-4.68	-4.25	-0.2	29.67	4.25
-1.93	-11.62	-0.93	-3.5	-0.2	41.91	0.97
-1.3	-12.19	-2.82	-3.63	-0.2	40.03	2.08
17.02	-18.23	-4.73	-4.57	-0.2	24.33	4.01
-1.2	-2.95	-3.11	-1.21	-0.2	81.66	2.49
-2.13	-2.78	-1.25	-1.15	-0.2	82.71	1
-0.03	-2.47	-2.31	-1.04	-0.19	84.61	1.72
1.02	-1.15	-3.96	-0.56	-0.19	93.19	3.27
-1.26	-3.87	-2.04	-1.52	-0.19	76.29	1.41
1.23	-3.63	-2.05	-1.43	-0.19	77.61	1.41
-0.51	-2.18	-1.77	-0.94	-0.19	86.46	1.33
-0.36	-0.64	0.09	-0.36	-0.19	96.77	0.67
-0.26	-12.05	-2.83	-3.6	-0.18	40.41	2.36
5.38	-20.3	-4.69	-4.8	-0.18	20.35	3.77
0.53	-5.84	-1.64	-2.1	-0.18	65.67	1.34
-1.91	-7.51	-2.84	-2.56	-0.18	57.74	2.12
-1.37	-13.58	-1.72	-3.88	-0.18	35.72	1.52
1.53	-2.06	-1.16	-0.89	-0.18	87.1	0.97

-1.25	-8.69	-2.56	-2.88	-0.17	52.69	1.74
-1.19	-11.99	-2.33	-3.58	-0.17	40.59	1.75
1.61	-6.2	-0.41	-2.2	-0.17	63.82	0.79
-0.96	-3.78	-2.05	-1.46	-0.17	76.68	1.49
0.25	-7.23	-2.7	-2.48	-0.17	59	2.07
-1.65	-14.11	-3.15	-3.95	-0.16	34.17	2.51
1.56	-4.23	-2.28	-1.62	-0.16	74.05	1.51
-0.78	-7.93	-1.44	-2.67	-0.16	55.83	1.23
-0.4	-0.61	-0.39	-0.34	-0.16	96.84	0.74
7.08	-0.27	-5.15	-0.19	-0.15	99.09	4.36
0.06	-2.51	-2.43	-1.03	-0.15	84.13	1.89
-0.24	-13.88	-6.16	-3.91	-0.15	34.8	7.45
1.2	-12.81	-3.56	-3.72	-0.15	37.93	2.35
-1.63	-14.23	-3.23	-3.96	-0.15	33.81	2.51
4.83	-9.71	-1.43	-3.07	-0.15	48.57	1.18
-1.23	-8.51	-0.08	-2.8	-0.15	53.32	0.74
-1.27	-2.89	-3.11	-1.17	-0.15	81.79	2.37
5.85	-0.32	-2.76	-0.21	-0.15	98.75	2.13
-2.1	-12.35	-2.94	-3.62	-0.15	39.38	2.29
-1.39	-13.45	-3.32	-3.83	-0.15	36.03	2.78
2.33	-8.17	-3.47	-2.7	-0.15	54.79	2.69
-1.85	-18.84	-3.49	-4.62	-0.14	22.99	2.77
-2.47	-12.55	-4.64	-3.67	-0.14	38.71	4.07
0.42	-10.55	-1.2	-3.24	-0.14	45.4	1.09
2.1	-3.93	-5.21	-1.5	-0.14	75.68	5.28
0.78	-9.91	-0.07	-3.12	-0.14	47.8	0.72
-1.63	-12.56	-3.67	-3.66	-0.14	38.68	2.79
-1.57	-19.43	-4.01	-4.68	-0.14	21.86	3.61
0.44	-6.92	-1.74	-2.38	-0.14	60.27	1.39
-0.19	-6.68	-1.83	-2.31	-0.14	61.41	1.48
-0.58	-4.69	-0.21	-1.73	-0.14	71.43	0.75
0.56	-4.56	-0.57	-1.71	-0.14	72.11	0.87
-1.59	-3.13	-0.46	-1.24	-0.14	80.28	0.79
-1.64	-3.03	-0.95	-1.2	-0.14	80.86	1
-1.1	-2.97	-0.57	-1.18	-0.14	81.21	0.84
-1.12	-2.75	-0.04	-1.11	-0.14	82.59	0.71
-0.08	-1	-0.49	-0.48	-0.14	93.93	1.07
-0.63	-0.5	1.38	-0.27	-0.13	97.36	0
-1.01	-7.86	-2.5	-2.63	-0.13	56.01	2.05
1.18	-13.85	-4.16	-3.89	-0.13	34.83	3.72
-1.65	-2.89	-0.88	-1.14	-0.13	81.68	0.95
-2.98	-18.85	-4.91	-6.48	-0.12	28.1	1.45
0.63	-5.62	-2.11	-2.02	-0.12	66.48	1.86
7.9	-11.42	-4.48	-3.42	-0.12	42.33	3.91

0.73	-2.39	-5.14	-0.98	-0.12	84.71	4.98
1.09	-0.18	-5.28	-0.14	-0.12	99.56	5.25
1.53	-5.91	-0.09	-2.08	-0.12	65.02	0.66
-1.13	-4.36	-1.05	-1.62	-0.12	73.16	0.99
-1.74	-8.62	-1.91	-2.8	-0.12	52.78	1.51
-0.11	-2.23	-0.98	-0.92	-0.12	85.73	1.11
0.01	-6.18	-1.96	-2.16	-0.12	63.69	1.53
3.81	-13.59	-4.89	-3.83	-0.11	35.49	4.94
-1.22	-8.61	-2.42	-2.82	-0.11	52.78	1.8
-2.93	-19.66	-5.82	-7.21	-0.11	28.07	1.4
-1.89	-13.65	-1.51	-3.83	-0.11	35.34	1.3
-1.95	-13.64	-1.45	-3.82	-0.11	35.37	1.24
10.16	-19.96	-5.75	-4.71	-0.11	20.85	5.74
0.05	-10.81	-1.4	-3.28	-0.11	44.38	1.22
0.16	-10.64	-1.45	-3.24	-0.11	44.99	1.23
-1.55	-14.22	-3.23	-3.93	-0.11	33.72	2.58
0.59	-9.34	-1.7	-2.96	-0.11	49.87	1.39
0.26	-3.07	-6.27	-1.2	-0.11	80.51	7.52
0.71	-10.41	-2.59	-3.19	-0.11	45.81	2
-0.74	-5.7	-2.01	-2.01	-0.11	66.02	1.58
0.88	-4.27	-1.4	-1.6	-0.11	73.57	1.12
-1.23	-13.64	-1.52	-3.84	-0.11	35.37	1.69
-1.64	-8.69	-1.67	-2.81	-0.1	52.42	1.45
-1.71	-8.65	-1.84	-2.8	-0.1	52.56	1.48
0.3	-10.64	-2.19	-3.24	-0.1	44.97	1.65
0.9	-10.51	-2.82	-3.21	-0.1	45.46	2.23
0.9	-5.87	-0.1	-2.06	-0.1	65.13	0.66
-0.87	-5.76	-1.7	-2.02	-0.1	65.69	1.38
-1.49	-6.38	-2.31	-2.22	-0.1	62.67	1.68
0.82	-7.56	-2.51	-2.52	-0.1	57.22	1.93
0.69	-7.23	-2.99	-2.43	-0.1	58.71	2.3
-0.51	-8.17	-3.23	-2.7	-0.09	54.52	2.37
0.77	-4.32	-3.46	-1.6	-0.09	73.17	2.73
-1.13	-11.82	-2.2	-3.49	-0.09	40.93	1.69
-1.77	-12	-1.84	-3.51	-0.09	40.34	1.48
4.59	-0.54	-4.23	-0.27	-0.09	96.77	3.5
-0.8	-5.7	-1.95	-2.01	-0.09	65.94	1.53
-0.61	-5.6	-2.14	-1.98	-0.09	66.43	1.67
-0.62	-5.63	-2.18	-1.98	-0.09	66.3	1.7
-1.82	-12.04	-1.91	-3.52	-0.09	40.19	1.55
-1.37	-13.46	-2.4	-3.8	-0.09	35.82	1.86
-0.05	-7.91	-2.65	-2.61	-0.09	55.65	2.01
0.53	-7.62	-2.92	-2.52	-0.09	56.92	2.43
-2.27	-15.11	-2.58	-4.08	-0.08	31.28	1.99

-1.54	-5.37	-2.9	-1.9	-0.08	67.57	2.3
1.39	-1.76	-3.61	-0.72	-0.08	88.48	3.2
-1.72	-14.15	-2.65	-3.91	-0.08	33.85	2.03
-1.71	-7.97	-3.23	-2.63	-0.08	55.37	2.56
6.14	-12.78	-5.99	-3.64	-0.08	37.82	6.74
5.93	-12.84	-5.82	-3.65	-0.08	37.65	6.62
-1.45	-3.02	-2.8	-1.16	-0.08	80.6	2.2
-1.46	-3.01	-2.82	-1.15	-0.08	80.65	2.17
1.73	-6.2	-0.77	-2.14	-0.08	63.39	0.97
0.77	-4.39	-1.05	-1.61	-0.08	72.79	1.06
-0.18	-9.75	-3.58	-3.06	-0.07	48.16	2.99
3.86	-15.54	-5.53	-4.12	-0.07	30.17	5.63
-2.24	-12.51	-4.75	-3.62	-0.07	38.66	4.59
1.28	-1.3	-4.2	-0.54	-0.07	91.44	3.87
-1.7	-12.58	-3.48	-3.61	-0.07	38.42	2.95
-1.47	-14.31	-3.15	-3.93	-0.07	33.4	2.67
-1.17	-3	-2.35	-1.15	-0.07	80.63	2.16
-1.99	-12.34	-2.78	-3.56	-0.07	39.19	2.24
0.66	-4.43	-1.2	-1.61	-0.07	72.46	1.15
0.51	-4.29	-1.28	-1.57	-0.07	73.23	1.22
2.47	-7.65	-3.27	-2.52	-0.07	56.72	2.58
-1.21	-18.77	-3.27	-4.56	-0.06	23	2.73
6.64	-3.47	-6.18	-1.31	-0.06	77.83	8.21
-1.75	-12.61	-3.56	-3.61	-0.06	38.3	2.86
-0.57	-8.05	-4.43	-2.62	-0.06	54.95	4.25
-1.12	-3.8	-0.72	-1.42	-0.06	75.97	0.72
-1.07	-2.65	-0.44	-1.02	-0.06	82.75	0.79
-0.23	-2.17	-0.63	-0.86	-0.06	85.69	0.92
2.58	-7.83	-3.57	-2.56	-0.06	55.88	2.97
6.34	-18.27	-4.87	-4.48	-0.05	23.98	4.69
2.13	-3.95	-4.75	-1.45	-0.05	74.99	4.71
-1.27	-7.86	-2.02	-2.58	-0.05	55.74	1.58
3.44	-17.23	-4.65	-4.35	-0.05	26.18	4.72
1.9	-6.34	-1.42	-2.16	-0.05	62.62	1.31
-1.05	-4	-1.27	-1.46	-0.05	74.74	1.23
-1.12	-2.46	-0.3	-0.95	-0.05	83.85	0.73
-0.91	-11.63	-0.45	-3.4	-0.04	41.41	0.85
2.83	-3.99	-5.55	-1.46	-0.04	74.72	6.12
3.13	-3.45	-5.82	-1.29	-0.04	77.84	6.71
0.3	-2.94	-5.67	-1.11	-0.04	80.83	6.31
-1.88	-13.65	-1.53	-3.77	-0.03	35.14	1.3
2.36	-3.52	-5.28	-1.3	-0.03	77.38	5.27
-0.43	-8.77	-0.87	-2.8	-0.03	51.86	1.17
-1.2	-8.62	-1.56	-2.78	-0.03	52.46	1.34

2.9	-17.45	-5.69	-4.37	-0.03	25.66	6.38
2.73	-3.35	-6.04	-1.25	-0.03	78.36	7.13
1.47	-10.6	-2.43	-3.19	-0.03	44.89	2.02
0.57	-4.25	-1.27	-1.53	-0.03	73.29	1.19
0.56	-2.82	-1.61	-1.06	-0.03	81.54	1.45
-0.3	-2.12	-1.75	-0.82	-0.03	85.84	1.44
3.12	-0.55	-1.44	-0.23	-0.03	96.29	1.38
-0.2	-8.09	-3.18	-2.64	-0.02	54.59	2.55
-1.63	-3.4	-1.81	-1.25	-0.02	77.98	1.55
-1.77	-12.02	-1.74	-3.46	-0.02	40.03	1.54
2.17	-10	-5.17	-3.04	-0.02	47.04	4.9
0.85	-6.96	-2.41	-2.31	-0.02	59.56	1.9
-1.09	-4.13	-2.22	-1.49	-0.02	73.84	1.71
1.22	-5.69	-2.74	-1.98	-0.01	65.6	2.45
0.16	-2.47	-3.29	-0.93	-0.01	83.56	2.85
7.61	-19.24	-5.59	-4.56	-0.01	22.02	5.66
2.9	-4.2	-5.7	-1.51	-0.01	73.43	6.4
2.54	-3.83	-5.48	-1.39	-0.01	75.49	5.78
-0.18	-7.96	0.22	-2.56	-0.01	55.1	0.64
-0.19	-7.68	0.18	-2.49	-0.01	56.34	0.62
3.12	-18.48	-5.68	-4.49	-0.01	23.49	6.38
5.04	-20.91	-5.2	-4.75	-0.01	19.05	5.4
2.36	-4.27	-3.62	-1.52	-0.01	73.05	2.91
1.32	-4.57	-1.5	-1.62	-0.01	71.41	1.33
-0.09	-9.88	-3.46	-3.04	0	47.39	3.03
-2.16	-15.07	-2.93	-4.02	0	31.2	2.45
-1.56	-5.4	-2.75	-1.86	0	67.03	2.29
-0.76	-10.39	0.02	-3.13	0	45.54	0.72
17.36	-17.95	-2.73	-4.4	0	24.53	2.4
1.28	-4.56	-1.29	-1.61	0	71.41	1.23
-0.08	-3.95	-0.04	-1.42	0	74.75	0.72
3.83	-2.42	-5.55	-0.91	0.01	83.7	5.71
6.54	-10.8	-0.62	-3.2	0.01	44.05	0.86
5.3	-10.13	-1.22	-3.06	0.01	46.44	1.12
-0.21	-7.07	-0.89	-2.33	0.01	58.95	1
-1.47	-3.02	-2.8	-1.1	0.01	80.02	2.22
0.44	-4.41	-1.24	-1.56	0.01	72.12	1.19
-1.62	-11.8	-4.41	-3.4	0.02	40.65	4.63
-0.61	-6.82	-3.64	-2.27	0.02	60.08	3.29
1.28	-1.36	-4.31	-0.51	0.02	90.46	4.17
1.22	-0.03	-5.62	0	0.02	99.66	6.22
-0.37	-8.02	-4.38	-2.56	0.02	54.74	4.38
0.77	-4.59	-1.47	-1.62	0.02	71.16	1.37
0.35	-4.51	-1.44	-1.58	0.02	71.55	1.26

5.44	-0.62	-4.74	-0.23	0.03	95.4	4.54
-1.13	-11.91	-2.62	-3.42	0.03	40.27	2.04
-1.17	-8.39	-0.19	-2.66	0.03	53.15	0.8
3.15	-3.14	-5.28	-1.13	0.03	79.23	5.64
0.35	-2.88	-5.43	-1.04	0.03	80.74	6.09
1.37	0.01	-5.06	0.02	0.03	99.84	5.28
0.12	-7.98	-2.65	-2.57	0.04	54.83	2.17
5.96	-16.44	-5.21	-4.17	0.04	27.78	5.07
1.17	-9.21	-1.87	-2.84	0.04	49.84	1.4
0.77	-1.67	-3.2	-0.62	0.04	88.22	3.11
0.34	-2.9	-5.77	-1.05	0.04	80.66	6.72
1.32	0	-5.26	0.03	0.04	99.75	5.67
1.71	-3.26	-2.02	-1.17	0.04	78.49	1.59
-1.35	-6.3	-1.51	-2.11	0.04	62.39	1.5
1.96	-6.2	-1.12	-2.07	0.04	62.89	1.18
4.93	-11.5	5.03	0.14	0.05	28.04	1.16
0.45	-2.59	-2.37	-0.93	0.05	82.42	2.11
-0.09	-13.7	-5.95	-3.74	0.05	34.79	7.34
2.72	-3.41	-5.51	-1.22	0.05	77.56	5.95
1.3	-10.46	-1.93	-3.11	0.05	45.11	1.62
1.95	-2.03	-0.33	-0.74	0.05	85.89	0.81
2.26	-2.03	-2.13	-0.73	0.06	85.84	1.78
0.26	-2.39	-3.13	-0.87	0.06	83.55	2.67
5	-13.2	-2.27	-3.63	0.06	36.2	1.85
-1.9	-12.28	-2.27	-3.46	0.06	39	1.91
-1.81	-12.27	-1.99	-3.46	0.06	39.03	1.74
1.16	-4.59	-0.79	-1.58	0.06	70.93	1.01
1.01	-4.58	-0.96	-1.58	0.06	70.98	1.06
-1.6	-8.69	-2.04	-2.7	0.06	51.79	1.77
-1.63	-3.35	-1.69	-1.19	0.06	77.84	1.5
-1.57	-3.25	-1.56	-1.16	0.06	78.41	1.45
0.84	-6.81	-2.52	-2.22	0.06	59.92	2.1
0.86	-6.68	-2.64	-2.18	0.06	60.5	2.16
0.86	-7.25	-2.58	-2.34	0.06	57.9	2.07
0.53	-2.87	-1.87	-1.02	0.06	80.69	1.53
-0.53	-7.88	-1.51	-2.51	0.07	55.14	1.35
-0.24	-7.3	1.05	-2.35	0.07	57.68	0.42
5.07	-20.87	-5.04	-4.69	0.07	19.03	5.02
1.37	-4.4	-0.99	-1.51	0.07	71.89	1.09
1.92	-8.63	-3.4	-2.69	0.07	51.99	2.52
0.59	-3.46	-2.2	-1.21	0.07	77.13	1.72
0.77	-7.77	-2.9	-2.46	0.07	55.62	2.44
-0.75	-11.58	0.4	-3.32	0.08	41.19	0.61
-0.87	-10.39	0.81	-3.09	0.08	45.29	0.46

-0.21	-7.52	0.35	-2.39	0.08	56.67	0.59
0.49	-2.23	-3.38	-0.79	0.08	84.46	3.18
-1.02	-3.5	-0.22	-1.22	0.08	76.88	0.82
-1.51	-13.63	-2.64	-3.71	0.08	34.92	2.48
-1.28	-13.48	-2.43	-3.69	0.08	35.34	1.9
-1.22	-2.88	0.24	-1.01	0.08	80.51	0.63
-1.15	-2.84	0.55	-1	0.08	80.72	0.65
-1.19	-2.78	0.02	-0.98	0.08	81.11	0.72
1.13	-3.27	-1.95	-1.15	0.09	78.12	1.75
1.32	-1.38	-4.53	-0.48	0.09	89.79	4.4
0.95	-4.83	-2.15	-1.65	0.09	69.51	1.99
-0.11	-7.46	0.05	-2.38	0.09	56.87	0.7
-1.75	-12.28	-1.94	-3.44	0.09	38.92	1.74
-1.06	-13.8	-5.86	-3.76	0.1	34.41	6.53
1.3	-1.33	-4.46	-0.45	0.1	90.1	4.33
-1.72	-12.67	-3.42	-3.52	0.1	37.67	2.89
-1.39	-14.38	-3.27	-3.82	0.1	32.79	2.86
1.31	-14.38	-3.71	-3.82	0.1	32.79	3.51
-1.16	-8.59	-1.24	-2.68	0.1	52.08	1.2
-1.37	-13.36	-3.3	-3.66	0.1	35.64	2.77
-0.2	-0.35	-0.08	-0.07	0.1	96.75	0.75
11.44	-20.64	-4.14	-4.64	0.11	19.34	4.42
8.71	-19.43	-5.92	-4.51	0.11	21.48	5.98
-2.13	-12.88	-0.44	-3.56	0.11	37.02	0.9
3.54	-3.92	-5.61	-1.34	0.11	74.34	6.03
1.31	-1.14	-3.59	-0.38	0.11	91.3	3.21
2.33	-3.88	-4.68	-1.33	0.11	74.54	4.93
2.95	-3.1	-5.05	-1.07	0.11	78.98	5.63
0.38	-2.76	-5.41	-0.96	0.11	81.01	6.19
0.39	-2.64	-5.36	-0.92	0.11	81.72	6.22
0.43	-2.51	-5.51	-0.87	0.11	82.55	6.42
0.46	-2.47	-5.37	-0.86	0.11	82.77	6.11
0.55	-2.38	-5.51	-0.83	0.11	83.32	6.43
5.17	-13.14	-2.83	-3.58	0.11	36.24	2.34
-1.04	-4.01	-0.95	-1.37	0.11	73.85	1.09
0.74	-2.87	-1.24	-0.98	0.11	80.35	1.3
-2.14	-2.85	-1.57	-0.98	0.11	80.51	1.32
2.78	-7.13	-3.47	-2.27	0.11	58.28	2.94
2.29	-3.97	-5.03	-1.36	0.12	74.01	5.44
2.37	-3.99	-5.03	-1.36	0.12	73.88	5.52
-0.18	-7.23	0.71	-2.3	0.12	57.77	0.51
0.49	-2.46	-5.71	-0.85	0.12	82.82	6.85
1.62	-10.48	-1.59	-3.06	0.12	44.8	1.51
-2.15	-3.16	-1.5	-1.08	0.12	78.62	1.39

0.64	-2.67	-1.22	-0.92	0.12	81.53	1.28
-0.16	-0.61	-1.48	-0.17	0.12	94.86	1.34
9.52	-3.01	-1.97	-1.04	0.13	79.42	1.51
3	-4.38	-5.64	-1.48	0.13	71.71	6.46
2.59	-4.26	-5.72	-1.43	0.13	72.37	6.9
2.67	-4.43	-5.58	-1.5	0.13	71.44	6.57
0.39	-2.69	-5.17	-0.92	0.13	81.29	5.85
0.39	-2.62	-5.74	-0.9	0.13	81.75	7.01
0.42	-2.53	-5.78	-0.87	0.13	82.3	7.03
5.43	-13.7	-2.85	-3.67	0.13	34.59	2.59
0.05	-4.21	-0.25	-1.42	0.13	72.62	0.88
-0.95	-3.9	0.21	-1.33	0.13	74.35	0.7
-1.08	-3.58	-1.21	-1.21	0.13	76.1	1.2
-0.01	-9.92	-3.18	-2.96	0.14	46.78	2.86
3.35	-15.24	-4.19	-3.94	0.14	30.46	3.48
-0.33	-8.5	-2.23	-2.61	0.14	52.29	2.01
2.78	-4.54	-5.25	-1.52	0.14	70.81	6.13
2.18	-6.29	-1.84	-2.03	0.14	62.02	1.65
0.42	-2.55	-5.64	-0.87	0.14	82.12	6.72
3.05	-0.41	-1.14	-0.08	0.14	96.11	1.22
5.71	-0.06	-1.58	0.06	0.14	98.56	1.42
4.26	-15.31	-5.09	-3.94	0.15	30.25	5.46
-2.74	-18.77	-4.53	-6.35	0.15	27.94	1.47
1.85	-13.64	-2.84	-3.68	0.15	34.72	2.23
2.16	-3.74	-4.35	-1.26	0.15	75.12	4.42
2.43	-3.88	-5.11	-1.3	0.15	74.3	5.72
-0.16	-7.28	0.32	-2.29	0.15	57.41	0.62
0.57	-2.27	-5.19	-0.76	0.15	83.8	5.85
6.89	-13.16	-5.28	-3.56	0.15	36.1	5.9
-1.72	-12.05	-1.97	-3.36	0.15	39.49	1.8
-1.67	-12.14	-2.09	-3.38	0.15	39.19	1.95
-1.6	-8.71	-1.98	-2.65	0.15	51.41	1.79
0.86	-3.34	-1.72	-1.12	0.15	77.4	1.55
-2	-12.3	-4.79	-3.42	0.16	38.63	4.78
-0.25	-7.69	-1.98	-2.4	0.16	55.58	1.73
8.28	-8.5	-3.92	-2.6	0.16	52.18	3.98
-1.43	-3.11	-2.56	-1.04	0.16	78.69	2.24
-1.41	-3.06	-2.74	-1.02	0.16	78.94	2.38
-1.29	-13.65	-2.51	-3.66	0.16	34.66	2.13
-0.39	-4.64	0.06	-1.53	0.17	70.1	0.74
-0.2	-4.52	0.03	-1.49	0.17	70.73	0.71
-0.65	-3.36	0.16	-1.12	0.17	77.18	0.73
-0.9	-18.78	-3.07	-4.4	0.18	22.59	2.81
3.89	-13.48	2.47	-1.57	0.18	27.91	1.26

-1.41	-13.4	-0.73	-3.59	0.18	35.32	1.22
4.13	-7.03	-1.93	-2.21	0.18	58.4	1.45
-0.16	-7.9	-4.41	-2.43	0.18	54.6	4.6
0.55	-2.43	-3.63	-0.81	0.18	82.62	3.37
0.85	-4.59	-1	-1.52	0.18	70.32	1.16
1.12	-3.1	-1.25	-1.03	0.18	78.61	1.34
0.05	-1.92	-0.06	-0.62	0.18	85.79	0.8
-0.05	-0.28	-1.27	0	0.18	96.72	1.36
-1.45	-17.47	-1.49	-4.21	0.19	25.23	1.54
6.53	-17.62	-4.08	-4.23	0.19	24.89	4.23
4.5	-15.71	-4.16	-3.96	0.19	29.19	3.68
2.22	-3.87	-4.33	-1.28	0.19	74.2	4.38
2.27	-3.81	-4.27	-1.26	0.19	74.53	4.24
7.47	-7.06	-4.68	-2.21	0.19	58.24	4.69
3.2	-3.17	-4.95	-1.05	0.19	78.17	5.56
0.4	-2.61	-5.41	-0.86	0.19	81.42	6.36
0.58	-2.14	-5.05	-0.69	0.19	84.3	5.65
0.58	-2.09	-5.2	-0.67	0.19	84.66	5.95
1.64	0.19	-4.62	0.19	0.19	100	4.88
-2.12	-3.25	-0.8	-1.07	0.19	77.68	1.05
-1.01	-2.83	-0.01	-0.93	0.19	80.15	0.69
0.8	-2.73	-1.08	-0.89	0.19	80.73	1.18
-0.11	-2.6	-0.29	-0.86	0.19	81.55	0.85
0.89	-2.59	-0.71	-0.85	0.19	81.61	1.14
-0.06	-2.36	-0.63	-0.77	0.19	82.96	0.97
2.12	-2.96	-5.51	-0.97	0.2	79.33	7.25
2.56	-4.12	-5.38	-1.35	0.2	72.75	6.39
-1.11	-8.5	-0.93	-2.58	0.2	52.07	1.24
0.58	-2.04	-5.26	-0.66	0.2	84.91	6.09
0.57	-1.98	-5.6	-0.63	0.2	85.3	6.82
0.57	-1.9	-5.46	-0.6	0.2	85.8	6.52
0.58	-1.83	-5.41	-0.57	0.2	86.21	6.48
1.55	0.2	-5.6	0.2	0.2	99.95	6.74
0.06	-3.93	-0.41	-1.28	0.2	73.79	0.9
-1.14	-3.67	-0.77	-1.21	0.2	75.24	0.83
-0.05	-2.44	-0.85	-0.79	0.2	82.43	1.06
2.94	-0.39	-1.06	-0.04	0.2	95.77	1.16
0.32	-2.33	-2.61	-0.75	0.21	83.04	2.24
0.09	-13.61	-5.88	-3.63	0.21	34.63	7.78
-2.02	-13.05	-0.99	-3.52	0.21	36.25	1.28
2.51	-3.88	-5.53	-1.27	0.21	74.01	6.68
17.38	-17.65	-4.31	-4.22	0.21	24.8	4.01
-1.24	-19.49	-3.88	-4.45	0.21	21.21	3.81
4.44	-20.39	-4.77	-4.55	0.21	19.62	5.32

1.8	-10.74	-1.68	-3.05	0.21	43.62	1.65
0.57	-3.05	-3.12	-0.99	0.21	78.7	2.86
4.32	-14.25	-5	-3.73	0.22	32.86	5.1
1.44	-2.87	-2.6	-0.93	0.22	79.77	2.25
-1.56	-3.1	-1.76	-1	0.22	78.35	1.65
-1.55	-3.12	-1.7	-1.01	0.22	78.27	1.62
1.59	-3.97	-0.62	-1.29	0.22	73.46	0.95
0.51	-2.67	-1.64	-0.86	0.22	80.87	1.45
-0.21	-0.38	-1.09	-0.01	0.22	95.69	1.19
-2.08	-15.12	-2.98	-3.87	0.23	30.56	2.81
6.14	-0.73	-4.58	-0.15	0.23	93.29	4.62
2.33	-6.41	-2.51	-2.01	0.23	61.04	2.28
0.45	-3.35	-3.26	-1.08	0.23	76.87	3.01
0.62	-2.96	-3.17	-0.95	0.23	79.15	2.93
0.45	-2.04	-3.31	-0.63	0.23	84.73	3.22
-1.55	-3.11	-1.88	-1	0.23	78.32	1.75
-1.52	-3.13	-1.84	-1.01	0.23	78.16	1.69
5.33	-0.34	-1.58	0	0.23	95.97	1.63
-1.56	-8.67	-2.14	-2.59	0.23	51.25	1.97
5.3	-0.25	-1.44	0.04	0.23	96.59	1.45
0.06	-10	-3.13	-2.92	0.24	46.15	2.97
-1.29	-17.55	-1.81	-4.19	0.24	24.95	1.66
-0.55	-6.86	-3.66	-2.14	0.24	58.95	3.58
-0.36	-11.62	-0.26	-3.22	0.24	40.56	0.91
-0.62	-10.14	0.12	-2.93	0.24	45.63	0.75
0.29	-8.06	-0.8	-2.43	0.24	53.67	1.13
0.42	-2.28	-3.45	-0.71	0.24	83.13	3.36
0.38	-3.78	-3.47	-1.21	0.24	74.4	3.27
0.6	-2.81	-3.46	-0.89	0.24	80	3.2
-0.31	-4.38	0.34	-1.4	0.24	71.16	0.66
-1.03	-3.66	0.35	-1.17	0.24	75.09	0.68
-0.66	-3.24	0.47	-1.04	0.24	77.42	0.62
0.94	-10.9	0.41	-3.07	0.25	42.92	0.73
1.47	-17.02	-3.34	-4.12	0.25	26.06	3.21
0.56	-7.11	-0.72	-2.18	0.25	57.74	0.99
2.07	-10.79	-2.61	-3.05	0.25	43.33	2.43
5.28	-13.58	-2.77	-3.57	0.25	34.64	2.33
-1.64	-12.06	-2.24	-3.3	0.25	39.15	2.14
-0.3	-4.46	0.15	-1.42	0.25	70.7	0.68
0.08	-4.35	-0.15	-1.38	0.25	71.25	0.87
-0.6	-3.17	-0.3	-1.01	0.25	77.86	0.86
-0.16	-2.94	0.39	-0.93	0.25	79.12	0.62
-2.99	-19.76	-5.5	-7.23	0.26	27.86	1.19
10.12	-3.18	-1.99	-1.01	0.26	77.7	2

1.51	-1.58	-3.47	-0.45	0.26	87.5	3.59
0	-7.93	-4.52	-2.38	0.26	54.19	5.06
-1.16	-6.15	-1.24	-1.93	0.26	62.11	1.35
4.94	-13.1	-3.35	-3.48	0.26	35.96	2.9
-1.03	-2.9	0.05	-0.9	0.26	79.35	0.73
4.7	-3.24	-4.49	-1.02	0.27	77.26	4.44
2.24	-4.04	-1.84	-1.29	0.27	72.85	1.61
0.98	-1.48	-5.39	-0.4	0.27	88.01	6.63
1.68	0.27	-4.4	0.27	0.27	100	4.54
-1.24	-6.13	-0.93	-1.92	0.27	62.16	1.13
2.87	-4.53	-5	-1.43	0.28	70.16	5.93
8.57	-11.35	-4.11	-3.15	0.28	41.34	3.77
1.85	-6.23	-2.29	-1.92	0.28	61.66	1.86
3.74	-17.2	-4.75	-4.13	0.28	25.63	5.49
2.97	-3.12	-5.35	-0.98	0.28	77.92	6.33
3.14	-3.72	-0.15	-1.18	0.28	74.5	0.63
0.68	-8.03	-3.14	-2.39	0.28	53.68	3.05
0.54	-2.36	-2.26	-0.72	0.29	82.4	2.06
0.68	-2.46	-2.49	-0.74	0.29	81.77	2.44
3.77	-11.78	-4.84	-3.23	0.29	39.92	4.67
1.12	-5.22	-2.73	-1.64	0.29	66.55	2.6
1.95	-8.28	-0.58	-2.46	0.29	52.6	0.8
-1.02	-2.86	-1.51	-0.88	0.29	79.38	1.67
-1.3	-6.66	-2.11	-2.05	0.29	59.6	2.07
0.2	-3.04	-1.22	-0.94	0.29	78.34	1.45
-1.21	-2.87	0.29	-0.88	0.29	79.33	0.65
0.9	-3.61	-1.97	-1.13	0.3	75.02	1.91
0.9	-1.5	-5.31	-0.4	0.3	87.71	6.4
1.92	-10.63	-2.17	-2.98	0.3	43.72	2.01
0.56	-7.85	-1.54	-2.34	0.3	54.37	1.57
0.62	-7.49	-2.58	-2.25	0.3	55.89	2.4
1.28	-5.67	-2.71	-1.77	0.31	64.2	2.76
0.93	-7.61	-0.2	-2.27	0.31	55.33	0.86
-0.83	-18.05	-3.78	-4.21	0.31	23.8	4.13
0.45	-2.28	-2.85	-0.68	0.31	82.77	2.76
0.63	-0.77	-3.46	-0.11	0.31	92.42	3.86
-1.65	-12.12	-1.85	-3.27	0.31	38.8	1.74
1.17	-3.24	-0.23	-1	0.31	77.08	0.92
2.94	-17.49	2.13	-2.98	0.32	21.99	0.73
0.12	-10.05	-2.98	-2.88	0.32	45.7	2.86
4.62	-15.67	-5.35	-3.88	0.32	29.02	6.39
1.17	-3.17	-1.86	-0.97	0.32	77.39	1.99
4.75	-3.37	-4.37	-1.03	0.32	76.26	4.54
0.31	-3.78	1.06	-1.16	0.32	73.93	0.52

-0.86	-2.8	-1.07	-0.84	0.32	79.58	1.21
0.77	-7.63	-3.08	-2.27	0.32	55.22	3.03
0.1	-0.14	-0.04	0.14	0.32	96.71	0.78
2.15	-23.14	-0.79	-4.73	0.33	15.29	1.15
6.59	-3.18	-5.52	-0.97	0.33	77.28	7.61
0.4	-4.76	-3.94	-1.46	0.33	68.71	3.9
0.7	-3.75	-0.61	-1.15	0.33	74.07	1.12
-0.97	-3.62	-0.23	-1.1	0.33	74.78	0.93
-0.82	-3.5	0.12	-1.07	0.33	75.49	0.78
-0.77	-3.46	0.53	-1.05	0.33	75.69	0.63
-0.84	-3.46	-0.34	-1.06	0.33	75.71	0.95
-0.24	-3.07	0.15	-0.92	0.33	77.94	0.71
1.12	-3.07	0.09	-0.93	0.33	77.96	0.73
-1.07	-2.92	0.51	-0.87	0.33	78.79	0.62
0.47	-2.4	-0.89	-0.7	0.33	81.95	1.16
2.84	-2.77	-2.13	-0.82	0.34	79.64	1.92
-0.44	-9.88	-1.08	-2.81	0.34	46.26	1.33
0.43	-8.17	-0.65	-2.39	0.34	52.84	1.08
2.44	-6.52	-2.09	-1.96	0.34	60.03	2.07
1.24	-1.39	-5.15	-0.33	0.34	88.19	6.35
5.53	-13.14	-3.55	-3.43	0.34	35.64	3.6
5.44	-13.14	-3.49	-3.43	0.34	35.64	3.52
0.84	-3.65	-1.34	-1.11	0.34	74.59	1.47
0.7	-6.43	-3.98	-1.94	0.34	60.45	4.21
-0.6	-3.23	0.15	-0.97	0.34	76.95	0.72
-0.7	-3.2	0.13	-0.96	0.34	77.12	0.77
-0.19	-0.2	-0.73	0.12	0.34	96.12	1.11
10.35	-14.36	5.18	-0.26	0.35	22.02	1.02
7.65	-12.16	-1.54	-3.25	0.35	38.54	1.59
0.25	-7.37	-3.05	-2.2	0.35	56.24	2.85
-1.07	-8.61	-0.41	-2.51	0.35	51.04	1.14
0.48	-8.27	-0.43	-2.42	0.35	52.41	1
0.5	-2.27	-2.78	-0.65	0.35	82.59	2.75
5.16	-13.16	-3.11	-3.43	0.35	35.58	2.91
-0.13	-11.6	-0.15	-3.14	0.36	40.31	0.87
0.07	-11.55	-0.4	-3.13	0.36	40.46	0.98
0.89	-3.56	-1.92	-1.08	0.36	74.98	1.87
0.51	-8.13	-0.59	-2.37	0.36	52.98	1.05
9.74	-10.84	-4.86	-3	0.36	42.82	5.77
2.09	-3.98	-0.52	-1.2	0.36	72.65	0.98
10.74	-20.06	-4.5	-4.4	0.37	19.97	4.8
0.37	-11.58	-0.43	-3.13	0.37	40.35	1.03
2.95	-4.72	-4.83	-1.44	0.37	68.69	5.65
2.13	-10.59	-1.77	-2.93	0.37	43.64	1.65

3.66	-20.24	-4.16	-4.43	0.37	19.66	4.72
1.41	-1.36	-4.97	-0.3	0.37	88.2	6.06
1.92	-2.81	-1.84	-0.82	0.37	79.26	1.63
6.45	0.31	-1.55	0.35	0.37	99.55	1.73
-1.57	-8.69	-1.58	-2.51	0.37	50.67	1.67
4.38	-2.59	-4.83	-0.73	0.38	80.45	5.24
-1.96	-15.24	-1.7	-3.8	0.38	29.94	1.81
1.06	-4.84	-2.53	-1.47	0.38	68	2.3
6.13	-14.17	-2.21	-3.58	0.38	32.7	2.29
-1.52	-11.93	-1.52	-3.19	0.38	39.19	1.7
1.16	-3.23	-0.09	-0.95	0.38	76.72	0.92
3.62	-10.67	-5.63	-2.93	0.38	43.32	7.6
1.02	-2.98	0.54	-0.87	0.38	78.2	0.64
-1.4	-6.32	-2.15	-1.9	0.38	60.79	1.74
-0.81	-2.66	-0.61	-0.76	0.38	80.04	1.09
0.5	-5.99	-3.84	-1.78	0.38	62.33	4.05
0.24	-0.09	-0.56	0.19	0.38	96.67	1.11
6.24	-16.86	-3.69	-4	0.39	26.16	3.51
-0.29	-10.09	-2.21	-2.83	0.39	45.34	2.18
4.28	-20.15	-5.1	-4.4	0.39	19.79	5.8
0.12	-4.19	0.77	-1.24	0.39	71.37	0.6
0.92	-6.41	-4.12	-1.9	0.39	60.32	4.49
-1.48	-8.59	-2.13	-2.47	0.39	51.02	2.13
1.15	-2.93	-0.2	-0.85	0.39	78.45	0.91
2.45	-22.95	0.32	-4.67	0.4	15.48	0.72
3.09	-2.85	-1.63	-0.81	0.4	78.83	1.72
-0.41	-13.9	-5.24	-3.58	0.4	33.36	6.15
4.64	-3.43	-4.6	-1	0.4	75.49	5.3
1.6	-16.04	-2.81	-3.88	0.4	27.97	2.77
1.04	-4.81	0.34	-1.45	0.4	68.06	0.71
0.24	-4.45	0.01	-1.32	0.4	69.94	0.79
-0.79	-3.49	0.93	-1.02	0.4	75.16	0.52
-0.37	-3.33	0.19	-0.96	0.4	76.04	0.76
4.43	-12.38	-6.03	-3.26	0.4	37.74	8.4
-0.69	-3.08	0.04	-0.89	0.4	77.49	0.77
-1.2	-2.78	-0.01	-0.78	0.4	79.19	0.81
0.84	-7.51	-3.08	-2.19	0.4	55.4	3.08
0.44	-0.09	-1.04	0.21	0.4	96.54	1.39
-1.62	-12.17	-3.93	-3.22	0.41	38.34	4.34
4.05	-4.04	-5.33	-1.19	0.41	72.11	6.58
-0.5	-10.06	1.3	-2.8	0.41	45.4	0.43
0.4	-6.07	-4.48	-1.79	0.41	61.83	4.91
0.57	-1.74	-1.36	-0.41	0.41	85.53	1.65
5.33	-13.28	-3.24	-3.41	0.41	35.05	3.21

-0.29	-3.18	0.01	-0.91	0.41	76.83	0.81
1.16	-6.37	-4.15	-1.88	0.41	60.41	4.73
-0.28	-3.15	0.2	-0.9	0.41	77	0.72
-1.02	-13.65	-2.77	-3.5	0.41	34.03	2.19
0.33	-6.09	-3.28	-1.79	0.41	61.72	3.31
0.27	-13.59	-5.74	-3.48	0.42	34.18	8.21
12.33	-21.33	-3.79	-4.5	0.42	17.83	4.3
-1.21	-6.23	-4.05	-1.84	0.42	61.05	4.57
-1.22	-6.81	-0.99	-2.01	0.42	58.39	1.42
1.57	-1.3	-4.84	-0.25	0.42	88.22	5.84
5.56	-13.67	-2.26	-3.47	0.42	33.97	2.1
0.93	-3.57	-1.2	-1.04	0.42	74.59	1.43
1.41	-2.77	0	-0.78	0.42	79.2	0.8
5.04	-13.48	-4	-3.46	0.43	34.48	4.35
6.84	-17.55	-4.03	-4.06	0.43	24.61	3.99
7.08	-12.96	-0.3	-3.35	0.43	35.95	0.73
-0.16	-9.85	-2.23	-2.74	0.43	46.07	2.26
-1.39	-11.92	-1.23	-3.16	0.43	39.08	1.46
0.7	-11.54	-0.22	-3.08	0.43	40.3	0.97
3.11	-4.88	-4.78	-1.44	0.43	67.52	5.73
1.66	-1.27	-4.96	-0.23	0.43	88.36	6.19
1.79	-0.88	-5.45	-0.08	0.43	90.95	7.32
1.84	0.43	-4.7	0.42	0.43	100	5.31
1.22	-5.6	-1.13	-1.67	0.43	63.98	1.47
5.97	-14.11	-1.41	-3.54	0.43	32.73	1.66
-0.86	-3.69	-1.19	-1.07	0.43	73.88	1.19
0.89	-2.22	-1.09	-0.57	0.43	82.44	1.49
0.84	-3.6	-1.33	-1.03	0.43	74.4	1.62
2.79	-23.65	-0.83	-4.7	0.44	14.51	1.27
6.63	-17.17	-4.27	-4	0.44	25.4	4.44
2.03	-14.11	0.96	-3.26	0.44	31.7	0.7
1.94	-11.79	2.81	-1.2	0.44	31.73	1.12
13.01	-21.39	-3.44	-4.5	0.44	17.71	3.56
11.92	-20.49	-3.68	-4.4	0.44	19.15	3.58
3.19	-4.66	-5.26	-1.37	0.44	68.65	6.22
3.18	-5.09	-5.03	-1.51	0.44	66.47	6.23
3.96	-17.05	-5.07	-4	0.44	25.65	6.26
-1.56	-12.08	-1.24	-3.17	0.44	38.55	1.52
0.33	-2.91	-1.32	-0.8	0.44	78.27	1.47
-1.28	-6.82	-1.85	-2	0.44	58.26	1.95
6.94	-10.79	4.07	-0.32	0.45	31.77	1.13
0.84	-2.49	-2.67	-0.66	0.45	80.69	2.99
0.65	-3.67	-1.88	-1.06	0.45	73.88	1.95
0.62	-3.71	-1.9	-1.06	0.45	73.66	1.97

-1.19	-14.71	-3	-3.65	0.45	31.13	3.14
2.55	-6.66	-2.17	-1.93	0.45	58.93	2.27
1.35	-14.33	-1.64	-3.58	0.45	32.1	1.78
0.65	-5.25	-0.22	-1.55	0.45	65.62	0.93
-1.57	-12.13	-1.42	-3.18	0.45	38.37	1.6
-1.53	-12.14	-1.39	-3.18	0.45	38.34	1.67
-1.02	-2.88	-0.79	-0.79	0.45	78.41	1.29
0.29	-3.13	-1.7	-0.87	0.45	76.95	1.77
6.26	0.36	-0.65	0.42	0.45	99.35	1.02
0.66	-3.74	-2.45	-1.07	0.46	73.44	2.51
4.2	-20.41	-4.44	-4.39	0.46	19.24	4.93
-1.12	-2.8	-1.58	-0.75	0.46	78.8	1.65
-1.16	-3.49	-1.64	-1	0.46	74.82	1.42
-1.47	-8.65	-1.93	-2.43	0.46	50.46	2.03
0.2	-6.18	-3.34	-1.79	0.46	61.09	3.39
0.35	-10.27	-2.95	-2.82	0.47	44.45	3.12
3.27	-5.27	-4.96	-1.54	0.47	65.43	6.27
1.27	-7.42	-0.19	-2.12	0.47	55.5	0.91
0.5	-2.25	-2.66	-0.56	0.47	81.96	2.78
2.76	-10.78	-2.74	-2.9	0.47	42.69	2.76
2.9	-10.69	-2.81	-2.88	0.47	42.98	2.9
1.12	-3.51	-0.29	-0.99	0.47	74.65	0.96
4.85	-12.11	-5.8	-3.16	0.47	38.37	8.26
4.65	-12.31	-5.6	-3.2	0.47	37.78	7.8
-1.51	-8.65	-2.04	-2.43	0.47	50.46	2.02
1.1	-2.9	0.23	-0.79	0.47	78.11	0.76
1.45	-2.85	-0.77	-0.77	0.47	78.44	1.25
0.25	-1.86	0.15	-0.43	0.47	84.38	0.84
0.96	-7.27	-2.67	-2.08	0.47	56.12	2.71
1.05	-7.11	-2.87	-2.04	0.47	56.83	3.05
-0.44	-6.86	-3.36	-1.98	0.48	57.9	3.8
7.81	0.39	-2.31	0.44	0.48	99.41	1.92
1.74	-9.85	0.13	-2.7	0.48	45.87	0.89
1.73	-15.88	-2.61	-3.81	0.48	28.19	2.64
-0.62	-8.37	1.38	-2.37	0.48	51.55	0.46
17.51	-17.91	-2.71	-4.08	0.48	23.78	2.82
1.15	-3	-4.32	-0.82	0.48	77.51	4.78
-0.96	-3.35	0.3	-0.92	0.48	75.48	0.71
0.63	-5.92	-3.13	-1.7	0.48	62.24	3.3
0.9	-7.83	-2.99	-2.22	0.48	53.75	3.03
0.25	0.02	-0.33	0.3	0.48	96.71	1
7.14	-17.53	-3.68	-4.02	0.49	24.55	4.4
12.76	-21.14	-3.43	-4.43	0.49	18.02	3.68
4.27	-18.52	-4.94	-4.16	0.49	22.59	6.31

-0.86	-13.12	-2.88	-3.36	0.49	35.33	2.6
0.39	0.04	-0.59	0.31	0.49	96.77	1.15
11.41	-14.28	6.63	-0.22	0.5	22.08	0.39
7.61	-11.86	-1.68	-3.1	0.5	39.06	1.73
1.59	-12.42	-0.51	-3.22	0.5	37.36	1.09
3.89	-6.13	-4.04	-1.76	0.5	61.13	4.55
1.68	-1.15	-5.2	-0.15	0.5	88.66	6.77
1.76	-0.98	-4.72	-0.09	0.5	89.85	5.76
1.77	-0.94	-4.6	-0.07	0.5	90.08	5.53
1.77	-0.92	-4.85	-0.06	0.5	90.22	6.04
1.93	0.5	-4.21	0.5	0.5	100	4.75
0.43	-5.53	-0.17	-1.6	0.5	63.96	0.81
5.64	-12.95	-3.38	-3.3	0.5	35.78	3.47
0.36	-4.27	-0.04	-1.2	0.5	70.37	0.9
0.39	-4.17	-0.4	-1.18	0.5	70.9	1.06
-1.49	-8.91	-1.6	-2.47	0.5	49.32	1.82
-1.62	-12.51	-3.23	-3.22	0.51	37.07	3.75
4.18	-6.54	-4.82	-1.87	0.51	59.23	6
1.88	-15.47	-2.56	-3.73	0.51	29.1	2.72
0.81	-1.37	-1.97	-0.23	0.51	87.16	2.14
1.75	-0.81	-5.25	-0.01	0.51	90.86	6.82
0.37	-4.24	0.09	-1.19	0.51	70.51	0.85
4.38	-13.6	-4.49	-3.41	0.52	33.92	5.06
-1.46	-7.61	-0.08	-2.13	0.52	54.49	0.97
5.27	-14.09	-3.81	-3.5	0.53	32.54	4.11
7.62	-12.42	-2.13	-3.18	0.53	37.28	2.3
4.73	-2.83	-4.47	-0.73	0.53	78.21	5.2
3.2	-23.9	-1.82	-4.66	0.53	14.09	2.04
3.38	-5.41	-4.75	-1.54	0.53	64.47	6.03
-1.48	-12.35	-1.26	-3.17	0.53	37.49	1.65
1.37	-5.56	-2.14	-1.6	0.54	63.67	2.38
-1.72	-12.06	-4.22	-3.13	0.54	38.32	4.55
13.03	-21.74	-3.94	-4.46	0.54	17.04	4.52
-0.03	-9.73	-2.4	-2.64	0.54	46.12	2.56
1.98	-0.75	-6.17	0.03	0.54	91.07	9.64
5.84	-13.8	-1.3	-3.41	0.54	33.29	1.69
-1.46	-8.9	-2.01	-2.44	0.54	49.24	2.17
-0.58	-2.9	0.98	-0.74	0.54	77.75	0.73
1.22	-0.23	0.85	0.23	0.54	94.55	0.64
-0.15	-19.03	-3.47	-4.19	0.55	21.52	3.98
-1.86	-15.28	-1.61	-3.69	0.55	29.47	1.9
0.66	-3.77	-2.6	-1.03	0.55	72.86	2.65
0.65	-3.94	-2.58	-1.08	0.55	71.92	2.71
6.56	-0.65	-3.64	0.08	0.55	91.63	3.63

1.64	-1.4	-3.04	-0.2	0.55	86.81	3.34
17.54	-17.7	-2.54	-4.01	0.55	24.1	2.85
0.77	-4.47	-0.42	-1.24	0.55	69.06	1.09
-1.44	-12.15	-2.19	-3.12	0.55	38.03	2.39
1.16	-2.99	0.53	-0.77	0.55	77.15	0.67
1.43	-2.75	0.22	-0.68	0.55	78.6	0.78
-1.12	-13.57	-2.09	-3.4	0.55	33.92	2.43
0.33	-1.71	0.48	-0.33	0.55	84.82	0.73
0.5	0.04	-0.64	0.35	0.55	96.33	1.23
-0.17	-8.37	0.12	-2.32	0.56	51.22	1.09
6.42	0.53	-1.78	0.55	0.56	99.76	1.95
1.57	-2.7	-1.14	-0.66	0.56	78.82	1.44
0.62	-5.69	-3.24	-1.59	0.56	62.95	3.52
0.57	-6.41	-2.89	-1.78	0.56	59.6	3.15
-1.02	-6.17	-3.92	-1.73	0.57	60.67	4.73
-0.97	-11.87	-2.04	-3.07	0.57	38.83	1.83
-1.4	-7.75	-0.07	-2.14	0.57	53.74	0.97
0.39	-4.2	0.93	-1.14	0.57	70.42	0.57
0.42	-4.09	0.8	-1.1	0.57	70.97	0.62
2.48	-2.82	-4.85	-0.69	0.58	78.03	6.66
12.93	-21.53	-3.94	-4.42	0.58	17.32	4.13
1.09	-11.67	0.24	-3.01	0.58	39.46	0.89
0.67	-4.23	-1.43	-1.16	0.58	70.17	1.77
4.43	-6.9	-4.68	-1.93	0.58	57.3	6.27
-1.32	-7.77	0.29	-2.14	0.58	53.59	0.88
9.05	-10.62	-4.55	-2.81	0.58	42.88	5.35
0.23	-4.24	-0.35	-1.14	0.58	70.17	1
-1.48	-8.89	-1.74	-2.42	0.58	49.16	1.91
3.81	-11.54	-1.9	-2.98	0.59	39.85	2.12
1.11	-12.96	-0.28	-3.25	0.59	35.52	1.02
1.45	-11.5	-0.5	-2.97	0.59	39.96	1.11
0.86	-4.6	-1.4	-1.26	0.59	68.22	1.79
3.89	-20.13	-4.08	-4.26	0.59	19.53	4.63
1.92	-0.83	-5.25	0.03	0.59	90.24	7.2
7.45	-12.3	-4.51	-3.11	0.59	37.47	4.83
-1.21	-17.07	-1.38	-3.89	0.6	25.33	1.75
12.89	-21.73	-4.19	-4.42	0.6	16.99	4.92
12.89	-21.11	-3.69	-4.36	0.6	17.93	4.1
4.73	-3.54	-3.94	-0.91	0.6	73.81	4.7
1.97	-0.71	-6.02	0.08	0.6	90.94	9.19
2	-0.71	-6.12	0.08	0.6	91	9.6
0.69	-4.18	0.31	-1.11	0.6	70.34	0.85
2.72	-3.8	-0.76	-1	0.6	72.4	1.19
1.14	-3.05	0.46	-0.76	0.6	76.58	0.77

6.01	-11.8	-4.53	-3.02	0.6	38.99	5.7
7.22	-1.29	-4.77	-0.13	0.61	87.11	5.71
0.44	-13.54	-5.1	-3.35	0.61	33.84	7.44
0.12	-9.24	-1.75	-2.49	0.61	47.69	1.98
1.39	-5.7	-1.52	-1.58	0.61	62.68	1.85
2.66	-6.74	-1.76	-1.86	0.61	57.9	2.06
4.09	-20.45	-4	-4.29	0.61	18.98	4.55
9.34	-13.29	-3.17	-3.29	0.61	34.56	3.78
2.13	-2.58	-1.58	-0.59	0.61	79.22	1.64
-1.67	-12.4	-1.33	-3.12	0.61	37.11	1.7
0.68	-1.43	0.18	-0.18	0.61	86.19	0.9
7.74	-12.36	-0.55	-3.12	0.62	37.23	1.56
0.25	-8.95	-1.7	-2.41	0.62	48.76	1.97
-1.6	-12.38	-1.59	-3.12	0.62	37.14	1.96
1.17	-3.04	0.09	-0.74	0.62	76.53	0.91
5.79	-11.36	-4.84	-2.91	0.62	40.32	6.31
5.35	-11.74	-5.1	-2.98	0.62	39.11	6.85
-1.47	-8.87	-1.45	-2.38	0.62	49.08	1.79
2.66	-12.74	0.82	-3.18	0.63	36.07	0.66
-1.4	-5.26	-2.02	-1.43	0.63	64.71	2.31
2.03	-17.82	-2.48	-3.97	0.63	23.7	2.76
-0.5	-19.37	-3.51	-4.16	0.63	20.79	4.02
1.23	-20.29	-4.45	-4.26	0.63	19.19	5.8
-0.81	-4.47	0.54	-1.2	0.63	68.7	0.92
0.58	-0.52	-2.59	0.17	0.63	92.01	3.03
1.36	-3.26	-0.59	-0.81	0.63	75.16	1.21
-1.38	-12.21	-2.33	-3.08	0.63	37.62	2.55
-1.41	-12.29	-1.95	-3.09	0.63	37.41	2.18
-1.61	-12.36	-1.23	-3.11	0.63	37.2	1.57
0.49	-6.62	-2.25	-1.8	0.63	58.32	2.52
6.96	-3.06	-5.25	-0.74	0.64	76.26	7.62
12.26	-11.55	-3.21	-2.96	0.64	39.65	3.59
-0.18	-19.86	-3.58	-4.21	0.64	19.93	4.21
0.07	-19.82	-4.01	-4.2	0.64	19.99	4.95
6.5	0.63	-1.78	0.64	0.64	99.93	2.07
-0.16	-3.05	0.37	-0.72	0.64	76.34	0.86
-1.17	-2.88	1.22	-0.66	0.64	77.33	0.53
-1.09	-13.87	-2.07	-3.39	0.64	32.88	2.37
0.49	-1.69	0.67	-0.27	0.64	84.4	0.71
1.24	-7.2	-2.67	-1.96	0.64	55.76	2.9
0.68	-1.18	-0.44	-0.07	0.64	87.59	1.16
0.62	-1.16	-0.18	-0.07	0.64	87.75	1.08
7.39	-12.11	-1.86	-3.05	0.65	37.86	2.05
3.15	-11.32	-1.09	-2.9	0.65	40.36	1.48

0.82	-6.92	-2.09	-1.89	0.65	56.95	2.17
17.48	-17.51	-2.61	-3.92	0.65	24.32	2.5
2.04	0.65	-3.86	0.66	0.65	100	4.38
5.81	-13.03	-2.63	-3.21	0.65	35.17	2.98
0.66	-4.13	0.57	-1.06	0.65	70.38	0.72
0.73	-3.95	-0.12	-1.03	0.65	71.34	0.99
1.48	-2.64	-0.29	-0.59	0.65	78.6	0.99
0.16	-12.7	1.75	-2.01	0.66	31.79	1.07
3.5	-5.53	-4.3	-1.49	0.66	63.28	5.39
-1.15	-6.68	-0.69	-1.82	0.66	57.96	1.35
1.96	-0.7	-5.45	0.12	0.66	90.57	7.77
0.58	-2.23	-2.18	-0.44	0.66	81.02	2.5
-1.38	-12.21	-1.88	-3.06	0.66	37.54	2.15
-1.45	-8.88	-1.47	-2.37	0.66	48.89	1.88
0.02	-0.28	-0.5	0.28	0.66	93.43	1.25
3.71	-23.61	-1.34	-4.54	0.67	14.31	1.83
4.1	-6.5	-4.79	-1.76	0.67	58.73	6.37
9.3	-11.27	-3.37	-2.88	0.67	40.48	3.58
-0.62	-8.36	0.62	-2.25	0.67	50.87	0.55
-1.4	-7.74	-0.12	-2.08	0.67	53.38	0.99
4.63	-19.13	-5.08	-4.11	0.67	21.16	7.19
2.06	-0.7	-5.49	0.12	0.67	90.57	8.16
3.7	-7.77	-3.36	-2.07	0.67	53.24	3.47
7.39	-12.39	-0.75	-3.09	0.68	36.97	1.52
0.61	-7.53	-2.21	-2.05	0.68	54.18	2.12
0.42	-10.3	-2.73	-2.7	0.68	43.66	3.21
-0.99	-8.67	-0.14	-2.32	0.68	49.61	1.13
-1.46	-7.57	-0.81	-2.02	0.68	54.04	1.31
8.92	-12.86	-3.58	-3.17	0.68	35.59	4.33
7.72	-12.78	-3.94	-3.15	0.68	35.8	4.48
0.45	-4.01	0.69	-1.01	0.68	70.8	0.67
-1.43	-12.13	-0.96	-3.03	0.68	37.75	1.62
1.47	-0.09	0.79	0.37	0.68	94.62	0.62
5.08	-15.99	-4.17	-3.68	0.69	27.51	5.2
3.48	-10.72	3.82	-0.27	0.69	31.83	1.39
7.38	-12.37	0.26	-3.07	0.69	36.97	0.82
1.45	-3.02	-2.29	-0.7	0.69	76.27	2.64
-1.34	-7.74	-0.48	-2.06	0.69	53.3	1.2
-1.41	-13.37	-1.86	-3.25	0.69	34.12	1.86
0.59	-4.22	0.47	-1.07	0.69	69.73	0.76
1.71	-5.12	-1.81	-1.35	0.69	65.14	1.84
1.08	-3.04	0.11	-0.7	0.69	76.14	0.96
2.62	-1.61	0.1	-0.2	0.69	84.59	0.79
0.42	-20.46	-3.77	-4.23	0.7	18.83	4.62

3.44	-3.91	-2.14	-0.97	0.7	71.25	2.36
-1.58	-12.64	-2.84	-3.12	0.71	36.14	3.51
0.2	-13.76	-4.89	-3.35	0.71	33.01	6.1
-1.69	-15.8	-2.34	-3.66	0.71	27.91	2.83
0.4	-8.82	-2.26	-2.33	0.71	48.95	2.61
1.48	-3.07	-3.17	-0.7	0.71	75.87	3.64
1.74	-1.4	-3.04	-0.11	0.71	85.81	3.75
17.49	-17.26	-1.67	-3.84	0.71	24.72	2.08
0.94	-20.17	-4.44	-4.19	0.71	19.29	5.64
5.92	-13.01	-3.26	-3.17	0.71	35.09	3.75
-1.8	-15.42	-2.16	-3.6	0.72	28.79	2.55
-0.69	-3.44	-1.55	-0.82	0.72	73.75	1.62
2.21	-16.84	-2.55	-3.79	0.72	25.58	2.91
6.53	0.7	-1.24	0.71	0.72	99.87	1.62
0.45	-6.59	-2.48	-1.74	0.72	58.09	2.87
0.62	-1.7	0.59	-0.21	0.72	83.82	0.78
1.43	-6.85	-3.02	-1.81	0.72	56.94	3.55
1.89	-12.01	-0.01	-2.98	0.73	37.96	1.01
4.46	-4.07	-4.82	-1	0.73	70.29	6.11
-1.09	-14.77	-2.9	-3.48	0.73	30.35	3.42
-1.43	-7.66	1.06	-2.01	0.73	53.48	0.62
0.51	-4.26	0.76	-1.05	0.73	69.27	0.69
-1.1	-13.54	-2.4	-3.27	0.73	33.56	2.62
4.5	-11.58	4.82	0.13	0.74	27.86	1.46
7	-17.55	-3.23	-3.85	0.74	24.05	3.57
0.05	-12.75	1.44	-2.12	0.74	32.01	1.17
6.91	-11.69	-0.41	-2.91	0.74	38.92	1.33
3.56	-5.1	-4.6	-1.32	0.74	65.02	5.79
0.59	-4.24	0.34	-1.05	0.74	69.33	0.83
0.75	-1.73	-0.36	-0.22	0.74	83.55	1.23
0.56	0.22	0.19	0.53	0.74	96.36	0.97
0.53	-13.06	2.08	-2.4	0.75	32.03	0.74
3.74	-5.9	-4.3	-1.55	0.75	61.12	5.33
4.01	-6.27	-4.41	-1.65	0.75	59.44	5.78
5.06	-18.57	-5.09	-4	0.75	22.06	7.81
2.12	-0.65	-5.26	0.19	0.75	90.37	7.73
0.63	-3.59	0.59	-0.84	0.75	72.75	0.82
0.87	-1.2	0.97	-0.02	0.75	86.76	0.7
1.05	-3.62	-1.21	-0.84	0.75	72.58	1.97
9.1	-12.55	-3.38	-3.05	0.76	36.29	3.93
0.82	-5.58	1.41	-1.46	0.76	62.57	0.66
-0.02	0.2	-1.35	0.54	0.76	95.97	1.83
1.74	-3.05	-1.51	-0.65	0.76	75.69	1.8
0.99	-2.3	-1.89	-0.4	0.77	79.98	2.41

0.94	-4.54	-1.76	-1.13	0.77	67.68	2.2
2.74	-6.74	-1.48	-1.76	0.77	57.23	1.96
3.1	-15.1	-0.73	-3.49	0.77	29.47	1.55
11.56	-10.34	-4.14	-2.64	0.77	43.26	4.92
0.82	-4.22	-0.16	-1.02	0.77	69.31	1.09
1.49	-5.54	-1.98	-1.44	0.78	62.64	2.48
0.27	-18.84	-3.07	-4.01	0.78	21.51	3.8
10.63	-2.83	-2.63	-0.58	0.78	76.77	2.81
4.88	-3.36	-4.29	-0.75	0.78	73.85	5.25
7.4	-0.8	-4.17	0.16	0.78	89.13	5.01
17.05	-17.66	-2.17	-3.85	0.78	23.78	3.18
1.36	-3.03	-3.75	-0.64	0.78	75.67	4.31
3.92	-11	-2.86	-2.74	0.78	41	3.4
6.23	-13.88	-1.61	-3.27	0.78	32.53	1.81
5.74	-13.97	-1.58	-3.29	0.78	32.29	1.83
0.73	-4.17	0.52	-0.99	0.78	69.5	0.78
1.45	-14.3	2.01	-2.28	0.79	27.82	1.37
1.84	-11.54	0.06	-2.85	0.79	39.25	1.04
1.47	-11.46	0.51	-2.83	0.79	39.49	0.82
1.47	-2.92	-2.12	-0.59	0.79	76.22	2.48
1.52	-3.11	-3.25	-0.67	0.79	75.17	3.98
4.06	-6.38	-4.4	-1.65	0.79	58.75	5.67
2.98	-10.65	-1.34	-2.68	0.79	42.15	1.71
0.63	-2.17	-2.14	-0.34	0.79	80.64	2.58
-0.96	-2.74	-1.02	-0.54	0.79	77.26	1.66
-0.05	0.28	-1.54	0.58	0.79	96.38	1.84
6.24	-13.84	-0.54	-3.26	0.79	32.6	1.51
3.66	-2.72	-0.42	-0.53	0.79	77.37	1.31
1.82	-2.61	0.54	-0.49	0.79	77.98	0.76
0.46	-6.57	-2.83	-1.69	0.79	57.91	3.39
3.21	-1.65	-0.13	-0.15	0.79	83.7	1.06
5.44	0	-0.22	0.47	0.79	94.46	1.39
-1.72	-15.66	-2.3	-3.58	0.8	28.07	2.62
2.4	-17.59	-2.84	-3.83	0.8	23.87	3.42
2.16	-0.6	-5.2	0.24	0.8	90.36	7.84
0.56	-1	0.39	0.09	0.8	87.77	0.86
-1.1	-5.36	-2.07	-1.35	0.81	63.42	2.57
4.09	-11.35	-2.71	-2.79	0.81	39.79	3.49
5.46	-10.35	-2.84	-2.58	0.81	43.09	3.24
2.14	-6.4	-3.9	-1.63	0.81	58.56	5.13
-1.08	-13.83	-2.67	-3.27	0.81	32.6	2.55
0.5	-6.66	-3.1	-1.69	0.81	57.43	3.87
-0.52	-14.13	2.18	-2.12	0.82	27.81	1
2.63	-11.32	0.08	-2.79	0.82	39.88	0.91

0.8	-4.1	0.35	-0.95	0.82	69.68	0.88
1.02	-4.4	-1.2	-1.05	0.83	68.08	1.9
0.15	-9.32	-1.12	-2.37	0.83	46.66	1.76
3.18	-3.79	-0.92	-0.85	0.83	71.22	1.38
6.23	-11.86	-4.23	-2.88	0.83	38.16	5.56
6.21	-13.52	0.69	-2.82	0.84	32.06	0.82
4.84	-24.08	-2.56	-4.47	0.84	13.57	3.09
12	-21.67	-3.77	-4.26	0.84	16.79	4.58
-1.37	-7.63	0.27	-1.94	0.84	53.19	0.85
2.58	-20.52	-3.47	-4.14	0.84	18.53	4.28
2.17	-0.55	-5.52	0.28	0.84	90.43	8.73
2.19	-0.53	-5.78	0.29	0.84	90.57	9.57
0.66	-2.16	-1.91	-0.31	0.84	80.38	2.4
5.87	-3.56	-0.29	-0.78	0.84	72.41	0.86
-1.25	-12.28	-0.88	-2.95	0.84	36.84	1.52
3.98	-7.17	-3.22	-1.81	0.84	55.09	3.66
4.13	0.41	-0.39	0.66	0.84	96.94	1.12
9.41	-6.01	9.76	3.74	0.85	32.15	1.27
1.58	-3.15	-2.88	-0.63	0.85	74.59	3.7
2.84	-6.53	-2.49	-1.65	0.85	57.83	2.62
2.76	-6.85	-1.24	-1.74	0.85	56.42	1.81
2.87	-7.07	-1.31	-1.79	0.85	55.46	1.92
1.55	-19.57	-3.66	-4.03	0.85	20.1	4.66
1.8	-20.01	-3.37	-4.08	0.85	19.36	4.3
2.3	-20.86	-3.75	-4.17	0.85	17.98	4.76
0.64	-0.33	-2.51	0.37	0.85	91.78	3.2
1.62	-3.14	-0.13	-0.64	0.85	74.69	1.04
12.88	-21.63	-4.01	-4.24	0.86	16.82	4.42
-0.55	-6.08	-3.78	-1.52	0.86	59.8	5.08
7.62	-1.12	-3.91	0.08	0.86	86.63	5.1
-0.93	-2.72	-1.1	-0.48	0.86	77.01	1.8
1.98	-1.33	-2.13	0.01	0.87	85.24	2.68
7.21	-2.78	-4.65	-0.5	0.88	76.54	6.95
7.21	-2.23	-4.67	-0.31	0.88	79.76	6.53
-0.77	-15.94	-2.7	-5.09	0.88	32.27	1
0.33	-13.1	1.73	-2.51	0.88	32.29	0.8
-1.78	-15.67	-2.02	-3.54	0.88	27.89	2.59
0.53	-8.58	-2.46	-2.16	0.88	49.24	3
6.38	-11.61	-0.19	-2.8	0.88	38.79	1.04
4.78	-4.33	-4.46	-0.99	0.88	68.2	6.14
0.42	-9.22	0.45	-2.31	0.88	46.87	0.91
4.56	-6.91	-4.15	-1.74	0.88	56.06	5.91
9.7	-12.75	-3.09	-3.01	0.88	35.38	3.9
2.38	-8.11	0.53	-2.03	0.89	51.03	0.63

-1.04	-13.85	-2.51	-3.22	0.89	32.34	2.91
10.16	-14.25	5.56	-0.19	0.9	22.09	0.98
5.54	-24.41	-2.03	-4.46	0.9	13.12	2.78
5.65	-11.61	0.14	-2.79	0.9	38.73	1.16
2.22	-0.45	-5.76	0.36	0.9	90.73	9.67
2.31	0.9	-3.85	0.9	0.9	100	4.85
1.42	-2.88	0.07	-0.52	0.9	75.86	0.99
2.4	-2.62	-0.02	-0.43	0.9	77.37	1.18
0.54	-6.59	-3.3	-1.62	0.9	57.39	4.32
3.78	-1.67	-0.61	-0.1	0.9	82.99	1.41
4.28	-17.45	0.99	-3.01	0.91	22.13	1.34
0.19	-13.15	1.12	-2.57	0.91	32.31	1.02
3.33	-12.27	1.31	-1.78	0.91	32.34	1.44
0.63	-13.72	-4.62	-3.19	0.91	32.64	7.12
7.2	-11.8	-0.38	-2.82	0.91	38.11	1.38
-0.18	-8.63	0.78	-2.16	0.91	48.96	0.77
-0.7	-8.59	0.04	-2.15	0.91	49.1	1.18
-1.27	-7.72	0.33	-1.92	0.91	52.55	0.98
2.31	-0.54	-5.56	0.33	0.91	90.03	9.49
8.9	-12.7	-2.81	-2.98	0.91	35.44	3.65
-0.45	-2.8	-0.27	-0.48	0.91	76.25	1.21
4.38	-8.25	-3.19	-2.04	0.91	50.4	3.82
-0.25	-6.84	-2.26	-1.7	0.92	56.16	2.95
0.88	-1.62	-0.77	-0.07	0.92	83.16	1.6
1.11	-2.23	-1.51	-0.28	0.92	79.5	2.23
2.52	-13.87	-1.5	-3.21	0.92	32.22	1.67
-1.37	-12.29	-0.3	-2.9	0.92	36.62	1.23
-0.03	-11.95	-1.03	-2.86	0.92	37.63	1.86
-1.21	-8.29	-0.46	-2.07	0.92	50.23	1.45
-1.29	-8.23	0.23	-2.05	0.92	50.48	0.99
0.89	-1.03	-0.88	0.15	0.92	86.78	1.65
0.26	-9.29	-1.17	-2.3	0.92	46.44	1.89
-0.52	-3.12	-0.33	-0.58	0.92	74.38	1.13
-0.94	-8.62	-0.67	-2.14	0.93	48.93	1.48
0.7	-2.13	-1.46	-0.24	0.93	80.05	2.08
1.3	-2.91	0.47	-0.51	0.93	75.51	0.87
1.2	-2.88	0.96	-0.5	0.93	75.7	0.66
-0.82	-2.61	-0.15	-0.4	0.93	77.27	1.17
-0.73	-2.55	-0.38	-0.38	0.93	77.55	1.3
1.35	-11.21	-1.39	-2.71	0.93	39.92	1.72
0.95	-1.15	1.86	0.11	0.93	86.01	0.44
1.61	-5.41	-1.97	-1.3	0.94	62.56	2.74
5.24	-2.85	-4.04	-0.49	0.94	75.84	5.12
0.02	-3.12	-2.39	-0.58	0.94	74.28	3.07

-1.02	-11.86	1.12	-2.81	0.94	37.84	0.66
4.37	-11.43	0.08	-2.73	0.94	39.18	1.13
5	-3.54	-3.96	-0.71	0.94	71.99	5.02
5.59	0.11	-5.29	0.6	0.94	94.16	8.78
13.59	-11.32	-2.49	-2.73	0.94	39.54	3.04
1.33	-2.97	0.75	-0.53	0.94	75.18	0.81
0.84	-1.07	-0.25	0.15	0.94	86.45	1.21
-1.4	-11.87	-4.07	-2.83	0.95	37.81	5.13
0.14	-3.21	-2.46	-0.6	0.95	73.76	3.12
-0.84	-5.5	-1.81	-1.3	0.95	62.09	2.47
1.87	-1.14	-2.5	0.13	0.95	85.93	3.16
-0.7	-11.53	-1.83	-2.75	0.95	38.83	2.31
1.77	-7.73	-2.55	-1.9	0.95	52.35	3.33
0.96	0.1	0.36	0.61	0.95	94.08	0.92
0.67	-8.42	-2.86	-2.08	0.96	49.57	3.74
-0.23	-8.69	1.63	-2.14	0.96	48.56	0.5
-0.84	-8.61	0.06	-2.12	0.96	48.88	1.15
1	-1	-1.31	0.19	0.96	86.74	2.02
0.48	0.48	0.14	0.76	0.96	96.58	1.06
-1.52	-15.84	-2.45	-3.5	0.97	27.32	3.11
2.23	-11.14	-0.16	-2.65	0.97	39.99	1.18
4.78	-6.5	-3.7	-1.57	0.97	57.49	5.38
-1	-14.73	-2.82	-3.31	0.97	29.92	3.65
1.26	-3.59	0.84	-0.7	0.97	71.64	0.77
2.45	-6.32	-4.04	-1.51	0.97	58.25	5.68
7.45	-17.34	-3.01	-3.67	0.98	24.07	3.73
-0.39	-6.02	-3.52	-1.43	0.98	59.57	4.77
-0.6	-8.48	1.04	-2.08	0.98	49.29	0.57
2.75	-17.71	-3.04	-3.74	0.98	23.34	4.05
3.34	-17.12	-0.69	-3.65	0.98	24.53	1.6
0.63	-6.56	-3.32	-1.57	0.98	57.15	4.46
-0.04	-12.7	1.44	-2.16	0.99	32.34	1.11
-0.8	-11.83	1.35	-2.77	0.99	37.82	0.61
-1.35	-8.25	0.3	-2.01	0.99	50.12	0.96
0.79	-2.11	-0.89	-0.21	0.99	79.79	1.68
9.18	-12.62	-2.97	-2.92	0.99	35.48	3.69
-0.29	-14.2	-0.26	-3.22	0.99	31.23	1.79
-0.07	-8.7	-0.07	-2.12	1	48.37	1.2
-0.74	-8.68	0.44	-2.11	1	48.45	0.91
-0.17	-8.61	-0.22	-2.1	1	48.71	1.23
-1.25	-7.7	0.04	-1.86	1	52.3	1.12
-1.49	-7.62	0.18	-1.83	1	52.62	0.94
6.03	-12.97	-3.2	-2.98	1	34.46	3.75
2.35	-5.18	-1.14	-1.18	1	63.41	1.76

-0.38	-2.53	-0.4	-0.34	1	77.34	1.42
-1.13	-12.26	-0.25	-2.84	1.01	36.48	1.31
-0.07	-3.05	-1.44	-0.51	1.01	74.32	2.11
5.23	-11.66	-0.05	-2.73	1.01	38.27	1.2
0.77	-2.06	-1.13	-0.17	1.01	79.93	1.85
0.82	-2.11	-1.46	-0.19	1.01	79.66	2.18
4.37	-11.06	-1.85	-2.61	1.01	40.14	2.65
0.93	-0.93	-0.79	0.25	1.01	86.86	1.59
3.41	-3.57	-0.58	-0.67	1.01	71.52	1.45
1.33	-2.92	1.62	-0.47	1.01	75.09	0.52
2.11	-2.54	1.22	-0.34	1.01	77.18	0.61
0.22	-0.08	1.75	0.57	1.01	92.39	0.57
1.69	-5.28	-1.48	-1.21	1.02	62.8	2.28
5.42	-16.62	-4.12	-3.56	1.02	25.49	5.62
-1.27	-7.71	0.53	-1.85	1.02	52.16	0.9
2.05	-20.3	-3.41	-4	1.02	18.67	4.24
0.73	-2.05	-1.39	-0.17	1.02	80.02	2.04
0.95	-0.9	-1.24	0.26	1.02	87.01	1.93
1.3	-0.94	-2.46	0.24	1.02	86.81	3.21
4	-3.94	-1.63	-0.78	1.02	69.48	2.18
6.52	1.02	-1.32	1.02	1.02	100	1.77
1.04	-1.17	0.49	0.16	1.02	85.32	0.9
4.84	-9.23	-3.65	-2.2	1.02	46.37	4.92
0.76	0.52	0.84	0.82	1.02	96.45	0.78
0.27	-3.14	-2.4	-0.52	1.03	73.72	3.07
6.64	-11.7	0.3	-2.73	1.03	38.09	1.01
0.02	-11.43	-2.09	-2.69	1.03	38.93	2.73
0.66	-9.19	-1.1	-2.21	1.03	46.49	1.84
0.86	-2.16	-1.7	-0.2	1.03	79.3	2.52
1.07	-0.94	-1.9	0.25	1.03	86.73	2.58
1.16	-0.92	-2.26	0.26	1.03	86.84	2.96
-0.47	-4.41	1.16	-0.94	1.03	67.08	0.89
1.38	-2.8	0.86	-0.41	1.03	75.59	0.75
2.68	-2.61	-0.49	-0.35	1.03	76.69	1.35
0.08	0.28	-0.53	0.73	1.03	94.76	1.31
0.91	0.52	0.59	0.82	1.03	96.4	0.89
0.66	0.52	1.19	0.81	1.03	96.43	0.63
-1.56	-15.99	-2.08	-3.47	1.04	26.84	2.79
-0.69	-5.48	-1.93	-1.23	1.04	61.8	2.69
2.09	-1.19	-2.24	0.17	1.04	85.1	3.12
14.22	-10.74	-2.61	-2.55	1.04	41.09	3.36
6.52	1.04	-0.81	1.04	1.04	100	1.61
0.75	-0.17	-2.76	0.55	1.04	91.64	3.85
7.31	-17.41	-2.36	-3.63	1.05	23.8	2.64

6.96	-2.45	-4.61	-0.29	1.05	77.47	7.13
-1.86	-15.63	-2.06	-3.42	1.05	27.64	2.74
0.49	-9.17	0.6	-2.19	1.05	46.46	0.91
16.32	-17.29	-3.25	-3.62	1.05	24.05	4.62
2.45	1.05	-3.38	1.05	1.05	100	4.38
1.25	-3.73	0.78	-0.7	1.05	70.48	0.79
2.82	-6.41	-4.18	-1.49	1.05	57.54	6.25
7.6	-10.95	-3.8	-2.56	1.05	40.39	5.84
-0.43	-1.96	0.5	-0.1	1.05	80.3	0.9
7.13	-14.53	4.6	-0.46	1.06	22.15	1.4
5.66	-13.67	-3.18	-3.09	1.06	32.42	3.81
-0.19	-5.95	-3.18	-1.35	1.06	59.54	4.45
5.8	0.48	-4.63	0.81	1.06	95.93	7.7
-1.16	-7.69	0.86	-1.82	1.06	52.11	0.8
1.2	-2.28	-3.34	-0.22	1.06	78.4	4.73
9.44	-12.64	-2.89	-2.88	1.06	35.25	3.79
-0.83	-3.8	0.28	-0.73	1.06	70.04	1.06
5.65	-10.73	-2.6	-2.5	1.06	41.07	3.11
-1.12	-12.36	0.29	-2.82	1.07	36.04	1.04
-0.05	-3	-1.63	-0.45	1.07	74.26	2.36
-0.68	-11.81	1.23	-2.72	1.07	37.65	0.73
1.09	-3.94	-0.57	-0.76	1.07	69.3	1.52
-0.03	-8.73	0.17	-2.08	1.07	48.03	1.11
0.77	-9.08	-0.6	-2.15	1.07	46.73	1.76
9.45	-12.15	-2.85	-2.78	1.07	36.63	3.33
2.14	-3.04	0.76	-0.47	1.07	74.01	0.84
3.39	-2.79	-4.92	-0.38	1.08	75.38	8.73
4.91	-13.85	-3.76	-3.09	1.08	31.9	4.65
4.12	-5.55	-4.19	-1.24	1.08	61.3	5.9
-0.02	-8.63	-0.15	-2.05	1.08	48.35	1.3
5.6	-18.31	-4.94	-3.75	1.08	22.02	8.63
2.36	-0.5	-5.85	0.45	1.08	89.16	10.99
0.85	-3.7	0.2	-0.67	1.08	70.46	1.24
4.32	0.62	-0.06	0.89	1.08	96.73	1.09
6.01	-13.84	-2.86	-3.1	1.09	31.9	3.83
0.84	-13.63	-4.51	-3.09	1.09	32.48	6.31
5.79	-3.4	-3.54	-0.58	1.09	72	4.92
0.5	-11.95	-1.51	-2.75	1.09	37.18	2.39
-0.33	-2.55	-0.4	-0.29	1.09	76.68	1.47
-0.66	-2.53	-0.28	-0.28	1.09	76.82	1.34
-0.62	-2.49	-0.24	-0.27	1.09	77.01	1.31
-0.53	-13.34	-1.79	-3.01	1.09	33.24	2.19
2.37	-3.39	-1.44	-0.55	1.09	72.03	2.05
5.91	-16.9	-3.96	-3.54	1.1	24.76	5.61

1.02	-1.47	-2.02	0.09	1.1	83.02	2.8
0.3	-3.21	-1.74	-0.51	1.1	72.97	2.49
4.99	-3.7	-3.6	-0.66	1.1	70.38	5.21
3.1	-7.35	-1.37	-1.71	1.1	53.35	2.23
-0.88	-2.61	-0.69	-0.3	1.1	76.3	1.54
0.3	-9.56	-1.56	-2.25	1.1	44.92	2.39
7.49	-11.28	-4.31	-2.59	1.1	39.21	6.79
7.39	-11.31	-4.49	-2.6	1.1	39.12	7.36
0.92	-0.94	1.61	0.28	1.1	86.28	0.51
1.92	-7.61	-2.05	-1.77	1.1	52.27	2.88
5.01	-8.94	-3.72	-2.08	1.1	47.14	5.15
1.03	0.59	1.66	0.89	1.1	96.37	0.56
0.62	-10.43	-2.09	-2.45	1.11	41.9	3.11
-1.43	-16.16	-1.55	-3.45	1.11	26.33	2.3
5.23	-3.34	-3.32	-0.54	1.11	72.25	4.34
4.29	-4.1	-1.66	-0.78	1.11	68.26	2.34
6.79	1.11	-0.98	1.1	1.11	100	2.06
-1.31	-8.74	-1.53	-2.05	1.11	47.84	2.36
-0.13	-6.81	-2.1	-1.56	1.12	55.49	2.97
6.98	-2.22	-4.09	-0.17	1.12	78.39	6.11
-1.66	-15.48	-1.93	-3.35	1.12	27.83	2.85
-0.95	-14.68	-2.21	-3.2	1.12	29.72	3.28
-0.67	-8.6	0.38	-2.02	1.12	48.36	1.13
9.03	-12.67	-1.87	-2.84	1.12	35.02	2.73
-0.5	-2.97	1.58	-0.41	1.12	74.16	0.58
1.22	0.59	0.11	0.9	1.12	96.2	1.22
1.11	0.59	0.35	0.9	1.12	96.23	1.03
6.72	1.12	-0.49	1.11	1.12	100	1.48
9.54	-13.85	6.6	0.12	1.13	22.15	0.62
7.31	-2.41	-4.47	-0.22	1.13	77.27	6.64
0.05	-8.96	-0.65	-2.09	1.13	46.97	1.71
0.63	-3.52	-0.28	-0.59	1.13	71.16	1.31
6.44	-3.57	-0.71	-0.6	1.14	70.86	1.6
5.72	-6.8	-0.98	-1.55	1.14	55.49	1.72
-0.63	-8.57	0.44	-2	1.14	48.39	1.01
-0.85	-3.69	0.36	-0.64	1.14	70.17	1.03
-0.22	-3.06	0.52	-0.42	1.14	73.59	0.94
-0.23	-3.01	0.31	-0.41	1.14	73.89	0.89
4.2	-1.51	-0.36	0.11	1.14	82.45	1.34
5.56	-8.96	-3.65	-2.06	1.14	46.91	5.33
-0.88	-12.44	0.25	-2.79	1.15	35.59	1.17
0.95	-7.88	-1.95	-1.82	1.15	51.01	2.85
1.75	-3.38	-2.21	-0.53	1.15	71.8	3.21
4.74	-6.67	-3.51	-1.5	1.15	56	4.77

-0.77	-8.56	0.94	-1.99	1.15	48.39	0.75
0.87	-2.11	-1.9	-0.11	1.15	78.9	2.85
0.89	-9.77	-0.88	-2.26	1.15	44.02	1.86
0.62	-3.36	0.36	-0.53	1.15	71.93	0.88
3.14	-6.28	-3.86	-1.39	1.15	57.7	5.82
1.23	-1.19	2.67	0.23	1.15	84.41	0.32
1.31	-2.06	-1.02	-0.08	1.16	79.12	1.94
0.06	-2.97	-1.21	-0.39	1.16	73.97	2.3
1.63	-3.03	-1.81	-0.41	1.16	73.62	2.67
0.18	-7.79	-0.19	-1.79	1.16	51.33	1.41
2.38	-0.49	-5.73	0.5	1.16	88.74	10.76
0.8	-9.57	-0.91	-2.22	1.16	44.7	1.85
1.33	-1.3	1.01	0.2	1.16	83.64	0.82
0.05	-3.41	-1.33	-0.53	1.17	71.55	2.29
1.67	-3.12	-2.34	-0.43	1.17	73.1	3.42
8.4	-0.95	-3.73	0.34	1.17	85.75	5.16
0.15	-9.27	-1.18	-2.14	1.17	45.72	1.94
7.1	-12.05	-3.98	-2.7	1.17	36.68	6.54
2.46	-2.43	0.78	-0.2	1.17	76.92	0.84
2.81	-2.37	0.27	-0.18	1.17	77.22	1.05
6.14	-16.93	-3.91	-3.5	1.18	24.55	6.09
0.73	-3.57	-0.86	-0.57	1.18	70.59	2
5.23	-4.78	-3.45	-0.94	1.18	64.5	5.06
0.08	-11.71	-1.25	-2.65	1.18	37.67	2.13
4.69	-11.11	-1.65	-2.51	1.18	39.51	2.6
1.57	-0.87	-2.37	0.37	1.18	86.19	3.36
1.59	-0.91	-2.33	0.36	1.18	85.94	3.36
0.33	-9.69	-1.52	-2.23	1.18	44.2	2.38
-1.27	-8.71	-1.33	-1.99	1.18	47.73	2.21
-1.3	-8.76	-1.14	-2.01	1.18	47.53	2.07
5.2	-9.12	-3.45	-2.07	1.18	46.19	4.79
-1.28	-16.1	-1.45	-3.39	1.19	26.3	2.43
0.22	-3.27	-1.22	-0.48	1.19	72.16	2.11
0.59	-9.17	-0.52	-2.1	1.19	46.02	1.54
4.48	-4.21	-1.62	-0.76	1.19	67.31	2.4
5.64	-9.12	-3.68	-2.07	1.19	46.19	5.46
1.25	-18.73	-2.49	-3.72	1.2	21.08	3.62
7.42	-17.43	-2	-3.54	1.2	23.51	3.03
0.07	-8.14	-0.55	-1.86	1.2	49.81	1.66
-1.55	-15.66	-2.17	-3.32	1.2	27.25	3.17
8.13	-24.62	-1.83	-4.27	1.2	12.6	3.19
0.82	-8.2	-2.22	-1.87	1.2	49.58	3.16
2.14	-1.24	-2.83	0.25	1.2	83.79	4.01
2.2	-1.32	-2.9	0.21	1.2	83.29	4.16

0.48	-8.37	1.36	-1.92	1.2	48.92	0.78
0.13	-7.46	-0.59	-1.68	1.2	52.51	1.58
4.68	-6.79	0.3	-1.5	1.2	55.25	0.83
1.15	-5.42	0.41	-1.13	1.2	61.36	0.78
1.21	-3.63	0.99	-0.57	1.2	70.24	0.81
1.6	-3.18	0.82	-0.42	1.2	72.61	1.02
1.27	-1.52	-0.05	0.13	1.2	82.08	1.32
7.08	-2.11	-3.99	-0.07	1.21	78.55	6.37
1.14	-1.41	-1.11	0.18	1.21	82.72	1.98
-1.5	-15.73	-2.7	-3.33	1.21	27.07	3.64
5.11	-6.68	-3.56	-1.47	1.21	55.7	5.3
0.74	-8.59	1.95	-1.96	1.21	48.05	0.57
2.97	-15.63	-2.79	-3.29	1.21	27.32	4.17
-0.75	-7.71	1.5	-1.73	1.21	51.48	0.66
5.18	-6.82	0.33	-1.51	1.21	55.13	1.11
2.57	1.21	-3.02	1.21	1.21	100	4.16
9.43	-12.15	-2.95	-2.69	1.21	36.26	4.04
1.67	-0.92	-2.11	0.37	1.21	85.7	3.09
1.2	-3.85	0.8	-0.63	1.21	69	0.87
1.19	-3.8	0.96	-0.62	1.21	69.3	0.79
-0.34	-2.91	0.35	-0.33	1.21	74.02	1.09
5.91	-11.08	-2.21	-2.48	1.21	39.52	3.01
-14.9	-33.61	-19.41	-18.56	1.22	22.16	0.9
0.06	-5.91	-3.54	-1.24	1.22	59.06	5.36
4.65	-6.96	-3.59	-1.53	1.22	54.48	5.32
4.9	-5.98	-3.84	-1.27	1.22	58.73	5.81
4.99	-6.15	-3.59	-1.32	1.22	57.99	5.47
0.8	-8.64	0.9	-1.96	1.22	47.83	0.87
-0.58	-8.51	1.11	-1.94	1.22	48.32	0.68
6.12	0.62	-4.22	0.97	1.22	95.72	7.31
3.95	-17.07	-0.52	-3.48	1.22	24.19	1.62
1.83	-5.25	0.02	-1.09	1.22	62.09	1.43
-0.83	-3.83	0.65	-0.64	1.22	69.08	0.97
0.12	-3.38	-0.37	-0.49	1.22	71.43	1.44
1.18	-0.96	2.54	0.36	1.22	85.35	0.34
5.5	-8.76	-3.29	-1.96	1.22	47.39	4.66
0.84	-13.87	-4.39	-3.01	1.23	31.5	7.54
5.46	-14.21	-3.34	-3.05	1.23	30.65	4.79
-0.46	-11.93	-0.92	-2.65	1.23	36.87	2.06
-1.06	-7.61	0.85	-1.69	1.23	51.8	0.82
2.51	-0.58	-4.89	0.51	1.23	87.73	8.4
2.53	-0.6	-4.73	0.5	1.23	87.59	7.94
2.59	-0.65	-4.72	0.47	1.23	87.29	7.92
2.62	-0.67	-4.56	0.47	1.23	87.2	7.58

1.1	-2.08	-3.16	-0.05	1.23	78.56	4.54
1.15	-2.14	-3.43	-0.07	1.23	78.26	4.99
1.17	-2.09	-3.39	-0.05	1.23	78.59	4.74
1	-9.54	-0.83	-2.17	1.23	44.56	1.83
1.18	-3.85	1.02	-0.62	1.23	68.92	0.78
6.3	-13.27	-2.25	-2.88	1.23	33.07	3.2
1.32	-3.6	0.09	-0.54	1.23	70.22	1.24
3.42	-3.46	-0.01	-0.51	1.23	70.99	1.06
1.9	-3.01	1.03	-0.36	1.23	73.36	0.75
0.21	0.36	-0.43	0.88	1.23	93.92	1.5
-1.12	-17.7	-4.75	-6.75	1.24	32.44	0.89
1.64	-3.05	-1.08	-0.37	1.24	73.14	2.03
1.73	-3.13	-0.9	-0.39	1.24	72.67	1.88
0.43	-8.68	0.47	-1.96	1.24	47.63	1.03
2.43	-0.5	-5.16	0.54	1.24	88.18	9.2
2.46	-0.53	-5.22	0.53	1.24	87.99	9.39
2.67	-0.66	-4.35	0.48	1.24	87.13	7.2
1.97	-5.05	-0.65	-1	1.24	62.9	1.42
-1.22	-8.44	-0.49	-1.9	1.24	48.52	1.63
0.86	-1.26	-0.6	0.26	1.24	83.42	1.68
0.89	-1.29	-0.83	0.25	1.24	83.25	1.96
1.11	-10.82	-1.95	-2.42	1.25	40.21	2.79
-0.58	-12.31	-0.92	-2.71	1.25	35.7	1.89
1.73	-2.93	-3.18	-0.32	1.25	73.75	4.4
3.58	-3.47	0.08	-0.49	1.25	70.83	1.17
-0.36	-13.98	-0.64	-3.01	1.25	31.19	1.56
3.17	-5.72	-1.78	-1.16	1.25	59.77	2.77
7.67	-11.69	0.95	-2.57	1.26	37.5	0.67
0.08	-3.38	-1.47	-0.47	1.26	71.22	2.44
4.85	-11.38	-0.17	-2.52	1.26	38.45	1.25
5.43	-4.57	-3.72	-0.83	1.26	65.13	5.71
1.1	-9.32	-0.04	-2.09	1.26	45.24	1.32
-0.63	-12.69	-0.92	-2.77	1.26	34.62	2.05
1.97	-1.32	-1.38	0.24	1.26	82.92	2.41
1.62	-0.8	-2.29	0.45	1.26	86.11	3.23
-0.23	-2.49	-0.58	-0.16	1.26	76.12	1.71
1.34	-3.24	1.45	-0.41	1.26	71.94	0.68
2.4	-7.64	-2.16	-1.68	1.26	51.52	3.33
6.67	-17.93	-4.33	-3.57	1.27	22.44	7.02
1.49	-2.04	-0.82	-0.01	1.27	78.65	1.91
6.91	-24.12	-1.43	-4.19	1.27	13.11	2.72
8.78	-24.72	-1.2	-4.23	1.27	12.42	2.52
2.27	-1.28	-2.52	0.27	1.27	83.12	3.92
3.22	-7.45	-1.56	-1.63	1.27	52.27	2.54

1.67	-0.92	-2.06	0.4	1.27	85.39	3.14
4.03	-2.35	0.58	-0.11	1.27	76.84	0.87
3.7	-6.33	-3.38	-1.32	1.27	56.96	5.17
2.24	-7.62	-2.07	-1.67	1.27	51.61	3.1
14.92	-7.26	15.14	5.64	1.28	22.2	0.8
6.86	-17.36	-4.11	-3.5	1.28	23.52	6.55
7.08	-17.37	-2.58	-3.48	1.28	23.52	4.04
11.11	-2.76	-1.9	-0.25	1.28	74.45	2.57
1.11	-7.74	-2.57	-1.71	1.28	51.07	3.86
-0.12	-5.57	-1.94	-1.12	1.28	60.31	3.02
-1.15	-8.73	-0.14	-1.94	1.28	47.31	1.44
2.62	-6.73	0.09	-1.42	1.28	55.21	1.25
8.97	-12.15	-1.76	-2.65	1.28	36.09	2.5
1.15	-3.94	0.7	-0.62	1.28	68.21	0.95
1.18	-3.79	1.2	-0.57	1.28	68.96	0.72
0.8	0.07	-2.64	0.79	1.28	91.63	3.95
0.8	0.01	-2.48	0.76	1.28	91.24	3.83
-0.36	-3.05	0.76	-0.33	1.28	72.9	0.94
-0.31	-3.02	0.84	-0.33	1.28	73.02	0.84
1.12	-6.6	-2.65	-1.38	1.28	55.77	3.99
3.52	-6.39	-1.75	-1.33	1.28	56.68	2.81
0.03	-1.4	0.67	0.24	1.28	82.35	0.85
-1.51	-15.58	-2.63	-3.25	1.29	27.26	3.86
0.23	-3.39	-0.33	-0.45	1.29	71.01	1.54
1.18	-8.99	-0.01	-2	1.29	46.3	1.36
0.59	-12.03	-1.35	-2.64	1.29	36.42	2.5
0.6	-3.68	0.19	-0.54	1.29	69.49	1.11
0.57	-3.51	0.18	-0.49	1.29	70.37	1.2
-0.35	-2.91	0.67	-0.28	1.29	73.57	0.93
0.45	0.33	0.47	0.89	1.29	93.32	1.05
7.47	-17.08	-1.8	-3.43	1.3	24.04	2.75
-1.48	-15.77	-2.18	-3.28	1.3	26.83	3.19
-0.57	-11.83	1.15	-2.58	1.3	36.99	0.81
0.33	-5.85	-3.68	-1.18	1.3	58.99	5.82
5.29	-7.01	-2.88	-1.5	1.3	53.99	4.8
-0.76	-7.61	0.89	-1.64	1.3	51.53	0.84
2.73	-0.63	-4.47	0.53	1.3	86.98	7.53
2.78	-0.63	-4.25	0.52	1.3	86.95	7.08
1.32	-2.17	-3.15	-0.05	1.3	77.7	4.94
4.97	-11.16	0.05	-2.44	1.3	39.02	1.57
-0.69	-3.85	0.39	-0.59	1.3	68.58	1.15
6.25	-12.73	-1.87	-2.74	1.3	34.38	2.6
6.06	-12.44	-2.35	-2.68	1.3	35.19	3.38
3.81	-17.38	0.85	-2.98	1.31	22.23	1.33

1.49	-1.53	-1.02	0.2	1.31	81.36	2.13
1.6	-1.61	-0.54	0.17	1.31	80.92	1.79
1.24	-3.54	-0.36	-0.48	1.31	70.13	1.63
0.1	-9.37	-1.11	-2.07	1.31	44.9	2.07
0.02	-9.26	-0.2	-2.05	1.31	45.31	1.59
1.39	-10.15	-1.15	-2.25	1.31	42.23	2.16
3.93	-6.25	-3.63	-1.28	1.31	57.16	5.81
-1.02	-6.29	-0.4	-1.31	1.31	56.99	1.78
1.7	-3.08	-0.42	-0.33	1.32	72.5	1.59
1.79	-3.27	-2.18	-0.4	1.32	71.54	3.33
-1.16	-8.5	-0.15	-1.86	1.32	48.04	1.39
-0.97	-6.39	0.42	-1.33	1.32	56.52	1.1
4.67	-17.05	-0.81	-3.41	1.32	24.07	1.94
1.27	-9.95	-0.87	-2.19	1.32	42.88	1.91
1.34	-9.96	-1.03	-2.2	1.32	42.85	2.06
1.16	-3.4	0.35	-0.44	1.32	70.79	1.07
2.71	-2.21	-0.63	-0.03	1.32	77.37	1.48
0.17	-14.32	-0.35	-3.03	1.32	30.2	1.86
0.81	-1.5	-0.57	0.22	1.32	81.48	1.74
0.15	-8.14	-0.6	-1.78	1.33	49.35	1.78
-1.48	-15.72	-2.2	-3.25	1.33	26.87	3.4
15.89	-16.71	-2.59	-3.36	1.33	24.75	3.68
-0.19	-2.47	-0.38	-0.11	1.33	75.81	1.63
-1.35	-12.84	-1.56	-2.75	1.34	33.99	2.88
9.74	-14.21	5.67	-0.23	1.34	22.27	0.97
1.47	-13.68	-4.33	-2.93	1.34	31.75	6.83
1.9	-1.23	-0.98	0.32	1.34	83.03	2.01
-0.07	-2.42	-0.74	-0.1	1.34	76.06	1.86
-0.02	-2.33	-0.75	-0.06	1.34	76.58	1.9
6.59	1.34	-0.32	1.34	1.34	100	1.62
6.75	1.34	-0.54	1.34	1.34	100	1.65
1.43	-6.58	-1.79	-1.35	1.34	55.61	2.78
1.26	-6.9	-1.95	-1.43	1.34	54.25	3.05
1.18	-6.81	-1.65	-1.4	1.34	54.65	2.69
2.52	-7.6	-2.17	-1.62	1.34	51.42	3.4
0.84	-1.39	-0.67	0.27	1.34	82.09	1.76
7.06	-2.15	-3.77	0	1.35	77.52	5.95
0.5	-8.62	0.68	-1.88	1.35	47.44	0.77
0.33	-11.86	-1.48	-2.57	1.35	36.76	2.18
3.37	-7.73	-1.56	-1.65	1.35	50.88	2.68
2.06	-1.29	-1.74	0.31	1.35	82.56	2.92
-0.14	-2.48	-0.54	-0.11	1.35	75.7	1.73
6.79	1.35	-0.77	1.35	1.35	100	1.82
4.22	-6.12	-3.68	-1.22	1.35	57.57	6.32

1.17	-5.68	-1.65	-1.09	1.35	59.51	2.72
1.3	-3.62	-0.58	-0.48	1.35	69.55	1.66
0.68	-10.44	-2.22	-2.29	1.36	41.11	3.52
-1.2	-16.19	-1.91	-3.3	1.36	25.8	3.11
-1.12	-16.24	-1.86	-3.3	1.36	25.68	2.98
1.77	-3.31	-2.07	-0.38	1.36	71.08	2.45
1.25	-7.58	-2.55	-1.62	1.36	51.42	3.93
1.41	-10.36	-0.08	-2.25	1.36	41.38	1.42
4.67	-5.36	-3.63	-1.01	1.36	60.91	5.38
-0.91	-14.78	-2.01	-3.06	1.36	28.98	3.34
-0.88	-13.94	-1.81	-2.93	1.36	31.06	3
-0.96	-6.44	0.7	-1.32	1.36	56.13	1.03
2.66	1.36	-2.88	1.35	1.36	100	4.19
1.32	-6.03	-1.99	-1.18	1.36	57.94	3.14
1.37	-6.55	-2.27	-1.32	1.36	55.65	3.51
4.98	0.67	0.5	1.07	1.36	95.2	1.1
1.42	0.82	1.14	1.13	1.36	96.19	0.83
1.33	0.82	1.2	1.13	1.36	96.25	0.78
-1.07	-16.18	-1.79	-3.29	1.37	25.8	2.95
0.59	-3.67	-0.64	-0.49	1.37	69.15	1.9
0.11	-5.74	-1.9	-1.11	1.37	59.18	3.17
0.21	-6.82	-0.8	-1.41	1.37	54.51	1.87
2.82	-0.59	-4.49	0.58	1.37	86.8	7.85
2.9	1.37	-3.49	1.36	1.37	100	5.87
1.08	-2.04	-2.43	0.05	1.37	78.05	3.62
1.32	-4.38	0.07	-0.71	1.37	65.57	1.45
1.38	-10.49	-0.36	-2.27	1.37	40.94	1.65
0.65	-3.35	0.76	-0.39	1.37	70.83	0.99
-0.33	-3.03	0.65	-0.28	1.37	72.57	0.98
-0.03	-13.06	1.07	-2.54	1.38	32.52	1
1.7	0.78	-0.61	1.13	1.38	95.77	1.85
-1.01	-8.39	0.58	-1.8	1.38	48.23	1.04
1.39	-2.15	-2.94	0.02	1.38	77.36	4.86
0.73	0.93	-0.64	1.2	1.38	96.8	1.83
1.76	-1.19	0.45	0.36	1.38	82.98	1.1
0.56	0.96	-0.39	1.21	1.38	97.03	1.44
1.4	-1.39	-0.96	0.3	1.39	81.72	2.03
1.42	-10.26	-0.11	-2.21	1.39	41.62	1.46
-1.06	-8.46	0.4	-1.81	1.39	47.94	1.17
-1.01	-8.37	0.21	-1.79	1.39	48.26	1.25
0.2	-11.86	-1.31	-2.55	1.39	36.65	2.49
3.19	-16.06	-2.81	-3.25	1.39	26.02	4.42
2.87	-0.61	-4.73	0.58	1.39	86.56	8.64
5.64	-10.88	-1.07	-2.33	1.39	39.63	2.38

0.04	-2.37	-0.57	-0.04	1.39	76.02	1.84
2.19	-3.46	0.38	-0.41	1.39	70.15	1.24
7.21	-8.23	-1.92	-1.73	1.39	48.81	3.24
4.55	0.87	0.59	1.17	1.39	96.32	0.9
4.79	-1.33	-0.72	0.33	1.4	82.05	1.77
1.59	-2.04	-1.03	0.07	1.4	77.86	2.25
6.06	-11.36	0.27	-2.42	1.4	38.11	1
0.24	-8.01	-0.41	-1.69	1.4	49.59	1.66
0.45	-8.89	-0.58	-1.91	1.4	46.34	1.56
6.21	-18.77	-4.06	-3.59	1.4	20.7	7.33
1.57	-1.08	-1.5	0.43	1.4	83.6	2.69
0.91	0.87	-0.92	1.19	1.4	96.26	2.05
0.02	-9.28	-0.65	-2	1.4	44.91	1.87
-0.7	-8.23	-0.34	-1.75	1.4	48.75	1.69
0.71	-1.23	1.61	0.37	1.4	82.66	0.58
0.1	-2.46	-0.39	-0.06	1.4	75.52	1.54
-1.42	-13.96	-0.65	-2.9	1.41	30.88	1.99
6.22	-3.51	-3.31	-0.42	1.41	69.82	4.92
1.83	-3.43	-2.21	-0.39	1.41	70.18	3.57
1.85	-1.22	-0.92	0.36	1.41	82.66	2.03
0.06	-9.26	-0.99	-1.99	1.41	44.97	2.1
-0.74	-8.25	-0.29	-1.75	1.41	48.67	1.59
-0.62	-11.62	-3.69	-2.48	1.42	37.28	5.38
12.88	-22.43	-1.16	-3.94	1.42	15.06	2.53
4.95	-3.8	-3.12	-0.49	1.42	68.22	4.58
-0.7	-13.12	-0.98	-2.74	1.42	33.04	2.08
-0.6	-12.64	-1.21	-2.66	1.42	34.33	2.33
16.81	-16.79	-0.84	-3.31	1.42	24.42	1.93
7.86	-10.5	-3.97	-2.23	1.42	40.76	6.7
1.59	-6.78	-1.86	-1.35	1.42	54.46	3.1
2.68	-7.55	-1.88	-1.56	1.42	51.32	3.2
6.5	-17.09	-3.92	-3.36	1.43	23.81	6.18
-1.05	-16.12	-1.35	-3.24	1.43	25.81	2.6
0.16	-8.64	1.23	-1.83	1.43	47.13	0.81
-0.86	-13.61	-1.62	-2.82	1.43	31.73	2.92
0.25	-11.85	-1.48	-2.52	1.43	36.59	2.59
0.82	0.15	-1.87	0.9	1.43	91.14	3.25
1.33	-6.87	-2.36	-1.36	1.43	54.04	3.73
1.48	-6.91	-2.28	-1.38	1.43	53.9	3.63
7.51	-17.31	-0.9	-3.37	1.44	23.35	2.09
1.65	-3.59	-0.26	-0.43	1.44	69.22	1.72
3.28	-2.53	-0.73	-0.08	1.44	74.9	1.73
-0.87	-13.95	-1.58	-2.88	1.44	30.85	2.74
1.5	-6.94	-1.1	-1.4	1.44	53.73	2.49

2.95	-6.54	-0.18	-1.28	1.44	55.38	1.44
2.72	1.44	-2.96	1.44	1.44	100	4.38
0.98	-2.03	-2.1	0.1	1.44	77.71	3.46
6.6	-8.24	-2.7	-1.71	1.44	48.58	3.76
-0.05	-12.69	1.52	-2.22	1.45	32.6	1.01
-0.61	-7.64	1.55	-1.56	1.45	50.87	0.73
6.2	-6.95	0.09	-1.4	1.45	53.64	1.36
1.33	-10.42	-0.56	-2.21	1.45	40.92	1.79
3.05	-5.12	-0.75	-0.88	1.45	61.63	1.82
4.33	-6.17	-2.35	-1.16	1.45	56.91	3.7
3.89	-6.22	-2.04	-1.18	1.45	56.71	3.16
0.37	0.18	0.56	0.93	1.45	91.26	1.38
1.58	0.89	0.78	1.22	1.45	96.08	1
3.78	-2.76	-4.06	-0.14	1.46	73.53	8.45
1.62	-7.07	-1.56	-1.44	1.46	53.1	2.45
7.15	-10.47	3.76	-0.27	1.46	32.62	1.3
-0.44	-11.75	1.25	-2.46	1.46	36.81	0.82
0.78	-5.77	-3.38	-1.06	1.46	58.63	5.88
1.44	-6.1	-1.77	-1.17	1.46	57.22	2.51
-0.37	-2.93	0.66	-0.19	1.46	72.64	0.98
7.74	-10.71	-3.68	-2.25	1.46	39.97	5.64
7.37	-7.18	7.88	2.61	1.47	32.63	1.46
5.95	-15.78	-3.06	-3.14	1.47	26.48	4.83
1.35	-10.37	0.57	-2.19	1.47	41.04	1.07
-1.04	-8.39	0.55	-1.75	1.47	47.9	1.08
3.42	-17.08	-2.65	-3.33	1.47	23.75	4.49
-0.71	-7.15	0.1	-1.44	1.47	52.73	1.19
2.99	-2.79	-2.38	-0.15	1.47	73.31	3.56
2.15	-1.27	-2.03	0.38	1.47	81.99	3.5
5.4	-11.34	-0.83	-2.37	1.47	37.98	2.01
8.29	-11.7	-1.93	-2.44	1.47	36.9	2.96
1.52	-1.1	-1.29	0.45	1.47	83.01	2.59
3.01	-2.73	-0.59	-0.11	1.47	73.61	1.7
6.83	-3.36	0.01	-0.33	1.47	70.3	1.32
0.05	-2.53	0.14	-0.04	1.47	74.74	1.36
1.06	-2.41	-1.29	-0.01	1.47	75.38	2.75
0.95	-1.17	-0.33	0.42	1.47	82.62	1.66
7.09	-18.02	-3.07	-3.44	1.48	21.94	5.36
6.39	-17.66	-1.06	-3.39	1.48	22.61	2.4
1.46	-10.61	0.57	-2.24	1.48	40.24	1.1
1.95	-3.58	-0.98	-0.39	1.48	69.09	2.29
1.04	0.79	-1.06	1.19	1.48	95.21	2.56
1.56	-11.17	-0.91	-2.34	1.48	38.48	2.14
-0.64	-8.23	-0.23	-1.7	1.48	48.51	1.75

0.1	-2.52	-0.11	-0.04	1.48	74.77	1.46
0.44	-14.25	-0.42	-2.91	1.48	30	1.95
7.16	-18.16	-3.61	-3.46	1.49	21.66	6.6
0.04	-6.82	-1.6	-1.34	1.49	53.99	2.96
1.72	-1.5	-1.12	0.31	1.49	80.56	2.52
10.34	-24.43	-0.74	-4.06	1.49	12.55	2.37
1.9	-3.63	-0.35	-0.41	1.49	68.76	1.74
9.11	-1.01	-3.06	0.49	1.49	83.46	4.6
-1.02	-8.69	0.4	-1.79	1.49	46.74	1.27
0.69	-12.07	-1.05	-2.52	1.49	35.79	2.34
0.79	-11.94	-0.94	-2.49	1.49	36.15	2.16
0.89	-11.99	-0.95	-2.51	1.49	36.03	2.21
1	-2.04	-1.97	0.12	1.49	77.42	3.37
7.56	-11.45	0.04	-2.38	1.49	37.62	1.71
1.71	-5.85	-1.22	-1.05	1.49	58.19	2.5
2.9	-7.49	-1.46	-1.49	1.49	51.28	2.84
2.5	-1.24	-1.89	0.42	1.5	81.97	3.35
-0.76	-13.53	-0.88	-2.77	1.5	31.77	2.12
2.84	-8.09	1.83	-1.65	1.5	48.94	0.48
2.86	1.5	-2.72	1.51	1.5	100	4.48
0.1	-2.37	-0.53	0.02	1.5	75.46	1.88
-0.93	-16.13	-1.46	-3.19	1.51	25.63	2.77
2.36	-0.91	-1.78	0.54	1.51	83.93	3.09
2.43	-1.17	-2.16	0.45	1.51	82.38	3.58
0.87	-8.98	-0.55	-1.86	1.51	45.63	1.97
3.81	-6.87	-1.53	-1.33	1.51	53.74	2.44
-0.89	-6.34	0.96	-1.2	1.51	55.96	0.95
7.55	-11.42	-0.91	-2.36	1.51	37.62	2.15
1.57	-11.36	-0.74	-2.37	1.51	37.83	2.05
0.97	0.1	-1.28	0.93	1.51	90.35	2.72
-0.21	-14.09	-0.84	-2.86	1.51	30.34	1.87
7.25	-8.59	-2.02	-1.74	1.51	47.06	3.49
10.51	-14.58	5.01	-0.57	1.52	22.32	1.06
1.87	-3.5	-0.37	-0.35	1.52	69.27	1.75
0.07	-8.58	0.8	-1.76	1.52	47.03	0.97
2.06	-4.93	0.84	-0.8	1.52	62.21	1.06
6.65	-17.37	-3.44	-3.33	1.53	23.08	5.64
3.05	-0.58	-4.11	0.69	1.53	85.88	7.7
1.06	-1.97	-2.55	0.17	1.53	77.57	4
-0.02	-9.25	-0.27	-1.91	1.53	44.61	1.69
6.27	-12.13	-2.03	-2.48	1.53	35.52	3.2
0.97	-1.22	0.27	0.45	1.53	81.92	1.31
1	-1.15	0.49	0.47	1.53	82.36	1.23
5.68	-14.41	4.97	-0.43	1.54	22.34	1.17

6.99	-17.48	-3.42	-3.34	1.54	22.86	5.65
1.04	-13.95	-3.63	-2.82	1.54	30.62	6.87
1.48	-1.1	-1.14	0.49	1.54	82.64	2.5
1.59	-11.31	-0.64	-2.33	1.54	37.89	2.01
6.62	-7.03	-0.19	-1.35	1.55	52.95	1.7
-0.59	-3.9	0.08	-0.46	1.55	67.11	1.68
0.67	-8.73	2.81	-1.78	1.55	46.4	0.34
0.98	0.95	-0.64	1.3	1.55	95.8	1.84
1.54	-10.98	-0.75	-2.26	1.55	38.88	2.05
1.55	-11.2	-0.86	-2.31	1.55	38.19	2.11
1.9	-1.52	1.03	0.36	1.55	80.02	0.89
4.79	-6.42	-2.67	-1.17	1.55	55.48	4.3
7.15	-8.49	-2.26	-1.69	1.55	47.27	3.54
6.73	-3.71	-3.04	-0.39	1.56	68	5.04
0.25	-2.8	-0.13	-0.09	1.56	72.81	1.65
3.23	-2.67	-0.66	-0.04	1.56	73.5	1.91
-0.03	-9.24	-0.33	-1.89	1.56	44.56	1.64
3.85	-3.23	0.39	-0.22	1.56	70.52	1.12
-1.02	-6.25	-0.52	-1.14	1.56	56.14	2.01
1.49	-0.92	0.19	0.57	1.56	83.61	1.44
1.55	-3.51	-0.5	-0.31	1.56	69.03	1.83
0.47	-8.19	-1.32	-1.63	1.57	48.29	2.68
0.87	-5.66	-0.63	-0.98	1.57	58.71	2.31
2.53	-3.46	-0.38	-0.31	1.57	69.28	1.75
0.4	-2.74	-0.28	-0.07	1.57	73.06	1.72
2.03	-3.62	-2.2	-0.35	1.57	68.45	3.98
0.44	-8.66	-0.06	-1.74	1.57	46.58	1.48
0.99	-12.11	-0.99	-2.48	1.57	35.47	2.31
3.13	-2.2	0.77	0.12	1.57	76.01	0.95
1.72	-5.65	-0.94	-0.95	1.57	58.74	2.29
1.59	-3.51	-0.94	-0.31	1.57	69.03	2.35
-1.29	-12.77	-1.53	-2.58	1.58	33.6	3.04
7.16	-17.66	-3.64	-3.34	1.58	22.44	5.96
0.34	-8.1	-1.46	-1.61	1.58	48.64	2.74
-0.67	-16	-1.43	-3.13	1.58	25.79	2.87
1.5	-10.85	1.04	-2.22	1.58	39.18	0.95
5.4	-4.19	-3.06	-0.53	1.58	65.54	5.23
-0.96	-8.67	-0.17	-1.73	1.58	46.49	1.7
1.39	-1.01	1.05	0.55	1.58	82.9	0.99
6.48	1.58	0.15	1.58	1.58	100	1.32
0.56	-6.01	-2.28	-1.05	1.59	57.05	4.03
0.36	-8.96	1.63	-1.81	1.59	45.47	0.67
2.01	-10.7	-2.29	-2.18	1.59	39.63	3.54
-0.82	-13.88	-1.2	-2.77	1.59	30.69	2.56

-0.79	-13.8	-1.19	-2.76	1.59	30.9	2.48
13.47	-17.27	-1.89	-3.27	1.59	23.18	3.33
7.13	-11.62	-0.33	-2.35	1.59	36.82	1.87
5.3	-6.22	-3.66	-1.1	1.59	56.12	6.7
-0.79	-16.09	-1.64	-3.12	1.6	25.58	3
11.84	-22.41	-0.88	-3.82	1.6	14.89	2.23
1.5	-10.93	0.52	-2.22	1.6	38.89	1.22
14.5	-17.06	-2.62	-3.23	1.6	23.58	4.51
17.33	-17.19	-0.93	-3.25	1.6	23.33	2.08
6.97	-11.16	-0.49	-2.25	1.6	38.17	1.83
7.09	-11.41	-0.67	-2.3	1.6	37.42	2
1.43	-1.02	-0.36	0.57	1.6	82.74	1.81
4.61	-6.01	-3.55	-1.04	1.6	57.02	6.06
4.94	-14.63	5.54	-0.63	1.61	22.35	0.79
3.83	-13.19	-0.79	-2.65	1.61	32.39	2.12
1.49	-10.99	0.31	-2.23	1.61	38.68	1.35
0.99	-5.78	-3.29	-0.97	1.61	57.99	5.89
5.48	-7.49	-3.44	-1.43	1.61	50.87	6.06
5.49	-7.77	-3.57	-1.51	1.61	49.74	6.55
-0.53	-7.61	1.64	-1.45	1.61	50.38	0.8
2.2	-4.85	0.9	-0.73	1.61	62.2	1.06
1.5	-10.94	-0.05	-2.22	1.61	38.8	1.58
1.06	-1.21	0.56	0.49	1.61	81.53	1.29
6.49	-14.2	-2.27	-2.82	1.62	29.84	4.01
-0.17	-11.78	1.62	-2.36	1.62	36.29	0.76
11.22	-23.31	-1.94	-3.88	1.62	13.74	3.28
4.01	-3.08	-2.6	-0.16	1.62	71	4.42
15.89	-10.7	-0.8	-2.17	1.62	39.55	2.19
1.96	-5.08	0.76	-0.79	1.62	61.14	1.1
0.98	0.92	-0.13	1.33	1.62	95.09	1.58
1.51	-11.14	-0.4	-2.25	1.62	38.17	1.79
1.64	-11.31	-0.36	-2.29	1.62	37.7	1.81
0.82	-2.94	0.5	-0.1	1.62	71.69	1.29
1.38	-0.96	0.23	0.6	1.62	83.02	1.36
1.77	1.01	0.43	1.36	1.62	95.71	1.3
1.65	-2.03	0.17	0.21	1.63	76.66	1.56
0.29	-4.88	-0.73	-0.72	1.63	61.98	2.35
0.54	-3.38	-0.43	-0.25	1.63	69.35	1.8
6.18	1.08	-3.89	1.4	1.63	96.14	6.94
6.53	-13.02	-2.26	-2.58	1.63	32.82	3.71
1.41	-1.2	2.38	0.5	1.63	81.48	0.53
1.65	-5.84	-0.84	-0.96	1.63	57.65	2.27
7.5	-8.06	-1.67	-1.54	1.63	48.59	2.92
6.83	-17.35	-0.54	-3.24	1.64	22.93	2.05

-0.27	-12.01	0.06	-2.41	1.64	35.57	1.58
0.14	-2.83	0.34	-0.04	1.64	72.22	1.36
0.79	-2.55	-0.49	0.04	1.64	73.76	1.99
1.65	-3.61	-0.52	-0.3	1.64	68.16	2.01
1.82	-1.36	-1.05	0.46	1.65	80.38	2.59
0.58	-8.25	-1.13	-1.6	1.65	47.81	2.61
4.92	-4.18	-3.21	-0.48	1.65	65.25	5.64
1.13	-12.35	-0.78	-2.47	1.65	34.57	2.34
0.36	-8.23	-0.5	-1.59	1.65	47.9	2.05
3.13	-0.46	-3.9	0.8	1.65	85.91	7.42
7.48	-11.42	0.31	-2.27	1.65	37.25	1.53
1.55	-0.95	-0.69	0.62	1.65	82.87	2.14
8.34	-9.73	-2.95	-1.92	1.65	42.6	4.99
8.15	-10.19	-3.2	-2.02	1.65	41.09	5.72
-0.67	-6.42	-0.57	-1.13	1.65	55.08	2.06
0.16	-2.82	-0.13	-0.04	1.65	72.25	1.72
2.08	-14.02	-3.95	-2.79	1.66	30.18	7.03
6.62	-18.47	-3.46	-3.39	1.66	20.84	6.49
7.07	-18.16	-3.32	-3.35	1.66	21.4	6.14
5.45	-4.42	-2.66	-0.55	1.66	64.02	4.54
2.59	-1.25	-1.65	0.51	1.66	80.99	3.44
1.62	-0.96	-1.71	0.63	1.66	82.7	3.42
0.96	0.98	-0.17	1.38	1.66	95.27	1.63
1.54	-3.49	-0.08	-0.26	1.66	68.63	1.49
6.92	-11.35	-3.01	-2.25	1.66	37.46	4.62
-0.84	-8.66	-0.44	-1.68	1.66	46.3	1.95
1.44	-6.37	-1.33	-1.09	1.66	55.25	2.88
0.84	-1.42	-0.88	0.45	1.66	80.03	2.1
2.42	1.01	0.83	1.39	1.66	95.44	1.09
0.87	-9.61	-0.95	-1.93	1.67	42.96	2.61
2.74	-18.62	-2.78	-3.4	1.67	20.57	4.87
5.29	-3.78	-3.5	-0.34	1.67	67.14	5.8
0.78	-6.19	-2.05	-1.05	1.67	55.93	3.87
0.9	-8.87	0.6	-1.74	1.67	45.51	1.06
2.25	-1.21	-1.49	0.52	1.67	81.16	3.03
5.03	-4.07	-1.34	-0.43	1.67	65.67	2.68
1.55	-1.06	1.14	0.58	1.67	82.07	0.95
1.37	-0.93	1.49	0.64	1.67	82.9	0.81
3.16	-6.72	-1.21	-1.19	1.67	53.77	2.69
1.81	-3.48	-0.6	-0.24	1.67	68.69	2.16
6.89	-17.21	-0.56	-3.2	1.68	23.15	1.76
0.15	-6.8	-1.5	-1.22	1.68	53.37	3.1
9.39	-24.16	-1.4	-3.91	1.68	12.68	3.24
1.55	-7.06	-2.57	-1.28	1.68	52.29	4.72

1.69	-10.94	0.78	-2.17	1.68	38.63	1.13
1.68	-10.91	0.66	-2.17	1.68	38.72	1.18
5.52	-4.38	-3.25	-0.52	1.68	64.17	5.33
0.38	-8.45	0.36	-1.63	1.68	46.97	1.34
16.28	-10.9	-1.39	-2.17	1.68	38.75	2.74
1.72	-3.02	1.74	-0.08	1.68	70.99	0.79
1.36	-0.95	1.17	0.64	1.68	82.71	0.9
1.7	-3.64	-0.31	-0.28	1.68	67.81	1.84
0.85	-10.63	-2.5	-2.13	1.69	39.56	4.43
0.91	-10.8	-2.38	-2.16	1.69	39.05	4.24
1.68	-11.65	3.22	-1.33	1.69	32.67	0.78
1.71	-10.72	0.47	-2.12	1.69	39.29	1.32
2.43	1.02	-0.23	1.41	1.69	95.31	1.79
1.17	-5.85	-3.07	-0.94	1.69	57.32	5.61
14.97	-16.15	-1.61	-3.05	1.69	25.28	2.92
0.29	-2.28	-0.76	0.17	1.69	74.99	2.3
1.42	-4.09	1.03	-0.43	1.69	65.51	1.03
1.43	-3.36	1.72	-0.19	1.69	69.17	0.76
1.43	-3.26	1.18	-0.16	1.69	69.69	0.92
0.74	-3.06	0.78	-0.1	1.69	70.74	1.3
3.88	-9.59	-1.83	-1.88	1.69	42.93	2.94
6.84	-18.19	-3.49	-3.33	1.7	21.28	6.4
0.06	-11.63	1.1	-2.29	1.7	36.51	1.01
0.57	-2.79	0.02	-0.01	1.7	72.15	1.7
5.48	-7.91	-3.56	-1.48	1.7	48.92	6.59
3.68	-14.77	-2.47	-2.85	1.7	28.32	4.74
3.31	-6.55	0.43	-1.12	1.7	54.34	1.23
6.85	-18.13	-4.12	-3.32	1.7	21.4	8.32
1.82	1.07	0.67	1.43	1.7	95.62	1.24
-11.66	-25.53	-15.75	-14.35	1.71	32.75	1.42
1.8	-11.62	3.7	-1.33	1.71	32.8	0.66
-3.79	-18.26	-4.72	-7.39	1.71	32.82	0.69
7.75	-11.63	0.64	-2.27	1.71	36.48	1.1
10.76	-23.56	-2.2	-3.84	1.71	13.35	3.89
9.99	-1.2	-2.32	0.56	1.71	81	4.12
0.95	-9.09	0.21	-1.76	1.71	44.58	1.66
3.34	1.69	-0.38	1.7	1.71	99.88	1.96
4.16	-3.46	0.5	-0.22	1.71	68.56	1.14
0.58	-2.59	0.5	0.08	1.71	73.12	1.3
0.24	-2.76	-0.03	0.03	1.71	72.21	1.66
1.51	-11.69	2.24	-1.41	1.72	32.84	1.26
3.37	-2.63	-0.43	0.07	1.72	72.88	1.86
2.46	1	0.01	1.42	1.72	94.98	1.69
-0.21	-11.79	0.19	-2.31	1.72	36	1.53

1.95	-3.31	0.28	-0.15	1.72	69.31	1.46
-1	-6.11	-0.31	-1.01	1.72	56.08	1.68
0.63	-2.55	0.06	0.09	1.72	73.32	1.59
0.71	-2.65	-0.37	0.07	1.72	72.74	1.9
0.92	-2.44	-0.6	0.14	1.72	73.9	2.1
9.01	1.72	0.22	1.72	1.72	100	1.41
1.77	-3.56	-0.34	-0.24	1.72	67.98	1.86
5.52	-7.92	-3.55	-1.47	1.73	48.78	6.76
-0.31	-12.05	-0.36	-2.35	1.73	35.22	1.86
-0.61	-8.18	-0.12	-1.54	1.73	47.82	1.82
7.38	-7.37	-3.42	-1.31	1.73	50.89	6.34
8.03	-9.92	-3.14	-1.91	1.73	41.73	5.23
-0.67	-6.44	-0.38	-1.09	1.73	54.66	2.1
0.53	-14.45	0.09	-2.78	1.73	28.99	1.57
1.64	-1.06	1.31	0.62	1.73	81.73	0.97
0.17	-2.83	-0.12	0.01	1.73	71.72	1.78
0.95	-2.3	-1.06	0.18	1.73	74.64	2.35
3.4	-7.34	-1	-1.31	1.73	51	2.67
-1.37	-14.45	-0.25	-2.77	1.74	28.96	1.76
5.51	-7.04	-2.89	-1.24	1.74	52.15	5.17
8.13	-7.9	-3.57	-1.44	1.74	48.84	7.12
-0.74	-8.56	-0.43	-1.6	1.74	46.37	2.12
0.86	-1.52	-0.45	0.45	1.74	78.97	1.85
1.51	-3.55	-0.07	-0.22	1.74	67.96	1.65
-0.58	-16.13	-1.16	-3.03	1.75	25.19	2.86
3.7	-2.18	0.28	0.24	1.75	75.14	1.51
4.47	-4.49	-2.98	-0.52	1.75	63.29	5.58
12.94	-17.01	-0.7	-3.13	1.75	23.44	2.38
6.95	-6.8	-3.62	-1.16	1.75	53.1	7.22
1.48	-6.8	-1.71	-1.15	1.75	53.1	3.43
0.08	0.86	0.18	1.38	1.75	93.83	1.32
2.25	1.04	1.15	1.45	1.75	94.99	1.09
0.88	-3.16	-0.23	-0.09	1.75	69.93	1.88
6.58	1.75	1.92	1.75	1.75	100	0.72
6.58	-17.47	0.66	-3.11	1.76	22.37	1.34
4.15	-18.81	-3.44	-3.36	1.76	20.11	6.48
0.19	-6.74	-1.6	-1.15	1.76	53.3	3.3
-4.07	-17.99	-6.25	-7.17	1.76	32.91	1.47
0.74	-5.26	-1.33	-0.75	1.76	59.68	2.56
1.73	-3.84	-0.47	-0.31	1.76	66.41	2.02
1.81	-10.65	0.54	-2.06	1.76	39.3	1.35
0.39	-8.29	-0.17	-1.54	1.76	47.28	1.73
12.19	-17.22	-1.9	-3.15	1.76	23.01	3.82
0.45	-2.67	0.87	0.08	1.76	72.47	1.09

2.52	-12.06	1.2	-1.75	1.77	32.94	1.38
0.54	-5.14	-1.45	-0.71	1.77	60.19	3.72
5.44	-6.72	-2.86	-1.13	1.77	53.36	5.32
5.42	-6.83	-2.92	-1.17	1.77	52.87	5.38
5.48	-6.79	-2.64	-1.15	1.77	53.05	4.87
11.5	-12.5	-0.43	-2.41	1.77	33.89	1.94
10.76	-17.27	-1.61	-3.15	1.77	22.88	3.55
2.36	-1.19	-2.07	0.58	1.77	80.74	4.03
1.5	-3.88	0.98	-0.32	1.77	66.14	1.11
1.79	-10.76	-0.02	-2.08	1.77	38.91	1.71
2.09	1.08	0.82	1.47	1.77	95.19	1.11
1.95	1.13	1	1.5	1.77	95.47	1.03
5.59	-8	-3.49	-1.46	1.78	48.3	6.75
7.05	-17.3	-0.66	-3.15	1.78	22.82	2.24
3.37	1.78	0.04	1.78	1.78	99.96	1.61
-0.2	-4.19	1.09	-0.41	1.78	64.58	1.08
1.77	-10.77	0.01	-2.07	1.78	38.88	1.67
6.45	-13.15	-1.75	-2.51	1.78	32.14	3.65
0.29	-2.73	0.35	0.07	1.78	72.01	1.42
0.38	-2.65	1.02	0.1	1.78	72.42	1.03
12.88	-21.52	-1.45	-3.61	1.79	15.88	2.56
1.54	-5.98	-3.55	-0.92	1.79	56.38	7.11
9.27	-17.26	-1.98	-3.14	1.79	22.89	3.83
1.69	-0.92	-1.96	0.71	1.79	82.24	3.91
3.38	1.75	-0.34	1.77	1.79	99.71	2.03
1.88	-4.87	-0.8	-0.61	1.79	61.34	2.52
1.68	-6.38	-0.72	-1.04	1.79	54.7	2.34
1.89	-3.72	1.03	-0.24	1.79	66.86	1.02
1.23	0.26	-0.88	1.16	1.79	89.6	2.64
2.31	-10.78	-0.25	-2.08	1.79	38.82	1.64
0.19	-2.78	-0.1	0.06	1.79	71.7	1.73
1.25	-1.45	-0.42	0.52	1.79	79.07	1.99
7.09	-6.86	-0.33	-1.15	1.8	52.65	1.82
-0.04	-11.94	0.15	-2.29	1.8	35.35	1.69
8.57	-17.37	-0.56	-3.15	1.8	22.64	2.22
-0.38	-3.73	0.86	-0.25	1.8	66.75	1.25
2.01	-3.25	0.29	-0.08	1.8	69.19	1.5
1.47	-0.97	2.06	0.69	1.8	81.91	0.61
1.13	-2.34	-0.32	0.21	1.8	74.05	2.21
1.07	-1.59	-0.38	0.48	1.8	78.22	2.04
0.99	-1.6	-0.31	0.46	1.8	78.16	1.98
1.89	-1.3	-1.01	0.58	1.81	79.82	2.77
4.43	-13.14	-1.01	-2.52	1.81	32.08	2.47
0.34	-11.41	-3.12	-2.19	1.81	36.85	5.34

3.52	-2.28	0.18	0.23	1.81	74.3	1.72
1.47	-8.85	1.39	-1.65	1.81	45.12	0.96
1.51	-8.11	1.27	-1.46	1.81	47.81	1.05
-0.14	-12.03	-0.03	-2.3	1.81	35.08	1.77
2.04	-5.67	-0.74	-0.83	1.81	57.66	2.14
0.37	-8.2	-0.29	-1.48	1.81	47.46	1.94
1.83	-3.66	1.69	-0.21	1.81	67.03	0.76
6.56	-12.73	-2.29	-2.42	1.81	33.17	3.87
7.83	-7.24	-3.28	-1.23	1.81	51.1	6.58
5.92	-7.21	-2.56	-1.21	1.81	51.25	4.75
0.85	-2.43	-0.45	0.19	1.81	73.5	2.01
0.94	-3.25	0.45	-0.09	1.81	69.19	1.51
7.18	-17.65	-2.49	-3.18	1.82	22.09	4.8
5.45	-4.47	-2.25	-0.46	1.82	63.05	4.05
0.47	-2.3	0.08	0.24	1.82	74.14	1.65
0.54	-2.33	0.04	0.23	1.82	74	1.72
3.53	-2.08	1.49	0.31	1.82	75.35	0.79
1.73	-0.89	2.6	0.73	1.82	82.2	0.44
6.61	1.82	2.22	1.82	1.82	100	0.63
1.36	-8.95	-0.3	-1.68	1.83	44.69	1.87
4.66	-18.76	-3.36	-3.31	1.83	20.09	6.46
5.09	-18.66	-3.29	-3.3	1.83	20.27	6.32
5.44	-18.52	-3.16	-3.29	1.83	20.52	6.22
5.85	-18.49	-3.5	-3.28	1.83	20.55	6.71
0.2	-4.43	-0.57	-0.46	1.83	63.25	2.05
0.39	-4.83	-0.62	-0.58	1.83	61.38	2.5
1.83	-10.24	0.99	-1.93	1.83	40.38	1.14
0.95	-8.82	0.71	-1.62	1.83	45.16	1.15
6.8	-11.71	-0.02	-2.21	1.83	35.95	1.85
0.24	-14.55	-0.03	-2.73	1.83	28.54	1.57
2.41	1.2	1.32	1.57	1.83	95.58	0.91
0.48	-11.7	1.94	-2.21	1.84	35.93	0.78
1.83	-10.43	0.82	-1.97	1.84	39.79	1.2
4.62	-4.3	-2.88	-0.39	1.84	63.78	5.15
1.72	-0.89	-2.1	0.75	1.84	82.14	4.25
3.64	1.51	0.24	1.7	1.84	97.6	1.6
0.68	1.13	0.29	1.55	1.84	95.04	1.52
1.2	0.4	-1.18	1.24	1.84	90.2	2.78
1.1	-10.84	-2.15	-2.07	1.85	38.47	4.33
7.82	-16.39	-0.45	-2.98	1.85	24.49	2.07
3.78	0.89	-3.36	1.45	1.85	93.32	6.35
3.36	1.85	0.28	1.85	1.85	100	1.51
1.53	-4.16	1.04	-0.36	1.85	64.43	1.21
1.8	-3.56	1.82	-0.15	1.85	67.35	0.73

1.08	-1.31	0.8	0.61	1.85	79.55	1.28
-15.69	-33.86	-20.76	-18.92	1.86	22.38	1.25
-4.75	-21.96	-5.7	-7.26	1.86	22.45	1.48
4.79	-2.3	-0.51	0.25	1.86	73.93	2.16
7.63	-23.95	-0.5	-3.78	1.86	12.75	1.84
1.69	-6.8	-2.99	-1.11	1.86	52.72	5.86
0.77	-2.77	0.09	0.1	1.86	71.39	1.71
2.46	1.12	0.34	1.55	1.86	94.82	1.52
5.42	-6.58	-2.94	-1.05	1.86	53.6	5.58
5.67	-7.72	-3.12	-1.34	1.86	49.09	6.25
5.44	-5.31	-2.18	-0.69	1.86	59.05	4.17
5.56	-5.6	-2.09	-0.78	1.86	57.75	4.11
5.67	-6.23	-2.35	-0.94	1.86	55.03	4.41
9.97	-16.86	-1.88	-3.04	1.86	23.54	3.66
2.38	-4.8	1.31	-0.56	1.86	61.36	0.96
2.18	-3.61	0.49	-0.17	1.86	67.1	1.43
2.69	-4.82	0.51	-0.56	1.87	61.19	1.54
1.81	-6.3	-3.03	-0.96	1.87	54.69	5.91
0.9	-2.69	-0.17	0.12	1.87	71.78	1.94
2.55	-4.77	1.29	-0.54	1.87	61.44	0.97
1.69	-5.99	-0.38	-0.88	1.87	56	1.99
1.66	-6.65	-0.95	-1.06	1.87	53.24	2.61
1.03	-3.11	0.78	0	1.87	69.57	1.17
6.13	-13.38	-1.52	-2.49	1.87	31.33	3.61
6.39	-12.96	-1.87	-2.42	1.87	32.42	3.77
8.95	-9.25	-1.9	-1.67	1.87	43.55	3.92
8.48	-8.59	-1.05	-1.52	1.87	45.86	2.67
6.59	1.87	2.03	1.87	1.87	100	0.6
2.34	1.05	-0.41	1.52	1.88	94.21	2.12
6.62	1.36	-3.51	1.66	1.88	96.33	7.44
15.36	-15.56	-1.95	-2.84	1.88	26.19	3.17
4.96	-3.42	-2.74	-0.11	1.88	67.96	5
1	-3.09	-0.46	0.01	1.88	69.63	2.06
0.88	-3.17	-0.19	-0.03	1.88	69.21	1.92
0.81	-3.19	-0.29	-0.02	1.88	69.1	2.03
0.93	1.24	0.17	1.61	1.88	95.51	1.43
0.83	-8.35	-0.74	-1.47	1.89	46.63	2.56
6.98	-15.75	-3.07	-2.87	1.89	25.76	6.12
7.75	-11.28	1.37	-2.09	1.89	37.01	0.92
1.96	-5.75	-2.46	-0.81	1.89	56.97	4.95
0.63	-3.41	-0.7	-0.1	1.89	67.97	2.12
2.34	1.01	-0.64	1.52	1.89	93.87	2.35
2.46	0.96	-0.41	1.5	1.89	93.5	2.12
2.59	0.9	-0.61	1.47	1.89	93.17	2.3

1.56	-8.66	0.9	-1.55	1.89	45.52	1.24
5.86	-6.72	-2.24	-1.05	1.89	52.9	4.59
2.08	-6.24	2.99	-0.94	1.89	54.87	0.47
3.48	-2.59	-0.36	0.18	1.89	72.2	1.82
1.92	-4.55	-1.25	-0.45	1.89	62.4	2.92
-0.64	-8.19	0.2	-1.43	1.89	47.24	1.69
4.28	-2.76	-0.47	0.13	1.89	71.29	1.95
0.9	-1.53	0.15	0.54	1.89	78.05	1.55
7.27	-17.74	-2.9	-3.14	1.9	21.79	5.63
7.24	-3.77	-2.94	-0.21	1.9	66.11	5.36
1.27	-12.55	-0.74	-2.35	1.9	33.43	2.69
0.63	-2.44	-0.11	0.24	1.9	72.95	1.96
3.43	1.78	-1	1.84	1.9	99.19	2.74
6.73	-12.01	-0.57	-2.22	1.9	34.91	2.29
-0.87	-12.8	-1.2	-2.38	1.91	32.73	3.11
6.2	-18.29	-2.96	-3.2	1.91	20.8	5.61
6.44	-18.27	-2.68	-3.2	1.91	20.83	5.28
6.94	-18.15	-3.09	-3.19	1.91	21.05	5.87
0.3	-6.71	-0.76	-1.05	1.91	52.87	2.5
7.6	-11.06	0.78	-2.03	1.91	37.62	1.12
1.83	-10.41	1.04	-1.91	1.91	39.65	1.12
2.69	0.85	0.2	1.47	1.91	92.64	1.69
1.33	-8.67	1.24	-1.54	1.91	45.43	1.02
1.75	-0.73	-1.27	0.85	1.91	82.69	3.03
1.76	-0.54	-0.98	0.93	1.91	83.82	2.73
1.77	-0.62	-1.25	0.9	1.91	83.33	3.06
1.8	-0.62	-1.18	0.9	1.91	83.3	3.05
0.6	-2.38	-0.52	0.27	1.91	73.23	2.27
0.7	-2.46	-0.52	0.24	1.91	72.81	2.34
-0.97	-12.81	-1.26	-2.37	1.92	32.7	3.07
-11.69	-25.4	-15.42	-14.3	1.92	33.01	1.28
1.54	-6.41	0.63	-0.97	1.92	54.04	2.76
1.84	-10.42	1.06	-1.91	1.92	39.56	1.11
4.66	-4.34	-2.67	-0.36	1.92	63.24	5.02
1.86	-6.21	-1.76	-0.92	1.92	54.87	4.11
1.23	-7.22	0.76	-1.18	1.92	50.78	1.28
1.19	-7.16	0.65	-1.16	1.92	51.04	1.37
1.74	-6.55	-0.59	-1	1.92	53.47	2.21
4.85	-10.46	-0.23	-1.92	1.92	39.47	1.66
8.28	-19.83	-3.64	-3.37	1.92	18.21	7.87
1.11	0.42	-1.34	1.3	1.92	89.81	3.23
-0.64	-8.5	-0.94	-1.48	1.92	46.02	2.7
6.61	1.92	2.07	1.92	1.92	100	0.65
1.58	-11.01	0.85	-2.02	1.93	37.74	1.25

5.44	-6.35	-2.15	-0.94	1.93	54.27	4.35
7.54	-19.23	-3.46	-3.3	1.93	19.17	6.99
6.52	-11.67	-0.16	-2.14	1.93	35.79	1.88
3.42	1.86	-0.21	1.9	1.93	99.49	1.99
1.82	-10.61	0.46	-1.95	1.93	38.96	1.51
1.79	-3.33	1.4	-0.03	1.93	68.14	0.92
3.68	1.56	0.66	1.77	1.93	97.4	1.41
9.52	1.93	0.37	1.93	1.93	100	1.45
1.55	-11.15	0.77	-2.05	1.94	37.29	1.33
1.63	-10.97	0.85	-2.01	1.94	37.83	1.25
0.67	-8.69	1.49	-1.53	1.94	45.26	0.95
3.34	0	-3.55	1.15	1.94	86.97	7.27
3.42	0.01	-3.63	1.15	1.94	87.02	7.53
3.37	1.94	-0.08	1.94	1.94	100	1.85
3.62	1.64	0.3	1.81	1.94	97.92	1.56
1.63	-7	-0.18	-1.11	1.94	51.61	1.79
1.8	-10.7	0.42	-1.96	1.94	38.67	1.56
6.48	-12.42	-1.1	-2.27	1.94	33.7	2.51
1.86	-1.87	0.59	0.45	1.94	75.88	1.41
9.2	-9.56	-1.51	-1.69	1.94	42.29	3.43
6.58	-13.74	-2.03	-2.52	1.95	30.24	3.77
10.84	-14.03	5.79	-0.17	1.95	22.49	0.74
1.29	-14.07	-3.52	-2.58	1.95	29.44	7.66
0.89	-2.61	-0.17	0.2	1.95	71.83	2
3.49	-2.07	-0.1	0.39	1.95	74.73	1.87
1.34	-3.06	0	0.05	1.95	69.45	1.88
1.33	-3.08	0.24	0.05	1.95	69.37	1.76
0.84	1.2	0.47	1.64	1.95	94.77	1.51
1.87	-5.09	-0.26	-0.58	1.95	59.66	2.2
2.47	-3.32	1	-0.02	1.95	68.09	1.16
7.26	-2.97	0.16	0.09	1.95	69.89	1.45
9.31	-9.01	-1.01	-1.57	1.95	44.12	2.88
3.51	-1.97	-0.35	0.43	1.96	75.2	2.08
1.85	-9.04	0.54	-1.61	1.96	43.97	1.42
10.76	-1.1	-2.2	0.74	1.96	80.19	4.32
1.04	-3.07	-0.05	0.06	1.96	69.34	1.77
3.37	1.96	-0.74	1.96	1.96	100	2.53
0.46	0.75	0.23	1.45	1.96	91.7	1.65
2.61	-11.88	-0.7	-2.19	1.96	35.13	2.17
1.1	-1.37	1.45	0.65	1.96	78.62	1.02
1.34	-1.5	-0.23	0.6	1.96	77.87	1.87
2.08	-1.84	-0.13	0.48	1.97	75.92	2.03
0	-4.21	0.41	-0.3	1.97	63.65	1.68
2.18	-3.86	-0.22	-0.19	1.97	65.34	2.14

3.87	0.81	-3.18	1.48	1.97	92.01	6.28
0.63	-3.16	-0.2	0.03	1.97	68.79	2.07
3.46	1.84	-0.61	1.92	1.97	99.08	2.34
3.68	-5.1	-0.44	-0.55	1.97	59.5	2.04
0.47	-4.27	-0.26	-0.32	1.97	63.34	2.19
0.81	-3.16	0.35	0.04	1.97	68.82	1.66
4.87	-17.36	0.69	-3.03	1.98	22.38	1.6
0.43	-6.67	-0.2	-1	1.98	52.77	2.07
6.69	-15	-3.28	-2.69	1.98	27.21	5.72
2.67	-1.11	-1.58	0.75	1.98	80	3.66
1.35	-12.95	-0.54	-2.37	1.98	32.19	2.7
6.36	-17.19	0.24	-3	1.98	22.69	1.52
3.5	0.07	-3.15	1.2	1.98	87.15	6.57
4.9	-8.31	-1	-1.39	1.98	46.5	2.96
6.03	1.98	1.23	1.98	1.98	100	1.07
1.12	-7.53	1.05	-1.21	1.99	49.35	1.08
0.62	-2.38	-0.31	0.32	1.99	72.78	2.11
1.47	-11.31	0.5	-2.05	1.99	36.67	1.53
6.57	-6.45	-3.29	-0.91	1.99	53.6	6.79
-0.68	-6.41	-0.63	-0.93	1.99	53.77	2.41
6.31	-6.7	-2.83	-0.97	1.99	52.63	5.35
3.98	-8.09	-0.87	-1.33	1.99	47.26	2.71
2.41	1.3	1.77	1.69	1.99	95.18	0.85
2.42	1.37	2.21	1.72	1.99	95.66	0.66
-0.3	-13.45	-1.13	-2.49	2	30.85	2.97
2.68	-14.11	-4	-2.59	2	29.27	7.87
12.28	-2.58	-0.86	0.24	2	71.7	2.3
1.46	-11.74	0.57	-2.13	2	35.43	1.49
0.88	-2.8	0.43	0.17	2	70.54	1.54
1.78	-10.79	0.99	-1.94	2	38.23	1.2
1.73	-10.78	1.22	-1.93	2	38.23	1.06
5.74	-7.88	-2.99	-1.29	2	48.01	6.32
1.08	-5.5	2.32	-0.67	2	57.58	0.7
3.79	-2.85	-0.26	0.16	2	70.26	2
1.18	-3.11	1.1	0.07	2	68.91	1.21
1.19	-3.05	1.07	0.09	2	69.24	1.18
6.05	-6.19	-2.65	-0.84	2	54.69	5.48
1.19	-10.91	-1.87	-1.98	2.01	37.81	4.3
0.6	-4.85	-1.23	-0.47	2.01	60.49	3.07
9.93	-23.79	-0.92	-3.67	2.01	12.79	2.47
1.61	-5.99	-1.77	-0.78	2.01	55.49	4.02
5.41	-6.33	-2.19	-0.88	2.01	54.03	4.57
5.4	-6.16	-2.32	-0.84	2.01	54.75	4.75
2.51	-1.3	-2.23	0.69	2.01	78.71	5.21

5.79	-4.05	-0.72	-0.21	2.01	64.18	2.41
2.16	-3.83	1.14	-0.15	2.01	65.3	1.15
5.75	-0.99	1.25	0.81	2.01	80.53	0.97
1.66	-0.89	3.45	0.84	2.01	81.11	0.33
0.83	-3.17	0.03	0.06	2.01	68.58	1.67
-0.73	-12.84	-1.14	-2.31	2.02	32.38	3.22
17.75	-6.4	15.9	6.24	2.02	22.53	0.86
2.11	-5.49	-2.26	-0.65	2.02	57.59	4.84
2.21	-5.94	-3.06	-0.76	2.02	55.66	6.52
5.43	-4.71	-2.35	-0.41	2.02	61.08	4.53
3.92	-16.08	-2.45	-2.84	2.02	24.82	5.27
3.53	0.13	-3.23	1.24	2.02	87.32	6.95
3.95	0.7	-3.05	1.47	2.02	91.01	6.2
1.26	-2.97	0.1	0.13	2.02	69.52	1.75
0.76	-2.45	-0.57	0.31	2.02	72.29	2.46
6.51	-12.93	-1.35	-2.32	2.02	32.14	3.11
0.64	-2.5	0.83	0.28	2.02	72.04	1.34
2.36	1.3	1.06	1.71	2.02	95.01	1.13
4.62	-15.02	5.08	-1.06	2.03	22.57	0.87
1.5	-11.19	0.66	-2	2.03	36.94	1.42
-0.36	-7.94	0.99	-1.29	2.03	47.67	1.2
3.72	1.61	0.06	1.85	2.03	97.09	1.94
1.46	-11.48	0.43	-2.06	2.03	36.1	1.58
0.71	-3.1	1.01	0.09	2.03	68.83	1.25
0.64	-3.06	0.77	0.11	2.03	69.02	1.38
8.16	-7.67	-3.44	-1.2	2.03	48.69	6.82
1.23	-2.24	0.64	0.39	2.03	73.39	1.68
4.26	-8.24	-1.17	-1.35	2.03	46.56	3.14
2.19	-6.87	-0.5	-1.03	2.04	51.75	2.09
0.95	-8.4	0.01	-1.4	2.04	45.98	1.97
-0.33	-7.91	0.67	-1.27	2.04	47.73	1.3
7.07	-10.97	0.05	-1.93	2.04	37.57	1.56
1.35	-3	-0.27	0.13	2.04	69.28	2.08
-0.25	-3.9	0.61	-0.16	2.04	64.8	1.4
1.45	-11.81	0.3	-2.11	2.04	35.14	1.69
0.97	-3.26	0.46	0.05	2.04	67.97	1.45
0.52	-4.2	0.25	-0.26	2.04	63.38	2.14
9.1	-9.19	-1.5	-1.55	2.04	43.22	3.34
2.67	-3.39	0.45	0.02	2.04	67.31	1.63
16.31	-6.6	15.49	6.05	2.05	22.6	0.98
1.99	-1.14	-0.59	0.78	2.05	79.41	2.46
0.05	-11.76	0.03	-2.09	2.05	35.23	2.07
1.31	-2.97	-0.23	0.14	2.05	69.39	1.99
3.38	2.05	-0.59	2.05	2.05	100	2.45

3.37	2.05	-0.62	2.05	2.05	100	2.45
1.42	-11.83	0.03	-2.11	2.05	35.05	1.93
1.42	-11.87	0.02	-2.12	2.05	34.96	1.96
5.73	-13.24	0.3	-2.35	2.05	31.29	1.82
-0.4	-8.16	0.27	-1.33	2.05	46.8	1.72
0.78	-14.47	0.29	-2.58	2.05	28.29	1.78
4.78	-7.78	-1	-1.22	2.05	48.2	2.92
0.82	-3.12	0.02	0.1	2.05	68.61	1.89
17.07	-6.33	15.79	6.27	2.06	22.63	0.83
5.27	-4.06	-1.57	-0.2	2.06	63.94	3.15
-0.12	-13.61	-0.64	-2.48	2.06	30.34	2.88
-0.39	-16.22	-1	-2.85	2.06	24.48	3.19
7.32	-15.72	-3.16	-2.75	2.06	25.5	6.28
2.75	-1.12	-1.06	0.79	2.06	79.44	3.12
1.13	-5.6	2.55	-0.66	2.06	56.94	0.68
3.5	1.92	-0.84	2	2.06	99.05	2.74
3.49	1.92	-0.57	2	2.06	99.02	2.38
1.32	1.24	-0.46	1.71	2.06	94.32	2.5
-0.43	-8.14	0.13	-1.31	2.06	46.83	1.83
-0.4	-8.11	0.37	-1.31	2.06	46.98	1.65
6.66	-6.6	-2.79	-0.91	2.06	52.73	5.71
2.02	-3.66	-0.56	-0.06	2.06	65.89	2.66
11.35	-14.04	5.42	-0.23	2.07	22.64	0.95
5.6	-4.19	-1.89	-0.24	2.07	63.27	4.06
7.7	-4.24	-2.8	-0.25	2.07	63.05	5.81
8.03	-7.03	-0.44	-1.03	2.07	50.98	2.35
1.85	-5.79	-1.8	-0.69	2.07	56.04	4.09
2.14	-5.51	-1.74	-0.61	2.07	57.26	4.11
4.83	-4.88	-2.36	-0.43	2.07	60.08	5.12
0.51	-8.61	1.95	-1.43	2.07	45.14	0.86
1.83	-0.5	-0.99	1.04	2.07	83.07	3.01
0.3	-3.04	-0.71	0.14	2.07	68.92	2.63
4.57	-2.26	1.58	0.39	2.07	73.07	0.85
1.23	-0.96	1.66	0.86	2.07	80.36	0.88
0.17	-3.28	-0.44	0.06	2.08	67.65	2.44
3.73	-1.99	1.58	0.49	2.08	74.45	0.86
6.83	-13.29	-0.86	-2.33	2.08	31.08	2.67
-0.31	-8.53	-1.03	-1.39	2.08	45.37	3.14
1.21	-0.81	2.32	0.92	2.08	81.22	0.65
0.7	-5.17	-1.42	-0.52	2.09	58.67	3.57
3.88	-5.11	-2.62	-0.49	2.09	58.98	5.56
4.54	-4.21	-2.69	-0.22	2.09	63.11	5.53
5.39	-6.33	-1.68	-0.84	2.09	53.76	3.89
5.38	-6.23	-2.02	-0.81	2.09	54.13	4.37

1.16	-3.11	0.88	0.13	2.09	68.46	1.37
1.97	-6.19	-1.58	-0.78	2.09	54.33	3.83
-11.84	-25.47	-14.69	-14.37	2.1	33.03	1.03
-3.17	-17.5	-4.44	-6.74	2.1	33.06	0.87
3.12	-5.67	-3.18	-0.64	2.1	56.47	7.11
1.32	-6.4	-0.17	-0.85	2.1	53.42	2.36
5.82	-3.72	-2.99	-0.07	2.1	65.4	6.01
1.79	-5.33	0.43	-0.55	2.1	57.94	1.53
0.8	-3.18	1.16	0.11	2.1	68.07	1.11
6.05	-13.35	-1.16	-2.34	2.1	30.9	3.11
0.7	-2.58	1.07	0.3	2.1	71.17	1.37
2.13	-1.23	0.28	0.78	2.11	78.57	1.9
0.84	-2.55	0.42	0.32	2.11	71.31	1.68
5.8	-7.85	-3.11	-1.22	2.11	47.74	6.77
6.81	1.52	-2.92	1.86	2.11	95.89	6.43
2.06	-4.19	0.08	-0.2	2.11	63.06	1.97
1.98	-4.56	-0.67	-0.31	2.11	61.35	2.42
0.67	-3.24	0.94	0.1	2.11	67.74	1.29
0.64	-3.23	1.01	0.1	2.11	67.77	1.21
0.64	-3.13	1.21	0.13	2.11	68.27	1.14
1.69	-12.04	0.28	-2.1	2.11	34.33	1.94
5.98	-12.98	-1.1	-2.28	2.11	31.81	3.1
6.25	-13.08	-1.14	-2.29	2.11	31.57	3.19
6.37	-13.11	-1.44	-2.3	2.11	31.48	3.31
7.58	-2.7	-0.46	0.28	2.12	70.46	2.06
2.23	-3.88	-0.29	-0.11	2.12	64.54	2.3
2.66	-5.99	-2.98	-0.72	2.12	55.05	6.74
1.39	-2.99	0.22	0.18	2.12	68.96	1.76
-0.16	-8.03	0.26	-1.26	2.12	47.04	1.79
7.79	-9.06	-0.89	-1.47	2.12	43.41	3.05
9.41	-9.01	-1.13	-1.46	2.12	43.59	3.21
0.5	-6.62	0.5	-0.89	2.13	52.37	1.58
0.58	-6.63	0.53	-0.89	2.13	52.34	1.61
2.33	-1.74	-0.15	0.61	2.13	75.57	2.2
2.74	-2.91	-0.57	0.21	2.13	69.27	2.18
1.52	-9.66	0.56	-1.62	2.13	41.37	1.7
1.68	-6.04	2.37	-0.75	2.13	54.8	0.7
1.87	-0.47	-0.42	1.08	2.13	82.94	2.46
1.34	-2.97	-0.32	0.2	2.13	68.99	2.19
5.9	2.01	0.85	2.08	2.13	99.21	1.87
1.46	-11.97	0	-2.09	2.13	34.45	2.04
6.99	-13.38	-0.72	-2.31	2.13	30.75	2.68
-0.39	-7.65	0.29	-1.16	2.13	48.41	1.81
-0.11	-8.11	0.12	-1.27	2.13	46.73	2.01

2.39	-5.46	-1.47	-0.55	2.14	57.21	3.84
5.07	-4.54	-1.79	-0.29	2.14	61.33	4.14
1.09	-3.06	0.36	0.17	2.14	68.47	1.6
0.48	-3.04	-0.35	0.18	2.14	68.58	2.45
-0.26	-8.16	0.65	-1.27	2.14	46.52	1.54
0.49	-7.19	-0.24	-1.03	2.14	50.15	2.39
0.46	-4.36	-0.62	-0.25	2.14	62.17	2.42
8.77	-9.33	-1.37	-1.52	2.14	42.44	3.69
-2.98	-15.06	-2.71	-4.55	2.15	33.1	1.46
4.36	-4.76	-2.26	-0.35	2.15	60.29	5.22
0.2	-3.23	-0.49	0.12	2.15	67.56	2.6
3.39	2.15	-1.18	2.15	2.15	100	3.27
3.37	2.15	-1.01	2.15	2.15	100	2.95
0.82	-3.07	0.6	0.18	2.15	68.39	1.49
8.14	-8.17	-3.02	-1.25	2.15	46.44	6.13
7.56	-15.87	-3.39	-2.71	2.16	25.01	7.17
4.1	-4.86	-2.6	-0.37	2.16	59.76	5.59
2.85	-0.76	-0.54	0.99	2.16	81.02	2.67
5.39	-6.11	-1.29	-0.73	2.16	54.37	3.44
1.92	-0.6	-1.11	1.05	2.16	81.96	3.46
0.92	-2.44	-0.63	0.39	2.16	71.6	2.63
1.41	-12.14	0.64	-2.09	2.16	33.91	1.54
6.14	-6.21	-2.62	-0.75	2.16	53.95	5.41
2.73	-3.56	-0.43	0.03	2.16	65.92	2.53
0.49	-4.57	-1.67	-0.29	2.17	61.06	4.03
6.05	-6.33	-1.35	-0.78	2.17	53.43	3.7
1.57	-5.95	1.17	-0.69	2.17	55	1.12
1.92	-5.76	3.23	-0.64	2.17	55.77	0.46
0.56	-2.48	1.26	0.38	2.17	71.36	1.15
2.87	0.9	1.19	1.64	2.17	91.31	1.21
3.83	1.63	1.17	1.93	2.17	96.2	1.29
8.2	-9.72	11.38	3.44	2.18	22.67	1.11
-0.06	-7.52	2.4	-1.07	2.18	48.71	0.67
0.87	-6.4	-1.31	-0.8	2.18	53.09	3.56
0.7	-6.48	-1.4	-0.82	2.18	52.75	3.76
1.16	-3.02	1.26	0.21	2.18	68.53	1.01
0.62	-2.45	0.82	0.4	2.18	71.47	1.44
3.7	-1.73	0.65	0.64	2.18	75.36	1.5
4.5	-5.33	-3.75	-0.49	2.19	57.56	9.04
0.9	-2.61	0.34	0.35	2.19	70.56	1.79
0.62	-2.54	0.52	0.37	2.19	70.94	1.67
3.64	0.17	0.03	1.35	2.19	86.51	2.11
2.2	-3.77	-0.2	-0.03	2.19	64.75	2.26
5.93	-7.56	-2.97	-1.1	2.19	48.54	6.65

4.8	-6.99	-0.41	-0.93	2.19	50.71	2.21
9.05	-19.02	-3.35	-3.11	2.19	19.16	7.7
1.4	-3	0.45	0.22	2.19	68.54	1.66
0.68	-3.28	1.03	0.13	2.19	67.17	1.33
0.7	-3.27	1.57	0.14	2.19	67.23	1.08
5.92	-7.13	8	2.51	2.2	33.16	1.24
-11.48	-25.37	-15.84	-14.33	2.2	33.24	1.44
4.96	-13.53	-0.5	-2.33	2.2	30.24	2.23
3.58	0.25	-0.18	1.39	2.2	86.93	2.24
2.28	-3.98	0.15	-0.09	2.2	63.67	1.91
-0.35	-7.82	0.8	-1.16	2.2	47.52	1.39
2.9	-4.69	1.59	-0.32	2.2	60.39	0.99
1.25	0.13	0.25	1.35	2.2	86.21	1.93
1.57	-12.19	0.12	-2.08	2.2	33.68	2.08
3.12	-4.67	0.67	-0.31	2.21	60.45	1.56
0.69	-6.61	0.79	-0.84	2.21	52.15	1.53
2.3	-11.9	1.92	-1.73	2.21	33.37	1.04
0.15	-11.37	0.58	-1.92	2.21	35.94	1.62
-0.2	-7.58	2.29	-1.07	2.21	48.4	0.77
1.39	-3.01	0.55	0.24	2.21	68.43	1.63
1.26	0.15	-0.11	1.36	2.21	86.27	2.21
2.02	-4.45	-0.41	-0.23	2.21	61.4	2.33
1.52	-12.09	0.03	-2.06	2.21	33.92	2.12
-0.1	-8.07	0.16	-1.21	2.21	46.59	2
2.79	-4	-0.35	-0.07	2.21	63.56	2.62
-1.2	-14.18	0.28	-2.42	2.22	28.62	1.89
5.69	-7.11	7.65	2.46	2.22	33.4	1.36
2.62	-5.44	-1.15	-0.5	2.22	56.95	3.46
3.72	-5.1	-1.69	-0.41	2.22	58.45	4.22
3.93	-5.19	-1.73	-0.43	2.22	58.02	4.38
4.51	-4.92	-1.72	-0.35	2.22	59.27	4.26
1.45	-9.48	0.09	-1.53	2.22	41.71	2.02
0.18	-3.14	0.02	0.19	2.22	67.68	2.08
0.55	-3	0.06	0.24	2.22	68.43	2.11
0.97	-2.94	-0.35	0.26	2.22	68.71	2.57
4.24	-2.77	0.18	0.32	2.22	69.62	1.9
1.73	-3.91	2.65	-0.05	2.22	63.92	0.6
-0.12	-8.31	0.38	-1.25	2.22	45.72	1.86
3.94	-5.14	-0.24	-0.42	2.22	58.28	2.13
2.95	-10.5	-0.81	-1.75	2.22	38.5	2.64
1.31	-14.35	0.03	-2.46	2.22	28.25	2.36
0.31	-4.05	0.08	-0.11	2.22	63.28	2.03
7.88	-2.68	0.04	0.34	2.23	70.01	2
1.46	-14.09	-2.86	-2.41	2.23	28.83	7.07

3.08	-5.46	-1.57	-0.5	2.23	56.83	4.01
3.29	-5.58	-1.91	-0.54	2.23	56.32	4.62
4.65	-4.57	-1.69	-0.25	2.23	60.81	3.93
1.29	-9.33	-0.1	-1.49	2.23	42.19	2.23
5.83	-11.55	0.14	-1.94	2.23	35.4	1.86
0.19	-3.24	-0.36	0.17	2.23	67.16	2.56
1.66	-3.96	2	-0.06	2.23	63.62	0.83
0.47	-4.54	-0.46	-0.24	2.23	60.93	2.45
7.15	-7.83	-2.73	-1.11	2.23	47.38	6.09
0.99	-6.54	-0.46	-0.8	2.24	52.3	2.68
-0.43	-12.73	0.55	-2.47	2.24	33.4	1.31
2.31	-5.29	-2.18	-0.46	2.24	57.54	5.15
3.01	-5.44	-2.43	-0.49	2.24	56.89	5.45
5.4	-6.2	-1.02	-0.71	2.24	53.68	3.16
2.08	-6.99	-1.03	-0.93	2.24	50.52	3.18
2.35	-1.55	2.55	0.75	2.24	76.01	0.69
4.56	-1.16	0.85	0.89	2.24	78.25	1.42
1.38	-0.45	1.98	1.15	2.24	82.42	0.87
6.67	-13.85	-1.32	-2.36	2.25	29.35	3.25
-0.28	-12.68	0.51	-2.43	2.25	33.41	1.31
-3.47	-17.57	-5.37	-6.93	2.25	33.45	1.1
0.72	-11.49	1.7	-1.92	2.25	35.5	1.09
2.38	-4.24	0.36	-0.13	2.25	62.25	1.96
3.65	-5.48	-2.88	-0.5	2.25	56.67	6.78
4.19	-15.34	-2.7	-2.59	2.25	25.98	6.57
1.64	-1.84	-1.08	0.65	2.25	74.41	3.53
0.31	-3.21	-0.3	0.19	2.25	67.22	2.39
1.35	-2.91	0.1	0.28	2.25	68.72	2.06
3.38	2.25	-1.95	2.25	2.25	100	4.75
9.24	-8.95	-0.26	-1.37	2.25	43.37	2.43
1.36	1.27	1.33	1.83	2.25	93.22	1.21
-15.35	-29.42	-18.45	-18.43	2.26	33.45	0.71
3.84	-0.01	0.23	1.33	2.26	84.95	2.01
1.42	-8.17	1.11	-1.2	2.26	46.07	1.26
6.02	-7.61	-2.87	-1.06	2.26	48.09	6.93
6.17	-6.85	-1.81	-0.87	2.26	51.01	4.47
3.74	0.18	0.27	1.4	2.27	86.06	1.91
0.4	-11.33	0.4	-1.87	2.27	35.89	1.81
1.26	-2.85	0.62	0.32	2.27	68.94	1.64
1.29	-2.86	0.4	0.31	2.27	68.91	1.81
1.35	1.38	0.12	1.89	2.27	93.82	1.96
1.29	0.16	0.59	1.4	2.27	85.9	1.72
6.69	-8.99	0.23	-1.37	2.27	43.2	2.03
6.92	-9.1	-0.3	-1.39	2.27	42.81	2.53

7.43	-9.44	-0.16	-1.46	2.27	41.69	2.37
8.96	-9.51	-0.95	-1.48	2.27	41.48	3.21
9.23	-9.17	-0.6	-1.4	2.27	42.61	2.71
1.49	-1.46	0.07	0.79	2.27	76.35	1.91
-0.45	-12.94	0.86	-2.68	2.28	33.46	0.99
1.12	-8.41	0.14	-1.24	2.28	45.14	2.1
0.23	-11.17	0.62	-1.83	2.28	36.34	1.69
1.78	-5.66	3.1	-0.54	2.28	55.77	0.49
1.67	-12.18	0.64	-2.03	2.28	33.53	1.73
0.32	-4.14	0.69	-0.09	2.28	62.57	1.74
5.22	-5.94	-1.25	-0.61	2.29	54.57	3.74
1.07	-9.19	0.38	-1.41	2.29	42.46	2
1.85	-6.04	2.65	-0.64	2.29	54.14	0.61
4.37	0.72	-2.93	1.63	2.29	89.38	6.8
1.38	-2.91	-0.05	0.31	2.29	68.53	2.19
1.62	-12.16	0.36	-2.02	2.29	33.56	1.9
-0.13	-8.17	0.29	-1.18	2.29	45.99	1.86
-0.09	-8.03	0.43	-1.15	2.29	46.46	1.81
0.55	-7.61	0.34	-1.05	2.29	48.03	1.94
6.1	-8.71	-0.53	-1.29	2.29	44.08	2.68
6.12	-8.09	-0.57	-1.15	2.29	46.25	2.78
8.15	-9.18	-0.75	-1.4	2.29	42.49	2.97
8.55	-9.03	-0.77	-1.37	2.29	42.99	2.84
5.75	-4.43	-1.01	-0.17	2.3	61.14	3.16
3.32	-4.69	0.04	-0.26	2.3	59.99	2.31
0.84	-6.51	0.53	-0.76	2.3	52.2	1.72
2.66	-1.64	-0.08	0.74	2.3	75.23	2.3
-0.28	-3.61	0.59	0.08	2.3	65.03	1.87
1.32	-5.85	0.11	-0.59	2.3	54.91	2.22
1.15	-11.31	-2.68	-1.86	2.3	35.89	5.54
3.74	-8.48	2.37	-1.24	2.3	44.88	0.65
2.94	-0.63	0.14	1.11	2.3	81.03	2.17
5.24	-5.91	-1.13	-0.59	2.3	54.63	3.5
0.24	-11.02	0.41	-1.8	2.3	36.74	1.77
4.27	0.76	-2.62	1.66	2.3	89.53	6.17
-0.02	-3.32	-0.02	0.18	2.3	66.39	2.17
0.05	-3.06	0.37	0.27	2.3	67.76	1.91
1.59	-2.44	0.41	0.48	2.3	70.92	1.88
6.51	-4.41	0	-0.14	2.3	61.22	2.43
1.3	0.23	-0.12	1.45	2.3	86.24	2.2
4.18	-2.2	0.91	0.55	2.3	72.19	1.15
6.59	-8.63	-0.45	-1.27	2.3	44.32	2.63
3.54	0.37	1.32	1.5	2.3	87.07	1.24
2.27	1.05	1.99	1.77	2.3	91.46	1.01

3.3	-13.93	-3.21	-2.36	2.31	29.02	7.18
-0.1	-16.16	-0.9	-2.68	2.31	24.16	3.31
7.82	-14.69	-2.97	-2.44	2.31	27.28	6.83
2.84	-5.31	-1.09	-0.42	2.31	57.16	3.4
2.55	-12.63	0.64	-2.1	2.31	32.26	1.97
2.68	-6.22	0.17	-0.67	2.31	53.37	1.82
1.22	-9.42	-0.02	-1.45	2.31	41.64	2.14
1.34	-9.38	0.02	-1.45	2.31	41.78	2.12
-0.07	-3.37	-0.23	0.17	2.31	66.12	2.41
4.01	-2.61	-0.13	0.43	2.31	69.98	2.05
1.43	-2.48	0.03	0.47	2.31	70.67	2.18
1.76	-3.75	2.8	0.05	2.31	64.25	0.59
-0.18	-8.15	0.39	-1.16	2.31	45.97	1.83
3.51	0.43	0.97	1.53	2.31	87.38	1.39
5.18	-6.03	-1.06	-0.62	2.32	54.06	3.53
5.42	-5.9	-1.11	-0.58	2.32	54.63	3.49
5.42	-5.97	-0.9	-0.6	2.32	54.35	3.14
2.13	-7.13	-0.63	-0.93	2.32	49.72	2.92
9.76	-19.7	-3.38	-3.1	2.32	17.91	8.37
0.2	-3.13	-0.21	0.25	2.32	67.29	2.5
3.4	2.32	-2.01	2.33	2.32	100	5.02
3.4	2.32	-1.49	2.32	2.32	100	3.96
0.74	-3.2	1.34	0.24	2.32	66.93	1.22
5.17	-12.93	0.3	-2.13	2.32	31.47	2.18
7.28	-7.28	-2.59	-0.92	2.32	49.14	5.9
2.98	1.08	2.26	1.79	2.32	91.52	0.85
1.34	1.31	1.56	1.89	2.32	93.05	1.16
3.51	0.04	-1.73	1.38	2.32	84.94	4.59
1.11	-6.65	-0.94	-0.78	2.33	51.54	3.53
5.25	-7.4	7.14	2.2	2.33	33.48	1.36
-0.21	-16.31	-0.89	-2.68	2.33	23.83	3.11
6.32	-6.27	-1.99	-0.66	2.33	53.03	5.14
4.21	0.85	-1.93	1.71	2.33	89.98	4.7
1.33	-2.44	-0.41	0.49	2.33	70.76	2.62
0.77	-3.03	1.39	0.29	2.33	67.76	1.11
5.53	-12.83	-0.07	-2.1	2.33	31.68	2.21
-0.17	-8.59	-0.79	-1.25	2.33	44.38	3.2
1.1	0.22	1.71	1.46	2.33	85.97	1.06
6.5	-8.77	-0.22	-1.28	2.33	43.77	2.43
11.49	-0.81	-1.09	1.07	2.34	79.68	3.15
5.26	-6.17	-1.05	-0.63	2.34	53.41	3.72
0.49	-6.5	-1.96	-0.72	2.34	52.12	4.14
3.79	0.44	-2.59	1.55	2.34	87.28	6.26
3.84	0.5	-2.83	1.57	2.34	87.67	6.83

1.35	0.22	0.4	1.46	2.34	85.86	1.79
3.86	-12.75	-0.11	-2.09	2.34	31.9	2.43
2.49	-1.78	1.78	0.73	2.34	74.19	1.03
7.37	-9.31	0.37	-1.39	2.34	41.91	1.88
6.76	-13.39	-1.43	-2.22	2.35	30.25	3.97
6.77	-13.47	-1.23	-2.23	2.35	30.07	3.59
4.91	-4.49	-3.35	-0.15	2.35	60.67	8.12
2.32	-1.18	0.86	0.94	2.35	77.53	1.64
5.56	-13.19	-0.5	-2.18	2.35	30.74	2.62
0.63	-11.17	1.35	-1.79	2.35	36.17	1.23
0.99	-2.5	0.79	0.48	2.35	70.35	1.56
2.01	-0.54	-1.34	1.18	2.35	81.24	4.22
1.3	0.22	0.86	1.47	2.35	85.84	1.54
1.31	0.19	0.89	1.46	2.35	85.64	1.55
2.15	-3.46	2.1	0.17	2.35	65.51	0.85
1.95	-3.31	1.42	0.22	2.35	66.24	1.18
4.76	-0.93	1.76	1.04	2.35	78.99	1.04
6.96	-8.86	0.16	-1.29	2.35	43.39	2.11
2	-12.16	1.45	-1.99	2.36	33.48	1.32
1.23	-8.5	0.26	-1.22	2.36	44.59	2.15
12.44	-13.11	0	-2.14	2.36	30.93	2.41
6.69	-4.77	-2.43	-0.23	2.36	59.32	5.51
0.82	-2.92	0.33	0.34	2.36	68.19	2.02
1.46	-13.18	0.24	-2.16	2.37	30.71	2.03
2.07	-6.24	2.61	-0.65	2.37	53.01	0.79
1.18	-5.71	1.3	-0.51	2.37	55.21	1.25
3.85	0.51	-2.66	1.6	2.37	87.54	6.72
4.3	0.8	-2.46	1.71	2.37	89.38	5.79
7.57	-8.51	-0.54	-1.19	2.37	44.51	2.72
1.98	1.34	2.01	1.93	2.37	92.89	0.9
3.98	1.68	2.63	2.07	2.37	95.15	0.68
-1.12	-14.21	0.93	-2.33	2.38	28.23	1.64
8.24	-3.89	-2.19	0.04	2.38	63.31	5.12
5.32	-7.16	7.63	2.4	2.38	33.48	1.3
-0.24	-3.71	0.42	0.1	2.38	64.15	1.79
0.25	-9.75	2.01	-1.52	2.38	40.4	1.05
5.41	-6.22	-0.97	-0.63	2.38	53.07	3.43
1.65	-5.93	2.44	-0.55	2.38	54.27	0.75
-0.23	-3.52	0.23	0.17	2.38	65.05	2.07
0.04	-3.04	0.44	0.31	2.38	67.44	1.88
0.22	-3.03	0.22	0.32	2.38	67.5	2.1
4.49	-2.61	0.08	0.47	2.38	69.61	2.08
1.71	-2.37	0.79	0.55	2.38	70.91	1.64
3.72	-13.31	0.88	-2.15	2.38	30.38	1.58

1.28	-2.5	2.08	0.5	2.38	70.19	0.8
9.87	2.38	0.73	2.39	2.38	100	1.38
3.34	-0.1	-2.12	1.37	2.38	83.71	5.35
9.52	-5.47	10.07	3.87	2.39	33.49	1.05
5.13	-5.89	-0.86	-0.53	2.39	54.36	3.28
5.16	-6.09	-1.05	-0.59	2.39	53.59	3.51
3.01	-10.47	-0.72	-1.62	2.39	38.13	2.48
2.7	-5.17	0.27	-0.34	2.39	57.43	1.67
11.97	-13.12	-0.11	-2.13	2.39	30.84	2.06
1.11	-6.26	-1.43	-0.63	2.39	52.9	3.9
1.79	-6.02	3.02	-0.58	2.39	53.85	0.54
3.43	2.39	-0.85	2.39	2.39	100	3.38
2.73	-12.36	0.69	-1.98	2.39	32.79	1.75
2.89	-2.38	2.51	0.55	2.39	70.74	0.75
0.43	-7.03	-0.03	-0.85	2.39	49.85	2.38
1.44	-0.71	2.58	1.14	2.39	79.97	0.75
3.5	0.43	1.74	1.57	2.39	86.88	1.04
3.39	0.24	-1.31	1.51	2.39	85.71	4
0.1	-16.11	-0.55	-2.61	2.4	24.11	3
5.13	-5.7	-1.16	-0.48	2.4	55.16	3.6
10.5	-19.16	-3.36	-2.99	2.4	18.65	8.77
0.19	-3.07	-0.36	0.32	2.4	67.2	2.65
1.14	-3.87	2.92	0.06	2.4	63.29	0.63
1.85	-3.44	2.46	0.2	2.4	65.36	0.7
2.13	-4.1	-0.02	0	2.4	62.23	2.11
1.87	-12.19	0.57	-1.95	2.4	33.21	1.88
1.43	0.21	0.93	1.5	2.4	85.49	1.48
3.2	0.32	-1.69	1.54	2.4	86.13	4.61
0.07	-4.02	-0.92	0.02	2.41	62.51	2.6
4.02	-2.27	0.02	0.59	2.41	71.22	1.99
4	-0.07	0.61	1.4	2.41	83.69	1.85
4.63	-4.67	-2.66	-0.17	2.41	59.56	6.05
1.68	-1.77	-0.84	0.76	2.41	73.93	3.43
3.41	2.41	-2.04	2.41	2.41	100	5.14
2.28	-2.05	2.32	0.69	2.41	72.38	0.74
2.39	-6.66	-1.54	-0.71	2.41	51.21	4.5
1.05	0.32	2.04	1.54	2.41	86.05	0.89
7.2	-8.31	0.43	-1.12	2.41	45.07	1.93
7.31	-9.03	0.82	-1.29	2.41	42.63	1.57
3.23	0.3	-1.78	1.53	2.41	85.96	4.73
-1.64	-15.81	-1.9	-5.31	2.42	33.49	0.64
2.49	-5.06	-1.62	-0.29	2.42	57.78	4.86
0.53	-8.59	3.01	-1.21	2.42	44.1	0.54
6.28	-7.58	-2.63	-0.95	2.42	47.68	6.92

2	-6.76	-1.41	-0.77	2.42	50.81	4.01
2.53	0.22	0.03	1.51	2.42	85.41	2.32
2.85	-2.61	1.54	0.49	2.42	69.45	1.16
3.01	-12.3	0.95	-1.96	2.42	32.88	1.68
2.41	-2.08	2.42	0.67	2.42	72.22	0.73
2.53	-1.63	2.17	0.83	2.42	74.65	0.87
1.27	0.24	1.46	1.51	2.42	85.58	1.22
2.14	1.31	1.85	1.95	2.42	92.34	0.95
3.08	-1.65	0.2	0.82	2.43	74.49	2.19
-0.25	-12.69	0.81	-2.47	2.43	33.52	1.19
-4.13	-17.86	-5.51	-7.22	2.43	33.52	1.06
0.48	-11.12	0.81	-1.73	2.43	36.09	1.63
7.04	1.63	-2.93	2.09	2.43	94.49	7.64
0.99	-2.88	0.63	0.4	2.43	67.98	1.78
1.15	-2.9	0.87	0.39	2.43	67.92	1.6
1.23	-2.89	0.75	0.4	2.43	67.93	1.63
6.83	-8.54	0.39	-1.16	2.43	44.22	1.89
6.98	-8.66	0.89	-1.19	2.43	43.81	1.51
0.89	-2.39	0.74	0.57	2.44	70.45	1.58
3.04	-0.71	0.3	1.17	2.44	79.71	2.18
1.62	-13.11	0.71	-2.1	2.44	30.75	1.78
1.24	-2.84	0.71	0.42	2.44	68.2	1.6
1.11	0.22	0.61	1.52	2.44	85.28	1.87
1.69	-2.28	1.07	0.61	2.44	71.09	1.48
1.7	-2	1.37	0.71	2.44	72.53	1.35
2.08	1.35	2.05	1.98	2.44	92.49	0.9
2.4	-8.68	0.47	-1.23	2.45	43.7	1.57
-3.03	-14.97	-2.74	-4.58	2.45	33.54	1.44
1.11	-5.4	0.59	-0.37	2.45	56.22	2.22
1.17	-5.36	2.15	-0.36	2.45	56.37	0.85
2.47	-4.16	1.03	0.01	2.45	61.76	1.48
0.3	-2.98	0.32	0.38	2.45	67.4	2.06
0.5	-2.97	0.35	0.39	2.45	67.46	1.93
2.13	-4.05	1.2	0.03	2.45	62.24	1.4
2.41	-2.34	0.69	0.6	2.45	70.68	1.87
2.21	-3.95	0.19	0.07	2.45	62.74	2.15
2.19	-3.92	-0.03	0.08	2.45	62.83	2.35
0.02	-7.99	0.76	-1.04	2.45	46.1	1.76
2.85	-4.09	0.07	0.04	2.45	62.07	2.33
4.03	1.7	2.7	2.13	2.45	94.78	0.73
3.5	-4.68	0.57	-0.16	2.46	59.31	1.9
1.42	-8.58	0.36	-1.18	2.46	43.99	2.28
4.84	2.43	-4.44	2.44	2.46	99.78	12.51
1.11	-9.24	0.46	-1.32	2.46	41.78	1.97

7.6	-5.8	-2.84	-0.48	2.46	54.49	7.21
-0.22	-3.56	0.53	0.2	2.46	64.53	2.05
0.38	-2.99	0.08	0.38	2.46	67.32	2.25
1.19	-2.99	0	0.38	2.46	67.32	2.59
1.87	-2.31	0.98	0.61	2.46	70.82	1.55
4.2	-13.28	0.8	-2.1	2.46	30.3	1.59
5.6	-12.92	0.57	-2.03	2.46	31.18	1.68
2.93	-2.47	3.19	0.57	2.46	69.93	0.57
0.46	-4.53	0.29	-0.1	2.46	59.99	2.09
3.95	1.82	2.26	2.18	2.46	95.55	0.82
5.22	-0.63	-2.02	1.21	2.46	80.02	5.37
6.43	-7.09	-1.45	-0.8	2.47	49.31	4.75
1.18	-9.35	0.14	-1.34	2.47	41.39	2.14
8.27	-7.34	-3.12	-0.87	2.47	48.38	8.21
-0.24	-3.5	0.19	0.22	2.47	64.73	2.2
2.53	-1.66	2.58	0.85	2.47	74.22	0.72
-0.05	-8.52	-0.64	-1.14	2.47	44.17	3.2
7.42	-8.05	-2.29	-1.01	2.47	45.79	5.61
5.32	1.91	1.48	2.23	2.47	96.12	1.24
4.28	-0.25	-1.78	1.37	2.47	82.21	4.74
6.49	-6.24	-2.61	-0.57	2.48	52.63	5.93
2.11	-7.27	-0.5	-0.86	2.48	48.65	3.11
-0.07	-3.38	-0.38	0.27	2.48	65.29	2.59
3.44	2.48	-1.13	2.48	2.48	100	3.81
3.43	2.48	-1.46	2.48	2.48	100	4.31
5.34	-12.86	0.51	-2.02	2.48	31.28	1.84
3.94	1.86	1.41	2.21	2.48	95.73	1.26
3.34	0.67	-0.84	1.72	2.48	87.91	3.34
3.2	0.58	-1.27	1.69	2.48	87.29	4.03
1.34	-11.13	-1.13	-1.72	2.49	35.92	3.88
-0.33	-13.39	-0.92	-2.17	2.49	29.93	3.18
6.31	-8.3	8.79	2.97	2.49	27.78	1.44
-0.26	-16.3	-0.86	-2.58	2.49	23.56	3.51
5.39	-6.19	-1.27	-0.55	2.49	52.8	3.94
2.65	-1.32	-2.12	0.96	2.49	75.99	5.89
17.17	-11.17	0.29	-1.71	2.49	35.8	1.53
4.26	0.82	-1.89	1.79	2.49	88.75	5.08
0.22	-2.95	-0.55	0.41	2.49	67.35	2.68
3.42	2.49	-1.67	2.49	2.49	100	4.56
3.91	1.91	0.93	2.24	2.49	95.9	1.6
1.99	-12.17	0.33	-1.9	2.49	33.04	2.14
4.14	-12.65	0.38	-1.98	2.49	31.79	2.05
2.16	-1.37	0.32	0.96	2.49	75.69	2.06
3.3	0.57	-1.28	1.69	2.49	87.21	3.91

12.22	-0.97	-1.08	1.1	2.5	77.9	3.69
2.12	0.16	0.92	1.54	2.5	84.55	1.56
2.46	-6.58	-1.4	-0.63	2.5	51.22	4.45
1.15	0.31	1.77	1.59	2.5	85.46	1.09
1.23	0.31	1.79	1.59	2.5	85.49	1.05
1.2	0.32	1.96	1.59	2.5	85.54	0.98
3.14	1.06	1.57	1.89	2.5	90.21	1.21
1.63	-11.57	-1.34	-1.8	2.51	34.62	4.69
2.39	-1.07	0.99	1.08	2.51	77.27	1.6
2.45	-1.2	1.01	1.02	2.51	76.58	1.73
4.56	-7.21	7.47	2.35	2.51	33.54	1.32
-1.68	-13.52	-0.31	-3.24	2.51	33.56	1.13
1.61	-14.62	-2.18	-2.31	2.51	27.05	6
3.09	1.21	0.87	1.96	2.51	91.11	1.65
4.47	-14.8	-2.68	-2.34	2.51	26.65	7.26
2.71	-10.18	1.2	-1.49	2.51	38.66	1.55
0.35	-3.77	0.62	0.17	2.51	63.26	1.95
4.78	1.78	0.95	2.19	2.51	94.97	1.79
3.02	0.43	-0.12	1.64	2.51	86.19	2.44
1.63	-2.3	1.97	0.65	2.51	70.55	1.07
1.26	0.25	1.4	1.58	2.51	85.02	1.35
-3.12	-17.33	-3.93	-6.73	2.52	33.57	0.73
-0.01	-16.17	-0.71	-2.54	2.52	23.78	3.21
1.72	-9.75	1.25	-1.4	2.52	39.97	1.42
1.78	-13.09	0.3	-2.05	2.52	30.64	2.32
1.93	-6.14	3.4	-0.53	2.52	52.87	0.51
1.67	-9.68	1.07	-1.38	2.52	40.18	1.57
2.21	-2.34	0.82	0.64	2.52	70.33	1.71
5.13	1.84	1	2.23	2.52	95.26	1.78
2.93	-4.07	0.42	0.1	2.52	61.8	2.09
3.07	-4.17	1	0.06	2.52	61.38	1.6
-0.15	-9.75	0.24	-1.43	2.53	39.97	2.35
0.88	-11.33	2.04	-1.71	2.53	35.26	0.96
3.15	0.91	0.91	1.84	2.53	89.09	1.66
5.09	-6.01	-0.37	-0.48	2.53	53.33	2.88
5.32	-6.43	-0.61	-0.59	2.53	51.66	3.18
5.36	-6.5	-0.63	-0.61	2.53	51.4	3.31
1.61	-9.64	0.64	-1.36	2.53	40.3	1.86
1.83	0.39	0.09	1.63	2.53	85.78	2.45
2.1	-12.22	0.31	-1.88	2.53	32.8	2.24
0.01	-8.01	0.53	-1	2.53	45.74	1.93
0.04	-7.95	0.55	-0.99	2.53	45.97	2.01
0.86	-7.67	0.66	-0.92	2.53	46.97	1.91
0.38	-4.14	1.01	0.05	2.53	61.47	1.7

0.4	-4.33	0.67	0	2.53	60.6	1.83
3.66	-14.14	-2.57	-2.24	2.54	28.07	6.57
8.77	-4.24	-1.92	0.03	2.54	60.96	5.29
3.47	-1.7	0.16	0.87	2.54	73.63	2.39
5.12	-5.64	0.07	-0.37	2.54	54.85	2.38
5.31	-6.48	-0.84	-0.6	2.54	51.43	3.49
0.54	-10.99	0.58	-1.64	2.54	36.2	1.79
1.52	-6.13	2.93	-0.51	2.54	52.81	0.65
1.15	-9.29	0.54	-1.28	2.54	41.37	1.89
-0.24	-3.61	0.77	0.23	2.54	63.94	1.71
1.67	0.32	0.03	1.62	2.54	85.33	2.43
2.13	0.22	0.5	1.58	2.54	84.69	1.91
4.39	-0.57	1.6	1.28	2.54	79.99	1.16
0.41	-4.44	0.67	-0.03	2.54	60.09	1.82
2.01	1.48	2.41	2.09	2.54	92.72	0.76
-7.51	-21	-9.78	-10.2	2.55	33.58	1.35
-0.16	-3.5	0.22	0.27	2.55	64.36	2.05
2.13	-0.46	-0.88	1.33	2.55	80.54	3.84
7.19	-4.09	0.54	0.1	2.55	61.59	2.06
2.36	-0.26	1.89	1.4	2.55	81.71	1.06
5.38	1.99	1.59	2.31	2.55	96.06	1.23
4.77	-0.21	-1.85	1.42	2.55	82.01	4.98
3.21	0.81	-0.32	1.82	2.55	88.26	2.78
0.23	-15.89	-0.24	-2.48	2.56	24.28	2.86
4.43	-0.15	1.22	1.45	2.56	82.34	1.56
5.1	2.51	-3.79	2.54	2.56	99.59	11.76
1.28	-5.62	2.09	-0.36	2.56	54.85	1.06
2.74	-1.48	-1.14	0.95	2.56	74.69	4.27
1.82	-1.68	-0.42	0.88	2.56	73.64	3.02
1.51	-4	2.99	0.11	2.56	61.98	0.66
1.15	-14.46	0.34	-2.25	2.56	27.3	1.9
4.6	-0.57	1.71	1.28	2.56	79.85	1.15
2.66	-0.17	1.69	1.44	2.56	82.21	1.21
3.26	0.84	1.8	1.83	2.56	88.43	1.14
4.26	1.8	2.19	2.23	2.56	94.73	1
3.33	0.91	-0.89	1.86	2.56	88.88	3.59
3.34	0.92	-0.96	1.86	2.56	88.93	3.73
3.37	0.94	-1.13	1.88	2.56	89.07	3.99
3.41	1.1	-0.96	1.95	2.56	90.14	3.85
1.23	-6.62	-0.75	-0.62	2.57	50.8	3.62
3.59	0.79	0.75	1.82	2.57	88.05	1.72
5.24	-5.73	-1.6	-0.38	2.57	54.37	4.78
5.16	-6	-1.74	-0.45	2.57	53.25	5.15
5.41	-6.03	-1.37	-0.46	2.57	53.11	4.23

4.03	0.62	-1.86	1.75	2.57	86.98	5.43
1.71	-1.67	-0.86	0.89	2.57	73.61	3.56
1.78	-1.72	-0.09	0.87	2.57	73.39	2.64
1.2	-3.87	2.9	0.16	2.57	62.54	0.62
4.36	-12.46	0.97	-1.88	2.57	32.11	1.6
3.46	-2.21	2.81	0.72	2.57	70.76	0.65
1.6	-2.08	2.21	0.77	2.57	71.45	0.89
1.64	-0.57	2.73	1.3	2.57	79.75	0.72
2.13	0.02	1.76	1.52	2.57	83.26	1.16
2.63	0.03	1.88	1.52	2.57	83.32	1.08
2.57	0.23	1.77	1.6	2.57	84.56	1.12
1.84	-7.29	2.29	-0.8	2.58	48.2	0.96
12.92	-13	0.61	-1.99	2.58	30.74	2.05
3.51	-6.02	0.79	-0.45	2.58	53.14	1.68
1.36	-3.1	1.66	0.42	2.58	66.23	1.16
1.49	-2.61	1.35	0.58	2.58	68.65	1.44
0.81	-2.45	1.46	0.63	2.58	69.46	1.34
3.58	-12.53	0.7	-1.89	2.58	31.9	1.75
2.74	-0.63	1.36	1.27	2.58	79.42	1.4
2.74	-0.53	1.34	1.31	2.58	79.97	1.43
1.93	-0.06	1.38	1.49	2.58	82.71	1.43
2.55	0.14	1.23	1.57	2.58	83.96	1.43
2.23	-4.06	0.39	0.12	2.59	61.57	2.16
6.47	-7.47	-2.54	-0.83	2.59	47.53	7
17.83	-11.3	0.42	-1.68	2.59	35.18	1.7
0.46	-3.81	0.52	0.21	2.59	62.72	2
2.05	0.35	1.01	1.66	2.59	85.18	1.51
3.45	1.15	-1.4	1.98	2.59	90.23	4.57
5.25	-5.16	-2.8	-0.2	2.6	56.62	7.75
1.42	-11.09	-1.37	-1.65	2.6	35.75	4.5
19.27	-4.08	11.09	5.06	2.6	33.58	1.12
8.25	-15.3	-2.47	-2.35	2.6	25.43	6.9
2.6	-10.2	1.15	-1.43	2.6	38.37	1.54
0.14	-3.7	0.87	0.24	2.6	63.19	1.71
0.07	-3.68	0.82	0.24	2.6	63.28	1.87
1.81	-1.97	1.46	0.81	2.6	71.9	1.38
3.13	-4.3	1.06	0.07	2.6	60.41	1.64
0.18	-19.22	-4.38	-6.7	2.61	27.77	1.01
-0.44	-12.66	0.44	-2.46	2.61	33.59	1.34
1.41	-11.52	2.88	-1.46	2.61	33.59	0.83
5.39	-6.44	-0.35	-0.55	2.61	51.33	3.05
1.83	-9.82	1.05	-1.35	2.61	39.51	1.65
2.51	-10.26	0.62	-1.44	2.61	38.16	2.01
2.01	0.29	0.17	1.64	2.61	84.66	2.42

2.5	0.02	0.32	1.54	2.61	83.05	2.26
1.8	-0.63	0.91	1.29	2.61	79.26	1.7
1.59	-2.16	2.83	0.75	2.61	70.82	0.63
0.42	-12.85	1.2	-1.92	2.61	31.02	1.57
0.2	-7.9	0.49	-0.93	2.61	45.9	2.11
0.9	-7.68	0.81	-0.87	2.61	46.69	1.98
5.5	2.07	2	2.37	2.61	96.17	1.15
-4.83	-18.81	-6.13	-8.13	2.62	33.62	0.82
2.44	-3.69	0.72	0.25	2.62	63.14	2.01
3.13	-0.2	0.6	1.46	2.62	81.72	2.07
2.59	-2.29	0.68	0.72	2.62	70.11	2.01
3.51	0.81	2.37	1.85	2.62	87.86	0.87
-0.61	-13.04	0.48	-2.83	2.63	33.68	1.13
-1.07	-15.2	-3.05	-4.82	2.63	33.71	1.41
-12.13	-25.54	-15.75	-14.65	2.63	33.74	1.23
6.14	-13.2	-0.4	-2	2.63	30.13	2.8
8.08	-14.79	-2.54	-2.25	2.63	26.45	6.56
-0.17	-3.51	0.36	0.31	2.63	63.96	2.25
1.48	-6.17	3.25	-0.47	2.63	52.34	0.54
-0.11	-3.54	0.38	0.31	2.63	63.84	2.19
5.07	-4.98	-0.18	-0.13	2.63	57.31	2.91
4.56	-4.56	0.42	0.01	2.63	59.15	2.04
4.49	-0.39	2.05	1.39	2.63	80.54	1.01
0.27	-3.92	0.25	0.18	2.63	62.02	1.94
3.39	0.83	2.03	1.87	2.63	87.98	1.03
1.98	1.59	2.33	2.18	2.63	92.86	0.82
3.8	-5.07	-0.45	-0.17	2.64	56.89	3.26
8.27	-2.22	0.14	0.74	2.64	70.36	2.03
-1.62	-13.46	0.08	-3.23	2.64	33.74	0.92
5.38	-5.39	-0.98	-0.24	2.64	55.49	3.77
5.08	-5.89	-0.91	-0.38	2.64	53.41	3.78
5.4	-6.13	-0.46	-0.45	2.64	52.46	3.12
1.56	-6.03	2.14	-0.42	2.64	52.83	1.02
11.18	-18.06	-3.17	-2.71	2.64	20.12	9.07
5.27	-4.97	-0.56	-0.12	2.64	57.28	3.31
0.11	-8.59	-0.1	-1.06	2.64	43.4	2.95
1.51	-0.81	3.03	1.25	2.64	78.02	0.64
2.66	-0.12	1.86	1.51	2.64	82.03	1.12
2.66	-0.05	2.27	1.53	2.64	82.48	0.94
3.08	1.39	2.73	2.11	2.64	91.48	0.75
5.45	-6.3	-2.5	-0.49	2.65	51.74	7.46
-1.38	-16.94	-1.36	-4.61	2.65	27.75	0.96
5.35	-5.67	-1.17	-0.31	2.65	54.3	4.27
5.41	-5.91	-0.98	-0.38	2.65	53.32	3.78

4.42	-6.42	1.85	-0.5	2.65	51.28	1.06
1.49	-6.14	2.91	-0.45	2.65	52.37	0.69
4.07	0.68	-2.13	1.82	2.65	86.84	6.2
4.34	0.85	-1.75	1.89	2.65	87.93	5.18
1.74	-0.67	2.47	1.31	2.65	78.72	0.86
2.42	-0.38	1.93	1.41	2.65	80.4	1.03
2.31	-0.22	3.01	1.48	2.65	81.4	0.65
3.6	0.92	2.14	1.92	2.65	88.4	0.97
4.2	1.96	1.85	2.35	2.65	95.16	1.09
3.47	1.43	-1.36	2.13	2.65	91.65	4.52
3.41	1.43	-1.8	2.13	2.65	91.68	5.38
1.55	-11.5	-1.45	-1.69	2.66	34.43	4.45
6.7	-11.33	5.57	0.43	2.66	27.59	1.33
-12.03	-25.41	-15.35	-14.53	2.66	33.76	1.18
4.67	2.16	-1.2	2.44	2.66	96.53	4.26
4.68	2.21	-1.34	2.47	2.66	96.84	4.62
5.31	-5.82	-1.8	-0.35	2.66	53.64	5.27
18.81	-10.14	0.33	-1.41	2.66	38.38	2.52
1.19	-3.11	2.58	0.47	2.66	65.78	0.81
1.69	-0.59	2.29	1.34	2.66	79.19	0.91
2.31	-0.54	1.86	1.36	2.66	79.47	1.15
1.78	1.56	1.64	2.19	2.66	92.5	1.25
3.31	1.41	-2.03	2.13	2.66	91.46	5.85
-3	-14.97	-2.36	-4.65	2.67	33.77	1.32
1.56	-8.52	1.15	-1.03	2.67	43.52	1.71
0.35	-7.62	2.67	-0.8	2.67	46.67	0.77
2.54	0.03	0.73	1.58	2.67	82.76	1.83
0.86	-2.45	1.42	0.69	2.67	68.98	1.45
1.68	-2.44	1.73	0.69	2.67	69.09	1.39
1.63	-0.86	2.1	1.25	2.67	77.59	0.98
7.64	-8.04	-1.98	-0.89	2.67	45.2	5.53
6.86	-13.27	-0.83	-1.98	2.68	29.83	3.27
11.41	-14.38	5.04	-0.53	2.68	22.67	0.74
3.98	-13.63	2.84	-1.57	2.68	27.51	1.1
4.64	2.15	-0.45	2.45	2.68	96.3	3.25
13.52	-13.63	0.42	-2.03	2.68	28.97	2.47
2.18	-0.39	-0.61	1.43	2.68	80.27	3.54
0.24	-3.79	0.91	0.26	2.68	62.45	1.68
3.31	0.61	0.06	1.81	2.68	86.29	2.41
1.89	-0.03	1.05	1.56	2.68	82.32	1.71
1.94	-0.6	0.7	1.35	2.68	78.97	1.88
0.86	-2.4	1.22	0.71	2.68	69.21	1.42
6.05	-0.8	2.01	1.27	2.68	77.84	0.98
3.13	1.45	2.85	2.15	2.68	91.63	0.66

-1.57	-13.51	0.41	-3.29	2.69	33.79	0.79
0.95	-8.33	1.86	-0.98	2.69	44.12	1.05
5.12	-5.8	-1.6	-0.33	2.69	53.62	5.18
0.01	-3.74	0.83	0.28	2.69	62.62	1.91
-0.23	-3.64	1.12	0.31	2.69	63.05	1.63
4.05	-2.04	0.93	0.84	2.69	71.04	1.95
4.88	2.11	1.35	2.44	2.69	95.97	1.38
-0.48	-9.84	0.8	-1.35	2.7	39.19	2.03
3.87	-1.67	0.22	0.97	2.7	72.98	2.57
1.48	-5.83	0.89	-0.33	2.7	53.45	1.66
1.9	-11.4	-2.07	-1.63	2.7	34.65	5.49
0.43	-7.57	2.67	-0.77	2.7	46.76	0.82
5.06	-5.7	-1.77	-0.29	2.7	53.99	5.38
11.83	-19.71	-2.16	-2.86	2.7	17.41	6.74
-0.2	-3.68	1.05	0.3	2.7	62.88	1.67
1.14	-3	0.41	0.53	2.7	66.12	2.45
2.08	0.32	0.14	1.7	2.7	84.35	2.36
2.35	-12.43	0.37	-1.81	2.7	31.88	2.39
3.55	-1	1.49	1.22	2.7	76.6	1.39
0.24	-7.8	0.81	-0.85	2.7	45.94	1.92
1.78	-15.5	-1.84	-2.31	2.71	24.79	6.08
4.7	2.28	-1.53	2.52	2.71	97	5.11
5.41	-6.49	-0.52	-0.5	2.71	50.77	3.35
2.13	-12.98	1.48	-1.91	2.71	30.48	1.65
2.02	-6.08	3.25	-0.4	2.71	52.41	0.52
11.13	-8.79	-3.17	-1.07	2.71	42.53	9.55
8.96	-6.93	-2.97	-0.62	2.71	49.09	7.62
4.14	0.73	-2.2	1.88	2.71	86.75	6.56
0.37	-7.62	0.43	-0.79	2.71	46.56	2.35
7.43	-13.91	-2	-2.07	2.71	28.27	5.87
4.65	-0.21	1.9	1.51	2.71	81.11	1.12
2.68	-6.61	-0.88	-0.51	2.71	50.31	3.94
2.01	1.63	2.15	2.24	2.71	92.58	0.96
5.51	-0.55	-1.82	1.39	2.71	79.15	5.18
-0.04	-13.75	-0.39	-2.08	2.72	28.61	3.35
5.06	-6.09	-0.25	-0.39	2.72	52.36	2.88
5.39	-6.26	-0.48	-0.43	2.72	51.64	3.27
1.87	-1.67	-0.25	0.98	2.72	72.85	3.17
1.66	0.91	-0.23	1.96	2.72	87.84	3.09
0.39	-7.03	0.07	-0.64	2.72	48.69	2.41
2.12	-0.52	1.98	1.4	2.72	79.23	1.1
2.02	1.6	1.82	2.24	2.72	92.32	1.18
0.41	-15.84	-0.16	-2.36	2.73	24.08	2.99
2.69	-4.71	-1.14	0.01	2.73	58.04	4.66

5.02	-5.53	-1.69	-0.22	2.73	54.57	5.45
1.56	-6.31	2.36	-0.45	2.73	51.41	0.94
1.64	-6.26	2.52	-0.43	2.73	51.61	0.88
4.31	0.9	-1.45	1.95	2.73	87.73	4.77
1.54	-0.9	2.99	1.27	2.73	77	0.69
7.15	-13.43	-2.51	-1.97	2.73	29.34	6.55
6.84	-13.48	-0.15	-1.98	2.74	29.19	2.69
6.82	-10.65	0.6	-1.44	2.74	36.67	1.88
4.29	0.87	-1.69	1.95	2.74	87.54	5.34
7.03	-3.74	0.38	0.32	2.74	62.38	2.03
1.92	-0.03	0.65	1.59	2.74	82.01	2.1
3.08	-2.64	2.72	0.67	2.74	67.72	0.79
2.16	-0.08	2.41	1.58	2.74	81.73	0.91
4.18	2.05	2.11	2.44	2.74	95.24	1
6.8	-14.2	-0.62	-2.09	2.75	27.54	3.56
6.57	-6.98	-2.29	-0.6	2.75	48.75	7.01
0.58	-3.78	0.83	0.3	2.75	62.16	1.8
1.23	-3.16	2.21	0.51	2.75	65.1	0.97
2.38	0	1.18	1.62	2.75	82.09	1.68
1.88	-1.97	2.33	0.89	2.75	71.11	1
2.26	-0.48	1.93	1.43	2.75	79.27	1.13
2.37	-0.4	1.85	1.46	2.75	79.79	1.14
3.36	-4.39	0.56	0.14	2.75	59.4	2.26
3.45	1.64	-1.84	2.28	2.75	92.44	5.7
3.47	1.65	-1.97	2.28	2.75	92.5	5.96
3.55	1.72	-1.71	2.31	2.75	92.93	5.55
-1.21	-13.29	0.95	-3.09	2.76	33.82	0.67
2.83	-10.36	1.6	-1.37	2.76	37.46	1.45
1.58	-2.55	0.76	0.71	2.76	68.06	2.04
0.26	-7.57	2.66	-0.73	2.76	46.57	0.79
1	-3.85	0.63	0.29	2.76	61.82	2.07
3.48	0.69	0.16	1.88	2.76	86.29	2.43
2.39	-0.62	1.24	1.39	2.76	78.44	1.57
6.48	-5.37	-1.25	-0.16	2.77	55.09	4.36
4.74	2.33	-1.68	2.58	2.77	96.99	5.47
-0.07	-3.66	1.2	0.36	2.77	62.64	1.64
5.74	-3.21	1.21	0.49	2.77	64.77	1.25
2.13	-0.03	0.87	1.61	2.77	81.85	1.92
2.25	-0.67	0.96	1.37	2.77	78.09	1.82
2.38	-3.59	0.48	0.38	2.77	62.95	1.99
3.68	-1.04	1.77	1.24	2.77	76.01	1.22
0.97	-7.72	0.93	-0.78	2.77	46.01	2
1.25	-8.76	1.26	-1.03	2.77	42.44	1.96
-1.17	-16.39	-4.6	-5.95	2.78	33.83	1.46

3.57	-2.61	0.56	0.69	2.78	67.64	1.84
2.56	-3.77	1.11	0.32	2.78	62.08	1.84
1.73	-6.99	1.95	-0.6	2.78	48.64	1.19
5.81	-7.37	0.08	-0.67	2.78	47.24	2.34
2.67	-0.63	1.29	1.39	2.78	78.31	1.52
2.49	-12.57	0.55	-1.79	2.78	31.34	2.27
4.82	-4.53	0.56	0.1	2.78	58.64	2.1
2.65	1.31	3.16	2.16	2.78	90.04	0.72
6.73	-1.81	-1.73	0.98	2.78	71.75	5.61
5.79	-0.75	-1.17	1.36	2.78	77.59	4.34
3.24	-0.36	0.5	1.5	2.79	79.78	2.31
2.04	-9.94	0.52	-1.26	2.79	38.63	2.39
14.83	-11.82	-2.89	-1.66	2.79	33.29	8.81
14.07	-10.36	-2.85	-1.37	2.79	37.37	8.5
0.29	-7.76	0.8	-0.78	2.79	45.81	1.99
0.33	-7.72	0.66	-0.77	2.79	45.93	2.11
0.79	-7.68	0.49	-0.76	2.79	46.07	2.05
4.97	-0.19	1.85	1.56	2.79	80.72	1.2
4.68	-0.11	1.79	1.59	2.79	81.24	1.27
7.22	-1.22	-1.76	1.19	2.79	74.94	5.8
3.9	-5.25	-0.07	-0.13	2.8	55.44	3.08
9.24	-3.91	-2.11	0.29	2.8	61.36	5.96
5.01	-5.46	-1.07	-0.16	2.8	54.58	4.41
5.42	-5.91	-0.64	-0.29	2.8	52.72	3.56
2.11	-7.39	-0.23	-0.7	2.8	47.07	3.5
1.92	-9.77	0.65	-1.23	2.8	39.14	2.16
0.41	-7.49	0.22	-0.71	2.8	46.72	2.46
5.26	2.21	2.36	2.54	2.8	95.83	0.96
6.83	-13.51	-0.06	-1.95	2.81	28.99	2.87
8.43	-7.25	0.54	-0.63	2.81	47.6	1.8
0.6	-15.75	-0.16	-2.3	2.81	24.12	3.19
4.59	2.24	-0.64	2.56	2.81	96.03	3.59
10.43	-8.41	-3.07	-0.93	2.81	43.52	8.79
-0.17	-3.75	1.36	0.35	2.81	62.06	1.49
4.75	-2.05	2.22	0.9	2.81	70.4	0.91
1.72	0.92	-0.15	2.01	2.81	87.36	3.21
1.8	-0.61	2.56	1.42	2.81	78.23	0.91
2.69	-0.28	2.42	1.53	2.81	80.14	0.96
5.66	-7.27	-2.18	-0.63	2.82	47.45	6.83
5.98	-8.74	-2.31	-0.99	2.82	42.36	7.58
1.4	-6.57	-0.35	-0.46	2.82	50.1	3.49
4.23	0.78	-1.56	1.96	2.82	86.47	5.52
4.38	0.96	-1.58	2.03	2.82	87.56	5.16
3.35	-3.17	2.68	0.55	2.82	64.72	0.92

2.03	-0.2	2.18	1.57	2.82	80.53	1.25
4.39	-0.01	1.48	1.66	2.82	81.61	1.49
3.44	-4.51	0.34	0.14	2.82	58.57	2.66
3.74	-2.3	2.09	0.83	2.82	69.02	1.55
3.6	1.87	-1.59	2.41	2.82	93.43	5.35
3.64	1.89	-1.57	2.42	2.82	93.57	5.33
3.67	1.94	-1.7	2.44	2.82	93.94	5.62
3.7	2.01	-1.77	2.47	2.82	94.36	5.79
3.75	2.06	-1.62	2.49	2.82	94.73	5.55
4.96	-6.39	1.62	-0.39	2.83	50.73	1.33
1.4	-3.66	0.59	0.39	2.83	62.37	2.17
1.6	-1.08	2.29	1.26	2.83	75.5	0.95
2.02	1.65	1.58	2.32	2.83	91.99	1.39
0.52	-7.49	2.61	-0.66	2.84	46.58	0.92
1.15	-3.78	2.68	0.35	2.84	61.76	0.69
4.92	2.24	1.09	2.57	2.84	95.79	1.63
2.26	0.04	0.56	1.68	2.84	81.84	2.18
0.39	-9.02	1.04	-1.04	2.84	41.36	1.88
4.37	2.12	2.35	2.53	2.84	95.02	0.91
2.56	-1.01	1.36	1.29	2.85	75.75	1.62
4.77	2.4	-1.34	2.66	2.85	96.82	4.94
4.8	2.34	-1.06	2.63	2.85	96.45	4.46
4.86	-14.57	-2.65	-2.09	2.85	26.53	8.38
7.67	-3.97	-0.44	0.31	2.85	60.89	3.16
2.47	0.1	0.93	1.71	2.85	82.09	1.86
2.49	-0.59	1.23	1.44	2.85	78.13	1.63
2.8	-12.83	1.11	-1.79	2.85	30.55	1.89
5.18	-6.53	0.98	-0.41	2.85	50.13	1.84
3.97	2.44	-1.6	2.67	2.85	97.16	5.51
6.12	-4.74	0.17	0.07	2.86	57.38	2.32
6.58	-6.84	0.02	-0.5	2.86	48.94	2.94
3.5	-10.26	0.42	-1.28	2.86	37.47	2.19
2.07	-7.52	1.07	-0.69	2.86	46.43	1.86
2.52	0.15	0.57	1.73	2.86	82.4	2.09
2.68	-12.82	0.94	-1.78	2.86	30.55	1.97
1.03	-7.75	1.34	-0.74	2.86	45.61	1.8
1.54	-7.9	0.76	-0.77	2.86	45.09	2.22
-0.1	-12.42	0.57	-1.7	2.87	31.53	2.43
6.72	-14.18	-0.57	-2.02	2.87	27.33	3.42
13.75	-13.67	0.11	-1.92	2.87	28.5	2.91
12.37	-19.3	-2.2	-2.71	2.87	17.82	7.38
15.35	-11.81	-2.58	-1.6	2.87	33.12	8.25
0.98	-2.89	0.68	0.67	2.87	65.85	2.24
1.97	-2.52	2.17	0.79	2.87	67.69	1.12

0.07	-8.68	1.19	-0.94	2.87	42.4	1.74
5.52	-6.28	0.75	-0.32	2.87	51.03	2.03
4.75	-0.03	2.4	1.67	2.87	81.26	0.97
5.93	-0.98	-1.2	1.33	2.87	75.84	4.53
3.81	2.24	-1.54	2.6	2.87	95.68	5.47
4.23	-13.95	-2.54	-2	2.88	27.83	7.2
8.5	-15.55	-2.35	-2.21	2.88	24.4	7.22
5.51	-5.63	-0.34	-0.16	2.88	53.57	3.16
5.01	-5.7	-0.17	-0.18	2.88	53.31	3.1
5.04	-5.59	0.01	-0.15	2.88	53.74	2.78
14.45	-10.62	-2.94	-1.36	2.88	36.39	8.87
7.69	-3.89	0.08	0.36	2.88	61.09	2.84
0.18	-8.82	1.15	-0.97	2.88	41.93	1.77
0.27	-8.9	0.85	-0.99	2.88	41.63	1.99
0.38	-9	0.74	-1.01	2.88	41.33	2.09
2.67	-0.33	2.63	1.56	2.88	79.44	0.87
7.65	-7.79	-1.1	-0.7	2.88	45.41	4.3
5.27	2.36	1.25	2.66	2.88	96.34	1.39
4.5	2.24	-0.39	2.6	2.89	95.46	3.35
5.01	-5.26	-0.62	-0.05	2.89	55.05	3.92
1.98	-9.84	0.23	-1.18	2.89	38.68	2.58
11.89	-9.41	-2.93	-1.11	2.89	39.99	8.87
1.91	-1.62	-0.05	1.1	2.89	72.22	3.09
3.2	-2.86	3.88	0.69	2.89	65.89	0.45
-1.09	-13.33	-0.33	-3.14	2.9	33.84	1.24
2.23	-6.26	2.34	-0.33	2.9	51.01	1.07
0.19	-8.58	-0.02	-0.9	2.9	42.64	2.98
4.44	-0.01	1.76	1.7	2.9	81.22	1.34
4.45	0.08	1.55	1.73	2.9	81.69	1.47
4.57	0.11	1.55	1.74	2.9	81.88	1.49
3.79	2.19	-1.3	2.59	2.9	95.04	4.99
3.85	2.34	-1.12	2.65	2.9	96.12	4.66
4.92	2.35	-0.39	2.66	2.91	96.07	3.46
4.95	2.35	0.13	2.67	2.91	96.15	2.87
4.97	2.33	-0.17	2.66	2.91	95.98	3.21
12.43	-8.74	-2.41	-0.94	2.91	42.08	7.9
4.36	0.91	-2.02	2.06	2.91	86.69	6.58
4.42	0.98	-1.55	2.09	2.91	87.14	5.31
5.77	-5.28	-0.36	-0.04	2.91	54.92	3.34
2.71	0.64	2.28	1.96	2.91	85.05	1.11
3.87	2.43	-1.35	2.7	2.91	96.63	5.07
4.45	2.8	-0.81	2.87	2.91	99.21	4.2
6.61	-14.28	-0.19	-2.01	2.92	27.03	3.16
2.75	-1.07	1.45	1.31	2.92	75.05	1.64

4.83	2.38	-0.96	2.68	2.92	96.21	4.34
6.78	-6.75	-2.08	-0.44	2.92	49.07	7.18
1.92	-7.15	1.98	-0.55	2.92	47.56	1.24
1.71	-3.7	0.55	0.44	2.92	61.8	2.28
2.49	-3.47	1.34	0.5	2.92	62.84	1.6
1.73	-2.47	1.75	0.84	2.92	67.7	1.47
1.88	-0.96	1.83	1.36	2.92	75.64	1.38
4.48	2.77	-0.53	2.85	2.92	98.99	3.73
1.41	-5.6	1.64	-0.13	2.93	53.52	1.51
0.74	-7.54	2.68	-0.62	2.93	46.15	0.89
8	-3.54	-0.63	0.5	2.93	62.51	3.91
1.73	-8.39	1.41	-0.84	2.94	43.15	1.65
1.14	-5.34	1.5	-0.05	2.94	54.52	1.54
4.94	2.37	-0.23	2.69	2.94	96.07	3.26
6.8	-7.09	0.76	-0.51	2.94	47.73	2.28
2.91	-12.81	1.14	-1.73	2.94	30.41	1.93
5.61	2.56	2.9	2.77	2.94	97.3	0.73
4.33	-1.58	-0.06	1.14	2.95	72.18	3.23
3.35	-0.13	0.77	1.68	2.95	80.18	2.34
5.02	-5.28	-0.29	-0.02	2.95	54.72	3.64
2.37	-12.82	0.97	-1.73	2.95	30.35	1.83
1.13	-2.99	0.57	0.68	2.95	65.01	2.44
3.31	-2.19	0.88	0.95	2.95	68.99	2.16
3.01	-12.9	1.07	-1.74	2.95	30.17	2
8.11	-14.19	-1.33	-1.96	2.95	27.16	4.91
4.86	0.02	2.39	1.74	2.95	81.07	0.98
4.91	-8.95	2.18	-0.94	2.96	41.26	0.88
5.01	-5.6	0.25	-0.11	2.96	53.41	2.67
6.68	-7.33	0.39	-0.56	2.96	46.8	2.58
2.63	-5.8	3.18	-0.17	2.96	52.6	0.54
2.15	-10.03	0.62	-1.18	2.96	37.91	2.38
13.51	-8.9	-2.61	-0.95	2.96	41.43	7.66
1.9	-1.59	0.47	1.14	2.96	72.02	2.61
1.07	-2.95	0.47	0.69	2.96	65.15	2.49
3.06	-2.23	0.73	0.93	2.96	68.74	2.23
7.13	-1.14	-1.72	1.32	2.96	74.46	5.67
0.1	-14.12	0.22	-1.99	2.97	27.28	2.86
12.92	-2.15	0.18	0.96	2.97	69.08	2.1
5.01	2.38	-0.12	2.7	2.97	95.91	3.25
1.76	1.03	-0.08	2.14	2.97	87.07	3.18
3.66	-9.6	0.04	-1.09	2.97	39.2	2.54
5.8	-8.22	-2.26	-0.77	2.98	43.6	6.94
6.14	-8.48	-2.11	-0.83	2.98	42.71	7.27
5	2.27	0	2.67	2.98	95.06	3.2

4.32	0.97	-1.55	2.12	2.98	86.66	5.54
1.79	1	-0.04	2.13	2.98	86.85	3.19
-0.59	-9.84	0.98	-1.17	2.99	38.39	2.11
9.3	-7.51	0.73	-0.59	2.99	46.05	2.37
5.03	2.36	-0.15	2.71	2.99	95.61	3.29
5.01	-5.49	0.35	-0.06	2.99	53.73	2.63
2.33	-6.32	2.19	-0.29	2.99	50.45	1.22
4.4	0.93	-1.62	2.11	2.99	86.35	5.9
4.46	0.94	-1.49	2.12	2.99	86.41	5.58
1.14	-3.11	0.89	0.66	2.99	64.23	2.35
7.89	-3.62	0.3	0.51	2.99	61.85	2.85
3.3	-2.93	3.58	0.73	2.99	65.13	0.53
4.42	2.89	-0.86	2.94	2.99	99.27	4.4
1.06	-11.63	1.7	-1.62	3	33.86	1.4
-3.99	-18.06	-6.4	-7.51	3.01	33.92	1.3
0.83	-7.47	2.84	-0.56	3.01	46.14	0.93
6.49	-4.04	0.65	0.37	3.01	59.88	2.24
4.45	0.95	-1.35	2.13	3.01	86.39	5.3
2.12	-3.1	1.93	0.68	3.01	64.21	1.35
1.92	-1.16	1.96	1.34	3.01	74.13	1.34
1.97	-0.85	2.02	1.44	3.01	75.79	1.3
1.7	-7.84	1.17	-0.67	3.01	44.81	2.34
2.68	0.66	2.47	2.02	3.01	84.55	1.06
4.24	2.71	-1.13	2.87	3.01	97.87	5.13
0.77	-15.78	-0.01	-2.17	3.02	23.71	3.47
10.14	3.02	1.02	3.02	3.02	100	1.69
0.68	-7.51	3.05	-0.56	3.02	45.93	0.74
1.19	-11.54	1.63	-1.44	3.02	33.47	1.53
1.29	-11.52	1.44	-1.45	3.02	33.53	1.68
3.11	-12.73	1.04	-1.66	3.02	30.42	2.09
4.57	-3.51	1.59	0.56	3.02	62.25	1.61
9.11	-1.85	-1.48	1.11	3.02	70.37	5.71
8.84	-1.6	-1.75	1.19	3.02	71.67	6.17
2.66	-11.4	-1.88	-1.42	3.03	33.84	6.15
3.95	-5.92	2.33	-0.15	3.03	51.84	1.09
13.21	-18.91	-1.96	-2.56	3.03	18.2	7.32
16.19	-12.83	-2.11	-1.69	3.03	30.17	8.53
2.34	-0.28	0.48	1.68	3.03	78.85	2.74
8.89	-14.39	-0.98	-1.94	3.03	26.58	4.59
8.4	-1.51	-1.54	1.23	3.03	72.12	6.01
0.01	-12.36	0.72	-1.58	3.04	31.31	2.41
1.85	-11.33	-0.04	-1.42	3.04	33.99	3.49
2.64	-4.27	0.49	0.33	3.04	58.7	2.65
4.75	2.12	0.36	2.63	3.04	93.69	2.82

5.69	-5.79	0.02	-0.11	3.04	52.33	3.01
16.86	-12.22	-2.07	-1.57	3.04	31.67	8.23
12.96	-8.32	-2.36	-0.76	3.04	43.08	7.55
1.9	-1.58	0.8	1.2	3.04	71.7	2.34
6.3	-6.87	0.69	-0.38	3.04	48.22	2.49
6.02	-6.54	1.02	-0.29	3.04	49.44	2.06
5.78	-6.4	1.2	-0.26	3.04	49.99	1.78
7.48	-12.84	0.87	-1.64	3.04	30.12	1.93
8.22	-8.15	-0.77	-0.69	3.04	43.67	4.29
3.37	0.9	3.06	2.14	3.04	85.87	0.8
4.03	-5.66	0.1	-0.09	3.05	52.85	3.31
14.33	-13.39	0.32	-1.76	3.05	28.8	3.03
19.24	-10.23	-0.05	-1.18	3.05	37.08	2.92
-0.3	-13.29	0.01	-1.8	3.06	28.98	2.98
1.57	-6.5	0.12	-0.29	3.06	49.5	3.17
8.39	-15.72	-2.39	-2.12	3.06	23.77	6.95
7.84	-12.81	1.06	-1.65	3.06	30.15	1.97
2.23	-3.34	1.31	0.62	3.06	62.82	1.76
3.47	-3.24	3.37	0.68	3.06	63.32	0.67
1.86	1.22	-0.01	2.28	3.06	87.71	3.19
1.92	1.19	-0.03	2.26	3.06	87.49	3.25
8.34	-7.78	-1.02	-0.59	3.06	44.85	4.78
3.04	0.91	3.17	2.15	3.06	85.82	0.72
2.74	1.55	3.33	2.41	3.06	89.84	0.68
6.75	-13.31	-0.44	-1.75	3.07	28.92	3.43
3.01	-8.36	1.99	-0.77	3.07	42.88	1.1
7.6	2.33	-1.37	2.74	3.07	94.89	5.74
7.69	2.27	-1.73	2.72	3.07	94.44	6.66
3.47	-4.78	2.06	0.19	3.07	56.37	1.03
5.94	-5.42	-0.61	0.01	3.07	53.74	3.89
4.43	2.97	-0.63	3.03	3.07	99.24	4.11
3.88	0.91	0.65	2.16	3.08	85.68	2.28
1.85	-2.72	1.9	0.84	3.08	65.73	1.45
1.82	-2.35	1.98	0.97	3.08	67.57	1.36
1.91	-1.07	2.28	1.42	3.08	74.21	1.13
2.32	-0.92	2.22	1.47	3.08	75.01	1.17
3.41	0.99	3.09	2.19	3.08	86.16	0.77
6.16	-7.96	-1.66	-0.64	3.09	44.15	6.51
3.04	-1.11	1.61	1.39	3.09	73.93	1.73
1.73	-2.56	1.21	0.89	3.09	66.46	1.98
4.62	2.16	-0.24	2.68	3.09	93.6	3.59
5.23	-15.8	-2.68	-2.12	3.09	23.56	9.71
2.15	-7.45	1	-0.52	3.09	45.92	2.22
7.97	-3.38	-0.02	0.64	3.09	62.51	3.16

2.8	-3.29	1.59	0.66	3.09	62.94	1.68
3.79	-4.49	1.89	0.3	3.09	57.54	1.59
4.28	2.82	-1.07	2.97	3.09	98.07	5.17
1.88	-8.39	1.15	-0.74	3.1	42.67	2.1
1.52	-5.84	1.37	-0.1	3.1	51.91	1.82
1.6	-5.92	1.16	-0.12	3.1	51.59	2.17
6.9	-7.47	1.13	-0.51	3.1	45.83	2.06
1.8	0.3	2.01	1.92	3.1	81.85	1.58
3.28	-12.59	1.15	-1.59	3.1	30.61	2.04
3.39	-12.73	1.56	-1.62	3.1	30.27	1.79
9.08	-14.32	-0.51	-1.89	3.1	26.58	3.95
8.38	-1.28	-1.09	1.35	3.1	72.99	5.08
18.46	-10.55	2.69	-1.21	3.11	36	0.83
5.37	3.03	-3.35	3.07	3.11	99.45	12.4
6.04	-5.95	0.54	-0.11	3.11	51.45	2.6
14.07	-13.37	0.11	-1.72	3.11	28.71	2.88
3.56	-2.08	1.34	1.08	3.11	68.78	1.84
3.79	-2.08	1.16	1.08	3.11	68.78	2.06
2.49	-2.86	2.62	0.81	3.11	64.93	0.94
1.98	-3.89	0.85	0.47	3.11	60.12	2.41
3.63	0.96	3.33	2.2	3.11	85.8	0.74
2.24	-3.3	1.4	0.67	3.11	62.8	1.7
2.45	-3.2	1.13	0.71	3.11	63.3	2.14
4.64	-3.58	1.54	0.59	3.11	61.53	1.74
-0.56	-9.99	1.4	-1.12	3.12	37.57	1.88
7.16	-8.59	5.6	1.02	3.12	33.94	0.95
2.76	-4.62	0.73	0.27	3.12	56.84	2.44
3.46	-0.51	0.48	1.64	3.12	77.04	2.76
4.85	2.18	0.47	2.71	3.12	93.58	2.66
4.54	2.37	0.07	2.79	3.12	94.8	2.99
5.14	2.39	0.36	2.79	3.12	94.97	2.86
2.21	-10.02	0.58	-1.07	3.12	37.51	2.6
2.42	-10.25	0.8	-1.12	3.12	36.82	2.41
1.42	-13.13	1.1	-1.65	3.12	29.26	2.1
1.53	-13.08	1.29	-1.64	3.12	29.36	1.96
1.09	-7.77	1.55	-0.58	3.12	44.71	1.84
3.12	-7.09	0.08	-0.38	3.12	47.12	3.25
8.42	-8.06	-0.69	-0.61	3.12	43.71	4.3
7.99	-7.53	-0.55	-0.49	3.12	45.57	3.83
7.66	-0.84	-1.53	1.52	3.12	75.22	5.46
0.11	-12.18	0.55	-1.5	3.13	31.56	2.7
2.12	-11.62	-0.01	-1.42	3.13	33	3.56
0.18	-14.09	0.22	-1.88	3.13	27.02	3.1
5.05	-4.91	0.4	0.19	3.13	55.61	2.8

5.01	-5.28	0.13	0.08	3.13	54.07	3
2.28	-10.05	0.58	-1.08	3.13	37.39	2.6
2.35	-10.01	0.82	-1.07	3.13	37.51	2.32
1.86	-1.56	0.99	1.26	3.13	71.34	2.23
5.02	-3.2	2.72	0.72	3.13	63.17	0.92
-0.27	-13.28	0.43	-1.75	3.14	28.87	2.63
4.57	2.28	-0.17	2.76	3.14	94.12	3.58
5.24	2.43	0.63	2.82	3.14	95.14	2.6
5.05	-4.44	-0.47	0.34	3.14	57.55	4.04
7.07	-6.78	-1.72	-0.31	3.14	48.2	5.7
2.52	-3.36	1.38	0.67	3.14	62.44	1.76
6.71	-7.08	0.75	-0.37	3.14	47.1	2.43
3.21	0.98	3.37	2.23	3.14	85.76	0.66
6.04	-8.06	-1.75	-0.63	3.15	43.63	6.54
6.25	-7.88	-1.69	-0.59	3.15	44.25	6.57
1.67	-6.48	-0.06	-0.24	3.15	49.25	3.49
1.77	-2.61	1.97	0.92	3.15	65.94	1.35
1.97	1.18	-0.21	2.3	3.15	86.93	3.68
1.99	1.03	-0.23	2.24	3.15	85.98	3.29
2.41	-0.96	2.16	1.49	3.15	74.42	1.34
0.31	-3.96	0.53	0.47	3.15	59.62	2.4
2.59	0.62	2.76	2.09	3.15	83.45	0.96
4.42	3.02	-0.44	3.1	3.15	99.03	3.97
1.96	-15.88	-0.59	-2.09	3.16	23.28	4.52
2.47	-6.31	1.96	-0.19	3.16	49.87	1.45
2.76	-3.33	1.85	0.69	3.16	62.44	1.52
5.19	-1.53	1.97	1.29	3.16	71.34	1.28
4.51	2.99	-0.54	3.08	3.16	98.78	4.36
4.41	3.01	-0.71	3.09	3.16	98.95	4.47
6.57	-7.31	-1.8	-0.44	3.17	46.16	7.22
3	-10.65	2.06	-1.17	3.17	35.55	1.5
4.37	-1.51	0.64	1.3	3.17	71.4	2.14
1.93	-2.73	1.69	0.89	3.17	65.28	1.7
0.87	-7.57	2.89	-0.48	3.17	45.22	1.01
13.04	-0.6	-0.59	1.63	3.17	76.28	3.56
5.03	-5.03	-0.42	0.18	3.17	54.94	4.05
7	-6.65	-1.71	-0.26	3.17	48.56	6.98
6.95	-7.28	0.71	-0.42	3.17	46.28	2.54
1.1	-3.78	2.8	0.54	3.17	60.33	0.93
1.15	-3.72	2.97	0.57	3.17	60.62	0.78
2.69	0.64	1.8	2.11	3.17	83.46	1.56
-15.26	-31.31	-20.02	-18.38	3.18	27.51	1.14
4.27	-5.67	1.02	0.01	3.18	52.27	1.92
2.45	-0.26	0.3	1.76	3.18	78.19	3.17

1.99	1.13	-0.14	2.3	3.18	86.44	3.68
9.33	-14	-0.51	-1.79	3.18	27.15	4.03
0.81	-3.88	1.7	0.52	3.18	59.86	2.07
2.31	-0.73	1.72	1.59	3.18	75.51	1.54
5.26	2.09	-0.16	2.71	3.19	92.47	3.6
4.72	-3.51	2.17	0.66	3.19	61.49	1.31
4.03	-2.22	0.83	1.08	3.19	67.66	2.45
2.77	-2.71	2.65	0.91	3.19	65.25	0.97
1.74	0.48	1.76	2.05	3.19	82.43	1.53
1.92	-13.3	1.69	-1.64	3.19	28.72	1.7
2.69	-13.06	1.13	-1.6	3.19	29.27	2.17
1.44	-7.69	1.52	-0.52	3.19	44.75	1.78
2.13	-1.22	1.46	1.41	3.19	72.85	1.86
4.75	-13.42	-1.99	-1.71	3.2	28.41	6.77
9.99	-4.18	-1.07	0.45	3.2	58.47	5.12
8.7	-15.79	-1.67	-2.04	3.2	23.38	6.62
5.22	-9.12	2.58	-0.84	3.2	40.04	0.93
2.86	-4.8	0.71	0.27	3.2	55.77	2.58
3.59	-0.28	0.95	1.76	3.2	77.92	2.44
6.3	-5.81	-0.05	-0.02	3.2	51.67	3.37
4.67	-5.56	2.38	0.05	3.2	52.65	1.12
2.2	-7.65	1.05	-0.51	3.2	44.87	2.24
17.68	-17.86	-1.87	-2.33	3.2	19.67	8.51
1.88	-1.49	1.1	1.32	3.2	71.36	2.13
0.5	-8.74	1.54	-0.75	3.2	41.23	1.78
0.55	-8.78	1.59	-0.75	3.2	41.11	1.78
-0.57	-9.89	0.99	-1.04	3.21	37.64	2.27
8.62	-15.01	-2.13	-1.93	3.21	24.93	6.96
5.02	2.1	-0.19	2.72	3.21	92.42	3.75
5	2.17	0.08	2.75	3.21	92.93	3.22
15.9	-11.62	-2.16	-1.36	3.21	32.82	7.64
5.7	-2.03	0.83	1.15	3.21	68.52	2.24
1.89	-11.15	-0.02	-1.28	3.22	34.04	3.5
1.85	-6.49	0.41	-0.2	3.22	49	3.12
5.11	-7.39	7.03	2.08	3.22	33.95	1.5
2.93	-4.42	-0.06	0.39	3.22	57.31	3.71
7.83	-13.94	0.49	-1.72	3.22	27.21	2.82
2.26	-3.75	-0.06	0.59	3.22	60.29	3.5
4.37	2.98	-0.31	3.11	3.22	98.28	3.99
-0.92	-14.3	2.55	-2.17	3.23	27.49	0.77
3.91	-10.05	0.67	-1.02	3.23	37.13	2.29
2.59	-6.4	2.35	-0.17	3.23	49.3	1.42
1.93	-2.03	1.4	1.16	3.23	68.44	2.11
2.02	1.12	-0.18	2.33	3.23	86.03	3.63

3.28	-7.28	0.22	-0.36	3.23	46.08	3.29
2.63	0.79	3.16	2.2	3.23	84.03	0.8
6.47	-7.49	-1.79	-0.44	3.24	45.32	6.93
4.05	-12.59	5.57	-0.65	3.24	27.48	0.54
-1.5	-13.42	-0.64	-3.26	3.24	33.97	1.25
-1.84	-14.96	-1.92	-4.68	3.24	34.03	0.98
2.93	-4.87	0.88	0.27	3.24	55.29	2.46
2.9	-1.83	-0.14	1.22	3.24	69.36	4.68
3.9	-3.08	3.36	0.82	3.24	63.28	0.73
4.14	1	0.59	2.29	3.24	85.26	2.55
6.82	-0.57	2.74	1.68	3.24	76.1	0.92
4.46	3.07	-0.42	3.16	3.24	98.81	4.17
4.48	3.07	-0.24	3.17	3.24	98.83	3.78
6.53	-14.42	0.35	-1.82	3.25	26.11	2.82
7.04	-7.35	0.23	-0.39	3.25	45.76	3.26
3.18	-5.92	3.64	-0.03	3.25	51.07	0.64
4.18	-3.59	2.72	0.67	3.25	60.89	1.08
6.2	-5.16	-0.35	0.19	3.25	54.09	3.84
2.72	-3.5	1.62	0.69	3.25	61.31	1.8
4.34	2.99	-0.44	3.14	3.25	98.18	4.21
4.36	2.95	-0.4	3.12	3.25	97.92	4.19
4.35	2.93	-0.54	3.11	3.25	97.75	4.49
4.52	3.1	-0.37	3.18	3.25	98.95	4.03
4.55	3.06	-0.43	3.17	3.25	98.72	4.2
4.54	3.05	-0.63	3.16	3.25	98.6	4.56
-1.03	-14.35	1.31	-2.2	3.26	27.46	1.44
1.95	-2.76	1.31	0.93	3.26	64.71	2.01
1.95	-2.74	1.62	0.93	3.26	64.82	1.81
14.05	-1.03	-0.86	1.52	3.26	73.48	4.79
2.3	-7.79	1.66	-0.5	3.26	44.18	1.89
2.82	0.72	1.12	2.19	3.26	83.4	2.19
2.86	0.72	1.09	2.19	3.26	83.4	2.23
4.81	-0.54	1.54	1.71	3.26	76.14	1.66
2.62	-3.33	1.4	0.75	3.26	62.04	1.97
6.28	-14.24	0.29	-1.77	3.27	26.45	3.21
5.3	-1.46	0.2	1.37	3.27	71.15	3.38
2.98	-4.87	1.34	0.29	3.27	55.18	2.01
14.45	-12.87	0.02	-1.53	3.27	29.56	3.24
1.62	-5.98	1.36	-0.04	3.27	50.76	1.5
4.27	-2.27	1	1.11	3.27	67.03	2.38
8.13	-2.96	-0.08	0.88	3.27	63.7	3.29
2.7	-3.29	0.75	0.76	3.27	62.16	2.29
2.26	-0.76	1.95	1.63	3.27	74.92	1.46
2.12	-13.41	1.71	-1.6	3.27	28.3	1.73

7.73	-13.45	1.14	-1.61	3.27	28.2	2.23
1.25	-7.64	1.33	-0.46	3.27	44.68	2.12
8.53	-8.12	-0.15	-0.54	3.27	43.05	3.53
2.64	0.83	3.28	2.25	3.27	84.01	0.76
2.73	0.71	1.2	2.19	3.27	83.37	2.05
2.46	-0.87	1.61	1.59	3.27	74.28	1.83
9.34	-3.1	-0.67	0.84	3.27	63.03	5.11
6.86	-6.3	-0.3	-0.11	3.28	49.48	3.55
10.37	-4.02	-1.13	0.53	3.28	58.8	5.61
2.48	-13.06	1.19	-1.54	3.28	29.1	2.1
1.21	-7.7	1.08	-0.47	3.28	44.44	2.24
0.2	-4.17	0.47	0.5	3.28	58.15	3.06
5.28	-0.55	1.81	1.72	3.28	75.98	1.54
4.8	-3.19	1.48	0.81	3.28	62.55	1.76
4.14	-5.79	0.53	0.02	3.29	51.39	2.96
5.22	-13.94	-1.94	-1.75	3.29	27.07	7.57
0.57	-19.85	-4.75	-7.16	3.29	27.42	1.12
13.87	-18.14	-2.07	-2.3	3.29	19.09	7.54
17.5	-13.83	-1.86	-1.7	3.29	27.31	8.1
5.18	-3.19	2.7	0.82	3.29	62.53	1
7.73	-12.96	1.24	-1.52	3.29	29.31	1.89
0.26	-4.03	0.85	0.54	3.29	58.72	2.53
7.19	-8.31	-1.62	-0.6	3.3	42.32	6.93
6.81	-8.82	-1.53	-0.72	3.3	40.68	7.08
-0.48	-10.02	0.71	-1.01	3.3	37.02	2.77
5.3	2.59	0.59	2.99	3.3	95.11	2.83
2.25	-7.55	0.28	-0.44	3.3	44.92	3.76
6.47	-2.63	0.42	1.01	3.3	65.14	3.11
3.44	-3.17	3.04	0.83	3.3	62.56	0.92
3.46	-2.99	2.58	0.89	3.3	63.4	1.16
0.31	-3.89	0.75	0.59	3.3	59.32	2.45
2.95	1.6	2.81	2.56	3.3	88.62	0.94
4.55	3.12	0.12	3.22	3.3	98.74	3.36
2.25	-11.58	0.2	-1.31	3.31	32.71	3.63
-0.47	-10.14	-0.04	-1.04	3.31	36.66	3.72
-0.96	-14.33	1.89	-2.17	3.31	27.4	1.09
6.31	-8.16	9.23	3.2	3.31	27.41	1.45
0.89	-15.89	0.3	-2	3.31	23.02	3.37
5.29	2.58	0.53	2.98	3.31	94.94	2.96
7.98	2.31	-1.2	2.87	3.31	93.18	6.13
3.46	-2.99	2.85	0.89	3.31	63.4	0.99
3.71	-13.18	1.99	-1.56	3.31	28.73	1.56
2.09	-2.19	2.78	1.16	3.31	67.24	1.1
2.95	1.54	2.22	2.54	3.31	88.18	1.45

9.54	-8.01	-0.01	-0.51	3.32	43.27	3.24
7.16	-7.4	0.29	-0.36	3.32	45.36	3.49
3.31	-3.48	1.28	0.74	3.32	61.07	2.08
6.68	-2.47	-0.16	1.07	3.32	65.81	3.85
8.31	-3.43	0.39	0.76	3.32	61.27	3.11
3.3	-3.05	2.04	0.88	3.32	63.04	1.5
4.46	1.02	0.56	2.35	3.32	84.89	2.75
2.55	-3.41	1.33	0.76	3.32	61.38	2.03
4.29	2.94	-0.05	3.15	3.32	97.34	3.8
4.33	2.84	-0.37	3.11	3.32	96.65	4.35
9.21	-10.31	4.19	-0.47	3.33	34.04	0.83
2.05	-8.37	1.65	-0.59	3.33	42.05	1.81
2.25	-11.6	1.42	-1.26	3.33	32.59	2.62
1.84	-3.92	2.85	0.6	3.33	59.04	1.05
1.95	-2.02	1.33	1.23	3.33	67.97	2.25
4.34	2.97	-0.56	3.18	3.33	97.51	4.59
4.47	3.15	-0.03	3.25	3.33	98.78	3.64
5.67	-15.74	-2.25	-1.96	3.34	23.27	9.52
2.27	-7.72	0.86	-0.43	3.34	44.19	2.4
1.91	0.34	1.59	2.08	3.34	80.7	2.14
2.98	-2.88	1.59	0.94	3.34	63.8	1.82
2.96	-2.92	1.45	0.92	3.34	63.58	1.94
2.87	-3.06	1.17	0.88	3.34	62.93	2.03
4.9	-2.05	3.54	1.23	3.34	67.8	0.69
3.26	-1.08	2.27	1.55	3.34	72.8	1.46
1.31	-7.52	1.8	-0.39	3.34	44.87	2.01
3.89	-4.67	2.17	0.41	3.34	55.77	1.56
3.04	0.84	1.26	2.28	3.34	83.7	2.16
5.4	2.44	2.11	2.94	3.34	93.87	1.46
3.49	-3.55	1.88	0.73	3.34	60.68	1.65
6.89	-7.68	-1.94	-0.42	3.35	44.31	7.61
4.24	-1.62	4.53	1.38	3.35	69.95	0.31
3.33	0.87	0.85	2.3	3.35	83.78	2.58
3.34	0.9	0.87	2.31	3.35	84.01	2.56
2.2	-0.95	1.8	1.6	3.35	73.42	1.64
3.47	-3.58	1.07	0.73	3.35	60.48	2.45
9.61	-1.39	-0.82	1.46	3.35	71.14	4.77
4.47	3.05	0.3	3.21	3.35	97.92	3.33
-7.76	-25.35	-12.32	-12.39	3.36	27.38	1.13
1.47	-5.6	1.08	0.12	3.36	51.9	2.26
1.49	-5.69	1.41	0.09	3.36	51.55	1.91
3.07	-4.85	1.37	0.34	3.36	54.97	2.08
3.71	-0.35	1.11	1.83	3.36	76.66	2.45
5.37	2.6	1.4	3.02	3.36	94.73	1.99

3.44	-3.35	2.46	0.81	3.36	61.47	1.23
2.53	-0.2	0.68	1.89	3.36	77.52	2.88
1.29	-3.03	1.19	0.9	3.36	62.96	2.51
1.85	-2.06	1.23	1.23	3.36	67.67	2.29
6.25	-2.36	1.4	1.13	3.36	66.16	2.03
7.6	-3.18	-0.17	0.86	3.36	62.29	4.2
2.28	-13.35	1.58	-1.54	3.36	28.24	1.86
3.29	0.99	0.61	2.35	3.36	84.48	2.9
3.36	1	0.56	2.35	3.36	84.51	3.11
2.55	-0.94	1.57	1.61	3.36	73.42	1.82
-0.44	-10.25	0.9	-1.02	3.37	36.18	2.49
3.37	-11.28	-1.86	-1.19	3.37	33.36	7.01
5.77	2.31	0.45	2.9	3.37	92.83	2.93
17.68	-16.28	-1.81	-2.02	3.37	22.19	7.78
20.23	-10.82	0.72	-1.1	3.37	34.58	2.54
5.97	-2.14	1.21	1.21	3.37	67.19	2.07
5.32	-3.08	2.69	0.9	3.37	62.69	1.05
4.89	-2.55	0.22	1.08	3.37	65.21	3.43
5.05	-2.68	0.06	1.03	3.37	64.57	3.68
7.71	-3.19	0.19	0.87	3.37	62.15	3.73
0.72	-8.76	1.31	-0.65	3.37	40.66	2.18
1.15	-7.74	1.17	-0.42	3.37	44.04	2.16
3.46	-7.44	0.11	-0.32	3.37	45.05	3.59
-0.53	-10.18	0.76	-1	3.38	36.37	2.77
4.43	-13.49	3.7	-1.38	3.38	27.32	0.77
7.29	-8.23	3.54	-0.5	3.38	42.36	0.81
6.78	-7.78	1.09	-0.4	3.38	43.87	2.09
3.45	-3.03	3.34	0.92	3.38	62.86	0.81
3.46	-2.9	2.92	0.97	3.38	63.45	1
3.45	-2.55	3.47	1.08	3.38	65.13	0.72
2.39	-3.66	-0.09	0.71	3.38	59.95	3.7
3.57	-7.53	0.49	-0.33	3.38	44.73	3.15
4.33	2.84	-0.19	3.15	3.38	96.27	4.1
4.49	3.13	0.45	3.27	3.38	98.22	3.13
4.68	3.1	-0.25	3.25	3.38	98.02	4.06
2.03	-6.46	0.44	-0.09	3.39	48.51	3.23
3.43	-3.37	2.83	0.82	3.39	61.25	1.04
3.48	-3.36	2.23	0.82	3.39	61.28	1.45
2.9	-3.15	2.97	0.89	3.39	62.27	0.97
6.73	-3.04	-0.02	0.92	3.39	62.75	3.93
3.94	-9.69	0.07	-0.86	3.39	37.78	2.96
8.76	-8.89	-0.47	-0.64	3.39	40.21	4.54
4.48	3.06	0.05	3.24	3.39	97.7	3.68
4.49	3.19	0.71	3.3	3.39	98.62	2.74

4.57	3.06	0.55	3.24	3.39	97.73	3.2
5.15	-14.53	0.75	-1.74	3.4	25.6	2.66
-0.45	-10.1	0.16	-0.97	3.4	36.55	3.38
1.88	-3.95	3.07	0.62	3.4	58.63	1
3.45	-3.59	2.25	0.75	3.4	60.24	1.41
3.34	-3.28	2.05	0.85	3.4	61.6	1.5
3.26	-3.17	2.39	0.89	3.4	62.1	1.27
7.69	-13.88	0.3	-1.6	3.4	26.99	3.54
0.21	-4.17	0.29	0.56	3.4	57.69	3.29
4.82	1.44	1.13	2.56	3.4	86.97	2.19
3.04	0.76	1.14	2.29	3.4	82.85	2.31
5.45	2.3	2.81	2.92	3.4	92.53	1.16
3.48	-3.63	1.76	0.74	3.4	60.07	1.76
4.54	3.1	-0.13	3.27	3.4	97.88	4.11
4.7	-11.77	5.01	0.14	3.41	27.31	1.23
7.05	-11.2	5.82	0.63	3.41	27.31	1.06
7.29	-6.32	-1.48	-0.03	3.41	48.98	6.94
4.04	-4.45	2.55	0.48	3.41	56.41	1.06
14.94	-18.74	-1.94	-2.3	3.41	17.98	8.33
3.46	-3.59	2.08	0.77	3.41	60.19	1.49
3.45	-3.56	2.24	0.77	3.41	60.3	1.42
6.84	-2.49	-0.1	1.11	3.41	65.3	3.83
3.45	-2.5	3.6	1.11	3.41	65.25	0.69
1.69	0.37	2.9	2.14	3.41	80.5	1.07
3.46	-3.6	1.97	0.76	3.41	60.13	1.6
4.44	2.99	-0.15	3.22	3.41	97.11	4.01
-0.4	-10.34	1.24	-1.02	3.42	35.83	2.42
4.64	-12.4	5	-0.39	3.42	27.21	0.87
2.25	-8.45	2.14	-0.56	3.42	41.53	1.59
0.75	-8.99	2.63	-0.67	3.42	39.8	1.26
8.24	-8.2	2.32	-0.47	3.42	42.34	1.4
1.32	-7.53	3.25	-0.32	3.42	44.61	0.91
1.22	-7.52	3.32	-0.32	3.42	44.64	0.83
2.02	-11.89	2.34	-1.26	3.42	31.66	1.57
3.45	-3.51	2.88	0.79	3.42	60.53	1.04
2.69	-0.28	1.38	1.9	3.42	76.7	2.44
5.56	-2.57	3.21	1.1	3.42	64.86	0.86
3	-2.72	1.75	1.04	3.42	64.16	1.75
9.8	-13.91	-0.29	-1.62	3.42	26.86	3.9
3.92	-2.51	1.4	1.1	3.42	65.17	2.14
3.5	-3.69	1.3	0.74	3.42	59.68	2.24
5.74	-1.37	0.44	1.5	3.43	70.85	3.37
9.79	-8.59	0.28	-0.58	3.43	41.06	3.4
6.61	-5.53	0.74	0.2	3.43	51.91	2.65

6.01	-3.29	2.8	0.88	3.43	61.43	1.08
1.93	-1.97	2.21	1.29	3.43	67.79	1.84
7.88	-3.08	0	0.94	3.43	62.45	3.8
5.42	-13.84	0	-1.58	3.43	27.02	3.97
3.45	-3.35	1.53	0.85	3.43	61.18	1.95
3.73	-3.52	1.68	0.8	3.43	60.42	1.87
3.79	-3.72	1.25	0.73	3.43	59.51	2.31
5.31	-3.71	2.19	0.74	3.43	59.6	1.58
4.49	3.21	0.27	3.32	3.43	98.46	3.33
5.61	3.34	-3.21	3.39	3.44	99.3	13.18
3.18	-4.88	1.35	0.38	3.44	54.5	2.26
6.83	-5.4	0.08	0.24	3.44	52.4	3.68
14.57	-13	0.32	-1.45	3.44	28.9	3.6
2.63	-0.16	1.64	1.96	3.44	77.29	1.88
5.76	-2.83	0.3	1.02	3.44	63.57	3.6
8.7	-8.81	-0.12	-0.6	3.44	40.31	4
3.28	0.94	1.05	2.37	3.44	83.73	2.39
3.77	-3.58	1.43	0.78	3.44	60.11	2.09
4.22	-6	0.74	0.05	3.45	50	2.95
0.41	-14.23	0.69	-1.7	3.45	26.13	3.31
8.74	-1.77	1.54	1.37	3.45	68.66	1.77
17.43	-8.94	-1.78	-0.66	3.45	39.86	8.41
2.19	-1.43	1.55	1.48	3.45	70.41	2.01
1.65	-2.52	0.95	1.12	3.45	64.95	2.71
5.49	-2.71	-0.08	1.06	3.45	64.05	4
0.94	-8.73	1.36	-0.59	3.45	40.55	2.22
2.55	-3.58	0.46	0.77	3.45	60.05	3.09
4.73	3.13	0.55	3.3	3.45	97.75	3.1
0.36	-14.23	0.45	-1.7	3.46	26.1	3.16
1.72	-16.21	0.42	-1.95	3.46	22.17	3.84
3.22	-4.11	-0.22	0.62	3.46	57.67	4.95
3.82	-0.62	1.07	1.79	3.46	74.69	2.62
14.5	-17.75	-1.75	-2.15	3.46	19.5	7.28
3.24	-3.49	2.4	0.82	3.46	60.42	1.31
3.47	-3.4	3.17	0.85	3.46	60.82	0.89
3.62	-3.23	1.5	0.9	3.46	61.61	1.98
3.45	-2.75	3.16	1.06	3.46	63.86	0.91
3.46	-2.67	3.48	1.09	3.46	64.25	0.76
0.31	-4.06	0.91	0.63	3.46	57.9	2.84
5.5	2.15	3.45	2.88	3.46	91.14	0.76
4.41	2.86	0.54	3.19	3.46	95.84	3.24
4.72	3.1	0.12	3.3	3.46	97.53	3.72
4.71	3.17	0.6	3.33	3.46	97.99	2.92
1.06	-16.01	0.45	-1.92	3.47	22.54	3.29

2.79	-6.55	2.65	-0.07	3.47	47.94	1.23
2.17	-2.15	3.04	1.27	3.47	66.66	1.05
4.72	3.11	-0.02	3.31	3.47	97.47	3.98
6.16	-14.19	1.05	-1.63	3.48	26.16	2.3
7.08	-8.52	-1	-0.55	3.48	41.12	5.56
2.22	-16.25	-0.19	-1.93	3.48	22.07	4.42
7.44	-13.38	-0.09	-1.5	3.48	27.95	3.82
2.26	-1.42	1.47	1.51	3.48	70.31	2.16
2.63	-0.11	1.31	2	3.48	77.36	2.23
6.23	-2.75	0.43	1.07	3.48	63.75	3.31
3.68	-2.84	2.65	1.04	3.48	63.33	1.24
2.89	1.59	1.9	2.66	3.48	87.47	1.74
5.12	1.12	1.04	2.47	3.48	84.6	2.58
7.45	-13.88	0.41	-1.55	3.48	26.84	3.47
8.58	-8.74	-0.54	-0.55	3.48	40.41	5
3.43	1.5	2.85	2.62	3.48	86.89	1.13
2.85	1.61	2.96	2.66	3.48	87.58	1.07
3.48	0.99	0.59	2.42	3.48	83.74	3.19
3.5	-3.09	2.06	0.96	3.48	62.17	1.6
4.64	3.12	0.4	3.32	3.48	97.48	3.36
1.4	-14.27	2.16	-1.64	3.49	25.97	1.58
3.19	-3.59	2.32	0.81	3.49	59.83	1.41
4.67	1.22	0.81	2.52	3.49	85.13	2.63
3.58	-2.35	2.68	1.21	3.49	65.6	1.19
6.43	-5.11	0	0.36	3.49	53.36	3.69
3.75	-7.44	0.97	-0.24	3.49	44.68	2.72
5.51	2.33	3.91	2.98	3.49	92.08	0.7
3.67	-2.81	2.15	1.06	3.49	63.41	1.56
4.28	-12.49	6.24	-0.47	3.5	27.2	0.39
-12.1	-25.44	-15.79	-14.64	3.5	34.05	1.28
1.14	-3.81	2.69	0.73	3.5	58.79	1.29
7.22	-0.68	3.39	1.79	3.5	74.09	0.81
1.67	-3.91	1.77	0.7	3.5	58.36	1.99
2.74	-0.87	2.34	1.72	3.5	73.07	1.51
3.65	-2.44	2.11	1.19	3.5	65.15	1.57
7.82	-3.19	0.51	0.95	3.51	61.59	3.19
3.07	-2.48	2.9	1.18	3.51	64.88	0.92
1.77	-3.86	1.7	0.72	3.51	58.56	1.96
3.7	-3.15	1.9	0.96	3.51	61.76	1.69
3.81	-3.51	1.6	0.85	3.51	60.12	2.06
3.93	-3.81	1.42	0.76	3.51	58.79	2.22
3.68	-6.44	1.48	-0.02	3.52	48.12	1.71
2.25	-1.99	1.12	1.34	3.52	67.23	2.53
6.44	2.37	0.36	3.01	3.52	92.17	3.38

17.66	-17.76	-1.46	-2.11	3.52	19.38	8.14
5.9	-3.11	2.82	0.98	3.52	61.9	1.13
1.64	-2.34	1.19	1.22	3.52	65.52	2.49
6.38	-2.71	-0.06	1.1	3.52	63.76	4.1
5.24	1.15	0.83	2.51	3.52	84.5	2.83
2.4	-1.97	3.15	1.35	3.52	67.37	0.91
1.05	-8.8	1.59	-0.56	3.52	40.11	2.15
4.19	-4.09	1.21	0.67	3.52	57.52	2.5
5.69	-3.63	1.72	0.81	3.52	59.53	1.95
5.88	-3.69	1.53	0.8	3.52	59.25	2.17
4.38	2.89	0.25	3.25	3.52	95.63	3.59
-0.54	-10.2	1.24	-0.91	3.53	35.9	2.41
5.66	-14.44	-1.91	-1.67	3.53	25.55	8.06
8.75	-15.58	-1.33	-1.8	3.53	23.26	6.45
6.61	2.25	-0.03	2.96	3.53	91.33	4.26
7.7	-7.9	-1.55	-0.35	3.53	42.97	7.11
17.25	-17.71	-1.33	-2.1	3.53	19.48	7.48
2.33	-1.43	1.72	1.53	3.53	70.04	2.02
1.59	-2.73	1.13	1.1	3.53	63.65	2.62
1.69	-2.24	1.25	1.26	3.53	65.97	2.47
6.91	-2.83	0.06	1.07	3.53	63.17	3.86
7.07	-2.94	-0.03	1.04	3.53	62.66	3.9
3.63	0.98	0.52	2.44	3.53	83.45	3.37
4.44	3.01	0.68	3.3	3.53	96.35	2.99
7.17	-9.16	-1.18	-0.65	3.54	38.98	6.89
-0.32	-13.13	0.79	-1.47	3.54	28.39	2.62
2.19	-6.28	0.58	0.05	3.54	48.65	3.36
2.1	-2.57	1.2	1.16	3.54	64.32	2.44
3.81	1.5	2.78	2.65	3.54	86.51	1.19
2.92	1.63	3.95	2.71	3.54	87.32	0.68
4.93	-14.55	1.18	-1.65	3.55	25.27	2.32
-0.5	-10.28	1.3	-0.92	3.55	35.66	2.31
1.27	-16.36	0.65	-1.91	3.55	21.77	3.18
13.39	-2.18	0.35	1.29	3.55	66.17	2.92
2.05	-7.52	3.05	-0.26	3.55	44.22	1.33
16.54	-11.18	-2.25	-1.06	3.55	33.2	8.01
3.85	-7.48	1.01	-0.22	3.55	44.37	2.73
3.58	1.48	3.48	2.65	3.55	86.35	0.91
4.59	3.22	0.62	3.4	3.55	97.67	3.1
4.44	-14.45	1.16	-1.62	3.56	25.46	2.46
4.87	1.29	1.23	2.58	3.56	85.07	2.29
2.23	-2.18	2.99	1.3	3.56	66.12	1.07
2.3	-2.06	2.92	1.35	3.56	66.73	1.08
7.56	-13.94	0.55	-1.51	3.56	26.54	3.25

7.65	-14.1	0.67	-1.53	3.56	26.2	2.97
2.92	1.6	3.14	2.71	3.56	87.02	1.04
4.34	-2.23	1.77	1.29	3.57	65.84	1.54
7.49	-6.42	-1.22	0.04	3.57	48.04	6.74
19.84	-9.74	1.62	-0.75	3.57	37.15	1.71
3.66	-2.71	3.1	1.13	3.57	63.52	0.98
3.69	-2.52	2.88	1.19	3.57	64.44	1.14
8.38	-8.53	-0.43	-0.45	3.57	40.84	4.79
3.65	-8.3	1.71	-0.45	3.58	41.55	1.66
1.49	-7.41	3.46	-0.19	3.58	44.49	0.88
3.71	-3.1	2.79	1.01	3.58	61.72	1.19
3.88	-2.87	2.46	1.09	3.58	62.76	1.43
5.47	1.18	0.52	2.55	3.58	84.27	3.28
3.54	-1.04	2.9	1.7	3.58	71.78	1.17
6.37	-13.66	0.53	-1.46	3.58	27.13	3.39
6.54	-13.76	0.63	-1.47	3.58	26.91	3.37
5.45	2.31	2.66	3.02	3.58	91.4	1.22
3.84	-2.94	2.11	1.07	3.58	62.39	1.65
4.3	-6.1	0.44	0.11	3.59	49.18	3.7
4.25	-13.55	3.84	-1.38	3.59	27.14	0.72
2.48	-8.44	1.85	-0.45	3.59	41.08	1.97
2.29	-1.87	1.4	1.42	3.59	67.5	2.47
5.86	3.44	-2.96	3.52	3.59	98.95	14.06
5.13	-5.61	1.32	0.27	3.59	51.01	2.17
20.41	-10.24	1.32	-0.85	3.59	35.64	2.5
8.04	-2.87	0.63	1.1	3.59	62.73	3.05
8.13	-2.9	0.59	1.09	3.59	62.56	3.04
3.51	-13.2	1.21	-1.38	3.59	28.14	2.6
5.81	-13.86	0.54	-1.48	3.59	26.66	3.26
1.64	-4.03	2.54	0.72	3.59	57.5	1.59
2.96	1.72	3	2.78	3.59	87.53	1.04
4.02	-4.83	2.39	0.51	3.59	54.16	1.56
5.58	2.31	2.89	3.03	3.59	91.4	1.12
4.09	-3.91	1.72	0.77	3.59	58.01	1.99
7.79	-11.46	5.15	0.48	3.6	27.05	1.46
8.82	-8.7	9.17	2.82	3.6	27.11	1.26
4.05	-11.06	-1.41	-1	3.6	33.39	6.9
3.94	-0.49	1.46	1.92	3.6	74.62	2.32
15.81	-18.14	-1.29	-2.11	3.6	18.68	7.32
5.47	-3.51	2.45	0.9	3.6	59.74	1.39
1.49	-2.87	1.61	1.1	3.6	62.65	2.1
1.65	-2.71	1.82	1.15	3.6	63.41	2.06
7.34	-7.07	1.98	-0.09	3.6	45.62	1.82
4.73	-13.63	0.74	-1.44	3.6	27.16	3.06

3.96	-7.52	0.92	-0.2	3.6	44.08	2.89
4.03	-3.91	1.74	0.78	3.6	57.98	1.97
4.12	-3.92	1.56	0.77	3.6	57.96	2.15
4.16	-3.83	1.6	0.8	3.6	58.33	2.06
4.21	-4.23	1.33	0.68	3.6	56.59	2.41
4.21	-4.28	1.37	0.67	3.6	56.39	2.39
10.11	-0.98	-0.9	1.75	3.6	72.01	5.15
0.65	-11.72	1	-1.11	3.61	31.65	2.87
10.68	-3.87	-0.6	0.78	3.61	58.13	4.97
3.28	-4.92	1.33	0.47	3.61	53.73	2.4
15.37	-17.58	-1.33	-2.04	3.61	19.58	6.76
16.7	-17.86	-1.57	-2.07	3.61	19.11	8.48
0.98	-3.74	2.63	0.82	3.61	58.67	1.35
1.41	-2.97	1.58	1.07	3.61	62.17	2.15
1.62	-2.77	1.53	1.14	3.61	63.07	2.22
1.41	-7.54	1.63	-0.23	3.61	43.96	1.75
4.04	-7.6	1.03	-0.21	3.61	43.79	2.81
3.75	1.03	0.18	2.52	3.61	83.22	4.03
4.21	-4.14	1.12	0.71	3.61	56.96	2.62
4.98	-13.62	4.01	-1.41	3.62	27.04	0.76
1.4	-8.97	2.5	-0.56	3.62	39.32	1.47
17.32	-11.27	-1.4	-1.04	3.62	32.81	6.8
5.61	2.24	2.9	3.01	3.62	90.68	1.22
4.25	-4.45	1.48	0.62	3.62	55.59	2.35
-2.67	-19.08	-4.64	-6.3	3.63	26.96	1.46
1.54	-16.67	0.41	-1.91	3.63	21.1	3.32
2.97	-6.65	3.04	0	3.63	47.03	1.12
7.25	-7.17	0.16	-0.11	3.63	45.15	4.11
0.3	-3.97	0.75	0.76	3.63	57.57	2.83
2.95	1.62	3.85	2.76	3.63	86.73	0.78
5.4	1.62	1.33	2.76	3.63	86.68	2.23
3.07	-14.34	1.91	-1.55	3.64	25.56	1.93
6.15	-13.67	4	-1.41	3.64	26.89	0.73
2.49	-16.25	0.26	-1.84	3.64	21.84	4.21
1.79	-9.04	3.52	-0.56	3.64	39.05	0.87
8.1	-13.78	0.9	-1.43	3.64	26.76	2.71
2.52	-14.36	2.11	-1.55	3.65	25.5	1.7
8.72	-15.72	-1.21	-1.75	3.65	22.8	7.03
2.71	-6.44	2.7	0.07	3.65	47.73	1.26
3.85	1.59	1.58	2.76	3.65	86.43	2.21
7.64	-6.36	-1.13	0.11	3.66	47.97	7.16
6.18	-14.9	-1.77	-1.63	3.66	24.38	9.63
7.97	-5.57	-0.21	0.31	3.66	50.91	4.36
1.97	-3.92	2.88	0.78	3.66	57.68	1.17

2.67	-0.2	1.71	2.06	3.66	75.93	2.18
4.77	-2.97	1.15	1.1	3.66	61.93	2.63
7.95	-8.47	-0.13	-0.38	3.66	40.76	4.66
-16.42	-32.39	-21.14	-19.25	3.67	26.89	1.15
3.86	-1.07	2.19	1.74	3.67	71.19	1.72
6.17	-1.42	1.14	1.62	3.67	69.41	2.85
9.08	-1.79	2.36	1.49	3.67	67.51	1.64
1.61	-7.43	3.31	-0.14	3.67	44.15	1.01
17.78	-14.87	-1.77	-1.62	3.67	24.41	8.13
4.14	-2.41	1.68	1.28	3.67	64.52	1.92
4.14	-7.7	1.27	-0.19	3.67	43.23	2.59
7.93	-8.49	-0.38	-0.38	3.67	40.67	5.03
1.61	-19.34	-4.33	-6.5	3.68	26.87	1.15
4.08	-0.36	1.8	2.01	3.68	74.88	2.11
2.38	-7.72	1.95	-0.24	3.68	43.15	1.93
1.23	-3.71	2.61	0.87	3.68	58.51	1.37
0.92	-3.69	2.64	0.87	3.68	58.6	1.32
1.02	-3.68	2.43	0.88	3.68	58.66	1.4
4.48	-13.98	0.82	-1.44	3.68	26.24	3
1.29	-8.82	2.34	-0.48	3.68	39.63	1.64
2.77	-0.97	2.05	1.79	3.68	71.69	1.85
4.3	-4.39	1.64	0.68	3.68	55.63	2.26
7.4	-8.76	-0.53	-0.47	3.69	39.75	5.31
-7.49	-25.38	-10.86	-12.23	3.69	26.84	0.73
10.91	-9.79	3.87	-0.66	3.69	36.68	0.98
3.35	-4.85	1.26	0.55	3.69	53.69	2.63
20.87	-11.46	1.09	-1.03	3.69	32.12	2.84
1.21	-8.78	1.79	-0.46	3.69	39.72	2.08
3.13	-3.37	0.69	0.98	3.69	59.99	3.22
3.87	1.06	0.37	2.57	3.69	82.94	3.83
3.93	-2.77	1.7	1.19	3.69	62.73	1.95
9.19	-8.71	9.83	2.92	3.7	26.75	1.09
4.89	-12.5	5.2	-0.36	3.7	26.81	0.8
-0.33	-10.29	1.21	-0.83	3.7	35.23	2.63
-0.25	-10.25	1.32	-0.83	3.7	35.35	2.71
0.83	-13.56	1.08	-1.44	3.7	27.11	2.93
8.76	-15.21	-1.25	-1.65	3.7	23.67	6.79
1.61	-9.07	2.91	-0.54	3.7	38.82	1.15
2.43	-1.34	1.47	1.66	3.7	69.64	2.36
6.02	-2.95	2.99	1.13	3.7	61.85	1.08
6.56	-2.5	2.68	1.28	3.7	63.94	1.4
1.97	0.66	1.49	2.41	3.7	80.52	2.28
2.98	-3.43	0.52	0.97	3.7	59.65	3.31
-0.33	-10.38	1	-0.85	3.71	34.98	2.94

17.09	-17.28	-1.57	-1.94	3.71	19.92	7.9
17.4	-16.23	-1.64	-1.79	3.71	21.75	7.92
4.08	-3.85	0.49	0.83	3.71	57.78	3.64
4.18	-3.68	0.81	0.88	3.71	58.55	3.31
2.47	-1.29	1.31	1.68	3.71	69.86	2.55
7.65	-8.65	-0.26	-0.4	3.71	40.08	4.82
8.29	-7.95	-0.15	-0.24	3.71	42.32	4.42
2.4	-11.36	0.52	-1.01	3.72	32.33	3.62
3.09	-6.72	3.15	0.04	3.72	46.49	1.14
7.39	-7.19	0.21	-0.07	3.72	44.84	4.11
7.51	-7.11	0.54	-0.05	3.72	45.1	3.7
7.67	-7.93	1.24	-0.23	3.72	42.32	2.42
5.95	-1.71	2.89	1.55	3.72	67.66	1.04
7.21	-13.88	1	-1.4	3.72	26.4	3.04
3.91	1.34	2.79	2.69	3.72	84.47	1.41
5.55	2.59	4.1	3.21	3.72	92.32	0.65
2.13	-3.93	3.56	0.82	3.73	57.36	1.28
3.72	-1.99	-0.32	1.44	3.73	66.24	5.05
3.78	-2.22	-0.15	1.37	3.73	65.15	4.89
3.89	-3.14	-0.53	1.07	3.73	60.85	5.59
4.39	-6.2	0.3	0.17	3.74	48.3	4.01
1.15	-12.05	1.2	-2.04	3.74	34.07	1.22
1.4	-9.1	3.23	-0.51	3.74	38.58	0.99
2.71	-0.19	2.08	2.11	3.74	75.54	1.94
3.88	1.58	2.45	2.8	3.74	85.79	1.52
6.2	1.22	0.56	2.65	3.74	83.62	3.59
5.8	1.32	0.88	2.69	3.74	84.2	2.95
7.98	-7.58	0.57	-0.13	3.74	43.42	3.39
4.1	1.2	2.61	2.65	3.74	83.5	1.44
3.89	1.56	2.51	2.8	3.74	85.65	1.5
5.58	2.57	3.43	3.22	3.74	92.07	0.89
2.5	-6.64	2.35	0.08	3.75	46.64	1.77
6.06	1.25	0.76	2.67	3.75	83.7	3.27
3.39	-2.17	1.78	1.41	3.75	65.29	2.18
2.84	-1.01	2.45	1.82	3.75	71.1	1.54
10.55	3.66	2.15	3.72	3.76	99.26	1.54
0.88	-9.05	2.79	-0.48	3.76	38.68	1.36
2.46	-7.88	2.18	-0.23	3.76	42.38	1.92
4.14	-4.78	3.14	0.62	3.76	53.7	1.12
1.31	-3.75	2.66	0.9	3.76	58.02	1.44
4.39	-2.42	1.6	1.33	3.76	64.03	2.14
4.33	-7.68	1.55	-0.14	3.76	43.04	2.38
4.58	-7.83	1.41	-0.17	3.76	42.53	2.61
5.67	2.07	3.79	3.01	3.76	88.7	0.82

2.8	-0.99	1.98	1.83	3.76	71.1	1.93
2.74	-8.43	1.6	-0.34	3.77	40.57	2.33
2.42	-6.1	1.74	0.23	3.77	48.54	2.12
8.93	-1.69	2.43	1.58	3.77	67.56	1.42
7.54	-8.04	0.5	-0.21	3.77	41.82	3.67
4.14	1.24	1.78	2.68	3.77	83.54	2.16
4.38	-4.28	1.75	0.76	3.77	55.74	2.22
4.5	-6.29	0.89	0.17	3.78	47.82	3.43
6.12	-14.39	-1.58	-1.51	3.78	25.2	8.04
3.43	-4.81	1.42	0.61	3.78	53.53	2.51
3.52	-4.89	1.44	0.58	3.78	53.18	2.63
7.13	-4.83	0.45	0.6	3.78	53.42	3.4
2	-5.31	3.65	0.45	3.78	51.54	1.01
6.26	-2.66	3.61	1.28	3.78	62.86	0.87
7.56	-8.66	0.82	-0.36	3.78	39.82	3.29
7.4	-8.71	-0.65	-0.4	3.79	39.64	5.75
8.62	-15.36	-1.35	-1.62	3.79	23.25	7.78
7.08	2.48	-0.23	3.21	3.79	91.16	4.67
16.5	-17.25	-1.55	-1.88	3.79	19.87	7.92
2.18	0.49	1.21	2.4	3.79	79.1	3.24
4.07	1.23	2.17	2.7	3.79	83.41	1.79
4.12	1.28	2.32	2.71	3.79	83.66	1.64
2.46	-10.99	0.64	-0.89	3.8	33.11	3.55
4.23	-1.07	2.31	1.81	3.8	70.55	1.78
8.64	-0.52	1.36	2.02	3.8	73.42	2.54
4.7	-10.82	-1.39	-0.84	3.8	33.56	7.53
5.43	-4.89	2.85	0.59	3.8	53.1	1.13
17.39	-16.7	-1.7	-1.8	3.8	20.8	7.95
4.28	-4.09	0.83	0.82	3.8	56.37	3.31
1.72	-2.72	1.02	1.26	3.8	62.5	2.72
2.1	0.61	0.7	2.44	3.8	79.66	3.51
8.02	-0.59	3.35	1.99	3.8	73.03	1.06
10.88	-14.04	0.7	-1.41	3.8	25.88	3.51
3.24	-3.19	0.86	1.1	3.8	60.3	3.1
2.32	-1.66	1.94	1.62	3.81	67.49	2.07
1.79	-7.39	3.51	-0.05	3.81	43.85	0.94
3.11	-1.48	-0.2	1.67	3.81	68.38	5.29
3.98	-3.28	-0.34	1.08	3.81	59.87	5.37
2.66	-1.85	3.35	1.56	3.81	66.54	1.05
1.47	-8.72	2.56	-0.37	3.81	39.56	1.56
7.2	-2.1	1.77	1.48	3.81	65.34	2.28
7.11	-2.04	2.17	1.49	3.81	65.67	1.93
6.93	-1.52	1.71	1.67	3.82	68.16	2.68
11.04	-9.96	2.68	-0.62	3.82	35.88	1.48

15.04	-1.25	0.21	1.76	3.82	69.5	3.72
14.7	-12.88	0.65	-1.2	3.82	28.42	3.36
3.84	-2.42	-0.57	1.35	3.82	63.77	5.62
3.01	-0.79	3.09	1.93	3.82	71.89	1.33
8.21	-0.55	2.86	2.02	3.82	73.14	1.33
2.07	-9.05	2.96	-0.45	3.83	38.51	1.3
1.95	-7.32	3.64	-0.02	3.83	44.03	0.93
6.83	-2.24	2.69	1.44	3.83	64.58	1.48
4.18	1.25	1.73	2.72	3.83	83.25	2.31
5.63	2.16	3.44	3.09	3.83	88.86	0.96
11.79	-5.11	0.26	0.57	3.83	52.15	4.87
9.26	-1.69	2.69	1.62	3.84	67.19	1.51
6.13	-9.59	3.75	-0.54	3.84	36.88	0.94
0.95	-8.9	2.97	-0.4	3.84	38.93	1.34
0.84	-3.65	3.02	0.98	3.84	58.12	1.19
3.29	-2.26	1.66	1.44	3.84	64.42	2.12
1.4	-7.51	1.98	-0.08	3.84	43.38	1.8
4.19	1.23	1.74	2.72	3.84	83.09	2.43
0.35	-18.71	-3.4	-5.88	3.85	26.74	1.18
-2.27	-16.05	-4.41	-5.7	3.85	34.08	1.5
8.55	-8.3	1.15	-0.24	3.85	40.76	2.78
4.03	-3.3	0.13	1.09	3.85	59.62	4.4
0.87	-3.69	2.3	0.97	3.85	57.89	1.82
4.73	-7.62	1.33	-0.07	3.85	42.96	2.73
5.68	1.96	2.75	3.01	3.85	87.5	1.48
4.47	-4.16	1.77	0.85	3.85	55.9	2.24
4.8	-5.68	1.86	0.41	3.85	49.84	2.24
6.7	-3.66	1.65	1	3.85	58.04	2.52
3	-8.45	1.41	-0.29	3.86	40.25	2.72
20.78	-8.87	1.15	-0.39	3.86	38.96	3.12
6.15	-2.68	3.4	1.32	3.86	62.37	0.96
6.34	-2.63	3.6	1.33	3.86	62.62	0.9
2.97	-1.78	3.64	1.62	3.86	66.66	0.95
4.91	-8.15	1.07	-0.19	3.86	41.21	3.09
5.09	-8.17	1.12	-0.2	3.86	41.15	3.1
-0.21	-10.31	1.35	-0.73	3.87	34.79	2.87
11.27	-3.27	0.15	1.13	3.87	59.66	4.6
0.71	-13.9	1.07	-1.39	3.88	26.04	3.31
7.63	-7.23	0.82	0.02	3.88	44.18	3.57
8.48	2.75	-0.7	3.37	3.88	92.31	6.93
5.34	-3.91	3.57	0.92	3.88	56.87	0.97
17.89	-16.71	-1.36	-1.75	3.88	20.65	7.5
4.38	-4.42	0.67	0.76	3.88	54.7	3.97
-0.47	-10.06	1.13	-0.66	3.89	35.4	2.62

0.63	-14.13	1.19	-1.42	3.89	25.55	3.02
3.18	-6.82	3.13	0.12	3.89	45.54	1.37
5.95	-10.25	1.14	-0.65	3.89	34.89	2.81
2.4	-7.58	0.71	-0.09	3.89	43	3.93
5.13	-2.75	1.16	1.3	3.89	61.95	2.72
7.36	-13.7	1.43	-1.27	3.89	26.45	2.38
5.7	0.22	2.66	2.35	3.89	76.99	1.13
10.09	-9.2	0.9	-0.43	3.9	37.85	3.02
3.6	-0.42	3.12	2.11	3.9	73.47	1.51
3.69	-2.47	-0.41	1.38	3.9	63.17	5.42
2.87	-1.76	3.55	1.65	3.9	66.59	0.98
2.76	-1.67	3.3	1.68	3.9	67.04	1.09
7.52	-8.54	0.96	-0.26	3.9	39.87	3.18
6.91	-2.08	2.17	1.54	3.9	65.05	1.85
10.26	-9.27	0.71	-0.44	3.91	37.61	3.48
14.61	-12.7	0.46	-1.11	3.91	28.64	3.88
9.84	-14.94	1.5	-1.46	3.91	23.88	2.38
3.06	-1.29	-0.12	1.79	3.91	68.88	5.09
4.67	-5.43	0.28	0.49	3.91	50.61	4.61
6.89	-5.33	0.35	0.54	3.91	50.95	3.91
1.72	-8.69	2.65	-0.31	3.91	39.39	1.6
3	1.79	3.12	2.98	3.91	86.12	1.08
3.59	-4.86	1.51	0.68	3.92	52.78	2.65
3.5	-0.19	2.31	2.2	3.92	74.61	1.68
2.29	-3.93	3.19	0.93	3.92	56.57	1.28
3.49	-0.17	2.77	2.22	3.92	74.67	1.43
4.61	-4.69	2.6	0.73	3.92	53.47	1.65
2.47	-1.69	1.85	1.68	3.93	66.79	2.31
1.61	-8.64	2.71	-0.28	3.93	39.45	1.5
3.86	1.67	2.78	2.94	3.93	85.2	1.39
4.71	-5.22	2.18	0.59	3.93	51.31	1.96
4.86	-5.77	2.07	0.44	3.93	49.21	2.06
4.91	-5.69	2.25	0.46	3.93	49.53	1.93
7.42	-4.35	1.7	0.85	3.93	54.82	2.5
-2.25	-18.92	-4.07	-6.08	3.94	26.74	1.29
8.4	-0.44	2.39	2.12	3.94	73.12	1.61
9.58	-8.26	3.24	-0.18	3.94	40.62	1.4
4.21	0.23	2.11	2.38	3.94	76.78	2.07
8.8	-7.96	-0.63	-0.12	3.94	41.58	6.06
8.72	-6.95	-0.66	0.12	3.94	44.96	6
20.66	-9.73	2.03	-0.53	3.94	36.2	1.75
1.38	-7.4	2.16	0.01	3.94	43.42	1.88
3.54	-14.44	1.59	-1.38	3.94	24.81	2.82
1.56	-3.97	2.09	0.94	3.94	56.34	2.1

5.66	-8.3	0.65	-0.18	3.94	40.5	3.9
4.96	-5.84	2.27	0.42	3.94	48.95	1.96
1.13	-11.59	1.43	-0.88	3.95	31.23	2.87
0.93	-13.59	1.42	-1.29	3.95	26.58	2.95
6.48	-14.17	-1.25	-1.37	3.95	25.34	7.75
2.6	-6.15	1.48	0.33	3.95	47.78	2.75
3.36	-8.54	1.32	-0.25	3.95	39.73	2.93
7.65	2.6	0.17	3.34	3.95	90.89	4.56
7.33	-4.29	0.37	0.86	3.95	54.97	4.13
2.07	-1.84	1.65	1.64	3.95	65.98	2.63
6.39	-2.48	3.52	1.43	3.95	62.92	0.98
6.07	-0.01	3.46	2.3	3.95	75.37	0.97
5.26	-8.17	1.05	-0.15	3.95	40.89	3.24
7.62	-8.52	1.06	-0.22	3.95	39.78	3.36
4.77	-5.35	1.96	0.56	3.95	50.76	2.11
8.43	-15.57	-1.32	-1.54	3.96	22.58	7.7
4.4	-4.7	2.8	0.76	3.96	53.25	1.5
4.22	-0.24	3.8	2.22	3.96	74.07	1.1
2.63	-11.29	0.98	-0.84	3.97	31.94	3.46
0.8	-13.62	0.86	-1.28	3.97	26.49	3.51
10.86	-9.74	3.79	-0.48	3.97	36.11	0.82
3.35	-6.85	2.91	0.16	3.97	45.2	1.6
4.57	-5.26	0.45	0.57	3.97	51.02	4.21
2.85	-0.13	2.67	2.27	3.97	74.63	1.64
4.12	-0.04	3.44	2.29	3.97	75.1	1.01
3.95	1.44	3.1	2.88	3.97	83.6	1.38
5.13	0.57	1.72	2.53	3.97	78.51	2.52
9.26	-9.53	2.76	-0.44	3.97	36.71	1.62
0.84	-13.57	0.6	-1.27	3.98	26.58	3.7
2.75	-16.05	0.54	-1.6	3.98	21.68	4.32
2.04	-16.94	0.59	-1.73	3.98	20.13	4.08
5.93	-1.86	1.68	1.64	3.98	65.71	2.21
4.28	-8.29	3.03	-0.21	3.98	40.42	1.16
3.44	-0.09	1.69	2.28	3.98	74.82	2.56
3.21	-1.58	-0.54	1.74	3.98	67.08	6.46
2.6	-1.85	3.34	1.65	3.98	65.77	1.19
12.95	-5.59	0.44	0.52	3.98	49.72	4.56
3.65	-2.37	-0.72	1.47	3.99	63.24	6.79
3.68	-2.47	-0.71	1.43	3.99	62.76	6.47
2.8	-0.08	2.67	2.3	3.99	74.8	1.57
4.57	-1.01	2.91	1.94	3.99	69.88	1.49
8.7	-7.64	2.28	0	3.99	42.48	1.96
2.15	-7.45	4.18	0.05	4	43.07	0.77
4.5	-4.81	3.64	0.76	4	52.71	1.02

20.8	-9.67	2.33	-0.48	4	36.24	1.99
9.42	-1.7	3.22	1.71	4	66.41	1.29
3.51	-0.19	2.88	2.25	4	74.16	1.39
2.88	-16.32	0.06	-3.69	4.01	26.69	0.97
6.58	-1.24	1.2	1.88	4.01	68.65	2.83
2.64	-6.94	2.31	0.16	4.01	44.77	1.92
2.23	-9.1	3.61	-0.35	4.01	37.87	1.09
7.87	-7.05	2.82	0.16	4.01	44.38	1.48
1.87	-5.17	3.17	0.63	4.01	51.23	1.26
6.98	-3.91	2.92	1.02	4.01	56.35	1.47
2.48	-1.69	2.34	1.74	4.01	66.42	2.02
6.52	1.55	0.65	2.94	4.01	84.05	3.62
3.51	-2.06	2.11	1.59	4.01	64.65	1.94
6.09	-8.29	1.79	-0.13	4.01	40.36	2.53
4.21	1.3	1.69	2.84	4.01	82.52	2.52
4.22	1.29	1.63	2.84	4.01	82.44	2.64
5.14	0.53	1.63	2.54	4.01	78.05	2.68
10.97	-4.28	0.23	0.9	4.02	54.78	4.42
6.05	-1.82	1.93	1.68	4.02	65.75	2.2
2.42	-1.6	1.95	1.76	4.02	66.81	2.3
2.5	-1.65	1.85	1.74	4.02	66.56	2.21
1.07	-8.69	3.1	-0.25	4.02	39.07	1.31
2.87	-7.31	3.19	0.07	4.02	43.49	1.28
4.33	-0.41	1.75	2.19	4.02	72.86	2.52
1.55	-4.02	2.22	0.97	4.02	55.84	2.34
1.35	-11.5	1.76	-0.82	4.03	31.3	2.7
1.04	-13.73	1.75	-1.26	4.03	26.12	2.57
6.86	-14.08	-0.81	-1.31	4.03	25.41	7.33
11.21	-4.91	-0.35	0.72	4.03	52.19	5.88
5.32	-11.18	-1.18	-0.77	4.03	32.1	7.85
9.59	-9.1	4.08	-0.31	4.03	37.81	0.84
3.69	-5.01	1.87	0.7	4.03	51.81	2.43
7.74	-13.79	2.74	-1.21	4.03	26	1.57
1.44	-3.82	1.67	1.03	4.03	56.64	2.67
4.23	1.33	1.49	2.87	4.03	82.61	2.86
4.62	-6.16	1.46	0.36	4.04	47.42	2.84
0.95	-13.55	1.52	-1.23	4.04	26.49	2.8
3.65	-8.55	1.11	-0.21	4.04	39.44	3.43
3.66	-3.71	0.76	1.08	4.04	57.05	4.47
8.08	2.54	-0.25	3.37	4.04	89.99	5.57
7.98	-6.06	-0.85	0.42	4.04	47.8	7.41
7.76	-7.15	0.76	0.13	4.04	43.94	4.18
2.01	-1.87	1.93	1.68	4.04	65.41	2.19
7.45	-1.89	3.06	1.68	4.04	65.28	1.37

9.14	-7.87	2.4	-0.02	4.04	41.56	1.99
11.47	-3.94	0.41	1.03	4.04	56.1	4.49
2.7	-11.55	1	-0.85	4.05	31.12	3.83
3.65	-2.2	-0.81	1.56	4.05	63.76	7.14
3.94	1.44	3.53	2.91	4.05	83.09	1.17
0.79	-13.68	0.84	-1.24	4.06	26.18	3.72
7.6	-1.53	1.34	1.8	4.06	66.96	3.44
4.49	-5.06	0.36	0.68	4.06	51.5	4.6
4.9	-5.82	0.18	0.47	4.06	48.59	4.92
20.57	-8.56	0.87	-0.2	4.06	39.35	3.7
8.99	-7.48	2.36	0.08	4.06	42.79	1.88
2.39	-5.76	1.88	0.5	4.07	48.77	2.3
4.64	-4.64	3.99	0.85	4.07	53.09	0.86
3.27	-1.43	-0.55	1.84	4.07	67.44	6.67
3.55	-1.9	-0.95	1.67	4.07	65.14	7.72
5.01	-6	0.47	0.43	4.07	47.92	4.52
0.3	-3.88	0.71	1.04	4.07	56.22	3.44
3.86	1.7	3.42	3.03	4.07	84.57	1.06
8.85	-7.73	2.32	0.02	4.07	41.95	1.94
8.58	-13.18	4.71	-0.9	4.08	26.68	0.75
6.22	3.87	-1.17	3.98	4.08	98.5	10.46
6.76	-13.96	-0.99	-1.23	4.08	25.57	8.56
2.36	-3.97	3.57	1.02	4.08	55.82	1.37
3.58	-1.91	-0.86	1.67	4.08	65.06	7.52
4.79	-5.59	0.19	0.55	4.08	49.38	5.11
12.93	-4.9	1.06	0.77	4.08	52.03	3.71
9.38	-1.65	2.63	1.78	4.09	66.24	1.7
15.55	-1.26	0.06	1.93	4.09	68.14	4.59
1.94	-5.19	3.35	0.67	4.09	50.9	1.08
6.2	-3.8	3.49	1.08	4.09	56.46	1.04
2.86	-0.09	2.52	2.34	4.09	74.24	1.84
6.58	-8.96	1.94	-0.24	4.09	38.09	2.37
6.67	-8.72	1.96	-0.18	4.09	38.78	2.33
6.77	-9.15	2	-0.27	4.09	37.52	2.27
7.59	-8.05	1.28	-0.03	4.09	40.85	3
5.17	0.52	1.51	2.58	4.09	77.54	2.93
5.19	0.58	1.68	2.6	4.09	77.88	2.66
3.07	-0.86	2.86	2.07	4.09	70.2	1.57
11.61	-3.49	0.52	1.19	4.09	57.8	3.98
1.25	-8.59	3.03	-0.18	4.1	39.17	1.49
4.43	-0.38	2.16	2.24	4.1	72.63	2.19
8.11	-6.2	-1.11	0.41	4.1	47.08	8.53
2.51	-7.66	2.35	0.03	4.1	42.08	1.95
3.14	-7.32	2.28	0.12	4.1	43.21	2.06

3.19	-7.29	2.54	0.12	4.1	43.3	1.67
3.26	-7.1	1.69	0.18	4.1	43.95	2.49
2.12	-8.61	2.8	-0.17	4.1	39.12	1.63
6.9	-8.13	1.47	-0.04	4.1	40.59	2.88
4.25	1.33	1.56	2.91	4.1	82.21	2.84
5.2	0.56	1.45	2.6	4.1	77.74	3.01
5.21	0.56	1.42	2.6	4.1	77.74	3
5.9	-6.36	1.9	0.39	4.1	46.52	2.5
7.63	-4.19	2.17	0.99	4.1	54.82	2.07
-0.12	-10.33	2.14	-0.59	4.11	34.15	2.34
2.86	-5.92	1.69	0.49	4.11	48.05	2.81
14.23	-2.11	0.97	1.63	4.11	63.94	3.63
3.77	-5.09	2.05	0.72	4.11	51.16	2.27
8.11	-7.16	2.55	0.19	4.11	43.71	1.55
1.91	-7.42	1.94	0.1	4.11	42.82	1.97
5.22	0.48	0.96	2.58	4.11	77.22	3.7
5.23	0.45	1.07	2.56	4.11	77.1	3.56
5.27	-5.92	2.01	0.51	4.11	48.07	2.36
11.02	-2.86	-0.19	1.4	4.11	60.5	4.86
1.21	-13.69	2.11	-1.2	4.12	26.07	2.24
4.08	-8.38	1.1	-0.12	4.12	39.72	3.68
9.37	-7.93	-0.72	-0.01	4.12	41.19	6.89
2.92	-0.08	2.39	2.37	4.12	74.11	2.07
1.95	-1.92	2.01	1.71	4.12	64.81	2.39
2.29	-1.74	1.71	1.78	4.12	65.66	2.56
5.69	-6.43	1.89	0.37	4.12	46.2	2.47
7.16	-3.7	1.51	1.15	4.12	56.81	2.63
8.36	-15.61	-0.73	-1.44	4.13	22.25	6.5
10.35	-8.78	4.43	-0.18	4.13	38.49	0.82
2.52	-7.63	1.21	0.05	4.13	42.08	3.49
2.26	0.56	1.16	2.61	4.13	77.58	3.46
6.35	-7.88	1.79	0.04	4.13	41.28	2.57
2.41	-15.65	0.81	-1.45	4.14	22.16	4.2
3.86	-5.19	1.68	0.71	4.14	50.7	2.79
8.75	3.12	-0.2	3.68	4.14	93.13	6.04
5.47	-2.51	1.69	1.52	4.14	61.95	2.45
2.29	0.37	0.62	2.54	4.14	76.5	4.31
2.34	0.02	0.6	2.41	4.14	74.56	4.32
4.51	-2.28	1.32	1.59	4.14	63.02	2.8
7.11	-7.08	1.88	0.23	4.14	43.87	2.59
14.05	-2.1	0.76	1.66	4.15	63.78	3.25
17.87	-15.7	-1.62	-1.44	4.15	22.04	7.7
3.33	-1.4	-0.31	1.9	4.15	67.2	6.47
3.36	-1.22	-0.19	1.96	4.15	68.07	6.03

3.52	-1.74	-1	1.78	4.15	65.52	7.9
3.57	-1.54	3.94	1.87	4.15	66.5	0.96
3.42	-6.97	2.94	0.24	4.16	44.2	1.59
2.59	-7.61	3.57	0.09	4.16	42.06	1.31
2.23	-7.23	3.42	0.18	4.16	43.31	1.4
14.73	-12.98	0.72	-1	4.16	27.53	4.22
3.6	-1.92	-0.77	1.72	4.16	64.6	7.31
2.31	0.21	0.54	2.49	4.16	75.5	4.44
3.13	-0.79	3.29	2.13	4.16	70.19	1.37
7.31	-1.75	2.56	1.8	4.16	65.47	1.61
5.26	0.5	1.38	2.61	4.16	77.06	3.11
-2.54	-15	-2.38	-4.75	4.17	34.08	1.38
10.21	-9.07	3.47	-0.22	4.17	37.53	1.29
3.63	-1.79	-0.91	1.77	4.17	65.19	7.59
6.65	1.62	1.1	3.06	4.17	83.54	3.16
3.61	-2.05	2.48	1.69	4.17	63.98	1.78
1.54	-3.78	2.76	1.13	4.17	56.24	1.87
5.18	0.6	2.3	2.65	4.17	77.59	2.04
5.29	0.46	1.51	2.6	4.17	76.79	2.98
9.77	-9.02	3.11	-0.21	4.18	37.65	1.32
15.38	-1.63	0.36	1.84	4.18	65.92	4.75
4.55	-0.18	2.66	2.36	4.18	73.29	1.8
5.98	-5.2	2.14	0.73	4.18	50.51	2.01
3.5	-1.77	-0.89	1.78	4.18	65.28	7.76
2.3	-8.64	2.82	-0.13	4.18	38.77	1.68
3.73	-14.58	1.74	-1.26	4.18	24.12	2.85
3.87	1.61	4	3.06	4.18	83.35	0.96
4.28	1.38	1.74	2.97	4.18	82.01	2.7
5.32	0.47	1.84	2.61	4.18	76.84	2.58
5.37	0.42	1.66	2.59	4.18	76.51	2.89
6.13	-6.45	2.06	0.41	4.18	45.94	2.43
1.54	-11.51	2.13	-0.72	4.19	30.92	2.48
9.93	-8.18	-0.44	-0.02	4.19	40.18	6.72
9.39	-7.38	-0.39	0.16	4.19	42.75	6.02
3.46	-0.12	2.04	2.38	4.19	73.57	2.29
2.96	-7.17	2.51	0.21	4.19	43.43	1.84
3.41	-1.48	-0.65	1.88	4.19	66.57	7.23
3.45	-1.65	-0.7	1.83	4.19	65.73	7.35
3.48	-1.75	-0.8	1.79	4.19	65.31	7.61
2.58	-1.7	2.38	1.83	4.19	65.53	2.16
1.42	-3.88	2.22	1.11	4.19	55.76	2.4
5.22	0.91	1.68	2.79	4.19	79.24	2.71
5.47	-6.05	1.91	0.52	4.19	47.3	2.43
4.72	-6.22	1.65	0.43	4.2	46.68	3.31

-7.7	-25.73	-11.93	-12.49	4.2	26.63	1.04
-0.17	-12.04	2.01	-0.87	4.2	29.6	2.49
5.91	-11.28	-1.1	-0.68	4.2	31.47	8.3
2.76	-7.62	3.34	0.1	4.2	41.91	1.25
3.99	-5.27	1.67	0.73	4.2	50.16	2.97
1.94	-1.93	2.33	1.76	4.2	64.38	1.95
11.08	-14.28	1.59	-1.2	4.2	24.68	2.71
-0.1	-12.4	1.74	-0.93	4.21	28.73	2.65
8.37	2.61	-0.11	3.49	4.21	89.37	5.79
7.66	-3.78	0.54	1.16	4.21	56.11	4.25
4.79	-4.19	4.47	1.06	4.21	54.39	0.71
2.73	-1.19	1.73	2	4.21	67.94	2.82
4.37	1.42	1.95	3	4.21	82.1	2.53
10.55	-9.07	1.72	-0.21	4.22	37.41	2.92
2.57	-7.63	2.43	0.11	4.22	41.83	2.19
5.16	-6.12	0.67	0.48	4.22	46.95	4.67
20.81	-9.12	1.41	-0.23	4.22	37.26	2.69
4.63	-3	3	1.4	4.22	59.42	1.26
3.92	1.54	3.69	3.05	4.22	82.72	1.13
5.72	2.04	4.5	3.26	4.22	85.73	0.77
4.53	1.38	1.3	2.99	4.22	81.77	3.44
2.36	-9.01	3.63	-0.21	4.23	37.57	1.15
2.67	-1.79	3.55	1.82	4.23	64.92	1.23
11.16	-14.23	1.39	-1.17	4.23	24.75	3.06
3.85	1.6	3.86	3.08	4.23	83.03	1.04
14.74	-12.59	0.83	-0.88	4.24	28.24	3.9
3.79	-2.04	2.67	1.74	4.24	63.66	1.78
3.64	1.68	3.3	3.12	4.24	83.45	1.42
7.48	-7.31	2.4	0.23	4.24	42.81	2.18
8.47	-7.4	2.53	0.2	4.24	42.52	1.87
8.59	-7.31	2.44	0.23	4.24	42.81	1.84
10.77	3.98	3.05	4.12	4.24	98.21	1.36
13.74	-4.35	0.83	1.03	4.24	53.62	4.4
4.99	-0.85	2.72	2.15	4.25	69.43	1.9
8	-14.3	0.76	-1.17	4.25	24.56	3.56
3.32	-6.84	2.95	0.33	4.25	44.36	1.51
1.43	-8.55	3.67	-0.07	4.25	38.87	1.2
2.8	-7.47	3.41	0.2	4.25	42.28	1.37
3.84	0.16	3.07	2.53	4.25	74.72	1.57
3.3	-10.55	3.25	-0.49	4.25	33.22	1.53
3.05	0.03	2.77	2.48	4.25	74.06	1.74
3.12	0.06	2.6	2.49	4.25	74.19	2.09
2.8	-1.76	2.93	1.84	4.25	64.98	1.61
3.73	1.61	3.02	3.09	4.25	82.95	1.53

6.86	1.64	1.43	3.1	4.25	83.14	2.98
7.36	-7.09	2.13	0.29	4.25	43.5	2.39
0.05	-10.38	3.01	-0.51	4.26	33.62	1.94
4.9	-1.28	1.36	2	4.26	67.26	2.87
2.44	-8.62	3.19	-0.08	4.26	38.63	1.43
3.24	-6.87	3.16	0.33	4.26	44.24	1.44
1.72	-4.79	3.34	0.88	4.26	51.8	1.37
3.52	-0.08	2.66	2.44	4.26	73.39	1.73
3.8	0.13	2.72	2.52	4.26	74.56	1.75
9.45	-9.62	1.76	-0.28	4.26	35.72	2.56
2.98	0.05	2.48	2.49	4.26	74.11	2.01
2.65	-8.67	2.97	-0.09	4.26	38.48	1.58
3.23	-8.48	1.74	-0.04	4.26	39.05	2.98
4.52	1.4	1.42	3.02	4.26	81.67	3.2
7.15	-6.79	2.23	0.37	4.26	44.51	2.26
7.23	-6.99	2.09	0.31	4.26	43.83	2.45
12.97	-4.61	0.1	0.96	4.26	52.52	5.25
2.14	-5.09	3.07	0.8	4.27	50.64	1.63
2.3	-5.39	2.7	0.72	4.27	49.48	1.91
8.2	-7.46	3.77	0.21	4.27	42.25	1
4.68	0.19	2.45	2.55	4.27	74.78	2.07
2.69	-1.75	2.29	1.86	4.27	64.9	2.4
4.36	1.56	2.06	3.09	4.27	82.54	2.42
4.43	1.39	1.62	3.03	4.27	81.54	2.97
4.42	1.37	1.42	3.01	4.27	81.43	3.25
4.5	1.42	1.88	3.03	4.27	81.73	2.62
4.65	1.43	1.13	3.05	4.27	81.79	3.71
4.75	1.43	1.39	3.05	4.27	81.79	3.33
5.42	0.39	1.83	2.63	4.27	75.9	2.73
-1.01	-13.25	-0.26	-3.13	4.28	34.08	1.24
5.82	0.65	2.98	2.73	4.28	77.26	1.73
2.9	-1.11	2.09	2.08	4.28	67.99	2.4
2.99	-1.12	2.19	2.07	4.28	67.96	2.31
3.07	-1.18	2.21	2.05	4.28	67.65	2.37
4.39	1.45	1.62	3.05	4.28	81.84	2.88
4.51	1.4	1.61	3.03	4.28	81.59	2.95
4.57	1.41	1.28	3.04	4.28	81.57	3.5
4.62	1.43	1.09	3.05	4.28	81.68	3.76
4.68	1.43	1.19	3.04	4.28	81.71	3.62
4.71	1.39	1.14	3.03	4.28	81.48	3.7
4.73	1.43	1.4	3.04	4.28	81.73	3.31
4.73	1.43	1.32	3.05	4.28	81.76	3.42
4.78	1.43	1.5	3.04	4.28	81.73	3.27
2.79	-1.14	1.74	2.07	4.29	67.85	2.85

2.84	-1.12	1.9	2.07	4.29	67.91	2.66
2.89	-1.17	2	2.06	4.29	67.68	2.53
9.05	-0.27	2.8	2.38	4.29	72.26	1.48
9.63	-0.46	3.05	2.32	4.3	71.23	1.57
10.56	-9.61	4.4	-0.26	4.3	35.64	0.67
3.49	-7.02	2.81	0.31	4.3	43.6	1.73
9.51	-9.08	3.21	-0.14	4.3	37.15	1.24
9.51	-7.68	4.47	0.18	4.3	41.45	0.82
8.35	-7.7	2.99	0.17	4.3	41.36	1.55
3.15	-5.79	2.12	0.64	4.31	47.87	2.69
2.78	-15.89	0.89	-1.37	4.31	21.46	4.53
8.13	2.52	-0.68	3.51	4.31	88.14	7.39
5.29	-6.24	0.61	0.51	4.31	46.26	4.69
5.44	-6.73	0.66	0.38	4.31	44.55	4.64
6.42	-6.24	2.48	0.54	4.31	46.23	2.13
7.66	-7.13	2.98	0.31	4.31	43.21	1.72
2.43	-9	3.99	-0.14	4.32	37.34	1
2.56	-8.95	3.96	-0.13	4.32	37.49	1.08
2.79	-1.86	3.83	1.84	4.32	64.16	1.09
3.92	1.68	3.22	3.17	4.32	82.97	1.4
-1.25	-17.11	-1.17	-4.36	4.33	26.57	0.99
11.28	-4.8	0.78	0.93	4.33	51.49	4.2
5.1	-4.72	4.26	0.97	4.33	51.84	0.88
10.77	-13.75	2.08	-1.02	4.33	25.56	2.21
3.93	-1.49	4.07	1.98	4.33	65.93	0.94
3.2	-0.74	3.19	2.24	4.33	69.59	1.5
7.1	-7.31	2.48	0.27	4.33	42.56	2.1
11.91	-9.17	3.42	-0.14	4.34	36.79	1.19
7.18	-6.86	2.15	0.4	4.34	43.99	2.34
13.23	-4.72	0.59	0.98	4.34	51.81	4.53
1.83	-4.8	3.02	0.93	4.35	51.44	1.45
2.8	-1.73	2.68	1.91	4.35	64.64	1.9
2.83	-1.72	2.43	1.91	4.35	64.7	2.07
5.82	1.01	4.21	2.91	4.35	78.92	0.85
11.26	-14.36	2.79	-1.12	4.35	24.26	1.72
1.57	-3.97	2.35	1.18	4.35	54.76	2.57
9.88	-4.23	1.82	1.12	4.35	53.66	3.05
-0.09	-10.45	2.63	-0.47	4.36	33.24	2.03
11.16	-8.59	3.8	0.01	4.36	38.43	1.08
4.78	0.39	2.48	2.68	4.36	75.44	2.17
3.32	-0.58	3.26	2.31	4.36	70.32	1.47
8.31	-7.54	2.63	0.24	4.36	41.7	1.89
3.99	-19.05	-4.1	-6.11	4.37	26.5	1.31
7.23	-13.94	-0.75	-1.08	4.37	25.1	7.76

7.9	-7.19	1.48	0.32	4.37	42.83	3.47
3.97	0.12	4.59	2.58	4.37	73.89	0.76
7.09	-6.58	2.32	0.49	4.37	44.88	2.1
4.55	-8.33	1.15	0.05	4.38	39.18	4.14
9.58	-7.36	3.09	0.3	4.38	42.24	1.39
7.84	-1.57	3.39	1.98	4.38	65.29	1.23
4.58	-1.42	4.34	2.04	4.38	66.05	0.91
10.51	-8.98	2.11	-0.09	4.39	37.22	2.54
2.71	-7.73	3.94	0.19	4.39	41.02	0.97
3.22	-7.29	4.61	0.29	4.39	42.42	0.91
4.07	-5.18	1.59	0.86	4.39	49.85	3.34
5.6	-7.44	0.97	0.25	4.39	41.94	4.26
4.15	-1.47	4.03	2.03	4.39	65.69	1
3.61	1.53	3.94	3.14	4.39	81.65	1.2
10.68	-7.03	12.06	4.44	4.4	26.48	0.98
10.52	-8.97	1.42	-0.08	4.4	37.23	3.46
3.05	-7.37	4.54	0.31	4.4	42.15	0.81
7.36	-13.07	-0.33	-0.88	4.4	26.87	7.91
2.9	-1.84	3.76	1.9	4.4	63.89	1.49
4.4	-1.56	4.19	2	4.4	65.27	0.93
5.78	1.1	4.23	2.97	4.4	79.15	0.9
6.62	-1.86	3.13	1.9	4.4	63.84	1.59
7.56	-7.25	2.93	0.34	4.4	42.54	1.72
12.24	-4.86	1.69	0.97	4.4	51.04	3.55
14.54	-1.98	1.38	1.84	4.41	63.25	3.34
7.1	1.73	1.4	3.24	4.41	82.77	3.31
3.24	-0.72	3.57	2.3	4.41	69.33	1.24
3.37	-0.57	3.66	2.35	4.41	70.11	1.32
2.33	-5.38	2.53	0.81	4.42	49	1.86
16.39	-2.15	0.81	1.8	4.42	62.37	4.33
6.84	-5.25	2.31	0.86	4.42	49.5	2.24
2.81	-1.86	3.05	1.91	4.42	63.73	1.76
2.76	-1.9	3.34	1.89	4.42	63.56	1.45
5.75	1.53	2.06	3.16	4.42	81.47	2.76
13.45	-4.05	0.8	1.22	4.42	54.17	4.6
4.46	-6.19	2.3	0.59	4.43	46.01	1.79
14.47	-12.67	2.03	-0.79	4.43	27.71	2.72
3.51	-0.09	2.63	2.53	4.43	72.49	2
8.42	-7.37	3.16	0.33	4.43	42.07	1.6
2.25	-3.91	3.18	1.24	4.43	54.69	1.64
4.83	1.41	1.81	3.12	4.43	80.8	3.05
5.59	1.06	1.79	2.98	4.43	78.74	3.02
5.66	1.25	1.79	3.06	4.43	79.83	3.02
5.69	1.39	1.91	3.11	4.43	80.66	2.91

5.73	1.51	1.85	3.16	4.43	81.38	2.94
13.38	-4.28	0.21	1.16	4.43	53.19	5.16
8.09	-3.55	1.05	1.37	4.44	56.15	3.92
2.77	-1.77	2.19	1.95	4.44	64.04	2.49
3.56	-8.58	1.77	0.04	4.44	38.26	3.22
1.51	-13.87	2.13	-1.02	4.45	25.08	2.62
1.63	-13.83	2.17	-1.02	4.45	25.17	2.7
9.78	-0.37	2.99	2.43	4.45	70.93	1.74
8.14	-1.35	3.23	2.09	4.45	66.04	1.93
8.19	-16.85	-0.77	-1.41	4.45	19.61	7.8
2.69	-7.77	4.78	0.22	4.45	40.7	0.67
9.63	-7.53	4.11	0.3	4.45	41.47	0.89
4.9	0.43	2.09	2.74	4.45	75.16	2.68
20.36	-9.25	2.08	-0.13	4.45	36.29	2.99
2.02	-4.12	2.23	1.19	4.45	53.77	2.87
6.56	-11.32	-0.57	-0.52	4.46	30.8	7.53
4.18	-4.54	1.55	1.08	4.46	52.07	3.42
4.26	-3.88	1.85	1.28	4.46	54.69	3.03
5.02	0.69	1.8	2.85	4.46	76.53	3.07
5.73	2.14	4.81	3.43	4.46	84.88	0.7
11.72	-6.82	12.8	4.62	4.47	26.47	0.86
3.33	-16.06	0.88	-1.29	4.47	20.92	5.01
10.32	-9.1	4.51	-0.04	4.47	36.66	0.73
2.95	-7.22	2.93	0.36	4.47	42.4	1.87
3.86	0.01	0.45	2.59	4.47	72.8	5.92
8.03	-7.21	3.74	0.39	4.47	42.46	1.14
3.44	-6.53	4.35	0.54	4.47	44.75	1.08
8.04	2.68	0.15	3.67	4.47	88.15	6.03
8.03	-7.1	0.76	0.41	4.47	42.82	4.88
5.77	-7.83	0.94	0.2	4.47	40.49	4.57
8.01	-1.79	1.96	1.96	4.47	63.85	2.52
0.68	-3.82	1.1	1.3	4.47	54.92	4.19
10.56	-9.18	1.51	-0.08	4.48	36.42	3.46
4.07	-2.18	0.81	1.82	4.48	61.99	4.93
7.32	-9.82	1.36	-0.21	4.48	34.62	3.2
3.94	0.25	3.56	2.68	4.48	74.03	1.23
4.07	0.33	3.95	2.71	4.48	74.47	0.96
0.53	-3.84	0.68	1.3	4.48	54.81	4.99
11.28	-4.79	1.36	1.02	4.49	51	3.58
5.44	-0.67	2.8	2.35	4.49	69.18	1.97
8.57	-5.93	-0.33	0.71	4.49	46.79	7.69
4.8	-2.72	3.27	1.65	4.49	59.53	1.35
2.93	-1.79	4.02	1.97	4.49	63.74	1.15
0.41	-3.85	0.68	1.29	4.49	54.73	4.19

10.9	-4.45	1.97	1.14	4.49	52.27	2.95
2.22	-5.11	2.86	0.93	4.5	49.72	1.77
3.17	0.04	2.68	2.63	4.5	72.81	2.19
2.26	-3.92	2.54	1.27	4.5	54.41	1.73
2.48	-3.97	3.46	1.27	4.5	54.18	1.71
5.71	1.02	4.1	2.99	4.5	78.18	0.97
4.85	1.45	1.91	3.17	4.5	80.6	2.99
4.99	1.76	2.39	3.31	4.5	82.43	2.6
5.78	1.54	2.26	3.2	4.5	81.1	2.64
5.41	-4.87	4	1.04	4.51	50.59	1.1
3.87	-8.53	2.03	0.09	4.51	38.24	3
4.85	1.44	2.06	3.18	4.51	80.49	2.8
4.96	1.74	2.25	3.3	4.51	82.21	2.56
4.98	1.71	1.78	3.29	4.51	82.05	3.17
7.18	-11.06	-0.48	-0.44	4.52	31.29	8.04
4.4	-3.29	2.25	1.49	4.52	56.91	2.75
11.61	-14.52	2.14	-1.02	4.52	23.69	2.64
5.76	-4.97	3.04	1.02	4.52	50.22	1.9
4.86	1.43	1.95	3.18	4.52	80.41	2.95
4.87	1.45	1.7	3.18	4.52	80.46	3.3
5.71	1.46	1.81	3.19	4.52	80.57	3.02
3.93	-7.35	4.16	0.37	4.53	41.81	1.78
5.14	0.88	1.91	2.96	4.53	77.19	3.03
5.27	0.96	2.1	2.99	4.53	77.66	2.81
5.96	0.49	3.12	2.8	4.53	75.07	1.9
8.06	-1.3	4.28	2.16	4.53	65.86	1.04
1.9	-3.99	2.45	1.28	4.53	53.99	2.36
6.97	-2.44	2.95	1.78	4.53	60.59	1.77
3.19	-15.55	1.25	-1.18	4.54	21.73	4.56
8.29	2.49	0.8	3.62	4.54	86.52	5.01
1.05	-3.88	1.54	1.31	4.54	54.39	3.54
-0.1	-11.57	1.86	-0.57	4.55	30	2.76
8.21	-7.16	0.95	0.43	4.55	42.38	4.8
8.37	-5.59	0.79	0.84	4.55	47.77	4.89
7.39	-0.89	2.89	2.31	4.55	67.8	1.43
0.74	-3.77	1.01	1.36	4.55	54.8	4.38
5.7	-0.61	3.29	2.41	4.56	69.17	1.66
10.32	-8.74	1.55	0.06	4.56	37.49	3.88
14.49	-2.01	1.59	1.92	4.56	62.43	3.51
3.67	-7.17	3.43	0.43	4.56	42.33	1.74
10.31	-8.36	4.64	0.17	4.56	38.61	0.78
7.51	-3.1	0.23	1.57	4.56	57.61	6.46
4.24	-19.28	-4.39	-6.3	4.57	26.45	1.42
10.42	-9.17	4.36	0	4.57	36.22	0.98

7.26	1.76	1.75	3.34	4.57	82.01	3.14
4.73	-0.04	4.7	2.63	4.57	72.02	0.81
5.12	-9.86	1.18	-0.17	4.57	34.3	3.81
5.71	1.48	4.32	3.22	4.57	80.37	0.94
3.62	1.61	4.21	3.27	4.57	81.14	1.07
13.6	-3.84	1.45	1.37	4.57	54.45	3.58
-15.45	-31.81	-19.64	-18.49	4.58	26.44	1
2.74	-8.84	3.84	0.05	4.58	37.13	1.26
10.54	-8.27	4.09	0.2	4.58	38.82	0.93
7.28	-2.81	2.54	1.67	4.58	58.75	2.25
3.73	0.09	3.2	2.68	4.58	72.66	1.65
3.92	0.28	3.21	2.75	4.58	73.67	1.68
3.41	-10.65	3.45	-0.31	4.58	32.22	1.54
5.93	-8.12	1.25	0.2	4.58	39.27	4.39
3.44	-0.7	4.01	2.4	4.58	68.59	1.24
5.75	2.16	4.77	3.5	4.58	84.27	0.76
5.82	1.43	2.6	3.21	4.58	80.03	2.41
12.35	-4.78	1.29	1.1	4.58	50.69	4.45
13.63	-4.43	1.22	1.2	4.58	52.09	3.96
13.45	-4.25	1.23	1.25	4.58	52.78	3.8
11.47	-4.83	0.93	1.07	4.59	50.49	4.86
11.7	-8.17	0.22	0.22	4.59	39.09	6.46
7.36	-3.02	0.2	1.61	4.59	57.78	6.45
3.7	-0.06	2.75	2.63	4.59	71.86	2.34
3.77	0.12	3.32	2.69	4.59	72.78	1.52
7.5	1.73	1.69	3.33	4.59	81.71	3.49
8	-7.56	3.25	0.38	4.59	40.99	1.98
8.46	-7.42	3.33	0.42	4.59	41.43	1.8
4.9	1.6	1.85	3.29	4.59	80.98	3.17
4.92	1.62	2.11	3.3	4.59	81.07	2.78
2.01	-11.41	2.22	-0.45	4.6	30.29	2.84
-11.44	-25.13	-15.28	-14.36	4.6	34.15	1.2
10.97	-7.97	-0.03	0.28	4.6	39.67	6.4
7.65	-5.53	1.63	0.89	4.6	47.86	3.55
4.14	-8.36	1.64	0.19	4.6	38.49	3.65
1.9	-3.87	2.79	1.36	4.6	54.2	2.4
2.19	-17.15	1.63	-2.96	4.61	22.69	0.92
1.69	-8.51	4.02	0.15	4.61	38.04	1.19
5.4	1.21	2.23	3.14	4.61	78.62	2.99
12.34	-9.12	0.07	0.01	4.61	36.25	7.54
1.27	-3.83	2.23	1.37	4.61	54.35	2.71
10.86	4.22	3.31	4.43	4.61	97.32	1.49
10.78	-10.54	3.25	-0.72	4.62	34.18	1.23
8.57	-1.59	1.65	2.11	4.62	64.14	3.35

4.8	-1.31	4.2	2.22	4.62	65.43	1.07
1.22	-3.85	2.1	1.37	4.62	54.23	2.86
4.5	-18.95	-4.08	-5.99	4.63	26.42	1.26
8.11	-17.6	-0.39	-1.4	4.63	18.2	7.5
1.19	-3.83	1.62	1.38	4.63	54.26	3.47
2.95	-11.55	1.61	-0.49	4.64	29.86	3.63
5.98	-0.52	3.21	2.49	4.64	69.19	1.9
4.17	-0.49	0.68	2.51	4.64	69.36	5.13
3.57	-0.42	0.9	2.52	4.64	69.75	5.26
8.15	2.56	1.57	3.71	4.64	86.41	3.75
8.34	2.76	0.56	3.79	4.64	87.59	5.42
1.63	-3.9	2.49	1.36	4.64	53.95	2.51
12.97	-5.16	2	1.03	4.64	49.07	3.54
3.07	-11.61	1.33	-0.49	4.65	29.71	4.62
3.83	-7.39	4.09	0.45	4.65	41.35	1.16
8.95	-5.91	-0.23	0.81	4.65	46.31	8.64
7.67	-3.02	-0.06	1.64	4.65	57.57	7.06
7.4	-3	0.28	1.65	4.65	57.65	6.33
5.45	-9.27	1.12	0	4.65	35.71	4.31
3.65	1.61	4.53	3.32	4.65	80.67	0.97
8.65	-14.99	1.35	-1.02	4.66	22.55	3.78
8.89	-1.66	1.42	2.1	4.66	63.58	4.09
8.7	-1.59	1.54	2.14	4.66	63.92	3.61
3.17	-10.61	3.01	-0.81	4.67	34.26	1.39
3.51	-0.09	2.92	2.66	4.67	71.24	1.97
7.94	-7.3	0.95	0.48	4.67	41.57	4.74
5.01	1.7	2.48	3.37	4.67	81.09	2.76
2.17	-11.42	2.42	-0.4	4.68	30.08	2.77
8.59	-3.21	1.67	1.61	4.68	56.63	3.45
7.79	1.71	1.59	3.38	4.68	81.09	3.71
4.96	-6.2	2.48	0.74	4.69	45.17	2.66
0.14	-10.06	2.94	-0.19	4.69	33.49	2.3
3.48	-5.59	2.76	0.92	4.69	47.32	2.24
9.98	-0.19	3.46	2.63	4.69	70.68	1.66
8.25	2.48	1.17	3.71	4.69	85.56	4.3
11.78	-10.16	-0.45	-0.17	4.69	33.21	9.22
10.1	-9.38	-0.1	0	4.69	35.32	6.5
10.3	-9.66	2.13	-0.03	4.69	34.56	2.53
8.79	-1.68	1.63	2.13	4.69	63.36	3.66
2.77	-0.15	3.11	2.65	4.69	70.88	1.84
3.52	-0.58	3.79	2.5	4.69	68.67	1.47
5.83	1.43	2.76	3.27	4.69	79.4	2.29
7.58	-12.9	3.45	-0.65	4.7	26.68	1.63
5.04	-8.18	1.53	0.28	4.7	38.75	4.02

8.05	-18.96	-0.04	-1.51	4.7	16.13	7.52
4.5	-2.81	2.53	1.75	4.7	58.29	2.57
2.79	-3.67	2.45	1.47	4.7	54.62	2.69
10.62	-4.06	2.3	1.38	4.7	53.06	2.64
5.02	-8.05	4.37	0.29	4.71	39.14	0.83
8.42	-6.77	1.45	0.63	4.71	43.18	4.23
2.77	-7.78	1.96	0.36	4.71	39.95	3.3
9.71	3.32	0.68	4.07	4.71	90.76	6.05
-2.97	-14.93	-2.13	-4.76	4.73	34.31	1.01
2.64	-7.82	4.14	0.37	4.73	39.77	1.02
6.23	-3.93	4.12	1.42	4.74	53.45	0.89
3.79	1.68	4.66	3.4	4.74	80.55	0.88
3.83	1.71	4.57	3.41	4.74	80.72	0.93
2.85	-14.14	2.76	-1.67	4.75	26.4	1.16
3.77	-5.46	2.58	0.99	4.75	47.59	2.61
11.26	-11.06	2.75	-0.27	4.75	30.8	2.24
9.48	-1.37	4.33	2.25	4.75	64.59	0.88
4	1.71	4.5	3.4	4.75	80.69	1.05
3.8	1.75	4.42	3.42	4.75	80.91	1
3.92	1.78	4.72	3.44	4.75	81.11	0.86
8.33	0.83	1.8	3.07	4.75	75.74	3.55
8.05	1.09	1.98	3.17	4.75	77.21	3.14
8.43	-7.61	3.97	0.47	4.76	40.35	1.46
16.31	-1.94	1.03	2.06	4.76	61.85	4.71
8.09	-7.4	0.96	0.51	4.76	41.01	4.95
5.11	-8.84	1.61	0.18	4.76	36.67	4.05
3.98	1.8	4.29	3.45	4.76	81.17	1.12
3.51	-0.13	2.95	2.7	4.77	70.56	2.08
4.89	-8.43	1.28	0.27	4.77	37.82	4.41
1.73	-3.73	3.52	1.5	4.77	54.15	1.65
2.24	-4.37	3.32	1.31	4.77	51.6	1.92
6.02	-4.87	3.52	1.19	4.77	49.68	1.63
5.85	1.47	2.6	3.33	4.77	79.25	2.47
5.89	1.45	2.41	3.32	4.77	79.11	2.81
4.15	0.05	2.99	2.77	4.77	71.51	2.19
2.44	-11.39	2.83	-0.33	4.78	29.94	2.44
8.22	2.36	1.16	3.7	4.78	84.3	4.68
6.76	-7.95	2.4	0.35	4.78	39.24	3.11
6.86	0.15	4.42	2.83	4.78	71.99	0.82
1.88	-8.53	3.62	0.25	4.78	37.52	1.53
2.14	-4.12	2.82	1.38	4.78	52.56	2.29
2.2	-4.21	3.22	1.36	4.78	52.21	1.9
6.22	-4.83	3.51	1.21	4.78	49.82	1.7
7.62	-13.9	-0.06	-0.82	4.79	24.46	7.06

14.06	-13.16	2.83	-0.65	4.79	25.95	2.63
2.89	-7.83	2.23	0.39	4.79	39.57	3.3
6.51	-7.38	1.92	0.5	4.79	40.98	3.47
1.81	-3.79	2.93	1.49	4.79	53.83	1.98
6.31	-8.14	1.63	0.32	4.8	38.61	3.94
8.2	-1.1	4.25	2.39	4.8	65.61	0.96
8.09	-12.62	2.29	-0.54	4.81	27.07	2.77
7.98	-13.16	0.67	-0.64	4.81	25.92	6.51
6.12	-8.27	1.33	0.3	4.81	38.22	4.49
19.69	-9.36	2.44	0.07	4.81	35.09	3.49
6.33	-2.05	2.61	2.07	4.81	61.18	2.27
5.27	-8.73	1.59	0.23	4.81	36.86	4.12
5.37	-8.62	1.41	0.25	4.81	37.19	4.48
7.84	-3.16	0.09	1.7	4.82	56.31	7.05
-11.43	-24.99	-15.88	-14.3	4.83	34.39	1.43
9.1	-5.1	0.98	1.14	4.83	48.66	4.9
3.86	1.74	4.34	3.47	4.83	80.41	1.06
-0.91	-15.76	-4.18	-5.52	4.84	34.4	1.44
3.67	-15.72	1.3	-1.02	4.84	20.98	4.87
3.76	-7.18	4.36	0.59	4.84	41.47	1.25
8.25	1.82	1.46	3.5	4.84	80.8	4.23
7.93	1.83	1.64	3.51	4.84	80.86	3.7
5.26	-6.25	3.21	0.81	4.85	44.51	2.05
5.52	-8.29	1.78	0.34	4.85	38.01	3.9
6.48	4.66	-0.97	4.76	4.85	98.7	11.94
7.41	-2.89	0.15	1.8	4.85	57.29	7.66
8.99	-1.43	1.57	2.3	4.85	63.82	3.93
8.59	1.8	1.1	3.5	4.85	80.66	5.1
6.38	-9.35	1.47	0.12	4.85	35.01	4.82
6.44	-4.84	3.7	1.25	4.85	49.55	1.65
7.93	-14.1	0.31	-0.8	4.86	23.93	7.03
3.38	-1.13	3.12	2.39	4.86	65.23	2.15
5.32	-8.88	1.42	0.22	4.86	36.29	4.58
1.96	-3.96	2.71	1.48	4.86	52.86	3.02
13.51	-4.55	0.92	1.33	4.86	50.6	4.43
3.66	-16.67	1.29	-1.13	4.87	19.33	4.76
7.56	-19.06	0.01	-1.41	4.87	15.8	8.22
4.64	-2.51	2.5	1.94	4.87	58.89	3
7.94	2.58	1.5	3.83	4.87	85.13	4.24
8.08	2.58	1.67	3.83	4.87	85.16	3.89
2.87	-0.07	2.77	2.79	4.87	70.42	2.51
4.87	-0.04	5.01	2.79	4.87	70.53	0.94
8.43	-7.24	3.37	0.62	4.87	41.18	1.55
2.72	-3.78	2.94	1.53	4.87	53.59	2.48

4.16	-0.2	2.49	2.75	4.87	69.72	2.69
4.13	-5.45	2.73	1.06	4.88	47.2	2.57
7.84	-19.1	0.09	-1.42	4.88	15.74	7.41
8.62	-6.89	1.84	0.7	4.88	42.28	3.93
12.86	-8.79	-0.09	0.24	4.88	36.5	7.52
10.75	-9.82	-0.52	0.02	4.88	33.7	9.08
20.49	-8.06	2.46	0.4	4.88	38.62	2.57
5.58	-1.46	4.93	2.31	4.88	63.54	0.84
4.67	0.03	5.33	2.83	4.88	70.84	0.63
2.63	-3.8	2.71	1.53	4.88	53.44	2.54
-1.25	-17.2	-0.94	-4.38	4.89	26.39	1.01
3.21	-11.55	1.79	-0.34	4.89	29.33	4.09
10.03	-0.08	3.35	2.78	4.89	70.25	1.8
4.2	-7.24	4.65	0.63	4.89	41.15	1.03
5.53	1.04	2.21	3.22	4.89	76.16	3.44
19.14	-8.7	1.79	0.26	4.89	36.74	3.93
5.99	-9.07	1.36	0.2	4.89	35.68	4.88
3.03	-1.63	3.18	2.25	4.9	62.65	2.13
0.87	-3.83	2.27	1.54	4.9	53.25	2.93
10.13	-8.64	1.94	0.3	4.91	36.87	3.21
6.15	0.13	3.93	2.88	4.91	71.24	1.51
3.55	-0.76	4.4	2.57	4.91	66.73	1.12
3.85	1.64	4.61	3.47	4.91	79.37	1.04
2.67	-11.34	2.98	-0.24	4.92	29.77	2.5
11.29	-4.51	1.55	1.35	4.92	50.52	4.42
11.35	-4.78	1.65	1.28	4.92	49.5	4.19
9.13	-15.1	1.67	-0.88	4.92	21.95	3.91
3.31	0.11	3.28	2.89	4.92	71.04	2.04
3.34	0.12	3.45	2.89	4.92	71.13	1.92
9.16	-1.55	1.56	2.3	4.92	62.96	4.44
4.52	-0.8	3.32	2.55	4.92	66.54	1.71
3.64	-0.58	4.3	2.63	4.92	67.63	1.24
4.37	0.07	5.33	2.86	4.92	70.85	0.86
3.88	1.77	4.41	3.53	4.92	80.07	1.04
8.58	0.66	1.95	3.1	4.92	73.92	3.43
13.46	-4.45	1.74	1.39	4.92	50.78	3.62
5.11	-0.89	1.81	2.53	4.93	66.03	3.23
12.8	-12.35	2.91	-0.4	4.93	27.45	2.53
4.22	0.2	3.85	2.92	4.93	71.44	1.54
5.48	-6.09	3.31	0.91	4.94	44.76	2.07
6.05	-8.19	1.78	0.42	4.94	38.08	4.05
3.82	-0.23	0.76	2.76	4.94	69.26	6.3
3.23	-12.03	3.51	-0.36	4.94	28.13	1.89
3.48	-0.06	3.16	2.83	4.94	70.1	1.99

12.44	-14.16	2.69	-0.71	4.94	23.69	2.44
5.81	-9.06	1.16	0.23	4.94	35.6	5.15
7.37	-4.64	3.17	1.36	4.94	49.97	2.17
4.21	0.27	3.35	2.96	4.94	71.8	1.92
4.53	0.16	2.82	2.92	4.94	71.22	2.62
7.5	-1.87	3	2.2	4.94	61.41	1.95
13.61	-4.66	1.26	1.35	4.94	49.91	4.36
8.01	-10.96	0.12	-0.16	4.95	30.63	7.47
9.21	-2.87	1.51	1.87	4.95	56.99	4.04
3.44	-1.09	3.09	2.46	4.95	65	2.27
6.26	-1.23	5.06	2.43	4.95	64.32	0.84
5.5	-8.63	1.46	0.34	4.95	36.78	4.54
1.66	-3.57	3.43	1.65	4.95	54.09	1.74
5.95	1.47	3.06	3.43	4.95	78.24	2.38
4.13	-0.22	2.88	2.79	4.95	69.23	2.29
4.41	0.13	2.91	2.91	4.95	71.02	2.43
-11.89	-25.14	-15.42	-14.45	4.96	34.4	1.23
5.88	-16.54	0.49	-1.06	4.96	19.43	7.15
7.97	2.52	1.58	3.86	4.96	84.24	4.25
9.35	-1.49	5.35	2.33	4.96	63.05	0.83
5.33	0.61	1.25	3.09	4.96	73.51	5.36
6.39	-7.51	3.77	0.61	4.96	40.08	1.67
2.59	-3.85	3.13	1.56	4.96	52.97	2.07
8.29	-12.97	2.86	-0.5	4.97	26.03	2.59
4.14	-16.74	1.03	-1.08	4.97	19.09	5.56
4.62	-17.07	0.73	-1.12	4.97	18.56	6.56
5.49	-16.59	0.09	-1.06	4.97	19.34	8.1
14.12	-13.04	2.39	-0.52	4.97	25.87	3.18
20.53	-8.56	2.48	0.34	4.97	36.94	2.52
6.7	-1.89	2.81	2.2	4.97	61.19	2.36
5.34	-7.46	3.81	0.63	4.97	40.2	1.61
0.24	-9.85	1.7	0.03	4.98	33.37	3.66
14.73	-1.78	2.36	2.24	4.98	61.64	2.77
8.02	-3.29	0.15	1.75	4.98	55.14	7.55
9.49	-0.29	2.92	2.76	4.99	68.71	2.52
9.45	-8.61	2.25	0.35	4.99	36.73	3.34
5.05	-7.47	3.97	0.64	4.99	40.14	1.46
6.74	-13.77	3.77	-1.32	5	26.38	0.84
4.66	-7.58	4.68	0.59	5	39.78	2.21
7.5	-2.99	0.28	1.85	5	56.32	7.75
25.46	-6.59	14.4	6.01	5.01	22.78	1.45
3.1	-1.66	4.42	2.31	5.01	62.07	1.21
9.3	-1.49	1.69	2.37	5.01	62.87	4.14
3.74	-0.73	4.11	2.63	5.01	66.44	1.39

6.47	-0.32	4.35	2.76	5.01	68.46	1.21
7.37	0.03	4.98	2.91	5.01	70.2	0.78
4.07	1.78	4.68	3.58	5.01	79.65	1.07
5.01	2.08	3.39	3.71	5.01	81.35	2.03
5.03	2.16	3.28	3.75	5.01	81.82	2.18
5.81	-17.07	0.56	-2.93	5.02	22.79	1.25
15.71	-1.79	1.53	2.27	5.02	61.45	4.23
6.27	-0.35	3.26	2.77	5.02	68.24	2.37
3.04	-0.06	3.47	2.87	5.02	69.67	2.06
8.35	-7.92	1.29	0.54	5.02	38.7	4.75
7.49	-7.42	4.13	0.66	5.02	40.21	1.53
3.04	-8.42	3.93	0.4	5.02	37.22	1.6
8.35	-1.19	4.16	2.47	5.03	64.17	1.66
3.14	-1.63	3.07	2.33	5.03	62.11	2.47
7.84	-7.32	3.83	0.7	5.03	40.48	1.44
3.8	-0.69	4.17	2.66	5.03	66.56	1.41
6.83	-0.4	4.88	2.75	5.03	67.99	1.01
6.19	-9.07	1.62	0.28	5.03	35.36	4.5
6.8	-4.71	3.76	1.38	5.03	49.43	1.6
4.31	0.17	3.26	2.97	5.03	70.82	2.06
4.57	0.18	2.93	2.98	5.03	70.87	2.49
4.6	0.25	2.91	3	5.03	71.21	2.47
4.56	0.23	3.22	2.99	5.03	71.18	2.07
4.56	-5.42	2.78	1.17	5.04	46.76	2.77
9.61	-0.36	3.77	2.77	5.04	68.08	1.71
3.87	-7.09	2.32	0.73	5.04	41.17	2.35
6.85	-7.42	4.19	0.68	5.04	40.15	1.37
11.35	-10.75	0.02	-0.07	5.04	30.95	8.17
13.2	-14.11	2.36	-0.64	5.04	23.61	3.02
5.37	0.46	1.44	3.08	5.04	72.27	5.24
6.28	-9.17	1.05	0.27	5.04	35.03	5.31
7.7	-4.74	2.44	1.38	5.04	49.26	3.2
9.95	-0.13	3.55	2.86	5.05	69.23	1.97
5.84	-7.5	4.26	0.66	5.05	39.88	1.32
9.97	3.6	0.89	4.38	5.05	90.35	6.6
20.16	-8.92	2.66	0.3	5.05	35.7	2.5
5.59	-7.37	4.03	0.7	5.05	40.27	1.51
0.23	-10.42	2.33	-0.04	5.06	31.72	3.34
0.27	-9.76	2.1	0.1	5.06	33.41	3.9
0.29	-9.97	2.06	0.05	5.06	32.86	3.84
9.75	-8.6	2.69	0.39	5.06	36.59	2.66
4.8	-7.37	4.45	0.71	5.06	40.24	1.17
12.4	-8.41	0.74	0.45	5.06	37.13	6.14
7.88	-2.11	2.45	2.17	5.06	59.84	2.66

6.26	0	4.44	2.92	5.07	69.77	1.36
12.38	-4.83	2.18	1.38	5.07	48.79	4.05
11.82	-4.11	2.88	1.58	5.07	51.54	2.6
3.35	-11.59	2.34	-0.23	5.08	28.86	3.46
5.07	-17.02	0.64	-1.05	5.08	18.51	6.8
8.84	-6.69	1.91	0.87	5.08	42.34	4.3
3.74	-0.15	4.07	2.87	5.08	68.96	1.52
18.59	-7.94	1.69	0.55	5.08	38.47	4.33
9.42	-1.64	2.1	2.36	5.08	61.86	3.77
-2.01	-19.13	-3.01	-6.11	5.09	26.29	0.83
9.55	-15.19	1.78	-0.79	5.09	21.53	4.29
8.31	-7.4	4.75	0.71	5.09	40.07	1.04
6.69	4.97	0.02	5.03	5.09	99.15	10.22
3.37	-1.76	5.15	2.31	5.09	61.24	0.92
3.58	0.09	3.68	2.97	5.09	70.13	1.86
4.73	-0.58	3.52	2.72	5.09	66.8	1.85
4.13	1.78	4.65	3.62	5.09	79.2	1.13
10.46	-4.31	1.59	1.54	5.09	50.72	5.11
7	-8.35	2.07	0.47	5.1	37.2	3.87
7.46	-8.44	1.84	0.45	5.1	36.95	4.31
8.7	-4.92	10.42	4.13	5.1	34.45	1.42
8.51	2.8	2.9	4.06	5.1	85.14	2.71
3.19	-1.64	4.44	2.37	5.1	61.78	1.23
9.37	-1.37	1.89	2.45	5.1	63	3.92
8.6	-1.36	5.05	2.47	5.1	63.03	0.92
9.15	1.88	1.61	3.67	5.1	79.7	4.68
9.03	1.86	1.69	3.67	5.1	79.59	4.49
4.08	1.88	4.83	3.67	5.1	79.73	0.98
5.04	2.1	3.52	3.77	5.1	80.95	1.96
5.08	2.24	3.68	3.83	5.1	81.79	1.81
5.08	2.2	3.62	3.81	5.1	81.51	1.9
4.68	0.17	3.43	3.01	5.1	70.5	2.15
8.38	-2.09	3.43	2.22	5.1	59.77	2.02
5.57	-6.09	3.33	1	5.11	44.25	2.33
4.92	-1.55	3.61	2.4	5.11	62.12	1.89
15.18	-2.14	1.61	2.2	5.11	59.54	4.33
8.25	2.84	2.14	4.08	5.11	85.29	3.7
3.6	-1.03	3.79	2.57	5.11	64.55	1.74
9.24	1.89	1.34	3.68	5.11	79.73	5.24
8.83	1.85	1.7	3.66	5.11	79.48	4.34
8.74	1.92	1.47	3.69	5.11	79.87	4.53
8.7	1.98	1.35	3.72	5.11	80.18	4.63
5.36	-2.26	3.5	2.15	5.11	58.94	1.64
10.95	-4.96	1.45	1.36	5.11	48.18	5.21

3.93	-0.69	3.76	2.7	5.11	66.19	1.74
6.28	-16.59	0.88	-0.97	5.12	19.13	6.87
3.48	-7.23	3.35	0.74	5.12	40.52	2.42
4.72	-2.21	2.95	2.18	5.12	59.17	2.59
8.14	2.68	2.05	4.02	5.12	84.23	3.74
9.66	-2.91	1.61	1.95	5.12	56.2	4.39
6.98	-1.25	4.84	2.51	5.12	63.51	1.06
9.92	-0.01	4.38	2.94	5.12	69.44	1.03
9.44	0.36	1.2	3.1	5.12	71.37	5.74
8.96	0.45	1.24	3.13	5.12	71.84	5.47
5.65	1.1	2.25	3.38	5.13	75.22	3.78
7.85	-1.34	5.06	2.49	5.13	62.98	0.97
9.73	-0.27	4.14	2.85	5.13	68.15	1.42
0.33	-10.07	2.35	0.08	5.14	32.44	3.65
4.81	-2	3.43	2.26	5.14	60	2.11
6.63	-7.55	4.05	0.71	5.15	39.44	1.5
8.23	-3.51	0.49	1.78	5.15	53.61	7.23
9.08	-1.25	5.37	2.51	5.15	63.35	0.81
4.08	-15.84	1.52	-0.84	5.16	20.32	5.33
10.37	-4.36	1.37	1.56	5.16	50.27	5.44
8.34	-8.33	2.42	0.52	5.17	37.09	3.38
9.23	-5.74	0.27	1.16	5.17	45.27	8.59
7.71	-3.01	0.41	1.94	5.17	55.57	8.26
5.88	1.71	3.73	3.65	5.17	78.38	1.89
1.22	-13.45	-1.07	-3.43	5.18	34.48	1.37
3.16	-8.17	4.24	0.56	5.18	37.51	1.49
9.69	1.85	1.84	3.7	5.18	79.08	4.58
4.2	1.85	5.02	3.7	5.18	79.08	0.96
5.47	-2.25	3.03	2.2	5.18	58.76	2.31
0.12	-11.28	2.01	-0.13	5.19	29.36	3.49
3.92	-0.28	0.65	2.88	5.19	67.8	7.42
3.21	-1.75	3.72	2.37	5.19	60.89	1.87
13.48	-12.68	3	-0.3	5.19	26.23	2.54
3.66	-0.99	3.98	2.62	5.19	64.37	1.63
3.43	-1.63	4.17	2.42	5.19	61.46	1.5
8.24	-1.26	4.97	2.55	5.19	63.1	1.1
9.43	-1.24	5.04	2.54	5.19	63.24	0.86
3.1	0.13	4.13	3.03	5.19	69.88	1.62
2.83	-6.71	4.23	0.92	5.19	41.94	1.52
5.89	1.73	3.41	3.66	5.19	78.41	2.17
5.94	1.72	2.85	3.67	5.19	78.3	2.9
8.79	-2.17	3.26	2.25	5.19	59.01	2.22
8.44	-13.6	0.73	-0.52	5.2	24.34	7.26
8.16	-14.03	0.68	-0.58	5.2	23.5	6.7

8.37	-7.3	4.57	0.8	5.2	40.08	1.01
5.19	-1.54	3.27	2.45	5.2	61.77	2.48
3.78	-1.04	3.44	2.61	5.2	64.12	2.28
7.17	-1.34	5.51	2.53	5.2	62.68	0.75
8.15	-1.26	4.77	2.56	5.2	63.08	1.08
5.78	1.02	2.49	3.38	5.2	74.4	3.21
4.46	0.36	6.14	3.13	5.2	71	0.43
5.01	-5.28	2.7	1.3	5.21	46.73	3.17
5.05	-1.46	3.1	2.48	5.21	62.12	2.53
10.09	-3.18	1.32	1.92	5.21	54.72	5.27
14.49	-12.86	3.77	-0.35	5.21	25.83	1.55
7.02	-8.54	2.85	0.48	5.21	36.39	2.97
3.57	-1.58	4.51	2.44	5.21	61.58	1.29
9.5	-1.71	2.13	2.4	5.21	60.98	4.03
4.29	1.85	4.97	3.72	5.21	78.89	1.04
9.38	-1.83	1.54	2.38	5.21	60.47	5.16
9.17	0.5	1.29	3.2	5.21	71.64	5.39
1.58	-17.24	1.81	-3.08	5.22	22.8	0.77
2.21	-13.82	2.56	-0.55	5.22	23.87	3.48
7.06	-7.27	4.16	0.82	5.22	40.11	1.36
1.89	-3.8	2.32	1.73	5.22	52.17	3.61
3.5	-11.51	2.43	-0.12	5.23	28.75	3.69
10.02	-0.05	3.47	2.99	5.23	68.71	1.79
8.79	-12.12	0.91	-0.21	5.23	27.36	7
14.24	-13.4	3.86	-0.39	5.23	24.68	2.24
5.04	-0.34	3.68	2.88	5.23	67.31	1.9
0.32	-10.5	1.59	0.05	5.24	31.13	4.9
4.5	0.04	5.51	3.02	5.24	69.19	0.74
1.7	-3.8	1.54	1.74	5.24	52.09	4.01
9.42	-1.55	1.82	2.48	5.24	61.58	4.79
5.87	1.74	3.78	3.69	5.24	78.12	1.85
8.72	-8.32	2.56	0.57	5.25	36.91	3.13
6.74	-0.35	4.11	2.89	5.25	67.17	1.63
5.92	1.68	4.41	3.69	5.25	77.76	1.35
8.87	-13.22	4.46	-0.78	5.26	26.24	0.91
3.99	0.22	4.18	3.11	5.26	69.92	1.56
5.42	0.53	2.21	3.22	5.26	71.54	4.07
3.4	-0.25	4.56	2.94	5.26	67.62	1.46
2.71	-6.74	4.8	0.96	5.27	41.65	1.1
2.67	-6.66	4.33	0.98	5.27	41.86	1.41
3.6	-0.1	4.23	3	5.27	68.33	1.57
4.06	0.23	4.08	3.12	5.27	69.98	1.62
4.16	0.28	3.63	3.14	5.27	70.23	1.96
3.8	-1.5	4.29	2.51	5.27	61.65	1.46

9.56	1.9	1.84	3.77	5.27	78.88	4.5
3.15	0.19	4.1	3.1	5.27	69.73	1.69
2.53	-6.81	4.29	0.94	5.27	41.41	1.61
5.44	-2.34	2.8	2.22	5.27	57.95	2.88
11.02	-4.86	1.57	1.46	5.28	48.01	5.07
5.72	-8.14	5.33	0.61	5.28	37.34	0.74
3.88	-1.05	3.41	2.66	5.28	63.66	2.45
6.14	1.25	2.95	3.51	5.28	75.23	2.88
7.16	-0.26	5.1	2.93	5.28	67.43	1
5.34	2.27	3.28	3.94	5.28	80.95	2.47
5.37	2.29	3.44	3.94	5.28	81.06	2.36
9.19	-2.22	3.11	2.28	5.28	58.43	2.43
5.34	-17.41	0.57	-3.27	5.29	22.87	1.16
15	-1.99	1.28	2.35	5.29	59.4	5.68
3.32	-12.02	2.81	-0.15	5.29	27.49	3.16
3.5	0.02	3.44	3.04	5.29	68.84	2.1
4.07	-11.08	4.36	0.04	5.29	29.62	1.52
4.35	0.29	5.46	3.15	5.29	70.15	0.85
1.98	-3.84	2.61	1.76	5.29	51.81	3.16
9.67	-0.12	1.57	3.02	5.29	68.08	4.91
6.04	1.89	2.68	3.79	5.29	78.72	3.24
5.72	-17.11	0.77	-3.01	5.3	22.9	1.22
7.03	-19.5	1.04	-1.2	5.3	14.78	6.26
7.57	-8.58	3.02	0.52	5.3	36.04	2.75
7.88	-8.48	2.82	0.55	5.3	36.34	3.07
2.08	-3.97	2.92	1.72	5.3	51.26	3.01
2.11	-3.74	2.37	1.79	5.3	52.13	3.71
2.04	-3.88	2.54	1.75	5.3	51.61	3.35
2.47	-4.17	3.52	1.67	5.3	50.47	2.47
-5.28	-23.76	-8.28	-9.14	5.31	22.93	1.45
0.34	-10.41	2.71	0.11	5.31	31.2	3.34
0.14	-11.11	2.56	-0.03	5.31	29.5	3.38
7.32	-18.53	0.94	-1.09	5.31	16.05	6.1
5.79	0.85	2.07	3.38	5.31	72.95	4.47
10.34	3.93	1.66	4.67	5.31	90.8	5.5
3.64	-11.52	2.46	-0.08	5.32	28.54	3.66
4.01	-0.33	0.95	2.94	5.32	66.91	7.52
10.4	-3	1.49	2.04	5.32	55.05	5.27
9.04	-6.73	1.7	1	5.32	41.54	5.24
7.08	-1.63	2.9	2.5	5.32	60.86	2.61
6.83	-0.33	4.23	2.95	5.33	66.91	1.56
7.74	0.19	5.59	3.14	5.33	69.44	0.63
3.25	-7.9	4.52	0.71	5.34	37.9	1.4
6.42	0.24	3.94	3.16	5.34	69.72	1.71

6.87	-0.36	3.91	2.94	5.34	66.72	1.87
7.82	-2.55	0.54	2.18	5.34	56.81	8.48
9.42	-1.36	2.27	2.6	5.34	61.97	3.68
8.67	-8.45	1.82	0.61	5.34	36.32	4.57
10.45	2.6	1.98	4.11	5.35	82.44	4.67
3.64	-6.33	3.97	1.11	5.35	42.74	1.79
10.12	1.99	1.81	3.85	5.35	78.93	4.89
9.96	1.96	1.95	3.84	5.35	78.76	4.49
5.39	-0.1	3.04	3.04	5.35	67.95	2.88
3.33	-0.15	4.63	3.02	5.35	67.67	1.32
5.98	1.78	4.26	3.77	5.35	77.81	1.4
4.82	0.13	4.08	3.14	5.35	69.08	1.8
8.62	-12.86	4.18	-0.25	5.36	25.57	1.78
5.54	-5.35	3.32	1.37	5.36	45.98	2.51
3.86	-7.16	3.96	0.9	5.36	40.07	1.84
8.14	-1.58	3.9	2.53	5.36	60.9	1.74
7.69	-7.21	4.74	0.92	5.36	39.92	1.07
14.85	-14.4	3.04	-0.47	5.36	22.55	2.66
3.18	0.21	4.09	3.17	5.36	69.44	1.7
3.21	0.1	4.22	3.12	5.36	68.91	1.58
6.17	1.4	2.68	3.62	5.36	75.64	3.31
5.51	0.33	2.51	3.21	5.36	70.06	3.69
3.27	0.05	4.34	3.1	5.36	68.66	1.49
4.4	1.86	4.71	3.81	5.36	78.21	1.29
2.96	-6.63	4.19	1.04	5.36	41.73	1.8
10.59	-5.35	2.26	1.37	5.37	45.96	4.16
8.74	-11.33	0.72	0.02	5.37	28.89	6.86
5.35	-1.56	3.34	2.54	5.37	61.01	2.67
3.5	-0.04	4.2	3.07	5.37	68.13	1.66
4.34	-1.32	3.87	2.62	5.37	62.06	1.95
7.4	-1.06	5.43	2.72	5.37	63.25	0.8
10.29	-2.19	3.43	2.34	5.37	58.22	2.22
9.56	-2.24	3.16	2.32	5.37	57.99	2.54
5.2	-16.44	1.95	-2.45	5.38	23	0.88
7.29	-8.75	3.24	0.53	5.38	35.38	2.57
2.21	-3.81	2.71	1.82	5.38	51.56	3.32
0.42	-9.71	2.28	0.3	5.39	32.78	4.28
9.4	-12.96	1.32	-0.26	5.39	25.29	6.6
7.09	-3.35	4.18	1.97	5.39	53.36	1.48
8.24	-0.22	4.63	3.02	5.39	67.18	1.42
3.05	-8.88	2.97	0.53	5.39	34.98	3.04
2.13	-3.95	2.83	1.78	5.39	51.01	3.15
2.21	-3.83	2.63	1.82	5.39	51.48	3.44
4.54	-15.82	2.03	-0.69	5.4	19.99	5.08

8.13	-0.27	4.76	3	5.4	66.87	1.21
5.7	-2.08	4.13	2.38	5.41	58.52	1.42
4.54	-1.25	4.14	2.68	5.41	62.22	1.79
8.13	-0.07	5.61	3.1	5.41	67.83	0.75
8.97	-6.08	1.96	1.24	5.41	43.38	4.75
12.03	-4.3	2.23	1.73	5.41	49.61	3.92
9.44	-5.67	0.62	1.32	5.42	44.72	8.62
18.01	-7.81	1.97	0.78	5.42	37.94	4.4
3.28	-9.02	2.96	0.52	5.42	34.53	3.13
0.38	-9.78	2.6	0.31	5.43	32.5	3.73
4	-7.4	3.94	0.89	5.43	39.12	2.06
3.37	-7.74	4.98	0.8	5.43	38.12	1.18
10.75	3.03	2.51	4.34	5.43	84.56	3.34
6.93	-0.34	3.9	3	5.43	66.4	1.96
6.98	-0.44	3.67	2.96	5.43	65.89	2.24
5.54	0.18	2.9	3.19	5.43	68.95	3.12
4.78	1.94	4.97	3.87	5.43	78.28	1.2
4.69	1.98	5.13	3.89	5.43	78.42	1.04
1.16	-17.22	1.8	-3.15	5.44	23.01	0.74
10.94	-5.46	2.08	1.39	5.44	45.37	4.46
3.53	-1.68	3.94	2.54	5.44	60.15	1.94
3.8	-6.55	4.48	1.11	5.44	41.77	1.57
4.06	-1.03	3.91	2.75	5.44	63.1	2.14
4.7	-1.13	4.07	2.73	5.44	62.62	1.94
8.8	-9.01	1.96	0.54	5.44	34.53	4.52
4.61	1.93	4.78	3.87	5.44	78.14	1.24
5.64	-5.91	3.34	1.24	5.45	43.83	2.65
5.65	-12.65	5.12	-0.28	5.45	26.18	1.01
5.32	-5.85	3.41	1.27	5.45	44.01	1.81
6.03	-5.41	2.88	1.4	5.45	45.52	3.27
11	-3.31	2.07	2.01	5.45	53.29	4.71
4.46	1.87	4.26	3.85	5.45	77.76	1.64
4.49	1.88	4.16	3.85	5.45	77.78	1.73
5.39	2.45	3.46	4.1	5.45	80.97	2.42
5.44	2.31	3.53	4.04	5.45	80.18	2.39
-5.94	-21.77	-5.3	-7.35	5.46	23.1	1.23
6.67	-18.35	1.45	-0.97	5.46	16.12	5.64
14.47	-9.87	1.08	0.35	5.46	32.23	6.67
8.36	-8.34	3.16	0.67	5.46	36.33	2.98
3.99	-0.95	3.53	2.79	5.46	63.39	2.37
8.51	-0.2	4.95	3.06	5.46	66.97	1.25
6.31	2.38	2.7	4.07	5.46	80.58	3.59
11.13	-14.4	4.59	-0.72	5.47	23.11	1.09
2.52	-13.81	3.1	-0.4	5.47	23.49	3.18

3.66	-7.2	3.18	0.95	5.47	39.64	2.89
9.49	2.87	2.02	4.28	5.47	83.37	4.62
10.66	-4.06	0.4	1.81	5.47	50.3	9.15
3.56	-12.09	2.62	-0.05	5.47	26.98	3.97
13.97	-9.95	0.88	0.34	5.47	31.99	6.79
5.01	0.24	5.37	3.23	5.47	69.02	0.96
3.79	-11.36	2.51	0.05	5.48	28.59	4.12
0.34	-10.58	2.64	0.18	5.48	30.42	3.47
0.42	-10.81	2.63	0.13	5.48	29.86	3.56
0.44	-10.85	2.5	0.13	5.48	29.77	3.94
5.92	0.79	1.67	3.45	5.48	71.79	5.83
9.3	-6.7	1.96	1.1	5.48	41.14	5.18
10.02	-3.56	0.37	1.96	5.48	52.22	8.81
10.46	2.03	2.01	3.94	5.48	78.43	4.81
8.15	-0.14	4.39	3.09	5.48	67.14	1.47
8.19	-0.11	4.52	3.1	5.48	67.28	1.41
23.14	-6.2	14.86	6.18	5.49	23.21	1.35
-0.12	-18.99	-3.06	-5.92	5.49	26.13	0.95
15.01	-1.68	2.68	2.56	5.49	59.93	2.9
17.56	-8.26	2.68	0.71	5.49	36.46	3.67
7.8	-0.19	5.33	3.08	5.49	66.86	0.94
9.31	-0.04	4.28	3.13	5.49	67.56	1.36
4.16	-0.25	0.74	3.07	5.5	66.53	8.65
8	-3	0.79	2.13	5.5	54.34	8.21
9.62	-1.61	2.64	2.6	5.5	60.19	3.65
12.91	-5.14	2.55	1.54	5.5	46.27	3.96
12.67	-4.72	2.18	1.66	5.5	47.75	3.97
10.39	-15.51	2.44	-0.58	5.51	20.38	4.07
3.65	-1.71	3.98	2.56	5.51	59.73	2.06
13.31	-13.31	3.64	-0.24	5.51	24.4	2.58
9.53	-1.31	2.68	2.71	5.51	61.5	3.63
9.57	-1.36	2.47	2.69	5.51	61.3	3.79
13.48	-4.84	2.34	1.63	5.51	47.34	4.5
4.93	0.17	4.14	3.23	5.51	68.52	1.88
6.87	-11.65	5.84	0.68	5.52	25.96	1.5
7.57	-13.75	3.61	-1.18	5.52	26	1.15
10.29	-5.4	2.46	1.45	5.52	45.33	4.01
6.29	-0.72	2.58	2.91	5.52	64.19	3.37
15.47	-2.12	2.13	2.44	5.52	57.94	4.98
7.03	-0.48	3.33	3	5.52	65.29	2.76
3.96	-0.07	3.64	3.14	5.52	67.29	2.18
6.21	1.24	3.06	3.64	5.52	73.95	3.19
3.47	-0.07	4.63	3.14	5.52	67.29	1.43
6.55	-5.31	2.75	1.48	5.53	45.57	3.64

10.96	0.96	3.57	3.54	5.53	72.39	2.07
4.91	-1.08	3.83	2.8	5.53	62.5	2.27
10.7	-2.24	3.65	2.41	5.53	57.37	2.26
8.67	-12.74	4.12	-0.12	5.54	25.49	1.96
-15.25	-33.1	-18.91	-18.52	5.54	23.26	0.82
2.21	-14.49	3.54	-1.81	5.54	25.92	0.68
6.09	1.01	2.74	3.56	5.54	72.62	4.12
10.68	4.03	1.73	4.83	5.54	89.96	6.71
2.55	-4.17	3.54	1.8	5.54	49.66	2.65
8.64	-14.12	0.79	-0.39	5.55	22.78	7.37
3.78	-7.26	3.31	0.99	5.55	39.23	2.83
4	-11.43	2.54	0.09	5.56	28.26	4.36
3.27	-7.79	2.46	0.85	5.56	37.62	4.68
13.53	-9.37	1.29	0.54	5.57	33.26	6.45
4.21	-0.41	1.18	3.05	5.58	65.39	7.57
8.13	-2.74	1.08	2.26	5.58	55.08	7.71
4.19	-0.1	4.21	3.17	5.58	66.83	1.83
17.15	-7.88	2.59	0.85	5.58	37.32	3.78
4.72	0.3	6.29	3.33	5.58	68.82	0.64
4.99	0.01	4.59	3.22	5.58	67.39	1.56
25.71	-6.43	14.41	5.93	5.59	23.4	1.21
7.09	-0.58	3.73	3	5.59	64.52	2.4
12.03	-13.34	2.31	-0.2	5.59	24.19	4.49
3.84	-6.54	4.61	1.19	5.59	41.34	1.54
9.9	-5.16	2.16	1.57	5.59	45.93	3.75
10.34	2.14	2.18	4.05	5.59	78.46	4.34
13.54	-4.66	2.54	1.73	5.59	47.68	3.92
13.51	-4.6	2.53	1.74	5.59	47.91	4.31
13.17	-4.09	2.5	1.89	5.59	49.76	3.9
7.24	-13.92	3.09	-1.29	5.6	25.9	1.35
3.04	-14.29	2.52	-1.63	5.6	25.91	1.4
3.87	-7.41	3.78	0.97	5.6	38.63	2.37
3.47	-7.61	5.14	0.94	5.6	38.05	1.14
6.98	5.52	0.86	5.56	5.6	99.49	9.08
4.84	-11.55	4.63	0.14	5.6	27.92	1.51
9.07	-1.41	2.74	2.72	5.6	60.66	3.8
6.25	1.38	3.5	3.74	5.6	74.28	2.57
6.21	1.34	3.43	3.73	5.6	74.06	2.72
8.96	-9.63	2.35	0.51	5.6	32.52	4.18
4.8	1.94	5.51	3.97	5.6	77.32	0.94
4.82	1.96	5.33	3.97	5.6	77.43	1.03
12.68	-5.22	2.32	1.58	5.6	45.7	4.7
12.81	-4.93	2.21	1.65	5.6	46.7	4.96
7.06	-5.43	2.95	1.5	5.61	44.93	3.56

3.82	-1.69	4.58	2.62	5.61	59.41	1.69
10.89	0.25	4.21	3.32	5.61	68.46	1.87
5.5	-1.47	4.06	2.7	5.61	60.41	2.07
3.47	0	4.36	3.22	5.61	67.23	1.72
5.14	-1.07	4.04	2.85	5.61	62.17	2.12
8.69	-1.02	5.3	2.85	5.61	62.43	0.89
10.63	2.15	1.88	4.05	5.61	78.41	5.26
10.6	2.18	1.81	4.07	5.61	78.58	5.24
3.72	-7.67	4.39	0.93	5.61	37.87	1.78
6	-2.18	2.97	2.46	5.61	57.27	2.86
11.89	-5.02	2.18	1.63	5.61	46.35	4.94
12.68	-4.84	2.11	1.68	5.61	46.97	4.68
11.44	-2.13	3.94	2.49	5.61	57.5	2.11
9.97	-5.5	2.61	1.48	5.62	44.66	3.97
4.06	-7.46	3.77	0.98	5.62	38.45	2.6
11.02	-0.14	3.8	3.18	5.62	66.5	2.25
13.05	-8.17	1.26	0.83	5.62	36.38	5.89
15.75	-11.86	1.44	0.07	5.62	27.21	7.02
3.5	0.04	4.11	3.24	5.62	67.34	1.92
8.94	-0.07	5.67	3.2	5.62	66.81	0.81
8.65	-12.54	4.24	-0.03	5.63	25.75	1.75
0.45	-10.88	2.6	0.21	5.63	29.41	4.15
5.38	0.77	6.25	3.52	5.63	70.93	0.72
3.02	-6.52	4.21	1.22	5.63	41.31	1.92
6.47	2.16	2.87	4.08	5.63	78.39	3.84
10	-13.16	1.65	-0.15	5.64	24.47	6.23
10.58	-13.4	1.35	-0.18	5.64	24	7.05
5.24	0.43	5.8	3.39	5.64	69.2	0.8
6.14	-2.34	3.8	2.43	5.64	56.53	2.07
0.47	-10.92	2.4	0.21	5.65	29.26	4.67
0.23	-10.99	2.89	0.2	5.65	29.1	3.2
3.52	-8.15	2.94	0.84	5.65	36.36	3.6
12.95	-12.97	3.29	-0.1	5.65	24.82	2.78
4.35	-0.05	4.38	3.23	5.65	66.76	1.71
13.51	-5.12	3.4	1.63	5.65	45.88	3.08
-1.35	-17.28	-1.46	-4.28	5.66	25.88	1.38
15.35	-1.66	2.34	2.67	5.66	59.31	3.96
9.62	-5.81	0.91	1.43	5.66	43.51	9.24
5.01	-0.11	4.91	3.21	5.66	66.45	1.47
11.08	-6.21	2.84	1.35	5.66	42.22	3.92
2.3	-14.54	3.08	-1.84	5.67	25.86	0.93
10.6	-15.64	2.73	-0.5	5.67	19.92	3.79
3.92	-6.56	5.36	1.24	5.67	41.05	1.1
7.14	-0.63	4.27	3.02	5.67	63.91	1.87

7.17	-0.79	4.1	2.97	5.67	63.18	2.04
7.23	-1.13	4.51	2.86	5.67	61.62	1.79
8.26	-2.36	1.23	2.43	5.67	56.3	7.42
5.01	0.01	4.72	3.26	5.67	66.96	1.5
4.81	1.96	5.33	4.01	5.67	77.09	1.07
3.11	-6.74	4.13	1.2	5.68	40.48	2.14
8.86	-5.41	2.8	1.55	5.69	44.73	3.82
4.06	-7.39	4.12	1.04	5.69	38.46	2.46
5.68	-1.39	4.84	2.77	5.69	60.4	1.51
14.23	-8.67	1.87	0.76	5.69	34.84	5.6
9.56	-6.74	2.11	1.2	5.69	40.45	5.48
7.21	-12.81	4.27	-0.05	5.69	25.1	1.95
5.13	-11.73	4.9	0.16	5.69	27.37	1.53
5.52	-12.01	4.75	0.11	5.69	26.75	1.54
5.39	-0.67	4.25	3.03	5.69	63.66	1.95
5.25	1.87	4.79	3.98	5.69	76.42	1.48
5.06	1.9	5.11	4	5.69	76.65	1.22
4.83	1.97	5.08	4.03	5.69	76.98	1.24
8.02	-5.43	2.53	1.55	5.7	44.64	4.33
9.3	-4.95	2.61	1.67	5.7	46.3	4.08
4.54	-7.45	3.58	1.03	5.7	38.28	2.34
9.48	-11.28	1.61	0.22	5.7	28.33	5.69
5.36	1.85	4.56	3.98	5.7	76.31	1.69
10.64	2.24	2.06	4.14	5.7	78.46	5.02
3.67	-0.29	4.25	3.17	5.7	65.39	2.07
5.69	0.31	5.26	3.39	5.7	68.31	1.22
9.04	-9.6	1.9	0.56	5.7	32.38	5.74
3.87	-7.64	4.69	0.98	5.7	37.7	1.59
5.75	-5.79	4.16	1.43	5.71	43.42	2.1
7.56	-5.25	2.63	1.6	5.71	45.24	4.14
8.83	-8.12	3.65	0.87	5.71	36.3	2.68
5.45	1.84	4.39	3.98	5.71	76.21	1.89
5.43	0.13	5.89	3.33	5.71	67.42	0.78
3.61	-9.21	3.87	0.65	5.71	33.37	2.54
2.74	-4.68	3.99	1.76	5.71	47.22	2.3
-5.42	-23.59	-8.32	-9.19	5.72	23.43	1.41
2.74	-13.78	3.35	-0.24	5.72	23.14	3.31
10.18	2.59	2.59	4.3	5.72	80.34	3.89
12.34	-4.3	0.73	1.88	5.72	48.58	9.24
11.84	-4.2	0.93	1.9	5.72	48.93	8.65
3.73	-12.13	3.2	0.09	5.72	26.44	3.22
3.85	-12.09	3.55	0.1	5.72	26.53	2.8
11.82	-12.79	1.29	-0.04	5.72	25.07	8.01
-15.43	-32.27	-20.85	-18.71	5.73	25.78	1.34

5.08	-15.69	2.5	-0.47	5.73	19.77	5.06
3.15	-4.07	2.34	1.94	5.73	49.37	5.18
3.42	-8.28	2.94	0.85	5.73	35.79	3.61
3.44	-8.24	3	0.86	5.73	35.91	3.41
3.5	-8.12	2.92	0.89	5.73	36.25	3.51
3.6	-8.09	2.84	0.9	5.73	36.34	3.82
11.2	-12.75	1.48	-0.03	5.73	25.13	6.86
2.63	-4.18	3.56	1.91	5.73	48.99	2.71
2.67	-4.35	3.61	1.86	5.73	48.37	2.6
0.45	-10.92	2.48	0.27	5.74	29.08	4.68
14.99	-2.13	3.39	2.54	5.74	57	2.83
8.72	-0.95	3.62	2.96	5.74	62.14	1.9
3.61	-8.16	2.96	0.89	5.74	36.13	3.66
5.7	-11.81	4.46	0.17	5.74	27.1	1.88
5.64	1.81	4.2	3.98	5.74	75.91	2.15
10.99	-13.98	5.02	-0.48	5.75	23.44	0.88
8.39	-2.28	1.47	2.5	5.75	56.32	6.97
4.28	-0.48	1.43	3.12	5.76	64.24	7.46
16.31	-11.49	1.67	0.21	5.76	27.75	7.15
-6.62	-21.66	-4.26	-7.41	5.77	23.52	0.77
7.95	-2.93	3.91	2.32	5.77	53.64	2.14
12.47	-4.67	2.51	1.83	5.78	47.06	4.26
11.7	-3.23	2.48	2.23	5.79	52.36	4.37
15.52	-10.4	1.88	0.45	5.79	30.2	6.38
4.44	-0.92	4.22	2.98	5.79	62.04	2.18
5.54	1.89	4.33	4.05	5.79	76.06	1.99
11.74	-1.86	3.72	2.68	5.79	57.91	2.56
11.25	-14.16	4.88	-0.68	5.8	23.54	1.1
8.77	-13.45	1.62	-0.14	5.8	23.64	6.31
10.68	2.17	2.28	4.17	5.8	77.51	4.73
5.19	-11	4.71	0.36	5.81	28.75	1.68
3.9	-6.35	4	1.34	5.81	41.31	2.45
12.58	-13.25	4.11	-0.04	5.81	23.99	2.33
12.82	-13.02	1.47	-0.02	5.81	24.46	7.88
5.42	0.18	5.76	3.41	5.81	67.16	0.86
3.4	-8.38	2.82	0.88	5.82	35.31	3.88
3.69	-8.12	3.18	0.94	5.82	36.04	3.56
5.81	1.87	4.45	4.06	5.82	75.76	2
5.11	0.33	6.1	3.46	5.82	67.86	0.73
0.75	-17.13	1.35	-3.28	5.83	23.54	0.84
4.19	-11.29	2.79	0.27	5.83	28.07	4.53
0.38	-10.94	2.53	0.31	5.83	28.84	4.33
14.61	-14.64	3.03	-0.24	5.83	21.4	3.32
4.42	0.21	4.44	3.42	5.83	67.19	2.08

5.64	-11.15	4.03	0.35	5.84	28.35	2.31
5.82	-11.16	3.65	0.34	5.84	28.32	2.74
4.31	-17.32	0.28	-3.46	5.84	23.55	1.17
7.29	-1.26	4.66	2.89	5.84	60.34	1.77
8.51	-2.14	1.68	2.6	5.84	56.56	7.14
11.11	-13.22	3.58	-0.02	5.84	24.02	2.96
15.17	-11.1	1.61	0.34	5.84	28.47	6.1
5.17	0.07	5.71	3.37	5.84	66.46	0.95
11.6	-13.02	3.23	0.02	5.85	24.4	3.29
14.47	-8.66	2.64	0.83	5.85	34.46	4.73
5.79	0.4	5.11	3.51	5.85	68.04	1.39
4.02	-7.71	4.62	1.06	5.85	37.11	1.85
13.83	-4.58	2.95	1.9	5.85	47.13	3.76
13.8	-3.94	2.33	2.08	5.85	49.45	4.57
-6.5	-21.54	-4.55	-7.34	5.86	23.59	0.86
10.88	-15.24	2.69	-0.33	5.86	20.34	4.35
7.8	-12.58	3.92	0.1	5.86	25.24	2.52
5.88	-0.73	4.33	3.11	5.86	62.62	2.12
8.8	-1.08	3.3	2.98	5.86	61.09	3.28
10.93	1.95	3.05	4.11	5.86	75.99	3.86
10.83	2.11	2.96	4.18	5.86	76.89	3.89
11.47	-5.34	2.91	1.69	5.86	44.49	3.81
0.31	-17.12	1.53	-3.29	5.87	23.6	0.75
14.98	-9.13	2.1	0.77	5.87	33.2	5.65
6.01	-11.68	4.64	0.27	5.87	27.14	1.83
4.24	-0.02	4.37	3.35	5.87	65.88	1.96
5.82	-11.68	4.45	0.28	5.88	27.11	1.97
9.34	-8.32	3.92	0.92	5.88	35.35	2.68
3.97	-0.21	4.11	3.3	5.88	64.98	2.24
5.84	-1.25	4.2	2.93	5.89	60.18	2.24
9.87	2.34	2.53	4.28	5.89	77.96	4.82
12.38	-13.15	1.8	0.01	5.89	24.06	6.42
5.57	-0.16	4.43	3.31	5.89	65.15	2.48
4.65	-11.51	4.53	0.32	5.89	27.45	1.72
3.8	-0.23	4.09	3.29	5.89	64.86	2.31
6.54	-11.18	4.6	0.38	5.9	28.17	2.03
7.15	5.85	1.02	5.88	5.9	99.65	9.36
6.37	1.6	2.65	3.99	5.9	73.93	4.48
9.86	-6.38	2.76	1.43	5.9	40.96	4.63
13.54	-4.59	0.88	1.9	5.9	46.93	9.54
12.98	-4.28	0.81	1.99	5.9	48.05	9.15
16.68	-7.96	3.12	1.02	5.9	36.3	3.3
3.75	-8.05	3.14	1.01	5.91	36.02	3.8
4.2	-8.85	3.51	0.83	5.91	33.82	3.21

3.45	-7.85	2.93	1.04	5.91	36.57	4.74
9.12	-1.49	4.51	2.87	5.92	59.02	1.61
3.35	-8.38	3.03	0.94	5.92	35.05	3.81
15.44	-8.21	3.17	0.97	5.92	35.54	3.85
16.11	-7.87	3.06	1.06	5.92	36.48	3.65
10.77	2.24	2.5	4.26	5.92	77.24	4.41
3.88	-9.31	4.38	0.75	5.92	32.59	2.19
14.91	-2	3.39	2.69	5.93	56.81	3.41
10.78	-11.35	2.4	0.34	5.93	27.74	5.5
3.24	-3.1	6.26	2.34	5.93	52.33	0.75
8.63	-1.71	3.36	2.81	5.93	58.05	3.41
11.27	-6.43	1.85	1.43	5.94	40.7	6.97
5.9	0.39	4.82	3.56	5.94	67.56	1.73
7.37	-11.4	5.28	0.37	5.95	27.58	1.53
10.15	-11.12	2	0.41	5.95	28.21	5.49
16.89	-2.33	2.52	2.61	5.95	55.38	5.02
6.21	-11.59	5.05	0.33	5.95	27.18	1.68
10.94	2.06	2.86	4.2	5.95	76.1	4.26
10.93	1.95	2.9	4.16	5.95	75.54	4.17
6.68	-2.75	3.63	2.47	5.95	53.64	2.5
4.04	-6.49	4.67	1.42	5.96	40.46	1.84
5.89	-5.68	4.12	1.61	5.97	43.01	2.71
4.45	-7.51	4.28	1.18	5.97	37.37	1.95
5.15	-5.34	4.09	1.72	5.97	44.12	2.58
4.13	-1.51	4.76	2.89	5.97	58.71	1.8
6.03	-1.33	4.22	2.95	5.97	59.49	2.46
10.84	2.4	2.57	4.36	5.97	77.89	4.48
12.26	-3.78	2.07	2.17	5.97	49.62	5.51
3.87	-6.7	4.34	1.35	5.97	39.8	2.31
4.05	-12.08	3.92	0.25	5.97	26.09	2.71
3.33	-3.08	5.7	2.37	5.97	52.28	1.02
4.85	-0.86	4.35	3.1	5.97	61.54	2.2
6.08	-0.65	4.53	3.19	5.97	62.53	2
6.6	1.64	3.3	4.05	5.97	73.78	4.08
4.32	-8.95	3.79	0.86	5.98	33.4	2.86
4.55	-0.84	4.37	3.11	5.98	61.62	2.12
12.14	-1.59	3.87	2.88	5.98	58.34	2.58
3.28	-8.38	3.3	0.98	5.99	34.87	3.57
4.09	-8.7	3.36	0.92	5.99	34.05	3.44
4.2	-12.14	3.72	0.25	5.99	25.94	2.91
4.08	-6.51	4.98	1.44	5.99	40.31	1.61
5.28	-11.98	4.8	0.29	5.99	26.28	1.94
4.44	-9.06	4.02	0.84	6	33.06	2.73
7.36	-1.39	4.92	2.95	6.01	59.07	1.64

3.27	-8.36	3.36	0.99	6.01	34.91	3.49
10.7	-12.93	4.2	0.12	6.01	24.29	2.34
15.26	-2.06	3.23	2.72	6.02	56.22	3.45
4.42	-0.42	1.38	3.29	6.02	63.37	8.65
16.76	-2.42	2.67	2.63	6.02	54.7	4.79
8.42	-12.77	4.4	0.16	6.02	24.6	2.25
9.05	-9.77	2.5	0.72	6.02	31.25	4.96
-1.16	-18.46	-2.99	-5.31	6.03	25.76	1.36
4.14	-7.48	5.26	1.22	6.03	37.32	1.42
16.05	-11.33	1.77	0.41	6.03	27.59	6.03
16.76	-13.48	4.48	0.08	6.03	23.2	2.45
-16.09	-33.6	-19.23	-19.18	6.04	23.6	0.67
4.32	-7.44	4.97	1.23	6.04	37.38	1.58
4.31	-7.57	5.46	1.2	6.04	37.02	1.32
4.61	-3.98	2.07	2.14	6.04	48.66	6.4
7.25	6	1.34	6.02	6.04	99.75	8.56
16.7	-1.69	2.82	2.88	6.04	57.67	3.78
9.97	-5.47	1.59	1.73	6.04	43.5	8.34
15.54	-9.19	2.25	0.86	6.04	32.64	5.7
13.29	-12.06	1.37	0.28	6.04	26.01	8.37
6.24	1.97	4.77	4.22	6.04	75.14	1.89
6.35	1.92	4.74	4.19	6.04	74.89	1.94
4.77	0.1	4.45	3.49	6.04	65.72	2.31
6.21	1.52	4.5	4.04	6.04	72.77	2.05
4.16	-9.44	4.67	0.8	6.04	32.02	2.07
9.01	-9.8	2.69	0.73	6.04	31.13	4.79
10.87	-5.74	3.08	1.69	6.04	42.6	3.54
5.99	0.33	4.73	3.59	6.04	66.82	1.95
12.56	-4.15	2.27	2.11	6.05	47.99	5.51
9.02	-12.66	3.85	0.2	6.05	24.76	2.9
11.21	4.42	2.91	5.28	6.05	89.29	5.83
7.7	0.57	4.41	3.67	6.05	67.94	1.79
6.59	-1.15	3.67	3.07	6.05	59.93	3.14
7.17	-1.27	3.45	3.02	6.05	59.44	3.47
7.39	-1.21	3.34	3.04	6.05	59.73	3.71
4.51	-0.01	4.17	3.46	6.05	65.13	2.49
6.25	1.52	4.5	4.05	6.05	72.74	2.06
4.7	-4.7	1.89	1.95	6.06	46.01	6.94
4.38	-1.37	4.53	2.99	6.06	58.99	2.08
8.96	-0.29	5.54	3.37	6.06	63.77	1.15
14.85	-1.93	3.2	2.8	6.07	56.55	3.66
4.25	-7.42	4.45	1.24	6.07	37.39	2.38
11.14	0.06	4.42	3.5	6.07	65.41	2.15
6.27	1.5	3.06	4.04	6.07	72.5	4.03

9.85	2.62	2.73	4.5	6.07	78.56	4.51
4.4	-12.04	3.89	0.31	6.07	26.01	2.94
13.77	-12.33	1.79	0.25	6.07	25.38	7.33
3.53	-2.96	5.79	2.47	6.07	52.41	1.16
4.14	-6.53	4.99	1.47	6.07	40.02	1.69
4.46	-1.31	3.83	3.01	6.07	59.19	2.82
5.56	-16.11	2.96	-0.32	6.08	18.64	4.7
3.22	-4.38	2.9	2.04	6.08	47.08	4.91
3.24	-8.25	3.46	1.06	6.08	35.04	3.51
3.27	-8.34	3.39	1.04	6.08	34.79	3.57
3.99	-8.19	3.09	1.08	6.08	35.19	4.27
3.98	-8.59	3.33	0.99	6.08	34.12	3.65
4.41	-11.19	3.45	0.45	6.09	27.78	3.74
4.79	-5.07	2.21	1.86	6.09	44.67	6.46
3.92	-8.12	3.16	1.1	6.09	35.37	4.02
4.39	-1.29	3.86	3.03	6.09	59.17	2.91
9.11	-1.53	3.02	2.96	6.09	58.14	4.02
9.61	-12.78	4.35	0.2	6.1	24.45	2.41
10.67	-4.98	3.05	1.91	6.1	44.94	3.67
10.5	-8.61	3.53	0.98	6.1	34.03	3.49
11.19	-15.96	3.24	-0.28	6.11	18.83	3.82
4.45	-0.61	1.6	3.27	6.11	62.11	8.09
4.53	-1.77	1.42	2.87	6.11	57.08	8.68
4.55	-2.74	1.36	2.56	6.11	53.12	8.86
4.65	-1.4	5.27	3.01	6.11	58.6	1.57
6.19	1.51	4.59	4.07	6.11	72.39	2.01
-6.04	-21.59	-5.68	-7.4	6.12	23.64	1.33
4.53	-7.61	5.52	1.24	6.12	36.72	1.32
7.38	6.11	1.47	6.12	6.12	99.92	8.86
9.05	-10.62	2.4	0.62	6.12	29	4.89
11.92	-8.77	3.15	0.97	6.12	33.54	4.31
13.81	-8.96	3.22	0.92	6.12	33.08	3.83
8.2	-0.84	3.8	3.21	6.12	61	2.85
8.53	-0.15	5.94	3.45	6.12	64.15	0.72
4.24	-6.57	4.86	1.5	6.13	39.73	1.81
4.33	-6.68	5.02	1.47	6.13	39.43	1.67
11.24	-8.93	3.24	0.94	6.13	33.11	3.89
6.76	-0.92	3.78	3.18	6.13	60.63	2.96
7.59	-1.16	3.58	3.11	6.13	59.6	3.29
8.01	-1.15	4.18	3.11	6.13	59.63	2.56
11	1.99	2.96	4.27	6.13	74.8	4.29
4.56	-0.07	4.16	3.48	6.13	64.52	2.48
7.41	-0.49	2.97	3.34	6.14	62.51	3.96
6.39	-1.4	4.47	3.03	6.14	58.49	2.39

16.25	-9.28	2.29	0.9	6.14	32.19	6.12
9.48	-0.62	2.61	3.28	6.14	61.97	4.61
15.59	-12.27	4.19	0.35	6.14	25.39	1.85
9.9	-8.2	4.08	1.1	6.14	35.04	2.65
9	-10.13	2.5	0.72	6.14	30.12	5.38
9.02	-14.36	1.81	-0.07	6.15	21.43	6.75
5.29	-5.35	3.87	1.82	6.15	43.55	3.38
10.5	2.13	3.06	4.34	6.15	75.48	4.17
3.23	-4.18	2.36	2.14	6.15	47.59	5.9
16.5	-12.34	1.94	0.3	6.15	25.24	6.77
5.4	-11.87	4.6	0.41	6.15	26.2	1.86
11.59	-4.75	3.08	2.02	6.15	45.58	3.48
12.66	-1.53	4.21	2.99	6.15	57.89	2.41
0.94	-20.1	-4.48	-6.8	6.16	25.75	0.95
-1.79	-14.05	-1.84	-3.98	6.16	34.49	1.45
14.35	-12.36	1.81	0.3	6.16	25.17	7.63
14.97	-11.72	1.87	0.41	6.16	26.52	7.65
4.55	-1.28	3.99	3.06	6.16	58.95	2.94
3.34	-6.81	4.19	1.45	6.16	38.95	2.65
6.2	-1.44	3.95	3.02	6.17	58.21	2.93
16.71	-8.81	1.63	1.01	6.17	33.33	7.94
4	-6.26	3.83	1.57	6.17	40.58	3.27
3.16	-7.86	3.18	1.2	6.17	35.91	4.46
3.19	-8.23	3.52	1.12	6.17	34.9	3.57
3.17	-8.22	3.6	1.12	6.17	34.9	3.31
3.25	-8.3	3.4	1.11	6.17	34.68	3.63
4.55	-9.17	4.02	0.92	6.17	32.41	2.94
10.17	-12.71	4.38	0.26	6.17	24.46	2.27
2.96	-5.01	4.87	1.93	6.17	44.63	1.74
11.29	-11.11	1.86	0.54	6.18	27.79	6.68
3.42	-3.82	2.45	2.26	6.18	48.77	5.93
3.2	-4.02	2.56	2.2	6.18	48.04	5.55
4.04	-8.35	2.85	1.1	6.18	34.53	4.88
4.02	-8.47	3.01	1.07	6.18	34.22	4.42
14.68	-12.08	2.06	0.36	6.18	25.71	6.66
4.36	-1.16	3.54	3.11	6.18	59.38	3.55
7.46	-1.77	5.85	2.91	6.18	56.77	1.11
4.92	0.34	4.53	3.66	6.18	66.2	2.34
6.04	0.48	4.6	3.71	6.18	66.88	2.22
10	-1.46	4.37	3.03	6.19	58.01	2.24
8.74	-2.07	1.57	2.81	6.19	55.48	8.78
4.56	0.1	4.27	3.57	6.19	65.02	2.04
15.26	-14.04	3.34	0.07	6.19	21.93	3.25
11.04	1.96	3.86	4.3	6.19	74.34	3.29

11.46	-15.86	3.41	-0.21	6.2	18.87	3.66
4.54	-0.89	1.23	3.22	6.2	60.47	9.72
10.99	2.07	3.34	4.34	6.2	74.9	4.13
4.39	-6.68	5.51	1.52	6.21	39.2	1.38
6.53	1.69	4.8	4.19	6.21	72.78	1.97
6.68	1.48	4.81	4.11	6.21	71.72	1.99
7.52	-2.37	4.09	2.74	6.21	54.18	2.81
3.28	-4.68	2.86	2.03	6.21	45.62	5.06
6.08	0.21	4.96	3.63	6.21	65.5	1.91
6.14	-5.84	4.39	1.72	6.22	41.76	1.88
4.12	-7.35	4.58	1.35	6.22	37.19	2.05
11.2	2.05	3.29	4.35	6.22	74.63	3.99
7.49	-2.08	4.16	2.84	6.22	55.37	2.7
12.77	-4.7	2.93	2.05	6.22	45.53	4.89
4.34	-7.07	3.98	1.41	6.22	38.01	3.18
6.73	1.92	4.57	4.29	6.22	73.99	2.26
4.88	0.29	4.4	3.66	6.22	65.79	2.43
4.97	0.35	4.61	3.68	6.22	66.12	2.27
6.68	1.2	4.07	4.02	6.22	70.26	3.08
4.73	-0.97	3.62	3.21	6.23	60.02	3.57
3.66	-2.84	5.08	2.59	6.23	52.3	1.69
4.58	-11.22	3.95	0.53	6.24	27.43	3.3
14.31	-4.14	1.14	2.22	6.24	47.43	9.47
3.99	-6.85	4.23	1.47	6.24	38.59	2.47
3.18	-8.17	3.36	1.17	6.24	34.87	3.9
4.67	-9.21	3.86	0.95	6.24	32.14	3.33
17.26	-13.62	3.98	0.18	6.24	22.62	2.57
3.78	-2.93	4.55	2.56	6.24	51.89	2.18
2.91	-4.94	4.62	1.99	6.24	44.64	2.21
3.26	-13.49	3.93	0.12	6.25	22.84	3.18
16.5	-12.49	1.99	0.33	6.25	24.75	7.02
4.64	-1.06	3.61	3.19	6.25	59.51	3.57
7.87	-9.49	4.98	0.88	6.26	31.4	1.8
4.89	-5.83	2.61	1.76	6.26	41.68	6.08
5.05	-5.53	2.61	1.83	6.26	42.64	5.89
5.09	-5.88	2.85	1.74	6.26	41.5	4.89
3.15	-8.13	3.26	1.19	6.26	34.94	4.15
7.66	-3.01	5.7	2.56	6.26	51.51	1.26
14.36	-6.7	14.26	5.61	6.27	23.65	1.19
3.56	-3.85	2.35	2.31	6.27	48.38	6.17
3.28	-4.76	2.66	2.04	6.27	45.18	5.61
3.29	-5.01	2.84	1.97	6.27	44.34	5.28
7.75	-3.03	5.5	2.57	6.28	51.37	1.46
7.72	-3.5	3.63	2.42	6.28	49.61	3.25

4.49	-6.75	5.57	1.55	6.29	38.75	1.39
6.84	1.6	4.98	4.2	6.29	71.96	2.02
7.53	-2.82	4.29	2.64	6.29	52.13	2.68
10.95	1.99	3.78	4.36	6.29	74	3.48
5.98	1.54	4.86	4.18	6.29	71.62	1.85
5.63	0.54	6.23	3.8	6.29	66.68	1.07
5.48	0.59	6.02	3.82	6.29	66.93	1.11
9.17	-14.19	2.2	0.05	6.3	21.5	6.6
7.59	6.3	2.02	6.3	6.3	100	7.74
7.54	-2.77	4.22	2.66	6.3	52.31	2.76
13.09	-4.23	2.97	2.23	6.3	46.88	4.65
7.57	-2.99	5.71	2.59	6.3	51.46	1.25
9.94	-1.5	2.8	3.08	6.3	57.43	5.34
7.05	1.98	4.91	4.36	6.3	73.92	1.95
7.56	2	4.63	4.37	6.3	74	2.36
7.41	1.97	4.73	4.35	6.3	73.84	2.25
11.04	2.22	3.37	4.46	6.3	75.15	3.77
4.44	-9.54	5.13	0.93	6.3	31.22	1.81
7.31	2.39	6.84	4.53	6.3	76.1	0.67
4.35	-7.36	5.1	1.4	6.31	36.93	1.89
5.97	0.12	4.3	3.64	6.31	64.59	3.55
4.66	-11.97	4.84	0.47	6.31	25.72	2.36
16.21	-13.07	4.21	0.31	6.31	23.53	2.2
7.57	1.98	4.48	4.36	6.31	73.84	2.49
5.07	0.47	4.32	3.77	6.31	66.26	2.66
6.01	1.4	4.3	4.13	6.32	70.82	2.78
4.19	-6.95	4.12	1.49	6.32	38.09	2.97
4.06	-6.26	4.23	1.66	6.32	40.15	3.01
3.44	-6.86	4.63	1.53	6.32	38.36	2.39
6.13	-12.66	4.83	-0.14	6.33	25.73	1.28
4.32	-5.86	3.86	1.77	6.33	41.39	3.56
15.7	-11.33	2.27	0.58	6.33	27.03	6.73
16.06	-11.89	2.17	0.48	6.33	25.85	6.83
6.6	-1.36	4.33	3.15	6.34	57.87	2.79
3.18	-7.71	3.46	1.34	6.34	35.87	4.07
3.19	-7.75	3.16	1.32	6.34	35.77	4.43
3.19	-8.16	3.19	1.23	6.34	34.64	4.56
7.49	-2.97	6.05	2.62	6.34	51.41	1.08
4.83	-1.53	3.61	3.08	6.34	57.18	3.89
7.48	2.67	5.81	4.66	6.34	77.34	1.34
13.25	-1.58	4.7	3.08	6.34	56.95	2.36
7.84	-3.03	5.47	2.61	6.35	51.12	1.54
4	-3.81	2.72	2.36	6.35	48.24	5.65
3.77	-3.75	2.61	2.39	6.35	48.45	5.76

3.2	-7.91	3.09	1.29	6.35	35.32	4.81
3.19	-8.03	3.13	1.26	6.35	34.98	4.74
4.36	-0.79	3.01	3.34	6.35	60.28	4.9
4.35	-0.95	3.22	3.28	6.35	59.62	4.34
7.4	1.94	4.48	4.36	6.35	73.43	2.63
7.52	2.73	5.49	4.69	6.35	77.68	1.5
6.77	2.65	5.03	4.66	6.36	77.15	1.98
7.52	-2.39	5.14	2.82	6.36	53.57	1.84
8.98	-1.49	2.12	3.1	6.36	57.27	7.49
3.3	-4.87	2.67	2.06	6.36	44.51	5.65
12.62	-9.58	4.09	0.93	6.36	30.98	3.19
5.99	0.82	4.14	3.93	6.37	67.65	3.13
8.86	-1.89	1.7	2.98	6.37	55.57	8.81
5.12	-7.61	5.49	1.38	6.37	36.08	1.55
11.89	-11.06	2.63	0.67	6.38	27.5	5.73
4.6	-6.84	5.38	1.58	6.38	38.25	1.67
7.15	2.1	4.54	4.45	6.38	74.11	2.48
11	2.11	3.42	4.45	6.38	74.14	3.81
5.17	0.88	4.91	3.97	6.38	67.91	2.09
4.74	-9.72	5.33	0.94	6.38	30.61	1.71
8.65	-9.79	3.26	0.93	6.38	30.42	4.73
6.15	0.2	5.27	3.72	6.38	64.69	1.71
13.02	-4.22	3.13	2.28	6.39	46.65	4.55
6.45	-11.57	4.95	0.6	6.39	26.41	2.17
9.37	-0.94	3.22	3.32	6.39	59.46	4.06
7.33	2.08	4.45	4.45	6.39	73.92	2.59
7.43	2.01	4.41	4.41	6.39	73.61	2.64
7.55	2.06	4.35	4.43	6.39	73.83	2.67
7.54	2.06	4.4	4.44	6.39	73.83	2.58
7.52	1.94	4.85	4.39	6.39	73.24	2.11
7.47	1.98	4.43	4.4	6.39	73.44	2.76
8.41	-9.45	2.91	1	6.39	31.23	5.41
8.51	-9.57	2.92	0.98	6.39	30.95	5.41
7.4	2.62	6.98	4.67	6.39	76.85	0.61
6.06	-5.42	4.39	1.93	6.4	42.57	3.16
3.72	-6.58	4.91	1.63	6.4	38.98	2.23
5.47	-5.9	4.32	1.81	6.4	41.06	3.02
6.85	-1.43	4.32	3.16	6.4	57.31	2.79
15.74	-4.24	1.79	2.28	6.4	46.54	9.26
5.04	-1.88	3.53	3	6.4	55.49	4.13
7.24	2.25	7.11	4.52	6.4	74.81	0.65
3.92	-3.19	5.03	2.57	6.4	50.37	2.08
3.53	-6.92	5.02	1.56	6.4	37.98	2.02
3.27	-5.05	5.18	2.05	6.4	43.8	1.93

4.8	-11.21	4.34	0.63	6.41	27.13	2.91
6.19	-15.9	3.44	-0.09	6.41	18.54	4.59
13	-4.77	3.25	2.14	6.41	44.69	4.42
16.21	-11.84	1.89	0.54	6.41	25.79	8.26
16.52	-12.08	2.33	0.49	6.41	25.32	7.37
4.98	-11.22	4.48	0.63	6.42	27.1	2.97
3.63	-13.44	4.08	0.23	6.42	22.67	3.44
7.08	-1.18	4.7	3.25	6.42	58.32	2.43
4.23	-5.92	3.84	1.81	6.42	40.95	3.61
4.81	-9.3	4.03	1.03	6.42	31.54	3.36
15.48	-11.69	2.34	0.57	6.42	26.1	6.48
5.14	-1.99	3.64	2.97	6.42	54.94	4.13
7.92	-3.82	4.12	2.41	6.42	47.96	2.84
6.28	0.13	5.58	3.71	6.42	64.19	1.52
15.07	-1.96	4.05	2.98	6.43	55.03	2.93
9.77	-1.7	4.6	3.08	6.43	56.07	2.09
10.47	-6.14	2.79	1.79	6.43	40.22	6.1
3.9	-6.41	3.82	1.69	6.43	39.41	3.04
4.27	-3.72	2.82	2.43	6.43	48.31	5.46
3.21	-7.6	3.5	1.41	6.43	35.97	4.19
3.17	-7.64	3.58	1.4	6.43	35.85	3.83
3.62	-7.89	3.31	1.34	6.43	35.17	5.08
7.5	-2.42	5.92	2.84	6.43	53.21	1.2
4.95	-1.78	3.32	3.05	6.43	55.78	4.48
5.24	1.3	5.44	4.15	6.43	69.72	1.63
7.48	2.53	6.17	4.66	6.43	76.16	1.12
10.53	-6.1	2.18	1.8	6.44	40.35	7.77
9.13	-1.19	2.16	3.25	6.44	58.18	7.91
3.33	-5.11	2.77	2.05	6.44	43.47	5.83
3.37	-5.29	2.93	2	6.44	42.87	5.64
7.95	-3.09	5.77	2.64	6.44	50.61	1.39
4.34	-0.61	3.1	3.45	6.44	60.72	4.85
7.01	1.86	5.54	4.39	6.45	72.5	1.61
5.58	0.48	6.96	3.85	6.45	65.67	0.84
5.44	1.17	4.89	4.12	6.45	68.99	2.16
8.57	-3.97	4.41	2.38	6.45	47.34	2.6
6.39	0.26	5.39	3.78	6.45	64.65	1.73
5.02	-1.4	4.78	3.2	6.46	57.2	2.45
5.31	1.25	5.39	4.15	6.46	69.33	1.68
5.36	1.24	5.07	4.15	6.46	69.28	1.97
5.88	1.14	4.85	4.11	6.46	68.77	2.13
5.41	-7.59	5.37	1.44	6.46	35.91	1.75
7.75	6.47	2.65	6.46	6.47	100	6.39
6.3	-0.55	4.52	3.49	6.47	60.82	2.97

11.09	2.34	3.8	4.6	6.47	74.92	3.19
8.84	-9.98	3.24	0.94	6.47	29.78	4.64
7.09	-8.52	2.57	1.24	6.47	33.4	4.96
6.84	0.88	4.42	4.02	6.47	67.48	2.84
9.41	-14.42	2.45	0.12	6.48	20.83	7.12
4.04	-6.38	5.24	1.73	6.48	39.35	2.06
4.57	-7.38	5.22	1.49	6.48	36.43	1.99
12	2.31	3.5	4.58	6.48	74.72	4.04
12.93	-5.25	3.19	2.04	6.48	42.91	4.63
8.85	-2.36	4.17	2.9	6.48	53.24	2.65
7.7	1.94	4.4	4.44	6.48	72.76	2.99
7.51	2.75	6.59	4.77	6.48	77.1	0.86
9.31	-13.49	2.21	0.27	6.49	22.46	6.84
4	-6.34	4.81	1.75	6.49	39.45	2.24
6.16	-0.39	4.22	3.56	6.49	61.5	3.06
14.97	-3.98	1.96	2.41	6.49	47.17	8.16
4.14	-6.29	4.88	1.75	6.49	39.63	2.67
6.52	0.2	7.07	3.78	6.49	64.17	0.67
7.58	1.95	4.37	4.44	6.49	72.76	2.93
7.19	2.59	6.29	4.7	6.49	76.15	1.02
4.02	-3.23	5.03	2.62	6.49	49.89	2.19
5.94	0.44	4.03	3.87	6.5	65.28	3.27
16.01	-4.38	1.92	2.3	6.5	45.75	8.65
4.45	-5.81	4.01	1.88	6.5	41.05	3.49
17.19	-15.55	4.17	0.04	6.5	18.96	3.61
12.92	-1.58	4.48	3.17	6.5	56.32	2.37
5.02	-5.8	2.71	1.9	6.51	41.08	5.99
4.54	-3.61	2.68	2.51	6.51	48.42	6.08
12.84	-5.64	3.77	1.97	6.51	41.56	4.49
18.38	-13.97	4.24	0.29	6.51	21.58	2.8
6.89	2.76	6.45	4.78	6.51	77	0.91
3.72	-7.14	5.63	1.58	6.51	37.05	1.6
7.33	-0.93	5.02	3.39	6.52	58.97	2.2
3.43	-5.53	2.94	1.98	6.52	41.87	5.76
4.87	0.12	4.63	3.76	6.52	63.67	2.4
3.66	-0.79	3.55	3.42	6.52	59.6	4.54
7.57	2.93	6.47	4.86	6.52	77.81	0.89
11.79	-14.98	3.93	0.11	6.53	19.83	3.59
7.66	4.1	3.91	5.38	6.53	84.48	3.6
6.71	2.74	5.12	4.8	6.53	76.78	2.14
17.1	-1.67	3.36	3.14	6.53	55.86	4.07
8.06	-3.06	5.52	2.7	6.53	50.39	1.63
8.16	-3.13	5.48	2.68	6.53	50.13	1.68
8.28	-3.16	5.58	2.67	6.53	50.01	1.65

-0.67	-19.06	-2.68	-5.83	6.54	25.69	0.85
7.93	6.54	2.85	6.54	6.54	100	6.61
7.76	3.79	4.82	5.24	6.54	82.57	2.42
7.21	1.88	5.57	4.44	6.54	72.16	1.63
13.5	-5.77	2.92	1.95	6.54	41.09	5.42
10.08	-1.07	2.83	3.36	6.54	58.34	5.48
10.86	2.18	4.19	4.57	6.54	73.76	3.25
10.91	2.17	4.14	4.55	6.54	73.7	3.41
10.96	2.09	4.17	4.53	6.54	73.23	3
6.47	0.43	6.06	3.9	6.54	65.01	1.19
5.81	0.65	6.78	3.97	6.54	66.08	0.84
12.4	-11.64	2.67	0.66	6.55	25.96	6.47
4.71	-6.88	5.74	1.66	6.55	37.69	1.54
13.28	-8.18	3.94	1.34	6.55	34.11	2.97
10.86	-1.43	4.37	3.24	6.56	56.7	2.68
16.87	-2.66	3.28	2.85	6.56	51.8	5.01
12.65	-4.4	3.57	2.32	6.56	45.49	4.25
6.64	-11.71	4.94	0.68	6.56	25.81	2.23
10.5	-0.93	6.35	3.43	6.56	58.8	1.04
8.3	-9.35	3.42	1.12	6.56	31.09	4.38
7.13	2.46	6.38	4.7	6.56	75.11	1.03
4.2	-3.25	5.17	2.65	6.56	49.6	2.35
9.24	-1.02	2.23	3.37	6.57	58.4	8.24
17.94	-13.63	4.3	0.38	6.57	22.09	2.5
11.38	0.13	5.57	3.8	6.57	63.48	1.36
7.92	2.02	4.4	4.51	6.57	72.73	3.03
3.76	-7.23	5.74	1.59	6.57	36.66	1.63
3.84	-6.48	4.69	1.76	6.58	38.79	2.6
3.94	-6.46	4.94	1.76	6.58	38.85	2.25
6.05	1.98	4.2	4.5	6.58	72.45	3.05
3.47	-5.68	2.8	1.97	6.58	41.24	6.15
8.56	1.94	4.55	4.49	6.58	72.25	2.99
5.62	-10.03	4.35	1	6.58	29.45	3.14
3.89	-6.69	4.45	1.71	6.59	38.16	2.7
18.74	-13.74	4.47	0.37	6.59	21.87	3.17
5.28	-2.01	3.93	3.07	6.59	54.25	3.99
6.66	0.4	6.78	3.91	6.59	64.65	0.84
10.96	2.04	4.37	4.53	6.59	72.73	2.81
5.63	-8.35	6.35	1.35	6.6	33.54	1.61
11.08	-5.84	2.73	1.96	6.6	40.67	7.05
16.65	-11.92	2.43	0.64	6.6	25.31	5.96
3.63	-0.77	3.42	3.47	6.6	59.35	4.97
3.63	-0.7	3.4	3.49	6.6	59.67	4.98
16.62	-15.35	4.28	0.12	6.6	19.15	2.86

14.52	-1.43	4.26	3.27	6.6	56.57	3.43
3.29	-7.46	3.34	1.55	6.61	35.93	4.63
8.45	-3.19	5.63	2.71	6.62	49.58	1.67
11.03	2.24	4.37	4.63	6.62	73.61	3.07
6.03	0.65	7.1	4.01	6.62	65.73	0.77
7.47	-8.51	2.8	1.33	6.62	33.08	5.86
5.77	-7.58	5.35	1.53	6.62	35.56	1.98
3.61	-6.93	4.33	1.68	6.63	37.34	2.91
7.4	1.93	5.56	4.51	6.63	71.93	1.73
13.09	1.88	4.14	4.5	6.63	71.73	3.25
10.84	2.22	4.19	4.63	6.63	73.5	3.3
5.59	-6.07	5.74	1.91	6.64	39.86	1.55
10.12	-1	6.71	3.45	6.64	58.22	0.84
11.12	-0.96	3.55	3.45	6.64	58.39	4.56
8.16	-9.83	3.74	1.07	6.64	29.8	4.1
7	2.83	7.08	4.88	6.64	76.67	0.69
7.6	-0.63	5.07	3.57	6.65	59.77	2.31
16.14	-2.39	4.41	2.99	6.65	52.52	2.98
6.76	0.36	6.7	3.92	6.65	64.24	0.9
3.87	-6.54	5.11	1.79	6.66	38.41	2.2
6.1	2.29	4.9	4.67	6.66	73.7	2.35
7.66	-3.06	6.07	2.77	6.66	49.91	1.31
9.8	-0.97	7.13	3.46	6.66	58.28	0.61
6.92	0.26	6.93	3.9	6.66	63.67	0.81
8.28	1.64	4.41	4.41	6.66	70.39	3.1
8.07	-0.24	3.2	3.71	6.67	61.4	4.92
8.24	4.28	3.97	5.53	6.67	84.74	3.66
18.74	-14.13	4.66	0.36	6.67	21.06	2.89
4.14	-3.23	4.77	2.71	6.67	49.3	2.52
7.75	3.56	6.06	5.21	6.67	80.56	1.32
3.65	-4.94	5.23	2.23	6.67	43.36	2.09
3.73	-4.94	5.18	2.23	6.67	43.36	2.16
13.19	-1.55	4.95	3.27	6.67	55.77	2.17
10.88	-5.87	3.37	2	6.68	40.35	5.42
4.31	-5.95	3.86	1.95	6.68	40.11	3.97
3.55	-6.54	3.83	1.81	6.68	38.35	4.53
5.12	-9.46	3.93	1.15	6.68	30.58	4.09
3.52	-4.98	5.51	2.22	6.68	43.21	1.79
14.96	-2.05	4.28	3.1	6.69	53.72	3.13
5.7	-5.91	5.7	1.98	6.69	40.2	1.68
3.73	-0.7	3.24	3.54	6.69	59.29	5.1
3.68	-0.61	3.37	3.58	6.69	59.69	5.2
10.88	-0.82	3.58	3.53	6.69	58.77	4.46
10.52	-0.71	3.31	3.57	6.69	59.26	4.82

6.63	2.8	5.51	4.9	6.7	76.18	1.91
4.39	-0.46	3.05	3.64	6.7	60.29	5.59
3.68	-6.75	4.43	1.76	6.71	37.68	2.89
8.62	-3.21	5.36	2.76	6.71	49.19	2.07
9.38	-0.99	2.47	3.46	6.71	57.97	8.11
5.42	-1.3	5.97	3.37	6.72	56.62	1.55
6.14	2.47	5.8	4.78	6.72	74.31	1.54
4.37	-0.43	3.37	3.67	6.72	60.35	5.28
9.87	2.08	4.4	4.62	6.72	72.29	3.31
10.57	2.11	4.23	4.63	6.72	72.43	3.32
6.06	-7.61	5.26	1.58	6.72	35.24	2.27
5.78	-5.81	5.82	2.02	6.73	40.42	1.71
7.86	-0.3	5.44	3.73	6.73	60.87	1.92
7.66	1.89	5.41	4.55	6.73	71.31	1.95
13.75	-4.25	3.71	2.46	6.73	45.49	4.62
13.63	-4.37	3.71	2.42	6.73	45.04	4.43
13.04	-4.43	3.81	2.41	6.73	44.86	4.43
18.31	-9.7	3.21	1.15	6.73	29.9	5.81
11.44	-0.39	3.55	3.7	6.73	60.47	4.5
8.99	2.11	4.74	4.64	6.73	72.37	2.75
9.32	1.91	4.68	4.56	6.73	71.36	2.84
9.46	2.1	4.62	4.63	6.73	72.37	2.92
5.94	-10.33	4.53	1.02	6.73	28.47	3.22
7.99	1.97	5.53	4.59	6.74	71.62	1.94
12.62	-3.81	3.93	2.59	6.74	46.98	3.89
18.67	-9.71	3.09	1.16	6.74	29.87	6.03
4.55	-5.25	4.46	2.16	6.74	42.14	3.45
4.9	-11.98	5.79	0.72	6.74	24.95	1.68
4.88	-6.85	5.55	1.78	6.74	37.29	1.85
12.06	-6.04	2.93	1.99	6.74	39.69	5.09
7.7	-10.51	3.62	0.99	6.74	28.04	4.45
7.83	-2.79	5.67	2.9	6.74	50.66	1.66
7.59	-2.14	5.57	3.1	6.74	53.17	1.56
7.62	3.28	7.46	5.12	6.74	78.62	0.61
14.21	-1.36	5.43	3.37	6.74	56.28	2.15
4.23	-6.2	5.12	1.93	6.75	39.18	2.31
16.64	-4.25	2.23	2.48	6.75	45.4	8.45
7.82	-8.66	2.86	1.37	6.75	32.41	6.21
4.84	-7.31	5.05	1.67	6.76	35.95	2.55
3.74	-0.55	3.58	3.64	6.76	59.65	5.03
5.37	-2.09	4.44	3.13	6.76	53.29	3.59
4.29	-6.3	5.28	1.92	6.77	38.82	2.25
8.19	-0.03	5.45	3.85	6.77	61.93	2.08
5.21	-9.63	4.2	1.17	6.77	30	3.94

7.6	3.19	7.08	5.11	6.77	77.96	0.74
3.72	-7.94	3.73	1.52	6.78	34.2	4.95
7.12	0.16	7.23	3.92	6.78	62.73	0.77
14.55	-1.23	4.54	3.44	6.78	56.69	2.96
6.4	-0.49	5.23	3.69	6.79	59.79	2.26
14.02	1.87	4.45	4.57	6.79	70.9	3.27
9.53	-14.12	2.71	0.35	6.8	20.89	6.8
8.07	6.8	3.61	6.81	6.8	100	5.1
4.48	-2.98	7.33	2.87	6.8	49.73	0.76
6.23	0.62	6.94	4.1	6.8	64.78	0.93
6.96	-10.41	4.23	1.05	6.8	28.14	3.72
6.2	2.76	6.2	4.94	6.8	75.47	1.33
4.35	-13.35	4.69	0.48	6.81	22.23	3.15
15.52	-2.66	4.44	2.99	6.81	50.91	3.27
8.78	-3.29	5.47	2.78	6.81	48.58	2.01
10.41	2.1	4.63	4.68	6.81	71.97	2.82
12.05	-15.48	3.76	0.21	6.82	18.67	4.69
11.9	-15.33	3.76	0.23	6.82	18.89	4.07
4.86	-3.57	3.09	2.69	6.82	47.55	5.79
11.36	-0.27	6.53	3.78	6.82	60.66	1.1
11.58	-0.38	3.9	3.75	6.82	60.14	4.4
11.24	-0.57	4.03	3.68	6.82	59.34	3.78
13.3	-11.48	2.99	0.85	6.83	25.8	6.78
6.52	-0.8	5.29	3.6	6.83	58.31	2.28
8.27	1.93	5.22	4.62	6.83	70.97	2.52
12.42	-4.45	3.84	2.47	6.83	44.49	3.81
12.81	-11.5	2.23	0.85	6.84	25.74	7.69
5.18	-12.09	5.32	0.76	6.84	24.55	2.33
5	-6.82	5.26	1.85	6.84	37.12	2.29
2.63	-14.32	2.43	-1.58	6.85	25.68	1.42
7.27	-16.68	3.96	0.07	6.85	16.86	4.51
6.09	-0.03	4.46	3.88	6.85	61.58	3.06
3.85	-0.73	3.67	3.63	6.85	58.52	4.8
5.23	-11.32	4.46	0.87	6.86	26.08	3.53
3.61	-6.1	3.26	2.02	6.86	39.16	5.93
12.36	-1.25	6.77	3.48	6.86	56.27	1.28
6.56	2.67	6.74	4.93	6.86	74.68	1.1
8.26	-2.59	5.07	3.02	6.86	51	2.55
4.54	-5.72	4	2.11	6.87	40.32	4.04
3.67	-6.34	3.52	1.96	6.87	38.44	5.63
3.49	-7.12	3.82	1.78	6.87	36.21	4.64
3.76	-0.5	3.69	3.71	6.87	59.41	4.93
8.09	-2.72	5.22	2.99	6.87	50.48	2.27
5.08	-7.06	5.22	1.81	6.88	36.36	2.53

6.51	0.56	7.31	4.12	6.88	64.12	0.83
14.6	-1.25	4.24	3.49	6.88	56.22	3.46
5.01	-7.29	4.64	1.75	6.89	35.69	3.23
8.18	6.89	3.56	6.9	6.89	100	5.37
3.79	-0.49	4.02	3.72	6.89	59.41	4.5
17.63	-13.31	3.89	0.6	6.89	22.18	4.32
5.76	-1.21	6.04	3.5	6.9	56.31	1.61
8.09	6.9	3.37	6.9	6.9	100	5.78
9.56	-0.94	2.44	3.57	6.9	57.43	9.04
4.43	-2.93	6.35	2.94	6.9	49.6	1.27
7.17	0.51	6.96	4.11	6.9	63.78	0.91
10.24	2.22	4.67	4.77	6.9	72.13	2.96
8.67	4.51	5	5.77	6.91	84.7	2.52
6.79	-1.13	5.46	3.53	6.91	56.6	2.15
8.58	0.03	5.65	3.94	6.91	61.59	2.08
10.2	0.46	3.2	4.1	6.91	63.56	4.78
8.6	0.82	5.25	4.23	6.91	65.2	1.9
7.25	0.37	6.52	4.07	6.91	63.13	1.2
6.73	-10.99	4.3	1	6.91	26.68	3.64
6.95	-5.89	5.24	2.1	6.92	39.66	1.98
6.56	-0.61	5.2	3.72	6.92	58.73	2.48
6.62	-0.89	5.33	3.62	6.92	57.55	2.28
4.82	-7.09	4.45	1.8	6.92	36.18	3.19
4.32	-3.06	5.85	2.91	6.92	49.05	1.82
4.87	-5.45	5.84	2.22	6.93	40.96	1.91
7.46	0.09	5.07	3.98	6.93	61.77	2.57
7.63	0.03	4.97	3.96	6.93	61.51	2.87
4.76	-5.15	4.73	2.3	6.94	41.9	3.38
3.45	-7.21	3.44	1.8	6.94	35.79	5.15
11.48	5.26	3.6	6.11	6.94	89.08	5.41
4.39	-0.37	3.47	3.81	6.94	59.71	5.37
5.72	0.52	7.73	4.14	6.94	63.7	0.64
11.33	-5.82	3.76	2.16	6.95	39.78	5.34
7.45	0.2	7.48	4.03	6.95	62.14	0.76
4.04	-7.18	6.07	1.82	6.95	35.85	1.77
4.03	-0.69	3.61	3.7	6.96	58.25	5.33
6.34	2.79	6.85	5.04	6.96	74.79	0.96
9.69	-4.06	5.57	2.65	6.97	45.38	2.11
9.91	-4.03	5.52	2.66	6.97	45.5	2.08
10.47	-4.05	4.9	2.66	6.97	45.41	2.78
8.3	6.98	3.95	6.98	6.98	100	4.69
13.18	-2.8	4.79	3.03	6.98	49.82	3.38
11.87	-1.04	5.98	3.61	6.98	56.7	1.28
9.87	-4.67	4.44	2.48	6.98	43.32	3.97

6.96	-1.26	5.66	3.54	6.99	55.75	2.03
13.99	1.7	4.13	4.61	6.99	69.03	3.99
5.11	-6.93	4.65	1.88	6.99	36.44	3.26
11.28	-0.32	6.29	3.86	6.99	59.69	1.32
6.5	-10.61	4.96	1.12	6.99	27.35	2.81
6.11	-1.18	5.79	3.57	7	56.07	1.92
15.36	-3.04	4.27	2.97	7	48.85	4.2
9.25	-3.34	5.19	2.87	7	47.79	2.67
12.81	-4.07	4.55	2.67	7	45.26	3.29
9.95	-10.28	3.27	1.2	7	28.06	5.16
8.55	-2.5	5.31	3.13	7	50.85	2.33
7.82	-16.36	4.53	0.21	7.01	17.12	3.76
13.65	-11.35	3.01	0.98	7.01	25.76	6.94
13.98	1.57	4.7	4.57	7.01	68.36	3.35
13.75	-4.32	4.14	2.59	7.01	44.37	4.6
8.2	-8.27	3.05	1.61	7.01	32.81	6.43
8.82	-4.69	4.08	2.49	7.01	43.14	4.51
13.89	-1.17	5.71	3.59	7.01	56.04	1.78
6.35	-5.19	5.12	2.34	7.02	41.52	3.11
9.17	-10.36	5.15	1.14	7.02	27.85	2.31
4.8	-13.29	4.87	0.61	7.02	22.03	3.33
17.33	-3.89	2.49	2.74	7.02	45.8	8.34
5.46	-12.01	4.98	0.88	7.02	24.41	3.06
4.37	-3.02	6.14	2.97	7.02	48.86	1.47
19.11	-13	5.64	0.74	7.02	22.53	2.03
10.82	-6.28	3.5	2.08	7.02	38.21	5.91
6.07	0.44	4.29	4.15	7.03	62.93	4.26
9.72	-1.98	4.79	3.33	7.03	52.73	2.82
13.96	-1.15	5.4	3.6	7.03	56.07	2.38
5.5	-11.33	4.67	0.97	7.04	25.76	3.73
5.26	-6.8	4.58	1.95	7.04	36.69	3.95
8.61	-0.84	5.85	3.71	7.04	57.29	1.88
6.06	0.28	4.41	4.09	7.04	62.16	3.52
10.76	-1.66	5.57	3.43	7.04	53.96	1.82
11.53	-6.61	3.54	2.01	7.04	37.24	6.71
5.23	-3.5	3.34	2.84	7.04	47.08	6.01
3.86	-0.47	4.08	3.82	7.04	58.84	4.76
5.59	-2.25	4.52	3.24	7.04	51.68	3.79
8.86	-2.26	5.35	3.23	7.04	51.62	2.44
8.88	-2.24	5.36	3.23	7.04	51.71	2.43
4.26	-6.2	4.14	2.09	7.05	38.37	3.84
3.75	-6.4	3.72	2.05	7.05	37.79	5.49
4.37	-0.38	3.72	3.86	7.05	59.19	5.32
5.71	-2.38	4.48	3.2	7.05	51.13	3.93

14.83	-1.81	4.83	3.39	7.05	53.32	3.5
9.74	-0.61	2.56	3.79	7.06	58.18	9.16
4.64	-5.56	4.59	2.26	7.06	40.28	3.63
9.75	-4.33	5.39	2.62	7.06	44.2	2.33
4.77	-5.67	5.52	2.25	7.07	39.92	2.25
8.33	7.07	3.7	7.07	7.07	100	5.6
7.09	-1.48	6.23	3.5	7.07	54.57	1.6
6.57	-1.17	5.81	3.62	7.08	55.79	1.99
8.54	1.92	5.75	4.75	7.08	69.73	2.23
9.51	-3.34	5.45	2.92	7.08	47.51	2.51
13.48	-3.55	4.59	2.86	7.08	46.76	3.48
4.7	-2.99	6.59	3.01	7.08	48.78	1.37
5.19	-7.06	5.04	1.93	7.08	35.87	2.88
11.22	-0.2	6.77	3.96	7.08	59.85	1.15
11.72	-0.41	4.38	3.88	7.08	58.96	3.53
4.76	-2.99	6.3	3.02	7.08	48.75	1.61
4.81	-2.99	5.89	3.02	7.08	48.75	2.11
11.85	-15.92	3.98	0.31	7.09	17.66	4.98
7.76	-3.84	5.61	2.78	7.09	45.75	1.96
8.99	-0.18	5.41	3.97	7.09	59.94	2.77
9.17	-4.52	4.28	2.58	7.09	43.48	4.11
13.9	-4.15	3.79	2.7	7.1	44.68	4.86
10.05	0.42	2.59	4.17	7.1	62.52	9.39
5.47	-6.25	4.94	2.11	7.1	38.1	2.99
4.21	-0.65	3.87	3.79	7.1	57.85	5.09
8.08	0.24	4.83	4.13	7.1	61.69	3.44
11.57	-1.26	5.37	3.6	7.11	55.33	2.15
5.96	-5.7	5.63	2.27	7.11	39.71	2.22
6.24	-5.32	5.31	2.37	7.11	40.87	2.87
13.27	-3	4.76	3.04	7.11	48.63	3.14
5.34	-7.18	5.07	1.92	7.11	35.44	3.03
5.47	-7.48	5.22	1.85	7.11	34.64	2.78
9.81	-7	3.95	1.95	7.11	35.96	5.01
9.96	-7.51	3.81	1.83	7.11	34.58	5.31
10.17	-7.11	3.72	1.93	7.11	35.66	5.59
10.57	-7.37	3.81	1.86	7.11	34.95	5.28
10.86	-6.71	3.45	2.02	7.11	36.76	6.26
10.89	-6.75	3.62	2.01	7.11	36.64	5.94
19.65	-9.28	3.03	1.46	7.12	30.1	7.53
4.88	-4.82	4.66	2.49	7.12	42.4	3.91
11.39	5.56	3.65	6.35	7.12	89.84	6.24
10.46	-7.58	3.88	1.81	7.12	34.37	5.17
14.89	-1.45	5.32	3.55	7.12	54.47	2.93
12.59	-1.12	6.76	3.67	7.13	55.77	1.19

3.92	-0.37	4.18	3.9	7.13	58.94	4.6
5.85	-2.52	4.46	3.2	7.13	50.35	4.1
8.85	-2.25	5.42	3.28	7.13	51.34	2.31
3.81	-6.32	3.79	2.12	7.14	37.8	5.54
3.64	-6.73	3.84	2.03	7.14	36.64	4.73
6.97	0.63	7.62	4.29	7.14	63.32	0.75
6.78	0.67	7.68	4.3	7.14	63.49	0.7
14.79	-1.22	4.91	3.65	7.14	55.34	2.84
9.54	-14.13	3.07	0.56	7.15	20.38	6.76
8.45	7.15	4.13	7.15	7.15	100	5.06
13	-3.13	5.27	3.03	7.15	48.05	2.64
9.87	-0.01	2.74	4.04	7.15	60.38	8.75
18.14	-12.94	4.32	0.81	7.15	22.45	4.26
11.84	-0.31	4.9	3.96	7.15	59.09	3.2
7.17	0.66	7.38	4.31	7.15	63.44	0.92
7.09	-1.32	5.65	3.61	7.16	54.85	2.25
7.14	-1.6	6.21	3.51	7.16	53.75	1.82
15.22	-6.53	3.48	2.11	7.16	37.13	6.56
4.36	-0.53	4.07	3.87	7.16	58.12	4.75
18.54	-11.76	4.02	1.01	7.16	24.67	4.77
9.16	-6.64	5.04	2.06	7.16	36.83	3.06
9.77	-3.56	5.51	2.91	7.17	46.45	2.58
14.02	-4.85	4.03	2.54	7.17	42.17	4.29
4.31	-0.57	4.32	3.85	7.17	57.92	4.21
8.51	-6.98	4.93	1.99	7.17	35.85	3.35
4.98	-5.37	6.01	2.39	7.18	40.51	2.06
9.17	4.78	5.47	6.01	7.18	84.74	2.28
12.92	-2.74	5.05	3.16	7.18	49.35	3.03
10.95	0.94	3.32	4.42	7.18	64.52	6.85
11.07	-0.52	6.79	3.89	7.18	58.09	1.28
7.89	-7.34	4.95	1.91	7.18	34.87	3.32
9.46	-4.89	4.07	2.53	7.18	42.02	4.61
4.02	-0.36	4.21	3.94	7.19	58.73	4.9
8.89	-8.05	3.41	1.76	7.19	32.99	6.81
8.97	-6.93	4.86	2.01	7.19	35.94	3.36
9.74	-0.09	5.65	4.06	7.19	59.9	2.38
8.9	-0.42	6.18	3.93	7.2	58.44	1.75
5.71	-12.17	5.26	0.95	7.2	23.8	2.93
5.41	-7.26	5.15	1.95	7.2	35.02	2.95
5.6	-7.51	5.24	1.89	7.2	34.35	2.79
5.74	-7.51	5.03	1.89	7.2	34.35	3.51
4.72	-5.81	5.2	2.29	7.21	39.12	2.72
5.98	-2.65	4.62	3.21	7.21	49.57	3.97
6.28	-2.92	5.07	3.11	7.21	48.59	3.25

11.79	-5.85	3.76	2.31	7.22	38.97	6.14
7.97	-6.44	4.15	2.14	7.22	37.26	5.75
5.64	-3.44	3.42	2.96	7.22	46.73	6.43
12.88	-1.21	7.11	3.69	7.22	55.06	0.94
5.93	0.56	7.27	4.3	7.22	62.63	1.03
14.87	-1.61	5.07	3.55	7.22	53.47	3.33
14.8	-1.85	4.82	3.46	7.23	52.5	2.92
8.39	-0.83	5.79	3.81	7.23	56.6	2.12
12.82	-2.97	5.59	3.12	7.23	48.33	2.5
10.23	0.55	2.66	4.29	7.23	62.57	9.72
9.29	-0.13	7.44	4.06	7.24	59.51	0.84
7.9	0.41	7.18	4.26	7.24	61.92	1.08
5.85	0.64	6.92	4.34	7.24	62.94	1.17
14.42	-1.53	4.88	3.59	7.24	53.73	3.58
7.62	-1.34	5.74	3.65	7.25	54.46	2.21
7.41	0.75	6.93	4.39	7.25	63.4	1.25
8.75	-6.93	4.9	2.04	7.25	35.8	3.35
9.22	-16.99	4.23	0.28	7.26	15.96	4.89
7.91	-3.66	7.44	2.93	7.26	45.84	1.69
13.29	1.59	4.79	4.71	7.26	67.29	3.74
10.72	-4.14	5.5	2.78	7.26	44.23	2.58
11.02	-3.9	5.19	2.85	7.26	45	3.04
4.85	-3.02	5.98	3.11	7.26	48.07	2.16
8.21	-7.05	5.1	2.02	7.26	35.43	3.04
14.84	-2.84	4.49	3.19	7.27	48.66	4
10.07	-3.59	5.19	2.95	7.27	46.05	3.08
9.26	0.06	6.19	4.15	7.27	60.23	1.78
9.52	0.04	5.57	4.15	7.27	60.09	2.4
9.44	-14.07	3.68	0.64	7.28	20.3	5.99
7.45	-11.63	6	1.12	7.28	24.72	2.02
8.52	-8.24	3.35	1.77	7.28	32.29	6.36
13.58	-1.14	4.68	3.74	7.28	55.11	4.21
4.47	-6.06	5.47	2.27	7.29	38.16	2.73
4.54	-5.91	5.57	2.31	7.29	38.61	2.72
5.84	-11.34	4.91	1.12	7.3	25.28	3.92
4.6	-5.88	5.31	2.33	7.3	38.68	2.74
4.97	-5.2	4.61	2.49	7.3	40.71	4.47
5.44	-9.81	4.82	1.44	7.3	28.53	4.09
6.12	-2.76	4.86	3.22	7.3	48.84	3.47
9.77	-2.29	5.6	3.36	7.3	50.58	2.62
9.83	-10.94	4.7	1.21	7.31	26.07	3.67
5.48	-7.19	4.61	2.01	7.31	34.95	4.3
6.35	-6.38	5.06	2.2	7.31	37.18	3.08
3.81	-6.67	4.16	2.14	7.31	36.39	5.66

4.1	-0.35	4.28	4.01	7.31	58.29	5.15
8.7	-14.02	3.36	0.67	7.32	20.33	7.62
12.48	-2.96	5.71	3.17	7.32	48.08	2.49
6.09	-6.68	4.76	2.16	7.32	36.3	4.02
4.96	-7.38	6.75	1.99	7.33	34.39	1.56
10.38	0.54	2.58	4.33	7.33	62.08	10.96
14.29	-5.85	4.28	2.38	7.33	38.68	3.96
9.82	-0.26	7.22	4.06	7.33	58.61	1.02
10.3	-0.25	7.76	4.07	7.33	58.64	0.86
11.23	-0.14	6.02	4.11	7.33	59.13	2.1
14.22	-1.18	4.76	3.75	7.33	54.77	3.92
14.4	-1.18	4.93	3.76	7.33	54.79	3.54
13.85	1.92	4.88	4.88	7.34	68.49	3.44
10.42	-3.65	5.83	2.97	7.34	45.61	2.24
11.38	-3.86	5.57	2.91	7.34	44.9	2.66
8.66	-0.01	6.96	4.16	7.34	59.65	1.19
10.51	-0.03	5.98	4.16	7.34	59.53	2.16
10.78	-0.28	5.85	4.07	7.34	58.47	2.24
13.44	-1.03	4.59	3.81	7.34	55.35	4.05
6.57	-5.01	5.91	2.57	7.35	41.17	3.47
8.81	-16.57	4.65	0.39	7.35	16.43	4.02
9	1.9	5.78	4.87	7.35	68.35	2.5
10.77	-11.63	3.53	1.15	7.35	24.6	6.76
6.07	-12.25	5.35	1.03	7.35	23.41	3.06
11.49	0.97	4.03	4.52	7.35	63.93	5.63
7.73	-11.66	6.42	1.16	7.35	24.54	1.74
7.68	0.74	6.22	4.44	7.35	62.91	1.91
10.68	-5.42	4.42	2.48	7.35	39.9	4.66
8.52	0.4	5.56	4.32	7.35	61.37	2.73
5.74	-13.44	5	0.79	7.36	21.24	3.92
8.98	-0.01	3.79	4.16	7.36	59.54	5.39
8.6	7.36	4.3	7.36	7.36	100	5.12
19.39	-13.21	6.02	0.91	7.36	21.65	2.22
10.87	-5.34	4.39	2.51	7.36	40.11	4.67
13.12	2.19	4.98	5	7.37	69.71	3.27
5.13	-5.29	5.58	2.51	7.37	40.27	3.62
13.19	0.23	3.52	4.26	7.37	60.57	7.09
19.25	-12.83	5.75	0.98	7.37	22.31	2.62
9.62	-8.03	3.41	1.87	7.37	32.61	7.22
9.39	-8	3.23	1.87	7.37	32.7	7.63
8.91	-14	3.84	0.71	7.38	20.27	6.82
11.36	-16.78	4.75	0.37	7.38	16.12	4.14
20.42	-9.29	3.58	1.61	7.38	29.57	6.92
19.96	-9.63	3.23	1.54	7.38	28.76	7.26

12.81	0.62	3.79	4.4	7.38	62.2	6.42
6.51	-7.4	5.13	2.02	7.38	34.22	3.57
14.15	-1.33	7.47	3.73	7.38	53.99	1.13
12.34	-6.08	3.96	2.35	7.39	37.84	6.4
6.18	-7.29	4.77	2.04	7.39	34.49	4.11
6.28	-7.26	4.88	2.06	7.39	34.59	3.85
9.48	-2.19	5.74	3.44	7.39	50.66	2.34
5.7	-6.9	4.36	2.13	7.4	35.51	4.71
5.92	-6.86	4.86	2.14	7.4	35.63	4.36
5.7	-3.39	3.69	3.07	7.4	46.34	6.51
5.72	-3.41	3.59	3.06	7.4	46.25	6.56
4.37	-0.72	4.47	3.93	7.4	56.4	4.7
13.16	-0.89	5.65	3.89	7.4	55.68	2.69
12.64	-3.36	5.86	3.1	7.41	46.4	2.21
4.36	-0.61	4.26	3.97	7.41	56.81	4.75
8.02	-0.08	7.39	4.18	7.41	59.03	0.99
13.39	-1.15	5.37	3.81	7.41	54.61	2.89
15.06	-1.6	5.17	3.66	7.41	52.86	2.84
7.17	-1.66	6.58	3.63	7.42	52.57	1.77
18.76	-10.54	4.04	1.39	7.42	26.7	5.54
7.96	0.76	6.44	4.48	7.42	62.67	1.79
8.16	0.69	6.57	4.46	7.42	62.38	1.71
14.96	-1.39	6.46	3.73	7.42	53.59	2.07
11.62	-16.39	4.67	0.46	7.43	16.59	4.46
14.25	-0.07	4.56	4.17	7.43	59	5.3
8.17	0.1	7.7	4.25	7.43	59.75	0.82
11.53	0.1	6	4.25	7.43	59.72	1.95
10.11	-16.61	4.37	0.43	7.44	16.28	5.11
10.79	-15.79	4.14	0.54	7.44	17.43	5.44
11.66	-15.92	4.44	0.52	7.44	17.24	4.36
3.94	-8.16	4.77	1.85	7.44	32.15	3.89
11.88	6.14	4.97	6.79	7.44	91.47	4.16
6.37	-7.47	5.1	2.03	7.44	33.91	3.53
10.71	-0.26	6.74	4.13	7.44	58.14	1.48
7.74	-3.86	6.23	2.98	7.45	44.56	1.64
13.78	1.93	4.78	4.94	7.45	68.04	3.74
11.72	-3.55	5.58	3.06	7.45	45.6	2.8
5.73	-9.9	5.74	1.52	7.46	28.02	2.63
11.71	6.25	4.5	6.85	7.46	92.07	4.75
7.76	-5.8	5.5	2.43	7.47	38.46	2.45
6.59	-4.92	4.67	2.68	7.47	41.1	4.9
6.48	-2.97	5.35	3.25	7.47	47.57	3.32
8.42	-13.95	3.68	0.78	7.48	20.22	6.78
5.91	-3.36	3.73	3.12	7.48	46.17	6.52

9.69	-2.05	5.27	3.54	7.48	50.85	3.19
8.7	7.49	5.06	7.5	7.49	100	3.78
14.91	-3.04	4.61	3.25	7.49	47.28	4.23
4.16	-0.26	4.24	4.14	7.49	57.95	5.5
4.19	-0.19	4.34	4.16	7.49	58.24	5.28
4.41	-0.73	4.49	3.97	7.49	56.01	4.92
10.29	-10	3.64	-0.35	7.5	34.6	1.02
12.3	-3.4	5.84	3.14	7.5	45.97	2.38
10.56	0.38	2.72	4.37	7.5	60.63	11.15
5.54	-2.93	7.01	3.27	7.5	47.6	1.54
13.79	-0.93	5.59	3.93	7.5	55.15	2.82
12.01	-3.5	5.66	3.11	7.51	45.61	2.74
12.12	-0.77	7.03	4	7.51	55.76	0.87
4.47	-0.58	4.65	4.03	7.51	56.57	4.03
8.65	0.57	6.59	4.46	7.51	61.46	1.84
9.66	-2.03	5.31	3.55	7.51	50.82	3.26
14.79	-1.29	6.67	3.82	7.51	53.66	1.61
14.18	-1.04	5.18	3.89	7.51	54.68	3.17
9.49	1.69	5.95	4.89	7.52	66.58	2.5
11.12	-16.51	4.58	0.5	7.53	16.32	4.56
5.11	-5.34	6.49	2.59	7.53	39.65	1.93
8.72	7.52	5.11	7.53	7.53	99.89	3.83
9.46	-1.28	5.93	3.83	7.53	53.67	2.81
14.96	-2.97	5.05	3.29	7.53	47.37	3.53
10.66	-0.08	7.14	4.23	7.53	58.54	1.19
4.37	-7.24	6.79	2.14	7.53	34.3	1.65
8.15	-13.56	4.09	0.87	7.54	20.79	6.28
10.51	-16.34	4.47	0.53	7.54	16.53	5.01
5.05	-4.97	4.8	2.69	7.54	40.74	4.56
6.2	-11.52	5.52	1.24	7.55	24.49	3.26
13.95	-11.34	3.35	1.29	7.55	24.84	7.15
13.47	-0.94	8.05	3.96	7.55	54.92	0.89
10.78	0.24	2.95	4.34	7.56	59.81	11.05
5.56	-9.88	5.42	1.58	7.56	27.87	3.21
18.13	-3.71	2.55	3.09	7.57	44.69	9.69
5.94	-7.67	5.2	2.06	7.57	33.1	3.96
4.41	-0.67	4.61	4.04	7.57	55.94	4.77
11.49	0.11	5.76	4.33	7.58	59.15	3.09
15.04	-1.42	5.44	3.81	7.58	52.95	2.54
9.91	4.94	5.83	6.3	7.59	83.31	2.36
14.81	-1.14	6.95	3.91	7.59	53.97	1.49
8.01	-11.72	6.8	1.28	7.59	24.02	1.65
11.97	-0.07	6.24	4.28	7.59	58.35	2.24
5.24	-2.93	7.3	3.32	7.6	47.29	1.21

14.89	-6.41	4.89	2.39	7.6	36.36	3.97
4.26	-0.2	4.43	4.21	7.6	57.77	5.53
9.98	1.6	5.83	4.9	7.61	65.75	2.81
9.16	0.29	6.65	4.41	7.61	59.82	1.89
9.17	-14.71	4.14	0.75	7.62	18.82	6.77
14.27	-11.53	3.86	1.3	7.62	24.34	6.91
11.95	6.39	5.04	7	7.62	91.89	3.9
11.96	6.26	5	6.93	7.62	91.09	4.4
6.79	-7.24	6.1	2.19	7.62	34.09	2.73
19.83	-12.72	6.72	1.14	7.62	22.14	1.89
6.17	-13.48	4.95	0.94	7.63	20.8	4.55
5.93	-9.91	6.44	1.61	7.63	27.66	2.18
6.81	-12.48	5.79	1.16	7.63	22.55	3.04
9.45	1.02	5.82	4.69	7.63	63	2.18
7.83	-13.57	3.93	0.93	7.64	20.64	6.56
6.96	-3.32	3.94	3.23	7.64	45.81	6.4
11.8	-2.39	6.48	3.53	7.65	49.05	1.55
5.07	-4.94	5	2.76	7.65	40.52	3.94
11.56	-11.48	3.69	1.36	7.66	24.38	7.16
4.27	-0.12	4.45	4.28	7.66	57.87	5.41
4.31	-0.07	4.41	4.29	7.66	58.07	5.59
4.44	-0.49	4.66	4.15	7.66	56.37	5
10.37	-10.81	5.48	1.44	7.67	25.69	2.94
9.37	0.45	6.84	4.51	7.67	60.23	1.82
10.86	-11.16	4.9	1.38	7.68	24.97	4.42
8.72	7.64	5.13	7.66	7.68	99.76	4.09
14.25	1.96	5.38	5.08	7.68	67.13	3.09
16.4	-6.59	4.88	2.39	7.68	35.67	4.57
6.16	0.62	7.36	4.57	7.68	60.98	1.25
7.25	-1.72	7.02	3.76	7.69	51.38	1.58
10.38	1.56	6.01	4.93	7.69	65.17	2.69
4.44	-0.59	4.62	4.13	7.69	55.82	4.93
8.88	0.7	6.6	4.61	7.69	61.27	1.85
9.14	0.57	6.3	4.56	7.69	60.69	2.24
14.57	-1.73	7.1	3.77	7.69	51.36	1.6
-0.53	-12.49	0.27	-2.59	7.7	34.64	1.33
14.7	-0.25	4.71	4.26	7.7	57.18	5.07
6.66	-3.05	5.49	3.35	7.7	46.54	3.53
5.12	-2.88	6.88	3.39	7.7	47.13	1.58
14.5	-11.34	4.05	1.39	7.71	24.57	6.68
12.94	-9.94	3.86	1.67	7.71	27.45	8.7
8.36	-11.63	6.81	1.37	7.71	24	1.76
4.48	-7.24	7.04	2.23	7.71	33.89	1.6
6.6	-11.49	5.85	1.34	7.72	24.26	3.32

19.81	-4.4	3.63	2.98	7.72	42.01	8.51
7.18	-13.56	4.19	0.99	7.73	20.52	6.48
5.4	-5.05	6.31	2.78	7.73	39.98	2.31
19.36	-3.98	3.45	3.1	7.73	43.34	8.22
11.39	1.16	4.04	4.79	7.73	63.16	5.88
4.35	-0.04	4.59	4.34	7.73	57.94	5.45
15.16	-0.99	6.13	4.04	7.73	54.08	2.45
6.26	-6.91	5.35	2.32	7.74	34.69	4.22
6.83	-5.41	4.76	2.7	7.74	38.86	5.24
12.53	-9.66	4.31	1.74	7.74	28.02	6.14
12.9	-9.81	4.25	1.71	7.74	27.67	6.77
4.25	-7.69	5.1	2.12	7.74	32.65	5.92
8.23	-7.23	4.76	2.25	7.75	33.83	5.85
4.44	-0.37	4.75	4.24	7.75	56.5	4.9
10.23	0.72	6.78	4.65	7.75	61.11	1.93
8.7	7.7	5.4	7.72	7.76	99.56	3.8
5.69	-3.07	7.87	3.37	7.76	46.28	1.36
10.47	-8.04	3.83	2.08	7.76	31.72	6.99
14.25	-3.05	5.27	3.4	7.79	46.25	4.28
10.66	1.55	5.88	4.97	7.79	64.68	3.04
12.15	-1.16	6.17	4.01	7.8	53.13	2.15
14.67	-11.6	4.14	1.39	7.8	23.92	7.25
9.8	0.24	6.11	4.5	7.8	58.85	2.61
10.12	0.08	5.8	4.44	7.8	58.18	3.22
5.35	-5.14	6.39	2.81	7.81	39.48	2.18
9.9	-2.31	5.92	3.64	7.81	48.81	3
6.83	-13.94	4.57	0.98	7.82	19.78	5.48
14.09	1.61	5.53	5.01	7.82	64.86	3.82
19.58	-12.85	5.84	1.23	7.82	21.6	3.25
19.61	-12.6	6.08	1.28	7.82	22.04	2.49
9.42	-15.62	4.69	0.74	7.83	17.21	5.16
14.42	1.72	5.45	5.06	7.83	65.35	3.35
13.88	-0.79	7.92	4.16	7.83	54.51	0.99
6.81	-3.14	5.67	3.4	7.83	45.84	3.46
14.79	-1.9	6.08	3.77	7.84	50.18	2.27
6.03	-7.65	5.26	2.22	7.84	32.54	4.16
4.4	-0.21	4.99	4.34	7.84	56.81	4.67
8.71	7.76	5.31	7.81	7.85	99.38	4.09
8.68	7.74	5.46	7.79	7.85	99.21	3.82
20.63	-9.12	4.15	1.91	7.85	29	6.27
13.12	-0.8	7.7	4.16	7.85	54.37	0.8
4.42	-0.04	4.67	4.4	7.85	57.5	5.8
9.78	0.78	6.96	4.72	7.85	60.96	1.69
10.01	0.82	6.86	4.74	7.85	61.16	1.79

11.97	0.38	6.14	4.57	7.85	59.2	2.47
6.26	0.6	7.49	4.65	7.86	60.15	1.35
8.74	-11.63	7.6	1.46	7.87	23.74	1.34
7.31	-1.94	6.98	3.79	7.88	49.92	1.73
7.38	-1.98	6.93	3.78	7.88	49.77	1.71
13.22	-3.35	5.85	3.37	7.88	44.95	3.24
15.71	-3.35	5.24	3.36	7.88	44.98	3.76
9.87	-1.86	5.74	3.81	7.88	50.21	2.83
-4.39	-17.7	-6.01	-7.39	7.89	34.67	1.14
5.59	-4.73	6.9	2.96	7.89	40.52	1.78
5.79	-4.63	6.91	2.99	7.89	40.83	1.81
6.12	-3.9	6.95	3.19	7.89	43.12	2.15
10.35	-2.05	6.35	3.77	7.89	49.48	2.46
11.26	-2.19	5.67	3.73	7.89	48.97	3.59
11.15	1.48	6.19	5	7.89	63.94	2.89
7.31	-11.98	6.38	1.39	7.89	23.05	2.78
4.98	-7.16	7.11	2.36	7.89	33.66	1.59
5.93	-4.33	6.78	3.07	7.9	41.73	1.9
5.4	-7.48	6.7	2.29	7.9	32.83	2.63
10.76	-2.17	5.75	3.74	7.9	49	3.36
12.6	-3.18	5.43	3.43	7.9	45.49	3.94
15.69	-3.92	5.4	3.22	7.9	43	4.41
15.19	-1.5	6.77	3.96	7.9	51.48	2.03
8.96	-1.64	4.03	3.91	7.91	50.92	6.5
10.6	-1.46	5.85	3.98	7.91	51.6	2.57
15.2	-0.74	6.43	4.21	7.91	54.38	2.15
13.71	-5.82	4.41	2.71	7.92	37.23	6.9
6.98	-3.13	5.82	3.45	7.92	45.59	3.45
5.42	-8.14	6.68	2.15	7.93	31.15	1.52
7.04	-5.48	4.71	2.78	7.93	38.18	5.8
13.45	-9.34	4.33	1.91	7.93	28.35	6.84
4.53	0.07	4.69	4.48	7.93	57.58	6
8.72	7.8	5.87	7.87	7.94	99.01	3.25
13.92	1.71	6.17	5.12	7.94	64.77	2.6
11.05	-0.35	3.45	4.33	7.94	55.87	11.71
19.32	-12.97	6.15	1.28	7.94	21.23	2.96
4.44	0.08	4.68	4.5	7.94	57.61	5.7
4.48	0.09	4.61	4.5	7.94	57.66	6.07
4.5	0.07	4.71	4.5	7.94	57.61	5.81
8.72	7.77	5.7	7.86	7.95	98.81	3.57
8.7	7.74	5.03	7.84	7.95	98.6	4.92
5.21	-4.89	5.88	2.94	7.95	39.86	3.53
6.64	-7.22	5.45	2.37	7.96	33.36	4.68
8.74	7.73	4.96	7.84	7.96	98.43	5.29

10.36	5.21	6.05	6.61	7.96	82.74	2.67
8.21	-4.29	7.23	3.14	7.96	41.68	1.92
11.32	-11.03	5.11	1.57	7.97	24.75	4.31
13.32	-0.59	7.9	4.3	7.97	54.77	0.98
2.19	-12.99	0.5	-3.07	7.98	34.69	0.82
11.89	-2.59	5.93	3.65	7.98	47.24	3.18
12.13	-10.59	4.34	1.71	7.98	25.6	6.27
6.95	-7.09	7.12	2.43	7.98	33.64	1.66
12.97	-1.22	6.24	4.09	7.99	52.23	2.47
11.54	-2.46	5.84	3.7	7.99	47.68	3.4
12.24	-2.71	5.8	3.62	7.99	46.82	3.27
19.04	-8.57	4.46	2.11	7.99	29.98	5.47
14.5	1.2	5.95	4.96	8.01	62.16	3.37
14.2	-9.79	4.44	1.86	8.01	27.23	7.25
6.24	-9.87	5.79	1.84	8.01	27.04	3.65
13.46	-9.31	3.34	1.97	8.01	28.26	9
11.09	-0.29	7.23	4.42	8.01	55.82	2.14
12.72	-2.76	6.7	3.62	8.02	46.53	1.92
4.48	-0.05	5.16	4.49	8.02	56.75	5.01
14.41	1.62	6.14	5.13	8.03	63.97	2.77
14.4	-1.32	8.05	4.09	8.03	51.71	0.73
4.44	-0.07	4.99	4.5	8.03	56.64	5.02
11.34	0.2	6.5	4.6	8.03	57.77	2.1
5.3	-7.92	6.85	2.27	8.04	31.45	1.94
14.46	-1.01	8.18	4.19	8.04	52.85	1.23
16.09	-3.58	5.38	3.4	8.05	43.69	3.97
16.93	-6.5	5.19	2.61	8.05	35.04	4.71
11.76	-11.56	4.97	1.52	8.06	23.56	5.44
-2.08	-16.43	-5.2	-6.23	8.06	34.71	1.48
8.8	7.81	5.44	7.92	8.06	98.35	4.47
21.17	-9.8	5.32	1.9	8.06	27.11	4.81
15.17	-0.23	5.04	4.46	8.06	55.86	5.46
14.93	-11.32	4.58	1.6	8.07	24	7.08
7.36	-5.66	5.24	2.81	8.07	37.31	5.12
12.71	0.73	6.02	4.81	8.07	59.85	3.5
7.85	-3.15	4.47	3.51	8.07	45.04	6.19
19.34	-9.1	4.89	2.05	8.07	28.61	5.58
15.14	-1.47	7.44	4.06	8.07	51.01	1.89
7.5	-3.56	6.53	3.41	8.08	43.69	2.36
7.05	-11.35	5.92	1.58	8.09	23.91	3.9
8.79	-4.01	4.63	3.28	8.09	42.19	6.31
20.83	-9.19	4.53	2.04	8.09	28.36	5.38
14.49	-5.48	4.57	2.89	8.09	37.75	7.02
7.39	-2.57	7.05	3.72	8.1	46.93	2.05

20.52	-4.3	3.73	3.22	8.1	41.25	9.22
7.23	-3.22	5.97	3.52	8.1	44.75	3.72
4.5	0.08	5.13	4.59	8.11	56.95	5.13
7.95	-6.31	5.85	2.67	8.12	35.39	3.01
4.58	0.12	4.81	4.61	8.12	57.06	6.14
8.81	7.89	5.36	8	8.13	98.37	5.2
13.61	-2.8	6.64	3.66	8.13	46.07	2.47
12.12	1.51	6.56	5.14	8.13	63.05	2.59
13.28	1.03	6.08	4.95	8.13	60.9	3.39
4.57	0.12	4.86	4.61	8.13	57.03	6.11
11.73	1.75	6.21	5.23	8.14	64.1	2.93
8.7	-3.91	12.24	4.89	8.15	34.73	0.86
7.75	-4.01	6.89	3.32	8.15	42.04	1.97
12.33	1.1	5.97	4.99	8.15	61.13	3.4
13.6	0.95	6.05	4.94	8.15	60.47	3.21
4.83	-0.67	4.92	4.34	8.15	53.78	5.08
9.17	-11.63	7.42	1.63	8.16	23.29	1.59
5.31	-7.34	6.95	2.47	8.17	32.58	1.93
21.35	-9.52	4.65	2.01	8.17	27.5	6.49
8.49	-3.16	4.55	3.56	8.17	44.73	6.8
10.32	1.1	6.26	5	8.17	61.02	2.7
11.43	-1.26	6.37	4.18	8.17	51.41	2.78
8.56	-5.66	6.32	2.87	8.18	37.02	2.42
12.84	-10.11	3.89	1.91	8.18	26.24	7.73
9.14	-5.26	4.35	3	8.19	38.16	7.48
4.53	0.18	5.22	4.67	8.2	57.02	5.34
7.9	-3.99	7.93	3.36	8.21	41.9	1.6
7.48	-2.97	7.36	3.66	8.22	45.21	1.68
6.64	0.47	7.85	4.8	8.22	58.12	1.43
6.49	0.59	8.18	4.84	8.22	58.64	1.06
17.83	-7.55	4.89	2.47	8.24	31.9	6.55
9.68	-11.59	7.69	1.68	8.24	23.23	1.53
15.44	-0.9	5.17	4.32	8.25	52.54	5.37
19.42	-7.67	5.1	2.45	8.25	31.59	5.96
13.64	-1.66	6.71	4.09	8.26	49.66	2.4
9.07	-2.9	4.58	3.69	8.26	45.31	7.22
7.87	-11.69	6.28	1.65	8.26	23.02	3.38
14.74	-9.79	4.99	2.02	8.27	26.75	6.49
5.32	-4.87	6.18	3.12	8.27	39.08	4.21
10.94	-8.25	5.03	2.32	8.27	30.16	5.24
7	-7.17	6.25	2.56	8.28	32.78	3.72
19.87	-11.98	7.3	1.64	8.28	22.45	2.01
7.76	-5.84	5.34	2.89	8.29	36.27	5.56
4.58	0.23	5.32	4.74	8.29	56.86	5.44

4.55	0.19	5.06	4.73	8.3	56.66	5.8
11.12	-1.85	6.52	4.04	8.31	48.81	2.29
-3.5	-17.24	-4.3	-6.97	8.33	34.74	0.74
10.8	5.44	6.99	6.9	8.33	82.02	2.02
13.97	-8.34	5.06	2.36	8.33	29.83	7.09
15.98	-1.73	5.5	4.1	8.33	49.19	5.81
11.21	-8.38	5.28	2.33	8.33	29.73	5.51
17.61	-7.11	4.94	2.63	8.34	32.78	5.83
19.05	-12.01	6.84	1.67	8.34	22.3	2.55
5.34	-5.12	5.95	3.1	8.35	38.15	3.94
5.42	-4.75	6.96	3.2	8.35	39.22	2.95
15.43	-9.71	4.72	2.09	8.37	26.73	7.68
20.83	-4.34	4.24	3.35	8.37	40.38	7.75
8.48	-7.68	5.84	2.49	8.37	31.33	5.43
7.49	-3.35	6.08	3.63	8.37	43.5	3.96
14.65	-1.88	6.5	4.07	8.39	48.43	2.48
13.43	-7.57	4.31	2.56	8.39	31.55	8.21
8.17	-13.57	4.09	-0.9	8.4	25.67	1.01
8.09	-4.45	7.53	3.35	8.42	39.93	1.7
15.92	-1.38	5.49	4.26	8.42	50.13	5.24
10.23	-11.88	7.62	1.73	8.42	22.43	1.75
10.84	-11.93	7.32	1.73	8.42	22.34	2.11
19.97	-12.71	7.1	1.6	8.42	20.98	2.01
6.89	-2.81	7.29	3.81	8.43	45.07	2.37
7.39	-2.79	7.27	3.81	8.43	45.16	2.29
6.99	-9.58	9.36	2.14	8.43	26.9	1.6
14.88	-11.42	5.17	1.79	8.43	23.25	5.89
8.9	8.18	5.84	8.3	8.43	98.29	4.68
8.15	-4.29	7.24	3.4	8.43	40.36	1.81
20.56	-9.64	5.29	2.14	8.43	26.77	5.64
8.43	-11.54	6.65	1.78	8.43	23.03	3.1
11.41	-8.32	5.08	2.4	8.43	29.68	6.5
7.51	-11.36	6.02	1.78	8.44	23.35	4.3
12.21	-11.43	5.02	1.76	8.44	23.22	5.73
6.34	-3.82	7.28	3.51	8.44	41.81	2.22
21.02	-10.01	5.3	2.07	8.44	25.99	5.62
15.83	-5.46	5.2	3.09	8.44	36.93	6.44
14.18	-8.55	4.54	2.38	8.44	29.12	6.93
13.73	-0.89	7.82	4.45	8.44	51.9	1.59
13.06	-7.56	4.93	2.6	8.45	31.43	7.02
7.98	-3.64	6.65	3.58	8.45	42.33	3.31
15.98	-9.64	4.98	2.15	8.46	26.71	7.19
7.71	-3.45	6.24	3.65	8.46	42.9	3.68
7.88	-3.76	7.2	3.56	8.47	41.88	1.88

6.77	-9.92	5.85	2.09	8.47	26.11	4.96
8.93	8.16	6.24	8.32	8.48	97.85	3.79
8.93	8.12	6.11	8.3	8.49	97.56	4.04
8.97	8.06	6.4	8.26	8.49	97.18	3.49
9.03	8.01	6.14	8.25	8.49	96.81	4
9.07	7.96	5.85	8.22	8.5	96.43	4.75
6.78	0.54	7.8	4.97	8.5	57.34	1.72
7.56	-7.18	7.83	2.7	8.5	32.27	1.66
11.61	-11.91	7	1.77	8.5	22.25	2.58
15.14	-11.64	5.33	1.79	8.51	22.72	6.2
12.24	-0.79	6.01	4.52	8.51	52.03	3.89
6.31	-0.66	5.27	4.53	8.51	52.56	5.68
12.82	-11.82	5.34	1.74	8.52	22.38	6.13
10.07	-3.06	4.55	3.79	8.52	43.97	8.13
15.29	-1.21	8.07	4.38	8.52	50.44	1.07
12.2	-0.82	8.15	4.51	8.52	51.88	1.5
19.62	-7.85	5.23	2.57	8.52	30.6	6.3
7.59	-3.71	6.85	3.61	8.53	41.89	2.61
16.35	-5.12	5.16	3.23	8.53	37.68	6.65
14.65	-1.05	8.27	4.44	8.53	51	0.97
14.27	-0.94	7.56	4.48	8.53	51.41	1.58
7.45	-7.8	6.34	2.57	8.55	30.66	4.01
8.18	-5.89	5.77	3.02	8.55	35.47	5.22
16.48	-10.22	5.55	2.09	8.55	25.37	5.9
11.63	-1.88	7	4.17	8.55	47.92	2.3
11.42	-0.72	4.35	4.54	8.56	52.13	11.14
11.23	5.47	7.16	7.03	8.57	80.87	2.24
13.27	-11.71	7.12	1.85	8.57	22.51	2.81
8.94	8.3	6.04	8.43	8.58	98.11	4.19
9.16	8	5.93	8.28	8.59	96.09	4.72
9.27	7.97	6.04	8.26	8.59	95.88	4.7
20.87	-10.12	5.83	2.13	8.59	25.5	5.14
14.56	-0.76	8.6	4.57	8.59	51.87	0.98
14.16	-1.47	6.67	4.34	8.6	49.21	2.83
7.95	-4.27	7.67	3.49	8.6	39.98	1.86
12.3	5.99	6.35	7.29	8.6	83.62	4.24
12.52	-11.47	6.99	1.91	8.6	22.89	2.7
7.34	-7.21	7.07	2.74	8.6	31.98	2.72
8.1	-4.17	7.08	3.53	8.61	40.23	2.66
5.52	-4.62	7.47	3.37	8.61	38.89	2.03
9.13	-11.64	6.54	1.86	8.61	22.57	3.83
13.57	-0.59	7.94	4.64	8.61	52.46	1.5
7.96	-7.58	7.8	2.66	8.61	31.04	2.15
12.59	-11.77	5.21	1.81	8.62	22.32	6.04

6.49	-3.39	7.31	3.73	8.62	42.63	2.32
6.62	-3.12	7.48	3.81	8.62	43.5	2.02
6.77	-2.79	7.44	3.91	8.62	44.59	1.95
5.44	-7.31	7.16	2.73	8.62	31.67	2.79
10.74	-5.17	4.51	3.26	8.62	37.29	8.8
8.52	-4.88	9.52	3.34	8.62	38.12	1.64
20.35	-10.09	5.59	2.15	8.62	25.5	5.38
22.66	-5.34	4.06	3.22	8.62	36.83	11.81
13.9	-0.89	8.3	4.54	8.62	51.28	1.42
18.06	-7.66	4.84	2.66	8.62	30.83	6.74
7.96	-11.26	6.28	1.9	8.63	23.24	4.38
10.67	-5.53	4.91	3.17	8.63	36.28	7.77
14.08	-0.79	7.73	4.58	8.63	51.64	1.55
21	-10.29	5.68	2.13	8.64	25.06	5.6
11.79	-1.89	6.99	4.21	8.64	47.58	2.66
7.19	-2.69	7.22	3.96	8.65	44.83	2.07
14.13	-11.79	6.71	1.88	8.65	22.23	3.23
7.68	-7.2	7.18	2.77	8.65	31.89	2.69
10.66	-4.86	5.33	3.37	8.67	38.04	7.48
22.02	-4.89	4.42	3.37	8.67	37.98	9.48
-0.23	-20.22	-4.03	-6.88	8.68	25.65	0.9
6.91	0.5	8.02	5.05	8.68	56.47	1.87
7.61	-2.79	7.91	3.95	8.69	44.36	2.11
9.41	8	6.1	8.33	8.69	95.43	4.79
7.94	-3.87	7.76	3.66	8.69	40.94	1.81
18.81	-10.35	5.4	2.15	8.7	24.85	6.67
16.95	-5.17	5.85	3.31	8.7	37.12	5.78
8.36	-4.65	6.46	3.45	8.71	38.56	3.28
17.92	-10.04	5.46	2.22	8.71	25.45	6.56
8.78	-6.02	6.93	3.07	8.71	34.75	2.58
18.39	-7.84	5.29	2.67	8.71	30.21	6.56
10.72	-5.51	5	3.23	8.72	36.11	7.75
23.49	-5.91	4.63	3.13	8.72	35.03	10.77
13.05	-0.91	8.33	4.6	8.72	50.86	1.47
7.76	-7.2	7.57	2.81	8.72	31.74	2.07
19.68	-7.79	5.36	2.69	8.72	30.34	6.41
11.5	-8.06	5.03	2.62	8.72	29.71	6.79
8.62	-6.06	5.91	3.07	8.73	34.6	5.54
20.41	-8.99	5.63	2.44	8.73	27.58	5.38
21.42	-4.37	4.5	3.54	8.73	39.33	8.47
11.73	-0.5	8.92	4.73	8.73	52.36	0.99
11.35	-0.4	8.72	4.77	8.73	52.71	1.1
7.34	-9.7	10.03	3.05	8.74	23.81	1.15
7.03	-9.89	5.9	2.25	8.74	25.7	5.39

12	-0.75	8.31	4.66	8.74	51.36	1.29
11.59	-1.87	7.23	4.26	8.74	47.33	2.66
9.55	7.82	6.28	8.27	8.76	93.81	4.51
9.48	7.97	6.13	8.35	8.77	94.7	4.77
20.47	-10.43	6.08	2.18	8.78	24.57	5.45
7.94	-7.13	7.24	2.86	8.78	31.78	2.76
17.67	-5.26	5.69	3.34	8.79	36.64	6.62
5.65	-4.72	8.06	3.44	8.79	38.14	2.25
12.48	-0.66	8.77	4.71	8.79	51.55	1.1
13.44	-11.26	5.45	2	8.8	22.97	6.83
15.1	-11.14	5.38	2.05	8.8	23.19	6.51
10.99	-2.7	5.02	4.04	8.8	44.34	7.65
12.47	6.34	6.08	7.54	8.8	84.55	5.17
13.28	-0.78	8.61	4.68	8.8	51.05	1.2
12.51	-0.72	9.54	4.7	8.8	51.29	0.69
11.66	-7.94	5.47	2.69	8.8	29.81	6.39
9.41	-5.68	7.15	3.21	8.81	35.41	2.37
13.2	-11.48	5.54	1.96	8.81	22.56	6.01
19.93	-9.41	5.23	2.4	8.81	26.56	7.21
19.85	-11.98	7.58	1.95	8.81	21.68	1.65
7.6	-9.93	6.71	2.29	8.82	25.49	4.32
13.22	-7.35	4.96	2.85	8.82	31.16	7.43
13.94	-0.78	8.42	4.68	8.82	50.99	1.26
13.46	-0.66	8.36	4.73	8.82	51.43	1.24
11.2	1.48	7.23	5.48	8.82	60.03	2.39
7.93	-7.91	5.91	2.7	8.83	29.82	5.87
23.61	-5.5	4.46	3.3	8.83	35.88	11.32
7.26	-9.93	6.02	2.3	8.83	25.46	5.17
8.32	-3.72	7.08	3.77	8.83	41.02	3.45
11.53	5.67	7.71	7.25	8.84	80.5	1.94
8.42	-11.19	6.18	2.04	8.85	23	5.27
9.63	7.79	6.51	8.29	8.85	93.09	4.23
23.2	-5.26	4.12	3.37	8.85	36.46	11.92
8.22	-7.2	6.96	2.88	8.85	31.47	3.31
6.82	-4.29	8.55	3.59	8.86	39.23	2.44
15.07	-10.73	5.44	2.15	8.86	23.85	6.62
14.57	-2.13	7.38	4.27	8.86	46.03	1.92
10.73	5.79	7.72	7.33	8.86	81.09	2.27
6.26	-2.9	8.7	4.02	8.87	43.48	1.31
7.21	0.45	7.99	5.13	8.87	55.55	2.16
7.5	-0.67	5.67	4.72	8.87	51.21	5.72
15.04	-11.1	5.5	2.1	8.88	23.13	7.03
6.15	-2.87	8.55	4.03	8.88	43.54	1.35
16.15	-2.34	6.37	4.2	8.88	45.32	4.59

5.85	-5.01	7.92	3.42	8.89	37.05	1.99
18.35	-11.98	8.12	1.99	8.89	21.56	1.83
8.51	-7.23	6.47	2.9	8.89	31.29	4.37
9	-6.19	6.59	3.14	8.9	33.87	4.51
6.1	-4.57	7.66	3.54	8.9	38.31	2.27
12.82	-0.38	8.61	4.87	8.9	52.22	1.19
8.8	-7.54	6.65	2.83	8.9	30.54	4.14
5.4	-7.09	7.28	2.94	8.91	31.58	2.6
8.42	-7.74	5.95	2.79	8.91	30.05	5.98
23.88	-5.73	5.19	3.28	8.91	35.05	9.01
6.25	-4.39	7.24	3.6	8.91	38.77	2.97
11.73	-7.59	5.82	2.83	8.91	30.39	5.81
13.56	-11.46	7.94	2.1	8.92	22.41	1.8
12.55	6.71	6.63	7.78	8.93	86	4.31
10.07	5.73	8.01	7.33	8.94	80.3	2.08
13.03	-0.49	6.55	4.84	8.95	51.63	3.91
11.02	5.89	7.51	7.41	8.96	81.12	2.47
5.99	-7.88	8.31	2.8	8.97	29.61	2.21
6.4	-8.53	8.08	2.66	8.97	28.14	1.68
9.02	-7.43	6.83	2.9	8.97	30.65	3.87
9.57	-7.63	6.77	2.85	8.97	30.18	4.1
11.84	-7.93	6.17	2.79	8.97	29.49	5.45
18.7	-7.73	5.58	2.84	8.98	29.93	7.12
18.6	-8.06	5.56	2.77	8.98	29.18	6.42
10.76	-5.04	5.29	3.5	9	36.73	7.39
5.86	-4.95	7.37	3.49	9	36.97	3
13.75	-7.22	5.25	2.98	9	31.09	7.66
8.87	-11.19	6.63	2.13	9.01	22.76	4.72
9.62	-6.22	7.09	3.18	9.01	33.55	3.05
9.75	7.91	7.02	8.45	9.03	92.72	3.65
19.71	-7.67	6.11	2.89	9.03	29.96	5.95
11.77	5.77	8.25	7.4	9.04	79.96	1.66
8.96	-7.76	6.23	2.86	9.05	29.72	5.94
10.47	5.81	7.83	7.42	9.05	80.16	2.3
10.25	5.86	7.75	7.45	9.05	80.44	2.19
7.3	0.37	8.29	5.19	9.05	54.56	1.84
0.03	-17.21	1.31	-3.46	9.06	23.82	0.76
19.65	-5.11	6.26	3.52	9.06	36.37	6.4
14.94	-11.23	8.27	2.22	9.06	22.61	1.99
14.04	-4.14	5.76	3.78	9.07	39.1	7.45
9.1	-4.96	8	3.56	9.07	36.77	2.35
6.5	-4.24	8.2	3.72	9.07	38.83	2.62
18.07	-11.58	8.31	2.17	9.07	21.98	1.51
6.52	-2.85	8.32	4.14	9.07	43.06	1.85

9.29	-7.53	6.69	2.93	9.07	30.22	4.21
10	-7.91	6.53	2.84	9.07	29.34	5.05
14.31	-1.84	7.76	4.46	9.08	46.37	1.99
8.69	-8.04	6.94	2.81	9.09	29	4.6
11.69	-8.14	6.39	2.8	9.09	28.78	5.56
5.52	-4.88	6.83	3.57	9.1	36.93	3.25
6.12	-4.61	7.18	3.64	9.1	37.66	3.09
12.56	7.2	6.94	8.11	9.1	87.89	3.95
18.44	-5	6.03	3.58	9.11	36.56	6.26
10.74	8.75	9.58	8.92	9.12	97.56	1.24
15.47	-11.62	8.83	2.19	9.13	21.83	1.53
6.68	-2.87	8.41	4.17	9.13	42.82	1.76
10.77	8.93	8.64	9.03	9.15	98.55	1.58
6.98	0.61	8.08	5.33	9.15	55.16	1.95
10.74	8.86	8.9	8.99	9.15	98.09	1.37
15.03	-11.13	6.07	2.25	9.16	22.65	6.17
10.44	-11.52	6.81	2.2	9.16	21.96	4.51
11.37	-8.01	6.43	2.87	9.16	28.95	5.27
11.51	-8.29	6.29	2.81	9.16	28.32	5.61
11.77	-8.13	6.2	2.85	9.16	28.66	5.82
11.78	-7.72	6.31	2.94	9.16	29.6	5.46
14.63	-10.57	5.61	2.35	9.17	23.66	7.83
6	-4.64	8.06	3.67	9.17	37.4	2.62
18.76	-8.41	5.85	2.8	9.17	28.04	6.84
6.84	-2.8	8.36	4.21	9.17	42.92	1.86
9.35	-11.23	6.56	2.22	9.18	22.43	5.17
23.86	-6.27	5.53	3.3	9.18	33.04	9.34
15.89	-11.63	8.2	2.21	9.18	21.74	1.71
10.09	-7.75	6.11	2.94	9.19	29.48	6.9
12.03	-4.29	5.51	3.8	9.19	38.35	7.18
11.78	-1.82	5.6	4.51	9.19	46.09	10.69
18.76	-8.16	6.01	2.87	9.19	28.54	6.06
8.66	-3.71	7.14	3.97	9.19	40.07	3.97
10.08	7.92	7.49	8.53	9.2	91.68	3.26
8.21	-8.14	7.79	2.87	9.2	28.57	2.27
10.94	7.35	7.05	8.24	9.21	88.15	3.95
10.86	7.09	6.94	8.11	9.21	86.56	4.24
15.64	-2.48	8.29	4.35	9.21	43.83	1.71
15.87	-2.62	6.29	4.29	9.21	43.41	5.42
9.92	7.94	6.95	8.54	9.22	91.71	4.13
10.7	7.38	6.99	8.26	9.22	88.24	4.05
10.84	6.89	7.23	8.03	9.23	85.26	3.96
7.51	0.36	8.99	5.28	9.23	53.86	1.39
7.92	-2.64	8.61	4.3	9.24	43.23	2.06

9.27	-4.28	8.64	3.84	9.24	38.27	2.07
18.86	-4.86	5.69	3.69	9.24	36.61	7.16
10.51	-8.34	6.78	2.85	9.24	28.04	4.82
14.36	-10.45	6.2	2.42	9.25	23.76	6.34
10.54	7.55	7.28	8.35	9.25	89.13	3.58
10.53	7.5	7.1	8.33	9.25	88.81	3.93
16.02	-2.3	6.75	4.41	9.25	44.32	4.14
13.61	-11.2	6.19	2.27	9.26	22.38	5.73
11.68	-7.89	6.09	2.96	9.26	29.02	6.25
9.51	-8.03	6.24	2.93	9.28	28.68	5.98
11.89	5.76	8.59	7.52	9.28	78.64	1.67
23.88	-5.67	5.79	3.5	9.28	34.35	7.21
15.63	-4.09	6.3	3.91	9.29	38.67	5.91
13.64	-1.96	8.03	4.53	9.3	45.26	2.42
13.88	-1.77	8.01	4.59	9.3	45.89	2.22
14.89	-2.1	7.8	4.51	9.3	44.81	2.27
10.19	7.98	7.46	8.6	9.3	91.48	3.34
10.43	7.94	7.42	8.58	9.3	91.25	3.42
10.56	7.78	7.32	8.5	9.3	90.23	3.55
13.79	-0.28	7.03	5.1	9.3	51.22	3.87
-6.37	-23.72	-8.88	-9.47	9.31	23.85	1.38
9.79	-10.86	6.98	2.36	9.31	22.92	4.47
11.91	5.76	7.64	7.53	9.31	78.53	3
9.88	5.83	8.35	7.56	9.31	78.84	2.21
19.88	-7.06	6.28	3.19	9.31	30.81	5.69
-7.44	-25.93	-11.79	-12.31	9.33	25.65	1
7.53	5.69	9.14	7.5	9.33	78.06	1.41
10.68	-8.16	7.12	2.93	9.33	28.27	4.1
13.61	-11.18	6.64	2.32	9.34	22.29	5.08
20.8	-5.89	6.26	3.48	9.34	33.65	6.96
11.04	-11.25	7.09	2.34	9.34	22.16	4.49
12.7	7.47	7.38	8.36	9.34	88.07	3.78
11.15	7.89	9.79	8.57	9.34	90.69	0.83
10.7	8.57	9.67	8.93	9.34	94.99	0.92
13.12	-4.27	6.23	3.9	9.35	38	6.2
14.16	-7.54	5.93	3.11	9.36	29.59	6.87
9.17	5.87	7.78	7.6	9.36	78.88	3.04
10.23	-10.89	6.84	2.39	9.37	22.76	4.87
10.34	-7.37	6.17	3.12	9.37	29.97	7.25
13.96	-4.5	6.06	3.84	9.37	37.3	7.2
9.94	-7.07	6.27	3.19	9.38	30.66	6.53
10.72	7.02	7.89	8.16	9.38	85.25	2.88
23.92	-5.61	5.36	3.57	9.38	34.27	9.52
9.04	-3.78	7.4	4.05	9.38	39.33	4.09

10.99	9.38	8.95	9.39	9.38	100	1.5
5.77	-4.84	7.25	3.73	9.39	36.32	3.25
7.68	5.64	8.97	7.51	9.39	77.45	1.43
7.64	0.37	9.04	5.38	9.42	53.25	1.53
8.51	-0.61	6.34	5.03	9.42	49.56	5.62
18.86	-8.59	6.42	2.91	9.43	27.15	6.38
19.86	-7.05	6.55	3.25	9.43	30.6	5.97
11.19	7.78	9.66	8.55	9.43	89.44	1.01
1.75	-14.65	3.34	-1.86	9.44	25.64	0.75
12.51	-3.11	5.71	4.26	9.44	41.2	8
14.22	-7.87	6.47	3.07	9.44	28.69	5.89
8.31	-2.83	8.17	4.35	9.44	42.05	2.5
8.53	-2.83	8.05	4.34	9.44	42.08	2.7
10.66	-11.11	6.75	2.4	9.45	22.23	5.62
11.13	-8.71	6.12	2.87	9.45	26.87	7.44
11.63	-9.2	6.05	2.77	9.45	25.86	7.7
22.28	-5.96	6.1	3.52	9.45	33.23	7.62
22.96	-5.23	5.82	3.71	9.45	35.09	8.68
23.11	-5.45	6.2	3.65	9.45	34.53	7.51
23.99	-5.55	6.07	3.63	9.45	34.28	7.74
10.77	-9.29	6.99	2.77	9.45	25.67	5.05
12.84	7.51	7.08	8.44	9.45	87.71	4.79
15.57	-2.26	7.87	4.54	9.46	43.81	2.68
10.49	-9.09	7	2.81	9.46	26.08	4.55
10.82	9.46	8.1	9.47	9.46	100	2.56
10.71	6.85	7.7	8.12	9.47	83.72	3.29
10.47	-6.64	7.66	3.33	9.47	31.51	2.94
10.71	6.72	7.8	8.06	9.48	82.93	3.19
11.42	6.13	8.09	7.79	9.48	79.59	2.25
10.65	9.49	9.43	9.5	9.49	100	1.19
11.88	5.94	8.12	7.7	9.5	78.5	2.54
14.52	-10.24	5.85	2.62	9.5	23.75	9.21
11.6	-11.41	7.71	2.4	9.5	21.64	3.82
12.5	-2.79	7.4	4.39	9.5	42	3.2
10.91	9.5	9.44	9.51	9.5	100	1.14
1.89	-14.61	3.36	-1.82	9.51	25.62	0.75
13.69	-11.12	7.02	2.42	9.51	22.14	4.66
7.88	0.54	8.94	5.5	9.51	53.53	1.6
10.15	-5.99	8.63	3.52	9.52	32.98	1.55
13.65	-4.11	6.5	4.03	9.52	38.03	5.89
8.45	-10.02	6.5	2.67	9.52	24.13	6.62
14.34	-6.61	7.17	3.39	9.52	31.46	4.77
14.76	-6.54	7.1	3.41	9.52	31.64	5.07
14.05	-9.73	6.3	2.72	9.53	24.67	6.37

10.65	9.53	8.53	9.53	9.53	100	2.14
18.84	-8.38	6.3	3.01	9.53	27.41	6.29
19.92	-6.45	6.62	3.44	9.53	31.83	5.42
10.94	8.59	9.44	9.03	9.53	93.87	1.13
13.82	-9.68	6.62	2.73	9.54	24.77	5.73
14.35	-4.34	5.97	3.98	9.54	37.32	7.87
8.85	-5.25	8.77	3.75	9.54	34.85	1.86
9.02	-5.39	8.52	3.71	9.54	34.48	1.97
20.35	-5.46	6.2	3.7	9.54	34.29	7.06
7.3	-3.83	7.92	4.1	9.54	38.77	3.28
9.2	5.92	7.84	7.71	9.54	78.17	3.18
11.11	9.54	9.56	9.55	9.54	100	1.06
10.85	-7.37	6.29	3.22	9.55	29.61	7.51
21.46	-5.71	6.71	3.64	9.55	33.64	6.4
23.56	-5.43	5.91	3.71	9.55	34.36	7.81
10.65	9.55	7.63	9.56	9.55	100	3.12
11.17	7.41	9.04	8.43	9.55	86.51	1.74
6.51	-4.23	7.46	3.99	9.56	37.6	3.36
19.73	-7.35	6.61	3.25	9.56	29.65	6.23
11.66	9.56	9.28	9.56	9.56	100	1.4
11.4	9.56	9.9	9.56	9.56	100	0.9
14.53	0.16	7.36	5.4	9.57	51.89	4.11
7.42	5.74	9.86	7.64	9.57	77.02	0.96
10.78	9.46	9.87	9.51	9.57	99.27	0.91
13.14	-9.94	7.17	2.72	9.58	24.2	4.95
10.85	9.44	10.22	9.5	9.58	99.01	0.63
9.78	6.06	10.28	7.8	9.59	78.66	0.79
10.72	9.59	10.37	9.6	9.59	100	0.65
13.45	-1.97	8.89	4.69	9.6	44.34	1.69
13.29	-12.12	6.98	2.31	9.61	20.29	5.87
9.55	6.01	8.5	7.79	9.61	78.27	2.43
7.76	0.47	9.07	5.52	9.61	52.93	1.55
19.4	-7.27	6.73	3.3	9.61	29.74	6.48
9.39	-2.7	7.88	4.48	9.61	41.98	3.38
7.39	6	10.21	7.78	9.61	78.21	0.86
7.37	6.01	10.1	7.79	9.61	78.3	0.82
11.53	-11.46	7.22	2.43	9.62	21.4	4.91
12.13	-9.65	6.46	2.79	9.62	24.68	6.79
13.02	-10.2	6.08	2.68	9.62	23.64	8.13
6.41	-4.47	6.86	3.96	9.62	36.75	3.75
9.4	5.98	8.2	7.78	9.62	78.05	2.74
7.4	5.87	9.74	7.72	9.62	77.45	1.06
10.75	9.55	9.68	9.58	9.62	99.55	1.09
13.83	-6.81	14.4	5.46	9.63	23.85	0.85

11.91	-11.56	6.87	2.42	9.63	21.21	5.91
12.51	-9.79	6.23	2.76	9.63	24.4	7.33
8.86	-2.45	9.91	4.55	9.63	42.71	1
10.77	6.94	8.11	8.24	9.63	83.34	2.93
11.84	5.98	8.31	7.78	9.63	78.02	2.53
23.28	-5.39	6.37	3.77	9.63	34.27	6.85
10.36	-9.23	7.33	2.88	9.63	25.5	4.25
11.4	-9.59	6.91	2.81	9.63	24.78	5.42
11.47	-7.55	6.26	3.23	9.64	29.03	7.82
23.28	-5.68	6.2	3.69	9.64	33.53	7.3
9.64	5.91	8.75	7.76	9.64	77.6	2.21
8.06	0.55	9.38	5.56	9.64	53.17	1.35
9.45	-3.87	7.75	4.17	9.64	38.41	3.92
8.19	-2.6	9.19	4.52	9.65	42.2	1.53
13.14	7.76	7.66	8.66	9.65	88.02	4.35
10.7	9.65	8.31	9.66	9.65	100	2.8
12.11	1.39	7.53	5.87	9.65	56.4	3.22
9.68	-2.71	8.12	4.49	9.65	41.87	3.19
13.58	-11.65	6.83	2.42	9.66	21.02	5.68
6.16	-7.75	8.9	3.21	9.66	28.56	1.91
10.74	6.56	8.28	8.07	9.66	81.05	2.83
8.86	-9.93	6.34	2.77	9.66	24.08	7.53
12.88	-9.6	7.02	2.83	9.66	24.72	5.1
13.67	-10.3	6.99	2.7	9.66	23.39	5.54
8.19	0.58	9.56	5.58	9.66	53.18	1.4
13.58	-11.64	6.86	2.42	9.67	21.02	5.53
12.95	-6.71	6.43	3.43	9.67	30.91	7.92
8.56	-2.52	9.66	4.56	9.67	42.39	1.14
6.69	-4.09	8.32	4.1	9.67	37.71	2.75
15.6	-4.79	6.84	3.93	9.67	35.76	4.96
21.9	-5.14	6.17	3.85	9.68	34.81	7.76
10.67	9.16	8.99	9.39	9.68	96.6	2.02
11.97	9.68	9.64	9.68	9.68	100	1.17
13.49	-9.99	6.8	2.75	9.69	23.93	5.96
11.93	-7.62	6.11	3.24	9.69	28.78	8.7
6.12	-7.65	8.7	3.26	9.72	28.66	2.27
6.67	-4.12	8.17	4.12	9.73	37.47	2.89
18.79	-8.05	6.75	3.18	9.73	27.78	6.02
11.93	6.17	8.99	7.92	9.74	78.5	2.08
10.1	6.34	9.28	8	9.74	79.41	1.43
10.76	9.71	9.64	9.72	9.74	99.79	1.18
-1.58	-19.42	-3.17	-5.47	9.75	23.86	1.44
9.31	-2.32	10.11	4.65	9.75	42.79	0.9
7.13	-3.84	8.1	4.2	9.75	38.21	2.9

13.76	-10.57	7.04	2.69	9.75	22.77	5.99
10.75	6.6	8.74	8.14	9.76	80.68	2.23
11.74	6.02	8.73	7.85	9.76	77.55	2.18
10.28	-5.74	9.78	3.74	9.76	33.08	1.91
13.13	-10.84	7.43	2.65	9.76	22.26	5.3
10.18	-2.72	8.34	4.54	9.76	41.52	2.9
11.06	-2.62	8	4.58	9.76	41.82	3.24
6.26	-7.99	9.4	3.22	9.77	27.81	1.57
15.79	-8.51	6.85	3.12	9.77	26.71	6.63
12.14	-10.24	7.63	2.77	9.77	23.34	4.18
12.62	-10.35	7.23	2.75	9.77	23.11	5.18
15.07	-6.9	7.29	3.46	9.77	30.26	4.92
10.71	9.77	8.36	9.78	9.77	100	2.88
9.4	6.06	8.56	7.89	9.77	77.66	2.63
18.73	-7.7	6.76	3.29	9.77	28.44	6.01
10.89	-2.73	7.76	4.55	9.77	41.46	3.74
12.8	-11.57	6.97	2.5	9.78	20.97	5.88
13.09	-11.58	7.07	2.49	9.78	20.97	5.52
13.43	-11.37	7.06	2.53	9.78	21.31	5.46
14.52	-4.23	6.55	4.13	9.78	37.05	7.22
6.78	-4.03	8.36	4.17	9.78	37.6	2.82
6.84	-4.09	8.5	4.15	9.78	37.42	2.83
11.87	-10.04	6.77	2.82	9.78	23.68	7.19
11.12	-3.35	7.46	4.37	9.78	39.57	4.41
1.23	-19.37	-3.5	-6.07	9.79	25.57	1.09
12.54	-11.34	6.82	2.54	9.79	21.35	6.13
12.51	-7.03	6.3	3.43	9.79	29.92	7.99
8.83	-5.48	8.49	3.82	9.79	33.67	2.35
8.95	-8.29	8.52	3.14	9.79	27.16	2.92
7	-3.92	8.29	4.21	9.79	37.88	2.94
7.05	-3.9	8.28	4.21	9.79	37.94	2.95
12.56	-3.12	6.36	4.44	9.79	40.21	7.3
9.25	6.15	8.19	7.93	9.79	78.06	2.72
8.59	5.62	9.69	7.69	9.79	75.29	1.24
7.38	-3.73	8.17	4.26	9.8	38.4	3.28
9.81	-9.25	7.39	2.97	9.8	25.17	4.53
10.64	6.4	8.94	8.07	9.81	79.37	2.12
13.17	-1.9	9.28	4.83	9.82	43.89	1.56
8.35	5.5	9.62	7.65	9.82	74.51	1.42
10.02	-2.64	8.41	4.6	9.82	41.56	2.79
7.63	6.47	11.46	8.1	9.82	79.68	0.36
16.07	-2.48	8.22	4.67	9.83	42.05	2.79
9.72	6.18	10.44	7.97	9.83	78.04	0.81
10.18	-2.28	10.02	4.72	9.84	42.63	1.16

7.51	6.14	10.07	7.96	9.85	77.71	1.15
7.9	5.58	8.84	7.71	9.86	74.74	1.83
7.26	-9.53	10.05	3.15	9.87	23.94	1.35
11.48	-2.24	9.14	4.74	9.87	42.69	1.98
15.09	-8.36	6.75	3.2	9.87	26.85	6.8
10.38	6.23	8.84	8.02	9.87	78.14	2.19
9.5	-0.67	7.02	5.24	9.87	47.9	5.41
-1.78	-13.36	-0.81	-3.43	9.88	34.75	1.3
-0.76	-12.86	-0.12	-2.97	9.88	34.82	1.36
10.59	6.41	8.99	8.1	9.88	79.05	2.16
15.59	-10.46	6.95	2.8	9.88	22.75	6.56
9.76	6.08	8.88	7.95	9.88	77.24	2.21
10.53	6.16	8.42	7.99	9.88	77.69	2.88
18.57	-7.76	7.24	3.33	9.88	28.11	5.24
9.94	-4.01	8.04	4.25	9.88	37.4	3.85
10.44	-2.74	8.05	4.6	9.88	41.11	3.52
10.52	-2.9	8.12	4.56	9.88	40.62	3.29
12.31	-7.31	6.71	3.42	9.9	29.05	7.28
13.67	-6.66	6.46	3.58	9.9	30.56	8.11
7.26	-4.82	8.57	4.01	9.9	35.14	2.9
6.95	-3.99	8.36	4.25	9.91	37.37	2.94
8.07	-3.38	7.88	4.41	9.91	39.12	4.5
10.74	9.53	10.24	9.7	9.91	97.46	0.86
6.94	-4.04	8.45	4.24	9.92	37.22	3.07
13.61	-8.98	7.38	3.1	9.92	25.5	4.99
7.76	6.49	10.77	8.16	9.92	79.23	0.67
11.63	6.04	8.69	7.95	9.93	76.77	2.45
14.98	-5.12	6.89	3.98	9.94	34.28	6.68
12.12	-6.47	8.15	3.63	9.94	30.91	3.22
9.68	6.18	10.38	8.03	9.95	77.43	0.94
13.91	-6.59	6.72	3.62	9.96	30.6	8.11
15.27	-10.53	6.76	2.83	9.96	22.5	7.01
11.92	6.37	8.79	8.12	9.97	78.33	2.27
10.02	6.13	9.63	8.02	9.97	77.01	1.72
11.89	-2.08	9.06	4.85	9.98	42.86	1.95
10.74	6.13	8.82	8.03	9.99	76.95	2.47
11.45	6.14	8.91	8.03	9.99	76.98	2.32
7.9	6.51	10.47	8.21	9.99	78.96	0.93
12.59	9.99	10.78	9.99	9.99	100	0.65
16.5	-8.03	7.02	3.35	10	27.3	5.84
10.72	6.11	8.99	8.03	10	76.76	2.22
18.97	-6.25	7.33	3.75	10	31.32	5.72
7.56	6.54	11.37	8.22	10	79.11	0.4
13.13	-9.53	7.28	3.04	10.01	24.27	6.39

13.94	-8.32	7.37	3.28	10.01	26.67	4.54
19.26	-6.74	6.84	3.64	10.01	30.13	5.87
7.83	-3.86	8.28	4.34	10.02	37.48	3.33
12.52	-9.63	7.3	3.02	10.02	24.08	6.15
13.51	8.27	8.22	9.08	10.02	88.92	4.1
7.17	-4.97	8.82	4.03	10.02	34.48	2.4
15.36	-5.58	7.1	3.91	10.03	32.89	6.91
7.5	-3.69	8.31	4.4	10.03	37.91	3.38
7.64	-3.71	8.32	4.39	10.03	37.85	3.4
7.71	-3.8	8.33	4.37	10.03	37.6	3.37
7.91	-3.93	8.53	4.33	10.03	37.26	2.99
11.05	-2.17	9.78	4.85	10.04	42.41	1.33
15.25	0.29	7.7	5.68	10.05	50.73	4.07
8.04	-4.15	8.67	4.28	10.06	36.59	2.94
10.66	-2.19	10.23	4.87	10.07	42.26	1.09
7.53	6.4	10.29	8.19	10.07	77.96	1.02
12.78	-1.93	8.99	4.95	10.08	43.05	2.12
10.74	6.03	9.78	8.03	10.08	75.96	1.72
8.32	0.62	10.26	5.81	10.08	51.83	1.19
15.94	0.51	7.9	5.77	10.08	51.45	4.33
13.65	10.08	10.68	10.08	10.08	100	0.88
-2	-15.15	-3.48	-5.08	10.09	34.85	1.46
3.65	-17.26	0.24	-3.56	10.1	23.96	1.1
2.25	-14.35	2.4	-1.56	10.1	25.53	1.49
12.15	-2.76	6.79	4.7	10.1	40.44	10.15
15.26	-5.44	7.41	3.99	10.11	33.08	6.13
18.47	-7.7	7.54	3.47	10.11	27.82	5.32
9.7	-5.65	9.84	3.96	10.12	32.52	1.53
10.65	6.23	8.47	8.14	10.12	76.82	3.02
18.65	-7.64	7.29	3.49	10.12	27.91	5.23
7.59	-4.13	9.46	4.31	10.12	36.47	1.98
14.12	-6.34	6.76	3.78	10.13	30.84	7.98
13.22	-9.47	7.27	3.12	10.13	24.19	5.5
10.79	10.13	8.72	10.12	10.13	100	2.87
17.54	-3.32	9.25	4.6	10.14	38.69	1.59
7.19	-5.91	8.3	3.86	10.14	31.84	3.67
10.5	-4.16	8.03	4.35	10.14	36.35	4.54
7.95	-3.98	7.98	4.37	10.15	36.82	4.25
8.03	-3.67	7.81	4.46	10.15	37.68	4.62
9.16	-8.43	8.91	3.32	10.16	26.18	3.41
14.9	-5	7.34	4.13	10.17	34.06	5.93
10.07	-6.08	9.11	3.88	10.17	31.37	2.15
14.41	-6.51	6.97	3.77	10.19	30.31	8.05
7.15	-4.34	10.51	4.48	10.2	34.88	1.29

12.98	10.2	11.08	10.2	10.2	100	0.59
8.01	6.64	10.28	8.36	10.21	78.51	1.21
7.91	-9.92	10.17	2.81	10.22	23.97	1.43
10.77	6.18	9.14	8.16	10.22	76.02	2.35
13.87	-3.52	7.11	4.56	10.23	37.91	6.85
7.44	-5.81	8.78	3.93	10.23	31.88	3.15
7.42	-4.21	9.53	4.35	10.23	35.99	1.9
7.5	-4.14	9.44	4.37	10.24	36.18	1.93
17.89	-5.57	7.14	4.04	10.24	32.48	6.97
9.68	6.27	10.77	8.21	10.24	76.39	0.87
8.2	-4.28	8.93	4.35	10.25	35.75	3.11
7.36	-4.78	8.75	4.21	10.25	34.41	2.9
7.41	-4.64	8.63	4.24	10.25	34.78	3.42
8.14	-3.35	8.35	4.6	10.25	38.31	3.98
7.11	-5.66	8.57	3.98	10.26	32.2	3.51
11.71	6.27	9.29	8.22	10.27	76.23	1.88
7.12	-5.18	8.91	4.11	10.27	33.35	2.79
13.77	8.56	8.17	9.35	10.28	89.06	4.77
10.72	6.27	8.89	8.23	10.28	76.18	2.7
7.15	-5.16	8.98	4.12	10.28	33.39	2.85
11.69	6.08	10.16	8.13	10.28	75.16	1.26
11.08	-4.33	8.48	4.39	10.29	35.53	4.06
8.47	0.65	10.79	5.94	10.3	51.22	0.9
13.48	10.3	11.55	10.29	10.3	100	0.5
13	1.38	8.11	6.2	10.31	53.94	3.64
10.75	6.19	9.46	8.22	10.33	75.54	2.23
9.43	5.85	10.62	8.05	10.33	73.78	0.76
14.8	-3.82	7.02	4.54	10.34	36.78	7.89
10.7	6.24	9.24	8.24	10.34	75.74	2.52
-2.27	-19.49	-3.1	-5.59	10.35	24	1.34
11.62	-8.14	8.6	3.51	10.35	26.45	3.71
9.88	-6.22	9.64	3.95	10.36	30.64	1.68
10.44	6.22	8.79	8.24	10.36	75.55	2.98
7.06	-5.48	8.92	4.08	10.36	32.4	3.09
7.11	-5.64	9	4.05	10.36	32.02	2.98
7.43	-4.54	9.08	4.33	10.36	34.8	2.79
7.45	-4.31	9.04	4.39	10.36	35.42	2.77
7.12	-5.84	8.55	4	10.37	31.52	3.49
7.09	-5.73	8.53	4.03	10.37	31.77	3.49
7.87	-4.1	8.51	4.46	10.37	35.95	3.67
11.24	7.1	9.01	8.67	10.38	80.14	3.04
7.68	-4.29	9.53	4.41	10.38	35.42	2.24
7.93	-4.05	8.25	4.47	10.38	36.07	4.3
7.77	-4.38	9.22	4.39	10.39	35.14	2.56

16.64	0.99	8.35	6.12	10.42	52.04	4.01
10.35	5.78	10.26	8.08	10.42	72.98	1.34
7.77	-4.5	10.22	4.38	10.43	34.74	2.37
9.8	6.3	10.73	8.31	10.43	75.62	1.09
7.5	-4.25	10.26	4.45	10.44	35.37	1.42
10.25	6.25	10	8.29	10.44	75.31	1.79
10.78	6.17	9.72	8.26	10.44	74.86	2.12
10.45	-0.73	7.65	5.51	10.44	45.92	5.42
18.84	-6.03	7.98	4.05	10.45	30.9	4.9
8.69	-5.69	9.76	4.13	10.46	31.69	1.61
14.11	8.74	8.36	9.53	10.46	89.11	4.91
10.78	6.34	9.09	8.35	10.46	75.66	2.65
11.75	-4.7	8.44	4.38	10.46	34.16	4.71
11.38	6.79	8.96	8.56	10.47	77.96	2.78
7.07	-5.41	9.35	4.16	10.47	32.35	2.55
7.34	-6.1	8.72	4	10.48	30.69	3.29
8.22	-3.54	9	4.67	10.48	37.23	3.13
12.97	-6.41	8.9	3.96	10.51	29.9	2.8
16.61	-2.26	8.85	5.11	10.53	40.77	2.51
9.38	0.57	11.14	6.02	10.53	50.13	1.03
-11.93	-24.88	-13.17	-14.34	10.55	34.9	0.6
0.96	-11.54	1.64	-1.82	10.55	34.91	1.26
7.34	-4.23	10.73	4.52	10.55	35.16	1.07
17.76	-8.13	8.28	3.62	10.55	26.12	5.32
10.77	6.32	9.61	8.38	10.56	75.06	2.03
17.07	-5.48	8.14	4.24	10.56	31.98	5.75
12.23	-7.75	8.79	3.71	10.57	26.85	3.9
7.69	-5.49	8.92	4.19	10.57	31.95	2.99
16.56	-5.4	7.84	4.26	10.57	32.17	6.37
8.3	6.77	9.34	8.61	10.58	77.33	2.93
18.39	-7.68	8.47	3.73	10.58	26.98	4.01
8.62	-3.44	9.43	4.75	10.58	37.24	2.65
17.51	-5.52	7.53	4.24	10.58	31.86	6.47
8.82	-5.98	9.57	4.13	10.59	30.73	1.8
14.49	10.59	9.9	10.58	10.59	100	1.9
10.18	6.33	8.79	8.4	10.59	74.95	3.46
8.34	-3.74	9.24	4.67	10.59	36.41	3.04
15.92	-5.3	7.5	4.3	10.59	32.36	7.41
11.7	6.15	11.01	8.33	10.62	73.88	1.17
13.74	10.66	11.36	10.65	10.66	100	0.62
16.59	-3.59	7.93	4.77	10.67	36.6	7.21
12.65	6.53	10.02	8.53	10.67	75.62	1.7
10.35	6.35	9.78	8.45	10.67	74.66	2.24
10.41	6.33	9.72	8.45	10.67	74.55	2.49

17.46	-7.91	8.85	3.73	10.67	26.36	4.08
18.14	-8.04	8.23	3.7	10.67	26.1	4.6
14.23	-4.2	8.24	4.63	10.67	34.96	5.94
-2.04	-16.33	-4.52	-6.19	10.68	34.92	1.29
14.69	9	8.89	9.77	10.68	89.37	4.32
14.22	10.68	10.55	10.68	10.68	100	1.54
12.89	-8.65	8.68	3.58	10.68	24.87	4.56
12.42	-4.89	8.68	4.45	10.68	33.18	4.62
9.38	-8.49	9.4	3.6	10.7	25.16	2.91
9.53	-8.41	9.43	3.61	10.7	25.32	2.99
9.91	6.45	10.34	8.51	10.7	75.01	1.74
8.42	-3.8	9.13	4.71	10.7	35.96	3.35
8.47	-3.79	9.09	4.72	10.7	35.99	3.3
8.54	-3.55	9.49	4.78	10.7	36.64	2.67
16.58	-8.43	8.44	3.65	10.73	25.22	5.35
8.42	6.89	9.1	8.75	10.75	77.09	3.74
13.92	1.29	8.81	6.4	10.75	52.04	3.63
11.74	6.23	10.67	8.43	10.75	73.68	1.1
8.82	0.73	11.13	6.2	10.76	49.96	1.04
14.05	10.77	11.21	10.76	10.77	100	0.92
16.63	-3.67	7.81	4.81	10.78	36.12	7.32
16.09	-3.35	7.57	4.9	10.79	36.96	7.54
10.4	6.37	9.71	8.52	10.8	74.12	2.76
14.07	-8.3	9.01	3.71	10.8	25.36	4.17
13.65	-4.56	8.53	4.6	10.8	33.76	5.24
14.86	-4.93	8.05	4.51	10.8	32.82	6.31
15.4	-4.49	8.12	4.62	10.8	33.92	5.93
8.53	-4.44	9.27	4.6	10.81	34.04	3.32
15.5	9.29	8.17	9.97	10.81	90.33	6.36
8.51	6.99	9.54	8.81	10.81	77.27	3.1
8.88	-3.49	9.49	4.85	10.81	36.53	3.32
13.39	-6.73	14.35	5.47	10.82	24.01	0.79
17.09	-4.36	9.25	4.66	10.82	34.2	2.87
8.63	-4.6	9.43	4.57	10.82	33.61	3.12
17.1	-7.52	8.95	3.89	10.82	26.91	4.28
10.11	6.21	10.8	8.46	10.82	73.19	1.4
10.03	6.28	10.46	8.51	10.85	73.42	1.67
17.31	1.52	9.37	6.53	10.86	52.5	2.89
-16.42	-32.86	-21.38	-19.21	10.87	25.52	1.25
14.31	10.87	11.57	10.87	10.87	100	0.68
13.5	-8.47	9.19	3.73	10.9	24.86	3.72
9.9	6.42	10.15	8.6	10.91	73.84	2
9.95	6.37	10.17	8.58	10.91	73.58	1.97
11.41	-0.83	8.12	5.73	10.91	44.18	5.8

13.05	-5.02	9.09	4.54	10.91	32.37	4
10.31	6.47	9.67	8.62	10.92	74.01	2.9
11.73	6.31	11.98	8.54	10.92	73.22	0.64
9.58	-8.56	9.63	3.71	10.93	24.64	3.16
8.65	6.93	9.32	8.84	10.93	76.31	3.59
8.62	7.04	9.25	8.89	10.94	76.91	3.75
11.78	6.24	11.4	8.52	10.94	72.76	0.87
11.8	6.23	11.86	8.51	10.95	72.68	0.65
12.49	-2.83	7.93	5.12	10.96	38	10.07
10	6.52	10.37	8.66	10.96	74.08	2
8.57	7.05	9.35	8.91	10.96	76.8	3.62
14.66	-8.85	9.18	3.69	10.96	24.04	4.05
13.92	-6.36	9.15	4.21	10.97	29.1	3.3
16.08	-3.62	8.19	4.92	10.97	35.8	6.64
14.09	10.97	11.86	10.97	10.97	100	0.74
13.37	6.65	10.19	8.74	10.98	74.65	1.91
8.73	6.84	9.46	8.81	10.98	75.62	3.52
12.02	5.82	9.36	8.36	10.99	70.5	3.32
8.97	0.74	11.29	6.33	11	49.22	1.1
10.21	6.12	10.84	8.5	11.01	71.87	1.61
15.21	-8.75	9.59	3.74	11.01	24.14	3.45
15.69	-8.45	9.12	3.8	11.01	24.71	4.07
16.14	-7.9	9.05	3.92	11.01	25.79	4.02
13.96	11.01	11.51	11.01	11.01	100	0.88
8.02	-5.67	9.32	4.39	11.02	30.59	3.52
10	-8.68	9.96	3.74	11.03	24.24	2.67
15.07	9.75	8.94	10.32	11.03	91.82	5.18
10.03	6.57	9.79	8.73	11.04	73.95	2.92
9.96	6.49	9.64	8.69	11.04	73.58	3.07
17.97	1.35	9.66	6.58	11.08	51.15	2.87
-6.21	-23.1	-8.54	-9.54	11.1	25.51	1.2
-11.9	-24.9	-14.75	-14.37	11.1	34.95	0.99
9.65	0.67	11.59	6.35	11.1	48.64	0.97
14.79	1.02	9.52	6.48	11.11	49.84	3.58
17.42	-4.14	8.33	4.86	11.13	34.08	6.48
8.82	4.82	10.64	7.97	11.13	65.12	1.88
9.83	-8.48	9.86	3.84	11.14	24.44	3.01
10.19	-8.72	10.18	3.79	11.14	23.99	2.9
8.32	-5.96	9.48	4.39	11.15	29.66	3.65
9.17	-3.55	9.86	5.02	11.15	35.55	3.48
9.64	-8.57	9.95	3.83	11.16	24.25	3.23
8.99	-4.85	9.91	4.68	11.16	32.24	3.52
12.78	5.99	9.59	8.51	11.16	70.51	3.44
8.8	6.8	9.51	8.89	11.16	74.53	3.81

8.84	6.65	9.53	8.82	11.16	73.79	3.74
9.04	-3.48	9.75	5.04	11.16	35.74	3.26
11.84	6.42	12.41	8.71	11.16	72.62	0.45
11.64	6.37	12.19	8.69	11.18	72.28	0.71
20.84	-6.44	10.58	4.35	11.2	28.5	2.18
8.54	-4.81	9.85	4.71	11.21	32.21	3.08
10.32	6.23	10.88	8.66	11.24	71.27	1.7
8	-3.97	10.02	4.95	11.25	34.25	2.49
8.82	5.12	10.28	8.16	11.25	65.98	2.52
11.87	6.35	12.8	8.71	11.25	71.84	0.41
8.91	6.49	9.7	8.79	11.27	72.45	3.71
8.88	6.61	9.55	8.85	11.28	73.02	4.02
11.67	6.55	11.95	8.82	11.28	72.68	0.97
14.06	-5.67	11.22	4.54	11.29	30.02	1.67
19.05	1.56	10.13	6.78	11.31	51.1	2.79
8.94	-4.96	9.79	4.72	11.31	31.66	3.43
0.78	-19.19	-3.56	-5.88	11.35	25.5	1.16
13.75	-12.57	4.4	0.02	11.35	25.51	1.36
10.41	6.19	11.09	8.7	11.35	70.58	1.59
13.4	6.09	9.63	8.65	11.35	70.1	3.71
12.41	-1.13	8.45	5.85	11.35	41.99	6.56
8.87	4.8	10.72	8.07	11.36	64.05	2.11
8.9	4.7	10.68	8.04	11.37	63.56	2.28
12	6.47	12.75	8.83	11.37	71.84	0.53
8.92	6.15	9.93	8.68	11.38	70.25	3.46
8.84	5.53	9.89	8.41	11.38	67.3	3.41
8.97	6.37	9.96	8.8	11.39	71.25	3.39
14.62	11.39	11.68	11.39	11.39	100	0.76
17.71	-4.15	10.27	5.03	11.4	33.42	2.13
-1.82	-13.29	-0.92	-3.42	11.41	34.96	1.3
10.04	0.68	12.2	6.51	11.41	47.67	0.84
9.87	0.8	11.89	6.55	11.41	48.09	0.96
19.44	1.87	10.03	6.93	11.41	51.95	3.16
10.52	6.13	11.15	8.7	11.42	70	1.65
15.37	9.96	10.58	10.61	11.44	90.65	2.61
15	10.08	10	10.69	11.45	91.29	3.48
-6.65	-21.48	-5.48	-7.49	11.46	24.11	1.19
18.64	-4.26	8.78	5	11.46	33.06	7.68
10.64	6.06	11.14	8.69	11.46	69.44	1.77
10.31	-8.91	10.18	3.93	11.47	23.13	3.14
10.48	-9.19	10.49	3.87	11.47	22.63	3
8.98	4.67	10.63	8.07	11.47	63	2.38
9.08	4.53	10.49	8.01	11.47	62.39	2.77
12.08	6.47	12.68	8.88	11.48	71.33	0.5

9.28	-5.22	10.11	4.76	11.49	30.67	3.43
9.37	-3.63	9.87	5.17	11.49	34.56	4.45
9.78	-8.74	10.14	3.98	11.5	23.39	3.14
9.17	-4.94	9.89	4.83	11.5	31.3	3.88
21.08	-5.91	10.75	4.65	11.51	29.07	2.04
14	6.8	10.47	9.06	11.54	72.65	2.21
15.67	1.04	9.89	6.7	11.54	48.51	3.48
12.75	-1.62	9.28	5.8	11.56	39.93	8.53
-1.77	-19.21	-2.88	-5.4	11.57	24.17	1.29
9.15	4.47	10.58	8.04	11.59	61.66	2.9
9.44	-5.27	10.3	4.81	11.6	30.34	3.48
15.03	-5.27	10.81	4.81	11.61	30.31	2.43
8.56	-12.84	4.58	-0.19	11.62	25.45	1.43
14.41	-5.45	10.82	4.77	11.62	29.9	1.95
18.03	-5.97	10.74	4.68	11.62	28.73	2.21
20.39	1.76	10.67	7	11.62	50.82	3.06
10.8	-7.36	11.36	4.36	11.63	25.82	1.65
9.43	-3.62	10.46	5.24	11.63	34.3	3.47
15.79	10.16	10.97	10.81	11.64	90.6	2.59
10.75	6.09	11.36	8.8	11.68	68.57	1.84
9.24	4.4	10.59	8.07	11.7	60.93	2.97
9.63	-5.29	10.35	4.86	11.71	30.07	3.73
18.68	-3.76	9.08	5.27	11.71	33.74	6.47
12.08	6.33	12.81	8.92	11.71	69.55	0.52
14.83	-6.7	10.43	4.55	11.74	26.94	2.54
16.67	10.06	11.67	10.81	11.75	89.37	1.68
10.15	-1.56	12.16	5.94	11.77	39.56	1.27
18.24	-6.55	10.99	4.62	11.78	27.19	2.28
10.85	6.13	11.8	8.86	11.78	68.31	1.5
10.56	-9.38	11.38	4.01	11.79	21.83	2.17
11.03	-7.03	11.35	4.53	11.8	26.18	1.89
9.92	-1.55	11.04	5.96	11.8	39.51	2.46
13.3	-1.28	8.74	6.03	11.8	40.31	7.7
9.07	-9.61	11.47	4	11.81	21.42	1.82
12.04	6.51	13.34	9.05	11.81	70	0.73
7.14	-11.64	6.45	0.87	11.82	25.43	1.34
9.31	4.31	10.56	8.08	11.82	60.05	3.24
11.73	6.49	13.21	9.06	11.84	69.75	0.5
14.75	6.24	9.67	8.95	11.85	68.52	5.03
9.35	4.1	10.49	8.01	11.85	59.07	3.68
15.12	-5.07	10.94	4.99	11.86	30.27	2.24
10.23	0.79	12.53	6.77	11.87	46.6	0.93
14.66	6.79	10.96	9.22	11.87	71.08	2.46
-2.11	-18.77	-2.73	-5.46	11.88	25.4	1.15

10.04	-5.31	10.08	4.93	11.88	29.7	3.38
11.19	-6.78	11.57	4.64	11.9	26.51	1.95
-1.89	-19.27	-2.74	-5.46	11.91	24.19	1.22
9.81	-10.03	11.83	3.98	11.91	20.57	1.51
9.79	-5.27	10.61	4.97	11.91	29.74	3.43
9.97	-1.62	11.54	5.99	11.91	39.03	1.86
9.17	-3.54	11.77	5.42	11.92	33.83	1.85
9.29	-9.89	11.72	4	11.92	20.79	1.62
9.43	-9.89	11.71	4	11.92	20.79	1.64
19.35	-3.82	9.33	5.36	11.92	33.11	7.17
9.96	-1.26	10.63	6.11	11.93	40.02	3.35
11.81	6.42	13.22	9.07	11.93	69	0.49
9.36	4.02	10.53	8.02	11.94	58.38	3.73
11.93	6.27	12.83	9	11.94	68.28	0.64
10.04	-5.25	10.74	4.99	11.95	29.68	3.65
11.74	6.5	12.85	9.12	11.95	69.29	0.66
10	-1.13	10.64	6.18	11.98	40.27	3.62
16.51	0.99	10.51	6.91	11.99	46.92	3.4
7.38	-9.69	10.03	2.92	12.02	24.2	0.92
12.27	6.66	13.54	9.21	12.02	69.76	0.55
12.14	6.76	13.13	9.26	12.02	70.22	0.71
21.46	-5.51	11.52	5.02	12.03	28.96	1.76
10.05	-1.7	11.44	6.03	12.03	38.48	2.16
10.12	-1.68	11.44	6.03	12.03	38.55	1.97
-1.23	-17.33	-0.3	-4.15	12.06	25.38	0.95
9.37	3.89	10.49	8.03	12.07	57.38	4.05
20.76	1.85	10.68	7.25	12.07	49.64	2.91
15.28	-4.76	11.2	5.17	12.08	30.55	2.58
21.11	1.54	11.14	7.16	12.09	48.51	2.98
10.98	6.21	12.15	9.04	12.09	67.31	1.49
9.38	3.7	10.6	7.97	12.1	56.51	4.08
20.16	-4.18	11.15	5.4	12.12	31.81	1.64
11.45	-6.9	11.55	4.73	12.13	25.86	1.83
9.66	-3.46	11.45	5.55	12.14	33.54	2.69
23.5	-5.87	11.6	5	12.16	27.93	2.55
9.38	3.56	10.64	7.94	12.16	55.69	4.13
9.38	3.39	10.74	7.89	12.18	54.99	4.05
15.69	-7.07	10.89	4.71	12.2	25.42	2.96
21.44	1.72	11.07	7.27	12.2	48.76	3.08
10.73	-10.15	12.5	4.12	12.21	19.98	1.28
10.73	-9.51	11.65	4.23	12.24	20.97	2.46
12.25	6.64	13.47	9.31	12.25	68.59	0.49
15.32	-4.83	12.29	5.25	12.26	30.03	1.67
10.3	-5.12	11.14	5.19	12.27	29.37	3.67

9.58	1.74	11.01	7.29	12.27	48.6	3.54
18.67	-7.14	11.58	4.76	12.28	25.15	2.52
18.63	-6.63	11.91	4.87	12.28	26.16	1.59
10.06	-0.73	10.63	6.46	12.28	40.65	4.01
23.93	-7.22	11.42	4.76	12.29	24.99	2.39
9.42	3.24	10.84	7.88	12.29	54	4.17
12.05	6.84	13.07	9.43	12.29	69.37	0.71
10.47	0.9	12.89	7.03	12.32	45.61	1.01
18.69	-8.34	11.36	4.53	12.33	22.86	2.75
15.48	6.41	9.88	9.25	12.34	67.14	5.44
10.05	-8.92	10.2	3.2	12.36	25.32	1.21
-1.27	-17.46	-0.16	-4.22	12.37	25.25	0.81
24.34	-7.02	12.02	4.85	12.38	25.22	2.19
9.47	3.03	10.87	7.84	12.38	52.89	4.43
9.47	2.49	10.92	7.63	12.38	50.89	3.99
9.66	1.48	11.06	7.26	12.38	47.38	3.62
9.75	1.19	11.01	7.16	12.38	46.41	3.83
10.05	0.59	10.85	6.95	12.38	44.43	4.19
10.12	-0.01	10.74	6.75	12.38	42.54	4.39
9.49	2.39	10.87	7.6	12.39	50.54	3.89
12.66	-6.59	13.81	5.19	12.41	25.23	0.85
10.77	0.87	13.24	7.08	12.42	45.23	0.74
11.11	0.92	13.39	7.09	12.42	45.38	0.74
11.94	6.85	12.49	9.49	12.42	68.85	1.33
15.35	6.96	11.4	9.55	12.43	69.31	2.47
9.86	1.05	11.01	7.13	12.43	45.78	3.89
11.24	-10.21	12.81	4.23	12.44	19.59	1.3
12.01	-6.86	12.53	4.91	12.44	25.42	1.59
16.59	10.57	12.82	11.39	12.44	88.39	0.97
10.34	-3.42	11.97	5.72	12.45	32.95	2.24
19.73	-3.54	9.82	5.71	12.47	32.64	6.24
12.32	6.73	13.71	9.45	12.47	68.06	0.55
10.45	-1.96	13.63	6.18	12.48	36.65	0.62
19.97	-4.73	9.94	5.4	12.48	29.83	7.37
9.83	-3.56	11.21	5.69	12.48	32.55	4.21
9.47	2.71	10.91	7.77	12.49	51.33	4.27
9.95	0.9	11.03	7.11	12.49	45.12	3.99
12.38	6.83	14.08	9.51	12.49	68.44	0.44
17.55	10.88	12.05	11.58	12.49	89.87	1.38
16.48	-7.1	11.31	4.86	12.51	24.85	3.03
17.34	1.19	11.45	7.23	12.51	46	2.69
12.06	6.76	13.12	9.5	12.52	67.98	0.93
11.99	6.89	13.14	9.55	12.52	68.56	0.87
18.83	-8.08	12.08	4.69	12.53	23	2.16

13.07	3.6	10.08	8.12	12.53	54.52	4.73
9.48	2.88	10.85	7.85	12.53	51.82	4.51
11.96	6.77	12.65	9.5	12.53	67.96	1.31
10.83	-9.55	12.11	4.38	12.55	20.49	2.25
11.17	-9.28	12.43	4.43	12.55	20.93	1.94
11.27	6.26	12.01	9.29	12.57	65.43	2.45
7.78	-14.18	3.23	-1.29	12.58	25.22	1.45
15.05	-4.57	12.71	5.49	12.59	29.97	1.54
10.64	-5.02	11.24	5.39	12.59	28.96	4.6
13.53	3.24	13.82	8.01	12.6	52.93	0.95
12.38	6.61	13.84	9.46	12.6	66.88	0.51
21.7	1.67	11.75	7.46	12.62	47.26	2.64
12.19	6.52	13.74	9.43	12.62	66.39	0.56
10.99	-5.12	11.13	5.37	12.64	28.64	3.26
10.69	-2.1	13.36	6.22	12.65	35.86	0.93
10.86	-3.49	12.57	5.8	12.66	32.35	2.06
24.56	-7.67	11.6	4.86	12.67	23.53	2.75
23.09	-5.37	12.61	5.39	12.68	28.05	1.91
15.05	-1.66	9.76	6.35	12.68	36.99	8.47
12.28	6.8	14.27	9.59	12.68	67.42	0.4
11.09	-9.48	12.15	4.47	12.69	20.4	2.23
12.16	6.84	14.59	9.61	12.69	67.56	0
11.81	-10.22	12.65	4.37	12.71	19.23	1.81
12.98	2.62	11.09	7.83	12.71	50.29	4.51
18.07	1.03	11.7	7.28	12.72	44.85	2.8
18.69	-9.97	11.83	4.43	12.73	19.58	3.39
18.33	-9.64	11.94	4.49	12.74	20.09	2.99
18.21	-9.59	11.48	4.51	12.75	20.15	3.68
25.45	-7.24	11.66	4.99	12.75	24.2	3.08
-1.39	-16.95	0.77	-3.72	12.77	25.16	0.74
11.4	6.29	11.97	9.41	12.79	64.64	2.89
16.28	6.44	11.06	9.47	12.79	65.3	4.19
10.96	-9.53	12.22	4.52	12.8	20.19	2.19
12.59	6.87	13.97	9.67	12.8	67.27	0.67
-1.5	-17.52	0.03	-4.24	12.85	25.16	0.75
17.88	-9.51	12.09	4.57	12.85	20.16	2.96
18.93	-9.23	11.93	4.63	12.85	20.61	2.75
11.83	0.88	14.07	7.28	12.85	44.02	0.69
16.08	7.04	12.1	9.78	12.85	67.8	2.45
11.44	1.03	13.33	7.35	12.86	44.47	1.08
3.2	-19.41	-3.79	-5.96	12.87	25.16	1.14
25.3	-8.1	12.39	4.88	12.87	22.46	2.38
10.91	-2.18	13.39	6.32	12.88	35.14	1.29
11.52	6.24	12.43	9.44	12.9	64	2.54

10.91	-4.93	11.43	5.58	12.93	28.52	4.98
10.01	-3.39	11.59	5.98	12.94	32	4.42
19.05	-9.24	12.14	4.68	12.95	20.46	2.41
22.03	1.47	12.11	7.55	12.95	45.62	2.63
12.77	6.9	13.56	9.75	12.95	66.7	0.99
12.82	6.96	13.68	9.78	12.95	66.99	0.92
14.76	-4.3	12.24	5.74	12.96	29.85	2.47
25	-8.1	12.13	4.92	12.96	22.34	2.66
-5.48	-23.3	-7.76	-9.22	12.98	24.21	1.11
12.39	-10.22	12.96	4.52	13	18.87	1.76
12.26	6.01	12.44	9.39	13.01	62.51	2.38
15.85	-2.14	10.13	6.38	13.01	34.93	9.41
11.01	-3.45	12.25	6	13.02	31.69	2.95
14.59	-4.29	12.04	5.78	13.02	29.76	3
12.38	5.87	12.88	9.32	13.02	61.87	2.08
12.91	7.05	13.61	9.87	13.04	67.01	1.05
12.72	6.95	14	9.83	13.05	66.49	0.73
25.73	-7.45	12.86	5.11	13.06	23.34	1.91
18.07	-9.35	12.1	4.73	13.08	20.11	2.77
17.03	6.5	11.35	9.64	13.08	64.35	4.49
15.97	-9.24	12.21	4.77	13.12	20.21	3.09
20.38	-4.23	11.11	5.86	13.12	29.71	5.98
11.25	-3.59	12.25	6.02	13.13	31.14	3.32
11.18	-4.73	11.43	5.74	13.16	28.54	5.2
18.83	0.6	11.98	7.35	13.16	42.25	3.06
13.23	6.88	13.59	9.85	13.17	65.68	1.3
12.27	0.97	14.57	7.48	13.18	43.36	0.56
17.27	-7.01	11.79	5.23	13.18	23.95	2.94
15.4	-9.51	12.46	4.76	13.19	19.71	2.58
17.48	-9.21	12.22	4.81	13.19	20.19	2.89
10.09	-3.56	11.87	6.06	13.19	31.08	4.38
-2.56	-19.51	-3.28	-5.7	13.2	24.26	1.3
13.99	-4.21	12.34	5.89	13.2	29.59	2.58
12.94	-10.24	12.8	4.63	13.2	18.6	2.28
17.73	6.5	11.55	9.69	13.2	63.83	4.61
11.65	6.35	12.58	9.63	13.23	63.05	2.67
14.63	-10.25	12.94	4.64	13.24	18.53	2.4
25.69	-7.67	12.83	5.15	13.24	22.68	2.08
11.97	6.11	12.45	9.53	13.24	62	3.02
12.17	6.07	12.26	9.52	13.24	61.8	2.91
11.52	-4.68	11.81	5.81	13.26	28.45	5.05
11.19	-2.25	12.98	6.49	13.26	34.08	1.99
16.64	-9.4	12.82	4.82	13.27	19.78	2.56
17.11	-10.02	12.61	4.71	13.28	18.82	2.7

16.95	7.23	12.5	10.06	13.28	66.77	2.54
11.41	-2.4	13.13	6.44	13.28	33.68	1.81
17.48	-9.44	12.22	4.82	13.29	19.68	2.91
20.14	0.16	11.75	7.26	13.29	40.58	4.9
12.01	-5.56	11.86	5.6	13.3	26.55	3.53
25.44	-7.16	11.92	5.3	13.31	23.48	2.78
11.75	6.28	12.67	9.65	13.34	62.34	2.83
12.54	5.93	13.08	9.5	13.34	60.85	2
11.91	6.2	12.43	9.62	13.35	61.94	3.06
12.07	6.11	12.5	9.59	13.37	61.47	3.13
13.6	-10.03	12.99	4.77	13.39	18.67	2.17
18.03	-7.22	12.05	5.3	13.39	23.24	3.41
22.32	1.26	12.85	7.69	13.39	43.64	2.32
13.28	-3.94	12.68	6.08	13.43	29.74	2.51
12.67	5.98	13.13	9.56	13.43	60.67	2.05
12.82	5.96	13.27	9.55	13.44	60.58	1.96
11.82	-3.71	12.59	6.14	13.45	30.22	3.29
11.83	6.29	12.77	9.71	13.45	61.93	2.74
11.7	-2.43	13.02	6.52	13.45	33.23	2.55
10.77	-0.71	12.67	7.01	13.45	37.71	3.46
10.21	-3.71	12.36	6.15	13.46	30.19	4.23
-1.97	-19.25	-2.41	-5.47	13.47	24.26	1.06
13.4	3.18	14.03	8.4	13.47	49.76	1.13
-0.84	-17.18	0.72	-3.6	13.48	24.27	0.88
25.76	-7.49	13.17	5.31	13.48	22.64	2.12
10.63	-1.19	12.4	6.87	13.48	36.34	3.92
10.68	-0.96	12.33	6.94	13.48	36.93	3.85
19.55	0.5	12.04	7.46	13.49	41.07	4.07
13.01	6.97	14.24	10.03	13.49	64.7	0.97
15.08	-9.09	12.97	5	13.5	19.96	2.09
17.45	7.37	12.72	10.22	13.5	66.44	3.06
17.18	7.27	12.78	10.17	13.5	66.01	2.69
13.51	2.94	13.83	8.33	13.51	48.83	0.75
13.76	3.07	15.05	8.37	13.51	49.28	0.53
10.66	-0.81	12.34	7	13.51	37.28	3.72
18.31	6.24	11.85	9.72	13.52	61.42	5.5
11.45	-9.3	13.93	4.95	13.54	19.58	1.37
12.07	-3.84	12.63	6.16	13.55	29.72	3.46
20.47	-4.55	11.48	6	13.55	28.2	6.08
10.37	-3.97	12.96	6.13	13.56	29.44	3.34
11.88	-4.72	12.23	5.96	13.58	27.79	4.64
18.75	-7.48	12.82	5.34	13.59	22.49	2.99
13.37	6.94	14.34	10.06	13.59	64.17	1.03
13	-5.17	12.33	5.86	13.6	26.8	3.81

10.64	-1.17	12.37	6.94	13.61	36.1	4.4
18.21	7.5	12.6	10.34	13.62	66.55	3.61
13.01	6.01	13.57	9.67	13.65	59.95	2.1
10.95	-0.64	12.45	7.13	13.66	37.39	3.85
17.45	-2.11	10.93	6.71	13.66	33.56	9.55
12.31	-4.68	12.6	6.02	13.67	27.7	4.32
10.8	-0.81	12.4	7.08	13.67	36.89	4.06
10.7	-1.21	12.7	6.95	13.67	35.83	3.59
25.78	-7.11	13.73	5.51	13.7	22.97	1.63
22.17	1.26	13.34	7.84	13.71	42.76	2.19
13.57	7.07	15.69	10.17	13.71	64.2	0.31
19.11	6.25	12.2	9.82	13.72	60.7	5.29
-2.19	-19.22	-3.19	-5.45	13.73	24.29	1.4
17.77	7.47	12.78	10.38	13.73	65.94	2.97
10.63	-1.39	12.74	6.94	13.74	35.21	3.27
11.07	-0.51	12.82	7.21	13.75	37.53	3.51
11.68	-9.37	13.73	5.05	13.76	19.21	1.93
13.68	7.06	15.67	10.2	13.77	63.95	0
12	-2.48	13.25	6.68	13.79	32.38	2.97
22.23	1.21	13.37	7.86	13.79	42.37	2.12
10.95	-0.67	12.86	7.18	13.79	37	3.86
13.49	7.05	14.6	10.21	13.79	63.83	0.74
10.25	-3.97	12.72	6.26	13.82	28.93	3.65
18.2	7.63	12.77	10.49	13.83	66.2	3.35
25.51	-6.91	13.27	5.62	13.84	23.12	2.53
18.23	7.61	13.09	10.49	13.84	66.08	2.79
12.08	-9.43	13.97	5.09	13.86	18.99	1.97
11.95	-9.53	13.77	5.08	13.87	18.83	1.91
11.81	-9.41	13.44	5.1	13.88	18.99	2.36
20.46	-4.69	11.82	6.12	13.88	27.31	6.07
12.73	-3.97	13.3	6.3	13.89	28.8	2.53
11.06	-0.62	12.54	7.25	13.9	36.86	4.85
13.97	-4.9	12.65	6.08	13.92	26.8	3.96
13.26	1.1	15.18	7.89	13.94	41.66	0.7
19.75	6.37	12.06	9.97	13.95	60.25	6.38
18.21	-2.46	11.53	6.77	13.98	32.02	8.78
13.8	6.94	15.65	10.24	13.99	62.51	0.53
10.5	-3.55	13.03	6.46	14	29.52	4.03
7.6	-9.84	9.59	2.76	14.04	24.3	1.14
12.63	-9.47	14.62	5.18	14.05	18.69	1.38
20.34	6.11	12.31	9.9	14.06	58.77	7.06
12.28	-2.5	13.42	6.81	14.06	31.78	3.08
20.26	-5.5	12.28	6.03	14.08	25.34	6.74
11.34	-0.33	12.72	7.43	14.08	37.23	4.14

20.46	-5.58	12.03	6.01	14.09	25.19	6.93
13.22	6.02	13.76	9.88	14.09	58.3	2.78
10.68	-4.02	13.86	6.39	14.09	28.32	2.65
18.89	-2.6	11.71	6.78	14.09	31.5	8.93
10.59	-3.88	13.54	6.43	14.1	28.6	2.77
22.16	1.22	14.6	8.01	14.11	41.56	1.38
12.82	-4.79	12.9	6.21	14.11	26.69	4.57
12.47	-9.51	14.25	5.21	14.12	18.56	1.64
25.13	-7.28	13.74	5.69	14.14	22.05	2.63
19.1	7.78	13.63	10.7	14.17	65.46	2.73
20.7	-0.75	11.99	7.4	14.19	35.83	6.04
13.81	1.11	14.71	8.02	14.2	40.98	1.25
19.54	-2.16	11.79	6.96	14.2	32.3	9.25
12.24	-9.53	13.95	5.25	14.21	18.41	2.09
12.55	-2.55	13.78	6.86	14.21	31.35	2.88
21.26	1.18	14.23	8.05	14.21	41.17	2.1
21.59	1.42	14.12	8.13	14.21	41.88	2.14
11.16	-0.43	12.99	7.46	14.21	36.62	4.65
-2.17	-19.18	-3.09	-5.45	14.22	24.38	1.34
20.16	-5.4	12.46	6.13	14.23	25.29	6.27
20.91	1.26	13.96	8.08	14.23	41.33	2.19
13.02	-6.75	14.33	5.33	14.24	24.4	0.69
24.84	-6.83	14.03	5.84	14.24	22.67	1.96
13.31	-4.61	12.81	6.33	14.26	26.8	5.34
20.97	6.23	12.36	10.05	14.28	58.43	6.79
21.29	-0.65	13.05	7.49	14.3	35.85	4.95
20.15	-1.53	12.14	7.19	14.3	33.61	9.05
14.28	7.08	15.43	10.43	14.3	61.87	0.61
14.49	6.9	16.14	10.37	14.31	61.08	0.52
13.85	-4.31	12.87	6.44	14.32	27.32	5.85
11.56	-0.49	13.22	7.5	14.33	36.19	4.39
10.58	-4.06	13.12	6.5	14.33	27.8	3.91
11.39	-0.27	12.78	7.58	14.34	36.76	5.6
19.54	7.83	13.93	10.82	14.36	64.86	2.72
16.16	1	14.42	8.06	14.36	40.23	1.9
12.94	-9.37	14.95	5.37	14.37	18.46	1.33
14.95	1.1	14.8	8.09	14.37	40.51	1.33
20	-6.41	12.74	5.98	14.4	23.16	5.97
21.86	-0.66	12.84	7.53	14.4	35.57	5.23
11.6	-0.16	12.8	7.64	14.4	36.89	5.62
10.85	-3.86	13.87	6.59	14.42	28.06	3.12
20.06	1.2	14.34	8.16	14.43	40.64	2.33
15.56	1.05	14.28	8.11	14.44	40.18	1.96
13.9	7.11	15.84	10.51	14.44	61.43	0.51

-0.2	-16.85	1.04	-3.38	14.45	24.44	0.84
20.17	-7.58	13.91	5.76	14.45	21.12	2.6
13.57	6.24	14.41	10.13	14.45	57.84	2.22
14.04	7.11	15.41	10.52	14.45	61.4	0.74
16.79	0.89	14.8	8.07	14.46	39.65	1.75
19.45	-5.62	12.97	6.21	14.5	24.45	6.44
21.9	1.75	14.88	8.4	14.52	42.02	1.18
18.69	-5.01	12.96	6.37	14.52	25.58	6.84
20.44	7.88	13.88	10.92	14.52	64.4	3.04
12.88	-2.49	14.02	7.04	14.53	30.85	3.05
11.68	-0.68	13.37	7.54	14.54	35.21	4.65
11.02	-3.95	13.68	6.63	14.54	27.66	3.7
19.58	1.06	14.23	8.17	14.55	39.91	2.25
-6.35	-21.18	-5.37	-7.35	14.56	24.49	1.09
13.41	6.23	13.96	10.18	14.56	57.42	2.69
11.31	-0.52	12.95	7.6	14.56	35.59	5.38
13.13	-9.31	15.56	5.48	14.57	18.31	0.88
22.23	6.18	13.2	10.17	14.58	57.12	5.41
21.69	6.37	13.15	10.26	14.59	57.84	5.29
11.42	-0.35	13.23	7.67	14.59	35.94	5.15
19.82	-6.15	12.87	6.14	14.61	23.31	6.24
13.77	6.24	14.94	10.2	14.61	57.25	1.64
20.79	-1.37	12.62	7.4	14.61	33.33	7.53
-5.55	-23.29	-7.83	-9.33	14.62	24.52	1.11
22.65	-6.15	14.98	6.19	14.63	23.28	1.86
14.44	-4.5	13.27	6.55	14.63	26.39	5.47
15.5	-4.52	13.36	6.55	14.65	26.33	6.04
19.09	1.08	14.72	8.22	14.65	39.71	1.74
11.6	-0.42	13.18	7.68	14.65	35.64	6.19
11.97	-0.77	13.32	7.57	14.65	34.75	5.41
-0.54	-16.87	0.92	-3.46	14.66	24.62	0.86
15.01	-5.23	13.08	6.39	14.69	24.89	4.75
20.8	-7.62	14.26	5.88	14.7	20.72	2.2
11.18	-3.93	13.93	6.71	14.7	27.42	4.29
16.49	-4.35	13.25	6.63	14.72	26.52	6.12
17.86	-4.68	13.23	6.54	14.72	25.88	6.34
19.08	-5.3	12.86	6.4	14.72	24.7	6.99
17.4	0.89	14.85	8.2	14.72	39	1.72
14.97	-4.9	13.21	6.5	14.73	25.44	6.07
18.26	-4.32	12.9	6.64	14.73	26.59	6.59
17.43	-4.86	13.07	6.51	14.74	25.5	6.7
13.2	-2.39	14.26	7.18	14.75	30.65	3.27
12.15	-0.59	13.5	7.67	14.75	34.99	5.94
13.32	-9.55	15.86	5.53	14.76	17.75	0.86

20.57	1.24	15.02	8.33	14.76	39.88	1.23
11.65	-0.45	13.17	7.72	14.76	35.3	6.21
11.89	-0.87	13.44	7.58	14.76	34.26	5.23
11.24	-4.1	13.74	6.7	14.76	26.94	4.15
21.3	7.97	14.29	11.06	14.77	63.75	2.56
22.79	6.73	13.58	10.51	14.8	58.5	4.84
13.96	6.27	15.05	10.31	14.82	56.58	1.66
16.98	-4.59	13.18	6.62	14.83	25.86	6.54
-6.29	-23.39	-8.5	-9.51	14.84	24.71	1.19
11.33	-3.93	14.13	6.79	14.85	27.15	3.7
-1.12	-17.1	0.84	-3.69	14.86	24.71	0.74
14.73	7.11	16.45	10.7	14.86	59.78	0.45
-1.51	-17.56	-0.33	-4.14	14.87	24.78	0.89
21.4	-8.08	14.32	5.87	14.87	19.77	2.65
22.25	7.84	14.36	11.05	14.87	62.81	3.12
18.51	0.73	14.86	8.21	14.87	38.18	1.74
16.01	-4.55	13.31	6.66	14.88	25.87	5.99
11.52	-3.96	14.25	6.81	14.91	26.99	3.65
-1.28	-17.03	0.27	-3.68	14.93	24.83	0.96
14.18	6.19	15.58	10.34	14.96	55.77	1.37
11.43	-4.05	14.2	6.81	14.96	26.71	3.47
12.67	0.01	13.64	7.97	14.97	35.99	5.12
11.26	-4.06	13.84	6.81	14.97	26.68	4.53
14.96	7.11	16.9	10.74	14.97	59.36	0.31
15.2	7.05	16.27	10.73	14.98	59.09	0.69
13.57	-9.33	15.87	5.7	15.01	17.77	1.01
23.25	6.55	13.87	10.53	15.01	57	5
22.3	-1.91	13.24	7.46	15.02	31.19	6.45
11.7	-4.01	14.52	6.86	15.06	26.62	3.84
-15.61	-32.66	-19.31	-18.74	15.08	24.85	0.87
21.38	-1.84	12.84	7.49	15.1	31.19	9.03
13.55	-1.98	14.7	7.48	15.12	30.85	3.29
24.02	6.5	13.58	10.55	15.13	56.34	6.51
15.44	7.1	16.2	10.82	15.13	58.71	0.77
15.72	7.12	15.55	10.82	15.13	58.83	1.42
24.43	6.51	13.88	10.57	15.15	56.31	5.8
-1.39	-17.46	0.28	-4.1	15.16	24.93	0.73
15.99	7.15	15.45	10.85	15.17	58.81	1.78
22.89	-9.14	15.09	5.83	15.18	17.84	2.84
22.98	7.87	14.88	11.2	15.19	61.67	2.82
22.96	8.02	14.65	11.27	15.19	62.29	3
10.55	-8.9	10.23	3.34	15.22	24.94	1.38
12.37	-0.94	14.01	7.79	15.22	33.08	6.54
13.97	-9.49	15.93	5.79	15.23	17.3	1.62

14.44	6.22	15.88	10.47	15.23	54.92	1.42
22.38	-1.95	13.29	7.54	15.26	30.64	9.39
11.85	-4.52	14.85	6.84	15.27	25.29	3.36
-1.27	-17.33	-0.69	-3.99	15.28	24.95	1.16
21.93	-1.32	13.18	7.73	15.28	32.04	9.4
22.75	7.95	15.09	11.27	15.29	61.57	2.55
24.73	6.52	13.79	10.63	15.29	55.83	6.28
14.71	3.19	17.13	9.25	15.3	44.26	0.58
22.5	7.9	14.6	11.26	15.32	61.25	3.52
11.8	-4.64	14.67	6.85	15.32	24.98	3.46
13.89	0.19	14.13	8.2	15.33	35.66	4.54
16.28	7.17	15.82	10.96	15.37	58.13	1.57
17.62	7.22	14.72	10.97	15.37	58.31	3.66
13.94	-1.69	15.11	7.68	15.37	31	3.13
23.55	-8.94	15.25	5.97	15.39	17.89	2.52
11.97	-4.56	15.15	6.89	15.39	25.01	2.77
16	-5.23	13.83	6.75	15.42	23.73	5.76
21.9	7.69	15.19	11.22	15.43	59.98	2.85
22.27	7.8	14.86	11.27	15.43	60.45	3.39
21.42	7.62	14.65	11.19	15.44	59.63	3.72
23.18	-4.35	14.14	7.03	15.47	25.28	6.76
20.81	7.58	14.94	11.18	15.48	59.31	3.52
11.77	-4.37	14.89	6.99	15.49	25.22	3.56
23.86	-8.86	15.05	6.04	15.5	17.86	3.15
22.45	-8.29	14.96	6.16	15.51	18.66	2.21
12.47	-1.07	14.2	7.89	15.51	32.16	6.77
20.36	7.61	15.13	11.23	15.54	59.23	3.06
20.56	7.6	15.24	11.22	15.54	59.2	2.88
25.13	6.49	14.35	10.72	15.54	54.83	5.13
18.97	7.25	14.91	11.07	15.55	57.75	3.8
14.7	6.29	16.12	10.64	15.55	54.08	1.42
17.34	7.32	15.24	11.1	15.56	57.99	2.55
18.31	7.19	14.94	11.05	15.56	57.49	3.56
18.09	7.22	14.8	11.05	15.57	57.55	3.84
12.2	-4.63	15.34	6.97	15.58	24.58	2.89
21.07	7.61	15.32	11.27	15.63	58.87	2.94
16.57	7.28	16.36	11.11	15.63	57.59	1.21
19.28	7.41	15.37	11.18	15.65	58.04	2.97
19.55	7.2	15.22	11.08	15.65	57.18	3.38
17.06	7.32	15.94	11.15	15.67	57.57	1.7
14.95	6.27	16.14	10.68	15.68	53.54	1.63
23.27	-1.45	13.73	7.88	15.68	30.94	9.31
16.84	7.33	16.24	11.16	15.69	57.54	1.33
23.17	-8.88	15.27	6.15	15.71	17.59	2.72

20.16	7.51	15.24	11.25	15.72	58.14	3.22
14.89	0.1	14.66	8.36	15.73	34.54	4.41
25.47	6.52	14.68	10.83	15.74	54.27	4.86
12.51	-4.44	15.1	7.1	15.75	24.66	3.78
19.87	7.04	15.1	11.07	15.76	56.19	3.91
22.87	-2.79	14.52	7.57	15.76	27.88	5.04
14.26	-9.55	15.99	6.05	15.77	16.64	2.04
17	-6.03	14.36	6.74	15.77	21.83	6.15
23.44	-3.83	13.95	7.31	15.78	25.76	7.48
14.41	-1.67	15.42	7.89	15.79	30.22	3.42
12.83	-4.3	15.02	7.15	15.79	24.86	4.65
22.89	-1.62	13.76	7.89	15.79	30.34	8.81
12.4	-4.47	15.22	7.11	15.8	24.54	3.64
12.68	-4.36	14.98	7.14	15.8	24.73	4.39
23.97	-8.54	15.33	6.26	15.81	17.95	2.78
12.61	-1.19	14.5	8	15.81	31.27	6.16
12.73	-1.21	14.52	7.98	15.81	31.24	6.77
23.78	-4.68	15.02	7.13	15.85	24.06	5.8
15.2	3.61	17.25	9.66	15.86	44	0.6
15.23	6.24	16.47	10.76	15.86	52.82	1.41
14.55	-9.58	16.1	6.1	15.88	16.48	2.03
25.62	6.36	14.77	10.82	15.9	53.13	5.52
24.23	-8.97	15.85	6.23	15.91	17.25	2.6
16.21	6.14	16.5	10.76	15.97	52.08	1.59
15.83	6.14	16.71	10.76	15.97	52.11	1.2
15.52	6.27	16.65	10.81	15.97	52.59	1.25
14.89	-1.81	15.52	7.95	16	29.5	3.71
23.65	-1.54	14.09	8.01	16	30.11	9.32
12.81	-1.48	14.74	8.01	16.02	30.21	6.87
12.83	-1.07	15.01	8.12	16.02	31.14	5.98
14.89	-9.71	16.73	6.17	16.07	16.11	1.36
14.48	2.55	16.95	9.37	16.07	40.28	1.19
14.67	2.91	16.8	9.5	16.07	41.31	0.8
15.09	3.53	16.84	9.73	16.07	43.14	1.04
15.48	-1.51	15.76	8.08	16.1	29.99	3.69
14.93	3.21	17.39	9.62	16.11	42.09	0.72
13.16	-4.47	15.48	7.26	16.11	24.05	4.49
13.05	-4.43	15.27	7.28	16.12	24.12	4.95
12.91	-2.06	15.04	7.89	16.13	28.75	5.76
24.53	-8.53	16.11	6.44	16.18	17.56	2.52
16.67	6.13	16.72	10.84	16.18	51.39	1.77
15.36	3.84	17.52	9.91	16.22	43.69	0.81
13.13	-1.48	15.28	8.1	16.22	29.81	6.84
14.56	2.49	17.33	9.42	16.24	39.67	0.57

15.19	-9.7	17.2	6.27	16.26	15.93	0.98
14.98	3.58	17.14	9.82	16.26	42.79	0.95
15.59	-9.69	17.37	6.27	16.27	15.94	0.93
14.85	3.11	17.14	9.66	16.28	41.33	0.99
25.81	6.17	15.3	10.91	16.28	51.2	6.06
24.28	-4.53	15.42	7.41	16.35	23.59	6.05
13.01	-2.18	14.92	7.96	16.35	28.09	6.53
15.87	0.21	15.19	8.69	16.37	33.41	5.06
25.78	6.35	15.12	11.03	16.39	51.46	5.26
18.45	-5.53	14.98	7.16	16.39	21.82	6.76
16.08	-1.29	16.16	8.29	16.4	29.9	3.45
24.01	-4.5	14.84	7.44	16.4	23.56	6.28
13.41	-3.89	16.16	7.57	16.42	24.63	3.95
23.99	-1.93	14.54	8.1	16.42	28.49	8.85
24.52	-8.82	16.8	6.52	16.45	16.87	2.32
13.23	-2.69	15.37	7.87	16.46	26.85	6.79
16.21	-9.54	17.36	6.4	16.47	15.92	1.15
24.27	-8.64	16.18	6.57	16.49	17.07	2.07
17.18	6.19	16.8	11.01	16.49	50.57	2.08
24.52	-9.09	16.82	6.51	16.52	16.43	1.9
24.4	-9.39	16.71	6.45	16.53	16.05	2.51
13.29	-2.79	15.47	7.88	16.53	26.54	6.93
23.85	-9.32	16.19	6.48	16.56	16.11	3.71
15.77	3.74	17.41	10.03	16.59	42.37	1.16
25.98	6.22	15.34	11.06	16.59	50.34	6.29
25.9	6.34	15.54	11.12	16.59	50.8	5.34
16.73	-1.5	16.43	8.32	16.59	29.08	3.53
24.12	-9.49	16.94	6.48	16.62	15.83	2.36
24.24	-1.65	14.77	8.27	16.62	28.7	9.47
24.22	-8.81	16.49	6.62	16.65	16.66	3
13.34	-2.92	15.66	7.9	16.65	26.1	6.59
15.92	3.55	17.84	10	16.68	41.58	1.12
17.75	6.13	16.97	11.07	16.68	49.75	2.33
25.94	6.24	15.85	11.11	16.68	50.14	5.09
15.62	3.91	17.46	10.14	16.69	42.61	1.21
23.33	-9.72	16.89	6.51	16.75	15.42	2.64
16.75	-9.4	17.81	6.57	16.76	15.81	0.97
13.63	-3.41	16.82	7.84	16.76	24.98	3.05
16.86	0.26	15.82	8.89	16.77	32.69	4.87
26.01	6.18	16.05	11.13	16.78	49.61	4.79
19.09	-6.37	15.46	7.16	16.78	19.95	6.76
13.43	-3.17	15.95	7.93	16.84	25.31	6.37
17.59	-9.87	17.47	6.53	16.85	15.14	1.4
18.19	-9.89	17.4	6.54	16.88	15.08	1.87

23.74	-9.04	16.42	6.7	16.88	16.13	3.12
24.64	-1.74	15.22	8.37	16.88	28.05	8.62
17.17	-9.5	17.94	6.62	16.89	15.56	0.9
25.86	6.23	16.03	11.2	16.89	49.45	5.59
18.37	6.14	17.24	11.16	16.9	49.11	2.44
14.24	-0.9	17	8.62	16.92	29.76	3.06
24.48	-5.13	16.36	7.57	16.99	21.64	6.57
17.77	-0.4	15.81	8.81	17.02	30.67	6.85
21.32	-9.95	17.22	6.63	17.08	14.84	3.04
13.57	-3.33	16.16	8	17.08	24.62	6.9
19.12	-9.93	17.69	6.64	17.1	14.84	1.86
25.87	6.4	16.44	11.36	17.1	49.36	3.98
24.65	-5.19	16.33	7.62	17.1	21.39	5.96
24.64	-5.2	16.54	7.62	17.11	21.36	5.82
24.69	-4.97	16.33	7.67	17.11	21.75	4.94
13.84	-3.49	16.98	8	17.14	24.24	3.63
14.06	-2.58	16.67	8.25	17.15	25.92	3.53
20.7	-9.95	17.41	6.68	17.17	14.75	2.25
24.92	5.97	16.54	11.22	17.2	47.63	5.84
20.42	-5.49	16.14	7.56	17.2	20.78	6.16
18.99	6.21	17.54	11.32	17.21	48.4	2.39
22.26	-3.9	16.37	7.97	17.21	23.4	7.25
24.63	-5.62	16.14	7.57	17.22	20.56	6.28
16.07	3.62	17.99	10.27	17.22	40.4	1.58
22.73	-9.83	17.52	6.73	17.24	14.82	2.33
22.26	-9.45	17.37	6.81	17.25	15.26	2.43
21.81	-10.01	17.51	6.71	17.26	14.6	2.46
13.66	-3.37	16.32	8.08	17.26	24.28	7.16
25.6	6.29	16.7	11.4	17.3	48.38	4.76
25.32	6.02	16.81	11.29	17.31	47.46	4.71
17.99	-2.32	17.17	8.43	17.31	26.16	3.48
20.15	6.15	16.91	11.35	17.32	47.83	4.75
14.6	-0.75	17.39	8.86	17.33	29.32	3.32
23.13	-9.31	17.91	6.88	17.35	15.33	1.36
24.25	6.05	16.83	11.32	17.35	47.44	5.49
20.19	-9.5	17.74	6.86	17.38	15.08	1.97
22.9	5.94	17	11.29	17.39	46.95	4.87
25.77	6.34	16.9	11.46	17.39	48.27	4.07
13.71	-3.46	16.52	8.12	17.4	23.91	6.92
19.58	6.15	17.29	11.38	17.41	47.57	3.11
23.48	-4.75	16.57	7.87	17.41	21.67	6.7
23.79	-5.72	16.63	7.65	17.41	20.16	6.82
24.36	-4.66	16.74	7.89	17.41	21.84	5.95
22.41	6.08	17.1	11.37	17.42	47.33	4.23

18.54	-3.01	17.03	8.29	17.42	24.68	4.76
23.85	6.23	17.03	11.44	17.45	47.73	4.55
23.45	6.08	17.06	11.39	17.49	47.09	4.65
20.75	6.17	17.3	11.43	17.5	47.37	3.71
21.89	6.19	17.1	11.44	17.5	47.43	4.42
18.7	-0.37	16.45	9.03	17.5	29.82	6.45
21.32	6.13	16.94	11.43	17.51	47.22	5.01
24.67	6.28	16.97	11.48	17.51	47.71	4.35
19.8	-3.45	16.8	8.23	17.51	23.75	6.3
22.75	-4.54	16.7	7.96	17.52	21.88	6.58
16.27	3.57	18.11	10.38	17.53	39.47	1.88
13.8	-3.49	16.67	8.19	17.56	23.6	6.88
13.89	-3.54	16.83	8.18	17.56	23.53	6.62
20.89	-4.44	16.87	8.03	17.61	21.92	6.34
15.45	-2.06	17.41	8.6	17.62	26.16	4.11
14.98	-0.41	17.78	9.1	17.64	29.48	3.14
25.22	-1.81	16.42	8.7	17.64	26.61	8.54
25.13	-2.53	16.19	8.5	17.64	25.22	8.15
20.43	-3.71	17.4	8.26	17.7	23.02	4.25
16.39	3.48	18.25	10.44	17.73	38.71	1.91
15.65	-1.98	17.83	8.68	17.74	26.1	3.58
15.12	-2.36	17.47	8.58	17.75	25.36	4.37
14.04	-3.58	17.06	8.26	17.76	23.16	7.25
14.9	-2.83	17.25	8.45	17.76	24.49	5.4
15.26	-2.27	17.38	8.61	17.76	25.52	4.38
15	-2.62	17.25	8.51	17.77	24.84	4.85
21.52	-4.31	17.51	8.15	17.8	21.87	4.67
16.49	3.44	18.6	10.47	17.83	38.37	1.41
25.39	-1.01	16.61	9.02	17.84	27.86	8.31
16.67	3.49	18.82	10.5	17.86	38.41	1.61
14.75	-3.04	17.39	8.45	17.86	23.95	5.04
14.79	-3.06	17.2	8.44	17.87	23.91	5.31
19.63	-0.26	17.08	9.25	17.9	29.31	5.79
21.32	-6.55	16.85	7.67	17.91	18.32	6.36
14.07	-3.39	17.19	8.38	17.93	23.24	6.85
15.38	-0.3	18.19	9.27	17.94	29.16	3.32
25.33	-1.27	16.66	8.99	17.95	27.16	9.17
14.11	-3.39	17.18	8.41	17.97	23.18	7.1
14.13	-2.55	17.16	8.63	17.97	24.67	6.69
15.86	-1.87	17.96	8.82	17.98	25.93	4.34
14.7	-3	17.48	8.52	17.99	23.83	5.27
21.4	-7.19	17.18	7.59	18.02	17.33	7.18
16.97	3.62	18.7	10.62	18.03	38.37	1.85
14.45	-2.41	17.63	8.71	18.06	24.77	5.54

17.17	3.79	19.07	10.73	18.13	38.6	1.43
14.18	-2.53	17.69	8.72	18.17	24.39	5.4
14.27	-2.48	17.7	8.73	18.17	24.49	5.66
14.41	-2.39	17.71	8.76	18.17	24.65	5.59
14.53	-2.48	17.77	8.73	18.17	24.49	5.63
14.68	-2.82	17.65	8.65	18.17	23.87	5.58
25.37	-1.97	16.92	8.9	18.17	25.43	9.02
14.22	-3.19	17.25	8.56	18.18	23.23	7.51
22.46	-7.16	17.32	7.71	18.25	17.12	7.75
14.38	-2.42	17.72	8.8	18.27	24.43	5.89
14.59	-2.59	17.77	8.75	18.27	24.14	5.77
25.31	-0.97	17.19	9.23	18.28	27.19	9.29
20.46	-0.45	17.55	9.38	18.3	28.19	5.85
14.64	-2.76	17.77	8.73	18.3	23.79	5.75
15.85	-0.47	18.46	9.41	18.37	28.04	4.01
16.87	3.39	18.86	10.71	18.42	36.83	1.85
25.33	-2.28	17.68	8.96	18.49	24.36	9.54
23.5	-7.62	17.59	7.75	18.53	16.24	7.62
25.05	-1.88	17.68	9.14	18.64	24.86	9.35
16.35	-0.75	18.86	9.46	18.65	27	4.1
25.45	-1.15	17.69	9.34	18.65	26.22	8.14
16.34	-2.02	18.61	9.09	18.66	24.57	4.67
21.36	-0.62	18.09	9.51	18.7	27.16	5.84
17.5	3.89	19.81	11.02	18.72	37.44	1.2
22.87	-6.1	18.16	8.16	18.73	18.02	6.87
17.65	3.82	20.19	11.04	18.82	37.04	0.95
24.27	-1.7	17.94	9.28	18.84	24.87	9.92
24.79	-2.12	18	9.19	18.88	24.07	9.44
16.63	-2.22	18.92	9.18	18.97	23.75	4.81
22.2	-1.08	18.5	9.51	19	25.77	5.96
17.88	3.81	20.21	11.13	19.03	36.53	1.33
23.29	-6.35	18.38	8.25	19.04	17.33	6.79
17.48	-1.25	18.91	9.5	19.05	25.38	5.96
16.91	-1.6	19	9.4	19.06	24.73	4.63
16.85	-2.67	19.24	9.15	19.17	22.69	4.83
18.14	3.76	20.31	11.22	19.3	35.8	1.5
23.76	-0.81	18.85	9.73	19.32	25.76	6.48
23.2	-2.3	18.63	9.33	19.32	23.1	9.62
24.44	-7.41	18.51	8.18	19.34	15.68	7.38
18.16	-1.02	19.06	9.7	19.36	25.31	6.68
22.76	-1.7	18.84	9.53	19.38	24.05	8.16
-2.88	-14.91	-1.82	-4.91	19.43	34.97	1.01
17.09	-3.34	19.57	9.12	19.47	21.19	4.7
25.34	-7.71	18.97	8.21	19.53	15.15	7.61

23.6	-2.97	18.59	9.27	19.56	21.65	8.84
22.26	-1.35	18.94	9.71	19.58	24.39	8.07
18.77	-1.34	19.38	9.73	19.62	24.33	6.91
21.81	-1.4	19.18	9.73	19.66	24.17	8.42
25.27	-0.93	19.4	9.87	19.71	24.92	7
17.35	-3.88	20.1	9.1	19.71	20.04	4.03
21.39	-1.39	19.25	9.78	19.76	24.04	8.53
19.33	-1.43	19.57	9.78	19.77	23.95	6.95
20.41	-1.31	19.58	9.81	19.78	24.14	6.78
20.92	-1.36	19.41	9.8	19.78	24.05	7.81
24.66	-1.22	19.44	9.84	19.82	24.25	6.32
26.1	-7.74	19.57	8.34	19.82	14.84	6.46
26.02	-0.66	19.64	10.04	19.91	25.1	6.73
26.76	-6.95	19.91	8.61	20.07	15.53	6.05
27.77	-0.26	20.26	10.28	20.2	25.39	6.26
18.87	3.45	21.01	11.5	20.21	33.11	2.48
19.13	3.07	21.07	11.38	20.24	32.15	2.49
28.89	0.4	20.29	10.53	20.31	26.47	6.37
26.92	-0.15	20.15	10.38	20.33	25.4	5.29
27.34	-7.53	20.22	8.62	20.34	14.62	5.8
29.76	0.73	20.27	10.68	20.41	26.94	7.15
27.81	-6.95	20.34	8.77	20.42	15.2	5.87
18.03	-4.13	21.02	9.42	20.56	18.67	5.52
28.3	-7.37	20.61	8.77	20.62	14.54	6.04
30.19	0.48	20.72	10.73	20.7	25.99	6.5
18.98	-4.18	21.26	9.54	20.85	18.27	5.02
28.78	-7.53	20.85	8.88	20.91	14.11	6.45
19.69	3.01	22.13	11.65	20.92	30.7	1.74
29.85	-0.15	21.21	10.66	20.97	24.41	5.96
19.44	-3.36	21.6	9.82	21.04	19.19	4.22
18.66	-4.55	21.16	9.56	21.06	17.53	5.7
18.36	-4.45	21.3	9.6	21.12	17.6	6.1
29.18	-7.98	21.38	8.89	21.12	13.45	6.6
20	3.13	22.28	11.8	21.19	30.47	1.74
29.07	-6.07	21.14	9.3	21.19	15.5	6.05
29.22	-6.83	21.33	9.17	21.26	14.57	7.72
7.06	-4.31	10.92	4.5	21.32	34.97	0.9
19.99	-3.72	22.13	9.87	21.33	18.36	3.9
20.76	3.16	22.62	11.87	21.35	30.22	1.54
29.38	-6.55	21.45	9.3	21.42	14.74	7.73
18.35	3.55	20.76	11.2	21.5	34.98	1.24
29.51	-7.71	21.68	9.12	21.51	13.4	7.7
28.98	-7.4	21.73	9.18	21.52	13.72	8.09
30.75	0.51	21.66	11.12	21.6	24.63	5.28

31.27	0.32	21.94	11.11	21.69	24.18	4.81
20.65	-4.08	22.3	9.95	21.72	17.45	5.25
21.27	-4.35	22.46	9.98	21.91	16.91	5.99
28.76	-7.35	21.91	9.37	21.91	13.46	7.96
12.36	-6.78	13.47	5.09	21.92	25.03	0.91
28.91	-7.46	21.76	9.34	21.92	13.33	7.97
21.93	-4.34	22.56	10.05	22.06	16.75	6.9
28.95	-7.2	22.44	9.49	22.13	13.44	8.3
32.51	-0.12	22.85	11.18	22.16	22.74	4.09
22.5	-5.99	22.79	9.77	22.24	14.63	7.49
29.06	-7.1	22.25	9.58	22.28	13.41	7.61
28.92	-6.41	22.49	9.72	22.3	14.12	7.92
24.87	-8.59	22.58	9.32	22.32	11.92	10.75
28.75	-7.22	22.66	9.57	22.32	13.26	9.65
22.24	2.86	23.68	12.2	22.39	27.78	2.12
24.08	-7.33	22.8	9.59	22.42	13.07	8.8
23.09	-6.89	22.99	9.7	22.49	13.46	7.45
27.41	-5.95	22.78	9.89	22.49	14.45	9.2
28.6	-6.73	22.74	9.74	22.49	13.62	9.51
27.92	-6.62	22.87	9.77	22.52	13.71	9.59
32.8	-0.26	23.11	11.31	22.56	21.99	3.65
28.49	-6.94	22.79	9.74	22.57	13.33	10.32
23.62	-5.22	23.05	10.08	22.58	15.2	7.52
24.44	-6.77	22.84	9.77	22.6	13.49	8.54
26.26	-7.24	22.78	9.69	22.6	13.02	9.3
22.83	2.79	24.07	12.3	22.67	27.19	2.06
24.17	-5.88	22.98	9.99	22.68	14.37	8.44
31.91	-1.26	24.14	11.19	22.93	19.98	3.64
33.42	-0.98	23.51	11.27	22.94	20.38	3.83
23.45	2.7	24.26	12.38	22.96	26.52	2.54
34.27	-0.33	24.01	11.48	23.02	21.27	3.33
30.49	-2.58	24.28	10.94	23.14	17.9	3.28
24.09	2.47	24.52	12.39	23.16	25.79	2.71
33.95	-0.43	24.18	11.52	23.17	20.92	2.95
31.1	-2.25	24.76	11.06	23.24	18.24	3.05
27.1	1	24.27	11.97	23.25	23.1	4.43
30.85	-1.74	24.16	11.2	23.25	18.92	3.4
26.15	1.67	24.46	12.18	23.26	24.22	3.12
26.05	1.55	24.35	12.15	23.27	23.99	3.57
30.43	-3.28	24.51	10.85	23.32	16.8	3.45
24.71	1.83	24.7	12.25	23.33	24.39	2.7
25.56	1.8	24.46	12.25	23.33	24.32	3.15
26.69	0.95	24.4	11.99	23.35	22.88	3.38
30.72	-1.94	24.89	11.22	23.43	18.44	3.12

28.46	-0.81	24.73	11.56	23.52	19.93	3.75
25.2	1.7	24.73	12.3	23.53	23.88	2.33
29.8	-1.31	24.8	11.43	23.53	19.21	3.12
28.29	0.74	24.7	12.02	23.56	22.23	3.47
29.91	-3.45	24.88	10.93	23.59	16.33	2.73
28.87	-0.86	24.88	11.59	23.61	19.74	3.52
32.25	-0.92	24.63	11.62	23.71	19.55	2.88
29.33	-1.76	24.83	11.4	23.72	18.37	3.07
